# Herbal plants coordinate COVID-19 in multiple dimensions - An insight analysis for clinically applied remedies

**DOI:** 10.7150/ijms.50260

**Published:** 2020-10-22

**Authors:** Yuejian Zhang, Yibo Li, Xiting Wang, Rendong Qu, Juan Li, Tengteng Li, Tian He, Zheyi Wang, Yansong Liu, Xiangming Shao, Tao Lu

**Affiliations:** 1 The School of Life Science, Beijing University of Chinese Medicine, Beijing 100029, China.; 2 Beijing University of Chinese Medicine Affiliated Dongzhimen Hospital, Beijing 100700, China.

**Keywords:** COVID-19 pandemic, Ttraditional Chinese Medicine, anti-coronavirus, functional assessment, mechanistic insight

## Abstract

The use of multipronged measures, including traditional Chinese medicine (TCM), has greatly increased in response to the COVID-19 pandemic, and we found the use of TCM and is positively correlated with the regional cure rate in China (R=0.77, *P*<10^-5^). We analyzed 185 commonly administered TCM recipes comprised of 210 herbs nationwide to reveal mechanistic insight. Eight out of the 10 most commonly used herbs showed anti-coronavirus potential by intersecting with COVID-19 targets. Intriguingly, 17 compounds from the 5 most commonly used herbs were revealed to have direct anti-SARS-CoV-2 potential by docking with the two core structures [CoV spike (S) glycoprotein (6SVB) and CoV 3CL hydrolase (6LU7)]. Seven reported COVID-19 drugs served as positive controls; among them, retionavir (-7.828 kcal/mol) and remdesivir (-8.738 kcal/mol) performed best with 6VSB and 6LU7, respectively. The top candidate was madreselvin B (6SVB: -8.588 kcal/mol and 6LU7: -9.017 kcal/mol), an appreciable component of Flos Lonicerae. Eighty-six compounds from 22 unlisted herbs were further identified among 2,042 natural compounds, completing our arsenal for TCM formulations. The mechanisms have been implicated as multifactorial, including activation of immunoregulation (Th2, PPAR and IL10), suppression of acute inflammatory responses (IL-6, IL-1α/β, TNF, COX2/1, etc.), enhancement of antioxidative activity (CAT and SOD1), and modulation of apoptosis (inhibited CASP3). It is of interest to understand the biological mechanisms of TCM recipes. We then analyzed 18 representative remedies based on molecular targets associated with 14 medical conditions over the disease course, e.g., pyrexia, coughing, asthenia, lymphopenia, cytokine storm, etc. The significant level of coherence (SLC) revealed, in part, the potential uses and properties of corresponding TCMs. Thus, herbal plants coordinate to combat COVID-19 in multiple dimensions, casting a light of hope before effective vaccines are developed.

## Introduction

Coronavirus disease 2019 (COVID-19) and its outbreak have been a daunting challenge to global health. The early clinical features and epidemiology of analyzed cases, along with its many complications, have been a stark reminder of a dire need for systematic and multipronged measures, in addition to antiviral treatments.

Coronaviruses are in the orthocoronavirinae subfamily, which includes RNA-containing spherical viruses of the family Coronaviridae, including several viruses that cause acute respiratory illnesses and a few that cause serious illness, e.g., SARS, MERS coronavirus, and the novel coronavirus. SARS-CoV-2 is the coronavirus that caused the COVID-19 pandemic.

The pathological characteristics of the patients who succumbed to SARS-CoV-2 from hypoxemia and respiratory distress suggest that overwhelming immune damage occurs in the wake of prolonged cytokine storms 1, including hyperactivation of cytotoxic CD8 T cells 2, upregulation of proinflammatory Th17, IL6, GM-CSF, IFN, etc. 3, and the consequent desquamation of pneumocytes and formation of hyaline membranes, which cause ARDS. Thus, plasma levels of IL-6 are a predicted biomarker of pneumonia severity 4. Regarding the mechanism of infection, SARS-CoV-2 invades cells via the angiotensin-converting enzyme II (ACE2) receptor, in the same manner as SARS-CoV 5; however, the binding affinity of the SARS-CoV-2 spike (S) glycoprotein is 10 to 20 times higher than that of the SARS-CoV S glycoprotein 6. At the tissue level, ACE2 is mainly expressed in kidney, heart muscle, lungs, endothelium and gastrointestinal tract, based on the Human Protein Atlas (https://www.proteinatlas.org/ENSG00000130234-ACE2/tissue) and previous reports 7-9. Therefore, SARS-CoV-2 initially invades lung cells and is likely to further damage other systems as well, causing severe complications.

The current knowledge about the etiopathology of this new disease has triggered a global research race against time to develop therapeutic solutions, including vaccines and specific antiviral medicines. However, the clinically application of TCM remedies has resulted in the accumulation of abundant experience in combating epidemics before this pandemic. Specifically, taking Hu'nan Province as an example, with the implementation of TCM (from 57.24% to 94.93%), hospitalizations were shortened by more than 2 days on average, while the severe/critical rate dramatically decreased from 8.13% to 0% after TCM was widely implemented 10. A clinical study across 10 provinces including 701 confirmed cases showed a curative rate of more than 90% with Qingfeipaidu Decoction treatment 11. Another comparative study on 710 cases jointly conducted by more than 30 hospitals indicated an 8.8% reduction in mortality rate and a 4-day decrease in hospitalization length among severe pneumonic patients after combining regular treatment with Xuebijing Injection 11-13. Moreover, as of May 12, a total of 605 clinical trials to combat COVID-19 were registered in China, including 76 related to TCM remedies (Table S15 and Table S16).

Nevertheless, TCM works by targeting syndromes beyond pathogens, including fever, coughing, fatigue, dyspnea, expectoration, diarrhea, nausea, etc., which is difficult to comprehensively assess. Herein, based on an integrated algorithm, we analyzed the multiple mechanisms of herbs, including direct anti-coronavirus effects, and further described the functionality of TCM remedies on a molecular target basis.

## Methods

### Epidemiological correlation of COVID-19 curative rate and regional TCM usage

The outbreak map was generated based on statistical data from the National Health Commission of the People's Republic of China and from provincial and municipal health commissions. The total number of confirmed and cured COVID-19 cases and the mortality of COVID-19 were updated as of March 15, 2020. In addition, the TCM coverage map was based on the statistical data for COVID-19 that were openly accessible from the National Administration of Traditional Chinese Medicine (http://www.natcm.gov.cn/xinxifabu/gedidongtai/), as shown in Table S1. The Pearson correlation of regional TCM coverage and curative rate, as well as the associated P value, were calculated using R software. The difference between Hubei and non-Hubei regions was assessed by the cure rate (total number of cured patients divided by confirmed cases) and by the mortality rate within Hubei, non-Hubei provinces and nationwide as of March 24 (Table S2).

### Construction of the database of TCM remedies for COVID-19

All 185 TCM recipes we identified were from different sources; 125 (92 for treatment, 33 for prevention) were from different versions of *“Diagnosis and Treatment Protocol for COVID-19”* published by a national and local health commission that included TCM administration, 30 (18 from Sichuan, 9 from Hubei and 3 from Zhejiang Province) were recommended by local officials, 16 were from hospitals and research institutes, 6 were from renowned experts' specific formulations, 1 was from Miao medicine, 6 were from Tibet medicine, and 8 were from established TCM recipes. After removing the duplicates, 185 prescriptions with 210 herbs were included, as shown in Tables S3 and S4. In addition, some detailed information was also included in the database, e.g., regional source (references), compositions, basic prescriptions and applied phase.

### Overview and analysis of TCM remedies for COVID-19

#### The remedies were categorized based on TCM formulation principles

Over thousands of years, TCM has developed its own rules to formulate remedies. A famous rule called “*Jun-Chen-Zuo-Shi”* (“*monarch stratum principle”*) states that the status of different herbs in a remedy should be prioritized from *“monarch”*, *“minister”*, *“assistant”* and *“courier”*, in a similar manner as a functional kingdom. The *“monarch”* and *“minister”* herbs are the key elements of a remedy. In addition, a TCM remedy could be modified by adding or removing certain herbs. According to the composition of the 185 TCM remedies, combined with the knowledge of formulations, particularly the *“monarch stratum principle”* documented in the fourth edition of* “pharmacology of TCM formulae* (*Fang-Ji-Xue*)*”* from the Chinese TCM publishing house, edited by Li Ji, we extrapolated the basic recipes from each remedy based on the *“monarch”* and *“minister”* herb composition. Then, the occupation (%) of a remedy with a basic recipe was calculated based on the common herbs (the details are shown in the first 130 rows of Table S3). Orphan remedies without a *“monarch”* were excluded. A total of 130 remedies with 49 basic recipes (Table 2) were obtained, and the top 10 basic recipes surrounded by their derivatives were illustrated (Fig. 2A) using Cytoscape 3.6.2 software (http://www.cytoscape.org/).

#### Frequency analysis of the top 10 original-related derivatives over the disease course

The frequency of the basic recipes used was counted based on the application of their derivatives over the course of the disease. The course of the disease was determined from clinical indications, which were categorized as the preventive, developing, severe or recovery stage; in particular, different terms were used for database searching, such as “prevention”, “medical observation period” and “suspects” for the preventive stage; “mild”, “heat in the early period”, “acute stage”, “medium”, “influenza”, and “pneumonia" for the developing stage; “seriously ill”, “major episode”, “severe”, and “ICU” for the severe phase; and “recovery” and “convalescence” for the recovery stage.

#### Frequency analysis of the applied herbs among TCM remedies

Analysis of the frequency of all herbs used among 185 TCM remedies was performed using R software (Table S4).

### Retrieval of COVID-19 gene targets

The GeneCards database (https://www.genecards.org/) and NCBI database were used to identify COVID-19-related targets. In particular, we selected the species *“Homo sapiens”* and used “novel coronavirus/new coronavirus/2019 novel coronavirus/2019-nCoV/coronavirus disease 2019/novel coronavirus pneumonia/new coronavirus pneumonia/COVID-19” as keywords to retrieve COVID-19 targets. Targets from different searches were further combined after removing duplicates. To conform to the statistical strategy, these targets were all constrained with the entire TCMSP target database we constructed.

### Retrieval of core herb gene targets

Active ingredients and related targets from an herb were obtained primarily from the Traditional Chinese Medicine Systems Pharmacology Database (TCMSP) (http://tcmspw.com/tcmsp.php) 14. In addition, a few unlisted herbs were obtained from other sources, including the Traditional Chinese Medicine Information Database (TCMID) (http://119.3.41.228:8000/tcmid/search/) and the Encyclopedia of Traditional Chinese Medicine (ETCM) (http://www.tcmip.cn/ETCM/index.php/Home/Index/index.html) 15, which were further constrained within TCMSP. For instance, 19 targets of Gypsum fibrosum (Shi Gao) were found in TCMID, among which 12 were also identified in TCMSP (Shi Gao, 12/19). A similar overlapping methodology was also applied with other databases, such as the ETCM library, including Herba Dendrobii (Shi Hu, 67/95), Herba Rhodiolae (Hong Jing Tian, 93/186), Lophatherum gracile (Dan Zhu Ye, 75/95) and Radix Ophiopogonis (Mai Dong, 272/434). To be consistent, all the targets were constrained with TCMSP, in which at least half of each of the targets for an herb could be incorporated.

### The acquisition and unification with herb targets

The full name of gene targets and the ingredients in each herb were downloaded from TCMSP and were used to establish a database for TCM-related ingredients and targets. The conversion of the gene full name to gene symbols was carried out using the UniProt database (https://www.uniprot.org/) and R software.

### Database construction of all TCMSP-associated targets

A total of 3,339 targets were downloaded from TCMSP (http://tcmspw.com/browse.php?qc=targets), in addition to targets from 150 herbs listed in TCMSP (10 ethnomedicines and 50 rare herbs were not listed among the total 210 herbs). Finally, the targets were formatted using the UniProt database and R software, and a total of 3,439 gene symbols formed the whole target library.

### Target count and directionality of regulation of herbs

As each ingredient compound from an herb might modulate multiple gene targets, multiple ingredients could modulate a particular target multiple times; this frequency is also called the target count (TC). The higher the TC is, the greater the likelihood that the target gene could be modulated by an herb. We built a TC database of core herbs after merging the data for each herb and removing redundancy. The TC should be greater than zero for at least 6 out of 8 herbs, which would ultimately remain. One limitation for network pharmacology analysis was the directionality of the correlation; we attempted to add this critical information to each target gene via literature corroboration (Table S6). The heatmap was plotted with the Seaborn module in Python. Consistent literature reports of the positive regulation of a certain gene by an herb were assigned a positive TC value (red), and a negative regulation was assigned a negative TC value (blue). Controversial reports, no data, no regulation, and a TC value of zero were assigned to zero (white). The gene targets shown in white in more than 4 herbs out of 8 were eliminated. The color depth represents the absolute TC value. Table S5 reports the data in detail.

### Pathway analysis and illustration of the multiple effects of herbs on COVID-19

We then identified the integrated directions of these 8 herbs based on whether the target was inhibited or activated, as shown in the “Regulation” and “Total Target Counts” lines with fore-symbols (Table S5). Thirty-three upregulated targets (represented as “+”), 8 downregulated targets (represented as “-”) and 2 unconfirmed targets (IL2 and BCL2L1) were finally obtained. For instance, MAPK1 was activated only by HQi but inhibited by 5 herbs and two unlisted herbs; these data were be integrated to show inhibition. We further calculated the total target counts from 8 herbs (the addition of 8 herbs for each target) and connected them with specific directions (Table S5), e.g., PTGS2 (COX2) was -573, iNOS (NOS2) was -144, PARP1 was +3, IL10 was +13, etc. Forty-one targets with directions and total TCs were input into IPA (Ingenuity Pathway Analysis Software) to analyze the pathways regulated by these core herbs. Ten pathways, either activated (red bar) or inhibited (blue bar), were selected and are presented in Fig. 3C, according to the log10 P from IPA. Based on target and pathway analysis, we illustrated the multiple effects and potential mechanisms of these herbs against COVID-19 (Fig. 3D) using Pathway Builder Tool 2.0 (www.proteinlounge.com).

### The most commonly used herbs and their molecular potential to combat COVID-19

The top 10 most commonly used herbs were identified among the total 210 herbs. The COVID-19 targets were constrained with 3,439 TCMSP targets in total. The number of overlapping targets associated with each herb and COVID-19 were obtained by VENNY (https://bioinfogp.cnb.csic.es/tools/venny/index.html). We then generally assessed the anti-coronavirus potential of these 10 candidates by SLC (significance level of coherence), that is, -log10 P, which was calculated by the chi-squared test. Two herbs with low SLC were excluded.

### Exploration of direct anti-SARS-CoV-2 potential via molecular docking

There were two core structures of SARS-CoV-2 referred to our research, namely, the CoV spike (S) glycoprotein with a single receptor-binding domain (PDB ID: 6VSB) and COVID-19 3CL hydrolase, the main protease in SARS-CoV-2 crystal structure (PDB ID: 6LU7), which were obtained from Protein Data Bank (PDB) (http://www.rcsb.org/). In total, 919 ingredients of 8 herbs were identified from the TCMSP database, including GC Gan Cao from licorice glycoside; JYH (Jin Yin Hua) from Flos Lonicerae; LQ (Lian Qiao) from Fructus Forsythiae; HX (Huo Xiang) from Herba Agastaches; HQi (Huang Qi) from Radix Astragali seu Hedysari; XR (Xing Ren) from Semen Armeniacae Amarum; HQin (Huang Qin) Radix Scutellariae; and MH (Ma Huang) from Herba Ephedrae. We then obtained detailed molecular structure data (SDF file) by searching each corresponding molecular name on the PubChem website (https://pubchem.ncbi.nlm.nih.gov/).

We further developed a molecular docking analysis, which was performed using Schrödinger Small Molecule Drug Discovery Suite (SSMDDS) software. The receptors (6LU7 and 6VSB) were previously subjected to docking grids. We subsequently analyzed the docking efficiency between the two receptors and the ingredients on the ligand docking panel. Seven positive controls were prepared for this docking as well, namely, lopinavir, ritonavir, ribavirin, arbidol, remdesivir, chloroquine phosphate and favipiravir (T705). Then, we screened all 2,042 natural compounds from a TCM natural product library (http://www.biopurify.cn/), among which 1,971 ingredients had valid docking structures.

### Coherence analysis between TCM remedies and COVID-19-related medical conditions

First, we established a database for COVID-19-related symptoms, syndromes and medical conditions (SSMs). The molecular targets of major SSMs for COVID-19 were obtained from the NCBI (https://www.ncbi.nlm.nih.gov/gene/) and GeneCards (https://www.genecards.org/) databases. Pneumonia was introduced to serve as a positive control, while nausea served as the negative control. The COVID-19 targets shown in Fig. 3 were also included. Other keywords used for SSMs included SARS, coughing, pyrexia/fever, myalgia/muscle soreness, asthenia/fatigue, cytokine storm, oxygen saturation, nausea/vomiting, mucus/phlegm/sputum, lymphopenia/decreased lymphocyte, dyspnea/difficult breathing and diarrhea. The targets with correlation scores ≥ 20 in GeneCards and all targets in NCBI were combined after TCMSP filtering and duplicate removal.

Second, we selected the most commonly reported TCM remedies that were applied over the course of the disease. Seventy-six clinical trials referred to TCM remedies, and TCM patents were registered in the *Chinese Clinical Trial Registry* (http://www.chictr.org.cn/searchproj.aspx) for all 605 clinical COVID-19 trials. This includes QFPD (Qingfeipaidu decoction), HSBD (Huashibaidu decoction), LHQW (Lianhuaqingwen granule/capsule), XBJI (Xuebijing injection), RDNI (Reduning injection), etc. Few basic recipes were also included, e.g., SFT (Shenfu injection), SMI (Shenmai decoction), MXSG (Maxingshigan decoction), etc. Three medicines (3Ms) and three recipes (3Rs) were recommended by the NHC: JHQG (Jinhuaqinggan capsule), LHQW, XBJI; QFPD, HSBD and Xuanfeibaidu decoction). These were the effective remedies for combating COVID-19 identified in the accumulated clinical trials in China. One representative recipe for prevention was selected for further discussion, abbreviated as the expert name, LSY. In addition, other specific basic recipes were further considered, such as DYY (Dayuan decoction), the classic anti-epidemic recipe; ECT (Erchen decoction), the base for all phlegm recipes; and SJZT (Sijunzi decoction), the basic energy-boosting recipe. HXZQ (Huoxiangzhengqi Powder), YPFS (Yupingfeng Powder) and YQS (Yinqiao Powder) were the representative recipes used across the preventive, developing and recovery stages.

As mentioned above, the targets of each herb obtained from TCMSP were combined within a remedy after removing duplicates. VENNY was used to identify targets from a remedy that intersected with one SSM; the SLC for each intersection was calculated by the Chi-square test. Finally, a heatmap was created based on the SLC value, represented as -log10 P.

## Results

### Regional promotion of TCM was positively correlated with the curative rate of COVID-19

**Figure 1:**
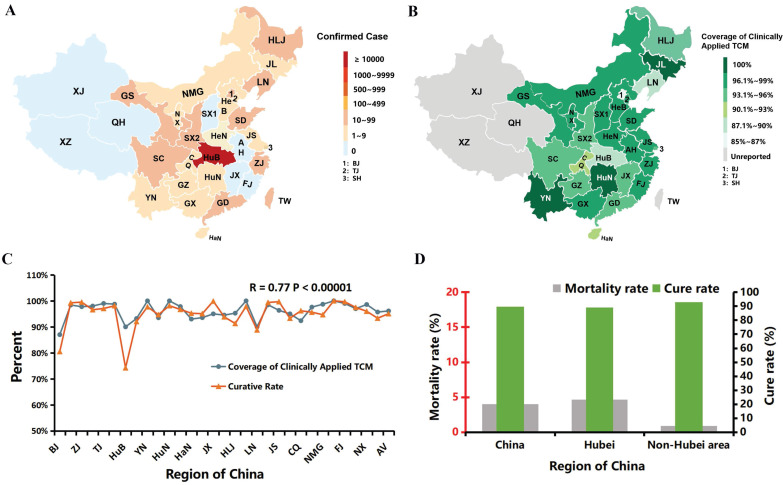
** The positive correlation of the curative rate of COVID-19 and the prevalence of regionally applied traditional Chinese medicine (TCM) in China.** (Data source: National Administration of TCM.) (**A**) An epidemic map of China as of March 15. Xinjiang (XJ), Qinghai (QH), Xizang (XZ) and a few other provinces were free of COVID-19 (gray - blue). Other affected regions are marked with red tones, and the depth of color represents the number of remaining confirmed patients (Table S1). (**B**) A TCM coverage map of China. The depth of green positively correlates with the TCM coverage rate. The abbreviations of the corresponding provinces were the same as in Fig. 2A, except BJ (Beijing), TJ (Tianjin) and SH (Shanghai), which are named 1, 2 and 3, respectively, due to the limited space on the map. Table S1 shows the details. (**C**) Correlation curve between regional curative rate and TCM coverage rate (R=0.77, *P*<10^-5^). Table S1 presents the details. (**D**) Comparison of Hubei with non-Hubei regions to the cure rate as of March 24 is pictured in green, while the mortality rate is in gray. Concrete data are shown in Table S2.

As of March 15, China had registered more than 82,000 confirmed cases of coronavirus and approximately 3,300 deaths, whereas over 76,000 infected individuals have been cured, with a curative rate of more than 92% across the population. The majority (67,781) of infections were in Hubei Province (HuB), as shown in Fig. 1A and Table S1. Outside HuB, many regions adopted primo TCM remedies after the outbreak. The official epidemiological data were gradually published, and it is intriguing to that the regional curative rate is robustly correlated with the local promotion of TCM (R=0.77, p value<10-5), as shown in Fig. 1B & C. The average regional TCM coverage is 96.12%, with the highest coverage in Yunnan (YN), Hu'nan (HN) and Jilin (JL) provinces and the lowest coverage in Beijing (BJ), Hubei (HuB) and Liaoning (LN) provinces (Fig. 1B, C). Hubei (HuB), the hardest-hit province, accounted for more than 82% of diagnoses and 96% of all deaths in China. The average mortality rate outside HuB was 0.88% versus 4.66% in HuB (Fig. 1D). Almost 200 regional remedies have been applied countrywide, which significantly shortened the disease course and reduced the conversion to ICUs (intensive care units). Zhejiang Province, one of the earliest regions that applied TCM, had more than 1200 diagnoses but only 1 death (mortality rate 0.08%). Based on these data, TCM has played a remarkable role in pulling China through this COVID-19 crisis when virtually no other effective medicines were available in the initial two months.

Thus, it is of particular interest to understand the underlying mechanisms of these TCM remedies and is also intriguing to partly classify the self-contained TCM theories in contemporary molecular biology.

### Insight analysis of TCM remedies based on the composing principle

A total of 185 TCM recipes were collected from different sources (the details are shown below in the Methods section), mainly originating from the Diagnosis and Treatment Protocol for Novel Coronavirus Pneumonia published by the National Health Commission (NHC). We then analyzed these recipes nationwide (Table S3), categorizing them based on the representative TCM principle.

**Figure 2:**
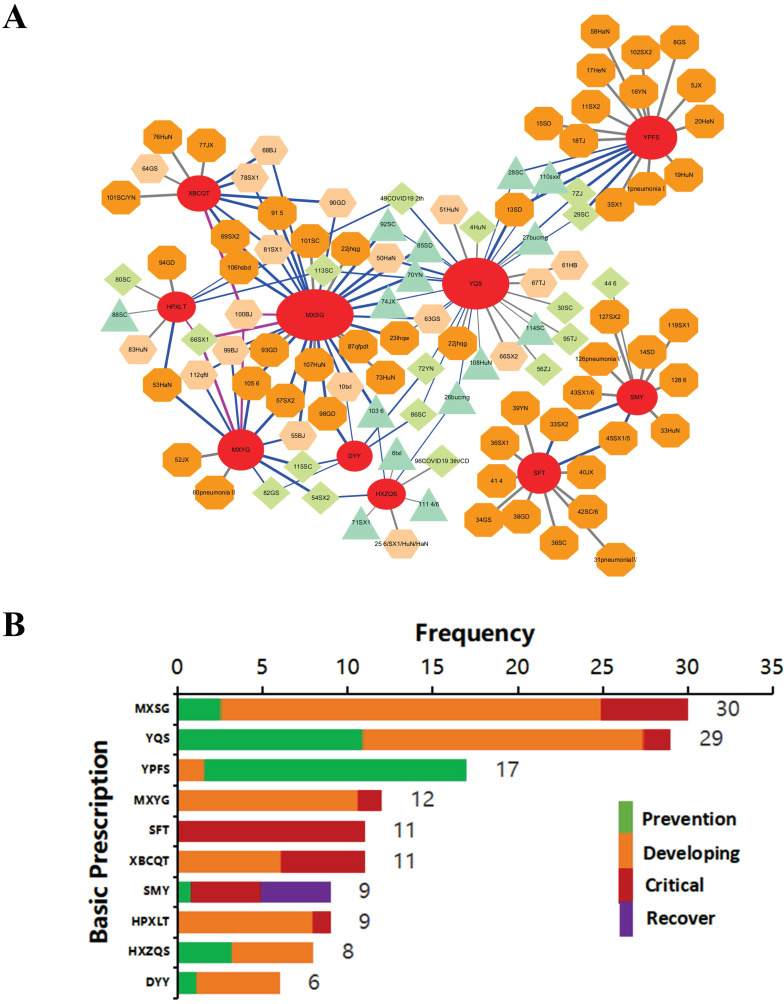
** The lineage of the originals to their derivatives with applied courses of the most commonly based recipes.** (**A**) A total of 130 basic recipes were extracted from 185 clinically applied recipes, according to TCM composition principles, with numbers assigned from 1 to 130 (the complete dataset is in Table S3). The relational network of the top 10 basic recipes and clinical TCM remedies are shown there. The red oval hubs represent original core recipes, which are labeled with acronyms, e.g., MXSG means Maxingshigan Decoction. The surrounding circles are derivatives arranged by similarity to their basic recipe (20%-100%), where deep orange octagon (100%), thin orange hexagon (75-100%), green diamond (50-75%), and turquoise triangle (<50%). The color of the connecting line represents the combination of basic recipes: gray for single, blue for two, and purple for three combined recipes. Those remedies applied in COVID-19 were named as described below. (1) The regional abbreviation was named after the number; for instance, 51HaN indicates recipe #51 from Hainan Province. (2) If the recipe was issued from a certain version (X) of the treatment protocol issued from the National Health Commission (NHC), X is added, such as 41 4. (3) Multiple origins are all shown with '/', such as 25 6/SX1/HuN/HaN. (4) Lowercase abbreviation represents other features, except regions and version number, including clinical effective recipes from individual or local application, such as 10txl from one expert, whose name is Tong Xiaolin, or 87qfpdt (Qingfeipaidu Decoction), the effective clinical recipe with more than 90% curative rate, etc. (5) Agreed COVID-19 prescriptions from some hospitals were translated following their meanings, e.g., 1 pneumonia I. (**B**) The top 10 basic recipes were categorized as applied courses, e.g., early-preventive (green), mild-moderate-developing (orange), severe-critical (red), and recovery stage (purple). The number indicates the frequency among all recipes. The counts within 4 stages of those top 10 basic recipes are shown in Table 1.

Over thousands of years, TCM has evolved its own theories to formulate a recipe. A famous one called the *“monarch stratum principle” (Jun-Chen-Zuo-Shi)* ranks and analyzes the status of different herbs in a recipe, including *“monarch”*, *“minister”*, *“assistant”* and *“courier”* herbs, analogous to a functional kingdom. The *“monarch”* and *“minister”* herbs are usually the core components of a recipe. In addition, a TCM recipe could be modified by adding or removing certain herbs or recipes to address various syndromes. Forty-nine basic recipes were identified (Table 2), and the origins of 130 of the 185 TCM recipes could be identified (Table S3) via similarity analysis following the *“monarch stratum principle”*. The top 10 were applied to insight analysis, as shown in Fig. 2A, B. MXSG decoction, the largest red oval hub, was the most commonly used as it has 30 derivatives. The similarity of derivatives to the original recipe is presented as different shapes and colors. Some recipes were the combination of two or three original recipes, as indicated by blue or purple lines (Fig. 2A).

TCM is a de facto precision medicine in which the recipe formulation is adjusted according to different stages of diseases. The top 10 basic recipes with derivatives are shown in detail (Fig. 2B and Table 1). For instance, Yupingfeng Powder (YPFS) was applied mainly in the early preventive stage (15 out of 17 remedies), with the goal of promoting immunity 16. Maxingshigan Decoction (MXSG), on the other hand, was primarily administered in the developing stage (22 out of 30) for antipyretic detoxification purposes 17, as was Yinqiao Powder (YQS), a well-known antipyretic/anti-exo pathogenic remedy 18. Shenfu Decoction (SFT), however, was only used in severe/critical patients (all 11), aiming to boost energy in recuperation 19. Similarly, Shengmai Decoction (SMY) was applied mainly in the recovery and severe/critical stages, as it specializes in regenerating the metabolism of body fluids 20. Moreover, the TCM formulation is strategized based on the stratification of the disease, e.g., exogenous pathogens started from the surface and then deepened inside amid exacerbation. According to this *“Stratification Principle” (“Wei-Qi-Ying-Xue”)* of TCM, the classical composing criterion for epidemics specifically, YQS was contrived to dispel exogenous pathogens over the surface, while Maxingyigan Decoction (MXYG) was focused on dispelling dampness or phlegm over expectoration 21. Xuanbaichengqi Decoction (XBCQT) was conceived to promote the discharge of lung toxins via the intestine 22. Moreover, Huopuxialing Decoction (HPXLT) and Huoxiangzhengqi Powder/Granule (HXZQS) were designed to block the entry of exogenous pathogens via the digestive system 23, 24. Finally, DYY, a specialized anti-epidemic remedy, has been applied successfully in H1N1 and SARS outbreaks 25.

To flexibly and systematically combat SARS-CoV-2, some modifications have been made based on these ancient empirical anti-epidemic remedies. The intermixed remedies and their lineages are presented in Fig. 2A. Some of the derivatives have proven more capable of treating COVID-19-related symptoms and syndromes than their precursors, which were assessed further on a molecular basis (Fig. 5).

### Exploratory study of the molecular functionality of the most common herbs against COVID-19

**Figure 3:**
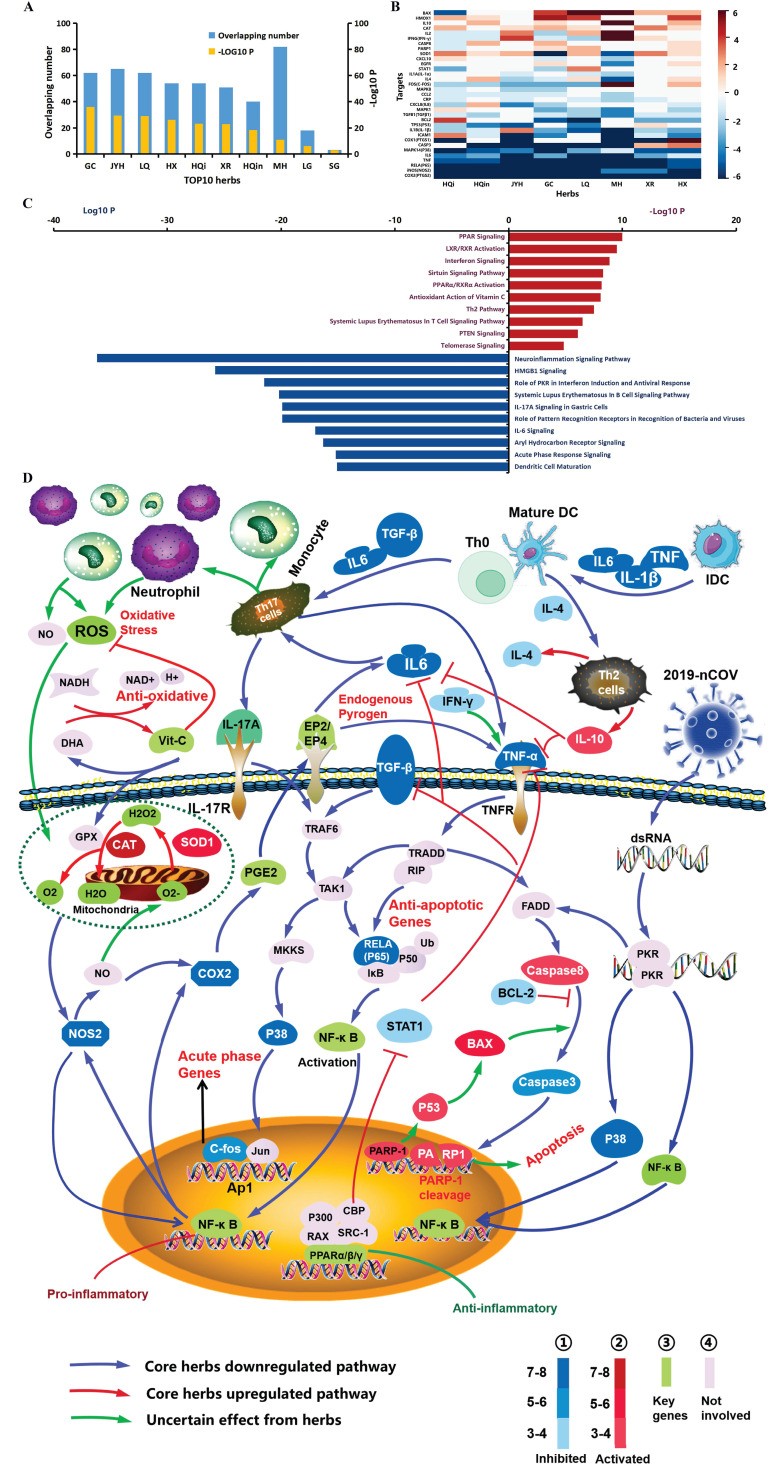
** Functional analysis and potential mechanisms of the top commonly used herbs.** (**A**) Intersection of the top 10 herb targets with COVID-19 targets. All the target analyses were constrained within the TCMSP database. The number of overlapping targets is shown in blue, while the significance (-Log10 P) of the coherent intersection is shown in orange. The most commonly used herbs are represented by acronyms. GC: licorice glycoside, Gan Cao; JYH: Flos Lonicerae, Jin Yin Hua; LQ: Fructus Forsythiae, Lian Qiao; HX: Herba Agastaches, Huo Xiang; HQi: Radix Astragali seu Hedysari, Huang Qi; XR: Semen Armenia cae Amarum, Xing Ren; HQin: Radix Scutellariae, Huang Qin; MH: Herba Ephedrae, Ma Huang; LG: Rhizoma Phragmitis, Lu Gen; SG: Gypsum Fibrosum, Shi Gao. (**B**) Heatmap of herb targets. The depth of color, as indicated in the number bar, represents the frequency of a target gene with compounds from a corresponding herb, the so-called target count (TC). A TC of more than 6, e.g., 112 TC of COX2 (PTGS2) to GC, were unified (the original data are shown in Table S5). Red represents positive regulation, blue represents negative regulation, and white represents no change, multiregulation or lack of reports, which were determined from the literature (Table S6). (**C**) Pathway analysis with directions of herb targets. Ten up- and downregulated pathways by 8 herbs, according to Log10 P using IPA, are shown. The red bar indicates activation, while the blue bar shows inhibition. The crucial identified pathways are shown in the diagram (Fig. 3D), including the activated pathways (“PPAR signaling”, “interferon signaling”, “PPARα/RXRα activation”, “antioxidant action of vitamin C” and “Th2 pathway”) and the inhibited pathways (“pole of PKR in interferon induction and antiviral response”, “IL-17A signaling”, “IL6 signaling”, “acute phase response signaling” and “dendritic cell maturation”). (**D**) Pathway diagram presenting the potential molecular mechanisms by which the core herbs impact COVID-19. The key targets modulated by both herbs and COVID-19 were classified into 3 categories, according to the results of Fig. 3B: inhibited (blue tones), activated (red tones) and not involved in herbs (discussed key target genes are presented in green, while the general in purple). The depth of color varies as the amounts of same regulation from 8 herbs. Pathways were divided into three categories: downregulated with herbs in blue, upregulated with red and uncertain with green.

We further evaluated the 210 herbs from all 185 recipes based on their usage frequency (Table S4). Fifty-one herbs within more than 10 formulas are listed in Table S4. The top 10 most commonly used herbs were further evaluated for their anti-pandemic potential on a molecular target basis via intersection with COVID-19 targets (Fig. 3A). A total of 352 potential targets that correlated with COVID-19 were identified from the NCBI and GeneCards libraries, and 172 remained after constraining with the entire TCMSP database, which contains all 3,439 targets regulated by herbs (Fig. 5A). The following 8 out of 10 herbs had a high significant level of coherence (SLC), equally used in -Log10 P, indicating the potential for these herbs to impact COVID-19: Flos Lonicerae (GC), Radix Glycyrrhizae (JYH), Fructus Forsythiae (LQ), Herba Agastaches (HX), Radix Astragali seu Hedysari (HQi), Semen Armeniacae Amarum (XR), Radix Scutellariae (HQin) and Herba Ephedrae (MH).

We then asked what the specific functionality of this regulation could be. As each ingredient compound could modulate multiple target genes, multiple ingredients from an herb could regulate multiple a particular target gene multiple times, the so-called target count (TC). First, we calculated the TC of the 8 herbs. To some extent, the higher the TC is, the more likelihood the target would be modulated by an herb. After merging data and removing duplicates, the final TC matrix for 8 herbs contained 53 targets, which required TC > 0 for at least 6 herbs. We determined the directionality of this regulation afterwards. The positive or negative regulation of every coronavirus-related gene target by each herb was corroborated via literature verification (Table S6). The summary of the coronavirus-related targets contains 33 targets, which were consistently associated with the top 8 herbs, as shown in the heatmap (Fig. 3B) and detailed in Table S5. The TC was indicated by the depth of color. For each gene target, the dominant number of herbs with either activating or inhibitory effects defined the regulatory status of a gene by all 8 herbs: 24 genes were primarily inhibited (blue colors), 8 target genes were activated (red colors), and one was uncertain (IL2: 3 inhibitions, 3 activation and 2 have yet to be reported). Pathway analysis was assessed further using IPA (Fig. 3C). “PPAR signaling”, “interferon signaling”, “PPARα/RXRα activation”, “antioxidant action of vitamin C” and “Th2 pathway” were the main upregulated pathways, while “role of PKR in interferon induction and antiviral response”, “IL6 signaling”, “acute phase response signaling”, “IL17A signaling” and “dendritic cell maturation” were the downregulated pathways (Fig. 3C, D).

Intriguingly, the target genes related to proinflammation, including COX2 (PTGS2), iNOS (NOS2), IL6, RELA (P65), TNF and MAPK14 (P38), were mainly inhibited, especially in response to treatment in the acute phase, which was consistently inhibited by all 8 herbs with high TC (dark blue). The other genes were primarily suppressed by most of the herbs, including COX1 (PTGS1), IL-1β, IL-1α, CRP, ICAM1, CCL2, MAPK1 and FOS (C-FOS). The herbs were found to have mixed effects on other genes; for example, IL4 was inhibited by four herbs and activated by three herbs, and IL2 was regulated in both directions by an equal number of herbs (3/3) with some dark red color (activated) by the top listed herbs. However, an opposite cohort, primarily involved in immunoregulation and antioxidation, such as IL10, HMOX1, CAT and SOD1, was activated by the core herbs. Furthermore, apoptosis plays a central role in the immune system, balancing the battle between viral infection and collateral damage from inflammation. Herbs modulated apoptosis from multiple dimensions; specifically, proapoptotic BAX was activated by five herbs and inhibited by one herb, while antiapoptotic Bcl2 was inhibited by four herbs and activated by one of the top listed herbs; caspase-8, which initiates the apoptosis cascade, was activated by three and suppressed by one, while caspase-3, the terminal downstream caspase, was inhibited by five herbs with high TC and activated by two herbs; PARP-1 cleavage, the signature of apoptosis induced by caspases, was upregulated by three herbs and downregulated by one herb (Fig. 3B).

Thus, we tried to illustrate how the core herbs coordinate the above targets and pathways. The diagram is presented in Fig. 3D. The replication of SARS-CoV-2 depends on the insertion of its double strained RNA (dsRNA) into polymerase (RdRp), which is the antiviral strategy of remdesivir 26. Eight of the candidates similarly targeted dsRNA to decrease virus replication and subsequent antiviral inflammation by downregulating the “role of PKR in interferon induction and antiviral response” pathway, thereby inhibiting P38/NF-κB. IL6, IL1, TNF-α, TGF-β and IFN-γ, endogenous pyrogens, leading to high pyrexia. The expression of these genes was decreased (“acute phase response signaling”/“IL6 signaling” pathway: NF-κB → COX2 → PGE2 → EP2/EP4 → IL6/IL1/TNF-α/TGF-β/IFN-γ), and antagonistic pathways were activated (“Th2 pathway”/“PPAR signaling”: Th2 cells → IL10 - IL6/TNF-α and PPAR - STAT1 - TNF-α/TGF-β/IL6) by herbs, which was the potential molecular mechanism against fever (also as shown in Fig. 5B). Dendritic cell maturation is also important for cell-mediated immunity, which promotes the differentiation and maturation of naive T cells and recruits many cytokines, neutrophils, monocytes, mast cells and eosinophils to combat the invaded virus. However, an overactivated immune status and even a severe cytokine storm will occur, one of the fatal mechanisms of COVID-19, when the immune system is out of balance or hard to eliminate. Most herbs downregulated dendritic cell maturation (Fig. 3C, D) by suppressing IL6, TNF and IL-1β (deep blue), the major mediators of the development of immature dendritic cells (IDCs) to dendritic cells (DCs). Furthermore, oxidative damage due to the accumulation of reactive oxygen species (ROS) and nitric oxide (NO) occurred during the epidemic course with enhanced collateral damage, including cardiac injury and multiple organ failure 27. A potential antioxidative therapy (e.g., vitamin C) was recommended to decrease the damage caused by COVID-19 28. Interestingly, the “antioxidant action of vitamin C” pathway was activated, which could certainly decrease the oxidative stress induced by ROS, including hydrogen peroxide (H_2_O_2_), superoxide (O^2-^), hydroxyl radical (OH), and singlet oxygen (O_2_), as shown in Fig. 3D circled by a dotted green line. In the circle, regulators of ROS were activated (Fig. 3D), including both CAT and SOD1, which promoted self-circulation to moderately decrease proinflammatory byproducts (ROS → NOS2 → NF-κB → COX2 → IL6 → ROS).

### Exploration of herbs with direct anti-SARS-CoV-2 potentials via molecular docking

**Figure 4:**
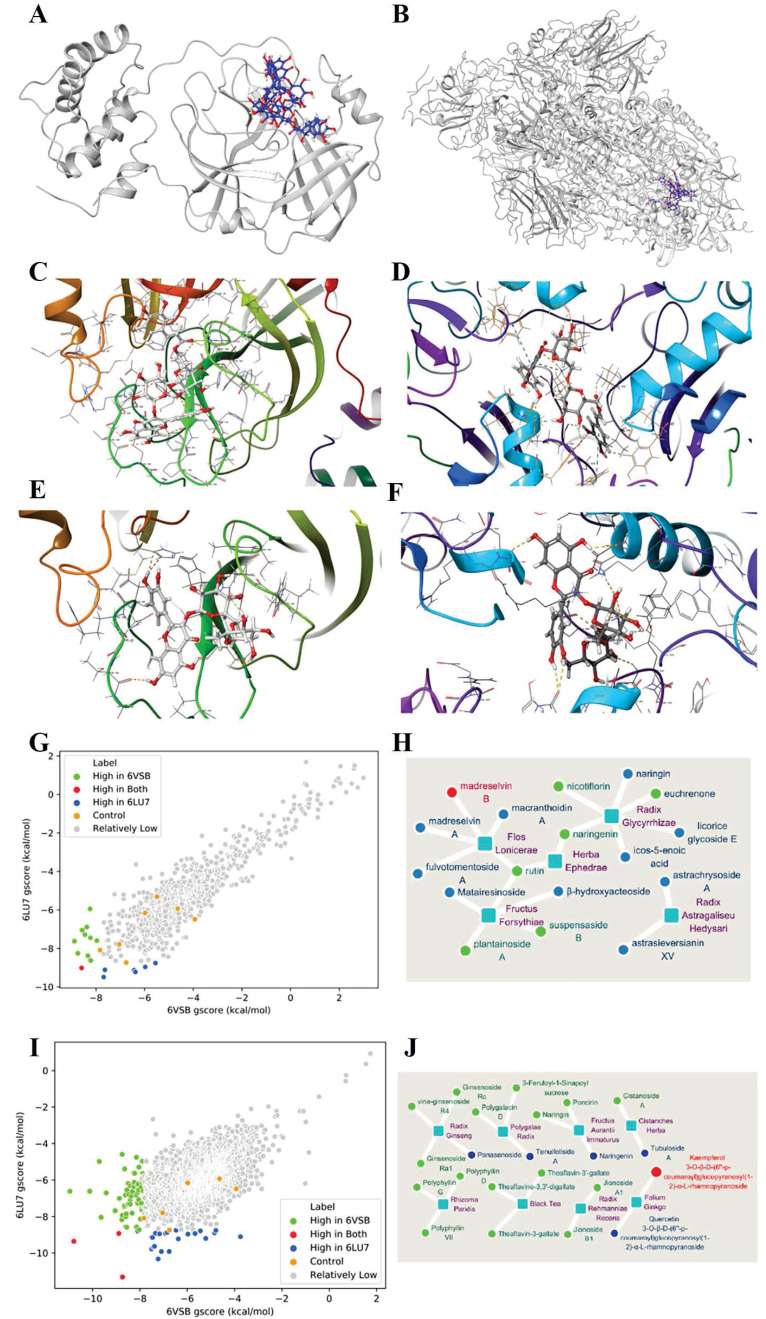
** Molecular docking between herbs and the two crystal structures of SARS-COV-2 proteins (PDB ID: 6LU7/6VSB).** (**A**) The structure diagram of 6LU7 (gray) with its pocket, which is shown by madreselvin B and rutin (blue with red). (**B**) Diagram of 6VSB (gray) and its pocket binding with madreselvin B and rutin (blue to red). (**C**) The concrete binding of madreselvin B with 6LU7 (CID: 44258007), Glide gscore= -8.383 kcal/mol. (**D**) The binding of madreselvin B with 6VSB (CID: 44258007), Glide gscore= -8.588 kcal/mol. (**E**) The docking configuration between rutin and 6LU7 (CID:5280805), Glide gscore= -9.225 kcal/mol. (**F**) The docking configuration between rutin and 6VSB (CID:5280805), Glide gscore= -6.377 kcal/mol. (**G**) Docking results of 919 unique compounds from the 8 most commonly used herbs. Every point means one compound. Seven antiviral drugs served as controls (orange), including remdesivir, ritonavir, lopinavir, arbidol, ribavirin, chloroquine phosphate and favipiravir. Compared with the controls, the compounds with better docking efficiency to 6LU7 (blue), 6VSB (green), or both (red) and with worse docking efficiency (grey) are shown. (**H**) A map of herbs and the ingredients with the highest potential among 919 ingredients from 8 herbs. The core squares in light blue are 5 herbs, and the surrounding circles are the 17 potential compounds (details in Table S7). This includes better docking with either 6LU7 (blue), 6VSB (green) or both (red). Herbs here named by each Latin name in purple, that is, Flos Lonicerae (JYH, Jin Yin Hua), Fructus Forsythiae (LQ, Lian Qiao), Radix Astragali seu Hedysari (HQi, Huang Qi), Radix Scutellariae (HQin, Huang Qin), Radix Glycyrrhizae (GC, Gan Cao) and Herba Ephedrae (MH, Ma Huang). (**I**) Preferable docking results of the 2042 additional natural compounds from a natural compound library. Table S9 shows the details. The figure details are similar to those in Fig. 4G. (**J**) Top herbs containing compounds that bind to both CoV structures among the 2042 natural compounds. The legend is similar to the Fig. 4H legend.

Then, we asked whether these top 8 herbs had direct anti-coronavirus effects. We first screened 919 unique compounds from these herbs, and we calculated their docking energy to the newly determined 3D structures of two proteins that are certainly related to COVID-19 function, namely, the CoV spike (S) glycoprotein (6VSB) and COVID-19 3CL hydrolase (6LU7), which is shown in detail with the docking pockets (Fig. 4A, B). The docking energy represents the binding affinity of one compound to the pocket of the core structure of another protein, which refers to its basic function. For the docking results, lower Glide G scores indicated better ligand-protein binding affinity. The results are shown in Fig. 4 and Table S7. Because a specific medicine for COVID-19 has yet to be developed, no ingredient or drug could be used for comparison. Thus, some antiviral drugs with potential anti-SARS-CoV-2 effects, including remdesivir 29, 30, ritonavir 31, lopinavir 32, 33, arbidol 33, 34, ribavirin 35, chloroquine phosphate 36 and favipiravir 35, which were employed as positive controls.

Curiously, 6 (green dots) and 10 (blue dots) compounds docked to 6LU7 and 6VSB, respectively, with higher efficiency than the controls (orange dots), indicating the anti-coronavirus potential of these 8 herbs (Fig. 4G, Table S7). Suspensaside B, the active compound of LQ, had the best 6LU7 docking score among the compounds (-9.491 kcal/mol), better than the best control (remdesivir, -8.738 kcal/mol), while madreselvin A from JYH had the best 6VSB docking score (-8.88 kcal/mol), better than the top control (ritonavir, -7.828 kcal/mol). Intriguingly, a compound called madreselvin B (6VSB: -8.588 kcal/mol, 6LU7: -9.017 kcal/mol), which originated from JYH, targeted both structures with high efficiency (Table S12; the docking configuration is shown in Fig. 4C, D). Rutin was the only common compound found in 3 herbs (LQ, MH and GC) with high (the second lowest gscore) 6LU7 binding efficiency but moderate 6VSB binding efficiency. The docking configurations of rutin to both structures are shown in Fig. 4E and F.

Each herb harbors multiple compounds, making them appealing for anti-coronavirus therapies. Moreover, 5 out of the 8 top herbs contain ingredients that can target either 6LU7 or 6VSB or both, as summarized in Fig. 4H and Table S7. These herbs are JYH (madreselvin B/A, rutin, etc.), LQ (suspensaside B, plantainoside A, matairesinoside, etc.), GC (rutin, licorice glycoside E, etc.), MH (rutin, naringenin, etc.) and HQi (astrachrysoside A, etc.). In accordance with Fig. 3A, which shows target genes, these five herbs were also found to have high SLC with COVID-19, except for MH, which had the most overlap amount but a low SLC, which might be related to its large base number (Table S4).

Thus, these very frequently used herbs might have strong anti-coronavirus potential. These results provided underlying mechanisms for the clinical observations that TCM recipes have significantly shortened the disease course and lessened the severity of the epidemic 13.

We then asked whether there were unlisted herbs with anti-coronavirus potentials as well. The docking of all 2,042 natural compounds with the two SARS-CoV-2 proteins was further examined, as shown in Table S11.

Interestingly, 61 (green dot) and 22 (blue dot) natural compounds were found to dock better to 6LU7 and 6VSB than the best control for each protein (Fig. 4I, Table S9). The good news was that three natural compounds showed promising binding to both (Table S12), as shown in Fig. 4I (red dot): 5-methoxypinocembroside (5 MPB), kaempferol 3-O-β-D-(6''-p-coumaroyl) glucopyranosyl (1-2)-α-L-rhamnopyranoside (KGR) and 1,2,3,4,6-pentagalloylglucose (PG). These compounds were mainly from GHC (Herba Penthori), YX (Ginkgo biloba) and WBZ (Galla Chinensis). Fig. 4J maps herbs and their ingredients (Table S10 in detail). The herbs included are Cistanche salsa (CS), Ginkgo biloba (GB), Chrysanthemum indicum (CI), ginseng (GS), Fructus aurantii immaturus (FAI), Fructus aurantii (FA), etc. Some of them (GB, GS, FA, CS) have been documented to be capable of promoting anti-inflammation, enhancing immunity, boosting circulation and combating pathogens 37-39.

Consequently, the identification of herbs with high efficiency docking to SARS-CoV-2 enriched our arsenal to combat COVID-19.

### Molecular biology assessments of clinically applied TCM recipes and some original remedies

TCM recipes are formulated based on self-contained theories, aiming to coordinate body balance, often modulated with symptoms, syndromes and medical conditions (SSMs). The development of modern medical science has been able to elaborate SSMs down to the molecular or pathway level. In addition, the herbs within a recipe have been concatenated functionally with a spectrum of molecular targets. Therefore, it is possible to evaluate the functionality of a recipe via the language of molecular biology.

**Figure 5:**
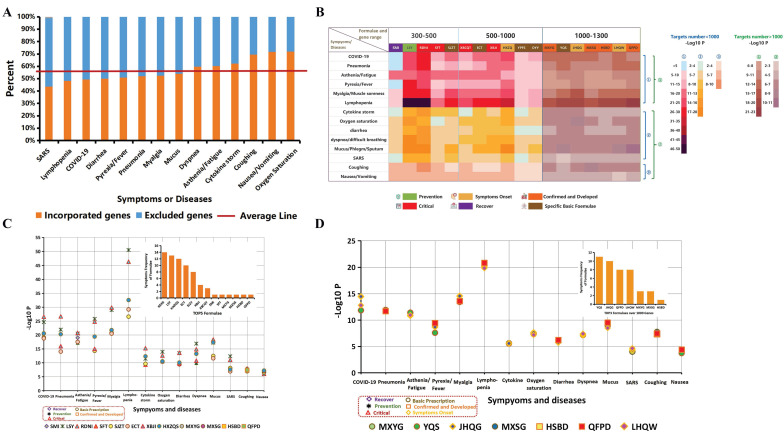
** Panorama overview of clinically applied TCM remedies in relationship to medical conditions based on molecular target analysis.** (**A**) SSMs (symptoms, syndromes and medical conditions) involved in COVID-19 constrained with the TCMSP database. Fourteen SSMs related to COVID-19 throughout the whole course are shown on the X axis, while the percent constrained with the TCMSP entire database is shown on the Y axis. Incorporated targets are orange, and blue reflects excluded targets. The average of incorporated genes was 56.88%, shown by the red line, which indicates that more than half of the targets were fit into the research. The raw data are shown in detail in Table S13. (**B**) Significance level of coherence (SLC, -Log10 P) of target intersections between remedies and SSMs are shown in each pixel. SSMs are shown on the left. Remedies are shown above and are divided into 3 scopes, 300-500, 500-900 and 900-1300, indicating the target number. The depth of color represents the value of SLC (-log10 P). The legend is shown on the right; the blue notes indicate fewer than 1000 targets, while the green notes indicate more than 1000 targets. Colors covering different remedies indicated the corresponding course and whether it was from original or clinical remedies, including green was prevention, yellow was symptom onset, orange indicated the confirmed and developed, red was critical stage, purple was the recovery stage, and brown represented some specific basic recipes. These are all shown at the bottom of the heat map. The raw data are presented in Table S14. (**C**) The top 5 remedies with the best SLC for each SSM are shown. These top 5 formulas that interacted within 14 SSMs were ranked in the upper right corner. Every point refers to one remedy whose shape and line color means which course it was. Specifically, the formulas represented by purple diamonds were used for the recovery stage, e.g., SMI (Shengmai injection); green asterisks were used for prevention, e.g., LSY (the preventive prescription from Liu Shangyi TCM master); orange squares were used for the confirmed and developed stages, e.g., HSBD (Huashibaidu decoction); red triangles were used for to the critical stage, e.g., XBJI (Xuebijing injection); and the brown circle represents basic prescriptions, e.g., HXZQ (Huoxiangzhengqi capsule). (**D**) Top 3 remedies that impacted more than 1000 genes are shown. The legend is similar to that of C, except that the yellow diamonds were used for symptom onset, e.g., JHQG (Jinhuaqinggan Granule).

To achieve this, we first reviewed the molecular targets of 14 SSMs merged from NCBI and GeneCards. The typical SSMs associated with COVID-19 were fever (88.7%) 40, coughing (67.8%) 40 41, fatigue (69.6%) 42, sputum production (28%) 43 and diarrhea (3.8%) 40. In addition, other SSMs were also included, such as SARS, pneumonia, nausea, asthenia, cytokine storm, oxygen saturation, lymphopenia and dyspnea 44. Those targets were constrained with the entire database, that is, all TCMSP targets (Fig. 5A). Then, we overlapped these targets with the targets of selected recipes, namely, 13 effective remedies and 5 specific original recipes. The significance level of coincidence (SLC) with this intersection was calculated, as shown in a heatmap using -Log10 P with the depth of color (Fig. 5B). Within the SSMs, pneumonia and nausea (due to their rareness) served as positive and negative controls, respectively. The COVID-19 targets were the same as in Fig. 3A. Owing to the decrease in background noise due to target base number diversity, we illustrated the separation (Fig. 5B, C, D).

In general, most clinically administered recipes as well as basic prescriptions, except for SMI (Shengmai Injection), had a high SLC among the major SSMs, such as pyrexia, myalgia, asthenia, lymphopenia, pneumonia and COVID-19. SMI had a suitable SLC in asthenia, myalgia and lymphopenia, which fit its clinical indication. Moreover, SMI was clinically applied across the prevention, critical and recovery stages but not in the confirmed and developing stages (Fig. 2B), which were in accordance with the low SLC in acute attack, such as pyrexia and cytokine storm (Fig. 5B).

Among the 18 referred remedies, JHQG (Jinhuaqinggan Granule), LHQW (Lianhuaqingwen Capsule) and XBJI (Xuebijing Injection); QFPD (Qingfeipaidu decoction), HSBD (Huashibaidu decoction) and Xuanfeibaidu decoction were the most typical and effectual remedies and contain 3 medicines (3Ms) and 3 recipes (3Rs) highly recommended by the NHC. The 3Ms (JHQG, LHQW and XBJI) were previously approved for influenza; furthermore, JHQG was approved for H1N1, LHQW for SARS, and XBJI for SARS critical care, such as acute lung injury, ARDS, pyemia, multiple organ failure, etc. In addition, JHQG and LHQW originated from MXSG (Maxingshigan decoction) and YQS (Yinqiao powder). Moreover, most of recipes have been used in registered national clinical research on COVID-19, including QFPD, HSBD, LHQW, RDNI (Reduning Injection), SFT (Shenfu Injection) and MXSG (Table S15). For instance, based on clinical research on COVID-19, the duration of fever was shortened by approximately 1.5 days, and dyspnea and expectoration were both diminished remarkably (77.8% vs 0; 64.3% vs 9.1%) by treatment with LHQW compared with the control 45. In addition, SARS-CoV-2 replication was significantly inhibited, and proinflammatory cytokines (IL6, CCL2, CXCL10, TNF-α) were also decreased notably after treatment with LHQW in Vero E6 cells 46. HXZQS (Huoxiangzhengqi capsule), LHQW and JHQG, the 3 common Chinese patent prescriptions for the early stages of COVID-19, were included in the *Diagnosis and Treatment Protocol for Novel Coronavirus Pneumonia (trial version 7)* released by the NHC. LSY, a representative ethical prescription formulated by a renowned TCM master named Liu Shangyi, is widely used in southern China. Some typical formulas corresponding to the symptoms and pathology of COVID-19 were further analyzed, including DYY (Dayuan Decoction, an anti-epidemic remedy since the Qing dynasty, 200 years ago), ECT (Erchen Decoction, the original recipe to eliminate phlegm), SJZT (Sijunzi Decoction, the energy-boosting foundation) and YPFS (Yupingfeng Powder), the most commonly used in prevention (Fig. 2B).

The pros and cons of the recipes are also presented in Fig. 5B from the molecular target perspective. For instance, SFT and SMY were formulated to recuperate and energize. They indeed demonstrated a higher SLC in asthenia and lower SLC in pyrexia, relative to other SSMs. Furthermore, to obtain a better overview of the coherence between SSMs and clinical remedies, the top remedies with high coherence to each SSM are presented in Fig. 5C, D. RDNI 47, a widespread clinical prescription used in upper respiratory infection and recommended by the NHC during the critical course, was among the best (top 5 showed effects on all 14 SSMs), followed by LSY, HXZQS, ECT 48, SJZT and XBJI 12. Recipes with more than 1000 targets were separately ranked and are shown in Fig. 5D to reduce the background noise caused by the base target number. YQS, JHQG and QFPD were the best 3 among all large formulas. Other remedies, such as DYY, YPFS, XBCQT, etc. were among the recipes with lower SLCs in SSMs.

Significance level coherence (SLC) analysis is, however, compromised by the variations of base number; thus, a comparison of SLCs with similar base number is preferred. For instance, as YQS was a basic prescription, the core herbs and their targets might have covered the majority of the listed SSMs; its derivatives usually have additional herbs, aiming to target other unlisted SSMs, the statistical SLC might, therefore, be reduced due to this “dilution effect”, especially for QFPD, the largest formula formed by a combination of 4 original recipes.

Therefore, the SLC was merely an indicator of potential therapeutic functions by adding biological evidence and explanations. Clinical trials are the only way to substantiate the efficacy of these remedies. In fact, 76 (12.56%) clinical trials with TCM remedies (Table S15 with details, e.g., sample size, study design, phase, etc.) have been implemented among 605 registered clinical studies from the NHC as of May 12 (Table S16), which have yet to be reported, including SFT, RDNI, XBJI, LHQW, MXSG, HSBD, QFPD, etc. whereas some were reported with appealing results, e.g., HSBD 41, LHQW 45, JHQG 39 and QFPD 12.

## Conclusions

In the battle against the COVD-19 pandemic, TCM played a critical role in China. We systematically analyzed clinically applied TCM recipes. TCM coordinated COVID-19 from multiple dimensions, not merely targeting viruses but also targeting the whole body. In particular, by multidirectional targeting converging to balance molecular pathways, herbs within a remedy implicated concerted mechanisms, largely attributable to most medical conditions as well as unincorporated symptoms by modulating certain herbs. A significant level of coherence (SLC) indicated the flexible and integrated functionality of TCM remedies in coordinating the pandemic. Moreover, the docking results certainly revealed the direct anti-SARS-CoV-2 potential of ingredients. Madreselvin B, 5MPB, PG and kaempferol 3-O-β-D-(6''-p- coumaroyl)glucopyranosyl(1-2)-α-L-rhamnopyranoside were among the best ingredients, while Flos Lonicerae (Jin Yin Hua), Herba Penthori (Gan Huang Cao), Chinese Gall (Wu Bei Zi) and Ginkgo Biloba (Yin Xing) were the herbs with best SARS-CoV-2-binding potential. Therefore, TCM has potential to combat COVID-19 via integrative mechanisms, which needs further exploration.

## Discussion

The outbreak of the COVID-19 pandemic has brought together Western and Eastern medicine. Statistical data show that, as of March 23, 91.5% of all confirmed cases, nearly 75000 patients, adopted integrated treatment with Eastern and Western medicine, reaching a curative rate of more than 90% based on clinical observations 49. In China, TCM has been particularly promoted by the government and NHC within the epidemic campaign. To date, 7 versions have been published to conduct the diagnosis and treatment for COVID-19. A total of 136 TCM clinical trials were registered among 605 clinical studies from the NHC (all details are presented in Table S16), as of May 12, including 76 trials clearly related to remedies or TCM patents (Table S15), 32 including other TCM treatments (e.g., shadowboxing, Liu-Zi-Jue Qigong, moxibustion, acupuncture, triple energy treatment, etc.) and 29 trials combined with Western medicine.

Some competitive superiority of TCM application includes multiple immunoregulatory, energy-boosting, blood circulation-promoting, appetite-enhancing activities, etc., especially for the prevention, recovery and partly critical stages. For instance, a 70% increase in lymphocytes among 102 cases treated with TCM 50 and 30% stabilization of other illnesses using QFPD under the major symptoms (e.g., fever, cough, nausea, etc.) were relieved among 214 confirmed patients across 4 provinces 51. Potent immunosuppressive effects that could inhibit cytokine storms and some common fatal injuries were demonstrated with compelling evidence 52, 53. Specifically, inflammatory cytokines, especially proinflammatory cytokines, such as Th1, Th2, Th17, IL6, IL-1β, TNF-α, IL-8, etc., were clearly suppressed *in vitro* and *in vivo* by Shen Fu Injection (SFT) 54, Reduning Injection (RDNI) 55, 56 and some compounds (e.g., tetrandrine 57) extracted from Chinese herbs. Therefore, it is likely that the implementation of TCM could control the threat of COVID-19 via different dimensions.

Nevertheless, the major obstacle for encouraging TCM usage in the COVID-19 crisis is the multitudinous formulations without clear molecular mechanistic descriptions, which granted an in-depth analysis of hierarchy relationships.

In this work, we evaluated 185 clinically used TCM recipes and provided insight into their taxonomic distance and origin based on TCM formulating principles. The top ten hubs of core recipes are presented (Fig. 2A, B). They are primarily ancient anti-epidemic formulations, including MXSG 58, YQS 59, YPFS 60, and MXYG 61. Many derivatives evolved from these ancient remedies based on empirical experiences and corresponding SSMs.

We further assessed the molecular relevance of TCM effectiveness via herbal medicine and medical biology analyses. We have combined network pharmacology, target count frequency, and regulation directionality to provide in-depth information on the molecular biological functions of herbs. The anti-inflammatory and antipyretic properties of TCM remedies were in line with the formulation clou called dispelling pathogenic heat or evil-heat. Another TCM formulation clou is to target exopathogens or extra-evil, equivalent to pathogenic microbes. The central question is whether TCM remedies have antivirus effects, specifically anti-coronavirus effects.

To address this question, we revealed that a group of ingredients have even higher potential to interfere with SARS-CoV-2 proteins than reported antiviral therapeutics (lopinavir, ritonavir, ribavirin, arbidol, remivir and chloroquine phosphate) by docking efficacy analysis to CoV spike (S) glycoprotein (6SVB) and CoV 3CL hydrolase (6LU7). Five out of the 8 top herbs, namely, JYH, LQ, GC, MH and HQi, contained ingredients with better targeting potential to either or both 6SVB and 6LU7 than the antiviral therapeutic controls. Seventeen potential ingredients were identified to target either COVID-19 protein or both proteins, including Madreselvin B/A, rutin, licorice glycoside E, suspensaside B, plantainoside A, euchrenone, etc. (Table S7, S12). We further expanded the search for all natural compounds beyond the listed ones, and 8 additional herbs were found (Fig. 4J, Table S9). Some of the results were recently substantiated by other groups 62, 63. The top three unlisted herbs with anti-coronavirus potential were Herba Penthori (Gan Huang Cao), Chinese Gall (Wu Bei Zi) and Ginkgo Biloba (Yin Xing). Ginkgo Biloba is well documented as a multifunctional herb involving inflammatory factor inhibition (NO, TNF-α, IL6, and PGE2) 64 and circulation-boosting and anti-platelet aggregation effects.

Interestingly, molecular docking results were in line with clinical outcomes. For instance, JYH which includes the top anti-SARS-CoV-2 compounds (madreselvin A/B 65 and rutin), was among the most commonly used herb during the outbreak (Fig. 3A). Enriched in flavone, triterpenoid saponin compounds and essential oil, Flos Lonicerae has multifactorial effects as well, including anti-pathogenic 66, anti-inflammatory, and metabolism regulation effects.

Furthermore, the major determinator for hypoxemia mortality in the COVID-19 crisis was sputum retention or dysfunction in expectoration. TCM has many antitussive and expectorant measured. ECT, the classical recipe specifically for phlegm, includes 5 herbs: Rhizoma Pinelliae (Ban Xia), Pericarpium Citri Reticulatae (Chen Pi), Poria (Fu Ling) and GC. The molecular analysis of the recipes also indicated the functionality of the mechanism for expectoration, with the highest SLC for lymphopenia, mucus and nausea separately within the first-degree cluster (recipe target number <1,000), which might be modulated by the integrated immune status. Other commonly applied herbs for sputum were enriched in those clinically effective recipes, even among the top 10 herbs used, including XR, HX, HQi and GC.

We then systematically assessed SSMs of major clinically applied recipes. SLC was used to illustrate the functionality of recipes. Using this statistical format, we could approximately assess the capabilities of the recipes to combat COVID-19, including the SSMs of pyrexia, asthenia, coughing, myalgia, nausea, diarrhea, pneumonia, cytokine storm, lymphopenia, oxygen saturation, etc. Most of the clinically applied remedies were found to be effective for these SSMs, except for a few that were formulated for special applications, which generally corresponded with clinical reports.

This approach of connecting molecular targets with functions of a recipe was appealing, nevertheless, the analysis was limited by a few factors, such as the limitation of SSMs and herbs on the molecular target basis, the numbers of targets within a database, the diversity of gene names, the loss of data during the gene symbol transfection, the large difference in target number within recipes and SSMs, etc. Therefore, more exploration is needed.

Finally, the corroboration of the TCM functions and principles might be a paradigm shift.

## Supplementary Material

Supplementary figures and tables.

## Figures and Tables

**Table 1:** Top 10 basic recipes with the course distribution of their related derivatives

Basic Recipes	Total Frequency	Early-Prevention	Mild-Moderate-Developing	Severe-Critical	Recovery
MXSG	30	3	22	5	/
YQS	29	10	18	1	/
YPFS	17	15	2	/	/
MXYG	12	/	11	1	/
SFT	11	/	/	11	/
XBCQT	11	/	7	4	/
SMY	9	1	/	4	4
HPXLT	9	/	8	1	/
HXZQS	8	3	5	/	/
DYY	6	1	5	/	/

The total frequency of each most commonly used original recipe shows the amounts of corresponding derivatives (all 49 basic recipes are shown in Table 2), and the separate frequency with each course was divided from unified indications. '/' means not applied for this course with the recipe. Each disease stage is represented by a color: green represents early preventive, orange represents mild-moderate-developing, red represents severe-critical, and purple indicates the recovery stage (the same as in Fig. 2B). In decreasing order, the top 10 basic recipes with abbreviations based on the total frequency among 185 remedies are as follows: MXSG: Maxingshigan Decoction, YQS: Yinqiao Powder, YPFS: Yupingfeng Powder, MXYG: Maxingyigan decoction, SFT: Shenfu Decoction, XBCQT: Xuanbaichengqi Decoction, SMY: Shengmai Decoction, HPXLT: Huopuxialing Decoction, HXZQS: Huoxiangzhengqi Powder, DYY: Dayuan Decoction.

**Table 2:** Overview of the 49 basic recipes extracted from 185 clinical remedies based on the “*monarch stratum principle”*

NO.	Basic Recipes	*Jun (Monarch)*	*Chen (Minister)*	*Zuo (assistant)*	*Shi (courier)*	Herb Amounts	Frequency
1	Maxingshigan Decoction	Herba Ephedrae (麻黄, Ma Huang), Gypsum Fibrosum (石膏, Shi Gao)	Semen Armeniacae Amarum (杏仁, Xing Ren)	Radix Glycyrrhizae (甘草, Gan Cao)		4	30
2	Yinqiao Powder	Fructus Forsythiae (连翘, Lian Qiao), Flos Lonicerae (金银花, Jin Yin Hua)	Herba Menthae Heplocalycis (薄荷, Bo He), Fructus Arctii (牛蒡子, Niu Bang Zi), Herba Schizonepetae (荆芥, Jing Jie), Semen Sojae Preparatum (淡豆豉, Dan Dou Chi)	Radix Platycodonis (桔梗, Jie Geng), Rhizoma Phragmitis (芦根, Lu Gen), Lophatherum gracile (淡竹叶, Dan Zhu Ye)	Radix Glycyrrhizae (甘草, Gan Cao)	10	29
3	Yupingfeng Powder	Radix Astragali seu Hedysari (黄芪, Huang Qi)	Rhizoma Atractylodis Macrocephalae (白术, Bai Zhu)	Radix Saposhnikoviae (防风, Fang Feng)		3	18
4	Maxingyigan Decoction	Herba Ephedrae (麻黄, Ma Huang)	Semen Armeniacae Amarum (杏仁, Xing Ren), Semen Coicis (薏苡仁, Yi Yi Ren)			4	13
5	Shenfu Decoction	Radix Ginseng (人参, Ren Shen), Radix Aconiti Lateralis Preparata (附子, Fu Zi)				2	11
6	Xuanbaichengqi Decoction	Gypsum Fibrosum (石膏, Shi Gao), Radix et Rhizoma Rhei (大黄, Da Huang)	Semen Armeniacae Amarum (杏仁, Xing Ren), Fructus Trichosanthis (瓜蒌, Gua Lou)			4	11
7	Shengmai Powder	Radix Ginseng (人参, Ren Shen)	Radix Ophiopogonis (麦冬, Mai Dong)	Fructus Schisandrae Chinensis (五味子, Wu Wei Zi)		3	10
8	Dayuan Decoction	Fructus Tsaoko (草果, Cao Guo)	Semen Arecae (槟榔, Bin Lang), Cortex Magnoliae Officinalis (厚朴, Hou Po)	Rhizoma Anemarrhenae (知母, Zhi Mu), Radix Scutellariae (黄芩, Huang Qin), Radix Paeoniae Alba (白芍, Bai Shao), Radix Glycyrrhizae (甘草, Gan Cao)		7	9
9	Huopuxialing Decoction	Herba Agastaches (藿香, Huo Xiang), Cortex Magnoliae Officinalis (厚朴, Hou Po), Rhizoma Pinelliae (半夏, Ban Xia), Poria (茯苓, Fu Ling)	Semen Armeniacae Amarum (杏仁, Xing Ren), Semen Coicis (薏苡仁, Yi Yi Ren), Fructus Amomi Rotundus (豆蔻, Dou Kou)	Polyporus Umbellatus (猪苓, Zhu Ling), Semen Sojae Preparatum (淡豆豉, Dan Dou Chi), Rhizoma Alismatis (泽泻, Ze Xie)		10	9
10	Huoxiangzhengqi powder	Herba Agastaches (藿香, Huo Xiang)	Rhizoma Pinelliae (半夏, Ban Xia), Pericarpium Citri Reticulatae (陈皮, Chen Pi), Rhizoma Atractylodis Macrocephalae (白术, Bai Zhu), Poria (茯苓, Fu Ling)	Folium Perillae (紫苏叶, Zi Su Ye), Radix Angelicae Dahuricae (白芷, Bai Zhi), Pericarpium Arecae (大腹皮, Da Fu Pi), Cortex Magnoliae Officinalis (厚朴, Hou Po), Radix Platycodonis (桔梗, Jie Geng), Rhizoma Zingiberis Recens (生姜, Sheng Jiang), Fructus Jujubae (大枣, Da Zao)	Radix Glycyrrhizae (甘草, Gan Cao)	13	8
11	Angongniuhuang Pills	Calculus Bovis (牛黄, Niu Huang), Rhinoceros unicornis L. (犀角, Xi Jiao), Moschus (麝香, She Xiang)	Rhizoma Coptidis (黄连, Huang Lian), Radix Scutellariae (黄芩, Huang Qin), Gardenia jasminoides Ellis (栀子, Zhi Zi)	Borneolum Syntheticum (冰片, Bing Pian), Radix Curcumae (郁金, Yu Jin), Realgar (雄黄, Xiong Huang), Cinnabaris (朱砂, Zhu Sha), Margarita (珍珠, Zhen Zhu), (金箔, Jin Bo)	Honey (蜜, Mi)	13	8
12	Zixue Dan	Rhinoceros unicornis L. (犀角, Xi Jiao), Cornu Saigae Tataricae (羚羊角, Ling Yang Jiao), Moschus (麝香, She Xiang)	Gypsum Fibrosum (石膏, Shi Gao), Calcitum (寒水石, Han Shui Shi), Talcum (滑石, Hua Shi)	Radix Scrophulariae (玄参, Xuan Shen), Rhizoma Cimicifugae (升麻, Sheng Ma), Radix Aucklandiae9 (木香, Mu Xiang), Flos Caryophylli (丁香, Ding Xiang), Lignum Aquilariae Resinatum (沉香, Chen Xiang), P. sibiricum. Delar. ex Redout6 (黄精,Huang Jing), Cinnabaris (朱砂, Zhu Sha), Magnetitum (磁石, Ci Shi), mirabilite (朴硝, Po Xiao), saltpetre (硝石, Xiao Shi)	Radix Glycyrrhizae (甘草, Gan Cao)	13	6
13	Shengjiang Powder	Bombyx Batryticatus (僵蚕, Jiang Can), Periostracum Cicadae (蝉蜕, Chan Tui)	Radix et Rhizoma Rhei (大黄, Da Huang), Rhizoma Curcumae Longae (姜黄, Jiang Huang)			4	6
14	Sanren Decoction	Talcum (滑石, Hua Shi)	Semen Coicis (薏苡仁, Yi Yi Ren), Semen Armeniacae Amarum (杏仁, Xing Ren), Fructus Amomi Rotundus (豆蔻, Jave Amonum Fruit)	Medulla Tetrapanacis (通草, Tong Cao), Lophatherum gracile (淡竹叶, Dan Zhu Ye), Rhizoma Pinelliae (半夏, Ban Xia), Cortex Magnoliae Officinalis (厚朴, Hou Po)		8	5
15	Xiaochaihu Decoction	Radix Bupleuri (柴胡, Chai Hu)	Radix Scutellariae (黄芩, Huang Qin)	Rhizoma Pinelliae (半夏, Ban Xia), Rhizoma Zingiberis Recens (生姜, Sheng Jiang), Radix Ginseng (人参, Ren Shen), Fructus Jujubae (大枣, Da Zao)	Radix Glycyrrhizae (甘草, Gan Cao)	7	4
16	Erchen Decoction	Rhizoma Pinelliae (半夏, Ban Xia)	Pericarpium Citri Reticulatae (陈皮, Chen Pi)	Poria (茯苓, Fu Ling), Prunus mume (乌梅, Wu Mei)	Radix Glycyrrhizae (甘草, Gan Cao), Rhizoma Zingiberis Recens (生姜, Sheng Jiang)	6	4
17	Sangbei powder	Cortex Mori (桑白皮, Sang Bai Pi)	Bulbus Fritillariae Thunbergii (浙贝母, Zhe Bei Mu)			2	3
18	Zhuye Shigao Decoction	Gypsum Fibrosum (石膏, Shi Gao)	Radix Ginseng (人参, Ren Shen), Radix Ophiopogonis (麦冬, Mai Dong)	Lophatherum gracile (淡竹叶, Dan Zhu Ye), japonica Rice (粳米, Jing Mi), Radix Glycyrrhizae (甘草, Gan Cao)		7	3
19	Shashen Maidong Decoction	Adenophora stricta Miq. (沙参, Sha Shen)	Radix Ophiopogonis (麦冬, Mai Dong)	Folium Mori (桑叶, Sang Ye), Rhizoma Polygonati Odorati (玉竹,Yu Zhu), Semen Dolichoris Album (白扁豆, Bai Bian Dou), Radix Trichosanthis (天花粉, Tian Hua Fen)	Radix Glycyrrhizae (甘草, Gan Cao)	7	3
20	Chaihu Dayuan Decoction	Radix Bupleuri (柴胡, Chai Hu), Radix Scutellariae (黄芩, Huang Qin)	Fructus Aurantii (枳壳, Zhi Qiao), Radix Platycodonis (桔梗, Jie Geng), Cortex Magnoliae Officinalis (厚朴, Hou Po), Fructus Tsaoko (草果, Cao Guo), Pericarpium Citri Reticulatae Viride (青皮, Qing Pi), Semen Arecae (槟榔, Bin Lang)	lotus petiole (荷梗, He Geng)	Radix Glycyrrhizae (甘草, Gan Cao)	10	3
21	Shenling Baizhu Powder	Radix Ginseng (人参, Ren Shen), Rhizoma Atractylodis Macrocephalae (白术, Bai Zhu), Poria (茯苓, Fu Ling)	Rhizoma Dioscoreae (山药, Shan Yao), Semen Nelumbinis (莲子, Lian Zi), Semen Dolichoris Album (白扁豆, Bai Bian Dou), Semen Coicis (薏苡仁, Yi Yi Ren)	Fructus Amomi Villosi (砂仁, Sha Ren), Radix Platycodonis (桔梗, Jie Geng)	Radix Glycyrrhizae (甘草, Gan Cao), Fructus Jujubae (大枣, Da Zao)	11	3
22	Qingwenbaidu Decoction	Gypsum Fibrosum (石膏, Shi Gao), Rhizoma Anemarrhenae (知母, Zhi Mu), Radix Glycyrrhizae (甘草, Gan Cao), Rhizoma Coptidis (黄连, Huang Lian), Radix Scutellariae (黄芩, Huang Qin), Gardenia jasminoides Ellis (栀子, Zhi Zi), Rhinoceros unicornis L. (犀角, Xi Jiao), Radix Rehmanniae Recens (生地, Sheng Di), Radix Paeoniae Rubra (赤芍, Chi Shao), Moutan DouCortex (丹皮, Dan Pi)	Fructus Forsythiae (连翘, Lian Qiao), Lophatherum gracile (淡竹叶, Dan Zhu Ye)	Radix Scrophulariae (玄参, Xuan Shen), Radix Platycodonis (桔梗, Jie Geng)		14	2
23	Ganlu Xiaodu micropills	Talcum (滑石, Hua Shi), Herba Artemisiae Scopariae (茵陈, Yin Chen), Radix Scutellariae (黄芩, Huang Qin)	Fructus Amomi Rotundus (豆蔻, Dou Kou), Rhizoma Acori Tatarinowii (石菖蒲, Shi Chang Pu), Herba Agastaches (藿香, Huo Xiang)	Fructus Forsythiae (连翘, Lian Qiao), Herba Menthae Heplocalycis (薄荷, Bo He), Rhizoma Belamcandae (射干, She Gan), Bulbus Fritillariae Thunbergii (浙贝母, Zhe Bei Mu), Akebia quinata (Houtt.) Decne. (木通, Mu Tong)		11	2
24	Sangju Decoction	Folium Mori (桑叶, Sang Ye), Flos Chrysanthemi (菊花, Ju Hua)	Herba Menthae Heplocalycis (薄荷, Bo He), Semen Armeniacae Amarum (杏仁, Xing Ren), Radix Platycodonis (桔梗, Jie Geng)	Fructus Forsythiae (连翘, Lian Qiao), Rhizoma Phragmitis (芦根, Lu Gen)	Radix Glycyrrhizae (甘草, Gan Cao)	8	2
25	Xiebai Powder	Cortex Mori (桑白皮, Sang Bai Pi)	Cortex Lycii (地骨皮, Di Gu Pi)	Radix Glycyrrhizae (甘草, Gan Cao), japonica Rice (粳米, Jing Mi)		4	2
26	Buzhongyiqi Decoction	Radix Astragali seu Hedysari (黄芪, Huang Qi)	Radix Ginseng (人参, Ren Shen), Radix Glycyrrhizae (甘草, Gan Cao)	Rhizoma Atractylodis Macrocephalae (白术, Bai Zhu), Radix Angelicae Sinensis (当归, Dang Gui), Rhizoma Cimicifugae (升麻, Sheng Ma), Radix Bupleuri (柴胡, Chai Hu)		8	2
27	Huangqi Guizhi Wuwu Decoction	Radix Astragali seu Hedysari (黄芪, Huang Qi)	Ramulus Cinnamomi (桂枝, Gui Zhi), Radix Paeoniae Alba (白芍, Bai Shao)	Rhizoma Zingiberis Recens (生姜, Sheng Jiang), Fructus Jujubae (大枣, Da Zao)		5	2
28	Sanao Decoction	Herba Ephedrae (麻黄, Ma Huang)	Semen Armeniacae Amarum (杏仁, Xing Ren), Radix Glycyrrhizae (甘草, Gan Cao)			3	2
29	Suhexiang Pills	Styrax (苏合香, Su He Xiang), Benzoinum (安息香, An Xi Xiang), Moschus (麝香, She Xiang), Dryobalanops aromatica Gaertn. f. (龙脑, Long Nao)	Flos Caryophylli (丁香, Ding Xiang), Fructus Piperis Longi (荜茇, Bi Ba), Rhizoma Cyperi (香附, Xiang Fu), Lignum Aquilariae Resinatum (沉香, Chen Xiang), Radix Aucklandiae9 (木香, Mu Xiang), Lignum Santali Albi (檀香, Tan Xiang)	Rhizoma Atractylodis Macrocephalae (白术, Bai Zhu), Olibanum (乳香, Ru Xiang), Fructus Chebulae (诃子肉, He Zi Rou), Cornu Bubali (水牛角, Shui Niu Jiao), Cinnabaris (朱砂, Zhu Sha)		15	2
30	Mahuang Decoction	Herba Ephedrae (麻黄, Ma Huang)	Ramulus Cinnamomi (桂枝, Gui Zhi)	Semen Armeniacae Amarum (杏仁, Xing Ren)	Radix Glycyrrhizae (甘草, Gan Cao)	4	2
31	Baihu Decoction	Gypsum Fibrosum (石膏, Shi Gao)	Rhizoma Anemarrhenae (知母, Zhi Mu)	japonica Rice (粳米, Jing Mi), Radix Glycyrrhizae (甘草, Gan Cao)		4	1
32	Xiaoxianxiong Decoction	Fructus Trichosanthis (瓜蒌, Gua Lou)	Rhizoma Coptidis (黄连, Huang Lian)	Rhizoma Pinelliae (半夏, Ban Xia)		3	1
33	Jingfangbaidu powder	Herba Schizonepetae (荆芥, Jing Jie), Radix Saposhnikoviae (防风, Fang Feng)	Rhizoma et Radix Notopterygii (羌活, Qiang Huo), Radix angelicae seu Hemsley (独活, Du Huo), Radix Bupleuri (柴胡, Chai Hu), Poria (茯苓, Fu Ling)	Radix Peucedani (前胡, Qian Hu), Fructus Aurantii (枳壳, Zhi Qiao), Radix Platycodonis (桔梗, Jie Geng), Rhizoma Ligustici Chuanxiong (川芎, Chuan Xiong), Radix Ginseng (人参, Ren Shen)	Radix Glycyrrhizae (甘草, Gan Cao)	12	1
34	Caoguo Zhimu Decoction	Fructus Tsaoko (草果, Cao Guo), Rhizoma Anemarrhenae (知母, Zhi Mu)	Rhizoma Pinelliae (半夏, Ban Xia), Cortex Magnoliae Officinalis (厚朴, Hou Po)	Radix Scutellariae (黄芩, Huang Qin), Prunus mume (乌梅, Wu Mei)		6	1
35	Xijiao Dihuang Decoction	Rhinoceros unicornis L. (犀角, Xi Jiao)	Radix Rehmanniae Recens (生地, Shen Di)	Radix Paeoniae Alba (白芍, Bai Shao), Moutan Cortex (丹皮, Dan Pi)		4	1
36	Zhisou Powder	Radix Asteris (紫菀, Zi Wan), Radix Stemonae (百部, Bai Bu)	Radix Platycodonis (桔梗, Jie Geng), Rhizoma Cynanchi Stauntonii (白前, Bai Qian)	Herba Schizonepetae (荆芥, Jing Jie), Pericarpium Citri Reticulatae (陈皮, Chen Pi)	Radix Glycyrrhizae (甘草, Gan Cao)	7	1
37	Wuling Powder	Rhizoma Alismatis (泽泻, Ze Xie)	Poria (茯苓, Fu Ling), Polyporus Umbellatus (猪苓, Zhu Ling)	Rhizoma Atractylodis Macrocephalae (白术, Bai Zhu), Ramulus Cinnamomi (桂枝, Gui Zhi)		5	1
38	Shegan Mahuang Decoction	Rhizoma Belamcandae (射干, She Gan)	Herba Ephedrae (麻黄, Ma Huang)	Asarum sieboldii Miq. (细辛, Xi Xin), Radix Asteris (紫菀, Zi Wan), Flos Farfarae (冬花, Dong Hua), Rhizoma Pinelliae (半夏, Ban Xia), Radix Asteris (紫菀, Zi Wan), Fructus Schisandrae Chinensis (五味子, Wu Wei Zi)	Rhizoma Zingiberis Recens (生姜, Sheng Jiang), Fructus Jujubae (大枣, Da Zao)	10	1
39	Qingqihuatan Decoction	Rhizoma Arisaematis Cum Bile (胆南星, Dan Nan Xing)	Fructus Trichosanthis (瓜蒌, Gua Lou), Radix Scutellariae (黄芩, Huang Qin), Rhizoma Pinelliae (半夏, Ban Xia)	Semen Armeniacae Amarum (杏仁, Xing Ren), Pericarpium Citri Reticulatae (陈皮, Chen Pi), Fructus Aurantii Immaturus (枳实, Zhi Shi), Poria (茯苓, Fu Ling)		8	1
40	Qingying Decoction	Rhinoceros unicornis L. (犀角, Xi Jiao)	Radix Rehmanniae Recens (生地, Shen Di), Radix Ophiopogonis (麦冬, Mai Dong), Radix Scrophulariae (玄参, Xuan Shen)	Flos Lonicerae (金银花, Jin Yin Hua), Fructus Forsythiae (连翘, Lian Qiao), Lophatherum gracile (淡竹叶, Dan Zhu Ye), Rhizoma Coptidis (黄连, Huang Lian), Radix Salviae Miltiorrhizae (丹参, Dan Shen)		9	1
41	Wangshiqingshuyiqi Decoction	Exocarium Citrulli (西瓜翠衣, Xi Gua Cui Yi), Radix Panacis Quinquefolii (西洋参, Xi Yang Shen)	lotus petiole (荷梗, He Geng), Herba Dendrobii (石斛, Shi Hu), Radix Ophiopogonis (麦冬, Mai Dong)	Rhizoma Coptidis (黄连, Huang Lian), Rhizoma Anemarrhenae (知母, Zhi Mu), Lophatherum gracile (淡竹叶, Dan Zhu Ye), japonica Rice (粳米, Jing Mi), Radix Glycyrrhizae (甘草, Gan Cao)		10	1
42	Sanshi Decoction	Talcum (滑石, Hua Shi)	Gypsum Fibrosum (石膏, Shi Gao), Calcitum (寒水石, Han Shui Shi), Flos Lonicerae (金银花, Jin Yin Hua)	Semen Armeniacae Amarum (杏仁, Xing Ren), Caulis Bambusae in Taenia (竹茹, Zhu Ru)	Medulla Tetrapanacis (通草, Tong Cao), Poop (金汁, Jin Zhi)	8	1
43	Huangqi Liujunzi Decoction	Radix Astragali seu Hedysari (黄芪, Huang Qi), Radix Ginseng (人参, Ren Shen)	Rhizoma Atractylodis Macrocephalae (白术, Bai Zhu), Poria (茯苓, Fu Ling), Rhizoma Dioscoreae (山药, Shan Yao)	Pericarpium Citri Reticulatae (陈皮, Chen Pi), Rhizoma Pinelliae (半夏, Ban Xia)	Radix Glycyrrhizae (甘草, Gan Cao)	8	1
44	Shenfulongmu Decoction	Radix Ginseng (人参, Ren Shen), Radix Aconiti Lateralis Preparata (附子, Fu Zi)	Bone fossil of big mammals (煅龙骨, Duan Long Gu), Oyster (煅牡蛎, Duan Mu Li)	Rhizoma Zingiberis Recens (生姜, Sheng Jiang), Fructus Jujubae (大枣, Da Zao)		6	1
45	Fangfeng Tongsheng Powder	Herba Ephedrae (麻黄, Ma Huang), Radix Saposhnikoviae (防风, Fang Feng), Herba Schizonepetae (荆芥, Jing Jie), Herba Menthae Heplocalycis (薄荷, Bo He), Radix Scutellariae (黄芩, Huang Qin), Gypsum Fibrosum (石膏, Shi Gao), Fructus Forsythiae (连翘, Lian Qiao), Radix Platycodonis (桔梗, Jie Geng), Gardenia jasminoides Ellis (栀子, Zhi Zi), Talcum (滑石, Hua Shi), Natrii Sulfas (芒硝, Mang Xiao), Radix et Rhizoma Rhei (大黄, Da Huang)	Radix Angelicae Sinensis (当归, Dang Gui), Radix Paeoniae Alba (白芍, Bai Shao), Rhizoma Ligustici Chuanxiong (川芎, Chuan Xiong)	Rhizoma Atractylodis Macrocephalae (白术, Bai Zhu), Radix Glycyrrhizae (甘草, Gan Cao)	Rhizoma Zingiberis Recens (生姜, Sheng Jiang)	18	1
46	Sijunzi Decoction	Radix Ginseng (人参, Ren Shen)	Rhizoma Atractylodis Macrocephalae (白术, Bai Zhu)	Poria (茯苓, Fu Ling)	Radix Glycyrrhizae (甘草, Gan Cao)	4	1
47	Shexiang Niuhuang Pills	Calculus Bovis (牛黄, Niu Huang), Moschus (麝香, She Xiang), Realgar (雄黄, Xiong Huang)	Rhizoma Coptidis (黄连, Huang Lian), Radix Scutellariae (黄芩, Huang Qin), Fructus Forsythiae (连翘, Lian Qiao), Cortex Phellodendri (黄柏, Huang Bo), Gardenia jasminoides Ellis (栀子, Zhi Zi), Flos Lonicerae (金银花, Jin Yin Hua), Radix Angelicae Sinensis (当归, Dang Gui), Gypsum Fibrosum (石膏, Shi Gao)	Radix Paeoniae Rubra (赤芍, Chi Shao), Radix Saposhnikoviae (防风, Fang Feng), Radix Ophiopogonis (麦冬, Mai Dong), Radix Platycodonis (桔梗, Jie Geng), Cinnabaris (朱砂, Zhu Sha), Borneolum Syntheticum (冰片, Bing Pian), Radix et Rhizoma Rhei (大黄, Da Huang), Ramulus Uncariae Cum Uncis (钩藤, Gou Teng)	Herba Menthae Heplocalycis (薄荷, Bo He), Radix Glycyrrhizae (甘草, Gan Cao)	21	1
48	Gegen Decoction	Radix Puerariae (葛根, Ge Gen), Herba Ephedrae (麻黄, Ma Huang)	Ramulus Cinnamomi (桂枝, Gui Zhi), Radix Paeoniae Alba (白芍, Bai Shao)	Rhizoma Zingiberis Recens (生姜, Sheng Jiang), Radix Glycyrrhizae (甘草, Gan Cao), Fructus Jujubae (大枣, Da Zao)		7	1
49	Situ Decoction	Rhizoma Smilacis Glabrae (土茯苓, Tu Fu Ling), Radix et Rhizome Achyranthes (土牛膝, Tu Niu Xi)	Rhizoma Bolbostemmatis (土贝母, Tu Bei Mu), Rumex madaio MakinoR. daiwoo Makino (土大黄, Tu Da Huang)			4	1

In total, 49 basic recipes were extracted from the database with 185 clinically applied TCM remedies combating COVID-19. “*monarch stratum principle” (“Jun-Chen-Zuo-Shi”)* is one of the classical principles used to compose herbs, arranging the dosage and weights, which were similarly administered within the country. The table presents the complete details of the 49 remedies, including herb details at the four sites, herb amounts of each and the frequency of related derivatives. In addition, the corresponding relationship between basic recipes and their clinical derivatives are presented in Table S3 in detail. **Notes:** The basic recipes are shown in Chinese Pin Yin, e.g., Maxingshigan Decoction, while each herb is presented as its Latin name (Chinese name, Chinese Pin Yin), e.g., Herba Ephedrae (麻黄, Ma Huang), etc. except a few use an English name without a Latin name, such as Poop (金汁, Jin Zhi), related to gut bacteria.

**Table 3:** The significant level of coherence (SLC) of the top 10 herbs correlated with COVID-19 on the target gene basis

NO.	Herbs	Number of Herb Targets	COVID-19 Genes	Number Overlapping	*P* Value	-Log10 P
1	Radix Glycyrrhizae (甘草, Gan Cao)	311	172	62	1.22E-36	35.92
2	Flos Lonicerae (金银花, Jin Yin Hua)	382	172	65	4.20E-30	29.38
3	Fructus Forsythiae (连翘, Lian Qiao)	356	172	62	9.91E-30	29.00
4	Herba Agastaches (藿香, Huo Xiang)	302	172	54	7.28E-27	26.14
5	Radix Astragali seu Hedysari (黄芪, Huang Qi)	325	172	54	7.15E-24	23.15
6	Semen Armeniacae Amarum (杏仁, Xing Ren)	298	172	51	1.25E-23	22.90
7	Radix Scutellariae (黄芩, Huang Qin)	230	172	40	5.04E-19	18.30
8	Herba Ephedrae (麻黄, Ma Huang)	889	172	82	1.76E-11	10.75
9	Rhizoma Phragmitis (芦根, Lu Gen)	125	172	18	9.22E-07	6.04
10	Gypsum Fibrosum (石膏, Shi Gao)	12	172	3	0.001455	2.84

The SLC is shown as -Log10 P, which was calculated by Chi-squared test. Here, we show crucial data, including herb name, herb target number, COVID-19 genes, number overlapping, p value and -Log10 P. These targets were all constrained with the TCMSP database. The herb targets were all from TCMSP, except for the unlisted Gypsum Fibrosum (12 targets were left out of 19 targets). COVID-19 target genes were obtained from NCBI and GeneCards (172 target genes remained out of 352 targets). The P value is shown in scientific notation. The data are partly shown in Fig. 3A.
