# Supplementary material for: Herbal plants coordinate COVID-19 in multiple dimensions - An insight analysis for clinically applied remedies
Source: Int J Med Sci. 2020 Oct 22;17(18):3125–45. doi: 10.7150/ijms.50260 (PMC7646114; doi:10.7150/ijms.50260)
Supplement: Supplementary file 1 — Supplementary figures and tables. [file ijmsv17p3125s1.pdf]

Table S1. The regional distribution of TCM coverage and curative rate nationwide.

NO.	Regions	TCM Coverage （%）	Cut-off Date	Source	Cumulative Cured Cases	Cumulative Confirmed Cases (Import Abroad Excluded)	Curative Rate (Cured Patients / Confirmed Cases) (%)
1	Beijing	87.00	2020.2.25	Beijing municipal Health Commission (http://wjw.beijing.gov.cn/)	334	415 (20)	80.48
2	Shanxi 1 （山西）	98.40	2020.3.10	National Administration of Traditional Chinese Medicine (http://www.satcm.gov.cn/)	132	133	99.25
3	Zhejing	97.76	2020.3.2	Zhejiang Administration of Traditional Chinese Medicine (http://www.zjtcm.gov.cn/)	1,209	1,215	99.51
4	Shandong	> 98.00	2020.3.5	National Administration of Traditional Chinese Medicine (http://www.satcm.gov.cn/)	734	760	96.58
5	Tianjin	99.00	2020.3.4	National Administration of Traditional Chinese Medicine (http://www.satcm.gov.cn/)	132	136	97.06
6	Henan	98.74	2020.2.27	National Administration of Traditional Chinese Medicine (http://www.satcm.gov.cn/)	1,249	1,273	98.11
7	Hubei	> 90.00	2020.3.6	National Administration of Traditional Chinese Medicine (http://www.satcm.gov.cn/)	50,316	67,781	74.23

8	Sichuan	93.20	2020.2.26	Sichuan administration of traditional Chinese medicine (http://sctcm.sc.gov.cn/)	496	539	92.02
9	Yunnan	100.00	2020.2.25	Yunnan administration of traditional Chinese medicine (ynswsjkw.yn.gov.cn)	170	174	97.70
10	Shanxi 2 （陕西）	93.50	2020.3.4	Shanxi administration of traditional Chinese medicine (http://atcm.shaanxi.gov.cn/)	232	245	94.69
11	Hunan	100.00	2020.3.4	Hu'nan provincial health commission (http://wjw.hunan.gov.cn/)	999	1,018	98.13
12	Gansu	97.80	2020.3.4	Health Commission of Gansu municipal (http://wsjk.gansu.gov.cn/)	88	91 (36)	96.70
13	Hainan	93.00	2020.2.27	National Administration of Traditional Chinese Medicine (http://www.satcm.gov.cn/)	160	168	95.24
14	Guangdong	93.54	2020.2.23	National Administration of Traditional Chinese Medicine (http://www.satcm.gov.cn/)	1,289	1,356	95.06
15	Jiangxi	95.00	2020.2.16	COVID 19 epidemic prevention and control work press conference of Jiangxi province (https://www.chinanews.com/sh/2020/02-16/9093640.shtml)	934	935	99.89

16	Guizhou	94.52	2020.2.25	Guizhou Administration of Traditional Chinese Medicine (http://atcm.guizhou.gov.cn/)	137	146	93.84
17	Heilongjiang	95.26	2020.2.18	Heilongjiang Administration of Traditional Chinese Medicine (http://news.cnr.cn/native/city/20200220/t20200220_524983029.shtml)	440	482	91.29
18	Jilin	100.00	2020.2.27	National Administration of Traditional Chinese Medicine (http://www.satcm.gov.cn/)	91	93	97.85
19	Liaoning	> 90.00	2020.3.4	National Administration of Traditional Chinese Medicine (http://www.satcm.gov.cn/)	111	125	88.80
20	Anhui	98.50	2020.2.20	National Administration of Traditional Chinese Medicine (http://www.satcm.gov.cn/)	984	990	99.39
21	Qinghai	unreported	unreported	unreported	18	18	100.00
22	Jiangsu	96.35	2020.3.11	Jiangsu Provincial Party Committee (http://www.jszzb.gov.cn/zyxw/info_110.aspx?itemid=29064)	629	631	99.68
23	Shanghai	95.00	2020.3.7	National Administration of Traditional Chinese Medicine (http://www.satcm.gov.cn/)	321	344	93.31
24	Chongqing	92.36	2020.3.4	National Administration of Traditional Chinese Medicine (http://www.satcm.gov.cn/)	554	576	96.18

25	Guangxi	97.60	2020.3.4	Guangxi zhuang autonomous region administration of traditional Chinese medicine (http://wsjkw.gxzf.gov.cn/)	241	252	95.63
26	Neimenggu	98.70	2020.3.12	Health commission of Inner Mongolia autonomous region (http://wjw.nmg.gov.cn/)	71	75	94.67
27	Suzhou, Jiangsu	100.00	2020.3.12	China News (http://www.js.chinanews.com/news/2020/0311/194630.html)	87	87	100.00
28	Fujian	99.00	2020.3.6	Health commission of Fujian province (http://wjw.fujian.gov.cn/)	295	296	99.66
29	Hebei	97.00	2020.2.29	National Administration of Traditional Chinese Medicine (http://www.satcm.gov.cn/)	310	318	97.48
30	Ningxia	98.60	2020.2.27	National Administration of Traditional Chinese Medicine (http://www.satcm.gov.cn/)	72	75	96.00
31	Shenzhen	95.70	2020.3.7	National Administration of Traditional Chinese Medicine (http://www.satcm.gov.cn/)	392	420	93.33
32	49 designated hospitals in Wuhan	94.74	2020.2.28	Health Commission of Hubei province (http://wjw.hubei.gov.cn/)			
33	43 designated hospitals in Hubei	97.71	2020.2.28	Health Commission of Hubei province (http://wjw.hubei.gov.cn/)			

34	16 *Fangcang hospitals*	99.93	2020.2.28	Health Commission of Hubei province (http://wjw.hubei.gov.cn/)

We developed COVID-19 epidemiology based on the openly published data from National Administration of Traditional Chinese Medicine largely, and the according websites were attached here. The regions nationwide, including provinces and the main hospitals. Provinces were noted by Chinese Pinyin by convention, and the two with same Pin Yin were differed by attaching numbers and Chinese characters. This included Shanxi 1 (山西) and Shanxi 2 (陕西). The confirmed and cured cases were recorded up to March 15, 2020. And curative rate was calculated out by them. In addition, confirmed cases imported abroad newly were excluded, including Beijing and Gansu, as shown in the brackets of "Cumulative confirmed cases (import abroad excluded)", e.g., Beijing: 415 (20), etc.

Table S2. The regional epidemiology nationwide in China.

NO.	Areas	Cumulative Confirmed Cases	Cumulative Cured Cases	Cure Rate(%)	Cumulative Death Toll	Fatal Rate(%)
**1**	**Nationwide**	**81753**	**73301**	**89.66154147**	**3283**	**4.015754774**
**2**	**Hubei region**	**67801**	**60323**	**88.97066415**	**3160**	**4.66069822**
**3**	**Non-hubei region**	**13952**	**12978**	**93.01892202**	**123**	**0.881594037**
4	Hubei	67801	60323	88.97066415	3160	4.66069822
5	Guangdong	1428	1333	93.34733894	8	0.56022409
6	Henan	1274	1250	98.11616954	22	1.726844584

7	Zhejiang	1240	1221	98.46774194	1	0.080645161
8	Hunan	1018	1014	99.60707269	4	0.392927308
9	Anhui	990	984	99.39393939	6	0.606060606
10	Jiangxi	936	934	99.78632479	1	0.106837607
11	Shandong	768	752	97.91666667	7	0.911458333
12	Jiangshu	636	631	99.21383648	0	0
13	Chongqing	578	570	98.61591696	6	1.038062284
14	Beijing	558	401	71.86379928	8	1.433691756
15	Sichuan	545	536	98.34862385	3	0.550458716
16	Heilongjiang	484	468	96.69421488	13	2.685950413
17	Shanghai	414	330	79.71014493	4	0.966183575
18	Hebei	319	310	97.17868339	6	1.880877743
19	Fujian	318	295	92.7672956	1	0.314465409
20	Guangxi	254	250	98.42519685	2	0.787401575
21	Shanxi 2(陕西)	249	240	96.38554217	3	1.204819277
22	Yunan	176	172	97.72727273	2	1.136363636
23	Hainan	168	162	96.42857143	6	3.571428571
24	Guizhou	146	144	98.63013699	2	1.369863014
25	Tianjing	142	133	93.66197183	3	2.112676056
26	Ganshu	136	118	86.76470588	2	1.470588235
27	Shanxi 1(山西)	134	133	99.25373134	0	0
28	Niaoling	127	124	97.63779528	2	1.57480315
29	Jiling	93	92	98.92473118	1	1.075268817
30	Xinjiang	76	73	96.05263158	3	3.947368421
31	Neimenggu	75	74	98.66666667	0	0
32	Ninxia	75	75	100	0	0

33	Qinghai	18	18	100	0	0
34	Xizhang	1	1	100	0	0
35	Hangkong	356	101	28.37078652	4	1.123595506
36	Taiwan	195	29	14.87179487	2	1.025641026
37	Macao	25	10	40	0	0

Up to March 24, 2020, the specific accumulative confirmed number, cure rate and fatal rate among the accumulative confirmed cases NO. Areas Confirmed Cumulative Cumulative Cure Rate Cumulative Fatal Rate were collected and calculated, which were divided into Hubei and non-Hubei in general. Provinces were noted by Chinese Pin Yin by convention, except for Hongkong and Macao, and the two with same Pin Yin were differed by attaching numbers and Chinese characters. This included Shanxi 1 (山西) and Shanxi 2 (陕西).

Table S3. The database of 185 clinically applied TCM remedies in details.

NO.	Formula Name	Composition of Herbs	References	Unified Phases	Basic Recipes	Herbs Number of Basic Recipes	Intersections	The Similarity（%）	Monarch Herb	Minister Herb	Correspond to Cytoscape
1	Pneumonia prophylaxis I (Modified	Radix Astragali seu Hedysari (黄芪, Huang Qi)15g,	Agreement on the prevention and treatment of	Prevention	Yupingfeng Powder	3	3	100.00	1	1	Pneumonia prophylaxis I

	Yupingfeng Powder)	Rhizoma Atractylodis Macrocephalae(白术, Bai Zhu)10g, Radix Saposhnikoviae(防风, Fang Feng)10g, RhizomaCyrtomiiFortunei(贯众, Guan Zhong)6g, Flos Lonicerae(金银花, Jin Yin Hua)10g, Pericarpium Citri Reticulatae(陈皮,Chen Pi)6g , Herba Eupatorii(佩兰, Pei Lan)10g	COVID-19 in Hubei hospital of traditional Chinese medicine (1st edition)							
2	Modified Xiaochaihu Decoction	Radix Bupleuri(柴胡, Chai Hu) 6g, Radix Scutellariae(黄芩, Huang Qin) 6g, Rhizoma Pinelliae(半夏, Ban Xia) 6g, Radix Codonopsis(党参,	The prevention and control of COVID-19 in Shanxi province (Trial)	Prevention	Xiaochaihu Decoction	7	6	85.70	1	1

		Dang Shen) 6g, Radix Saposhnikoviae(防风, Fang Feng) 6g, Fructus Forsythiae(连翘, Lian Qiao) 6g, Adenophora stricta Miq.(沙参, Sha Shen) 6g, Flos Lonicerae(金银花, Jin Yin Hua) 6g, Rhizoma Zingiberis Recens(生姜, Sheng Jiang) 6g, Radix Glycyrrhizae(甘草, Gan Cao) 6g									
3	Modified Yupingfeng Powder	Radix Astragali seu Hedysari(生黄芪, Sheng Huang Qi) 12g, Rhizoma Atractylodis Macrocephalae(白术, Bai Zhu) 9g, Radix Saposhnikoviae(防	The prevention and control of COVID-19 in Shanxi province(Trial)	Prevention	Yupingfeng Powder	3	3	100.00	1	1	3SX1

4	Modified Yinqiao Powder	风, Fang Feng) 9g, Herba Agastaches(藿香, Huo Xiang) 6g, Radix Glehniae(北沙参, Bei Sha Shen)12g, Flos Lonicerae(金银花, Jin Yin Hua) 9g, Bulbus Lilii(百合, Bai He) 12g, RhizomaCyrtomiiFortunei(贯众, Guan Zhong) 6g, Fructus Forsythiae(连翘, Lian Qiao) 9g Flos Lonicerae(金银花, Jin Yin Hua), Fructus Forsythiae(连翘, Lian Qiao), Herba Schizonepetae(荆芥, Jing Jie), Herba Menthae Heplocalycis(薄荷, Bo He), Radix	The prevention and control of COVID-19 in Hunan province (Trial version 3)	Prevention	Yinqiao Powder	10	6	60.00	1	2 月 4 日	4HuN

5	Modified Yupingfeng Powder	Glycyrrhizae(甘草, Gan Cao), Radix Isatidis(板蓝根, Ban Lan Gen), Cortex Mori(桑白皮, Sang Bai Pi), Rhizoma Phragmitis(芦根, Lu Gen) Radix Astragali seu Hedysari(生黄芪, Sheng Huang Qi)12g, Radix Saposhnikoviae(防风, Fang Feng)10g, Rhizoma Atractylodis Macrocephalae(白术, Bai Zhu)10g, Flos Lonicerae(金银花, Jin Yin Hua)10g, Fructus Forsythiae(连翘, Lian Qiao)10g, RhizomaCyrtomiiFortunei(贯众, Guan	The prevention and control of COVID-19 in Jiangxi province (Trial)	Treatment/ Mild Symptoms	Yupingfeng Powder	3	3	100.00	1	1	5JX

6	Hanshi(wen) Prescription	Zhong)6g, Herba Eupatorii(佩兰, Pei Lan)10g, Pericarpium Citri Reticulatae(陈皮, Chen Pi)10g, Rhizoma Atractylodis(苍术, Cang Shu)10g, Radix Platycodonis(桔梗, Jie Geng)10g Folium Perillae(紫苏叶,Zi Su Ye)6g, Herba Agastaches(藿香, Huo Xiang)叶 6g, Pericarpium Citri Reticulatae(陈皮,Chen Pi)9g, 煨 Fructus Tsaoko(草果,Cao Guo)6g, Rhizoma Zingiberis Recens(生姜,Sheng Jiang)3 片(寒湿重者, Rhizoma	Professor Tong Xiaolin	Prevention	Huoxiang Zhengqi Powder	10	3	30.00	1	1	6txl

7	Yinqiao Powder I(Close contact with historians)	Zingiberis Recens(生姜,Sheng Jiang)用 5~10 片) Flos Lonicerae(金银花, Jin Yin Hua)15g, Fructus Forsythiae(连翘, Lian Qiao)15g, Radix Astragali seu Hedysari(黄芪, Huang Qi)15g, Radix Saposhnikoviae(防风, Fang Feng)10g, Rhizoma Atractylodis Macrocephalae(白术, Bai Zhu)15g, Fructus Arctii(牛蒡子, Niu Bang Zi)15g, Rhizoma Phragmitis(芦根, Lu Gen)30g, Radix Glycyrrhizae(甘草, Gan Cao)6g	Recommendation plan for TCM treatment of COVID-19 in zhejiang province (Trial)	Prevention	Yinqiao Powder and Yupingfeng Powder	10/3	5/3	50.00/100.00	Yinqiao Powder：1 Yupingfeng Powder：1	Yinqiao Powder：1/4 Yupingfeng Powder：1	7ZJ
8		Radix Astragali seu	The prevention and	Prevention	Yupingfeng	3	3	100.00	1	1	8GS

	Hedysari(生黄芪, Sheng Huang Qi)15-30g, Rhizoma Atractylodis Macrocephalae(白术, Bai Zhu)15-30g, Radix Saposhnikoviae(防风, Fang Feng)6-9g, Rhizoma et Radix Notopterygii(羌活, Qiang Huo)3-6g, Herba Eupatorii(佩兰, Pei Lan)10-15g, Rhizoma Zingiberis Recens(生姜, Sheng Jiang)3-6g	control of COVID-19 in Gansu province (Trial)	of frail crowd	Powder					
9	Radix Bupleuri(柴胡, Chai Hu)18g, Radix Scutellariae(黄芩, Huang Qin)12g, Fructus Aurantii(枳壳, Zhi Qiao)12g, Radix	The prevention and control of COVID-19 in Tianjin	Prevention of close contact group	Chaihu Dayuan Decoction	10	10	100.00	1	1

Platycodonis(桔梗,
Jie Geng)10g,
Cortex Magnoliae
Officinalis(厚朴,
Hou Pu)12g, Semen
Arecae(槟榔, Bin
Lang)18g, Flos
Lonicerae(金银花,
Jin Yin Hua)15g,
RhizomaCyrtomiiF
ortunei(贯众, Guan
Zhong)10g
Fructus Tsaoko(草
果, Cao Guo)6g
Pericarpium Citri
Reticulatae
Viride(青皮, Qing
Pi)6g, Herba
Eupatorii(佩兰, Pei
Lan)10g,   lotus
petiole(荷梗, He
Geng)6g, Radix
Astragali seu
Hedysari(黄芪,
Huang Qi)18g ,
Radix

10	Hospital-made Prescription of TCM	Glycyrrhizae(甘草, Gan Cao)6g Herba Ephedrae(生麻黄, Sheng Ma Huang)6g, Gypsum Fibrosum(生石膏, Sheng Shi Gao)15g, Semen Armeniacae Amarum(杏仁, Xing Ren)9g, Rhizoma et Radix Notopterygii(羌活, Qiang Huo)15g, Semen Lepidii(葶苈子, Ting Li Zi)15g, RhizomaCyrtomiiFortunei(贯众, Guan Zhong)15g, Lumbricus(地龙, Di Long)15g, Radix Cynanchi Paniculati(徐长卿, Xu Chang Qing)15g, Herba Agastaches(藿香, Huo Xiang)15g,	The notification of the use of TCM agreement in the treatment of COVID-19	Prevention of COVID-19 suspects	Maxing Shigan Decoction and Dayuan Decoction	4/7	3/3	75.00/42.86	Maxing Yigan Decoction：1 Dayuan Decoction：1	Maxing Yigan Decoction：1 Dayuan Decoction：1	10txl

		Herba Eupatorii(佩兰, Pei Lan)9g, Rhizoma Atractylodis(苍术, Cang Shu)15g, Poria(茯苓, Fu Ling)45g, Rhizoma Atractylodis Macrocephalae(生白术, Sheng Bai Zhu)30g, (焦三仙, Jiao San Xian)各9g, Cortex Magnoliae Officinalis(厚朴, Hou Pu)15g, Semen Arecae(槟榔, Bin Lang)9g, Fructus Tsaoko(草果, Cao Guo)9g, Rhizoma Zingiberis Recens(生姜, Sheng Jiang)15g									
11	Modified Yupingfeng Powder	Radix Astragali seu Hedysari(生黄芪, Sheng Huang	Chinese medicine prevention plan of COVID-19 in	Prevention	Yupingfeng Powder	3	3	100.00	1	1	11SX2

	Qi)15g, Rhizoma Atractylodis Macrocephalae(炒白术, Chao Bai Zhu)10g, Radix Saposhnikoviae(防风, Fang Feng)6g, Bulbus Lilii(百合, Bai He)30g, Herba Dendrobii(石斛, Shi Hu)10g, pear peel(梨皮, Li Pi)30g, Radix Platycodonis(桔梗, Jie Geng)10g, Rhizoma Phragmitis(芦根, Lu Gen)30g, Radix Glycyrrhizae(生甘草, Sheng Gan Cao)6g	Shanxi province						
12	Radix Bupleuri(柴胡, Chai Hu)10g,Radix Astragali seu Hedysari(黄芪,	Zhong Nanshan's team	Shengjiang Powder	4	4	100.00	1	1

Huang Qi)10g, Semen Coicis(薏苡仁, Yi Yi Ren)15g,Rhizoma Atractylodis(苍术, Cang Shu)10g, Radix Ophiopogonis(麦冬, Mai Dong)15g, Radix Glehniae(北沙参, Bei Sha Shen)15g,Radix Glycyrrhizae(生甘草, Sheng Gan Cao)10g, Flos Lonicerae(金银花, Jin Yin Hua)15g,Bombyx Batryticatus(僵蚕, Jiang Can)10g, Periostracum Cicadae(蝉蜕, Chan Tui)5g,Radix et Rhizoma Rhei(大黄, Da Huang)5g,Rhizoma

13		Curcumae Longae(姜黄, Jiang Huang)10g Radix Astragali seu Hedysari(黄芪, Huang Qi)10g, Rhizoma Atractylodis Macrocephalae(炒白术, Chao Bai Zhu)10g, Radix Saposhnikoviae(防风, Fang Feng)6g, Radix Pseudostellariae(太子参, Tai Zi Shen)12g, Radix Ophiopogonis(麦冬, Mai Dong)10g, Fructus Forsythiae(连翘, Lian Qiao)10g, Flos Lonicerae(金银花, Jin Yin Hua)15g, Folium Perillae(紫苏叶, Zi Su Ye)6g,	The prevention and control of Winter and Spring Flu 2020 and COVID-19 in Shandong province	Prevention for healthy people	Yupingfeng Powder and Yinqiao Powder	3/10	3/3	100.00/30.00	Yupingfeng Powder：1 Yinqiao Powder：1	Yupingfeng Powder：1 Yinqiao Powder：0	13SD

14	Prevention of coronary heart disease	Radix Glycyrrhizae(甘草, Gan Cao)3g Radix Codonopsis(党参, Dang Shen)12g, Radix Ophiopogonis(麦冬, Mai Dong)9g, Fructus Schisandrae Chinensis(五味子, Wu Wei Zi)3g, Radix Salviae Miltiorrhizae(丹参, Dan Shen)9g, Flos Lonicerae(金银花, Jin Yin Hua)12g	The prevention and control of Winter and Spring Flu 2020 and COVID-19 in Shandong province	Prevention	Shengjiang Powder	3	3	100.00	1	1	14SD
15	Prevention of chronic respiratory diseases	Flos Lonicerae(金银花, Jin Yin Hua)9g, Radix Codonopsis(党参, Dang Shen)12g, Rhizoma Atractylodis Macrocephalae(炒白术, Chao Bai	The prevention and control of Winter and Spring Flu 2020 and COVID-19 in Shandong province	Prevention	Yupingfeng Powder	3	3	100.00	0	1	15SD

16	Partial cold constitution crowd square prevention square	Zhu)12g, Radix Saposhnikoviae(防风, Fang Feng)9g, Radix Glycyrrhizae(甘草, Gan Cao)6g Radix Astragali seu Hedysari(生黄芪,Sheng Huang Qi)15g, Rhizoma Atractylodis Macrocephalae(炒白术, Chao Bai Zhu)15g, Radix Saposhnikoviae(防风,Fang Feng)9g, Folium Perillae(紫苏叶,Zi Su Ye)9g, Herba Agastaches(藿香, Huo Xiang)9g, 炙 Radix Glycyrrhizae(甘草, Gan Cao)6g	The prevention and control of COVID-19 in Yunnan province (Trial)	Prevention	Yupingfeng Powder	3	3	100.00	1	1	16YN
17	Prevention for special	Radix Astragali seu Hedysari(生黄芪,	The prevention and control of	Prevention	Yupingfeng Powder	3	3	100.00	1	1	17HeN

	population 2	Sheng Huang Qi)15 克, Radix Saposhnikoviae(防风, Fang Feng)10 克, Rhizoma Atractylodis Macrocephalae(炒白术,Chao Bai Zhu)15 克, Radix Peucedani(前胡, Qian Hu)10 克, Herba Agastaches(藿香, Huo Xiang)10 克, Semen Coicis(生薏苡仁, Sheng Yi Yi Ren)15 克, Radix Glycyrrhizae(生甘草, Sheng Gan Cao)3 克	COVID-19 in Henan province (Trial)								
18		Cortex Mori(桑白皮, Sang Bai Pi)15 克, Cortex Lycii(地骨皮, Di Gu Pi)15 克, Radix Astragali seu Hedysari(生黄	The prevention and control of COVID-19 in Tianjin	Prevention	Xiebai Powder and Yupingfeng Powder	4/3	3/3	75.00/100.00	Xiebai Powder：1 Yupingfeng Powder：1	Xiebai Powder：1 Yupingfeng Powder：1	18TJ

		芪, Sheng Huang Qi)15 克, Rhizoma Phragmitis(芦根, Lu Gen)20 克, Radix Platycodonis(桔梗, Jie Geng)10 克, Rhizoma Atractylodis Macrocephalae(炒白术, Chao Bai Zhu)10 克, Radix Scrophulariae(玄参, Xuan She)20 克, Radix Scutellariae(黄芩, Huang Qin)10 克, Radix Glycyrrhizae(生甘草, Sheng Gan Cao)10 克, Radix Saposhnikoviae(防风, Fang Feng)10 克									
19	Guizhi Decoction,Yupingfeng	Radix Astragali seu Hedysari(黄芪, Huang Qi),	The prevention and control of COVID-19 in	Prevention	Huangqi Guizhi Wuwu	5/3	5/3	100.00/100.00	Huangqi Guizhi Wuwu	Huangqi Guizhi Wuwu	19HuN

	Powder,Modified Shenzhu Powder	Ramulus Cinnamomi(桂枝, Gui Zhi), Radix Paeoniae Alba(白芍, Bai Shao), Rhizoma Atractylodis(苍术, Cang Shu), Radix Saposhnikoviae(防风, Fang Feng), Radix Puerariae(葛根, Ge Gen), Rhizoma Zingiberis(干姜, Gan Jiang), Radix Glycyrrhizae(甘草, Gan Cao), Fructus Jujubae(大枣, Da Zao)	Hunan province (Trial version 3)		Decoction and Yupingfeng Powder				Decoction：1 Yupingfeng Powder：1	Decoction：1 Yupingfeng Powder：0	
20	Prevention for children with special constitution I	Radix Pseudostellariae(太子参, Tai Zi Shen)10 克, Radix Astragali seu Hedysari(生黄芪, Sheng Huang Qi)10	The prevention and control of COVID-19 in Henan province (Trial)	Prevention	Yupingfeng Powder	3	3	100.00	1	1	20HeN

		克, Radix Saposhnikoviae(防风, Fang Feng)6 克, Rhizoma Atractylodis Macrocephalae(炒白术, Chao Bai Zhu)10 克, Folium Eriobotryae(枇杷叶, Pi Pa Ye)3 克, RhizomaCyrtomiiFortunei(贯众, Guan Zhong)5 克, Fructus Jujubae(大枣, Da Zao)6 克, Radix Glycyrrhizae(甘草, Gan Cao)3 克								
21	Prevention for the elderly	Radix Codonopsis(党参, Dang Shen)10g, Poria(茯苓, Fu Ling)15g, Rhizoma Atractylodis Macrocephalae(炒白术, Chao Bai Zhu)9g, Radix	The prevention and control of Winter and Spring Flu 2020 and COVID-19 in Shandong province	Prevention	Shenling Baizhu Powder	11	3	27.30	2/3	0

22	Jinhuaqinggan Granule	Astragali seu Hedysari(黄芪, Huang Qi)12g, Bulbus Lilii(百合, Bai He)6g Flos Lonicerae(金银花, Jin Yin Hua), Gypsum Fibrosum(石膏, Shi Gao), Herba Ephedrae(麻黄, Ma Huang), Semen Armeniacae Amarum(杏仁, Xing Ren), Radix Scutellariae(黄芩, Huang Qin), Fructus Forsythiae(连翘, Lian Qiao), Bulbus Fritillariae Thunbergii(Bulbus Fritillariae Thunbergii(浙贝母, Zhe Bei Mu)母, Zhe Bei Mu), Rhizoma	1,The prevention and control of COVID-19 in Shanxi province (Trial)<br>2,Diagnosis and treatment of COVID-19 (Trial version 6)	Treatment/ Medical observation period	Maxing Shigan Decoction and Yinqiao Powder	4/10	4/5	100.00/50.00	Maxing Shigan Decoction：1<br>Yinqiao Powder：1	Maxing Shigan Decoction：1<br>Yinqiao Powder：2/4	22jhqg

23	Lianhuaqingwen Capsule (Granule)	Anemarrhenae(知母, Zhi Fu), Fructus Arctii(牛蒡子, Niu Bang Zi), Herba Artemisiae Annuae(青蒿, Qing Hao), Herba Menthae Heplocalycis(薄荷, Bo He), Radix Glycyrrhizae(甘草, Gan Cao) Fructus Forsythiae(连翘, Lian Qiao), Flos Lonicerae(金银花, Jin Yin Hua), Herba Ephedrae(麻黄, Ma Huang), Semen Armeniacae Amarum(杏仁, Xing Ren), Gypsum Fibrosum(石膏, Shi Gao), Radix Isatidis(板蓝根, Ban Lan Gen),	1, The prevention and control of COVID-19 in Shanxi province (Trial)<br>2, Diagnosis and treatment of COVID-19(Trial version 6)<br>3, The prevention and control of COVID-19 in Hainan province (The public version	Treatment Medical observation period	Maxing Shigan Decoction and Yinqiao Powder	4/10	4/2	100.00/20.00	Maxing Shigan Decoction：1 Yinqiao Powder：1	Maxing Shigan Decoction：1 Yinqiao Powder：1/4	23lhqw

		RhizomaCyrtomiiFortunei(贯众, Guan Zhong), Herba Houttuyniae(鱼腥草, Yu Xing Cao), Herba Agastaches(藿香, Huo Xiang), Radix et Rhizoma Rhei(大黄, Da Huang), Herba Rhodiolae(红景天, Hong Jingtian), Herba Menthae Heplocalycis(薄荷, Bo He)脑, Radix Glycyrrhizae(甘草, Gan Cao).Excipients are starch.	of the second edition of the trial)							
24	Fangfengtongsheng Pill (Granule)	Radix Saposhnikoviae(防风, Fang Feng), Herba Schizonepetae(荆芥, Jing Jie), Herba	The prevention and control of COVID-19 in Shanxi province (Trial) Diagnosis and	Treatment Medical observation period	Fangfeng Tongsheng Powder	17	17	100.00	1	1

Menthae Heplocalycis(薄荷, Bo He), Herba Ephedrae(麻黄, Ma Huang), Radix et Rhizoma Rhei(大黄, Da Huang), Natrii Sulfas(芒硝, Mang Xiao), Gardenia jasminoides Ellis(栀子, Zhi Zi), Talcum(滑石, Hua Shi), Radix Platycodonis(桔梗, Jie Geng), Gypsum Fibrosum(石膏, Shi Gao), Rhizoma Ligustici Chuanxiong(川芎, Chuan Qiong), Radix Angelicae Sinensis(当归, Dang Gui), Radix Scutellariae(黄芩, Huang Qin),	treatment of COVID-19(Trial version 4)

		Fructus Forsythiae(连翘, Lian Qiao), Radix Glycyrrhizae(甘草, Gan Cao), Radix Paeoniae Alba(白芍, Bai Shao), Rhizoma Atractylodis Macrocephalae(炒白术, Chao Bai Zhu).Coating accessory is Talcum.									
25	Huoxiang Zhengqi capsule (Pill,Water,oral liquid)	Rhizoma Atractylodis(苍术, Cang Shu), Pericarpium Citri Reticulatae(陈皮, Chen Pi), Cortex Magnoliae Officinalis(厚朴, Hou Pu), Radix Angelicae Dahuricae(白芷, Bai Zhi), Poria(茯	National Health Council/The prevention and control of COVID-19 in Hainan province (The public version of the second edition of the trial)/Shanxi Health Commission/Hunan Health Commission	Treatment Medical observation period	Huoxiang Zhengqi Powder	13	10	76.90	1	3/4	25 6/SX1/HuN/HaN

26	Prevention for adults (Professor Gu Xiaohong of BUCM)	苓, Fu Ling), Pericarpium Arecae(大腹皮, Da Fu Pi), Rhizoma Pinelliae(生半夏, Sheng Ban Xia), Radix Glycyrrhizae(甘草, Gan Cao), Herba Agastaches(藿香, Huo Xiang), Folium Perillae(紫苏叶, Zi Su Ye)<br>Radix Astragali seu Hedysari(生黄芪, Sheng Huang Qi)9g, Flos Lonicerae(金银花, Jin Yin Hua)9g, Fructus Forsythiae(连翘, Lian Qiao)9g, Herba Agastaches(藿香, Huo Xiang)6g, Rhizoma	Professor Gu Xiaohong	Prevention	Yinqiao Powder and Huoxiang Zhengqi Powder	10/13	4/6	40.00/46.15	Yinqiao Powder：1 Huoxiang Zhengqi Powder：1	Yinqiao Powder：0 Huoxiang Zhengqi Powder：2/4	26bucmg

27	Prevention for children and adolescents	Atractylodis(苍术, Cang Shu)6g, Cortex Magnoliae Officinalis(厚朴, Hou Pu)6g, Pericarpium Citri Reticulatae(陈皮, Chen Pi)6g, Poria(茯苓, Fu Ling)9g, Radix Platycodonis(桔梗, Jie Geng)6g, Rhizoma Phragmitis(芦根, Lu Gen)15g Herba Agastaches(藿香, Huo Xiang)3g, Semen Raphani(莱菔子, Lai Fu Zi)6g, Pericarpium Citri Reticulatae(陈皮, Chen Pi)3g, Poria(茯苓, Fu Ling)6g, Radix Platycodonis(桔梗,	Professor Gu Xiaohong	Prevention	Yinqiao Powder	10	4	40.00	1	0	27bucmg

28	Prevention (general population)	Jie Geng)3g, Flos Lonicerae(金银花, Jin Yin Hua)6g, Fructus Forsythiae(连翘, Lian Qiao)6g, Rhizoma Phragmitis(芦根, Lu Gen)9g, Radix Scrophulariae(玄参, Xuan She)6g Flos Lonicerae(金银花, Jin Yin Hua)10g Fructus Forsythiae(连翘, Lian Qiao)10g Radix Saposhnikoviae(防风, Fang Feng)10g Herba Menthae Heplocalycis(薄荷, Bo He)10g Herba Agastaches(藿香, Huo Xiang)10g Rhizoma Atractylodis	Technical guidelines for TCM prevention and control of COVID-19 in Sichuan province (Revised edition)	Prevention	Yinqiao Powder and Yupingfeng Powder	10/4	3/2	30.00/50.00	28SC

29	Prevention (for the weak)	Macrocephalae(生白术, Sheng Bai Zhu)10g Radix Astragali seu Hedysari(黄芪, Huang Qi)15g Radix Saposhnikoviae(防风, Fang Feng)10g Flos Lonicerae(金银花, Jin Yin Hua)10g Fructus Forsythiae(连翘, Lian Qiao)10g Herba Menthae Heplocalycis(薄荷, Bo He)10g Herba Agastaches(藿香, Huo Xiang)10g Rhizoma Atractylodis Macrocephalae(炒白术, Chao Bai Zhu)10g Fructus Aurantii(枳壳, Zhi Qiao)10g	Technical guidelines for TCM prevention and control of COVID-19 in Sichuan province (Revised edition)	Prevention	Yupingfeng Powder and Yinqiao Powder	3/10	3/3		100.00/30.00	Yupingfeng Powder：1 Yinqiao Powder：1	Yupingfeng Powder：1 Yinqiao Powder：1/4	29SC

30	Prevention (children)	Flos Lonicerae(金银花, Jin Yin Hua)10g, Fructus Forsythiae(连翘, Lian Qiao)10g, Herba Schizonepetae(荆芥, Jing Jie)10g, Herba Menthae Heplocalycis(薄荷, Bo He)10g, Radix Isatidis(板蓝根, Ban Lan Gen)10g, Rhizoma Phragmitis(芦根, Lu Gen)10g, Herba Agastaches(藿香, Huo Xiang)10g	Technical guidelines for TCM prevention and control of COVID-19 in Sichuan province (Revised edition)	Prevention	Yinqiao Powder	10	5	50.00	1	2/4	30SC
31	Pneumonia IV (Sini with Renshen Decoction, Angong Niuhuang Pills, Zixue Powder)	Radix Ginseng(人参, Ren Shen)10g, Radix Aconiti Lateralis Preparata(附子, Fu Zi)10g, Angong Niuhuang Pills(安宫牛黄丸) or Zixue	Agreement on the prevention and treatment of COVID-19 in hubei hospital of traditional Chinese medicine (1st edition)	Treatment Critical illness	Shenfu Decoction and Angong Niuhuang Pills or Zixue Dan	2/11 or 15	2/11 or 15	100.00/100.00 or 100.00	1/1/1	1/1/1	31pneumonia IV

32	Shengmai Powder, Sanshi Decoction, Angong Niuhuang Pills	Powder(紫雪散) Radix Panacis Quinquefolii(西洋参, Xi Yang Shen), Radix Ophiopogonis(麦冬, Mai Dong), Fructus Schisandrae Chinensis(五味子, Wu Wei Zi), Calcitum(寒水石, Han Shui Shi), Talcum(滑石, Hua Shi), Gypsum Fibrosum(生石膏, Sheng Shi Gao), 苦 Semen Armeniacae Amarum(杏仁, Xing Ren), Flos Lonicerae(金银花, Jin Yin Hua), Medulla Tetrapanacis(通草, Tong Cao), Caulis Bambusae in Taenia(竹茹, Zhu	The prevention and control of COVID-19 in Hunan province (Trial version 3)	Treatment Critical illness	Shengmai Powder and Sanshi Decoction	3/8	3/7	100.00/87.50	Shengmai Powder：1 Sanshi Decoction：1	Shengmai Powder：1<br>Sanshi Decoction：1

33	Modified Shenfu Decoction	Ru)<br>Radix Ginseng(生晒参, Sheng Shai Shen)(First decoction,stew second), Radix Aconiti Lateralis Preparata(附子, Fu Zi)(First decoction), Asparagus cochinchinensis(Lour.)Merr.(天冬, Tian Dong), Radix Ophiopogonis(麦冬, Mai Dong), Radix et Rhizoma Rhei(生大黄, Sheng Da Huang)(After decoction), Flos Lonicerae(金银花, Jin Yin Hua), Cornu Bubali(水牛角, Shui Niu Jiao)(First decoction), Fructus Corni(山茱萸, Shan Zhu Yu), Fructus	The prevention and control of COVID-19 in Shanxi province (Trial version 1)	Treatment/ Critical illness	Shenfu Decoction and Shengjiang Powder	2/3	2/3	100.00/100.00	Shenfu Decoction：1<br>Shengjiang Powder：1	Shenfu Decoction：1<br>Shengjiang Powder：1	33SX2

34	Sini with Renshen Decoction, Angong Niuhuang Pills, Zixue Dan	Schisandrae Chinensis(五味子,Wu Wei Zi), Rhizoma Phragmitis(芦根, Lu Gen), Radix Glycyrrhizae(生甘草, Sheng Gan Cao) Radix Ginseng(人参, Ren Shen), Radix Aconiti Lateralis Preparata(附子, Fu Zi), Fructus Corni(山茱萸, Shan Zhu Yu), tabasheer(天竺黄, Tian Zhuhuang)Decoction and Angong Niuhuang Pills(安宫牛黄丸) or Zixue Dan(紫雪丹) Smell with your nose	The prevention and control of COVID-19 in Gansu province (Trial)	Treatment/ Critical illness	Shenfu Decoction and Angong Niuhuang Pills or Zixue Dan	2/11or16	2/11or16	100.00/100.00 or 100.00	1/1/1	1/1/1	34GS
35		Radix Ginseng(人参, Ren Shen) 15g,	The prevention and control of	Treatment/ Critical	Shenfu Decoction	2/15or11or21	2/15or11or21	100.00/100.00 or	1/1/1/1	1/1/1/1	35SX1

		Radix Aconiti Lateralis Preparata(附子, Fu Zi) 10g(First decoction), Fructus Corni(山茱萸, Shan Zhu Yu) 15g, Suhexiang pills(苏合香丸) or Angong Niuhuang Pills(安宫牛黄丸) or Shexiang Niuhuang(麝香牛黄丸) Pills	COVID-19 in Shanxi province (Trial)	illness	and Suhexiang Pill or Angong Niuhuang Pills or Shexiang Niuhuang Pill			100.00			
36	Sini with Renshen Decoction (Shenfu Decoction),Angong Niuhuang Pills,Zixue Powder	Radix Ginseng(生晒参, Sheng Shai Shen)20-30g, Radix Aconiti Lateralis Preparata(附子, Fu Zi)(First decoction)30-60g, Fructus Corni(山茱萸, Shan Zhu Yu)15-20g, Angong Niuhuang Pills(安宫牛黄丸) or Zixue	The Prescription for the intervention of TCM intervention of COVID-19 in sichuan province (Trial version 1)	Treatment/ Critical illness	Shenfu Decoction and Angong Niuhuang Pills or Zixue Dan	2/11or15	2/11or15	100.00/100.00 or 100.00	1/1/1	1/1/1	36SC

		Powder.people of Yang burst can use Shenfu Injection by Intravenous injection									
37	Shenfulongmu Decoction	Radix Ginseng(人参, Ren Shen), Radix Aconiti Lateralis Preparata(附子, Fu Zi), Bone fossil of big mammals(煅龙骨, Duan Long Gu), Oyster(煅牡蛎, Duan Mu Li)	The prevention and control of COVID-19 in Hunan province (Trial version 3)	Treatment/ Critical illness	Shenfulong mu Decoction	4	4	100.00	1	1	
38	Modified Shenfu Decoction	Radix Ginseng Rubra(红参, Hong Shen)10g, Radix Aconiti Lateralis Preparata(附子, Fu Zi)10g(First decoction), Fructus Corni(山茱萸, Shan Zhu Yu)30g, Radix Ophiopogonis(麦冬, Mai Dong)20g,	The prevention and control of COVID-19 in Guangdong province (Trial version 1)	Treatment/ Critical illness （critical phase）	Shenfu Decoction	2	2	100.00	1	1	38GD

39		Radix Notoginseng(三七, San Qi)10g Radix Ginseng(人参, Ren Shen), Radix Aconiti Lateralis Preparata(附子, Fu Zi), Fructus Corni(山茱萸, Shan Zhu Yu), Angong Niuhuang Pills(安宫牛黄丸) or Zixue Powder(紫雪散)	The prevention and control of COVID-19 in Yunnan province (Trial)		Shenfu Decoction and Angong Niuhuang Pills or Zixue Dan	2/11or16	2/11or16	100.00/100 .00 or 100.00	1	1	39YN
40	Sini with Renshen Decoction (Shenfu Decoction),An gong Niuhuang Pills,Zixue Dan	Radix Ginseng(人参, Ren Shen), Radix Aconiti Lateralis Preparata(附子, Fu Zi), Fructus Corni(山茱萸, Shan Zhu Yu), Angong Niuhuang Pills(安宫牛黄丸) or Zixue Powder(紫雪散)	The prevention and control of COVID-19 in Jiangxi province (Trial)		Shenfu Decoction and Angong Niuhuang Pills or Zixue Dan	2/11or16	2/11or16	100.00/100 .00 or 100.00	1	1	40JX
41		Radix Ginseng(人	Diagnosis and	Treatment/	Shenfu	2/15or11	2/15or11	100.00/100	1	1	41 4

		参, Ren Shen)15g, Radix Aconiti Lateralis Preparata(附子, Fu Zi)10g(First decoction), Fructus Corni(山茱萸, Shan Zhu Yu)15g, Suhexiang pills(苏合香丸) or Angong Niuhuang Pills(安宫牛黄丸)	treatment of COVID-19(Trial version 4)	Critical illness	Decoction and Suhexiang Pill or Angong Niuhuang Pills			.00 or 100.00			
42	Shenfu Injection	Radix Ginseng Rubra(红参, Hong Shen), 附片(Radix Aconiti Lateralis Preparata(附子, Fu Zi).Excipients:Polysorbate 80g	1, The Prescription for the intervention of TCM intervention of COVID-19 in Sichuan province (Trial version 1) 2, Diagnosis and treatment of COVID-19(Trial version 6)	Treatment/ Critical illness	Shenfu Decoction	2	2	100.00	1	1	42SC/6
43	Shengmai Injection	Radix Ginseng Rubra(红参, Hong Shen), Radix	The prevention and control of COVID-19 in		Shengjiang Powder	3	3	100.00	1	1	43SX1/6

		Ophiopogonis(麦冬, Mai Dong), Fructus Schisandrae Chinensis(五味子,Wu Wei Zi)	Shanxi province (Trial) Diagnosis and treatment of COVID-19(Trial version 6)							
44	Shenmai Injection	Radix Ginseng Rubra(红参, Hong Shen), Radix Ophiopogonis(麦冬, Mai Dong)	Diagnosis and treatment of COVID-19(Trial version 6)	Shengjiang Powder	3	2	66.70	1	1	44 6
45	Xuebijing Injection,Shenfu Injection,Shengmai Injection	Flos Carthami(红花, Hong Hua), Radix Paeoniae Rubra(赤芍, Chi Shao), Rhizoma Ligustici Chuanxiong(川芎, Chuan Qiong), Radix Salviae Miltiorrhizae(丹参, Dan Shen), Radix Angelicae Sinensis(当归, Dang Gui), Radix Ginseng Rubra(红	The prevention and control of COVID-19 in Shanxi province (Trial)/Diagnosis and treatment of COVID-19(Trial version 5)	Shenfu Decoction and Shengjiang Powder	2/3	2/3	100.00/100.00	Shenfu Decoction：1<br>Shengjiang Powder：1	Shenfu Decoction：1<br>Shengjiang Powder：1	45SX1/5

46	Angong Niuhuang Pills	参, Hong Shen), 附片(Radix Aconiti Lateralis Preparata(附子, Fu Zi)), Radix Ophiopogonis(麦冬, Mai Dong), Fructus Schisandrae Chinensis(五味子,Wu Wei Zi) Calculus Bovis(牛黄, Niu Huang), Cornu Bubali(水牛角, Shui Niu Jiao)Concentrated Powder, Moschus(Moschus(麝香, She Xiang),She Xiang), Margarita(珍珠, Zhen Zhu), Cinnabaris(朱砂, Zhu Sha), Realgar(雄黄, Xiong Huang), Rhizoma	Angong Niuhuang Pills	11	11	100.00	1	1

47	Zixue Dan	Coptidis(黄连, Huang Lian), Radix Scutellariae(黄芩, Huang Qin), Gardenia jasminoides Ellis(栀子, Zhi Zi), Radix Curcumae(郁金, Yu Jin), Borneolum Syntheticum(冰片, Bing Pian) Gypsum Fibrosum(石膏, Shi Gao), Calcitum(寒水石, Han Shui Shi), Magnetitum(磁石, Ci Shi), Talcum(滑石, Hua Shi), Rhinoceros unicornis L. (犀角, Xi Jiao), Cornu Saigae Tataricae(羚羊角, Ling Yang Jiao), Radix Aucklandiae9(木香,	Zixue Dan	16	16	100.00	1	1

	Mu Xiang), Lignum Aquilariae Resinatum(沉香, chenxiang), Scrophularianingpoensis Hemsl.(元参, Yuan Shen), Rhizoma Cimicifugae(升麻, Sheng Ma), Radix Glycyrrhizae(甘草, Gan Cao), Flos Caryophylli(丁香, Ding Xiang), mirabilite(朴硝, Po Xiao), saltpetre(硝石, Xiao Shi), Moschus(Moschus(麝香, She Xiang),She Xiang), Cinnabaris(朱砂, Zhu Sha)								
48	Gypsum Fibrosum(生石膏, Sheng Shi Gao)30-60g(First	Diagnosis and treatment of COVID-19 (Trial version 7)	Treatment/ Critical illness	Qingwenbaidu Decoction	14	11	78.60	8/10	1

decoction), Rhizoma Anemarrhenae(知母, Zhi Fu)30g, Radix Rehmanniae Recens(生地, Sheng Di)30-60g, Cornu Bubali(水牛角, Shui Niu Jiao)30g(First decoction), Radix Paeoniae Rubra(赤芍, Chi Shao)30g, Radix Scrophulariae(玄参, Xuan She)30g, Fructus Forsythiae(连翘, Lian Qiao)15g, Moutan Cortex(丹皮, Dan Pi)15g, Rhizoma Coptidis(黄连, Huang Lian)6g, Lophatherum gracile(淡竹叶, Dan

49	Yinqiaohuopo Antipyretic (COVID-19 II)	Zhu Ye)12g, Semen Lepidii(葶苈子, Ting Li Zi)15g, Radix Glycyrrhizae(生甘草, Sheng Gan Cao)6g Flos Lonicerae(金银花, Jin Yin Hua)30g, Fructus Forsythiae(连翘, Lian Qiao)30g, Herba Schizonepetae(荆芥, Jing Jie)15g, Fructus Arctii(牛蒡子, Niu Bang Zi)15g, Herba Menthae Heplocalycis(薄荷, Bo He)15g, Radix Platycodonis(桔梗, Jie Geng)30g, Semen Armeniacae Amarum(杏仁, Xing Ren)15g,	Hospital of chengdu university of Chinese medicine (Sichuan central hospital)	Treatment	Yinqiao Powder and Huopu Xialing Decoction	10/11	7/7	70.00/63.60	Yinqiao Powder: 1, Huopu Xialing Decoction: 3/4	Yinqiao Powder: 3/4, Huopu Xialing Decoction: 1	49COVID192th

Herba
Agastaches(藿香,
Huo Xiang)15g,
Cortex Magnoliae
Officinalis(厚朴,
Hou Pu)15g,
Poria(茯苓, Fu
Ling)30g, 法
Rhizoma
Pinelliae(半夏, Ban
Xia)15g, Fructus
Amomi
Rotundus(Fructus
Amomi
Rotundus(豆蔻,
Dou Kou), Dou
Kou)15g, Semen
Coicis(薏苡仁, Yi
Yi Ren)30g, Semen
Dolichoris
Album(白扁豆, Bai
Bian Dou)30g,
Crataegus
pinnatifida(焦山楂,
Jiao Shan Zha)30g,
(建曲, Jian Qu)15g,

50	Yinqiao Powder, Qingwenbaidu Decoction, Lianhuaqingwen Granule and Pudilanxiaoyan tablet (liquid)	Rhizoma Phragmitis(芦根, Lu Gen)30g Fructus Forsythiae(连翘, Lian Qiao), Flos Lonicerae(金银花, Jin Yin Hua), Radix Platycodonis(桔梗, Jie Geng), Herba Menthae Heplocalycis(薄荷, Bo He)(After decoction), Fructus Arctii(牛蒡子, Niu Bang Zi), Lophatherum gracile(淡竹叶, Dan Zhu Ye), Rhizoma Phragmitis(芦根, Lu Gen), Semen Sojae Preparatum(淡豆豉, Dan Dou Chi), Herba Ephedrae(麻黄, Ma Huang),	The prevention and control of COVID-19 in Hainan province (The public version of the second edition of the trial)	Treatment/ Mild Symptoms	Yinqiao Powder and Maxing Shigan Decoction	10/4	9/4	90.00/100.00	Yinqiao Powder: 1<br>Maxing Shigan Decoction: 1	Yinqiao Powder: 3/4<br>Maxing Shigan Decoction: 1	50HaN

51	Sangju Decoction,Yinqiao Powder	Gypsum Fibrosum(生石膏, Sheng Shi Gao)(First decoction), Semen Armeniacae Amarum(杏仁, Xing Ren), Radix Bupleuri(柴胡, Chai Hu), Periostracum Cicadae(蝉蜕, Chan Tui), Radix Glycyrrhizae(甘草, Gan Cao) Folium Mori(桑叶, Sang Ye), Flos Chrysanthemi(菊花, Ju Hua), Radix Platycodonis(桔梗, Jie Geng), 苦 Semen Armeniacae Amarum(杏仁, Xing Ren), Fructus Forsythiae(连翘, Lian Qiao), Rhizoma	The prevention and control of COVID-19 in Hunan province (Trial version 3)	Treatment/ Fever in the early period	Sangju Decoction and Yinqiao Powder	8/10	8/9	100.00/90.00	Sangju Decoction: 1<br><br>Yinqiao Powder: 1	Sangju Decoction: 1 Yinqiao Powder: 3/4	51HuN

		Phragmitis(芦根, Lu Gen), Radix Glycyrrhizae(甘草, Gan Cao), Herba Menthae Heplocalycis(薄荷, Bo He), Flos Lonicerae(金银花, Jin Yin Hua), 淡 Lophatherum gracile(淡竹叶, Dan Zhu Ye), Herba Schizonepetae(荆芥, Jing Jie), Semen Sojae Preparatum(淡豆豉, Dan Dou Chi)									
52	Modified Maxing Yigan Decoction	Herba Ephedrae(麻黄, Ma Huang), Semen Armeniacae Amarum(杏仁, Xing Ren), Semen Coicis(薏苡仁,Yi Yi Ren), Radix Glycyrrhizae(生甘草, Sheng Gan	The prevention and control of COVID-19 in Jiangxi province (Trial)	Treatment/ Mild Symptoms	Maxing Yigan Decoction	4	4	100.00	1	1	52JX

		Cao), Radix Bupleuri(柴胡,Chai Hu), Radix Scutellariae(黄芩,Huang Qin), Fructus Forsythiae(连翘, Lian Qiao), RhizomaCyrtomiiFortunei(贯众,Guan Zhong), Folium Isatidis(大青叶,Da Qing Ye), Fructus Arctii(牛蒡子,Niu Bang Zi), Rhizoma Atractylodis(苍术,Cang Shu), Fructus Tsaoko(草果,Cao Guo)									
53	Huopu Xialing Decoction and Maxing Yigan Decoction,Qingkailing oral liquid,Tongxuan Lifei Pills	Herba Agastaches(藿香, Huo Xiang)6g, Rhizoma Pinelliae(半夏, Ban Xia)4.5g, light red Indian Bread(赤茯	The prevention and control of COVID-19 in Hainan province (The public version of the second edition of the trial)	Treatment/ Early stage/Progressive stage	Huopu Xialing Decoction and Maxing Yigan Decoction	11/4	11/4	100.00/100.00	Huopu Xialing Decoction: 1 Maxing Yigan Decoction:	Huopu Xialing Decoction: 1 Maxing Yigan Decoction	53HaN

苓, Chi Fu Ling)9g, Semen Armeniacae Amarum(杏仁, Xing Ren)9g, Semen Coicis(生薏苡仁,Sheng Yi Yi Ren)12g, Fructus Amomi Rotundus(Fructus Amomi Rotundus(豆蔻, Dou Kou), Dou Kou)3g, Medulla Tetrapanacis(通草,Tong Cao)3g, Polyporus Umbellatus(猪苓, Zhu Ling)9g, Semen Sojae Preparatum(淡豆豉, Dan Dou Chi)9g, Rhizoma Alismatis(泽泻, Ze Xie)4.5g, Cortex Magnoliae Officinalis(厚朴,

1 : 1

54	Ganlu Xiaodu micropills and Modified Huoxiang Zhengqi Powder	Hou Pu)3g, Herba Ephedrae(麻黄, Ma Huang)Qujie,Tangpao6g, Radix Glycyrrhizae(炙甘草, Gan Cao)3g Herba Agastaches(藿香, Huo Xiang)(After decoction), Folium Perillae(紫苏叶, Zi Su Ye), Radix Platycodonis(桔梗, Jie Geng), Herba Menthae Heplocalycis(薄荷, Bo He)(After decoction), Fructus Forsythiae(连翘, Lian Qiao), Rhizoma Phragmitis(芦根, Lu Gen), Talcum(滑石, Hua Shi)(Package Fried), Rhizoma	The prevention and control of COVID-19 in Shanxi province (Trial version 1)	Treatment/ Mild Symptoms	Huoxiang Zhengqi Powder or Maxing Yigan Decoction and Shengjiang Powder	13 or 4/4	8 or 4/4		61.54 or 100.00/100.00	Huoxiang Zhengqi Powder: 1	Huoxiang Zhengqi Powder: 2/3	54SX2

	Atractylodis Macrocephalae(白术, Bai Zhu), Poria(茯苓, Fu Ling), Pericarpium Citri Reticulatae(陈皮, Chen Pi), Cortex Magnoliae Officinalis(厚朴, Hou Pu), Radix Glycyrrhizae(生甘草, Sheng Gan Cao)；or select modified Maxing Yigan Decoction and Shengjiang Powder									
55	Herba Ephedrae(麻黄, Ma Huang)4g, Gypsum Fibrosum(石膏, Shi Gao)20g, Rhizoma Anemarrhenae(知母, Zhi Fu)9g, Semen Armeniacae Amarum(杏仁,	The prevention and control of COVID-19 in Beijin(Version 2)	Treatment for children	Maxing Shigan Decoction and Maxing Yigan Decoction	4/4	3/3	75.00/75.00	Maxing Shigan Decoction: 1,<br>Maxing Yigan Decoction: 1	Maxing Shigan Decoction: 1,<br>Maxing Yigan Decoction: 1	55BJ

56	Yinqiao PowderII(People with mild influenza-like symptoms)	Xing Ren)10g, Semen Coicis(薏苡仁, Yi Yi Ren)10g, Rhizoma Phragmitis(芦根, Lu Gen)10g, Radix Platycodonis(桔梗, Jie Geng)6g, Cortex Mori(桑白皮, Sang Bai Pi)10g, Flos Lonicerae(金银花, Jin Yin Hua)10g Flos Lonicerae(金银花, Jin Yin Hua)15g, Fructus Forsythiae(连翘, Lian Qiao)15g, Radix Scutellariae(黄芩, Huang Qin)15g, Herba Schizonepetae(荆芥, Jing Jie)10g, Folium Isatidis(大青叶, Da Qing Ye)12g, Rhizoma et	Recommendation plan for TCM treatment of COVID-19 in zhejiang province (Trial)	Treatment/ Mild Symptoms	Yinqiao Powder and Shengjiang Powder	10/4	7/2	70.00/50.00	Yinqiao Powder: 1, Shengjiang Powder: 1	Yinqiao Powder: 3/4, Shengjiang Powder: 0	56ZJ

		Radix Notopterygii(羌活, Qiang Huo)10g, Herba Menthae Heplocalycis(薄荷, Bo He)6g, Fructus Arctii(牛蒡子, Niu Bang Zi)15g, Periostracum Cicadae(蝉蜕, Chan Tui)6g, Bombyx Batryticatus(僵蚕, Jiang Can)10g, Rhizoma Phragmitis(芦根, Lu Gen)30g, Radix Lithospermi(紫草, Zi Cao)15g, Radix Glycyrrhizae(甘草, Gan Cao)6g									
57	Daqinglong Decoction and Modified Qianjinweijing Decoction	Herba Ephedrae(麻黄, Ma Huang), Ramulus Cinnamomi(桂枝, Gui Zhi), Semen Armeniacae	The prevention and control of COVID-19 in Shanxi province (Trial version 1)	Treatment/ Mild Symptoms	Mahuang Decoction and Maxing Shigan Decoction and Maxing	4/4	4/4	100.00/100.00/100.00	Mahuang Decoction: 1 Maxing Shigan Decoction:	Mahuang Decoction : 1 Maxing Shigan Decoction	57SX2

		Amarum(杏仁, Xing Ren), Gypsum Fibrosum(生石膏,Sheng Shi Gao)(First decoction), Rhizoma Phragmitis(芦根, Lu Gen), Semen Benincasae(冬瓜仁, Dong Gua Ren), Semen Persicae(桃仁, Tao Ren), Rhizoma Zingiberis Recens(生姜, Sheng Jiang), Semen Coicis(生薏苡仁, Sheng Yi Yi Ren), Fructus Jujubae(大枣, Da Zao), Radix Glycyrrhizae(生甘草,Sheng Gan Cao)			Yigan Decoction				1<br><br>Maxing Yigan Decoction: 1	: 1<br><br>Maxing Yigan Decoction : 1	
58	Fanggan Decoction	Radix Astragali seu Hedysari(黄芪, Huang Qi)20g, Rhizoma	The prevention and control of COVID-19 in Hainan province	Prevention	Yupingfeng Powder	3	3	100.00	1	1	58HaN

		Atractylodis Macrocephalae(白术, Bai Zhu)15g, Radix Saposhnikoviae(防风, Fang Feng)10g, Radix Paeoniae Rubra(赤芍, Chi Shao)10g, Fructus Forsythiae(连翘, Lian Qiao)10g, Radix Isatidis(板蓝根, Ban Lan Gen)15g, Radix Glycyrrhizae(甘草, Gan Cao)10g	(The public version of the second edition of the trial)							
59	Pneumonia I (Xiaochaihu Decoction and Sanren Decoction or Ganlu Xiaodu micropills)	Radix Bupleuri(柴胡, Chai Hu)24g, Radix Scutellariae(黄芩, Huang Qin)9g, Rhizoma Zingiberis Recens(生姜, Sheng Jiang)10g, Rhizoma Pinelliae(半夏, Ban Xia)12g, Semen	Agreement on the prevention and treatment of COVID-19 in hubei hospital of traditional Chinese medicine (1st edition)	Treatment/ Pneumonia period	Xiaochaihu Decoction and Sanren Decoction	7/8	5/5	71.43/62.50	Xiaochaihu Decoction: 1<br>Sanren Decoction: 1	Xiaochaihu Decoction: 1, Sanren Decoction: 1

		Armeniacae Amarum(杏仁, Xing Ren)15g, Fructus Amomi Rotundus(白 Fructus Amomi Rotundus(豆蔻, Dou Kou), Bai Dou Kou)10g, Semen Coicis(薏苡仁,Yi Yi Ren)30g, Lophatherum gracile(淡竹叶, Dan Zhu Ye)15g, Talcum(滑石, Hua Shi)15g, 土 Poria(茯苓, Fu Ling)30g, Radix Glycyrrhizae(生甘草, Sheng Gan Cao)10g									
60	Pneumonia II(Maxing Yigan Decoction,Xiaoxianxiong	Herba Ephedrae(麻黄, Ma Huang)10g, Semen Armeniacae Amarum(杏仁, Xing Ren)10g,	Agreement on the prevention and treatment of COVID-19 in hubei hospital of	Treatment/ Pneumonia period	Maxing Yigan Decoction, Xiaoxianxiong	4/3/8/9	4/3/3/4	100.00/100.00/37.50/44.44	Maxing Yigan Decoction: 1 Xiaoxianxi	Maxing Yigan Decoction: 1 Xiaoxianx	60pneumonia II

Decoction,Caoguo Zhimu Decoction)	Semen Coicis(薏苡仁,Yi Yi Ren)30g, Rhizoma Coptidis(黄连,Huang Lian)6g, Rhizoma Pinelliae(半夏,Ban Xia)10g, Fructus Trichosanthis(瓜蒌,Gua Lou)皮 10g, Fructus Tsaoko(草果,Cao Guo)10g, Rhizoma Anemarrhenae(知母,Zhi Fu)10g, Herba Houttuyniae(鱼腥草, Yu Xing Cao)15g, Radix Glycyrrhizae(生甘草, Sheng Gan Cao)10g , 白 Fructus Amomi Rotundus(Fructus Amomi Rotundus(豆蔻,	traditional Chinese medicine (1st edition)		Decoction,Caoguo Zhimu Decoction,Sanren Decoction		ong Decoction: 1 Caoguo Zhimu Decoction: 1<br>Sanren Decoction: 0	iong Decoction : 1 Caoguo Zhimu Decoction : 1/2<br>Sanren Decoction : 1

61	Influenza II (Yinqiao Powder and Modified Qingwen Baidu Powder)	Dou Kou), Dou Kou)9g Flos Lonicerae(金银花, Jin Yin Hua)10g, Fructus Forsythiae(连翘, Lian Qiao)10g, Herba Schizonepetae(荆芥, Jing Jie)10g, Fructus Arctii(牛蒡子, Niu Bang Zi)10g, Herba Menthae Heplocalycis(薄荷, Bo He)10g, Radix Glycyrrhizae(生甘草, Sheng Gan Cao)10g, Lophatherum gracile(淡竹叶, Dan Zhu Ye)10g, Rhizoma Phragmitis(芦根, Lu Gen)15g, Rhizoma	Agreement on the prevention and treatment of COVID-19 in hubei hospital of traditional Chinese medicine (1st edition)	Yinqiao Powder	10	8	80.00	1	3/4	61HB

62	Influenza I (Gegen Decoction or Chaige Jieji Decoction)	Coptidis(黄连, Huang Lian)6g Radix Puerariae(葛根, Ge Gen)15g, Herba Ephedrae(麻黄, Ma Huang)10g, Ramulus Cinnamomi(桂枝, Gui Zhi)6g, Radix Paeoniae Alba(白芍, Bai Shao)15g, Rhizoma Zingiberis Recens(生姜, Sheng Jiang)10g, Radix Glycyrrhizae(生甘草, Sheng Gan Cao)10g, Fructus Jujubae(大枣, Da Zao)10g, Flos Lonicerae(金银花, Jin Yin Hua)20g	Agreement on the prevention and treatment of COVID-19 in hubei hospital of traditional Chinese medicine (1st edition)	Treatment/ Mild Symptoms /Flu period	Gegen Decoction	7	7	100.00	1	1	
63	Maxing Shigan Decoction and Modified Yinqiao Powder	Herba Ephedrae(麻黄, Ma Huang), Semen Armeniacae Amarum(杏仁, Xing Ren), Gypsum	The prevention and control of COVID-19 in Gansu province (Trial)	Treatment	Maxing Shigan Decoction and Yinqiao Powder	4/10	3/2	75.00/20.00	Maxing Shigan Decoction: 1, Yinqiao	Maxing Shigan Decoction: 1, Yinqiao	63GS

		Fibrosum(石膏, Shi Gao), Flos Lonicerae(金银花, Jin Yin Hua), Fructus Forsythiae(连翘, Lian Qiao), Radix Scutellariae(黄芩, Huang Qin), Radix Curcumae(郁金,Yu Jin), Bulbus Fritillariae Thunbergii(Bulbus Fritillariae Thunbergii(浙贝母, Zhe Bei Mu)母, Zhe Bei Mu), Radix Paeoniae Rubra(赤芍, Chi Shao), Rhizoma Arisaematis Cum Bile(胆南星, Dan Nan Xing)							Powder: 1	Powder: 0	
64	Xuanbai Chengqi Decoction and	Semen Armeniacae Amarum(杏仁, Xing Ren), Gypsum	The prevention and control of COVID-19 in	Treatment	Xuanbai Chengqi Decoction	4	3	75.00	1	1/2	64GS

	Huanglian Jiedu Decoction, and Modified Xijiao Dihuang Decoction	Fibrosum(生石膏,Sheng Shi Gao), Rhizoma Arisaematis Cum Bile(胆南星, Dan Nan Xing), Radix et Rhizoma Rhei(大黄, Da Huang), Herba Ephedrae(麻黄, Ma Huang), Semen Lepidii(葶苈子,Ting Li Zi), Cornu Bubali(水牛角, Shui Niu Jiao), Semen Persicae(桃仁, Tao Ren), Radix Paeoniae Rubra(赤芍, Chi Shao), Radix Glycyrrhizae(生甘草, Sheng Gan Cao)	Gansu province (Trial)								
65	Maxing Yigan Decoction,Modified Sanren Decoction	Herba Ephedrae(麻黄, Ma Huang) 6g, Semen Armeniacae Amarum(杏仁, Xing Ren) 9g,	The prevention and control of COVID-19 in Shanxi province (Trial)	Treatment/ Preliminary stage	Maxing Shigan Decoction and Maxing Yigan	4/4/11	4/4/8	100.00/100.00/72.72	Maxing Shigan Decoction: 1/2, Maxing	Maxing Shigan Decoction: 1, Maxing	65SX1

Semen Coicis(薏苡仁, Yi Yi Ren) 18g, Fructus Amomi Rotundus(Fructus Amomi Rotundus(豆蔻, Dou Kou), Dou Kou) 9g, Herba Agastaches(藿香, Huo Xiang) 9g, Cortex Magnoliae Officinalis(厚朴, Hou Pu) 12g, Rhizoma Pinelliae(半夏, Ban Xia) 9g, Poria(茯苓, Fu Ling) 12g, Polyporus Umbellatus(猪苓, Zhu Ling) 9g, Radix Scutellariae(黄芩, Huang Qin)9g, Fructus Forsythiae(连翘, Lian Qiao) 12g, Radix	Decoction and Huopu Xialing Decoction	Yigan Decoction: 1,<br><br>Huopu Xialing Decoction: 1	Yigan Decoction : 1, Huopu Xialing Decoction : 1

66	Yinqiao Powder and Modified Maxing Ganshi decoction	Glycyrrhizae(甘草, Gan Cao) 6g Fructus Forsythiae(连翘, Lian Qiao), Flos Lonicerae(金银花, Jin Yin Hua), Radix Platycodonis(桔梗, Jie Geng), Herba Menthae Heplocalycis(薄荷, Bo He)(After decoction), Fructus Arctii(牛蒡子, Niu Bang Zi), Lophatherum gracile(淡竹叶, Dan Zhu Ye), Rhizoma Phragmitis(芦根, Lu Gen), Semen Sojae Preparatum(淡豆豉, Dan Dou Chi), Herba Ephedrae(麻黄, Ma Huang), Gypsum	The prevention and control of COVID-19 in Shanxi province (Trial version 1)	Treatment/ Mild Symptoms	Yinqiao Powder and Maxing Ganshi decoction	10/4	8/4		80.00/100.00	Yinqiao Powder: 1, Maxing Ganshi Decoction: 1	Yinqiao Powder: 3/4, Maxing Ganshi Decoction : 1	66SX2

67	Yinqiao Powder and Modified Qingwenbaidu Decoction	Fibrosum(生石膏, Sheng Shi Gao)(First decoction), Semen Armeniacae Amarum(杏仁, Xing Ren), Radix Bupleuri(柴胡, Chai Hu), Periostracum Cicadae(蝉蜕, Chan Tui), Radix Glycyrrhizae(生甘草, Sheng Gan Cao) Flos Lonicerae(金银花, Jin Yin Hua), Fructus Forsythiae(连翘, Lian Qiao), Herba Menthae Heplocalycis(薄荷, Bo He), Radix Platycodonis(桔梗, Jie Geng), Herba Schizonepetae(荆芥, Jing Jie), Fructus Arctii(牛蒡	The prevention and control of COVID-19 in Tianjin	Treatment/ Mild Symptoms	Yinqiao Powder	10	8	80.00	1	3/4	67TJ

68	Modified Maxing Shigan Decoction	子, Niu Bang Zi), Radix Scrophulariae(玄参, Xuan She), Moutan Cortex(丹皮, Dan Pi), Radix Scutellariae(黄芩, Huang Qin), Gypsum Fibrosum(石膏, Shi Gao), Rhizoma Phragmitis(芦根, Lu Gen), Radix Glycyrrhizae(甘草, Gan Cao) Gypsum Fibrosum(生石膏, Sheng Shi Gao)45g(First decoction), Herba Ephedrae(麻黄, Ma Huang)10g, Semen Armeniacae Amarum(杏仁, Xing Ren)10g, Flos Lonicerae(金银花,	The prevention and control of COVID-19 in Beijin(Version 2)	Treatment/ Critical illness	Maxing Shigan Decoction and Xuanbai Chengqi Decoction	4/4	3/4	75.00/100.00	Maxing Shigan Decoction: 1,<br><br>Xuanbai Chengqi Decoction: 1	Maxing Shigan Decoction : 1, Xuanbai Chengqi Decoction : 1	68BJ

Jin Yin Hua)15g,
Rhizoma
Anemarrhenae(知
母, Zhi Fu)10g,
Cornu Bubali(水牛
角, Shui Niu
Jiao)30g, Bulbus
Fritillariae
Thunbergii(Bulbus
Fritillariae
Thunbergii(浙贝母,
Zhe Bei Mu)母, Zhe
Bei Mu)10g,
Fructus
Trichosanthis(瓜蒌,
Gua Lou)30g,
Radix et Rhizoma
Rhei(生大黄,Sheng
Da
Huang)10g(After
decoction), Cortex
Magnoliae
Officinalis(厚朴,
Hou Pu)15g,
Lumbricus(地龙, Di
Long)20g, Semen

69	Maxing Ganshi decoction and Modified Xuanbai Chengqi Decoction	Lepidii(葶苈子, Ting Li Zi)20g, cRadix Paeoniae Rubra(赤芍, Chi Shao)20g, Radix Astragali seu Hedysari(生黄芪,ShengHuang Qi)20g Gypsum Fibrosum(生石膏, Sheng Shi Gao)(First decoction), Semen Armeniacae Amarum(杏仁, Xing Ren), Radix et Rhizoma Rhei(生大黄, Sheng Da Huang)(After decoction), Fructus Trichosanthis(瓜蒌, Gua Lou), Herba Ephedrae(麻黄, Ma Huang), Rhizoma Anemarrhenae(知	The prevention and control of COVID-19 in Shanxi province (Trial version 1)	Treatment/ Critical illness	Maxing Shigan Decoction and Xuanbai Chengqi Decoction	4/4	4/4	100.00/100.00	Maxing Shigan Decoction: 1, Xuanbai Chengqi Decoction: 1	Maxing Shigan Decoction: 1, Xuanbai Chengqi Decoction: 1	69SX2

70	Maxing Shigan Decoction,Yinqiao Powder	母, Zhi Fu), Radix Scutellariae(黄芩, Huang Qin), Rhizoma Phragmitis(芦根, Lu Gen), Radix Glycyrrhizae(生甘草, Sheng Gan Cao) Herba Ephedrae(麻黄, Ma Huang), Semen Armeniacae Amarum(杏仁, Xing Ren), Gypsum Fibrosum(石膏, Shi Gao), Cortex Mori(桑白皮, Sang Bai Pi), Flos Lonicerae(金银花, Jin Yin Hua), Fructus Forsythiae(连翘, Lian Qiao), Radix Scutellariae(黄芩, Huang Qin), Bulbus Fritillariae Thunbergii(Bulbus	The prevention and control of COVID-19 in Yunnan province (Trial)	Treatment	Maxing Shigan Decoction and Yinqiao Powder	4/10	4/3	100.00/30.00	Maxing Shigan Decoction: 1, Yinqiao Powder: 1	Maxing Shigan Decoction: 1, Yinqiao Powder: 0	70YN

71	Modified Huoxiang Zhengqi Powder	Fritillariae Thunbergii(浙贝母, Zhe Bei Mu)母, Zhe Bei Mu), Radix Glycyrrhizae(生甘草, Sheng Gan Cao) Rhizoma Atractylodis(苍术, Cang Shu) 15g, Pericarpium Citri Reticulatae(陈皮, Chen Pi) 9g, Rhizoma Pinelliae(半夏, Ban Xia) 9g, Cortex Magnoliae Officinalis(厚朴, Hou Pu) 9g, Herba Ephedrae(麻黄, Ma Huang) 6g, Herba Agastaches(藿香, Huo Xiang) 9g, Fructus Tsaoko(草果, Cao Guo) 6g, Rhizoma et Radix Notopterygii(羌活,	The prevention and control of COVID-19 in Shanxi province (Trial)	Treatment/ Preliminary stage	Huoxiang Zhengqi Powder	13	6	46.10	1	2/4	71SX1

72	Maxing Yigan Decoction,Shengjiang Powder,Dayuan Decoction	Qiang Huo) 9g, Rhizoma Coptidis(黄连, Huang Lian) 6g, Rhizoma Zingiberis Recens(生姜, Sheng Jiang) 9g Herba Ephedrae(麻黄, Ma Huang), Semen Armeniacae Amarum(杏仁, Xing Ren), Fructus Tsaoko(草果, Cao Guo), Semen Arecae(槟榔, Bin Lang), Periostracum Cicadae(蝉蜕, Chan Tui), Fructus Forsythiae(连翘, Lian Qiao), Rhizoma Atractylodis(苍术, Cang Shu), Radix Platycodonis(桔梗, Jie Geng), Radix Scutellariae(黄芩,	The prevention and control of COVID-19 in Yunnan province (Trial)	Treatment	Sanao Decoction and Dayuan Decoction and Yinqiao Powder	3/7/10	3/4/4	100.00/57.14/40.00	Sanao Decoction: 1, Dayuan Decoction: 1, Yinqiao Powder: 1/2	Sanao Decoction: 1, Dayuan Decoction: 1/2, Yinqiao Powder: 1/4	72YN

73	Maxing Shigan Decoction,Sang Bei Powder	Huang Qin), Fructus Arctii(牛蒡子, Niu Bang Zi), Radix Glycyrrhizae(生甘草, Sheng Gan Cao) Herba Ephedrae(麻黄, Ma Huang), Semen Armeniacae Amarum(杏仁, Xing Ren), Gypsum Fibrosum(石膏, Shi Gao), Cortex Mori(桑白皮, Sang Bai Pi), Bulbus Fritillariae Thunbergii(Bulbus Fritillariae Thunbergii(浙贝母, Zhe Bei Mu)母, Zhe Bei Mu), Radix Glycyrrhizae(生甘草, Sheng Gan Cao)	The prevention and control of COVID-19 in Hunan province (Trial version 3)	Treatment/ Critical illness	Maxing Shigan Decoction and SangBei Powder	4/2	4/2	100.00/100.00	Maxing Shigan Decoction: 1, SangBei Powder: 1	Maxing Shigan Decoction: 1, SangBei Powder: 1	73HuN
74	Modified Maxing Shigan Decoction	Herba Ephedrae(麻黄, Ma Huang), Semen Armeniacae	The prevention and control of COVID-19 in	Treatment	Maxing Shigan Decoction,	4/10/11	4/3/4	100.00/30.00/36.36	Maxing Shigan Decoction:	Maxing Shigan Decoction	74JX

Amarum(杏仁, Xing Ren), Gypsum Fibrosum(石膏, Shi Gao), Radix Glycyrrhizae(生甘草, Sheng Gan Cao), Flos Lonicerae(金银花, Jin Yin Hua), Fructus Forsythiae(连翘, Lian Qiao), Folium Isatidis(大青叶, Da Qing Ye), Radix Scutellariae(黄芩, Huang Qin), Bulbus Fritillariae Thunbergii(Bulbus Fritillariae Thunbergii(浙贝母, Zhe Bei Mu)母, Zhe Bei Mu), Rhizoma Atractylodis(苍术, Cang Shu), Talcum(滑石, Hua Shi), Herba	Jiangxi province (Trial)	Yinqiao Powder and Ganlu Xiaodu micropills	1,<br><br>Yinqiao Powder: 1, Ganlu Xiaodu micropills: 2/3	: 1, Yinqiao Powder: 0, Ganlu Xiaodu micropills : 1/3

75	Sangbei Zhisou Powder (empirical formula of professor Xiong Jibo)	Agastaches(藿香, Huo Xiang) Cortex Mori(桑白皮, Sang Bai Pi), Bulbus Fritillariae Thunbergii(Bulbus Fritillariae Thunbergii(浙贝母, Zhe Bei Mu)母, Zhe Bei Mu), Radix Stemonae(百部, Bai Bu), Radix Asteris(紫菀, Zi Wan), Rhizoma Cynanchi Stauntonii(白前, Bai Qian), Radix Platycodonis(桔梗, Jie Geng), Herba Schizonepetae(荆芥, Jing Jie), Pericarpium Citri Reticulatae(陈皮, Chen Pi), Semen Armeniacae Amarum(杏仁,	The prevention and control of COVID-19 in Hunan province (Trial version 3)	Treatment/ Fever in the early period	Sangbei Zhisou Powder（SangBei Powder and Zhisou Powder)	2/7	2/7	100.00/100.00	SangBei Powder: 1, Zhisou Powder: 1	SangBei Powder: 1, Zhisou Powder: 1

		Xing Ren), Radix Glycyrrhizae(甘草, Gan Cao)									
76	Xuanbai Chengqi Decoction and SangBei Powder	Semen Armeniacae Amarum(杏仁, Xing Ren), Gypsum Fibrosum(生石膏, Sheng Shi Gao), Fructus Trichosanthis(瓜蒌, Gua Lou), Radix et Rhizoma Rhei(大黄, Da Huang), Cortex Mori(桑白皮, Sang Bai Pi), Bulbus Fritillariae Thunbergii(Bulbus Fritillariae Thunbergii(浙贝母, Zhe Bei Mu)母, Zhe Bei Mu)	The prevention and control of COVID-19 in Hunan province (Trial version 3)	Treatment/ Critical illness	Xuanbai Chengqi Decoction and SangBei Powder	4/2	4/2	100.00/100.00	Xuanbai Chengqi Decoction: 1, SangBei Powder: 1	Xuanbai Chengqi Decoction: 1, SangBei Powder: 1	76HuN
77	Modified Xuanbai Chengqi Decoction	Semen Armeniacae Amarum(杏仁, Xing Ren), Gypsum Fibrosum(生石膏, Sheng Shi Gao),	The prevention and control of COVID-19 in Jiangxi province (Trial)	Treatment/ Critical illness	Xuanbai Chengqi Decoction	4	4	100.00	1	1	77JX

		Fructus Trichosanthis(瓜蒌, Gua Lou), Radix et Rhizoma Rhei(大黄, Da Huang), Semen Lepidii(葶苈子,Ting Li Zi), RhizomaCyrtomiiFortunei(贯众, Guan Zhong), Folium Isatidis(大青叶, Da Qing Ye), Semen Persicae(桃仁, Tao Ren), Rhizoma Imperatae(白茅根, Bai Mao Gen), Rhizoma Phragmitis(芦根, Lu Gen), Radix Glycyrrhizae(生甘草, Sheng Gan Cao)									
78	Modified Maxing Shigan Decoction	Herba Ephedrae(生/炙麻黄, Sheng/Zhi Ma Huang)respectively 6g, Semen	The prevention and control of COVID-19 in Shanxi province (Trial)	Treatment/ Progressive stage	Maxing Shigan Decoction and Xuanbai Chengqi	4/4	3/4	75.00/100.00	Maxing Shigan Decoction: 1, Xuanbai	Maxing Shigan Decoction: 1, Xuanbai	78SX1

		Armeniacae Amarum(杏仁, Xing Ren) 9g, Gypsum Fibrosum(生石膏, Sheng Shi Gao) 30g, Semen Lepidii(葶苈子, Ting Li Zi) 9g, Fructus Trichosanthis(瓜蒌, Gua Lou) 30g, Fructus Tsaoko(草果, Cao Guo) 6g, Semen Arecae(槟榔, Bin Lang) 12g, Rhizoma Atractylodis(苍术, Cang Shu) 9g, Semen Persicae(桃仁, Tao Ren) 9g, Radix et Rhizoma Rhei(生大黄, Sheng Da Huang) 6g			Decoction				Chengqi Decoction: 1	Chengqi Decoction : 1
79	COVID-19 III (Baihu	Gypsum Fibrosum(石膏, Shi	Agreement on the prevention and	Treatment /Pneumonia	Baihu Decoction	4/4	2/4	50.00/100.00	Baihu Decoction:	Baihu Decoction

Decoction and Ginseng Decoction and SiTu Decoction)	Gao)30g, Rhizoma Anemarrhenae(知母, Zhi Fu)10g, Rhizoma Dioscoreae(山药, Shan Yao)15g, Radix Panacis Quinquefolii(西洋参, Xi Yang Shen)5g, 土 Poria(茯苓, Fu Ling)30g, Radix et Rhizoma Rhei(土大黄,Tu Da Huang)10g, Rhizoma Bolbostemmatis(土贝母, Tu Bei Mu)10g, Radix et Rhizome Achyranthes (土牛膝,Tu Niuxi)10g, Lignum Sappan(苏木, Su Mu)10g, Eupolyphaga Seu Steleophaga(土	treatment of COVID-19 in hubei hospital of traditional Chinese medicine (1st edition)	period	and SiTu Decoction	1,<br>SiTu Decoction: 1	: 1, SiTu Decoction : 1

80	Jingfang Baidu Powder,Modified Huopu Xialing Decoction	鳖,Tu Bie)10g, Retinervus Citri Furctus(橘络, Ju Luo)15g, Semen Raphani(莱菔子, Lai Fu Zi)20g, Semen Lepidii(葶苈子, Ting Li Zi)15g, Retinervus Luffae Fructus(丝瓜络, Si Gua Luo)30g Herba Schizonepetae(荆芥, Jing Jie)15g, Radix Saposhnikoviae(防风, Fang Feng)15g, Rhizoma Ligustici Chuanxiong(川芎, Chuan Qiong)15g, Radix Angelicae Dahuricae(白芷, Bai Zhi)15g, Herba Menthae Heplocalycis(薄荷, Bo He)15g, Radix	Sichuan provincial administration of TCM/The prevention and control of COVID-19 in Sichuan province (Trial version 1)	Treatment/ Acute stage	Jingfang Baidu Powder and Huopu Xialing Decoction	11/11	5/7	45.45/63.64	Jingfang Baidu Powder: 1, Huopu Xialing Decoction: 1	Jingfang Baidu Powder: 1/4, Huopu Xialing Decoction: 1	80SC

Platycodonis(桔梗,
Jie Geng)30g,
Herba
Agastaches(藿香,
Huo Xiang)15g,
Folium Perillae(紫
苏叶, Zi Su Ye)15g,
Cortex Magnoliae
Officinalis(厚朴,
Hou Pu)15g,
Rhizoma
Atractylodis
Macrocephalae(白
术, Bai Zhu)30g,
Rhizoma
Pinelliae(半夏, Ban
Xia)15g, (建曲, Jian
Qu)15g, Semen
Coicis(薏苡仁, Yi
Yi Ren)30g,
Poria(茯苓, Fu
Ling)30g, Fructus
Amomi
Rotundus(Fructus
Amomi
Rotundus(豆蔻,

81	Modified Maxing Shigan Decoction	Dou Kou), Dou Kou)15g, Semen Armeniacae Amarum(杏仁, Xing Ren)15g, Crataegus pinnatifida(焦山楂, Jiao Shan Zha)30g, Semen Dolichoris Album(白扁豆, Bai Bian Dou)30g, Rhizoma Phragmitis(芦根, Lu Gen)30g Herba Ephedrae(麻黄, Ma Huang) 9-12g, Semen Armeniacae Amarum(杏仁, Xing Ren) 9g, Gypsum Fibrosum(生石膏, Sheng Shi Gao) 30g, Fructus Trichosanthis(瓜蒌, Gua Lou) 30g,	The prevention and control of COVID-19 in Shanxi province (Trial)	Treatment/ Progressive stage	Maxing Shigan Decoction and Xuanbai Chengqi Decoction	4/4	3/3	75.00/75.00	Maxing Shigan Decoction: 1, Xuanbai Chengqi Decoction: 1/2	Maxing Shigan Decoction: 1, Xuanbai Chengqi Decoction: 1	81SX1

		Radix Scutellariae(黄芩, Huang Qin) 12g, Cortex Mori(桑白皮, Sang Bai Pi) 30g, Bulbus Fritillariae Thunbergii(Bulbus Fritillariae Thunbergii(浙贝母, Zhe Bei Mu)母, Zhe Bei Mu) 9g, Folium Eriobotryae(枇杷叶, Pi Pa Ye) 9g, Adenophora stricta Miq.(沙参, Sha Shen) 12g, Periostracum Cicadae(蝉蜕, Chan Tui) 6g									
82	Maxing Yigan Decoction and Shengjiang Powder or Dayuan Decoction；　or	Herba Ephedrae(麻黄, Ma Huang), Semen Armeniacae Amarum(杏仁, Xing Ren), Fructus Tsaoko(草果, Cao	The prevention and control of COVID-19 in Gansu province (Trial)	Treatment	Maxing Yigan Decoction and Dayuan Decoction	4/7	2/3	50.00/42.86	Maxing Yigan Decoction: 1, Dayuan Decoction: 1	Maxing Yigan Decoction: 1/2, Dayuan Decoction	82GS

	Modified Qianghuo Shengshi Decoction	Guo), Cortex Magnoliae Officinalis(厚朴, Hou Pu), Semen Arecae(槟榔, Bin Lang), Periostracum Cicadae(蝉蜕, Chan Tui), Fructus Forsythiae(连翘, Lian Qiao), Rhizoma et Radix Notopterygii(羌活, Qiang Huo), Rhizoma Atractylodis(苍术, Cang Shu), Radix Platycodonis(桔梗, Jie Geng), Radix et Rhizoma Rhei(大黄, Da Huang)								: 1	
83	Wangshi Lianpu Drink,Huopu Xialing Decoction	Rhizoma Coptidis(黄连, Huang Lian), Cortex Magnoliae Officinalis(厚朴, Hou Pu), 法	The prevention and control of COVID-19 in Hunan province (Trial version 3)	Treatment/ Fever in the early period	Huopu Xialing Decoction	11	10	90.10	1	1	83HuN

Rhizoma
Pinelliae(半夏, Ban
Xia), Herba
Agastaches(藿香,
Huo Xiang),
Poria(茯苓, Fu
Ling), Polyporus
Umbellatus(猪苓,
Zhu Ling),
Talcum(滑石, Hua
Shi), Fructus
Amomi
Rotundus(Fructus
Amomi
Rotundus(豆蔻,
Dou Kou), Dou
Kou), Semen
Armeniacae
Amarum(杏仁,
Xing Ren), Semen
Coicis(薏苡仁, Yi
Yi Ren), Medulla
Tetrapanacis(通草,
Tong Cao),
Rhizoma
Alismatis(泽泻, Ze

84	Baihu Decoction and Modified Qingwen Baidu Powder	Xie) Gypsum Fibrosum(石膏, Shi Gao)30g(First decoction), Cornu Bubali(水牛角, Shui Niu Jiao)30g, Talcum(滑石, Hua Shi)10g, Radix Lithospermi(紫草, Zi Cao)15g, Radix Glycyrrhizae(生甘草, Sheng Gan Cao)6g, Flos Lonicerae(金银花, Jin Yin Hua)15g, Fructus Forsythiae(连翘, Lian Qiao)15g, Radix Scutellariae(黄芩, Huang Qin)15g, Radix Paeoniae Rubra(赤芍, Chi Shao)12g, Periostracum	Recommendation plan for TCM treatment of COVID-19 in zhejiang province (Trial)	Treatment	Qingying Decoction and Shengjiang Powder	9/4	3/4	30.00/100.00	Qingying Decoction: 1, Shengjiang Powder: 1	Qingying Decoction: 0, Shengjiang Powder: 1

85	Flu-like mild treatment	Cicadae(蝉蜕, Chan Tui)9g, Bombyx Batryticatus(僵蚕, Jiang Can)10g, Semen Armeniacae Amarum(杏仁, Xing Ren)10g, Semen Persicae(桃仁, Tao Ren)6g, Radix et Rhizoma Rhei(大黄, Da Huang)6g, Rhizoma Curcumae Longae(姜黄, Jiang Huang)6g, Rhizoma Phragmitis(芦根, Lu Gen)30g Flos Lonicerae(金银花, Jin Yin Hua)18g, Fructus Forsythiae(连翘, Lian Qiao)12g, Herba Ephedrae(麻黄, Ma Huang)6g , Semen Armeniacae Amarum(杏仁,	The prevention and control of Winter and Spring Flu 2020 and COVID-19 in Shandong province	Treatment/ Mild Symptoms	Yinqiao Powder and Maxing Shigan Decoction	10/4	3/4	30.00/100.00	Yinqiao Powder: 1, Maxing Shigan Decoction: 1	Yinqiao Powder: 0, Maxing Shigan Decoction : 1	85SD

86	Maxing Yigan Decoction, Shengjiang Powder, Dayuan Decoction	Xing Ren)9g, Gypsum Fibrosum(生石膏, Sheng Shi Gao)15g, Radix Glycyrrhizae(生甘草, Sheng Gan Cao)3g Herba Ephedrae(麻黄, Ma Huang)10g, Semen Armeniacae Amarum(杏仁, Xing Ren)15g, Fructus Tsaoko(草果, Cao Guo)10-20g, Semen Arecae(槟榔, Bin Lang)10-15g, Periostracum Cicadae(蝉蜕, Chan Tui)5-10g, Fructus Forsythiae(连翘, Lian Qiao)10-30g, Rhizoma Atractylodis(苍术, Cang Shu)10-15g,	Sichuan provincial administration of TCM/The prevention and control of COVID-19 in Sichuan province (Trial version 1)	Treatment/ Acute stage	Sanao Decoction, Dayuan Decoction and Yinqiao Powder	3/7/10	3/4/4	100.00/57.14/40.00	Sanao Decoction: 1, Dayuan Decoction: 1, Yinqiao Powder: 1/2	Sanao Decoction: 1, Dayuan Decoction 1/2, Yinqiao Powder: 1/4	86SC

87	Qingfei Pai du mixture (Pneumonia I)	Radix Platycodonis(桔梗, Jie Geng)20-30g, Radix Scutellariae(黄芩, Huang Qin)15g, Fructus Arctii(牛蒡子, Niu Bang Zi)15g, Radix Glycyrrhizae(生甘草, Sheng Gan Cao)5-10g Herba Ephedrae(麻黄, Ma Huang)9g, Radix Glycyrrhizae(甘草, Gan Cao)6g, Semen Armeniacae Amarum(杏仁, Xing Ren)9g, Gypsum Fibrosum(生石膏, Sheng Shi Gao)15-30g(First decoction), Ramulus	Southwest medical medicine hospital/Diagnosis and treatment of COVID-19 (Trial version 6)	Treatment	Maxing Shigan Decoction and Wuling Powder and Xiaochaihu Decoction and Shegan Mahuang Decoction	4/5/7/9	4/5/6/6	100.00/100.00/85.71/66.67	Maxing Shigan Decoction: 1, Wuling Powder: 1, Xiaochaihu Decoction: 1, Sheganmahuang Decoction: 1	Maxing Shigan Decoction: 1, Wuling Powder: 1, Xiaochaihu Decoction: 1, Sheganmahuang Decoction	87qfpd

Cinnamomi(桂枝,
Gui Zhi)9g,
Rhizoma
Alismatis(泽泻, Ze
Xie)9g, Polyporus
Umbellatus(猪苓,
Zhu Ling)9g,
Rhizoma
Atractylodis
Macrocephalae(白
术, Bai Zhu)9g,
Poria(茯苓, Fu
Ling)15g, Radix
Bupleuri(柴胡, Chai
Hu)16g, Radix
Scutellariae(黄芩,
Huang Qin)6g, 姜
Rhizoma
Pinelliae(半夏, Ban
Xia)9g, Rhizoma
Zingiberis
Recens(生姜, Sheng
Jiang)9g, Radix
Asteris(紫菀, Zi
Wan)9g, Flos
Farfarae(冬花,

: 1

88	Qingqi Huatan Decoction,Modified Huopu Xialing Decoction	Dong Hua)9g, Rhizoma Belamcandae(射干, She Gan)9g, Asarum sieboldii Miq.(细辛, Xi Xin)6g, Rhizoma Dioscoreae(山药, Shan Yao)12g, Fructus Aurantii Immaturus(枳实, Zhi Shi)6g, Pericarpium Citri Reticulatae(陈皮, Chen Pi)6g, Herba Agastaches(藿香, Huo Xiang)9g Pericarpium Citri Reticulatae(陈皮, Chen Pi)10-15g, Semen Armeniacae Amarum(杏仁, Xing Ren)10-15g, Radix Scutellariae(黄芩, Huang Qin)10-15g,	Sichuan provincial administration of TCM/The prevention and control of COVID-19 in Sichuan province (Trial version 1)	Treatment/ Acute stage	Qingqi Huatan Decoction and Huopu Xialing Decoction	8/11	5/4	62.50/36.36	Qingqi Huatan Decoction : 0, Huopu Xialing Decoction: 3/4	Qingqi Huatan Decoction ： 2/3 , Huopu Xialing Decoction : 1/3	88SC

		Fructus Trichosanthis(瓜蒌, Gua Lou)皮 10-15g, Poria(茯苓, Fu Ling)15-30g, Herba Agastaches(藿香, Huo Xiang)15g, Cortex Magnoliae Officinalis(厚朴, Hou Pu)10-20g, Herba Artemisiae Annuae(青蒿, Qing Hao)20-30g, Rhizoma Phragmitis(芦根, Lu Gen)20-30g, Flos Lonicerae(金银花, Jin Yin Hua)15-30g, Radix Pseudostellariae(太子参, Tai Zi Shen)30g, Radix Glycyrrhizae(生甘草, Sheng Gan Cao)5-10g								
89	Sanren	Semen Armeniacae	The prevention and	Treatment/	Sanren	8/4	8/3	100.00/75.	Sanren	Sanren

Decoction and Modified Shengjiang Powder	Amarum(杏仁, Xing Ren), Semen Coicis(薏苡仁,Yi Yi Ren), Fructus Amomi Rotundus(Fructus Amomi Rotundus(豆蔻, Dou Kou), Dou Kou), Talcum(滑石, Hua Shi), Medulla Tetrapanacis(通草, Tong Cao), Lophatherum gracile(淡竹叶, Dan Zhu Ye), Rhizoma Pinelliae(半夏, Ban Xia), Cortex Magnoliae Officinalis(厚朴, Hou Pu), Rhizoma Alismatis(泽泻, Ze Xie), Radix et Rhizoma Rhei(大黄, Da Huang), Bombyx	control of COVID-19 in Tianjin	Mild Symptoms	Decoction and Shengjiang Powder		00	Decoction: 1, Shengjiang Powder: 1	Decoction: 1, Shengjiang Powder: 1/2

90	Xuanbai Chengqi Decoction,Huanglian Jiedu Decoction and Modified Jiedu Huoxue Decoction	Batryticatus(僵蚕, Jiang Can), Periostracum Cicadae(蝉蜕, Chan Tui), Poria(茯苓, Fu Ling), Radix Platycodonis(桔梗, Jie Geng), Rhizoma Cynanchi Stauntonii(白前, Bai Qian) Herba Ephedrae(麻黄, Ma Huang)8g, Semen Armeniacae Amarum(杏仁, Xing Ren)12g, Gypsum Fibrosum(生石膏, Sheng Shi Gao)30g, Radix et Rhizoma Rhei(生大黄, Sheng Da Huang)10g, Fructus Trichosanthis(瓜蒌, Gua Lou)仁 30g, Semen Persicae(桃	The prevention and control of COVID-19 in Guangdong province (Trial version 1)	Treatment/ Progressive stage	Maxing Shigan Decoction and Xuanbai Chengqi Decoction	4/4	3/4	75.00/100.00	Maxing Shigan Decoction: 1, Xuanbai Chengqi Decoction: 1	Maxing Shigan Decoction: 1, Xuanbai Chengqi Decoction: 1	90GD

仁, Tao Ren)10g, Radix Paeoniae Rubra(赤芍, Chi Shao)15g, Semen Lepidii(葶苈子, Ting Li Zi)20g, Rhizoma Coptidis(黄连, Huang Lian)3g, Radix Scutellariae(黄芩, Huang Qin)10g, Cortex Mori(桑白皮, Sang Bai Pi)10g, Rhizoma Paridis(重楼, Chong Lou)10g, Moutan Cortex(丹皮, Dan Pi)15g, Radix Curcumae(郁金, Yu Jin)15g, Rhizoma Acori Tatarinowii(石菖蒲, Shi Chang Pu)15g, Radix Rehmanniae Recens(生地, Sheng

91	Modified Maxing Shigan Decoction	Di)15g, Radix Scrophulariae(玄参, Xuan She)15g Semen Armeniacae Amarum(杏仁, Xing Ren)10g, Gypsum Fibrosum(生石膏, Sheng Shi Gao)30g, Fructus Trichosanthis(瓜蒌,Gua Lou)30g, Radix et Rhizoma Rhei(生大黄, Sheng Da Huang)6g(After decoction),Herba Ephedrae(生/炙麻黄, Sheng/Zhi Ma Huang) respectively 6g, Semen Lepidii(葶苈子,Ting Li Zi)10g, Semen Persicae(桃仁, Tao Ren)10g, Fructus Tsaoko(草果, Cao Guo)6g,	Diagnosis and treatment of COVID-19(Trial version 5)	Treatment/ Progressive stage	Xuanbai Chengqi Decoction and Maxing Shigan Decoction	4/4	4/3	100.00/75.00	Xuanbai Chengqi Decoction: 1, Maxing Shigan Decoction: 1	Xuanbai Chengqi Decoction: 1, Maxing Shigan Decoction: 1	91 5

92	Maxing Shigan Decoction,Yinqiao Powder	Semen Arecae(槟榔, Bin Lang)10g, Rhizoma Atractylodis(苍术, Cang Shu)10g Herba Ephedrae(麻黄, Ma Huang)10g, Semen Armeniacae Amarum(杏仁, Xing Ren)10-15g, Gypsum Fibrosum(石膏, Shi Gao)20-30g, Cortex Mori(桑白皮, Sang Bai Pi)15g, Flos Lonicerae(金银花, Jin Yin Hua)20-30g, Fructus Forsythiae(连翘, Lian Qiao)20-30g, Radix Scutellariae(黄芩, Huang Qin)15g, Bulbus Fritillariae Thunbergii(Bulbus Fritillariae	Sichuan provincial administration of TCM/The prevention and control of COVID-19 in Sichuan province (Trial version 1)	Treatment/ Acute stage	Maxing Shigan Decoction and Yinqiao Powder	4/10	4/3	100.00%/30.00%	Maxing Shigan Decoction: 1, Yinqiao Powder: 1	Maxing Shigan Decoction: 1, Yinqiao Powder: 0	92SC

93	Maxing Shigan Decoction,Ganlu Xiaodu micropills and Modified Shengjiang Powder	Thunbergii(浙贝母, Zhe Bei Mu)母, Zhe Bei Mu)15g, Radix Glycyrrhizae(生甘草, Sheng Gan Cao)5-10g Herba Ephedrae(生麻黄, Sheng Ma Huang)8g, Semen Armeniacae Amarum(杏仁, Xing Ren)12g, Gypsum Fibrosum(生石膏, Sheng Shi Gao)30g, Radix Glycyrrhizae(生甘草, Sheng Gan Cao)10g, Talcum(滑石, Hua Shi)30g, Herba Artemisiae Scopariae(茵陈, Yin Chen)20g, Radix Scutellariae(黄芩, Huang Qin)15g,	The prevention and control of COVID-19 in Guangdong province (Trial version 1)	Treatment/ Progressive stage	Maxing Shigan Decoction and Ganlu Xiaodu micropills and Shengjiang Powder and Xijiao Dihuang Decoction	4/11/4/4	4/5/4/3	100.00/45.45/100.00/100.00	Maxing Shigan Decoction: 1, Ganlu Xiaodu micropills: 1, Shengjiang Powder: 1, Xijiao Dihuang Decoction: 0	Maxing Shigan Decoction: 1, Ganlu Xiaodu micropills: 1, Shengjiang Powder: 1, Xijiao Dihuang Decoction: 1	93GD

Fructus Amomi
Rotundus(Fructus
Amomi
Rotundus(豆蔻,
Dou Kou), Dou
Kou)10g(After
decoction), Herba
Agastaches(藿香,
Huo Xiang)15g, 法
Rhizoma
Pinelliae(半夏, Ban
Xia)15g, Rhizoma
Atractylodis(苍术,
Cang Shu)15g,
Semen Lepidii(葶苈
子, Ting Li Zi)20g,
Fructus
Forsythiae(连翘,
Lian Qiao)15g, 白
Bombyx
Batryticatus(僵蚕,
Jiang Can)5g,
Periostracum
Cicadae(蝉蜕, Chan
Tui)5g, Rhizoma
Curcumae

		Longae(姜黄, Jiang Huang)10g, Radix et Rhizoma Rhei(生大黄, Sheng Da Huang)5g, Rhizoma Paridis(重楼, Chong Lou)10g, Moutan Cortex(丹皮, Dan Pi)15g, Radix Paeoniae Rubra(赤芍, Chi Shao)15g, Radix Curcumae(郁金, Yu Jin)15g, Rhizoma Acori Tatarinowii(石菖蒲, Shi Chang Pu)15g, Radix Rehmanniae Recens(生地, Sheng Di)15g, Radix Scrophulariae(玄参, Xuan She)15g								
94	Huopu Xialing Decoction and Modified Xiaochaihu	Herba Agastaches(藿香, Huo Xiang)10g(After	The prevention and control of COVID-19 in Guangdong	Treatment/ Preliminary stage	Huopu Xialing Decoction and	11/7	11/6	100.00/85.71	Huopu Xialing Decoction: 1,	Huopu Xialing Decoction : 1,	94GD

Decoction	decoction), Cortex Magnoliae Officinalis(厚朴, Hou Pu)10g, 法 Rhizoma Pinelliae(半夏, Ban Xia)10g, Poria(茯苓, Fu Ling)15g, Radix Bupleuri(柴胡, Chai Hu)15g, Radix Scutellariae(黄芩, Huang Qin)10g, Radix Codonopsis(党参, Dang Shen)10g, Semen Armeniacae Amarum(杏仁, Xing Ren)10g, Semen Coicis(薏苡仁, Yi Yi Ren)20g, Polyporus Umbellatus(猪苓, Zhu Ling)10g, Rhizoma Alismatis(泽泻, Ze	province (Trial version 1)	Xiaochaihu Decoction	Xiaochaihu Decoction: 1	Xiaochaihu Decoction: 1

		Xie)10g, Fructus Amomi Rotundus(Fructus Amomi Rotundus(豆蔻, Dou Kou), Dou Kou)10g(After decoction), Semen Sojae Preparatum(淡豆豉, Dan Dou Chi)10g, Medulla Tetrapanacis(通草, Tong Cao)10g, Rhizoma Zingiberis Recens(生姜, Sheng Jiang)5g, Fructus Jujubae(大枣, Da Zao)5g									
95	Baihu Decoction and Qingying Decoction and Modified Qingwenbaidu Decoction	Gypsum Fibrosum(生石膏, Sheng Shi Gao), Rhizoma Anemarrhenae(知母, Zhi Fu), Radix Scrophulariae(玄参,	The prevention and control of COVID-19 in Tianjin	Treatment/ Critical illness	Qingwenbaidu Decoction and Yinqiao Powder	14/10	10/5	71.43/50.00	Qingwenbaidu Decoction : 8/10, Yinqiao Powder : 1	Qingwenbaidu Decoction : 8/10, Yinqiao Powder : 1/4	95TJ

Xuan She), Radix
Rehmanniae
Recens(生地, Sheng
Di), Moutan
Cortex(丹皮, Dan
Pi), Lophatherum
gracile(淡竹叶, Dan
Zhu Ye), Flos
Lonicerae(金银花,
Jin Yin Hua),
Fructus
Forsythiae(连翘,
Lian Qiao), Radix
Scutellariae(黄芩,
Huang Qin),
Gardenia
jasminoides Ellis(栀
子, Zhi Zi), Herba
Menthae
Heplocalycis(薄荷,
Bo He), Radix
Paeoniae Rubra(赤
芍, Chi Shao),
Radix Curcumae(郁
金, Yu Jin), Semen
Lepidii(葶苈子,

96	Jingfanghuopu Mixture (COVID-19 III)	Ting Li Zi), Radix Glycyrrhizae(甘草, Gan Cao) Herba Schizonepetae(荆芥, Jing Jie)15g, Radix Saposhnikoviae(防风, Fang Feng)15g, Rhizoma Ligustici Chuanxiong(川芎, Chuan Qiong)15g, Radix Angelicae Dahuricae(白芷, Bai Zhi)15g, Herba Menthae Heplocalycis(薄荷, Bo He)15g, Radix Platycodonis(桔梗, Jie Geng)30g, 广 Herba Agastaches(藿香, Huo Xiang)15g, Folium Perillae(紫苏叶, Zi Su Ye)15g, Cortex Magnoliae	Hospital of chengdu university of Chinese medicine (Sichuan central hospital)	Treatment	Huoxiang Zhengqi Powder and Sanren Decoction	13/8	7/5		53.85/62.50	Huoxiang Zhengqi Powder: 1, Sanren Decoction: 0	Huoxiang Zhengqi Powder: 3/4, Sanren Decoction: 1	96COVID19 3th

Officinalis(厚朴,
Hou Pu)15g,
Rhizoma
Atractylodis
Macrocephalae(白
术, Bai Zhu)30g,
Rhizoma
Pinelliae(半夏, Ban
Xia)15g, (建曲, Jian
Qu)15g, Semen
Coicis(薏苡仁, Yi
Yi Ren)30g,
Poria(茯苓, Fu
Ling)30g, Fructus
Amomi
Rotundus(Fructus
Amomi
Rotundus(豆蔻,
Dou Kou), Dou
Kou)15g, Semen
Armeniacae
Amarum(杏仁,
Xing Ren)15g,
Crataegus
pinnatifida(焦山楂,
Jiao Shan Zha)30g,

97	Modified Chaihu Dayuan Decoction	Semen Dolichoris Album(白扁豆, Bai Bian Dou)30g, Rhizoma Phragmitis(芦根, Lu Gen)30g Radix Bupleuri(柴胡, Chai Hu), Radix Scutellariae(黄芩, Huang Qin), Fructus Aurantii(枳壳, Zhi Qiao), Fructus Tsaoko(草果, Cao Guo), Semen Armeniacae Amarum(杏仁, Xing Ren), Cortex Magnoliae Officinalis(厚朴, Hou Pu), Semen Arecae(槟榔, Bin Lang), Herba Agastaches(藿香, Huo Xiang), Talcum(滑石, Hua Shi), Bulbus	The prevention and control of COVID-19 in Tianjin	Treatment/ Critical illness	Chaihu Dayuan Decoction	10	6	60.00	1	3/6

98	Maxing Shigan Decoction and Modified Dayuan Decoction	Fritillariae Thunbergii(浙贝母, Zhe Bei Mu), Poria(茯苓, Fu Ling), Radix Paeoniae Rubra(赤芍, Chi Shao), Semen Coicis(生薏苡仁, Sheng Yi Yi Ren), Semen Raphani(莱菔子, Lai Fu Zi), Lumbricus(地龙, Di Long), Radix Glycyrrhizae(甘草, Gan Cao) Herba Ephedrae(麻黄, Ma Huang)8g, Semen Armeniacae Amarum(杏仁, Xing Ren)10g, Gypsum Fibrosum(生石膏, Sheng Shi Gao)30g, Radix Glycyrrhizae(甘草,	The prevention and control of COVID-19 in Guangdong province (Trial version 1)	Treatment/ Preliminary stage	Maxing Shigan Decoction and Dayuan Decoction	4/7	4/7	100.00/100.00	Maxing Shigan Decoction: 1, Dayuan Decoction: 1	Maxing Shigan Decoction: 1, Dayuan Decoction: 1	98GD

	Gan Cao)10g, Semen Arecae(槟榔, Bin Lang)10g, Cortex Magnoliae Officinalis(厚朴, Hou Pu)10g, Fructus Tsaoko(草果, Cao Guo)10g, Rhizoma Anemarrhenae(知母, Zhi Fu)10g, Radix Paeoniae Alba(白芍, Bai Shao)10g, Radix Scutellariae(黄芩, Huang Qin)15g, Radix Glycyrrhizae(甘草, Gan Cao)5g									
99	Radix Scutellariae(黄芩, Huang Qin)15g, Rhizoma Anemarrhenae(知母, Zhi Fu)10g, Herba Ephedrae(麻	The prevention and control of COVID-19 in Beijin(Version 2)	Treatment/ Ordinary type	Maxing Shigan Decoction and Maxing Yigan Decoction and Sanren	4/4/8	3/3/3	75.00/75.00/37.50	Maxing Shigan Decoction: 1/2, Maxing Yigan Decoction:	Maxing Shigan Decoction: 1, Maxing Yigan Decoction	99BJ

	黄, Ma Huang)10g, Semen Armeniacae Amarum(杏仁, Xing Ren)9g, Fructus Amomi Rotundus(豆蔻, Dou Kou)6g, Semen Coicis(薏苡仁, Yi Yi Ren)30g, Cortex Mori(桑白皮, Sang Bai Pi)15g, Rhizoma Atractylodis(苍术, Cang Shu)10g, Radix Astragali seu Hedysari(生黄芪, Sheng Huang Qi)10g, Semen Lepidii(葶苈子, Ting Li Zi)15g			Decoction				1, Sanren Decoction: 0	: 1, Sanren Decoction : 1	
100	Herba Ephedrae(麻黄, Ma Huang)4g, Gypsum Fibrosum(生石膏, Sheng Shi Gao)20g, Rhizoma	The prevention and control of COVID-19 in Beijin(Version 2)	Treatment for children	Maxing Shigan Decoction and Maxing Yigan Decoction	4/4/4	3/3/4	75.00/75.00/100.00	Maxing Shigan Decoction: 1, Maxing Yigan	Maxing Shigan Decoction : 1, Maxing Yigan	100BJ

		Anemarrhenae(知母, hi Fu)9g, Semen Armeniacae Amarum(杏仁, Xing Ren)10g, Semen Coicis(薏苡仁, Yi Yi Ren)10g, Fructus Trichosanthis(瓜蒌, Gua Lou)10g, Radix et Rhizoma Rhei(大黄, Da Huang)5g, Cortex Mori(桑白皮, Sang Bai Pi)10g, Semen Lepidii(葶苈子, Ting Li Zi)6g, Cornu Bubali(水牛角, Shui Niu Jiao)10g, Lumbricus(地龙, Di Long)10g, Radix Ginseng(人参, Ren Shen)6g			and Xuanbai Chengqi Decoction				Decoction: 1, Xuanbai Chengqi Decoction: 1	Decoction: 1, Xuanbai Chengqi Decoction: 1	
101	Xuanbai Chengqi	Semen Armeniacae Amarum(杏仁,	1, Sichuan provincial	Treatment Acute stage	Xuanbai Chengqi	4/4	4/4	100.00/100.00	Xuanbai Chengqi	Xuanbai Chengqi	101SC/YN

	Decoction,Huanglian Jiedu Decoction ,Jiedu Huoxue Decoction	Xing Ren)15g, Gypsum Fibrosum(生石膏, Sheng Shi Gao)20-30g, Fructus Trichosanthis(瓜蒌, Gua Lou)皮 15g, Radix et Rhizoma Rhei(大黄, Da Huang)5g, Herba Ephedrae(麻黄, Ma Huang)10g, Semen Lepidii(葶苈子,Ting Li Zi)15-20g, Semen Persicae(桃仁, Tao Ren)10g, Radix Paeoniae Rubra(赤芍, Chi Shao)15g, Radix Glycyrrhizae(生甘草, Sheng Gan Cao)5-10g	administration of TCM/The prevention and control of COVID-19 in Sichuan province (Trial version 1) 2, The prevention and control of COVID-19 in Yunnan province(Trial)		Decoction and Maxing Shigan Decoction				Decoction: 1, Maxing Shigan Decoction: 1	Decoction : 1, Maxing Shigan Decoction : 1	
102		Radix Astragali seu Hedysari(生黄芪,	Chinese medicine prevention plan of	Prevention for children	Yupingfeng Powder	3	3	100.00	1	1	102SX2

	Sheng Huang Qi)9g, Rhizoma Atractylodis Macrocephalae(炒白术, Chao Bai Zhu)6g, Radix Saposhnikoviae(防风, Fang Feng)3g, Radix Scrophulariae(玄参, Xuan She)6g, Bulbus Lilii(百合, Bai He)9g, Radix Platycodonis(桔梗, Jie Geng)6g, Cortex Magnoliae Officinalis(厚朴, Hou Pu)6g, Radix Glycyrrhizae(生甘草, Sheng Gan Cao)6g	COVID-19 in Shanxi province								
103	Herba Ephedrae(生麻黄, Sheng Ma Huang)6g, Gypsum Fibrosum(生石膏, Sheng Shi Gao)15g,	Diagnosis and treatment of COVID-19(Trial version 6)	Treatment/ Mild Symptoms	Maxing Shigan Decoction and Huoxiang	4/13	4/6	100.00/46.15	Maxing Shigan Decoction: 1, Huoxiang	Maxing Shigan Decoction : 1, Huoxiang	103 6

Semen Armeniacae Amarum(杏仁, Xing Ren)9g, Rhizoma et Radix Notopterygii(羌活,Qiang Huo)15g, Semen Lepidii(葶苈子,Ting Li Zi)15g, RhizomaCyrtomiiFortunei(贯众, Guan Zhong)9g, Lumbricus(地龙, Di Long)15g, Radix Cynanchi Paniculati(徐长卿, Xu Chang Qing)15g, Herba Agastaches(藿香, Huo Xiang)15g, Herba Eupatorii(佩兰, Pei Lan)9g, Rhizoma Atractylodis(苍术, Cang Shu)15g, Poria(茯苓, Fu Ling)45g, 生	Zhengqi Powder	Zhengqi Powder: 1	Zhengqi Powder: 2/4

104	Modified Dayuan Decoction	Rhizoma Atractylodis Macrocephalae(白术, Bai Zhu)30g, (焦三仙, Jiao San Xian)各 9g, Cortex Magnoliae Officinalis(厚朴, Hou Pu)15g, Semen Arecae(槟榔, Bin Lang)9g, Fructus Tsaoko(草果, Cao Guo)9g, Rhizoma Zingiberis Recens(生姜, Sheng Jiang)15g Semen Arecae(槟榔, Bin Lang)10g, Fructus Tsaoko(草果, Cao Guo)10g, Cortex Magnoliae Officinalis(厚朴, Hou Pu)10g, Rhizoma Anemarrhenae(知母, Zhi Fu)10g,	Diagnosis and treatment of COVID-19(Trial version 6)	Treatment/ Mild Symptoms	Chaihu Dayuan Decoction	10	5	50.00	1	3/6

		Radix Scutellariae(黄芩, Huang Qin)10g, Radix Bupleuri(柴胡, Chai Hu)10g, Radix Paeoniae Rubra(赤芍, Chi Shao)10g, Fructus Forsythiae(连翘, Lian Qiao)15g, Herba Artemisiae Annuae(青蒿, Qing Hao)10g(After decoction), Rhizoma Atractylodis(苍术, Cang Shu)10g, Folium Isatidis(大青叶, Da Qing Ye)10g, Radix Glycyrrhizae(生甘草, Sheng Gan Cao)5g									
105	Maxing Shigan Decoction,Modified Maxing	Herba Ephedrae(生麻黄, Sheng Ma Huang)6g, Semen	Diagnosis and treatment of COVID-19(Trial	Treatment/ Ordinary type	Maxing Shigan Decoction	4/4	4/4	100.00/100.00	Maxing Shigan Decoction:	Maxing Shigan Decoction	105 6

Yigan Decoction	Armeniacae Amarum(杏仁, Xing Ren)15g, Gypsum Fibrosum(生石膏, Sheng Shi Gao)30g, Semen Coicis(生薏苡仁,Sheng Yi Yi Ren)30g, Rhizoma Atractylodis(苍术, Cang Shu)10g, Herba Agastaches(藿香, Huo Xiang)15g, Herba Artemisiae Annuae(青蒿, Qing Hao)12g, Rhizoma Polygoni Cuspidati(Rhizoma Polygoni Cuspidati(虎杖, Hu Zhang),Hu Zhang)20g, Herba Herba Verbenae(马鞭草, Ma Bian Cao)30g, Rhizoma	version 6)	and Maxing Yigan Decoction	1, Maxing Yigan Decoction: 1	: 1, Maxing Yigan Decoction : 1

106	Maxing Shigan Decoction and Modified Huoxiang Zhengqi Powder, Huashi Baidu formula	Phragmitis(芦根, Lu Gen)30g, Semen Lepidii(葶苈子, Ting Li Zi)15g, Citri Grandis Exocarpium(化橘红, Huajuhong)15g, Radix Glycyrrhizae(生甘草, Sheng Gan Cao)10g Herba Ephedrae(生麻黄, Sheng Ma Huang)6g, Gypsum Fibrosum(生石膏, Sheng Shi Gao)15g, Semen Armeniacae Amarum(杏仁, Xing Ren)9g, Radix Glycyrrhizae(甘草, Gan Cao)3g, Herba Agastaches(藿香, Huo Xiang)10g(After decoction), Cortex Magnoliae	1, Diagnosis and treatment of COVID-19(Trial version 6)<br>2, Diagnosis and treatment of COVID-19(Trial version 7)	Treatment/ Critical illness	Maxing Shigan Decoction and Huopu Xialing Decoction	4/11	4/4		100.00/36.36	Maxing Shigan Decoction: 1, Huopu Xialing Decoction: 1	Maxing Shigan Decoction: 1, Huopu Xialing Decoction: 1/3	106hsbd

		Officinalis(厚朴, Hou Pu)10g, Rhizoma Atractylodis(苍术, Cang Shu)15g, Fructus Tsaoko(草果, Cao Guo)10g, Rhizoma Pinelliae(半夏, Ban Xia)9g, Poria(茯苓, Fu Ling)15g, Radix et Rhizoma Rhei(生大黄, Sheng Da Huang)5g(After decoction), Radix Astragali seu Hedysari(生黄芪, Sheng Huang Qi)10g, Semen Lepidii(葶苈子,Ting Li Zi)10g, Radix Paeoniae Rubra(赤芍, Chi Shao)10g									
107	Yinhuang Qingfei	Semen Lepidii(葶苈子, Ting Li Zi),	The prevention and control of	Recommended use of	Maxing Shigan	4	4	100.00	1	1	107HuN

Capsule	Herba Ephedrae(麻黄, Ma Huang), Semen Armeniacae Amarum(杏仁, Xing Ren), Bulbus Fritillariae Thunbergii(Bulbus Fritillariae Thunbergii(浙贝母, Zhe Bei Mu),Zhe Bei Mu), Folium Eriobotryae(枇杷叶, Pi Pa Ye), Folium Isatidis(大青叶, Da Qing Ye), Rhizoma Acori Tatarinowii(石菖蒲, Shi Chang Pu), Dioscorea nipponica Makino(穿山龙, Chuan Shan Long), Artemisia rupestris L.(一枝蒿, Yi Zhihao), Folium Ginkgo(银杏叶,	COVID-19 in Hunan province (Trial version 3)	proprietary Chinese medicines	Decoction

108	Qingre Jiedu Granules	Yin Xingye), Fructus Schisandrae Chinensis(五味子, Wu Wei Zi), Fructus Aurantii Immaturus(枳实, Zhi Shi), Gypsum Fibrosum(生石膏, Sheng Shi Gao), Radix Glycyrrhizae(甘草, Gan Cao) Flos Lonicerae(金银花, Jin Yin Hua), Fructus Forsythiae(连翘, Lian Qiao), Cornu Bubali(水牛角, Shui Niu Jiao), Folium Isatidis(大青叶, Da Qing Ye), Gypsum Fibrosum(石膏, Shi Gao), Rhizoma Coptidis(黄连, Huang Lian), Radix	The prevention and control of COVID-19 in Hunan province (Trial version 3)	Recommended use of proprietary Chinese medicines	Yinqiao Powder	10	2	20.00	1	0	108HuN

109	Mahuang Guigan Decoction	Rehmanniae Recens(生地, Sheng Di), Rhizoma Anemarrhenae(知母, Zhi Fu), Radix Scrophulariae(玄参, Xuan She), Excipients are dextrin,sucrose Herba Ephedrae(麻黄, Ma Huang), Radix Astragali seu Hedysari(黄芪, Huang Qi), Ramulus Cinnamomi(桂枝, Gui Zhi), Radix Glycyrrhizae(甘草, Gan Cao), Folium Artemisiae Argyi(艾叶,Ai Ye), Radix Paeoniae Alba(白芍, Bai Shao), Ajuga decumbens thunb (散血草, San Xuecao), Rhizoma	Professor Ding's empirical formula	Mahuang Decoction and Huangqi Guizhi Wuwu Decoction	4/5	3/5	75.00/100.00	Mahuang Decoction: 1, Huangqi Guizhi Wuwu Decoction: 1	Mahuang Decoction: 1, Huangqi Guizhi Wuwu Decoction: 1

110		Zingiberis(干姜, Gan Jiang), Fructus Jujubae(大枣, Da Zao)<br>Radix Astragali seu Hedysari(黄芪, Huang Qi) 10g,Rhizoma Atractylodis Macrocephalae(白术, Bai Zhu)10g,Radix Saposhnikoviae(防风, Fang Feng)10g, Flos Lonicerae(金银花, Jin Yin Hua)10g,Fructus Forsythiae(连翘, Lian Qiao)10g,Rhizoma Phragmitis(芦根, Lu Gen)10g	Shanxi Provincial Hospital of TCM		Yupingfeng Powder and Yinqiao Powder	3/10	3/3	100.00/30.00	Yupingfeng Powder: 1, Yinqiao Powder: 1	Yupingfeng Powder: 1, Yinqiao Powder: 0	110sxxf
111	Modified Huoxiang Zhengqi Powder	Rhizoma Atractylodis(苍术, Cang Shu)15g, Pericarpium Citri	Diagnosis and treatment of COVID-19(Trial version 4)	Treatment/ Ordinary type	Huoxiang Zhengqi Powder	13	5	38.50	1	1/4	

		Reticulatae(陈皮, Chen Pi)10g, Cortex Magnoliae Officinalis(厚朴, Hou Pu)10g, Herba Agastaches(藿香, Huo Xiang)10g, Fructus Tsaoko(草果, Cao Guo)6g, Herba Ephedrae(生麻黄, Sheng Ma Huang)6g, Rhizoma et Radix Notopterygii(羌活, Qiang Huo)10g, Rhizoma Zingiberis Recens(生姜, Sheng Jiang)10g, Semen Arecae(槟榔, Bin Lang)10g	Diagnosis and treatment of COVID-19(Trial version 6)								
112	Qingfeitongluo Prescription	Herba Ephedrae(麻黄, Ma Huang)9g Semen Armeniacae Amarum(杏仁, Xing Ren)9g Gypsum	Gansu Provincial Health Committee	Treatment/ Critical illness	Maxing Shigan Decoction and Maxing Yigan Decoction	4/4	3/3	75.00/75.00	Maxing Shigan Decoction: 1, Maxing Yigan	Maxing Shigan Decoction : 1, Maxing Yigan	112GS

		Fibrosum(生石膏, Sheng Shi Gao)20g(First decoction)Rhizoma Arisaematis Cum Bile(胆南星, Dan Nan Xing)6g Semen Lepidii(葶苈子, Ting Li Zi)15g Semen Persicae(桃仁, Tao Ren)9g Radix Paeoniae Rubra(赤芍, Chi Shao)15g Rhizoma Belamcandae(射干, She Gan)9g Semen Coicis(薏苡仁, Yi Yi Ren)15g Hirudo(水蛭, Shui Zhi)3g							Decoction: 1	Decoction: 1	
113	Yinqiao Powder and Modified Huopu Xialing Decoction	Flos Lonicerae(金银花, Jin Yin Hua)30g Fructus Forsythiae(连翘, Lian Qiao)30g Herba	Technical guidelines for TCM prevention and control of COVID-19 in Sichuan province	Clinical Treatment period	Yinqiao Powder,Huopu Xialing Decoction	10/11	7/7	70.00/63.64	Yinqiao Powder: 1, Huopu Xialing Decoction: 1	Yinqiao Powder: 3/4, Huopu Xialing Decoction	113SC

Schizonepetae(荆芥, Jing Jie)15g Fructus Arctii(牛蒡子, Niu Bang Zi)15g Herba Menthae Heplocalycis(薄荷, Bo He)15g Radix Platycodonis(桔梗, Jie Geng)30g Semen Armeniacae Amarum(杏仁, Xing Ren)15g Herba Agastaches(藿香, Huo Xiang)15g Cortex Magnoliae Officinalis(厚朴, Hou Pu) 15g Poria(茯苓, Fu Ling) 30g Rhizoma Pinelliae(半夏, Ban Xia)15g Fructus Amomi Rotundus(Fructus Amomi

(Revised edition)

: 1

114	Sangju Decoction and Modified Yinqiao Powder	Rotundus(豆蔻, Dou Kou), Dou Kou)15g Semen Coicis(薏苡仁, Yi Yi Ren)30g Semen Dolichoris Album(白扁豆, Bai Bian Dou)30g Crataegus pinnatifida(焦山楂, Jiao Shan Zha)30g (建曲, Jian Qu)15g Rhizoma Phragmitis(芦根, Lu Gen)30g Folium Mori(桑叶, Sang Ye)10g Flos Chrysanthemi(菊花, Ju Hua)10g Semen Armeniacae Amarum(杏仁, Xing Ren)10g Flos Lonicerae(金银花, Jin Yin Hua)15gFructus Forsythiae(连翘,	Technical guidelines for TCM prevention and control of COVID-19 in Sichuan province (Revised edition)	Clinical Treatment period	Sangju Decoction,Yinqiao Powder	8/10	3/4	37.50/40.00	Sangju Decoction: 1, Yinqiao Powder: 1	Sangju Decoction: 1/3, Yinqiao Powder: 1/4	114SC

115	Maxing Yigan Decoction, Modified Dayuan Decoction	Lian Qiao)10g Fructus Arctii(牛蒡子, Niu Bang Zi)10g Radix Glycyrrhizae(甘草, Gan Cao)5g Herba Ephedrae(麻黄, Ma Huang)10g Semen Armeniacae Amarum(杏仁, Xing Ren)15g Semen Coicis(薏苡仁, Yi Yi Ren)20-30g Fructus Tsaoko(草果, Cao Guo)10-20g Semen Arecae(槟榔, Bin Lang)10-15g Fructus Forsythiae(连翘, Lian Qiao)10-30g Rhizoma Atractylodis(苍术, Cang Shu)10-15g Cortex Magnoliae Officinalis(厚朴,	Technical guidelines for TCM prevention and control of COVID-19 in Sichuan province (Revised edition)	Clinical Treatment period	Maxing Yigan Decoction, Dayuan Decoction	4/7	4/5	100.00/71.43	Maxing Yigan Decoction: 1, Dayuan Decoction: 1	Maxing Yigan Decoction: 1, Dayuan Decoction: 1	115SC

116	Zhuye Shigao Decoction and Modified Sijunzi Decoction	Hou Pu)10-15g Radix Scutellariae(黄芩, Huang Qin)15g Radix Glycyrrhizae(生甘草, Sheng Gan Cao)5-10g。Lophatherum gracile(淡竹叶, Dan Zhu Ye)15g, Gypsum Fibrosum(石膏, Shi Gao)15-20g, Radix Pseudostellariae(太子参, Tai Zi Shen)20-30g, Radix Ophiopogonis(麦冬, Mai Dong)10-15g, Rhizoma Pinelliae(半夏, Ban Xia)10g, Rhizoma Atractylodis Macrocephalae(白术, Bai Zhu)15-20g,	The Prescription for the intervention of TCM intervention of COVID-19 in sichuan province(Trial version 1)	Treatment/ Convalescence	Zhuye Shigao Decoction and Sijunzi Decoction	7/4	6/4	85.71/100.00	Zhuye Shigao Decoction: 1, Sijunzi Decoction: 1	Zhuye Shigao Decoction: 1, Sijunzi Decoction: 1

117	Erchen Decoction and Modified Wangshiqingshuyiqi Decoction	Poria(茯苓, Fu Ling)15-20g, Radix Glycyrrhizae(甘草, Gan Cao)5-10g Radix Panacis Quinquefolii(西洋参, Xi Yang Shen)20g, Herba Dendrobii(石斛, Shi Hu)10g, Radix Ophiopogonis(麦冬, Mai Dong)10g, Rhizoma Anemarrhenae(知母, Zhi Fu)10g, Lophatherum gracile(淡竹叶, Dan Zhu Ye)10g, Rhizoma Coptidis(黄连, Huang Lian)3g, Radix Glycyrrhizae(甘草, Gan Cao)6g, Poria(茯苓, Fu Ling)15g, 法	The prevention and control of COVID-19 in Guangdong province (Trial version 1)	Treatment/ Convalescence	Erchen Decoction and Wangshiqingshuyiqi Decoction	4/10	4/7	100.00/70.00	Erchen Decoction: 1, Wangshiqingshuyiqi Decoction: 1/2	Erchen Decoction: 1, Wangshiqingshuyiqi Decoction: 2/3

118	Rhizoma Pinelliae(半夏, Ban Xia)10g, Citri Exocarpium Rubrum(橘红, Ju Hong)10g, Pericarpium Citri Reticulatae(陈皮, Chen Pi)10g, Fructus Hordei Germinatus(炒麦芽, Chao Mai Ya)30g Adenophora stricta Miq.(沙参, Sha Shen)15g, Radix Ophiopogonis(麦冬, Mai Dong)15g, Radix Astragali seu Hedysari(生黄芪, Sheng Huang Qi)15g, Massa Medicata Fermentata(神曲, Shen Qu)20g, Radix Paeoniae Rubra(赤	The prevention and control of COVID-19 in Beijin(Version 2)	Treatment/ Convalescence	Shashen Maidong Decoction and Xiebai Powder	7/4	2/4	28.57/100.00	Shashen Maidong Decoction: 1, Xiebai Powder: 1	Shashen Maidong Decoction: 1, Xiebai Powder: 1

119	芍, Chi Shao)15g, Cortex Mori(桑白皮, Sang Bai Pi)15g, Cortex Lycii(地骨皮, Di Gu Pi)15g, Fructus Aurantii(枳壳, Zhi Qiao)10g, Herba Artemisiae Annuae(青蒿, Qing Hao)15g, Radix Rehmanniae Recens(生地, Shen Di)15g Radix Pseudostellariae(太子参, Tai Zi Shen) 15g(or Radix Panacis Quinquefolii(西洋参, Xi Yang Shen) 15g), Radix Ophiopogonis(麦冬, Mai Dong) 18g, Fructus Schisandrae Chinensis(五味子,	The prevention and control of COVID-19 in Shanxi province (Trial)	Convalescence	Shengjiang Powder and Shenling Baizhu Powder	3/10	3/5	100.00/50.00	Shengmai Decoction: 1, Shenling Baizhu Powder2/3	Shengmai Decoction: 1, Shenling Baizhu Powder1/4	119SX1

120	Wu Wei Zi) 9g, Rhizoma Pinelliae(半夏, Ban Xia) 9g, Pericarpium Citri Reticulatae(陈皮, Chen Pi) 9g, Poria(茯苓, Fu Ling) 15g, Fructus Amomi Villosi(砂仁, Sha Ren) 6g, Lablab purpureus (Linn.) Sweet(扁豆, ai Pian Dou) 15g Rhizoma Pinelliae(半夏, Ban Xia)9g, Pericarpium Citri Reticulatae(陈皮, Chen Pi)10g, Radix Codonopsis(党参, Dang Shen)15g, Radix Astragali seu Hedysari(黄芪, Huang Qi)30g, Poria(茯苓, Fu	Diagnosis and treatment of COVID-19(Trial version 4)	Treatment/ Convalescence	Erchen Decoction	6	3	50.00	1	1

		Ling)15g, Herba Agastaches(藿香, Huo Xiang)10g, Fructus Amomi Villosi(砂仁, Sha Ren)6g(After decoction)								
121	Huangqi Liujunzi Decoction	Radix Astragali seu Hedysari(黄芪, Huang Qi), Radix Codonopsis(党参, Dang Shen), Poria(茯苓, Fu Ling), Rhizoma Atractylodis Macrocephalae(白术, Bai Zhu), 法 Rhizoma Pinelliae(半夏, Ban Xia), Pericarpium Citri Reticulatae(陈皮, Chen Pi), Radix Glycyrrhizae(甘草, Gan Cao)	The prevention and control of COVID-19 in Hunan province (Trial version 3)		Huangqi Liujunzi Decoction	8	7	87.50	1/2	2/3
122	Shashen Maidong	Adenophora stricta Miq.(沙参, Sha	The prevention and control of	Treatment/ Convalesce	Shashen Maidong	7	7	100.00	1	1

	Decoction	Shen), Radix Ophiopogonis(麦冬, Mai Dong), Lablab purpureus (Linn.) Sweet(扁豆, Bai Pian Dou), Folium Mori(桑叶, Sang Ye), Rhizoma Polygonati Odorati(玉竹,Yu Zhu), Radix Trichosanthis(天花粉, Tian Huafen), Radix Glycyrrhizae(甘草, Gan Cao)	COVID-19 in Hunan province (Trial version 3)	nce	Decoction					
123	Modified Erchen Decoction	Rhizoma Pinelliae(半夏, Ban Xia)9g, Pericarpium Citri Reticulatae(陈皮, Chen Pi)10g, Radix Codonopsis(党参, Dang Shen)15g, Radix Astragali seu Hedysari(黄芪,	Diagnosis and treatment of COVID-19 (Trial version 6)	Treatment/ Convalescence	Erchen Decoction	6	4	66.70	1	1

124	Modified Shenling Baizhu Powder	Huang Qi)30g, Poria(茯苓, Fu Ling)15g, Herba Agastaches(藿香, Huo Xiang)10g, Fructus Amomi Villosi(砂仁, Sha Ren)6g(After decoction), Radix Glycyrrhizae(甘草, Gan Cao)6g Radix Ginseng(生晒参, Sheng Shai Shen)10g, Rhizoma Atractylodis Macrocephalae(炒白术, Chao Bai Zhu)15g, Poria(茯苓, Fu Ling)15g, Semen Dolichoris Album(白扁豆, Bai Bian Dou)30g, Fructus Amomi Villosi(砂仁, Sha Ren)6g(After decoction), Semen	The prevention and control of COVID-19 in Guangdong province (Trial version 1)	Treatment/ Convalescence	Shenling Baizhu Powder	10	10	100.00	1	1

		Nelumbinis(莲子,Lian Zi)30g, Radix Glycyrrhizae(甘草, Gan Cao)6g, Radix Platycodonis(桔梗,Jie Geng)10g, Rhizoma Dioscoreae(山药, Shan Yao)15g, Semen Coicis(薏苡仁, Yi Yi Ren)30g, Fructus Hordei Germinatus(炒麦芽, Chao Mai Ya)30g, Massa Medicata Fermentata(神曲, Shen Qu)10g								
125	Modified Zhuye Shigao Decoction	Lophatherum gracile(淡竹叶, Dan Zhu Ye), Gypsum Fibrosum(生石膏, Sheng Shi Gao), Radix Pseudostellariae(太	The prevention and control of COVID-19 in Shanxi province (Trial version 1)	Treatment/ Convalescence	Zhuye Shigao Decoction	7	7	100.00	1	1

		子参, Tai Zi Shen), Radix Ophiopogonis(麦冬, Mai Dong), Rhizoma Pinelliae(半夏, Ban Xia), japonica Rice(粳米, Jing Mi), Rhizoma Phragmitis(芦根, Lu Gen), Fructus Setariae Germinatus(炒谷芽, Chao Gu Ya), Radix Glycyrrhizae(生甘草, Sheng Gan Cao)								
126	Pneumonia V (Shashen Maidong Decoction)	Adenophora stricta Miq.(沙参, Sha Shen)15g, Radix Ophiopogonis(麦冬, Mai Dong)15g, Fructus Schisandrae Chinensis(五味子, Wu Wei Zi)15g, Radix Ginseng(人	Agreement on the prevention and treatment of COVID-19 in hubei hospital of traditional Chinese medicine (1st edition)	Treatment/ Convalescence	Shashen Maidong Decoction and Shengjiang Powder	7/3	3/3	42.86/100.00	Shashen Maidong Decoction: 1, Shengmai Decoction: 1	Shashen Maidong Decoction : 1, Shengmai Decoction : 1	126pneumonia V

参, Ren Shen)12g,
Semen Raphani(莱
菔子, Lai Fu
Zi)15g , Retinervus
Luffae Fructus(丝瓜
络, Si Gua Luo)15g,
Retinervus Citri
Furctus(橘络, Ju
Luo)15, Fructus
Perillae(苏子, Su
Zi)12g, Bulbus
Fritillariae
Thunbergii(Bulbus
Fritillariae
Thunbergii(浙贝母,
Zhe Bei Mu)母,Zhe
Bei Mu12g,
Semen Armeniacae
Amarum(杏仁,
Xing Ren)12g,
Radix
Scutellariae(黄芩,
Huang Qin)15g,
Radix
Glycyrrhizae(生甘
草, Sheng Gan

127	Cao)10g Radix Pseudostellariae(太子参, Tai Zi Shen), Radix Ophiopogonis(麦冬, Mai Dong), Fructus Schisandrae Chinensis(五味子, Wu Wei Zi), Radix Astragali seu Hedysari(生黄芪, Sheng Huang Qi), Rhizoma Atractylodis Macrocephalae(炒白术, Chao Bai Zhu), Pericarpium Citri Reticulatae(陈皮, Chen Pi), Radix Angelicae Sinensis(当归, Dang Gui), Radix Glehniae(北沙参, Bei Sha Shen), Semen Armeniacae	The prevention and control of COVID-19 in Shanxi province (Trial version 1)	Shengjiang Powder and Buzhongyiqi Decoction	3/8	3/6	100.00/75.00	Shengmai Decoction: 1, Buzhongyiqi Decoction: 1	Shengmai Decoction: 1, Buzhongyiqi Decoction: 1	127SX2

128	Shengjiang Powder and Modified Zhuye Shigao Decoction	Amarum(杏仁, Xing Ren), Folium Eriobotryae(枇杷叶, Pi Pa Ye), Bulbus Lilii(百合, Bai He), Radix Glycyrrhizae(甘草, Gan Cao) Adenophprae Ae Radix(南沙参, Nanshashen),Radix Glehniae(北沙参, Bei Sha Shen)各10g, Radix Ophiopogonis(麦冬, Mai Dong)15g, Radix Panacis Quinquefolii(西洋参, Xi Yang Shen)6g, Fructus Schisandrae Chinensis(五味子, Wu Wei Zi)6g, Gypsum Fibrosum(生石膏, Sheng Shi Gao)15g,	Diagnosis and treatment of COVID-19 (Trial version 6)	Convalescence	Shengjiang Powder and Zhuye Shigao Decoction	3/7	3/5	100.00/71.43	Shengmai Decoction: 1, Zhuye Shigao Decoction: 1	Shengmai Decoction: 1, Zhuye Shigao Decoction: 1	128 6

129	Lophatherum gracile(淡竹叶, Dan Zhu Ye)10g, Folium Mori(桑叶, Sang Ye)10g, Rhizoma Phragmitis(芦根, Lu Gen)15g, Radix Salviae Miltiorrhizae(丹参, Dan Shen)15g, Radix Glycyrrhizae(生甘草, Sheng Gan Cao)6g<br>Radix Codonopsis(党参, Dang Shen) 15g, Radix Astragali seu Hedysari(黄芪, Huang Qi) 30g, Poria(茯苓, Fu Ling) 15g, Rhizoma Pinelliae(半夏, Ban Xia)9g, Pericarpium Citri Reticulatae(陈皮, Chen Pi) 9g,	The prevention and control of COVID-19 in Shanxi province (Trial)	Convalescence	Erchen Decoction	6	3	50.00	1	1

130	Yifei Jianpi Recipe	Herba Agastaches(藿香, Huo Xiang) 9g, Fructus Amomi Villosi(砂仁, Sha Ren) 6g Radix Astragali seu Hedysari(黄芪, Huang Qi)15g Radix Codonopsis(党参, Dang Shen)6g Pericarpium Citri Reticulatae(陈皮, Chen Pi)9g Radix Angelicae Sinensis(当归, Dang Gui)9g Radix Bupleuri(柴胡, Chai Hu)6g Radix Platycodonis(桔梗, Jie Geng)3g 麸 Rhizoma Atractylodis Macrocephalae(炒白术, Chao Bai	Gansu Provincial Health Committee	Recovery	Buzhongyiqi Decoction	8	7	87.50	1	1/2

131	Suggested Prescription of professor DuJiang(Miao medicine expert)	Zhu)15g 炒 Radix Paeoniae Alba(白芍, Bai Shao)9g Fructus Amomi Villosi(砂仁, Sha Ren)3g 生 Fructus Hordei Germinatus(麦芽, Mai Ya15g 炙 Radix Glycyrrhizae(甘草, Gan Cao)6g Serissa serissoides (DC.)Druce (锐过买 (白马骨), Bai Ma Gu)15g, (窝嘎乃 (墨斗菜), Wo Ga Nai)10g, Typhonium giganteum Engl.(加格略(独角莲), Du Jiao Lian)10g, Polygonum perfoliatum L. (加欧万囊 (蛇倒退), She Dao Tui)15g,	Guizhou provincial administration of TCM	Prevention

	Hyrtanandra hirta (Bl.) Miq.(加嘎旅(生扯拢), Sheng Che Long)12g, Polygala japonica Houtt. (锐草连（爪子金), Zhua Zi Jin)10g, Saxifraga stolonifera Curt.(窝比省(虎耳草), Hu Er Cao)12g, Centellaasiatica(L.) Urban(窝比赊溜(积雪草), Ji Xue Cao)12g, (锐阿都偏（岩虹豆), Yan Hong Dou)12g, Mahonia oiwakensis Hayata(都阿能（十大功劳), Shi Da Gong Lao)10g		
132	Serissa foetida Comm(白马骨, Bai Ma Gu)15g,Herba Echiptae(墨旱莲,	The prevention and control of COVID-19 in Beijin(Version 2)	Critical illness

	Mo Han Lian)10g,Dysosma pleiantha(独角莲, Du Jiao Lian)10g,Polygonum perfoliatum L.(蛇倒退, She Diao Tui)15g,Lycopodium serratum Thunb(生扯拢, Sheng Che Long)12g, (爪子金, Zhua Zi Jin)10g,saxifrage(虎耳草, Hu Er Cao)12g,Centalla asiatica(积雪草, Ji Xue Cao)12g, (岩虹豆, Yan Hong Dou)12g,Mahonia fortunei(十大功劳, Shi Da Gong Lao)10g		
133	RhizomaCyrtomiiFortunei(贯众, Guan Zhong)9-12g,	The prevention and control of COVID-19 in	Prevention

		Perilla frutescens (L.) Britt.(苏梗, Su Geng)12-15g, Semen Sojae Preparatum(淡豆豉, Dan Dou Chi)3-6g, Radix et Rhizoma Rhei(大黄, Da Huang)3-6g, Rhizoma Atractylodis(苍术, Cang Shu)6-9g	Gansu province (Trial)	
134	Professor Peng Yu's Child Prevention Advice	Radix Isatidis(板蓝根, Ban Lan Gen)20g, RhizomaCyrtomiiFortunei(贯众, Guan Zhong)15g, Herba Agastaches(藿香, Huo Xiang)10g, Herba Menthae Heplocalycis(薄荷, Bo He)10g, Flos Chrysanthemi(菊花, Ju Hua)15g, Herba Schizonepetae(荆	Guizhou provincial administration of TCM	Prevention

135	Prevention of early pregnancy (Within 3 months of conception)	芥, Jing Jie)10g, Radix Puerariae(葛根, Ge Gen)6g, Rhizoma Phragmitis(芦根, Lu Gen)10g, Radix Glycyrrhizae(甘草, Gan Cao)6g Rhizoma Atractylodis Macrocephalae(白术, Bai Zhu)9g, Radix Scutellariae(黄芩, Huang Qin)9g, Folium Perillae(紫苏叶, Zi Su Ye)3g, Flos Lonicerae(金银花, Jin Yin Hua)6g, Radix Ophiopogonis(麦冬, Mai Dong)6g	The prevention and control of Winter and Spring Flu 2021 and COVID-19 in Shandong province	Prevention
136		Flos Lonicerae(金银花, Jin Yin Hua)3g, Rhizoma Phragmitis(芦根,	The prevention and control of COVID-19 in Beijing (Version 2)	Prevention for children

137	Lu Gen)6g, Pericarpium Citri Reticulatae(陈皮, Chen Pi)2g Radix Astragali seu Hedysari(生黄芪, Sheng Huang Qi)9g, Radix Glehniae(北沙参, Bei Sha Shen)9g, Rhizoma Anemarrhenae(知母, Zhi Fu)9g, Trollius chinensis(金莲花, Jin Lian Hua)5g, Fructus Forsythiae(连翘, Lian Qiao)9g, Rhizoma Atractylodis(苍术, Cang Shu)9g, Radix Platycodonis(桔梗, Jie Geng)6g	The prevention and control of COVID-19 in Beijing (Version 2)	Prevention
138	Trollius chinensis(金莲花,	The prevention and control of	Prevention

	Jin Lian Hua)two flower,Radix Ophiopogonis(麦冬, Mai Dong)five grain,Canarium album (Lour.) Raeusch.(青果,Qing Gu)two grain(Smash),Flos Chrysanthemi(白菊花, Bai Ju Hua)two flower	COVID-19 in Beijing (Version 2)	
139	Herba Agastaches(藿香, Huo Xiang)15-30g, Herba Eupatorii(佩兰,Pei Lan)15-30g, Borneolum Syntheticum(冰片, Bing Pian)6-9g, Radix Angelicae Dahuricae(白芷, Bai Zhi)15-30g	The prevention and control of COVID-19 in Gansu province (Trial)	Prevention
140	Radix Ophiopogonis(麦冬, Mai Dong)3g,	The prevention and control of COVID-19 in	Prevention

		Folium Mori(桑叶, Sang Ye)3g, Flos Chrysanthemi(菊花, Ju Hua)3g, Pericarpium Citri Reticulatae(陈皮, Chen Pi)2g, Radix Astragali seu Hedysari(黄芪, Huang Qi)10g	Beijing (Version 2)
141	Baihe Jiufei Decoction		Frontline treatment effect feedback
142	Wuwei Baidu Decoction	Rhizoma et Radix Notopterygii(羌活, Qiang Huo), Heracleum hemsleyanum Diels(独活, Du Huo), Radix Peucedani(前胡, Qian Hu), Radix Bupleuri(柴胡, Chai Hu), Fructus Aurantii(枳壳, Zhi Qiao), Radix Platycodonis(桔梗,	Frontline treatment effect feedback

		Jie Geng), Radix Glycyrrhizae(甘草, Gan Cao), Radix Ginseng(人参, Ren Shen), Poria(茯苓, Fu Ling), Rhizoma Ligustici Chuanxiong(川芎, Chuan Qiong), Radix et Rhizoma Rhei(大黄, Da Huang), Rhizoma Atractylodis(苍术, Cang Shu)		
143	Zhenqifuzheng Granules	Radix Astragali seu Hedysari(黄芪, Huang Qi), Fructus Ligustri Lucidi(女贞子, Nv Zhen Zi)	Frontline treatment effect feedback	
144	Chaihu Daxiong Prescription (Strong Pneumonia I)		Development of Hubei Provincial Hospital of Traditional Chinese Medicine	Early stage/Progr essive stage
145	Cold antifebrile Prescription		Development of Hubei Provincial	

			Hospital of Traditional Chinese Medicine						
146	Xuanqing Hehua Prescription (Modified on the basis of "cold antifebrile Prescription")		Development of Hubei Provincial Hospital of Traditional Chinese Medicine						
147	Xuanfeibaidu Granule		National Health Commission of the People's Republic of China						
148	Xuanfeihuazhuo Powder	Herba Ephedrae(麻黄, Ma Huang)6 g, Fructus Forsythiae(连翘, Lian Qiao)15g, Radix Peucedani(前胡, Qian Hu)9g, Rhizoma Pinelliae(半夏, Ban Xia)12g, Rhizoma Atractylodis(苍术,	Gansu Provincial Health Committee	148	Xuanfeihuazhuo Powder	Herba Ephedrae(麻黄, Ma Huang)6 g, Fructus Forsythiae(连翘, Lian Qiao)15g, Radix Peucedani(前胡, Qian Hu)9g, Rhizoma Pinelliae(半夏, Ban Xia)12g, Rhizoma Atractylodis(苍术,	Gansu Provincial Health Committee	148	Xuanfeihuazhuo Powder

		Cang Shu)12g, Herba Agastaches(藿香, Huo Xiang)6g, Rhizoma et Radix Notopterygii(羌活, Qiang Huo)9g, Radix et Rhizoma Rhei(大黄, Da Huang)6g ,Pericarpi um Citri Reticulatae(陈皮, Chen Pi)6g, Radix Scutellariae(黄芩, Huang Qin)6g					Cang Shu)12g, Herba Agastaches(藿香, Huo Xiang)6g, Rhizoma et Radix Notopterygii(羌活, Qiang Huo)9g, Radix et Rhizoma Rhei(大黄, Da Huang)6g ,Pericarpi um Citri Reticulatae(陈皮, Chen Pi)6g, Radix Scutellariae(黄芩, Huang Qin)6g				
149	Cuitang Granule		Tibetan medicine Prescription	Prevention	149	Cuitang Granule		Tibetan medicine Prescriptio n	Prevention	149	Cuitang Granule
150	Cuitang Granule or Influenza Pill		Tibetan medicine Prescription		150	Cuitang Granule or Influenza Pill		Tibetan medicine Prescriptio n		150	Cuitang Granule or Influenza Pill
151	Renqing Mangjue Capsules	terminaliae billericae fructus(毛诃子, Mao He Zi),	Tibetan medicine Prescription		151	Renqing Mangjue Capsules	terminaliae billericae fructus(毛诃子, Mao He Zi),	Tibetan medicine Prescriptio		151	Renqing Mangjue Capsules

		Eugenia jambos L.(蒲桃, Pu Tao), Flos Carthami(红花, Hong Hua), Calculus Bovis(牛黄, Niu Huang), Moschus(Moschus(麝香, She Xiang),She Xiang), Cinnabaris(朱砂, Zhu Sha)					Eugenia jambos L.(蒲桃, Pu Tao), Flos Carthami(红花, Hong Hua), Calculus Bovis(牛黄, Niu Huang), Moschus(Moschus(麝香, She Xiang),She Xiang), Cinnabaris(朱砂, Zhu Sha)	n			
152	Fuzheng Kegan Granule (Based on "Qixiangyiqijiedu Granule")		Neijiang city hospital of traditional Chinese medicine development,Produced by Sichuan tiande pharmaceutical co. LTD		152	Fuzheng Kegan Granule (Based on "Qixiangyiqijiedu Granule")		Neijiang city hospital of traditional Chinese medicine development,Produced by Sichuan tiande pharmaceutical co. LTD		152	Fuzheng Kegan Granule (Based on "Qixiangyiqijiedu Granule")
153	Qixiangyiqijied	Radix Astragali seu	Sichuan Academy	Prevention	153	Qixiangyiqi	Radix Astragali seu	Sichuan	Prevention	153	Qixiangyiqijie

	u Granule	Hedysari(黄芪, Huang Qi), Rhizoma Atractylodis Macrocephalae(白术, Bai Zhu), Herba Agastaches(藿香, Huo Xiang), Flos Chrysanthemi(菊花, Ju Hua), Herba Houttuyniae(鱼腥草, Yu Xing Cao), Rhizoma Cimicifugae(升麻, Sheng Ma), Rhizoma Atractylodis(苍术, Cang Shu), Radix Isatidis(板蓝根, Ban Lan Gen)	of TCM,Sichuan Second TCM Hospital			jiedu Granule	Hedysari(黄芪, Huang Qi), Rhizoma Atractylodis Macrocephalae(白术, Bai Zhu), Herba Agastaches(藿香, Huo Xiang), Flos Chrysanthemi(菊花, Ju Hua), Herba Houttuyniae(鱼腥草, Yu Xing Cao), Rhizoma Cimicifugae(升麻, Sheng Ma), Rhizoma Atractylodis(苍术, Cang Shu), Radix Isatidis(板蓝根, Ban Lan Gen)	Academy of TCM,Sichuan Second TCM Hospital			du Granule
154	Shenge Yifei Capsule	Radix Ginseng Rubra(红参, Hong Shen), Gekko gecko(蛤蚧, Ge Jie), Radix Notoginseng(三七,	Traditional Chinese Medicine Hospital of Southwest Medical University	Prevention	154	Shenge Yifei Capsule	Radix Ginseng Rubra(红参, Hong Shen), Gekko gecko(蛤蚧, Ge Jie), Radix Notoginseng(三七,	Traditional Chinese Medicine Hospital of Southwest Medical	Prevention	154	Shenge Yifei Capsule

		San Qi), Lumbricus(地龙, Di Long), Rhizoma Ligustici Chuanxiong(川芎, Chuan Qiong), Bulbus Fritillariae Cirrhosae(川贝母, Chuan Bei Mu), Radix Glycyrrhizae(甘草, Gan Cao)等					San Qi), Lumbricus(地龙, Di Long), Rhizoma Ligustici Chuanxiong(川芎, Chuan Qiong), Bulbus Fritillariae Cirrhosae(川贝母, Chuan Bei Mu), Radix Glycyrrhizae(甘草, Gan Cao)等	University			
155	Fufangyinchai Granule		Sichuan provincial hospital of traditional Chinese medicine, affiliated hospital of chengdu university of traditional Chinese medicine	Prevention	155	Fufangyinchai Granule		Sichuan provincial hospital of traditional Chinese medicine, affiliated hospital of chengdu university of traditional Chinese medicine	Prevention	155	Fufangyinchai Granule

156	Chuanshegan total flavone Capsule	Rhizoma Belamcandae(射干,She Gan), Flavonoids extracts	Technical guidelines for TCM prevention and control of COVID-19 in Sichuan province (Revised edition)	Prevention	156	Chuanshegan total flavone Capsule	Rhizoma Belamcandae(射干,She Gan), Flavonoids extracts	Technical guidelines for TCM prevention and control of COVID-19 in Sichuan province (Revised edition)	Prevention	156	Chuanshegan total flavone Capsule
157	Cold sliced mulberry ginger (Capsule)	Folium Mori(桑叶, Sang Ye), Fructus Forsythiae(连翘, Lian Qiao), Flos Chrysanthemi(菊花, Ju Hua), 苦 Semen Armeniacae Amarum(杏仁, Xing Ren), Folium Perillae(紫苏叶, Zi Su Ye), Rhizoma Zingiberis(干姜, Gan Jiang)	Technical guidelines for TCM prevention and control of COVID-19 in Sichuan province (Revised edition)	Prevention	157	Cold sliced mulberry ginger (Capsule)	Folium Mori(桑叶, Sang Ye), Fructus Forsythiae(连翘, Lian Qiao), Flos Chrysanthemi(菊花, Ju Hua), 苦 Semen Armeniacae Amarum(杏仁, Xing Ren), Folium Perillae(紫苏叶, Zi Su Ye), Rhizoma Zingiberis(干姜, Gan Jiang)	Technical guidelines for TCM prevention and control of COVID-19 in Sichuan province (Revised edition)	Prevention	157	Cold sliced mulberry ginger (Capsule)
158	Antivirus Granule	Radix Isatidis(板蓝根, Ban Lan Gen),	Technical guidelines for TCM	Prevention	158	Antivirus Granule	Radix Isatidis(板蓝根, Ban Lan Gen),	Technical guidelines	Prevention	158	Antivirus Granule

		Caulis Lonicerae(忍冬藤, Ren Dong Teng), Radix Sophorae Tonkinensis(山豆根, Shan Dou Gen), Rhizoma Belamcandae(射干, She Gan), Herba Houttuyniae(鱼腥草, Yu Xing Cao), Rhizoma Paridis(重楼, Chong Lou), RhizomaCyrtomiiFortunei(贯众, Guan Zhong), Radix Angelicae Dahuricae(白芷, Bai Zhi), Herba Artemisiae Annuae(青蒿, Qing Hao)	prevention and control of COVID-19 in Sichuan province (Revised edition)				Caulis Lonicerae(忍冬藤, Ren Dong Teng), Radix Sophorae Tonkinensis(山豆根, Shan Dou Gen), Rhizoma Belamcandae(射干, She Gan), Herba Houttuyniae(鱼腥草, Yu Xing Cao), Rhizoma Paridis(重楼, Chong Lou), RhizomaCyrtomiiFortunei(贯众, Guan Zhong), Radix Angelicae Dahuricae(白芷, Bai Zhi), Herba Artemisiae Annuae(青蒿, Qing Hao)	for TCM prevention and control of COVID-19 in Sichuan province (Revised edition)			
159	Bufei Decoction and Modified Sanzi Yangqin	Radix Astragali seu Hedysari(黄芪, Huang Qi)15g Radix	Technical guidelines for TCM prevention and control of	Convalescence	159	Bufei Decoction and Modified	Radix Astragali seu Hedysari(黄芪, Huang Qi)15g Radix	Technical guidelines for TCM prevention	Convalescence	159	Bufei Decoction and Modified Sanzi Yangqin

	Decoction	Pseudostellariae(太子参, Tai Zi Shen)15g Fructus Corni(山茱萸, Shan Zhu Yu)15g Fructus Aurantii Immaturus(枳实, Zhi Shi)10g Fructus Perillae(苏子, Su Zi)10g Semen sinapis(白芥子, Bai Jie Zi)10g Fructus Schisandrae Chinensis(五味子,Wu Wei Zi)10g Semen Persicae(桃仁, Tao Ren)10g Flos Carthami(红花, Hong Hua)10g	COVID-19 in Sichuan province (Revised edition)			Sanzi Yangqin Decoction	Pseudostellariae(太子参, Tai Zi Shen)15g Fructus Corni(山茱萸, Shan Zhu Yu)15g Fructus Aurantii Immaturus(枳实, Zhi Shi)10g Fructus Perillae(苏子, Su Zi)10g Semen sinapis(白芥子, Bai Jie Zi)10g Fructus Schisandrae Chinensis(五味子,Wu Wei Zi)10g Semen Persicae(桃仁, Tao Ren)10g Flos Carthami(红花, Hong Hua)10g	and control of COVID-19 in Sichuan province (Revised edition)			Decoction
160	Huanglian Jiedu Decoction	Flos Lonicerae(金银花, Jin Yin Hua), Radix Scutellariae(黄芩, Huang Qin), Fructus Forsythiae(连翘,	Chinese academy of sciences Shanghai institute of medicine and wuhan institute of virus joint research	Early stage/Progressive stage	160	Huanglian Jiedu Decoction	Flos Lonicerae(金银花, Jin Yin Hua), Radix Scutellariae(黄芩, Huang Qin), Fructus Forsythiae(连翘, Lian Qiao)	Chinese academy of sciences Shanghai institute of medicine and wuhan	Early stage/Progressive stage	160	Huanglian Jiedu Decoction

		Lian Qiao)						institute of virus joint research			
161	Prevention for childrenII(Special constitution)	Radix Pseudostellariae(太子参, Tai Zi Shen)10g, Herba Dendrobii(石斛, Shi Hu)6g, Radix Ophiopogonis(麦冬, Mai Dong)3g, Flos Lonicerae(金银花, Jin Yin Hua)3g, RhizomaCyrtomiiFortunei(贯众, Guan Zhong)5g, Fructus Jujubae(大枣, Da Zao)6g	The prevention and control of COVID-19 in Henan province (Trial)	Prevention	161	Prevention for childrenII(Special constitution)	Radix Pseudostellariae(太子参, Tai Zi Shen)10g, Herba Dendrobii(石斛, Shi Hu)6g, Radix Ophiopogonis(麦冬, Mai Dong)3g, Flos Lonicerae(金银花, Jin Yin Hua)3g, RhizomaCyrtomiiFortunei(贯众, Guan Zhong)5g, Fructus Jujubae(大枣, Da Zao)6g	The prevention and control of COVID-19 in Henan province (Trial)	Prevention	161	Prevention for childrenII(Special constitution)
162	Prophylaxis for middle and third trimester pregnancy (More than 3 months pregnant)	Rhizoma Atractylodis Macrocephalae(白术, Bai Zhu)9g, Radix Scutellariae(黄芩, Huang Qin)9g, Flos	The prevention and control of Winter and Spring Flu 2020 and COVID-19 in Shandong province	Prevention	162	Prophylaxis for middle and third trimester pregnancy (More than 3 months	Rhizoma Atractylodis Macrocephalae(白术, Bai Zhu)9g, Radix Scutellariae(黄芩, Huang Qin)9g, Flos	The prevention and control of Winter and Spring Flu 2020 and	Prevention	162	Prophylaxis for middle and third trimester pregnancy (More than 3 months pregnant)

		Lonicerae(金银花, Jin Yin Hua)6g, Radix Ophiopogonis(麦冬, Mai Dong)6g, Pericarpium Citri Reticulatae(陈皮, Chen Pi)6g				pregnant)	Lonicerae(金银花, Jin Yin Hua)6g, Radix Ophiopogonis(麦冬, Mai Dong)6g, Pericarpium Citri Reticulatae(陈皮, Chen Pi)6g	COVID-19 in Shandong province			
163	Formula Ifor General population adults	Radix Lithospermi(紫草, Zi Cao)10 克, Semen Phaseoli(赤小豆, Chi Xiao Dou)30 克, Vigna radiata (Linn.) Trollius chinensis(金莲花, Jin Lian Hua)30 克, Radix Glycyrrhizae(生甘草, Sheng Gan Cao)6 克	The prevention and control of COVID-19 in Henan province (Trial)	Prevention	163	Formula Ifor General population adults	Radix Lithospermi(紫草, Zi Cao)10 克, Semen Phaseoli(赤小豆, Chi Xiao Dou)30 克, Vigna radiata (Linn.) Trollius chinensis(金莲花, Jin Lian Hua)30 克, Radix Glycyrrhizae(生甘草, Sheng Gan Cao)6 克	The prevention and control of COVID-19 in Henan province (Trial)	Prevention	163	Formula Ifor General population adults
164	Prescription I for prevention of Special population	Herba Agastaches(藿香, Huo Xiang)3g, Pericarpium Citri	The prevention and control of COVID-19 in Henan province	Prevention	164	Prescription I for prevention of Special	Herba Agastaches(藿香, Huo Xiang)3g, Pericarpium Citri	The prevention and control of	Prevention	164	Prescription I for prevention of Special population

			(Trial)			population		COVID-19 in Henan province (Trial)			
165	Prescription III for prevention of Special constitution of children	Reticulatae(陈皮,Chen Pi)3g, Folium Mori(桑叶, Sang Ye)3g, Rhizoma Phragmitis(芦根, Lu Gen)2 克, Radix Platycodonis(桔梗, Jie Geng)3g, Radix Glycyrrhizae(甘草, Gan Cao)2g Herba Agastaches(藿香, Huo Xiang)6g, Semen Coicis(薏苡仁, Yi Yi Ren)12g, Rhizoma Atractylodis(苍术, Cang Shu)3g, Cortex Magnoliae Officinalis(厚朴, Hou Pu)3g, Flos Lonicerae(金银花, Jin Yin Hua)3g, RhizomaCyrtomiiFortunei(贯众, Guan	The prevention and control of COVID-19 in Henan province (Trial)	Prevention	165	Prescription III for prevention of Special constitution of children	Reticulatae(陈皮,Chen Pi)3g, Folium Mori(桑叶, Sang Ye)3g, Rhizoma Phragmitis(芦根, Lu Gen)2 克, Radix Platycodonis(桔梗, Jie Geng)3g, Radix Glycyrrhizae(甘草, Gan Cao)2g Herba Agastaches(藿香, Huo Xiang)6g, Semen Coicis(薏苡仁, Yi Yi Ren)12g, Rhizoma Atractylodis(苍术, Cang Shu)3g, Cortex Magnoliae Officinalis(厚朴, Hou Pu)3g, Flos Lonicerae(金银花, Jin Yin Hua)3g, RhizomaCyrtomiiFortunei(贯众, Guan	The prevention and control of COVID-19 in Henan province (Trial)	Prevention	165	Prescription III for prevention of Special constitution of children

166	Prevention for eucrasia children	Zhong)5g Gypsum Fibrosum(生石膏, Sheng Shi Gao)10 克, Vigna radiata (Linn.) Trollius chinensis(金莲花, Jin Lian Hua)12 克, Herba Taraxaci(蒲公英, Pu Gong Ying)6 克, Lophatherum gracile(淡竹叶, Dan Zhu Ye)6 克 Rhizoma Phragmitis(芦根, Lu Gen)3 克, RhizomaCyrtomiiFortunei(贯众, Guan Zhong)3 克	The prevention and control of COVID-19 in Henan province (Trial)	Prevention	166	Prevention for eucrasia children	Zhong)5g Gypsum Fibrosum(生石膏, Sheng Shi Gao)10 克, Vigna radiata (Linn.) Trollius chinensis(金莲花, Jin Lian Hua)12 克, Herba Taraxaci(蒲公英, Pu Gong Ying)6 克, Lophatherum gracile(淡竹叶, Dan Zhu Ye)6 克 Rhizoma Phragmitis(芦根, Lu Gen)3 克, RhizomaCyrtomiiFortunei(贯众, Guan Zhong)3 克	The prevention and control of COVID-19 in Henan province (Trial)	Prevention	166	Prevention for eucrasia children
167	Prescription IV for prevention of Special population	Radix Astragali seu Hedysari(生黄芪, Sheng Huang Qi)9 克, Rhizoma Belamcandae(射干, She Gan)5 克,	The prevention and control of COVID-19 in Henan province (Trial)	Prevention	167	Prescription IV for prevention of Special population	Radix Astragali seu Hedysari(生黄芪, Sheng Huang Qi)9 克, Rhizoma Belamcandae(射干, She Gan)5 克, Radix	The prevention and control of COVID-19 in Henan	Prevention	167	Prescription IV for prevention of Special population

		Radix Glehniae(北沙参, Bei Sha Shen)9 克, Flos Lonicerae(金银花, Jin Yin Hua)9 克, Rhizoma Atractylodis(苍术, Cang Shu)9 克, Herba Agastaches(藿香, Huo Xiang)6 克, RhizomaCyrtomiiFortunei(贯众, Guan Zhong)5 克					Glehniae(北沙参, Bei Sha Shen)9 克, Flos Lonicerae(金银花, Jin Yin Hua)9 克, Rhizoma Atractylodis(苍术, Cang Shu)9 克, Herba Agastaches(藿香, Huo Xiang)6 克, RhizomaCyrtomiiFortunei(贯众, Guan Zhong)5 克	province (Trial)			
168	Prevention of diabetes	Flos Lonicerae(金银花, Jin Yin Hua)12g, Adenophora stricta Miq.(沙参, Sha Shen)10g, Radix Ophiopogonis(麦冬, Mai Dong)15g, Rhizoma Phragmitis(芦根, Lu Gen)15g, Semen Coicis(生薏苡仁,	The prevention and control of Winter and Spring Flu 2020 and COVID-19 in Shandong province	Prevention	168	Prevention of diabetes	Flos Lonicerae(金银花, Jin Yin Hua)12g, Adenophora stricta Miq.(沙参, Sha Shen)10g, Radix Ophiopogonis(麦冬, Mai Dong)15g, Rhizoma Phragmitis(芦根, Lu Gen)15g, Semen Coicis(生薏苡仁,	The prevention and control of Winter and Spring Flu 2020 and COVID-19 in Shandong province	Prevention	168	Prevention of diabetes

		Sheng Yi Yi Ren)15g					Sheng Yi Yi Ren)15g				
169	Prevention for children (Antipyretic prevention)	Herba Taraxaci(蒲公英, Pu Gong Ying)6g, Fructus Arctii(牛蒡子,Niu Bang Zi)3g, Radix Glycyrrhizae(生甘草, Sheng Gan Cao)1.5g	The prevention and control of Winter and Spring Flu 2020 and COVID-19 in Shandong province	Prevention	169	Prevention for children (Antipyretic prevention)	Herba Taraxaci(蒲公英, Pu Gong Ying)6g, Fructus Arctii(牛蒡子,Niu Bang Zi)3g, Radix Glycyrrhizae(生甘草, Sheng Gan Cao)1.5g	The prevention and control of Winter and Spring Flu 2020 and COVID-19 in Shandong province	Prevention	169	Prevention for children (Antipyretic prevention)
170	Formula IIfor General population adults	Rhizoma Phragmitis(芦根, Lu Gen)30 克, Rhizoma Imperatae(白茅根,Bai Mao Gen)30 克, Radix Glycyrrhizae(生甘草, Sheng Gan Cao)10 克, Radix Platycodonis(桔梗,Jie Geng)10 克, Radix Astragali seu	The prevention and control of COVID-19 in Henan province (Trial)	Prevention	170	Formula IIfor General population adults	Rhizoma Phragmitis(芦根, Lu Gen)30 克, Rhizoma Imperatae(白茅根,Bai Mao Gen)30 克, Radix Glycyrrhizae(生甘草, Sheng Gan Cao)10 克, Radix Platycodonis(桔梗,Jie Geng)10 克, Radix Astragali seu	The prevention and control of COVID-19 in Henan province (Trial)	Prevention	170	Formula IIfor General population adults

171	Prescription III for prevention of Special population	Hedysari(黄芪,Huang Qi)10 克 Radix Ophiopogonis(麦冬, Mai Dong)5, Flos Chrysanthemi(菊花, Ju Hua)2, Rhizoma Belamcandae(射干, She Gan)2g, Folium Mori(桑叶, Sang Ye)2g, Canarium album (Lour.) Raeusch.(青果,Qing Gu)2g, RhizomaCyrtomiiFortunei(贯众, Guan Zhong)2g	The prevention and control of COVID-19 in Henan province (Trial)	Prevention	171	Prescription III for prevention of Special population	Hedysari(黄芪,Huang Qi)10 克 Radix Ophiopogonis(麦冬, Mai Dong)5, Flos Chrysanthemi(菊花, Ju Hua)2, Rhizoma Belamcandae(射干, She Gan)2g, Folium Mori(桑叶, Sang Ye)2g, Canarium album (Lour.) Raeusch.(青果,Qing Gu)2g, RhizomaCyrtomiiFortunei(贯众, Guan Zhong)2g	The prevention and control of COVID-19 in Henan province (Trial)	Prevention	171	Prescription III for prevention of Special population
172	Prevention for children (Jiedu Fanggan Formula)	Flos Lonicerae(金银花, Jin Yin Hua)6g, Herba Taraxaci(蒲公英, Pu Gong Ying)3g, Radix Glycyrrhizae(生甘草, Sheng Gan	The prevention and control of Winter and Spring Flu 2020 and COVID-19 in Shandong province	Prevention	172	Prevention for children (Jiedu Fanggan Formula)	Flos Lonicerae(金银花, Jin Yin Hua)6g, Herba Taraxaci(蒲公英, Pu Gong Ying)3g, Radix Glycyrrhizae(生甘草, Sheng Gan	The prevention and control of Winter and Spring Flu 2020 and COVID-19	Prevention	172	Prevention for children (Jiedu Fanggan Formula)

		Cao)1.5g					Cao)1.5g	in Shandong province			
173	Prevention for children (Prevention of Fuzheng)	Radix Astragali seu Hedysari(生黄芪, Sheng Huang Qi)6g, Radix Scutellariae(黄芩, Huang Qin)3g, Radix Glycyrrhizae(甘草, Gan Cao)1.5g	The prevention and control of Winter and Spring Flu 2020 and COVID-19 in Shandong province	Prevention	173	Prevention for children (Prevention of Fuzheng)	Radix Astragali seu Hedysari(生黄芪, Sheng Huang Qi)6g, Radix Scutellariae(黄芩, Huang Qin)3g, Radix Glycyrrhizae(甘草, Gan Cao)1.5g	The prevention and control of Winter and Spring Flu 2020 and COVID-19 in Shandong province	Prevention	173	Prevention for children (Prevention of Fuzheng)
174	Suggested Prescription of professor Liu Shangyi(Chinese Medicine Master)	Radix Glehniae(北沙参, Bei Sha Shen)10g, Rhizoma Polygonati Odorati(玉竹, Yu Zhu)20g, Herba Dendrobii(石斛, Shi Hu)20g, RhizomaCyrtomiiFortunei(贯众, Guan Zhong)20g, Rhizoma	Prevention of "COVID-19" Chinese medicine Prescription, ethnic medicine Prescription	Prevention	174	Suggested Prescription of professor Liu Shangyi(Chinese Medicine Master)	Radix Glehniae(北沙参, Bei Sha Shen)10g, Rhizoma Polygonati Odorati(玉竹, Yu Zhu)20g, Herba Dendrobii(石斛, Shi Hu)20g, RhizomaCyrtomiiFortunei(贯众, Guan Zhong)20g, Rhizoma	Prevention of "COVID-19" Chinese medicine Prescription, ethnic medicine Prescription	Prevention	174	Suggested Prescription of professor Liu Shangyi(Chinese Medicine Master)

175	Prevention of hypertension	Atractylodis(苍术, Cang Shu)10g, Rhizoma Acori Tatarinowii(石菖蒲, Shi Chang Pu)10g Flos Chrysanthemi(菊花, Ju Hua)15g, Ramulus Uncariae Cum Uncis(钩藤, Gou Teng)12g, Radix Paeoniae Alba(白芍, Bai Shao)9g, Lycium barbarumL.(枸杞, Gou Qi)10g, Poria(茯苓, Fu Ling)9g	The prevention and control of Winter and Spring Flu 2020 and COVID-19 in Shandong province	Prevention	175	Prevention of hypertensio n	Atractylodis(苍术, Cang Shu)10g, Rhizoma Acori Tatarinowii(石菖蒲, Shi Chang Pu)10g Flos Chrysanthemi(菊花, Ju Hua)15g, Ramulus Uncariae Cum Uncis(钩藤, Gou Teng)12g, Radix Paeoniae Alba(白芍, Bai Shao)9g, Lycium barbarumL.(枸杞, Gou Qi)10g, Poria(茯苓, Fu Ling)9g	The prevention and control of Winter and Spring Flu 2020 and COVID-19 in Shandong province	Prevention	175	Prevention of hypertension
176	Suhexiang Pill	Styrax(苏合香, Su He Xiang)50g , Benzoinum(安息香, An Xi Xiang)100g, Borneolum Syntheticum(冰片, Bing Pian)50g ,	1, The prevention and control of COVID-19 in Shanxi province (Trial)	Critical illness	176	Suhexiang Pill	Styrax(苏合香, Su He Xiang)50g , Benzoinum(安息香, An Xi Xiang)100g, Borneolum Syntheticum(冰片, Bing Pian)50g ,	1, The prevention and control of COVID-19 in Shanxi province	Critical illness	176	Suhexiang Pill

Cornu Bubali(水牛角, Shui Niu Jiao)Concentrated Powder200g , Moschus(麝香, She Xiang)75g, Lignum Santali Albi(檀香,Tan Xiang)100g , Lignum Aquilariae Resinatum(沉香, chenxiang)100g , Flos Caryophylli(丁香, Ding Xiang)100g, Rhizoma Cyperi(香附, Xiang Fu)100g , Radix Aucklandiae9(木香, Mu Xiang)100g, Olibanum (乳香, Ru Xiang)(制)100g, Fructus Piperis Longi(荜茇, Bi Ba)100g, Rhizoma	2, Diagnosis and treatment of COVID-19(Trial version 4)

Cornu Bubali(水牛角, Shui Niu Jiao)Concentrated Powder200g , Moschus(麝香, She Xiang)75g, Lignum Santali Albi(檀香,Tan Xiang)100g , Lignum Aquilariae Resinatum(沉香, chenxiang)100g , Flos Caryophylli(丁香, Ding Xiang)100g, Rhizoma Cyperi(香附, Xiang Fu)100g , Radix Aucklandiae9(木香, Mu Xiang)100g, Olibanum (乳香, Ru Xiang)(制)100g, Fructus Piperis Longi(荜茇, Bi Ba)100g, Rhizoma	(Trial) 2, Diagnosis and treatment of COVID-19 (Trial version 4)

177	Shexiang Niuhuang Pill	Atractylodis Macrocephalae(白术, Bai Zhu)100g, Fructus Chebulae(诃子肉, He Zi Rou)100g, Cinnabaris(朱砂, Zhu Sha)100g Calculus Bovis(牛黄, Niu Huang)3g, Moschus(麝香, She Xiang)1.5g, Radix Saposhnikoviae(防风, Fang Feng)9g, Radix Paeoniae Rubra(赤芍, Chi Shao)15g, Rhizoma Coptidis(黄连, Huang Lian)15g, Radix et Rhizoma Rhei(大黄, Da Huang)30g, Ramulus Uncariae Cum Uncis(钩藤, Gou Teng)15g,	The prevention and control of COVID-19 in Shanxi province (Trial)	Critical illness	177	Shexiang Niuhuang Pill	Atractylodis Macrocephalae(白术, Bai Zhu)100g, Fructus Chebulae(诃子肉, He Zi Rou)100g, Cinnabaris(朱砂, Zhu Sha)100g Calculus Bovis(牛黄, Niu Huang)3g, Moschus(麝香, She Xiang)1.5g, Radix Saposhnikoviae(防风, Fang Feng)9g, Radix Paeoniae Rubra(赤芍, Chi Shao)15g, Rhizoma Coptidis(黄连, Huang Lian)15g, Radix et Rhizoma Rhei(大黄, Da Huang)30g, Ramulus Uncariae Cum Uncis(钩藤, Gou Teng)15g,	The prevention and control of COVID-19 in Shanxi province (Trial)	Critical illness	177	Shexiang Niuhuang Pill

Fructus Forsythiae(连翘, Lian Qiao)30g, Cortex Phellodendri(黄柏, Huang Bo)15g, Gardenia jasminoides Ellis(栀子, Zhi Zi)15g, Flos Lonicerae(金银花, Jin Yin Hua)30g, Radix Ophiopogonis(麦冬, Mai Dong)9g, Radix Platycodonis(桔梗, Jie Geng)12g, Radix Angelicae Sinensis(当归, Dang Gui)15g, Radix Scutellariae(黄芩, Huang Qin15g, Radix Glycyrrhizae(甘草, Gan Cao)9g,

Fructus Forsythiae(连翘, Lian Qiao)30g, Cortex Phellodendri(黄柏, Huang Bo)15g, Gardenia jasminoides Ellis(栀子, Zhi Zi)15g, Flos Lonicerae(金银花, Jin Yin Hua)30g, Radix Ophiopogonis(麦冬, Mai Dong)9g, Radix Platycodonis(桔梗, Jie Geng)12g, Radix Angelicae Sinensis(当归, Dang Gui)15g, Radix Scutellariae(黄芩, Huang Qin15g, Radix Glycyrrhizae(甘草, Gan Cao)9g,

178	Shufeng Jiedu Capsule (Granule)	Gypsum Fibrosum(石膏, Shi Gao)30g, Realgar(雄黄, Xiong Huang)15g, Cinnabaris(朱砂, Zhu Sha)30g, Borneolum Syntheticum(冰片, Bing Pian)15g, Herba Menthae Heplocalycis(薄荷, Bo He)3g Rhizoma Polygoni Cuspidati(虎杖, Hu Zhang), Fructus Forsythiae(连翘, Lian Qiao), Radix Isatidis(板蓝根, Ban Lan Gen), Radix Bupleuri(柴胡, Chai Hu), Herba Patriniae (败酱草, Bai Jiang Cao), Herba Verbenae(马鞭草, Ma Bian	1, The prevention and control of COVID-19 in Shanxi province (Trial)<br>2, Diagnosis and treatment of COVID-19(Trial version 6)	Prevention /Early stage	178	Shufeng Jiedu Capsule (Granule)	Gypsum Fibrosum(石膏, Shi Gao)30g, Realgar(雄黄, Xiong Huang)15g, Cinnabaris(朱砂, Zhu Sha)30g, Borneolum Syntheticum(冰片, Bing Pian)15g, Herba Menthae Heplocalycis(薄荷, Bo He)3g Rhizoma Polygoni Cuspidati(虎杖, Hu Zhang), Fructus Forsythiae(连翘, Lian Qiao), Radix Isatidis(板蓝根, Ban Lan Gen), Radix Bupleuri(柴胡, Chai Hu), Herba Patriniae (败酱草, Bai Jiang Cao), Herba Verbenae(马鞭草, Ma Bian	1, The prevention and control of COVID-19 in Shanxi province (Trial) 2, Diagnosis and treatment of	Prevention /Early stage	178	Shufeng Jiedu Capsule (Granule)

		Cao), Rhizoma Phragmitis(芦根, Lu Gen), Radix Glycyrrhizae(甘草, Gan Cao)					Cao), Rhizoma Phragmitis(芦根, Lu Gen), Radix Glycyrrhizae(甘草, Gan Cao)	COVID-19 (Trial version 6)			
179	Xingnaojing Injection	Moschus(麝香, She Xiang), Radix Curcumae(郁金, Yu Jin), Borneolum Syntheticum(冰片, Bing Pian), Gardenia jasminoides Ellis(栀子, Zhi Zi). The auxiliary materials are polysorbate 80 and sodium chloride	Diagnosis and treatment of COVID-19(Trial version 6/7)	Critical illness	179	Xingnaojing Injection	Moschus(麝香, She Xiang), Radix Curcumae(郁金, Yu Jin), Borneolum Syntheticum(冰片, Bing Pian), Gardenia jasminoides Ellis(栀子, Zhi Zi). The auxiliary materials are polysorbate 80 and sodium chloride	Diagnosis and treatment of COVID-19 (Trial version 6/7)	Critical illness	179	Xingnaojing Injection
180	Pudilan Xiaoyan Tablet	Herba Taraxaci(蒲公英, Pu Gong Ying), Radix Scutellariae(黄芩, Huang Qin), Corydalis Bungeanae Herba(苦地丁, Ku Di Ding), Radix	The prevention and control of COVID-19 in Hainan province (The public version of the second edition of the trial)	Early stage/Progressive stage	180	Pudilan Xiaoyan Tablet	Herba Taraxaci(蒲公英, Pu Gong Ying), Radix Scutellariae(黄芩, Huang Qin), Corydalis Bungeanae Herba(苦地丁, Ku Di Ding), Radix	The prevention and control of COVID-19 in Hainan province (The public	Early stage/Progressive stage	180	Pudilan Xiaoyan Tablet

		Isatidis(板蓝根, Ban Lan Gen)					Isatidis(板蓝根, Ban Lan Gen)	version of the second edition of the trial)			
181	Qingkailing Oral Liquid	Cholic acid, Concha Margaritifera(珍珠母, Zhen Zhu Mu), hyodeoxycholic acid, Gardenia jasminoides Ellis(栀子, Zhi Zi), Cornu Bubali(水牛角, Shui Niu Jiao), Radix Isatidis(板蓝根, Ban Lan Gen), Baicalin , Flos Lonicerae(金银花, Jin Yin Hua)	The prevention and control of COVID-19 in Hainan province (The public version of the second edition of the trial)	Early stage/Progressive stage	181	Qingkailing Oral Liquid	Cholic acid, Concha Margaritifera(珍珠母, Zhen Zhu Mu), hyodeoxycholic acid, Gardenia jasminoides Ellis(栀子, Zhi Zi), Cornu Bubali(水牛角, Shui Niu Jiao), Radix Isatidis(板蓝根, Ban Lan Gen), Baicalin , Flos Lonicerae(金银花, Jin Yin Hua)	The prevention and control of COVID-19 in Hainan province (The public version of the second edition of the trial)	Early stage/Progressive stage	181	Qingkailing Oral Liquid
182		Cortex Eucommiae(杜仲, Du Zhong)30-45g, Radix Dipsaci(川断, Chuan Duan)30-45g, Radix Angelicae Sinensis(当归,	The prevention and control of COVID-19 in Gansu province (Trial)	Prevention（Foot-bathing Decoction）	182		Cortex Eucommiae(杜仲, Du Zhong)30-45g, Radix Dipsaci(川断, Chuan Duan)30-45g, Radix Angelicae Sinensis(当归,	The prevention and control of COVID-19 in Gansu province (Trial)	Prevention（Foot-bathing Decoction）	182	

		Dang Gui)15-20g, Radix Astragali seu Hedysari(黄芪, Huang Qi)30-45g, Herba Agastaches(藿香, Huo Xiang)15-30g, Rhizoma Zingiberis Recens(生姜, Sheng Jiang)15-20g					Dang Gui)15-20g, Radix Astragali seu Hedysari(黄芪, Huang Qi)30-45g, Herba Agastaches(藿香, Huo Xiang)15-30g, Rhizoma Zingiberis Recens(生姜, Sheng Jiang)15-20g				
183	Xiyanping Injection	Andrographolide sulfonate	Diagnosis and treatment of COVID-19 (Trial version 6/7)	Critical illness（Chinese patent medicine is recommended）	183	Xiyanping Injection	Andrographolide sulfonate	Diagnosis and treatment of COVID-19 (Trial version 6/7)	Critical illness（Chinese patent medicine is recommended）	183	Xiyanping Injection
184	Feiyuling Spray	Rhizoma Polygonati Odorati(玉竹, Yu Zhu), (克龙母参, Ke Long Mu Shen), Radix Scutellariae(黄芩, Huang Qin), Fructus	Professor Chen Hua	Early stage/Progressive stage/Critical illness	184	Feiyuling Spray	Rhizoma Polygonati Odorati(玉竹, Yu Zhu), (克龙母参, Ke Long Mu Shen), Radix Scutellariae(黄芩, Huang Qin), Fructus Forsythiae(连翘,	Professor Chen Hua	Early stage/Progressive stage/Critical illness	184	Feiyuling Spray

		Forsythiae(连翘, Lian Qiao), RhizomaCyrtomiiFortunei(贯众, Guan Zhong), Tetrastigma hemsleyanum Diels et Gilg(三叶青, San Ye Qing), Flos Lonicerae(金银花, Jin Yin Hua), Bulbus Fritillariae Cirrhosae(川贝母, Chuan Bei Mu), Rhizoma Bolbostemmatis(土贝母, Tu Bei Mu), Sarcandra glabra (九节茶, Jiu Jie Cha), Rhizoma Belamcandae(射干, She Gan), Folium Mori(桑叶, Sang Ye)					Lian Qiao), RhizomaCyrtomiiFortunei(贯众, Guan Zhong), Tetrastigma hemsleyanum Diels et Gilg(三叶青, San Ye Qing), Flos Lonicerae(金银花, Jin Yin Hua), Bulbus Fritillariae Cirrhosae(川贝母, Chuan Bei Mu), Rhizoma Bolbostemmatis(土贝母, Tu Bei Mu), Sarcandra glabra (九节茶, Jiu Jie Cha), Rhizoma Belamcandae(射干, She Gan), Folium Mori(桑叶, Sang Ye)				
185	Tanreqing Injection	Radix Scutellariae(黄芩, Huang Qin), (熊胆	Diagnosis and treatment of COVID-19 (Trial	Critical illness （Chinese	185	Tanreqing Injection	Radix Scutellariae(黄芩, Huang Qin), (熊胆	Diagnosis and treatment	Critical illness （Chinese	185	Tanreqing Injection

粉, Xiong Dan Fen), Cornu caprae hircus(山羊角, Shan Yang Jiao), Flos Lonicerae(金银花, Jin Yin Hua), Fructus Forsythiae(连翘, Lian Qiao), The auxiliary material is propanediol	version 6/7)	patent medicine is recommended）	粉, Xiong Dan Fen), Cornu caprae hircus(山羊角, Shan Yang Jiao), Flos Lonicerae(金银花, Jin Yin Hua), Fructus Forsythiae(连翘, Lian Qiao), The auxiliary material is propanediol	of COVID-19 (Trial version 6/7)	patent medicine is recommended）

185 TCM recipes were collected throughout nationwide for combating COVID-19. The details were shown here. NO.1 to NO.130 were the recipes could trace back basic prescriptions. Some remedies attributed to top10 basic formulas would showed in Fig 2A as well, and the corrsponded names were indicated in the last line. The raw data showing the correlation between the clinical and basic remdies were presented at the last seven lines. Specifically, the list of intersection showed the common amounts within the both, regardless of monarch and stratum herbs. If one clinically applied remedy could correspond to more than one basic remedies, the amounts would be shown in sequence and divided by "/". We then calculated the similarity, using the amounts of intersection divided by the basic amounts. In addition, as for monarch or stratum herbs, we used A/B (A showed the actual amounts in clinical recipes, and B showed the original amounts) to describe various circumstances, except for the number "1" indicated the entire cosisitence (the clinically applied recipes contained all of the monarch or remedies), while "0" showed the contrary. Composing herbs within each remedy were named by Latin name (Chinese name, Chinese Pin Yin), e.g., Flos Lonicerae(金银花, Jin Yin Hua). References showed the recipe which originated from, including The Diagnosis and Treatment Protocol for COVID-19 published by national and local health administration, renown experts, agreed hospital recipes and TCM patents, etc. The applying course of each remedy was showed in the "unified phase" based on clinical indications from the "references", including preventive, developing, severe and recovery stage. In particular, different terms were used for database searching, such as "prevention”, “medical observation period” and the “suspects" for preventive stage; "mild”, “heat in the early period”, “acute stage”, “medium”, “influenza”, and “pneumonia" for developing stage; "seriously ill”, “very

period”, “severe”, and “ICU" for severe phase; while "recovery” and “convalescence" for the recovery stage.

Table S4. The all list of 210 used herbs among 185 recipes.

NO.	Herbs(Latin scientific name (Chinese name, Chinese Pinyin))	Acronym	English Name	Frequency
1	Radix Glycyrrhizae(甘草, Gan Cao)	GC	Liquorice Root	84
2	Semen Armeniacae Amarum(杏仁, Xing Ren)	XR	Bitter Apricot Seed	59
3	Flos Lonicerae(金银花, Jin Yin Hua)	JYH	Honeysuckle Bud and Flower	55
4	Fructus Forsythiae(连翘, Lian Qiao)	LQ	Weeping Forsythia Capsule	51
5	Gypsum Fibrosum(石膏, Shi Gao)	SG	Gypsum	46
6	Herba Ephedrae(麻黄, Ma Huang)	MH	Ephedra	45
7	Radix Scutellariae(黄芩, Huang Qin)	HQin	Baical Skullcap Root	42
8	Herba Agastaches(藿香, Huo Xiang)	HX	Wrinkled Gianthyssop Herb	42
9	Radix Astragali seu Hedysari(黄芪, Huang Qi)	HQ	Milkvetch Root	40
10	Rhizoma Phragmitis(芦根, Lu Gen)	LG	Reed Rhizome	34
11	Rhizoma Atractylodis Macrocephalae(白术, Bai Zhu)	BZ	Largehead Atractylodes Rhizome	34
12	Poria(茯苓, Fu Ling)	FL	Indian Bread	31
13	Rhizoma Atractylodis(苍术, Cang Shu)	CZ	Atractylodes Rhizome	31
14	Radix Platycodonis(桔梗, Jie Geng)	JG	Platycodon Root	30
15	Cortex Magnoliae Officinalis(厚朴, Hou Po)	HP	Officinal Magnolia Bark	27
16	Rhizoma Pinelliae(半夏, Ban Xia)	BX	Pinellia Tuber	26

17	Pericarpium Citri Reticulatae(陈皮, Chen Pi)	CP	Dried Tangerine Peel	25
18	Radix et Rhizoma Rhei(大黄, Da Huang)	DH	Rhubarb Root and Rhizome	25
19	Semen Coicis(薏苡仁, Yi Yi Ren)	YYR	Coix Seed	25
20	Radix Ophiopogonis(麦冬, Mai Dong)	MD	Dwarf Lilyturf Tuber	23
21	Radix Saposhnikoviae(防风, Fang Feng)	FF	Divaricate Saposhnikovia Root	23
22	Herba Menthae Heplocalycis(薄荷, Bo He)	BH	Wild Mint Herb	21
23	RhizomaCyrtomiiFortunei(贯众, Guan Zhong)	GZ	Fortunes Boss Fern Rhizome	20
24	Radix Ginseng(人参, Ren Shen)	RS	Ginseng	20
25	Semen Lepidii(葶苈子, Ting Li Zi)	TLZ	Pepperweed Seed	20
26	Fructus Tsaoko(草果, Cao Guo)	CG	Fruit of Caoguo	18
27	Radix Paeoniae Rubra(赤芍, Chi Shao)	CSh	Peony Root	17
28	Rhizoma Anemarrhenae(知母, Zhi Mu)	ZM	Common Anemarrhena Rhizome	15
29	Radix Aconiti Lateralis Preparata(附子, Fu Zi)	FZ	Prepared Common Monkshood Branched Root	15
30	Adenophora stricta Miq.(沙参, Sha Shen)	SS	Root of Straight Ldybell	14
31	Fructus Arctii(牛蒡子, Niu Bang Zi)	NBZ	Great Burdock Achene	14
32	Radix Bupleuri(柴胡, Chai Hu)	CH	Chinese Thorowax Root	14
33	Rhizoma Zingiberis Recens(生姜, Sheng Jiang)	SJ	Fresh Ginger	13
34	Bulbus Fritillariae Thunbergii(浙贝母, Zhe Bei Mu)	ZBM	Thunberbg Fritillary Bulb	13
35	Cortex Mori(桑白皮, Sang Bai Pi)	SBP	White Mulberry Root-bark	13
36	Semen Arecae(槟榔, Bin Lang)	BL	Areca Seed	13
37	Herba Schizonepetae(荆芥, Jing Jie)	JJ	Fineleaf Schizonepeta Herb	13
38	Lophatherum gracile(淡竹叶, Dan Zhu Ye)	DZY	Folium Phyllostachytis	13
39	Fructus Amomi Rotundus(豆蔻, Jave Amonum Fruit)	DK	Jave Amonum Fruit	13
40	Fructus Trichosanthis(瓜蒌, Gua Lou)	GL	Snakegourd Fruit	12

41	Rhinoceros unicornis L. (犀角, Xi Jiao)	XJ	Rhinoceros Horn	11
42	Talcum(滑石, Hua Shi)	HS	Talc	11
43	Fructus Schisandrae Chinensis(五味子, Wu Wei Zi)	WWZ	Chinese Magnoliavine Fruit	11
44	Rhizoma Coptidis(黄连, Huang Lian)	HL	Golden Thread	10
45	Calculus Bovis(牛黄, Niu Huang)	NH	Bezoar	10
46	Radix Codonopsis(党参, Dang Shen)	DS	Tangshen	10
47	Semen Persicae(桃仁, Tao Ren)	TR	Peach Seed	10
48	Radix Scrophulariae(玄参, Xuan She)	XS	Figwort Root	10
49	Radix Isatidis(板蓝根, Ban Lan Gen)	BLG	Isatis Root	10
50	Fructus Corni(山茱萸, Shan Zhu Yu)	SZY	Asiatic Cornelian Cherry Fruit	9
51	Rhizoma et Radix Notopterygii(羌活, Qiang Huo)	QH	Incised Notopterygium Rhizome and Root	9
52	Folium Mori(桑叶, Sang Ye)	SY	Mulberry Leaf	9
53	Cornu Bubali(水牛角, Shui Niu Jiao)	SNJ	Buffalo Horn	9
54	Radix Pseudostellariae(太子参, Tai Zi Shen)	TZS	Heterophylly Falsestarwort Root	9
55	Flos Chrysanthemi(菊花, Ju Hua)	JH	Chrysanthemum Flower	9
56	Folium Perillae(紫苏叶, Zi Su Ye)	ZSY	Perilla Leaf	8
57	Moschus(麝香, She Xiang)	SX	Musk	7
58	Gardenia jasminoides Ellis(栀子, Zhi Zi)	ZZ	GARDENIAE FRUCTUS	7
59	Radix Curcumae(郁金, Yu Jin)	YJ	Turmeric Root Tuber	7
60	Periostracum Cicadae(蝉蜕, Chan Tui)	CT	Cicada Slough	7
61	Folium Isatidis(大青叶, Da Qing Ye)	DQY	Dyers Woad Leaf	7
62	Fructus Jujubae(大枣, Da Zao)	DZ	Chinese Date	7
63	Herba Eupatorii(佩兰, Pei Lan)	PL	Fortune Eupatorium Herb	7
64	Radix Paeoniae Alba(白芍, Bai Shao)	BS	Debark Peony Root	7

65	Lumbricus(地龙, Di Long)	DL	Earthworm	7
66	Rhizoma Belamcandae(射干, She Gan)	SGa	Blackberry Lily Rhizome	7
67	Semen Sojae Preparatum(淡豆豉, Dan Dou Chi)	DDC	Fermented Soybean	6
68	Semen Dolichoris Album(白扁豆, Bai Bian Dou)	BBD	Hyacinth Bean	6
69	Herba Artemisiae Annuae(青蒿, Qing Hao)	QH	Sweet Wormwood Herb	6
70	Polyporus Umbellatus(猪苓, Zhu Ling)	ZL	Agaric	6
71	Radix Angelicae Sinensis(当归, Dang Gui)	DG	Chinese Angelica	6
72	Rhizoma Ligustici Chuanxiong(川芎, Chuan Xiong)	CXi	Sichuan Lovage Rhizome	6
73	Radix Rehmanniae Recens(生地, Shen Di)	SD	Unprocessed Rehmannia Root	6
74	Fructus Amomi Villosi(砂仁, Sha Ren）	SR	Villous Amomum Fruit	6
75	Radix Ginseng Rubra(红参, Hong Shen)	HoS	Red Ginseng	6
76	Cinnabaris(朱砂, Zhu Sha)	ZS	Cinnabar	5
77	Borneolum Syntheticum(冰片, Bing Pian)	BP	Borneol	5
78	Bulbus Lilii(百合, Bai He)	BH	Lily Bulb	5
79	Ramulus Cinnamomi(桂枝, Gui Zhi)	GZ	Cassia Twig	5
80	Bombyx Batryticatus(僵蚕, Jiang Can)	JC	BOMBYX BATRYTICATUS	5
81	Medulla Tetrapanacis(通草, Tong Cao)	TC	Ricepaperplant Pith	5
82	Radix Panacis Quinquefolii(西洋参, Xi Yang Shen)	XYS	American Ginseng	5
83	Rhizoma Alismatis(泽泻, Ze Xie)	ZX	Oriental Waterplantain Rhizome	5
84	Radix Angelicae Dahuricae(白芷, Bai Zhi)	BaZ	Dahurian Angelica Root	5
85	Moutan Cortex(丹皮, Dan Pi)	DP	Tree Peony Bark	5
86	Fructus Aurantii(枳壳, Zhi Qiao)	ZQ	AURANTII FRUCTUS	5
87	Radix Salviae Miltiorrhizae(丹参, Dan Shen)	DS	Danshen Root	4
88	Crataegus pinnatifida(焦山楂, Jiao Shan Zha)	JSZ	Charred FRUCTUS CRATAEGI	4

89	Semen Raphani(莱菔子, Lai Fu Zi)	LFZ	Radish Seed	4
90	Folium Eriobotryae(枇杷叶, Pi Pa Ye)	PPY	Loquat Leaf	4
91	Herba Taraxaci(蒲公英, Pu Gong Ying)	PGY	Dandelion	4
92	Rhizoma Acori Tatarinowii(石菖蒲, Shi Chang Pu)	SCP	Grassleaf Sweetflag Rhizome	4
93	Herba Dendrobii(石斛, Shi Hu)	SH	Dendrobium	4
94	Fructus Aurantii Immaturus(枳实, Zhi Shi)	ZSh	Immature Orange Fruit	4
95	Herba Houttuyniae(鱼腥草, Yu Xing Cao)	YXC	Heartleaf Houttuynia Herb	4
96	(建曲, Jian Qu)	JQ	/	4
97	Radix Ginseng(生晒参, Sheng Shai Shen)	SSS	Dried Fresh Ginseng	4
98	Radix Puerariae(葛根, Ge Gen)	GG	Kudzuvine Root	3
99	Rhizoma Curcumae Longae(姜黄, Jiang Huang)	JH	Turmeric	3
100	Rhizoma Dioscoreae(山药, Shan Yao)	SYa	Common Yam Rhizome	3
101	Rhizoma Polygonati Odorati(玉竹,Yu Zhu)	YZ	Fragrant Solomonseal Rhizome	3
102	Radix Lithospermi(紫草, Zi Cao)	ZC	Gromwell Root	3
103	Styrax(苏合香, Su He Xiang)	SHX	Storax	3
104	Fructus Hordei Germinatus(炒麦芽, Chao Mai Ya)	CMY	Germinated Barley	3
105	Rhizoma Arisaematis Cum Bile(胆南星, Dan Nan Xing)	DNX	Bile Arisaema	3
106	Rhizoma Zingiberis(干姜, Gan Jiang)	GJ	Zingiber	3
107	Rhizoma Paridis(重楼, Chong Lou)	CL	Paris Root	3
108	Radix Peucedani(前胡, Qian Hu)	QH	Hogfennel Root	3
109	Flos Carthami(红花, Hong Hua)	HH	Safflower	3
110	Citri Exocarpium Rubrum(橘红, Ju Hong)	JH	dried tangerine peel	2
111	Realgar(雄黄, Xiong Huang)	XH	Realgar	2
112	Massa Medicata Fermentata(神曲, Shen Qu)	SQ	Medicated Leaven	2
113	Radix Aucklandiae9(木香, Mu Xiang)	MX	Common Aucklandia Root	2
114	Lignum Aquilariae Resinatum（沉香, Chen Xiang）	CX	Chinese Eaglewood	2

115	Flos Caryophylli（丁香, Ding Xiang）	DX	Clove	2
116	Calcitum(寒水石, Han Shui Shi)	HSS	Gypsum Rubrum	2
117	Rhizoma Cimicifugae(升麻, Sheng Ma)	SM	Largetrifoliolious Bugbane Rhizome	2
118	(焦三仙, Jiao San Xian)	JSX	Including 3 herbs: Malt, Burnt Hawthorn, Medicated Leaven	2
119	Fructus Chebulae(诃子肉, He Zi Rou)	HZR	Medicine Terminalia Fruit	2
120	Rhizoma Imperatae(白茅根, Bai Mao Gen)	BMG	Cogon Grass Rhizome	2
121	Rhizoma Cynanchi Stauntonii(白前, Bai Qian)	BQ	Willowleaf Rhizome	2
122	Rhizoma Bolbostemmatis(土贝母, Tu Bei Mu)	TBM	Paniculate Bolbostemma	2
123	Cortex Lycii(地骨皮, Di Gu Pi)	DGP	Chinese Wolfberry Root-bark	2
124	Trollius chinensis(金莲花, Jin Lian Hua)	JLH	Chinese Globeflower Flower	2
125	Retinervus Citri Furctus(橘络, Ju Luo)	JL	Tangerine Pith	2
126	Vigna radiata (Linn.) Wilczek(绿豆, Lv Dou)	LD	Mung Bean, Green Gram	2
127	Herba Verbenae(马鞭草, Ma Bian Cao)	MBC	European verbena herb	2
128	Retinervus Luffae Fructus(丝瓜络, Si Gua Luo)	SGL	Luffa Vegetable Sponge	2
129	Radix Cynanchi Paniculati(徐长卿, Xu Chang Qing)	XCQ	Paniculate Swallowwort Root	2
130	Radix Asteris(紫菀, Zi Wan)	ZW	Tatarian Aster Root	2
131	Ramulus Uncariae Cum Uncis(钩藤, Gou Teng)	GT	Gambir Plant Nod	2
132	Bulbus Fritillariae Cirrhosae(川贝母, Chuan Bei Mu)	CBM	Tendrilleaf Fritillary Bulb	2
133	Rhizoma Polygoni Cuspidati(虎杖, Hu Zhang)	HZ	Giant Knotweed Rhizome	2
134	Radix Notoginseng(三七, San Qi)	SQ	Sanqi	2
135	Fructus Perillae(苏子, Su Zi)	SZ	Perilla Fruit	2
136	Poria Rubra(赤苓, Chi Ling)	CL	light red Indian Bread	1
137	Margarita(珍珠, Zhen Zhu)	ZZ	Pearl	1
138	Magnetitum(磁石, Ci Shi)	CiS	Magnetite	1

139	Cornu Saigae Tataricae(羚羊角, Ling Yang Jiao)	LYJ	Antelope Horn	1
140	mirabilite(朴硝, Po Xiao)	PX	Mirabilitum Depuratum	1
141	saltpetre(硝石, Xiao Shi)	XiS	Saltpeter	1
142	Benzoinum(安息香, An Xi Xiang)	AXX	Benzoin	1
143	Lignum Santali Albi(檀香, Tan Xiang)	TX	Sandalwood	1
144	Rhizoma Cyperi(香附, Xiang Fu)	XF	Nutgrass Galingale Rhizome	1
145	Olibanum (乳香, Ru Xiang)	RX	Frankincense	1
146	Fructus Piperis Longi(荜茇, Bi Ba)	BBa	Long Pepper	1
147	Folium Artemisiae Argyi(艾叶, Ai Ye)	AY	Argy Wormwood Leaf	1
148	Radix Stemonae(百部, Bai Bu)	BB	Stemona Root	1
149	Herba Patriniae (败酱草, Bai Jiang Cao)	BJC	Atrina Glass	1
150	silkworm excrement(蚕砂, Can Sha)	CS	Silkworm sand	1
151	Fructus Setariae Germinatus(炒谷芽, Chao Gu Ya)	CGY	Millet Sprout	1
152	Semen Phaseoli(赤小豆, Chi Xiao Dou)	CXD	Rice Bean	1
153	Radix Dipsaci(川断, Chuan Duan)	CD	Himalayan Teasel Root	1
154	Dioscorea nipponica Makino(穿山龙, Chuan Shan Long)	CSL	Dioscorea Nipponica,JapanEse Yam Rhizome	1
155	Herba Andrographis(穿心莲, Chuan Xin Lian)	CXL	Common Andrographis Herb	1
156	Pericarpium Arecae(大腹皮, Da Fu Pi)	DFP	Areca Peel	1
157	Semen Benincasae(冬瓜仁, Dong Gua Ren)	DGR	Waxgourd Seed	1
158	Flos Farfarae(冬花, Dong Hua)	DH	Common Coltsfoot Flower	1
159	Cortex Eucommiae(杜仲, Du Zhong)	DZ	Eucommia Bark	1
160	Bone fossil of big mammals(煅龙骨, Duan Long Gu)	DLG	Forged Keel	1
161	Oyster(煅牡蛎, Duan Mu Li)	DML	Calcined Oyster	1
162	*Lycium barbarum*L.(枸杞, Gou Qi)	GQ	Lycium chinensis	1
163	lotus petiole(荷梗, He Geng)	HG	Lotus Petiole	1

164	Herba Rhodiolae(红景天, Hong Jing Tian)	HJT	Rose-boot	1
165	japonica Rice(粳米, Jing Mi)	JM	Rice	1
166	Sarcandra glabra (九节茶, Jiu Jie Cha)	JJC	Glabrous Sarcandra Herb	1
167	(克龙母参, Ke Long Mu Shen)	KLMS	/	1
168	(梨皮, Li Pi)	LP	Pear Peel	1
169	Semen Nelumbinis(莲子, Lian Zi)	LZ	Lotus Seed	1
170	Natrii Sulfas(芒硝, Mang Xiao)	MX	Crystallized Sodium Sulfate	1
171	Pericarpium Citri Reticulatae Viride(青皮, Qing Pi)	QP	Immature Tangerine Peel	1
172	Tetrastigma hemsleyanum Diels et Gilg(三叶青, San Ye Qing)	SYQ	Tetrastigma hemsleyanum Diels et Gilg	1
173	Ajuga decumbens thunb (散血草, San Xue Cao)	SXC	Herb of Clarke Boea	1
174	Radix Trichosanthis(天花粉, Tian Hua Fen)	THF	Snakegourd Root	1
175	tabasheer(天竺黄, Tian Zhu Huang)	TZH	Concretio silicea bambusae,tabaschir,tabasheer	1
176	Eupolyphaga Seu Steleophaga(土鳖, Tu Bie)	TB	Ground beetle	1
177	Rumex madaio MakinoR. daiwoo Makino(土大黄, Tu Da Huang)	TDH	Nepal Dock Root	1
178	Radix et Rhizome Achyranthes (土牛膝, Tu Niu Xi)	TNX	Native Achyranthes （root）	1
179	Asarum sieboldii Miq.(细辛, Xi Xin)	XX	Asari Radix	1
180	*Artemisia rupestris* L.(一枝蒿, Yi Zhi Hao)	YZH	Alpine Yarrow Herb	1
181	Herba Artemisiae Scopariae(茵陈, Yin Chen)	YC	Virgate Wormwood Herb	1
182	Folium Ginkgo(银杏叶,Yin Xing Ye)	YXY	Ginkgo Leaf	1
183	Caulis Bambusae in Taenia(竹茹, Zhu Ru)	ZR	Bamboo Shavings	1
184	Perilla frutescens (L.) Britt.(苏梗, Su Geng)	SG	Stem of Common Perilla	1
185	Lignum Sappan(苏木, Su Mu)	SM	Sappan Wood	1
186	Cornu caprae hircus(山羊角, Shan Yang Jiao)	SYJ	Goat Horn	1
187	(熊胆粉, Xiong Dan Fen)	XDF	PULVIS ELLIS URS	1

188	Concha Margaritifera(珍珠母, Zhen Zhu Mu)	ZZM	Nacre	1
189	Corydalis Bungeanae Herba(苦地丁, Ku Di Ding)	KDD	Bunge Corydalis Herb	1
190	Cortex Phellodendri(黄柏, Huang Bo)	HB	Amur Cork-tree	1
191	Rhizoma Smilacis Glabrae(土茯苓, Tu Fu Ling)	TFL	Glabrous Greenbrier Rhizome	1
192	Hirudo(水蛭, Shui Zhi)	SZ	Leech	1
193	Radix angelicae seu hemsley(独活, Du Huo)	DuH	Angelica Tuhov, Radix	1
194	Fructus Ligustri Lucidi(女贞子, Nv Zhen Zi)	NZZ	Fruit of Glossy Privet	1
195	Syzygium jambos Alston (Eugenia jambos L.)(蒲桃, Pu Tao)	PT	Rose Apple	1
196	Gekko gecko(蛤蚧, Ge Jie)	GJ	GECKO	1
197	Caulis Lonicerae(忍冬藤, Ren Dong Teng)	RDT	Honeysuckle Stem	1
198	Euchresta japonica Hook. f. ex Regel (山豆根, Shan Dou Gen)	SDG	Vietnamese Sophora Root	1
199	Serissa serissoides (DC.)Druce(锐过买 (白马骨), Bai Ma Gu)	BMG	Serissa foetida Comm.	1
200	(窝嘎乃 (墨斗菜), Wo Ga Nai)	WGN	/	1
201	Typhonium giganteum Engl. (加格略 (独角莲), Du Jiao Lian)	DJL	RHIZOMA TYPHONII	1
202	Polygonum perfoliatum L. (加欧万囊 (蛇倒退), She Dao Tui)	SDT	Herba Polygoni Perfoliati	1
203	Hyrtanandra hirta (Bl.) Miq. (加嘎旅 (生扯拢), Sheng Che Long)	SCL	Herb of Fanshaped Corallodiscus	1
204	Polygala japonica Houtt. (锐草连 (爪子金), Zhua Zi Jin)	ZZJ	Japanese milkwort	1
205	Saxifraga stolonifera Curt. (窝比省(虎耳草), Hu Er Cao)	HEC	Saxifraga	1
206	Centellaasiatica(L.)Urban(窝比赊溜 (积雪草), Ji Xue Cao)	JXC	HERBA CENTELLAE	1
207	(锐阿都偏 (岩虹豆), Yan Hong Dou)	YHD	/	1
208	Mahonia oiwakensis Hayata(都阿能 (十大功劳), Shi Da Gong Lao)	SDGL	China Mahonia	1
209	Asparagus cochinchinensis(Lour.)Merr.(天冬, Tian Dong)	TD	asparagus cochinchinensis	1
210	Canarium album (Lour.) Raeusch. (青果,Qing Gu)	QG	Chinese olive	1

210 herbs were all listed in various names (herbs Latin name, Chinese name, Chinese Pin Yin, acronyms and English name) here with the frequency applied

among all 185 clinical recipes. Some specific herbs were not equiped with all of the names due to no records could find. e.g., (锐阿都偏 (岩虹豆), Yan Hong Dou), this herb showed without corresponded Latin name and English name (using "/" indicated this condition). The ethnologic herbs (NO. 199 to NO.208) were attached the local name in Chinese, and the general name were listed in the brackets, corresponded with the Chinese Pin Yin, such as Centellaasiatica(L.)Urban(窝比赊溜 (积雪草), Ji Xue Cao).

Table S5. The target count (TC) with directions of 8 herbs.

Targets	HQi	HQin	JYH	GC	LQ	MH	XR	HX	Regulation	Total Target Counts
PTGS2（COX2)	-39	-71	-78	-112	-61	-122	-46	-44	Inhibited	-573
Inos (NOS2)	-13	-23	-12	-73	-6	-4	-4	-9	Inhibited	-144
RELA (P65)	-6	-6	-10	-9	-12	-15	-6	-5	Inhibited	-69
TNF	-5	-5	-11	-6	-10	-19	-6	-5	Inhibited	-67
IL6	-4	-3	-8	-3	-6	-11	-3	-1	Inhibited	-39
MAPK14 (P38α)	-8	-5	-4	-55	-3	-1	-5	-5	Inhibited	-86
CASP3	-5/+5	-8	-11	-7	-10	-16	+2	+3	Inhibited	-47
PTGS1 (COX1)	-28	+/=56	-48	=60	/40	-84	-36	+32	Inhibited	-164
ICAM1	+3	-1	-6	-4	/7	-10	-1	-4	Inhibited	-23
IL1B	-2	-1	+3	-3	-5	-7	-2	-3	Inhibited	-20
TP53（P53）	-2	-4	-5	=/-2+2	-4	/6	+1	/2	Inhibited	-14
BCL2	+4	-8/+8	-8/+8	-5	-7	/10	-3	-3	Inhibited	-14
TGFB1 (TGF-β1)	-1	-2	/2	-1	-2	-4	-1	-1	Inhibited	-12

MAPK1	+1	-0	/2	-3	-2	-4	/1	-2	Inhibited	-10
CXCL8（IL8）	-2	+2	-4	-2	/4	/9	/1	-1	Inhibited	-7
CRP	-1	-0	-1	/1	-1	-2	-1	/1	Inhibited	-6
CCL2	-1	-1	-1	-1	=2	-2	/0	/1	Inhibited	-6
MAPK8	-1	+/-0	-2	-2	/2	/2	/1	-0	Inhibited	-5
FOS (C-FOS)	-2	-2	-3	-3	-3	+6	/2	+2	Inhibited	-5
IL4	-/+2	+2	-2	-1	+1	-5	-0	+1	Inhibited	-4
IL1A	/1	-0	-1	/1	-1	-2	-0	/1	Inhibited	-4
STAT1	-3	-0	/2	-2	+3	=4	/0	/1	Inhibited	-2
EGFR	-1/+1	+0	/2	+1	/2	-4	-0	+1	Activated	-2
CXCL10	+1	-0	/1	-1	/1	-2	/0	/1	Inhibited	-2
SOD1	+3	+1	+2	-6	+2	-5	+3	+1	Activated	+1
PARP1	-1	+/-0	/1	+1	+2	/3	-0	+1	Activated	+3
CASP8	-1	+2	/4	+2	/4	/4	/2	+1	Activated	+4
IFNG (IFN-γ)	-1	-1	+4	-1	-2	+6	+1	/2	Inhibited	-6
IL2	-1	-1	+3	-2	+2	+6	/0	/3	——	7
CAT	+2	+0	+1	+2	+1	+/-/-/-7	+3	+1	Activated	+10
IL10	-/+2	+2	/4	+1	=/-3	+8	-2/+2	+2	Activated	+13
HMOX1	+2	+1	/6	+5	+4	/12	/1	+4	Activated	+16
BAX	-5	+5/-5	-5/+5	+4	+7	+9	+2	+2	Activated	+19
BCL2L1	-1	+2	/4	+2	-3	/7	/2	/2	——	0
HSPA5	-1	-0	/1	-/+1	=1	/2	/0	/1	Inhibited	-1
MCL1	-0	-/+2	/3	-1	-3	/4	/1	/1	Inhibited	-4

Herbs were named in acronyms here. The targets listed here were related to both 8 herbs and COVID-19. The merging database with duplicates were constructed after intersection of herbs and COVID-19 targets. As each compound from an herb might modulate multiple gene targets; thus, multiple ingredients could modulate a particular target multiple times; this frequency is also called target count (TC), which is presented as the number in each cross at the table. The directions regulated by herbs were confirmed by the sufficient literature reports, at least 3 were attached each herb with each target (Next table show the details below). The fore-symbols indicated the regulatory directions, "+" was activated, "-" was inhibited, "=" showed no difference and "/" showed unlisted reports. We then identified the integrated directions of whole herbs based on whether inhibited or activated took the hand, which was shown in the "Regulation" line. For instance, MAPK1 was activated only by HQi but inhibited by 5 herbs and two unlisted, that finally confirmed to inhibited. There were two targets (IL2 and BCL2L1) can't confirm the general orientation, due to neck and neck for inhibited or activated, thus indicated by the symbol "-". Total target counts were the addition of 8 herbs in one target, and the direction was the same as the "Regulation" ("+" was activated, "-" was inhibited and no fore-symbol was the uncertainty).

Table S6. The literature records of the 33 crucial targets regulated from 8 core herbs.

NO.	Herb	Target	Regulation	Key Points of Literature	References
1	HQi(Phenolic derivatives from Radix Astragali)	PTGS2	-1	All four compounds exhibited potent inhibitory effects on TNF-α production and TNF-α, COX-2, IL-1β, IL-6 and iNOS mRNA expression at 50 μM.	Chen W, Zhang YY, Wang Z ,et al.Wang HB. Phenolic derivatives from Radix Astragali and their anti-inflammatory activities. Nat Prod Commun. 9,1577–1580(2014).
2	HQi(Hedysarum polybotrys)	PTGS2	-1	M-EA had the highest formononetin and total proanthocyanidin content and showed stronger inhibitory effects on the production and expression of NO, PGE2, iNOS and COX-2 in LPS-activated RAW 264.7 cells and splenocytes than the other fractions.	Huang GC, Lee CJ, Wang KT, et al. Immunomodulatory effects of Hedysarum polybotrys extract in mice macrophages, splenocytes and leucopenia. Molecules. 18,14862–14875(2013).
3	HQi(Phenolic derivatives from	iNOS	-1	All four compounds exhibited potent inhibitory effects on TNF-α production and TNF-α, COX-2, IL-1β, IL-6	Chen W, Zhang YY, Wang Z, et al. Phenolic derivatives from Radix Astragali and their anti-inflammatory activities. Nat Prod Commun.

	Radix Astragali)			and iNOS mRNA expression at 50 μM.	9,1577–1580(2014).
4	HQi(Hedysarum polybotrys)	iNOS	-1	M-EA had the highest formononetin and total proanthocyanidin content and showed stronger inhibitory effects on the production and expression of NO, PGE2, iNOS and COX-2 in LPS-activated RAW 264.7 cells and splenocytes than the other fractions.	Huang GC, Lee CJ, Wang KT, et al. Immunomodulatory effects of Hedysarum polybotrys extract in mice macrophages, splenocytes and leucopenia. Molecules. 18,14862–14875(2013)
5	HQi(Astragalus Polysaccharide RAP)	iNOS	+1	mRNA of iNOS (Figure 3A), IL-6 (Figure 3B), TNF-a (Figure 3D), and C-X-C motif chemokine 10 (CXCL10) (Figure 3E) were significantly induced in the Astragalus Polysaccharide(RAP)and LPS treatment group compared with the control group.	Wei Wei,Zhi-Peng Li,Zhao-Xiang Bian,et al. Astragalus Polysaccharide RAP Induces Macrophage Phenotype Polarization to M1 via the Notch Signaling Pathway. Molecules. 24,(2019).
6	HQi(total flavonoids of Astragalus)	RELA(P65)	-1	The proliferative capacity of osteoarthritis chondrocytes after treatment with5,10, 20 and 20 mg / L astragalus total flavonoids increased, the content of IL-6 and IL-1β in the cell culture supernatant decreased, and the activities of SOD and GSH-Px increased. NF-κBp65 and MMP-13 protein expression levels decreased ($P < 0.05$)	Ren Weiliang, Jiao Yongwei, Zhang Jian, et al.Effects of total flavonoids of Astragalus on oxidative stress and secretion of inflammatory factors in chondrocytes of osteoarthritis.Journal of Zhengzhou University(Medical Sciences). 54,603-606(2019).
7	HQi(Astragalus polysaccharide (APS))	TNF	-1	Astragalus polysaccharide (APS) can inhibit the secretion of TNFα, NO and IL-1 by activated macrophages to varying degrees	Lu Jingtao, Yang Yan, Chen Minzhu. Effect of astragalus polysaccharides on IL-1, NO and TNFα secreted by lipopolysaccharide-induced normal rat peritoneal macrophages.Acta Universitatis Medicinalis Anhui. 39,(2004).
8	HQi(Astragalus Polysaccharide(RAP))	TNF	+1	mRNA of iNOS (Figure 3A), IL-6 (Figure 3B), TNF-a (Figure 3D), and C-X-C motif chemokine 10 (CXCL10) (Figure 3E) were significantly induced in the Astragalus Polysaccharide(RAP)and LPS treatment	Wei Wei,Zhi-Peng Li,Zhao-Xiang Bian,et al. Astragalus Polysaccharide RAP Induces Macrophage Phenotype Polarization to M1 via the Notch Signaling Pathway. Molecules. 24,(2019).

				group compared with the control group.	
9	HQi(Astragaloside)	TNF	-1	The mental state of mice pretreated with AS-IV improved somewhat and the survival rate increased significantly; the bacterial load of blood, abdominal fluid and various organs decreased; the expression of CXCR2 on the surface of neutrophils was significantly increased, and CXCL2 tended to increase Neutrophils, increase the number of neutrophils in blood and peritoneal fluid; at the same time, the level of inflammatory factor TNF-α decreases	Huang Ping. Study on the effect of astragaloside on the bactericidal activity of neutrophils.Guangdong Pharmaceutical University. (2017).
10	HQi(Astragalus polysaccharides)	TNF	-1	We also found that the decreased viral replication after APS treatment was associated with reduced mRNA levels of the cytokines IL-1B, IL-6, IL-8 and TNF-α	Zhang P, Liu X, Liu H, et al. Astragalus polysaccharides inhibit avian infectious bronchitis virus infection by regulating viral replication. Microb Pathog. 114,124–128(2018).
11	HQi(Astragalus polysaccharides)	TNF	+1	At most time points, the titer of IBV-specific antibodies, lymphocyte proliferation, and IL-1β, IL-2, IL-8, and TNF-α mRNA expression levels were higher in three APS groups than in the vaccine control group, and these increases were dose-dependent.	Zhang P, Wang J, Wang W, et al. Astragalus polysaccharides enhance the immune response to avian infectious bronchitis virus vaccination in chickens. Microb Pathog. 111,81–85(2017).
12	HQi(Astragalus and Codonopsis pilosula polysaccharides)	TNF	-1	COPD showed defective AM phagocytosis and increased levels of interleukin (IL)-6, IL-8, and tumor necrosis factor (TNF)-α in bronchoalveolar lavage fluid and serum. PM2.5 exposure aggravated the damage, and this effect was reversed by APS and CPP gavage.	Chu X, Liu XJ, Qiu JM, Zeng XL, Bao HR, Shu J. Effects of Astragalus and Codonopsis pilosula polysaccharides on alveolar macrophage phagocytosis and inflammation in chronic obstructive pulmonary disease mice exposed to PM2.5. Environ Toxicol Pharmacol. 48, 76–84(2016).
13	HQi(Astragaloside and Baicalin)	TNF	-1	The combination group had reduced levels of IL-1β, IL-8, and TNF-α in LPS-induced MSCs, much more than in the other 2 groups. Compared with the other	Zhu L, Liu YJ, Shen H, Gu PQ, Zhang L. Astragalus and Baicalein Regulate Inflammation of Mesenchymal Stem Cells (MSCs) by the Mitogen-Activated Protein Kinase (MAPK)/ERK Pathway. Med Sci

				groups, the combination of Astragaloside and Baicalin more efficiently reduced IL-1β, IL-8, and TNF-α levels in the LPS-induced MSCs model, and ERK inhibitor was capable of recovering the inflammatory effect.	Monit. 23,3209–3216(2017).
14	HQi(Astragalus)	IL6	-1	Astragalus can reduce the concentration of IL-1, IL-6, IFN-γ, and TNF-α in children with viral myocarditis, and increase the level of HSP70	Niu Ling, An Xinjiang, Xu Hui, et al. Fu Mingyu, Wang Qingwen, Li Chunli, Xu Yongmao.Effect of Astragalus Membranaceus on Cytokines and HSP70 in Children with Viral Myocarditis.Hebei Medicine. 26,154-158(2020).
15	HQi(Phenolic derivatives from Radix Astragali)	IL6	-1	All four compounds exhibited potent inhibitory effects on TNF-α production and TNF-α, COX-2, IL-1β, IL-6 and iNOS mRNA expression at 50 μM.	Chen W, Zhang YY, Wang Z, Luo XH, Sun WC, Wang HB. Phenolic derivatives from Radix Astragali and their anti-inflammatory activities. Nat Prod Commun. 9,1577–1580(2014).
16	HQi(the Astragalus polysaccharide (APS))	IL6	-1	Compared with the control group, the Astragalus polysaccharide (APS) group IL 1β, IL 2, IL 6, IL 12, TNF α, INF γ, Fas, iNOS mRNA expression levels were significantly reduced, IL 4, IL 5, IL 10, TGF β, Bcl 2, SOD mRNA expression levels were significantly up-regulated	Chen Wei, Yu Maohua, Liu Fang.Effects of Astragalus Polysaccharides of Gene Expression of Cytokines of NOD Mice's lslets.Fudan University Journal of Medical Sciences. 607-610（2004）
17	HQi(Both Yupingfeng polysaccharide and Astragalus polysaccharide)	IL6	+1	Both Yupingfeng polysaccharide and Astragalus polysaccharide can significantly increase the levels of SIgA, IL-2, TGF-β1 and IL-6 in mice (P <0.05 or P <0.01)	DENG Hua, YANG Hong, JIANG Yan-ping,et al.Regulatory Effects of Yupingfeng Polysaccharides on Intestinal Mucosal Immune Response and Immune Injury in Mice.Chinese Journal of Veterinary Drug 52, 43-48（2018）.
18	HQi(Cisplatin combined with astragaloside)	IL6	-1	Cisplatin combined with astragaloside IV is superior to cisplatin alone in the treatment of breast cancer in rats ~ + Level, down-regulate IL-1, IL-6, TNF-α, CD8 ~ +	Liu Tiegang, Wen Chunyan, Shen Xuansan.Effects of cisplatin combined with astragaloside on inflammatory factors and immune function in breast cancer rats.Chinese Journal of Gerontology 40,

				level	863-865（2020）.
19	HQi(Astragalus Polysaccharide RAP)	IL6	+1	mRNA of iNOS (Figure 3A), IL-6 (Figure 3B), TNF-a (Figure 3D), and C-X-C motif chemokine 10 (CXCL10) (Figure 3E) were significantly induced in the Astragalus Polysaccharide(RAP)and LPS treatment group compared with the control group.	Wei Wei,Zhi-Peng Li,Zhao-Xiang Bian,et al. Astragalus Polysaccharide RAP Induces Macrophage Phenotype Polarization to M1 via the Notch Signaling Pathway. Molecules. 24,（2019）.
20	HQi(Astragalus polysaccharides)	IL6	-1	We also found that the decreased viral replication after APS treatment was associated with reduced mRNA levels of the cytokines IL-1B, IL-6, IL-8 and TNF-α	Zhang P, Liu X, Liu H, et al. Astragalus polysaccharides inhibit avian infectious bronchitis virus infection by regulating viral replication. Microb Pathog. 114,124–128(2018).
21	HQi(Astragalus and Codonopsis pilosula polysaccharides)	IL6	-1	COPD showed defective AM phagocytosis and increased levels of interleukin (IL)-6, IL-8, and tumor necrosis factor (TNF)-α in bronchoalveolar lavage fluid and serum. PM2.5 exposure aggravated the damage, and this effect was reversed by APS and CPP gavage.	Chu X, Liu XJ, Qiu JM,et al. Effects of Astragalus and Codonopsis pilosula polysaccharides on alveolar macrophage phagocytosis and inflammation in chronic obstructive pulmonary disease mice exposed to PM2.5. Environ Toxicol Pharmacol. 48,76–84(2016).
22	HQi(Astragalus polysaccharide)	MAPK14（P38α）	-1	Astragalus polysaccharide can significantly improve the cardiac function and reduce myocardial injury in diabetic cardiomyopathy model rats, and its role may be related to the inhibition of MAPK signaling pathway activity	Wang Yu, Yu Ruixue, Hu Sibo,et al. Protective effect of astragalus polysaccharide on diabetic cardiomyopathy model rats.The Chinese Journal of Clinical Pharmacology. 35,2754-2758(2019).
23	HQi(Astragalus Polysaccharides)	CASP3	+1	APS significantly promoted anti-proliferative and apoptotic effects of cisplatin on nasopharyngeal carcinoma cells. APS also enhanced the anti-tumor effects and cisplatin-induced apoptosis in the xenograft model. The level of Bcl-2 decreased, while the levels of Bax, caspase-3, and caspase-9 increased in cisplatin combined with APS treatment compared to cisplatin	Zhou Zhen,Meng Minhua,Ni Haifeng,Chemosensitizing Effect of Astragalus Polysaccharides on Nasopharyngeal Carcinoma Cells by Inducing Apoptosis and Modulating Expression of Bax/Bcl-2 Ratio and Caspases. Med. Sci. Monit. 23,462-469(2017).

				only treatment. The ratio of Bax to Bcl-2 was significantly enhanced by the APS to cisplatin	
24	HQi(hedysari polysaccharide)	CASP3	-1	We found that caspase-3 is overexpressed in high glucose-induced apoptosis; HPS can inhibit apoptosis in high glucose state, inhibit the generation of ROS, and inhibit the overexpression of JNK and caspase-3	Liu Jing,Deng Wenjuan,Fan Lei et al. The role of radix hedysari polysaccharide on the human umbilical vein endothelial cells (HUVECs) induced by high glucose. .Eur. J. Intern. Med. 23,287-92(2012).
25	HQi(Astragalus)	PTGS1(COX1)	-1	The WB results of gastric cancer cells show that specific protein bands can be seen at the relative molecular mass of 70X10 ^ 3 in COX-1 groups, and specific protein bands can be seen at 80X10 ^ 3 in the COX-2 groups. Gray scale analysis of each band, two drugs have inhibitory effects on COX1 \ COX2, Astragalus is greater than celecoxib	Shen Hong, Liu Zengwei, Zhang Kun,et al.Effect of Astragalus membranaceus on expression of COX-1, COX-2, VEGF and PGE-2 in gastric cancer cell line SGC7901.Tumor. 194-198(2007).
26	HQi(Astragalus injection)	ICAM1	-1	Application of astragalus can help reduce liver function damage caused by OJ and have a protective effect on liver cells. The mechanism may be related to reducing the expression of TNF-α and ICAM-1 in liver cells.	Zhang Xinyu, Hu Fengai, Li Dezhi.The effects of astragalus injection on expression of TNF-α and ICAM-1 in liver cells of rats with Obstructive Jaundice.Journal of Binzhou Medical University. 18-21(2015).
27	HQi(Scutellaria baicalensis stem and leaf total flavonoids)	ICAM1	-1	SSTF has an anti-AS effect, which may be related to the down-regulation of VCAM-1 and ICAM-1 expression, thereby inhibiting the inflammation of the blood vessel wall	Zhou Xiaohui, Ren Liqun, Gao Xuan, et al. Effects of scutellaria baicalensis stem and leaf total flavonoids on the expression of VCAM-1 and ICAM-1 in hyperlipidemia rabbit aorta .*Chinese Journal of Gerontology*.30, 3124- 3126(2010).
28	HQi(Phenolic derivatives from Radix Astragali)	IL1B(IL1β)	-1	All four compounds exhibited potent inhibitory effects on TNF-α production and TNF-α, COX-2, IL-1β, IL-6 and iNOS mRNA expression at 50 μM.	Chen W, Zhang YY, Wang Z, et al. Phenolic derivatives from Radix Astragali and their anti-inflammatory activities. Nat Prod Commun. 9,1577–1580(2014).
29	HQi(Astragalus	IL1B(IL1	-1	Compared with the control group, the mRNA	Chen Wei, Yu Maohua, Liu Fang.Effects of Astragalus

	Polysaccharides HQi)	β)		expression levels of IL 1β, IL 2, IL 6, IL 12, TNF α, INF γ, Fas, and iNOS in the APS group were significantly down-regulated, IL 4, IL 5, IL 10, TGF β, Bcl 2 , SOD mRNA expression level was significantly increased	Polysaccharides of Gene Expression of Cytokines of NOD Mice's lslets.Fudan University Journal of Medical Sciences. 607-610(2004)
30	HQi(Astragalus polysaccharides)	IL1B(IL1 β)	-1	We also found that the decreased viral replication after APS treatment was associated with reduced mRNA levels of the cytokines IL-1B, IL-6, IL-8 and TNF-α	Zhang P, Liu X, Liu H, et al. Astragalus polysaccharides inhibit avian infectious bronchitis virus infection by regulating viral replication. Microb Pathog. 114,124–128(2018).
31	HQi(Astragalus polysaccharides)	IL1B(IL1 β)	+1	At most time points, the titer of IBV-specific antibodies, lymphocyte proliferation, and IL-1β, IL-2, IL-8, and TNF-α mRNA expression levels were higher in three APS groups than in the vaccine control group, and these increases were dose-dependent.	Zhang P, Wang J, Wang W, et al. Astragalus polysaccharides enhance the immune response to avian infectious bronchitis virus vaccination in chickens. Microb Pathog. 111,81–85(2017).
32	HQi(Astragalus and Baicalein)	IL1B(IL1 β)	-1	The combination group had reduced levels of IL-1β, IL-8, and TNF-α in LPS-induced MSCs, much more than in the other 2 groups. Compared with the other groups, the combination of Astragaloside and Baicalin more efficiently reduced IL-1β, IL-8, and TNF-α levels in the LPS-induced MSCs model, and ERK inhibitor was capable of recovering the inflammatory effect.	Zhu L, Liu YJ, Shen H, Gu PQ, Zhang L. Astragalus and Baicalein Regulate Inflammation of Mesenchymal Stem Cells (MSCs) by the Mitogen-Activated Protein Kinase (MAPK)/ERK Pathway. Med Sci Monit. 23,3209–3216(2017).
33	HQi(Astragalus Polysaccharides)	TP53 （P53）	-1	Astragalus polysaccharide can down-regulate the expression of P53, P65, VEGF protein in PLGC model rats, reduce AI, thereby controlling the progress of PLGC	Xu Jingwen, Wang Nan. Effects of Astragalus Polysaccharides on the Protein Expression of P53,P65,VEGF and AI in Gastric Mucosa of Rats with Precancerous Lesions of Gastric Cancer. China Pharmacy. 027,3069-3071(2016).
34	HQi(Astragalus Decoction (AE))	BCL2	+1	To study the effect and mechanism of Astragalus Decoction (AE) on renal tubular cell apoptosis in rats	Zhou Kan, Lu Dongning, Tao Zhihu.Effect of Astragalus Decoction on Apoptosis of Renal Tubular Cells in Rats with IR Injury and Its

				with renal ischemia-reperfusion injury. The expression of Bax and Fas in the AE pretreatment group decreased significantly (P <0.05), while the expression of Bcl-2 increased significantly (P <0.05), so Bax / Bcl-2 decreased (P <0.05)	Mechanism Analysis.Guangxi Journal of Traditional Chinese Medicine. 42,39-41(2019).
35	HQi(Astragalus)	BCL2	-1	Astragalus can promote the apoptosis of leukemia cells and improve the efficacy of patients. The mechanism may be by reducing the expression of Bcl-2 and BCL-xl and increasing the expression of Bax and Bak.	Yu Dan, Cheng Hui, Yi Xue, et al.The Influence of Astragalus on BCL-2's family in Eld patients with acute myeloid leukemia.Harbin Medical Journal. 36,501-503(2016).
36	HQi(Astragalus polysaccharide)	TGFB1(TGFβ1)	-1	this increase in tissue factor (TF), transforming growth factor (TGF-β), and interleukin-8 (IL-8) could be inhibited by the addition of APS. The immune response mediated by Toll-like receptor 4 (TLR4) in ECs may be unregulated by CSFV as it was also inhibited by pre-treatment with APS.	Zhuge Z, Dong Y, Li L, et al. Effects of astragalus polysaccharide on the adhesion-related immune response of endothelial cells stimulated with CSFV in vitro. PeerJ . 5,e3862(2017).
37	HQi(Astragalus polysaccharide (APS))	TGFB1(TGFβ2)	-1	Astragalus polysaccharide (APS) significantly reduced fasting blood glucose, blood creatinine, urea nitrogen, urine KIM-1, and urine OPN concentration in DM rats, and inhibited the activity of renal TGF-β1 / Smads signaling pathway. Conclusion APS has renal protective effect on DM rats, and its mechanism may be related to the inhibition of TGF-β1 / Smad signaling pathway in DM rat kidney.	Li Chengde, Wang Yu, Qu Jingrong,et al.Effects of astragalus polysaccharide on renal TGF-β1/Smads signaling pathway in rats with diabetes mellitus.Chinese Pharmacological Bulletin. 34,512-516(2018).
38	HQi(Astragalus polysaccharides)	MAPK1	+1	Astragalus polysaccharides can increase the activity of ERK and Akt after ischemia-reperfusion injury in human cardiomyocytes and reduce apoptosis	Fan Zongjing, Xie Liandi, Cui Jie, et al.Study on the expression of ERK and Akt on HCMEC of myocardial ischemia-reperfusion injury and the effects of astragalus polysaccharides.Global

					Traditional Chinese Medicine.11,207-210(2018).
39	HQi(Astragaloside IV)	CXCL8（IL8）	-1	Astragaloside IV can protect the gastric mucosal Injury Induced by clopidogrel by reducing the levels of serum inflammatory factors IL-6, IL-8 and TNF-α	Zhang Jing, Wang Yanrong, Zhang Qiuzhan.Effects of Astragaloside IV on Serum IL-6, IL-8 and TNF-α in Rats with Gastric Mucosal Injury Induced by Clopidogrel.Journal of Hebei North University(Natural Science Edition). 34,12-14,18(2018).
40	HQi(Astragalus mongholicus injection)	CRP	-1	Before treatment, there were no statistically significant differences in cTnI, hs-CRP, CK-MB, miR-146b, miR-155, IL-10, TNF-β, IL-17, IL-21, etc. Serum cTnI, hs-CRP, CK-MB and other myocardial injury indicators were lower than before treatment, and the observation group was significantly lower than the control group	Qi Guibin, Gao Jianbu.Effect of Astragalus Injection on miR and Treg / Th17 Cytokines in Patients with Viral Myocarditis.Journal of Chinese Medicinal Materials. 42,924-927(2019).
41	HQi(Huangqi glycoprotein)	CCL2	-1	HQGP treatment also reduced the expression of chemokines such as CCL2 and CCL5 and the production of tumor necrosis factor α (TNF-α), interleukin (IL)-1β, IL-6, but increased the level of IL-10.	Xing Y, Liu B, Zhao Y, et al. Immunomodulatory and neuroprotective mechanisms of Huangqi glycoprotein treatment in experimental autoimmune encephalomyelitis. Folia Neuropathol. 57,117–128(2019).
42	HQi(Astragalus)	CCL2	-1	In brain, chemokines such as CCL2 and CCL5 were inhibited in HQGP-treated EAE compared with control mice.	Zhang H, Guo M, Zhang L, et al. Folia Neuropathol. 55,308–316(2017).
43	HQi(Astragalus mongholicus injection)	CCL2	-1	Astragalus injection can significantly reduce the expression level of ccl2mrna and the activity of ccl2 protein in rat serum and lung tissue	Wang Jun. The Preventive and therapeutic effect of astragalus mongholicus injection on acute radiation-induced lung and its mechanism [D].Hubei University Of Traditional Chinese Medicine.(2015).
44	HQi(Astragalus polysaccharide)	MAPK8(JNK)	-1	Astragalus polysaccharides (APS) alleviate LPS-induced cardiomyocyte apoptosis by inhibiting	Han Lin, Wang Hongxin, Lu Meili.Effect of Astragalus polysaccharide on LPS-induced cardiomyocyte apoptosis by

				NF-κB and JNK signaling pathways.	inhibiting NF-κB and JNK signaling pathway.Chinese Pharmacological Bulletin.34,243-249(2018).
45	HQi(Astragalus polysaccharide)	FOS(C-FOS)	+1	After treatment with APS, the number of hippocampal neuronal apoptosis in rats with traumatic brain injury was significantly reduced, the expression of c-fos protein and genes in brain tissue was up-regulated, and the content of NO decreased (P <0.01).	Wu Dan, Zhao Yanhe, Zhang Haibo,et al.Effects of Astragalus Polysaccharide on hippocampal neuronal apoptosis and cfos and NO contents in brain tissue of rats with brain injury.Progress of Anatomical Sciences.25,579-582(2019).
46	HQi(Astragalus polysaccharide)	FOS(C-FOS)	-1	APS intervention significantly inhibited the activity of NF-κB and MAPK signaling pathways in rat hippocampus (all P <0.05), and reduced the levels of p-c-Fos and p-c-Jun (all P <0.05)	Li Chengde. The antidepressant effects and the mechanism of Astragalus polysaccharide in rat models of depression induced by chronic unpredictable mild stress and lipopolysaccharide[D].Shandong University Of Traditional Chinese Medicine.(2018).
47	HQi(Astragalus)	FOS(C-FOS)	+1	After astragalus intervention, the blood sugar decreased slightly during acute hypoglycemia, which had no effect on the basic blood glucose. Epinephrine and Cortisol in the Astragalus group were significantly higher than those in the control group. The expression of c-fos in PVN of Astragalus group was significantly higher than that of control group.	Mao Dandan, Chen Gang, Lu Lingyun,et al.Effective component of Radix Astragali on impaired retroegulation of endocrine in rat models induced by hypoglycemia.Shanghai Journal of Traditional Chinese Medicine. 46,64-66(2012).
48	HQi(Astragalus polysaccharide)	FOS(C-FOS)	-1	Stress control group (B), intraperitoneal injection of 0.5ml saline per day after amputation; stress + APS high (C), medium (D), low (E) dose group, APS1000mgkg, 500mgkg, 250mgkg, respectively The saline was diluted to 0.5ml intraperitoneally and injected once a day. Compared with group B, the expressions of NFκBmRNA and IL10mRNA in thymus	Zeng Guangxian, Liu Junying, Xiong Jinrong, et al.Study on effect of Astragalus polysaccharide for traumatic stress mice cell immunity.Chinese Journal of Microbiology and Immunology. 20-23(2004).

				and spleen tissues of mice in groups C, D and E were suppressed (P <0.01); CD4 + antigen level and CD4 + CD8 + ratio in thymus and spleen tissues were increased (P <0.01); C-fos antigen levels in thymus and spleen tissue decreased (P <0.01)	
49	HQi(astragalosides)	FOS(C-FOS)	-1	astragalosides can reduce the content of smooth muscle in atherosclerotic plaques, and its mechanism may be related to the reduction of Ras, c-jun, c-fos, c-myc expression.	Li Jun'an, Zhang Guoyuan, fan quming, et al.Effects of astragalosides on smooth muscle in rabbit atherosclerotic plaques.Pharmacology and Clinics of Chinese Materia Medica. 25,25-27(2009).
50	HQi(Astragalus）	IL4	-1	Astragalus intervention mice compared with each group of asthma model mice IL-2 content in alveolar lavage fluid increased, IL-4 content was significantly reduced (P <0.01); Astragalus + hormone intervention group compared with simple hormone group, IL-2 Increased content, decreased IL-4 (P <0.01)	Zhang Ya, Yang Lin, Jiang Rongyan, et al..Effect of Astragalus on IL-2, IL-4 and behavioral changes in bronchoalveolar lavage fluid of asthmatic mice.Journal of Taishan Medical College.40,914-916(2019).
51	HQi(Selenium-enriched Astragalus extract）	IL4	+1	Compared with the model group, the levels of IL-2, IL-4, and IFN-γ in each intervention group were increased, and the content of TNF-α was decreased, the difference was statistically significant (P <0.05)	Li Qin, fan Qiang, Hu Jihong, et al.Effects of Selenium-enriched Astragalus extract on immunosuppressed cytokines IL-2, IL-4, IFN-γ and TNF-α Effects of γ and TNF-α.Western Journal of Traditional Chinese Medicine. 31,22-25(2018）.
52	HQi	IL1A(IL-1α)			
53	HQi(Astragaloside IV）	STAT1	-1	Astragaloside IV (ASI) can inhibit IFN-γ-induced microglial activation, and its mechanism is to inhibit the activation of STAT1 / IκB / NF-κB signaling pathway, and reduce the IL-1β, TNF- Gene expression of α and i NOS is related to the reduction of	He Yixin, Shi Hailian, Liu hongshuai,et al.Astragaloside IV regulates STAT1 / IκB / NF-κB signaling pathway to inhibit activation of BV-2 cells.China Journal of Chinese Materia Medica.40,124-128（2015）.

				NO and TNF-α production.	
54	HQi(Astragalus Polysaccharide）	EGFR	-1	The research results showed that APS can effectively inhibit the growth and metastasis of Lewis lung cancer in mice, improve immune organ function, inhibit the protein expression of VEGF and EGFR in tumor tissues, and have a concentration-effect relationships.	Zhao L, Zhong Y, Liang J, et al. Effect of Astragalus Polysaccharide on the Expression of VEGF and EGFR in Mice with Lewis Transplantable Lung Cancer. J Coll Physicians Surg Pak. 29, 392–394(2019).
55	HQi(Astragaloside IV）	EGFR	+1	All eight astragalosides studied enhanced epidermal growth factor receptor (EGFR) activity in HaCaT cells. Among them, astragaloside VI (AS-VI) showed the strongest EGFR activation.	Lee SY, Chang WL, Li ZX, et al. Astragaloside VI and cycloastragenol-6-O-beta-D-glucoside promote wound healing in vitro and in vivo. Phytomedicine. 38,183–191(2018).
56	HQi(Astragaloside IV）	EGFR	-1	Astragaloside IV can significantly down-regulate the phosphorylation levels of EGFR and Akt, and the expression levels of EGFR and Akt total protein remain unchanged	Li Xiao, Zhou Yanyan, song Xiaojie,et al.Effect of Astragaloside IV on Radiosensitivity of Cervical Cancer Cell Lines and Related Mechanisms.Chinese Journal of Basic Medicine in Traditional Chinese Medicine.24,1540-1543(2018).
57	HQi(Astragalus Polysaccharide）	CXCL10	+1	mRNA of iNOS (Figure 3A), IL-6 (Figure 3B), TNF-a (Figure 3D), and C-X-C motif chemokine 10 (CXCL10) (Figure 3E) were significantly induced in the Astragalus Polysaccharide(RAP)and LPS treatment group compared with the control group.	Wei Wei,Zhi-Peng Li,Zhao-Xiang Bian,et al. Astragalus Polysaccharide RAP Induces Macrophage Phenotype Polarization to M1 via the Notch Signaling Pathway. Molecules. 24,(2019).
58	HQi(Astragalus polysaccharide (APS) ）	SOD1	+1	Compared with the control group, the Astragalus polysaccharide (APS) group IL 1β, IL 2, IL 6, IL 12, TNF α, INF γ, Fas, iNOS mRNA expression levels were significantly reduced, IL 4, IL 5, IL 10, TGF β, Bcl 2, SOD mRNA expression levels were significantly up-regulated	Chen Wei, Yu Maohua, Liu Fang.Effects of Astragalus Polysaccharides of Gene Expression of Cytokines of NOD Mice's lslets.Fudan University Journal of Medical Sciences. 607-610(2004).
59	HQi(Astragalus	SOD1	-1	Astragalus polysaccharide treatment and prophylactic	Lu Chunhua, Ma Yanmei, Wang Hongxia,et al.Protective effect and

	polysaccharide）			administration can reduce lung index and reduce lung injury; down-regulate lung MDA level and up-regulate SOD, GSH-Px activity (P <0.05); inhibit TNF-α, IFN-α, IL-6, IL -1β secretion (P <0.05); reduce the expression of Caspase-3, 8, 9 (P <0.05)	mechanism of Astragalus polysaccharide treatment and prophylactic administration on PR8 infected mice.Chinese Journal of Immunology. 35,1699-1702+1707(2019).
60	HQi(Astragalus injection）	PARP1	-1	After 20 g / L Astragalus injection for 0, 6, 12, and 24 hours, the expression of cleared caspase-3 and cleared caspase-9 in human cervical immortalized epithelial cells H8 gradually increased, and the difference was significant (P <0.05)); The expression of Cleared PARP protein gradually decreased, and the difference was significant (P <0.05)	Lu Ling, Xiao Chenguang, Liu Qing, et al.Effects of astragalus injection on human immortalized cervical epithelial cell apoptosis in vitro.Chinese Journal of Tissue Engineering Research.20,743-747(2016).
61	HQi	CASP8	/		
62	HQi(the Astragalus polysaccharide (APS) ）	IFNG(IFN -γ)	-1	Compared with the control group, the Astragalus polysaccharide (APS) group IL 1β, IL 2, IL 6, IL 12, TNF α, INF γ, Fas, iNOS mRNA expression levels were significantly reduced, IL 4, IL 5, IL 10, TGF β, Bcl 2, SOD mRNA expression levels were significantly up-regulated	Chen Wei, Yu Maohua, Liu Fang.Effects of Astragalus Polysaccharides of Gene Expression of Cytokines of NOD Mice's lslets.Fudan University Journal of Medical Sciences. 607-610(2004).
63	HQi(Cisplatin combined with astragaloside IV）	IFNG(IFN -γ)	+1	Compared with the control group, the mRNA expression levels of IL 1 β, IL 2, IL 6, IL 12, TNF α, INF-γ, Fas, iNOS in APS group were significantly down regulated, while the mRNA expression levels of IL 4, IL 5, IL 10, TGF β, BCL 2, SOD were significantly up regulated	Liu Tiegang, Wen Chunyan, Shen Xuansan.Effects of cisplatin combined with astragaloside on inflammatory factors and immune function in breast cancer rats.Chinese Journal of Gerontology. 40,863-865(2020).
64	HQi(Radix	IL2	-1	Radix Astragali significantly attenuated elevated levels	Zhao P, Su G, Xiao X, et al. Chinese medicinal herb Radix Astragali

	Astragali）			of the Th1 cytokines (IFN-gamma and IL-2), and increased the Th2 cytokines (IL-4 and IL-10) in autoimmune myocarditis.	suppresses cardiac contractile dysfunction and inflammation in a rat model of autoimmune myocarditis. Toxicol Lett. 182,29–35(2008).
65	HQi(Astragalus polysaccharide (APS) ）	IL2	-1	Compared with the control group, the Astragalus polysaccharide (APS) group IL 1β, IL 2, IL 6, IL 12, TNF α, INF γ, Fas, iNOS mRNA expression levels were significantly reduced, IL 4, IL 5, IL 10, TGF β, Bcl 2, SOD mRNA expression levels were significantly up-regulated	Chen Wei, Yu Maohua, Liu Fang.Effects of Astragalus Polysaccharides of Gene Expression of Cytokines of NOD Mice's lslets.Fudan University Journal of Medical Sciences. 607-610(2004).
66	HQi(Astragalus polysaccharide）	IL2	+1	Yupingfeng polysaccharides and Astragalus polysaccharides enhanced SIgA, IL-2, TGF-β1 and IL-6 levels significantly (P<0.05 or P<0.01)	Deng Hua,Yang Hong,Jiang Yan-ping,et al.Regulatory Effects of Yupingfeng Polysaccharides on Intestinal Mucosal Immune Response and Immune Injury in Mice.Chinese Journal of Veterinary Drug. 52,43-48(2018).
67	HQi(Cisplatin combined with astragaloside IV）	IL2	+1	Cisplatin combined with astragaloside IV is superior to cisplatin alone in the treatment of breast cancer in rats ~ + Level, down-regulate IL-1, IL-6, TNF-α, CD8 ~ + level	Liu Tiegang, Wen Chunyan, Shen Xuansan.Effects of cisplatin combined with astragaloside on inflammatory factors and immune function in breast cancer rats.Chinese Journal of Gerontology. 40,863-865(2020).
68	HQi(Astragalus ）	IL2	+1	Astragalus intervention mice compared with each group of asthma model mice IL-2 content in alveolar lavage fluid increased, IL-4 content was significantly reduced (P <0.01); Astragalus + hormone intervention group compared with simple hormone group, IL-2 Increased content, decreased IL-4 (P <0.01)	Zhang Ya,Yamg Lin,JIANG Rong-yan,et al.The effect of Astragalus membranaceus on the changes of IL 2, IL 4 and their behavior in BALF of asthmatic mice.Journal of Taishan Medical College. 40,914-916(2019).
69	HQi(Radix Astragali）	IL2	-1	Radix Astragali significantly attenuated elevated levels of the Th1 cytokines (IFN-gamma and IL-2), and increased the Th2 cytokines (IL-4 and IL-10) in	Zhao P, Su G, Xiao X,et al. Chinese medicinal herb Radix Astragali suppresses cardiac contractile dysfunction and inflammation in a rat model of autoimmune myocarditis. Toxicol Lett. 182,29–35(2008).

				autoimmune myocarditis.	
70	HQi(Astragalus polysaccharides）	IL2	+1	At most time points, the titer of IBV-specific antibodies, lymphocyte proliferation, and IL-1β, IL-2, IL-8, and TNF-α mRNA expression levels were higher in three APS groups than in the vaccine control group, and these increases were dose-dependent.	Zhao P, Su G, Xiao X,et al. Chinese medicinal herb Radix Astragali suppresses cardiac contractile dysfunction and inflammation in a rat model of autoimmune myocarditis. Toxicol Lett. 182,29–35(2008).
71	HQi(Fufang Huangqi Yiqi oral liquid）	CAT	+1	The content of GSH-Px, SOD and CAT in liver tissue increased significantly, and the amount of MDA production decreased significantly (P <0.05)	Yan Wenrui, Wang Lingzhi, Hou Lingling, et al.Protective effect of Fufang Huangqi Yiqi oral liquid against acute liver injury induced by AFB1 in rats.Journal of Shanxi Medical University. 50,1435-1439(2019).
72	HQi(Astragalus polysaccharide）	IL10	-1	Compared with group B (stress group), the expression of NFκBmRNA and IL10mRNA in thymus and spleen tissues of mice in groups C, D and E (stress + astragalus polysaccharide group) was suppressed (P <0.01)	Zeng Guangxian, Liu Junying, Xiong Jinrong, et al.Study on effect of Astragalus polysaccharide for traumatic stress mice cell immunity.Chinese Journal of Microbiology and Immunology.20-23(2004).
73	HQi(Astragalus polysaccharide）	IL10	+1	Compared with the control group, the Astragalus polysaccharide (APS) group IL 1β, IL 2, IL 6, IL 12, TNF α, INF γ, Fas, iNOS mRNA expression levels were significantly reduced, IL 4, IL 5, IL 10, TGF β, Bcl 2, SOD mRNA expression levels were significantly up-regulated	Chen Wei, Yu Maohua, Liu Fang.Effects of Astragalus Polysaccharides of Gene Expression of Cytokines of NOD Mice's lslets.Fudan University Journal of Medical Sciences. 607-610(2004).
74	HQi(Radix Astragali）	IL10	+1	Radix Astragali significantly attenuated elevated levels of the Th1 cytokines (IFN-gamma and IL-2), and increased the Th2 cytokines (IL-4 and IL-10) in autoimmune myocarditis.	Zhao P, Su G, Xiao X, et al. Chinese medicinal herb Radix Astragali suppresses cardiac contractile dysfunction and inflammation in a rat model of autoimmune myocarditis. Toxicol Lett. 182,29–35(2008).
75	HQi(Astragalus	IL10	+1	Astragalus injection can reduce the expression of	Li Qiang, Qin Yi, Du Qinchuan,et al.Effect of Astragalus Injection

	injection）			TNF-α around the bleeding focus and increase the expression of IL-10 around the bleeding focus	on the expression of TNF-a and IL-10 around the focal cerebral hemorrhage in rats.Ningxia Medical Journal. 33,105-107+92(2011).
76	HQi	HMOX1	+1		
77	HQi(Astragalus Decoction (AE)）	BAX	-1	To study the effect and mechanism of Astragalus Decoction (AE) on renal tubular cell apoptosis in rats with renal ischemia-reperfusion injury. The expression of Bax and Fas in the AE pretreatment group decreased significantly (P <0.05), while the expression of Bcl-2 increased significantly (P <0.05), so Bax / Bcl-2 decreased (P <0.05)	Zhan Kan,LV Dongning, Tao Zhihu.Effect of Astragalus Decoction on Apoptosis of Renal Tubular Cells in Rats with IR Injury and Its Mechanism Analysis.Guangxi Journal of Traditional Chinese Medicine. 42,39-41(2019).
78	HQin(Flavonoids: from the extract of Scutellariae)	PTGS2	-1	The expression level of Cox-2 was shown in similar pattern with that of NFκB, and the inhibitory effect was in a dose-dependent manner	Gong G, Wang H, Kong X, et al. Flavonoids are identified from the extract of Scutellariae Radix to suppress inflammatory-induced angiogenic responses in cultured RAW 264.7 macrophages. Sci Rep. 8,17412(2018).
79	HQin(Wogonin, a plant flavone from Scutellaria radix)	PTGS2	-1	wogonin at the doses of 250-1000 microg/ear/3 days potently lowered mRNA levels of COX-2 and tumor necrosis factor-alpha with less effect on intercellular adhesion molecule-1 and interleukin-1beta in a sub-chronic skin inflammation model of tetradecanoylphorbol-13-acetate-induced ear edema (multiple treatment).	Chi YS, Lim H, Park H, et al. Effects of wogonin, a plant flavone from Scutellaria radix, on skin inflammation: in vivo regulation of inflammation-associated gene expression. Biochem Pharmacol. 66,1271–1278(2003).
80	HQin(Baicalein)	PTGS2	-1	Baicalein can significantly inhibit the production of inflammatory mediators NO and PGE2 and the release of inflammatory factors TNF-α and IL-6, and significantly reduce the expression levels of iNOS and COX-2	Zhang Qian, Li Hui-Xiang, Liu Pan,et al.In vitro Anti-inflammatory and Antioxidative Activity of Baicalein.Journal of Yantai University(Natural Science and Engineering Edition).31,232-238(2018).

81	HQin(Flavonoids: from the extract of Scutellariae)	iNOS	-1	The expression of LPS-induced iNOS was decreased under application of various concentrations of SR herbal extract, as compared to the control	Gong G, Wang H, Kong X, et al. Flavonoids are identified from the extract of Scutellariae Radix to suppress inflammatory-induced angiogenic responses in cultured RAW 264.7 macrophages. Sci Rep. 8,17412(2018).
82	HQin(Baicalein)	iNOS	-1	Baicalein can significantly inhibit the production of inflammatory mediators NO and PGE2 and the release of inflammatory factors TNF-α and IL-6, and significantly reduce the expression levels of iNOS and COX-2	Zhang Qian, Li Hui-Xiang, Liu Pan,et al.In vitro Anti-inflammatory and Antioxidative Activity of Baicalein.Journal of Yantai University(Natural Science and Engineering Edition).31,232-238(2018).
83	HQin(Heat-Processed Scutellariae Radix)	RELA(P65)	-1	The augmented expressions of hepatic oxidative stress and inflammation-related proteins, phospho-p38, phosphorylated extracellular signal-regulated kinase, phosphorylated c-Jun N-terminal kinase, nuclear factor-[Formula: see text] B p65, activator protein-1, cyclooxygenase-2, inducible nitric oxide synthase, MCP-1, intercellular adhesion molecule-1, tumor necrosis factor-[Formula: see text], and IL-6, were downregulated by the heat-processed Scutellariae Radix.	Park CH, Shin MR, An BK, et al. Heat-Processed Scutellariae Radix Protects Hepatic Inflammation through the Amelioration of Oxidative Stress in Lipopolysaccharide-Induced Mice. Am J Chin Med. 45,1233–1252(2017).
84	HQin(Baicalein)	RELA(P65)	-1	Moreover, baicalein significantly inhibited reactive oxygen species (ROS) production, decreased cyclooxygenase-2 (COX-2) and nuclear factor-b (NF-κB)/p65 expression	Yan Jiao-Jiao,Du Guan-Hua,Qin Xue-Mei, et al. Baicalein attenuates the neuroinflammation in LPS-activated BV-2 microglial cells through suppression of pro-inflammatory cytokines, COX2/NF-κB expressions and regulation of metabolic abnormality. .Int. Immunopharmacol. 79, 106092(2020).
85	HQin(Heat-Processed	RELA(P65)	-1	The augmented expressions of hepatic oxidative stress and inflammation-related proteins, phospho-p38,	Park CH, Shin MR, An BK, et al. Heat-Processed Scutellariae Radix Protects Hepatic Inflammation through the Amelioration of

	Scutellariae Radix)			phosphorylated extracellular signal-regulated kinase, phosphorylated c-Jun N-terminal kinase, nuclear factor-[Formula: see text] B p65, activator protein-1, cyclooxygenase-2, inducible nitric oxide synthase, MCP-1, intercellular adhesion molecule-1, tumor necrosis factor-[Formula: see text], and IL-6, were downregulated by the heat-processed Scutellariae Radix.	Oxidative Stress in Lipopolysaccharide-Induced Mice. Am J Chin Med. 45,1233–1252(2017).
86	HQin(Baicalein)	TNF	-1	Baicalin may play a protective role in pancreatic tissue by reducing the production and release of TNF-α and IL-6, and down-regulating TNF-α / IL-10.	Li Huiyan, Zhao Shuguang, Zhao Baomin, et al. Effects of baicalin on TNF-α, IL-6 and IL-10 in rats with severe acute pancreatitis. Medical Journal of National Defending Forces in Southwest China. 32-35(2009).
87	HQin(Baicalin and Baicalin nanoliposomes)	TNF	-1	After treatment with BA and BA-NL, the positive expression of macrophages and neutrophils in the liver of the mice was significantly reduced compared with the MCD group. The expression of m RNA of molecules (ICAM, ECAM, ELAM) and chemokines (CCL2, CXCL2) was significantly reduced, and the expression of TLR4, NF-κB, P-P65, P-P38 protein in liver tissue decreased	Yuan Yinglin. Baicalin nanoliposomes ameliorated non-alcoholic fatty liver disease by suppression TLR4 signaling pathway in mice[D].Chongqing Medical University.(2017).
88	HQin(Scutellaria baicalensis)	TNF	+1	In HMC-1 cells, SB restored IL-8 and TNF-α expression and inhibited MAP kinase expression in compound 48/80-induced HMC-1 cells. These data suggest that SB may prove to be a useful anti-inflammatory agent through its downregulation of the expression of various inflammatory mediators.	Jung HS, Kim MH, Gwak NG, et al. Antiallergic effects of Scutellaria baicalensis on inflammation in vivo and in vitro. J Ethnopharmacol. 141,345–349(2012).

89	HQin(Scutellaria baicalensis water Extraction)	IL6	-1	Compared with the model group, the expression of IL-6 mRNA in the jejunum of mice in each dose group of Scutellaria baicalensis water Extraction decreased significantly (P <0.01)	Liu Xiaoxi, Dong Jie, Li Minxia,et al.Effect of the Scutellaria baicalensis Water Extraction on the Regulation of Jejunum Damage and Repair in the Model of Enteritis Mice[J/OL].Chinese Journal of Animal and Veterinary Sciences. 392-398（2020).
90	HQin(Scutellaria)	IL6	-1	Scutellaria can significantly reduce the content of serum TNF-α, IL-1, IL-6 on the third day after infection, and can increase the content of IFN-γ in each phase after infection, and increase IL-10 on the first 1 to 5 days after infection content	Li Yali, Xu Hongri, Cao Hongyun,et al.Study on the Mechanism of Anti-influenza of Five Kinds of Heat-clearing and Detoxifying Drugs from the Perspective of Immunoinflammatory Injury and Its Clinical Significance.Journal of Emergency in Traditional Chinese Medicine.29,189-192+205(2020).
91	HQin(Flavonoids :from the extract of Scutellariae Radix)	IL6	-1	The mRNAs encoding IL-1β, IL-6 and TNF-α were restrained upon the SR treatment. The SR herbal extract (1 mg/mL) showed the strongest inhibition, i.e.~50% for IL-1β, ~60% for IL-6 and ~60% for TNF-α	Gong G, Wang H, Kong X, et al. Flavonoids are identified from the extract of Scutellariae Radix to suppress inflammatory-induced angiogenic responses in cultured RAW 264.7 macrophages. Sci Rep. 8,17412(2018).
92	HQin(Baicalein)	IL6	-1	Baicalein can significantly inhibit the production of inflammatory mediators NO and PGE2 and the release of inflammatory factors TNF-α and IL-6, and significantly reduce the expression levels of iNOS and COX-2	Zhang Qian, Li Hui-Xiang, Liu Pan,et al.In vitro Anti-inflammatory and Antioxidative Activity of Baicalein.Journal of Yantai University(Natural Science and Engineering Edition).31,232-238(2018).
93	HQin(Heat-Processed Scutellariae Radix)	IL6	-1	The augmented expressions of hepatic oxidative stress and inflammation-related proteins, phospho-p38, phosphorylated extracellular signal-regulated kinase, phosphorylated c-Jun N-terminal kinase, nuclear factor-[Formula: see text] B p65, activator protein-1, cyclooxygenase-2, inducible nitric oxide synthase, MCP-1, intercellular adhesion molecule-1, tumor	Park CH, Shin MR, An BK, et al. Heat-Processed Scutellariae Radix Protects Hepatic Inflammation through the Amelioration of Oxidative Stress in Lipopolysaccharide-Induced Mice. Am J Chin Med. 45,1233–1252(2017).

				necrosis factor-[Formula: see text], and IL-6, were downregulated by the heat-processed Scutellariae Radix.	
94	HQin(Baicalin and Baicalin nanoliposomes)	IL6	-1	After treatment with BA and BA-NL, the positive expression of macrophages and neutrophils in the liver of the mice was significantly reduced compared with the MCD group. The expression of m RNA of molecules (ICAM, ECAM, ELAM) and chemokines (CCL2, CXCL2) was significantly reduced, and the expression of TLR4, NF-κB, P-P65, P-P38 protein in liver tissue decreased	Yuan Yinglin. Baicalin nanoliposomes ameliorated non-alcoholic fatty liver disease by suppression TLR4 signaling pathway in mice[D].Chongqing Medical University. (2017).
95	HQin(Scutellaria)	MAPK14（P38α）	-1	It is speculated that the anti-inflammatory substances in Scutellaria baicalensis reduce the release of pro-inflammatory cytokines such as IL-β and TNF-α by inhibiting the MAPK14 gene, thereby exerting an anti-inflammatory effect	1）Kong T T, Zhang C M, Liu Z P. Recent developments of p38α MAP kinase inhibitors as antiinflammatory agents based on the imidazole scaffolds . Curr Med Chem. 20, 1997-2016(2013).<br>2）Liu Yu. Study on the Analgesia,Healing and anti-inflammatory Mechanisms of Simiaojunyi ointment based on the Neuroendocrine-immunoregulatory Network [D]. Changsha:Hunan University Of Chinese Medicine. (2016).
96	HQin(Baicalein)	CASP3	-1	Baicalein can inhibit the oxidative stress response of adrenal epithelial cells, and by down-regulating the level of phosphorylated Caspase-3 protein, thereby inhibiting apoptosis and reducing cell damage	Guo Yaju, Zhang Yongjun, Song Shuxian.Effect of baicalein on H2O2-induced apoptosis of renal tubular epithelial cells.Journal of Clinical and Experimental Medicine. 17,2053-2056(2018).
97	HQin(Baicalein)	CASP3	+1	caspase-3 caspase-9 increased expression in vivo and in vitro after baicalin treatment	Baicalin induces apoptosis of gallbladder carcinoma cells in vitro via a mitochondrial-mediated pathway and suppresses tumor growth in vivo.
98	HQin(wogonin,	PTGS1(C	+1	When applied topically on the intact skin, only a high	Chi YS, Lim H, Park H, et al. Effects of wogonin, a plant flavone

	a plant flavone from Scutellaria radix)	OX1)		dose treatment of wogonin (1000 microg/ear/3 days) slightly increased COX-1 and fibronectin mRNA.	from Scutellaria radix, on skin inflammation: in vivo regulation of inflammation-associated gene expression. Biochem Pharmacol. 66,1271–1278(2003).
99	HQin(Baicalin and Baicalin nanoliposomes)	ICAM1	-1	After treatment with BA and BA-NL, the positive expression of macrophages and neutrophils in the liver of the mice was significantly reduced compared with the MCD group. The expression of m RNA of molecules (ICAM, ECAM, ELAM) and chemokines (CCL2, CXCL2) was significantly reduced, and the expression of TLR4, NF-κB, P-P65, P-P38 protein in liver tissue decreased	Yuan Yinglin. Baicalin nanoliposomes ameliorated non-alcoholic fatty liver disease by suppression TLR4 signaling pathway in mice[D].Chongqing Medical University. (2017).
100	HQin(Huangqin Decoction)	IL1B(IL1β)	+1	The results of the research on the mechanism of Huangqin Decoction's effective part formula in the treatment of UC rats showed that the content of IL-1β in the peripheral blood of UC rats was significantly increased, further confirming the important role of IL-1β in the development of ulcerative colitis	Ding Xiaogang. Experimental Study on Anti-ulcerative Colitis in Rats Treated by Effective Components of Huangqin Decoction[D].Beijing University of Chinese Medicine.(2003).
101	HQin(Flavonoids :from the extract of Scutellariae Radix)	IL1B(IL1β)	-1	The mRNAs encoding IL-1β, IL-6 and TNF-α were restrained upon the SR treatment. The SR herbal extract (1 mg/mL) showed the strongest inhibition, i.e.~50% for IL-1β, ~60% for IL-6 and ~60% for TNF-α	Gong G, Wang H, Kong X, et al. Flavonoids are identified from the extract of Scutellariae Radix to suppress inflammatory-induced angiogenic responses in cultured RAW 264.7 macrophages. Sci Rep. 8, 17412(2018).
102	HQin(Extracts From Flos lonicerae in Combination	IL1B(IL1β)	-1	There were increases (P < 0.05) in the relative expression of ileal IFNG, TNFA, IL8, and IL1B at 3 DPI of layers in response to S. pullorum challenge (Figure 1). However, they were all decreased (P < 0.05)	Wang W, Jia H., Zhang H., et al. Supplemental Plant Extracts From Flos lonicerae in Combination With Baikal skullcap Attenuate Intestinal Disruption and Modulate Gut Microbiota in Laying Hens Challenged by Salmonella pullorum. Frontiers in Microbiology, 10.

	With Baikal skullcap)			in layers of T group when compared with those in PC group. Furthermore, an up-regulation ($P < 0.05$) in the relative expression of ileal IL10 at 3 DPI was observed in the birds from T group relative to PC group.	
103	HQin(Baicalin and Baicalin nanoliposomes)	IL1B(IL1β)	-1	After treatment with BA and BA-NL, the positive expression of macrophages and neutrophils in the liver of the mice was significantly reduced compared with the MCD group. The expression of m RNA of molecules (ICAM, ECAM, ELAM) and chemokines (CCL2, CXCL2) was significantly reduced, and the expression of TLR4, NF-κB, P-P65, P-P38 protein in liver tissue decreased	Yuan Yinglin. Baicalin nanoliposomes ameliorated non-alcoholic fatty liver disease by suppression TLR4 signaling pathway in mice[D].Chongqing Medical University. (2017).
104	HQin(Baicalin)	TP53（P53）	-1	Baicalin has a strong effect on inducing apoptosis in human liver cancer HepG-2 cells, and its mechanism may be related to the down-regulation of P53 gene expression and the reduction of Bcl-2 / Bax ratio	Meng Lu, Zhang Xuewu, Li Zhenglu. Experimental Study on Anti-ulcerative Colitis in Rats Treated by Effective Components of Huangqin Decoction. Lishizhen Medicine and Materia Medica Research.21,2212-2213(2010).
105	HQin(Baicalein)	BCL2	+1	In the SAP + baicalein group, the levels of serum amylase, IL-6, TNF-α, MDA content in the lung tissue and apoptosis index decreased, SOD activity increased, Bax and p-p38MAPK in the lung tissue of the rats in the SAP and baicalein group Decreased protein expression, increased Bcl-2 protein expression ($P < 0.05$)	Zhu Xiaolin, Zhao Haiyan, Yang Jilin.Effects of baicalein on lung tissue injury in rats with severe acute pancreatitis[J/OL].Journal of Zhengzhou University(Medical Sciences). 37-40(2020).
106	HQin(Baicalin)	BCL2	+1	The protective effect of baicalin on SH-SYSY cell damage induced by H2O2 may be related to baicalin up-regulating the expression of Bcl-2 and Bcl-xL to	Yan Ming, Li Hongzhi, Liu Jieting, et al.Effects of Baicalin on Bcl-2 and Bcl-xL mRNA Expression Against SH-SY5Y Cells Apoptosis.Herald of Medicine. 31,843-845(2012).

				play an anti-apoptotic role.	
107	HQin(Baicalein)	BCL2	-1	After 20, 40 and 80 μmol / L baicalein treatment for 24 h, the expression of pro-apoptotic protein Bax in ovarian cancer HO-8910 cells was significantly increased, while the expression of anti-apoptotic protein Bcl-2 was significantly reduced	Huang Yan, Fu Jingli.Baicalein induces apoptosis in human ovarian cancer HO-8910 cells by activating Caspase and Bcl-2 family proteins.Chinese Traditional and Herbal Drugs.50,2620-2624(2019).
108	HQin(Baicalin)	TGFB1(TGFβ1)	-1	Baicalin down-regulates miR-21 expression of RA synovial fibroblasts, promotes the expression of Smad7 protein, and then inhibits the expression of TGF-β1, thereby reducing the excessive secretion of synthetic extracellular matrix by RA synovial fibroblasts.	Duan Haizheng, Bai Lin, Bai Ya,et al.Study on the molecular mechanism of baicalin on alleviating synthesis of extracellular matrix of HFLS-RA.Lishizhen Medicine and Materia Medica Research.30,1299-1302(2019).
109	HQin(Wogonoside)	MAPK1	-1	wogonoside down-regulates miR-21 expression of RA synovial fibroblasts, promotes the expression of Smad7 protein, and then inhibits the expression of TGF-β1, thereby reducing the excessive secretion of synthetic extracellular matrix by RA synovial fibroblasts.	Song Yan,Gong Rui,Yang Bo et al.Effects of wogonoside on anoxia-reoxygenation induced H9c2 myocardial cells injury and expression of P38 and ERK1/2.Medical Journal of West China.31,1820-1825(2019).
110	HQin(Scutellaria Decoction)	CXCL8（IL8）	-1	Serum level of IL-6, IL-8 and MTV in the observation group All are significantly lower than the control group (P <0.01)	Wang Jinling.Study on the Effect of Scutellaria Decoction and the Changes of Serum IL-6 IL-8 and MVD and the Quality of Life in Patients with Gastric Cancer.Hebei Medicine,23,679-682(2017).
111	HQin(Scutellaria baicalensis)	CXCL8（IL8）	+1	In HMC-1 cells, SB restored IL-8 and TNF-α expression and inhibited MAP kinase expression in compound 48/80-induced HMC-1 cells. These data suggest that SB may prove to be a useful anti-inflammatory agent through its downregulation of the expression of various inflammatory mediators.	Jung HS, Kim MH, Gwak NG, et al. Antiallergic effects of Scutellaria baicalensis on inflammation in vivo and in vitro. J Ethnopharmacol. 141,345–349(2012).

112	HQin(Baicalin Oral Sustained Release Membrane)	CRP	-1	Compared with the control group, the test group had lower levels of CRP, TNF-α, and IL-6	Guo Changqing.Effect of Baicalin Oral Sustained Release Membrane Combined with Minocycline on Inflammatory Indicators in Patients with Type 2 Diabetic Periodontitis.Guangming Journal of Chinese Medicine. 34,3801-3803(2019).
113	HQin(Heat-Processed Scutellariae Radix)	CCL2	-1	The augmented expressions of hepatic oxidative stress and inflammation-related proteins, phospho-p38, phosphorylated extracellular signal-regulated kinase, phosphorylated c-Jun N-terminal kinase, nuclear factor-[Formula: see text] B p65, activator protein-1, cyclooxygenase-2, inducible nitric oxide synthase, MCP-1, intercellular adhesion molecule-1, tumor necrosis factor-[Formula: see text], and IL-6, were downregulated by the heat-processed Scutellariae Radix.	Park CH, Shin MR, An BK, et al. Heat-Processed Scutellariae Radix Protects Hepatic Inflammation through the Amelioration of Oxidative Stress in Lipopolysaccharide-Induced Mice. Am J Chin Med. 45,1233–1252(2017).
114	HQin(Baicalin)	MAPK8	+1	Baicalin induces apoptosis of human hepatocellular carcinoma HepG2 cells by activating JNK signaling pathway	Zhou Shu, Cai Tao, Qin Xinggui et al.Effect of JNK Signal Transduction of Pathway on Apoptosis Induced by Baicalin in Hepatoma Carcinoma Cells.Medical & Pharmaceutical Journal of Chinese People's Liberation Army. 20-23(2015).
115	HQin(Baicalin)	MAPK8	-1	The relative expression levels of various indexes in the baicalin treatment and preventive administration groups were PERK (1.53 ± 0.09, 1.96 ± 0.21), CHOP (2.10 ± 0.26, 2.75 ± 0.12), pJNK (2.57 ± 0.33, 3.42 ± 0.34) and Caspase- 12 (1.75 ± 0.21, 2.44 ± 0.38), compared with the PR8 group (2.86 ± 0.23, 4.75 ± 0.38, 5.02 ± 0.49, 3.64 ± 0.36), the expression level decreased significantly (F = 135.340, P <0.01; F = 74.100 , P ＜	Wu Tong, Niu Shuli, Bai Mei.Effects of a baicalin intervention on endoplasmic reticulum stress in response to infection with the PR8 strain of influenza virus.Journal of Pathogen Biology. 12,553-556,559(2017).

				0.01).	
116	HQin(Baicalein)	FOS(C-FOS)	-1	Baicalein co-acted for 1 h, can down-regulate the expression of c-FOS, CTGF, CYR61, and EGR1 downstream of estrogen-induced GPR30 in MCF-10A cells; similarly, baicalein can also inhibit estrogen up-regulation of target gene c- in MCF-12A cells. MRNA levels of FOS and CYR61.	Wang Jing.baicalein has protective effects on the 17β-estradiol-induced transformation of breast epithelial cells by interfering with estrogen receptor 30-mediated signaling transduction[D].Guizhou Medical University. (2017).
117	HQin(Baicalin)	FOS(C-FOS)	-1	Baicalin 750 and 375 mg.kg-1 doses can significantly reduce the inflammatory damage of lung tissue; significantly reduce the expression of c-jun and c-fosmRNA (P <0.05, P <0.01), significantly reduce c-jun and phosphorylation c-jun protein expression (P <0.01); significantly inhibits the secretion of TNF-α and IL-1β (P <0.01)	Wan Qiaofeng, Gu Ligang, Yin Shengjun,et al.Mechanism of Baicalin on lung tissue injury of mice with FM1 induced pneumonia.Chinese Pharmacological Bulletin.28,208-212(2012).
118	HQin(Baicalin)	IL4	+1	Baicalin not only significantly inhibits the secretion of INF-γ and IL-2 factors by Th1-type cells, but also significantly promotes the secretion of IL-4 and IL-10 factors by Th2-type cells, which makes Th2 in the immune microenvironment The overall level of type cytokines is up-regulated, which promotes the Th1 / Th2 balance to develop towards Th2 type	Ma Yannan, Yang Xiaoqi, Ma Xiaojun et al.Effect of the Baicalin on recurrent spontaneous abortion mouse model of maternal fetal immune microenvironment.Journal of Changchun University of Chinese Medicine. 34,627-630(2018).
119	HQin	IL1AIL-1α)	/		
120	HQin(scutellaria baicalensis stem-leaf total	STAT1	-1	scutellaria baicalensis stem-leaf total flavonoids (SSTF) may have a certain preventive effect on apoptosis during myocardial ischemia-reperfusion injury by	Yu Xiaomin, Hao Xiangjun, Gong Mingyu.Protective effect and mechanism of scutellaria baicalensis stem-leaf total flavonoids on myocardial ischemia-reperfusion apoptosis in rats.Chinese Journal

	flavonoids (SSTF))			down-regulating STAT1 protein and up-regulating STAT3 protein expression	of Gerontology. 33,3132-3134(2013).
121	HQin(scutellaria baicalensis stem-leaf total flavonoids (SSTF))	STAT1	-1	scutellaria baicalensis stem-leaf total flavonoids (SSTF) can prevent JAK2 and STAT1 protein expression and up-regulate STAT3 protein expression by prophylactic medication. Among them, SSTFII and III groups have better protective effect than SSTFI group, and SSTFII and III groups have an effect on injured myocardium. No obvious difference in protection	Yu Xiaomin. Intervention of scutellaria baicalensis stem-leaf total flavonoids on myocardial ischemia and reperfusion JAK / STAT pathway in rats[D].Chengde Medical College.(2012).
122	HQin(Baicalein)	EGFR	-1	Different concentrations of baicalein (10 μM, 20 μM, 40 μM, and 80 μM) after 24 h of U251 cells significantly inhibited the phosphorylation of protein EGFR and Akt in U251 cells, and blocked the promoting effect of hEGF on it. More obvious	Yuelinlin. Baicalein suppresses hEGF-induced proliferation and migration of glioma cells via the EGFR / Akt signaling pathway[D].Qingdao University. (2017).
123	HQin(Skutellaria extract SBX)	EGFR	+1	Protein Pathway Array technology was used to analyze the effect of SBX on H1975 protein expression. A total of 166 phosphorylated and non-phosphorylated proteins were detected, 53 of which were expressed, and 15 differentially expressed proteins. Among them, Cdk6, EGFR, Survivin and mTor protein expression levels were up-regulated, while p27, p-RB, Cyclin D1, Cyclin E, XIAP, p53, p-p53, p-AKT, Akt, Notch4 and Cdk4 protein expression levels were down regulated.	Liu Xiao-liang, Zhao Xin, Han Wei,et al.Influence of Skutellaria extract SBX on NSCLC cell proliferation and signaling networks.Chinese Journal of Cancer Prevention and Treatment.21,25-28(2014).
124	HQin(Baicalin)	CXCL10	-1	Fluorescence real-time quantitative PCR detection showed that the expression of CXCL9, CXCL10, CXCR3 and RAB27A in the halide group and 5%	Zhu Yiping,Jin Rong, Wang Suiquan,et al.Effect of baicalin on vitiligo mice induced by monobenzone.Chinese Journal of Clinical Pharmacology and Therapeutics.22,27-32(2017).

125	HQin(Scutellaria baicalensis）	SOD1	+1	baicalin group was significantly lower than that in the model group, while PI3K was significantly increased Biochemical index test results showed that baicalin can significantly increase the content of antioxidant enzymes SOD and CAT in Drosophila (P <0.05 or P <0.01); RT-PCR results showed that baicalin significantly up-regulated antioxidant enzymes in Drosophila Related gene SOD1, SOD2 and CAT expression levels (P <0.01 or P <0.001) and down-regulated MTH expression levels (P <0.001)	Xue Liying. Study on Screening and Mechanism of anti-aging Effective Ingredients of Scutellaria baicalensis Georgi[D].Shanxi University.(2018).
126	HQin(Scutellaria flavonoids bw-lzj-6504, bw-lzj-6517a）	PARP1	-1	Scutellaria flavonoids bw-lzj-6504, bw-lzj-6517a can significantly reduce PARP1, caspase-3 protein levels	Nie Mingxiu, Zhang Hongxiu, Jiang Liping, et al. Scutellaria flavonoids bw-lzj-6504 and bw-lzj-6517a inhibit proliferation of renal clear cell carcinoma cells in vitro. Chinese Journal of Cellular and Molecular Immunology. (2016).
127	HQin(Baicalin)	PARP1	+1	After 48 hours of 50 and 100 μg / mL baicalin, the expression levels of Parp-1 and caspase 3 spirosomal protein were significantly higher than those of the control group (all P values <0.01), while XIAP, NF-κB and Bcl- 2 The level of protein expression was significantly reduced (all P values <0.05)	Bai Huiru, Sa Yunli, Lu Shan, et al..Baicalin induces cell cycle arrest and apoptosis of human colon cancer in vitro and in vivo.Tumor.37,208-217(2017).
128	HQin(Saikosaponin d combined with baicalin）	PARP1	-1	Saikosaponin d combined with baicalin may inhibit the expression of PARP-1 in MCAO rats, reduce NAD + consumption, and achieve its neuroprotective effect on cerebral ischemia / reperfusion injury in rats	Dong Liping, Cui Yuhuan, Zhao Baomin, et al..Effect of saikosaponin d combined with baicalin on PARP-1 expression in rats with cerebral ischemia / reperfusion injury.Shanxi Journal of Traditional Chinese Medicine.37,929-931(2016).
129	HQin(Baicalin)	CASP8	+1	Baicalin can obviously induce the apoptosis of tongue squamous cell carcinoma Tca8113, which may be	Zhang Ying, Li Junmei, Qin Bowen,et al.The effects of scutellarin on apoptosis and the expression of caspase-8 in human tongue

				related to the up-regulation of caspase-8 protein expression.	squamous carcinoma Tca8113 cells.Tianjin Medical Journal.43,237-240(2015).
130	HQin(Baicalin)	IFNG(IFN -γ)	-1	After baicalin intervention, the abortion rate and embryo absorption rate of group C were significantly lower than those of group B of RSA model (P <0.01), and the contents of INF-γ and IL-2 in uterine homogenate tissue were significantly reduced (P <0.01) The levels of IL-4, IL-10 and progesterone were significantly increased (P <0.05)	Ma Yannan, Yang Xiaoqi, Ma Xiaojun,et al.Effect of the Baicalin on recurrent spontaneous abortion mouse model of maternal fetal immune microenvironment.Journal of Changchun University of Chinese Medicine.34,627-630(2018).
131	HQin(Extracts From Flos lonicerae in Combination With Baikal skullcap)	IFNG(IFN -γ)	-1	There were increases (P < 0.05) in the relative expression of ileal IFNG, TNFA, IL8, and IL1B at 3 DPI of layers in response to S. pullorum challenge (Figure 1). However, they were all decreased (P < 0.05) in layers of T group when compared with those in PC group. Furthermore, an up-regulation (P < 0.05) in the relative expression of ileal IL10 at 3 DPI was observed in the birds from T group relative to PC group.	Wang W, Jia H., Zhang H., et al. Supplemental Plant Extracts From Flos lonicerae in Combination With Baikal skullcap Attenuate Intestinal Disruption and Modulate Gut Microbiota in Laying Hens Challenged by Salmonella pullorum. Frontiers in Microbiology. 10.
132	HQin(Baicalin)	IL2	-1	After baicalin intervention, the abortion rate and embryo absorption rate of group C were significantly lower than those of group B of RSA model (P <0.01), and the contents of INF-γ and IL-2 in uterine homogenate tissue were significantly reduced (P <0.01) The levels of IL-4, IL-10 and progesterone were significantly increased (P <0.05)	Ma Yannan, Yang Xiaoqi, Ma Xiaojun,et al.Effect of the Baicalin on recurrent spontaneous abortion mouse model of maternal fetal immune microenvironment.Journal of Changchun University of Chinese Medicine.34,627-630(2018).
133	HQin(Baicalin)	CAT	+1	Compared with the H group, the uterine tissue damage of the H + Bai (baicalin) group was reduced, the level	Gao Shansong, Wang Lei, Li Huatao, et al.Baicalin Regulates Heat Stress-induced Uterine Oxidative Damage in Mice(Mus musculus)

				of apoptosis was reduced, the content of malondialdehyde (MDA) was significantly reduced (P <0.01), and the total superoxide dismutation Enzyme (total superoxide dismutase dismutase, T-SOD) activity is significantly increased (P <0.01), glutathione peroxidase (glutathione peroxidase, GSH-Px) and catalase (catalase, CAT) Significantly increased (P <0.05)	via Nrf2/Keap1 Signaling Pathway.Journal of Agricultural Biotechnology.27,2013-2022(2019).
134	HQin(Baicalin)	IL10	+1	After baicalin intervention, the abortion rate and embryo absorption rate of group C were significantly lower than those of group B of RSA model (P <0.01), and the contents of INF-γ and IL-2 in uterine homogenate tissue were significantly reduced (P <0.01) The levels of IL-4, IL-10 and progesterone were significantly increased (P <0.05)	Ma Yannan, Yang Xiaoqi, Ma Xiaojun,et al.Effect of the Baicalin on recurrent spontaneous abortion mouse model of maternal fetal immune microenvironment.Journal of Changchun University of Chinese Medicine.34,627-630(2018).
135	HQin	HMOX1	/		
136	HQin(Baicalein)	BAX	-1	In the SAP + baicalein group, the levels of serum amylase, IL-6, TNF-α, MDA content in the lung tissue and apoptosis index decreased, SOD activity increased, Bax and p-p38MAPK in the lung tissue of the rats in the SAP and baicalein group Decreased protein expression, increased Bcl-2 protein expression (P <0.05)	Zhu Xiaolin, Zhao Haiyan, Yang Jilin.Effects of baicalein on lung tissue injury in rats with severe acute pancreatitis[J/OL].Journal of Zhengzhou University(Medical Sciences).37-40(2020).
137	HQin(Baicalein)	BAX	+1	After 20, 40 and 80 μmol / L baicalein treatment for 24 h, the expression of pro-apoptotic protein Bax in ovarian cancer HO-8910 cells was significantly	Huang Yan, Fu Jingli.Baicalein induces apoptosis in human ovarian cancer HO-8910 cells by activating Caspase and Bcl-2 family proteins.Chinese Traditional and Herbal Drugs.50,2620-2624(2019).

				increased, while the expression of anti-apoptotic protein Bcl-2 was significantly reduced	
138	JYH(Flos Lonicerae extracts)	PTGS2	-1	The suppression of transcription of IL-1β, IL-6, COX-2, and P-Selectin genes with Flos Lonicerae extracts was greater than that of CGA in PFOS-treated HUVECs, while the degree of suppression on PFOS-induced expression of NOS3 and ICAM-1 was greater for CGA.	Liao Y, Dong S, Kiyama R, et al. Flos lonicerae extracts and chlorogenic acid protect human umbilical vein endothelial cells from the toxic damage of perfluorooctane sulphonate. Inflammation. 36,767–779(2013).
139	JYH(unfermented Flos Lonicera (UFL) and fermented Flos Lonicera (FFL))	PTGS2	-1	In cell-based studies, treatment with both unfermented Flos Lonicera (UFL) and fermented Flos Lonicera (FFL) formulations resulted in suppression of LPS-induced NO production and gene expression of vital proinflammatory cytokines (TNF-α, COX-2, and IL-6) in RAW 264.7 cells	Wang JH, Bose S, Kim GC, et al. Flos Lonicera ameliorates obesity and associated endotoxemia in rats through modulation of gut permeability and intestinal microbiota. PLoS One. 9,e86117(2014).
140	JYH(extracts of Lonicera japonica)	PTGS2	-1	Boiled LJ aqueous extracts directly inhibited both COX-1 and COX-2 activity, while non-boiled extracts stimulated COX-1. Boiled LJ extracts also inhibited expression of IL-1beta-induced COX-2 protein expression and suppressed its mRNA induction by IL-1beta in A549 cells.	Xu Y, Oliverson BG, Simmons DL. Trifunctional inhibition of COX-2 by extracts of Lonicera japonica: direct inhibition, transcriptional and post-transcriptional down regulation. J Ethnopharmacol. 111,667–670(2007).
141	JYH	iNOS	/		
142	JYH(Lonicera japonica aqueous extract)	RELA(P65)	-1	Observation of the improvement effect and mechanism of the Lonicera japonica aqueous extract on diabetic retinopathy. Western blot results showed that FL can inhibit the nuclear factor-κB (NF-κB, NF-κB in streptozotocin (STZ) -induced diabetic mouse	Zhou Lingyu, Yu Zengyang, Palida Abuliz,et al.Improvement of Lonicera japonica aqueous extract on diabetic retinopathy.Chinese Pharmacological Bulletin.31,1710-1714(2015).

				retina)) Transnuclear activation of the p65 subunit.	
143	JYH(Honeysuckle)	TNF	-1	Honeysuckle can inhibit the overexpression of IL-1β, PGE2, and TNF-α, reduce cervical mucosal necrosis, and has a certain effect on rat cervicitis.	Wang Ping, Zhang Xiaoling.Therapeutic effect of honeysuckle on cervicitis in rats. Chinese Journal of Gerontology.032,1441-1443(2012).
144	JYH(Lonicera japonica Thunb extract)	IL6	-1	Lonicera japonica Thunb extract has an immunosuppressive effect, inhibiting or reducing inflammation by inhibiting pro-inflammatory cytokines such as TNF-α, IL-1β, and IL-6	Yan Xuelong, Meng Aiping, Pu sheban.Research Progress on Activities of Anti-inflammation and Immunity from the flower of Lonicera japonica Thunb..Chinese Wild Plant Resources.35,41-44(2016).
145	JYH(Flos Lonicerae extracts)	IL6	-1	The suppression of transcription of IL-1β, IL-6, COX-2, and P-Selectin genes with Flos Lonicerae extracts was greater than that of CGA in PFOS-treated HUVECs, while the degree of suppression on PFOS-induced expression of NOS3 and ICAM-1 was greater for CGA.	Liao Y, Dong S, Kiyama R, et al. Flos lonicerae extracts and chlorogenic acid protect human umbilical vein endothelial cells from the toxic damage of perfluorooctane sulphonate. Inflammation. 36,767–779(2013).
146	JYH(unfermented Flos Lonicera (UFL) and fermented Flos Lonicera (FFL))	IL6	-1	In cell-based studies, treatment with both unfermented Flos Lonicera (UFL) and fermented Flos Lonicera (FFL) formulations resulted in suppression of LPS-induced NO production and gene expression of vital proinflammatory cytokines (TNF-α, COX-2, and IL-6) in RAW 264.7 cells	Wang JH, Bose S, Kim GC, et al. Flos Lonicera ameliorates obesity and associated endotoxemia in rats through modulation of gut permeability and intestinal microbiota. PLoS One.9, e86117(2014).
147	JYH(Polyphenol Components Isolated from Korea Lonicera japonica)	MAPK14（P38α）	-1	polyphenol components isolated from Korea L. japonica T. should have anti-inflammatory effect on LPS-stimulated RAW 264.7 cells through the decrease of proinflammatory mediators expression by suppressing NF-κB and p38 MAPK activity.	Park Kwang-Il,Kang Sang-Rim,Park Hyeon-Soo, et al. Regulation of Proinflammatory Mediators via NF-κB and p38 MAPK-Dependent Mechanisms in RAW 264.7 Macrophages by Polyphenol Components Isolated from Korea Lonicera japonica THUNB. .Evid Based Complement Alternat Med. 2012, 828521(2012).

148	JYH(Honeysuckle)	CASP3	-1	Honeysuckle can significantly inhibit the expression of Caspase-3 and NF-κB in mice with viral myocarditis, providing an experimental basis for the clinical application of honeysuckle in the treatment of viral myocarditis	Lou Xusheng, Hu Jinghong, Wang Fen,et al.Effect of honeysuckle on expression of Caspase-3 and NF-κB in mice with viral myocarditis.Shanghai Journal of Traditional Chinese Medicine.53,71-74(2019).
149	JYH(extracts of Lonicera japonica)	PTGS1(COX1)	-1	Boiled LJ aqueous extracts directly inhibited both COX-1 and COX-2 activity, while non-boiled extracts stimulated COX-1. Boiled LJ extracts also inhibited expression of IL-1beta-induced COX-2 protein expression and suppressed its mRNA induction by IL-1beta in A549 cells.	Xu Y, Oliverson BG, Simmons DL. Trifunctional inhibition of COX-2 by extracts of Lonicera japonica: direct inhibition, transcriptional and post-transcriptional down regulation. J Ethnopharmacol. 111,667–670(2007).
150	JYH(Honeysuckle)	ICAM1	-1	Compared with normal saline group, serum IFN-γ level and ICAM-I and IL-12 expression in skin lesions of dexamethasone group, Cortex Dictamni group, honeysuckle group and radix angelicae sinensis group were significantly lower than that of normal saline group ($P < 0.01$-0.05)	Huang Peng. Study on the mechanism of the effects of Cortex Dictamni, rehmannia glutinosa, catmint, angelica, honeysuckle and radix angelicae sinensis on allergic contact dermatitis[D].Guangdong:Guangdong Medical University.(2007).
151	JYH(Lonicera japonica Thunb extract)	IL1B(IL1β)	-1	Lonicera japonica Thunb extract has an immunosuppressive effect, inhibiting or reducing inflammation by inhibiting pro-inflammatory cytokines such as TNF-α, IL-1β, and IL-6	Yan Xuelong, Meng Aiping, Pu sheban.Research Progress on Activities of Anti-inflammation and Immunity from the flower of Lonicera japonica Thunb..Chinese Wild Plant Resources.35,41-44(2016).
152	JYH(Flavonoids Isolated from Flowers of Lonicera japonica Thunb)	IL1B(IL1β)	-1	PELJ significantly inhibited LPS-induced interleukin-1β and tumor necrosis factor-α expressions and LPS-induced nitric oxide (NO) and prostaglandin E2 expressions by down-regulating inducible enzyme NO synthase and cyclooxygenase-2 at the protein and	Han MH, Lee WS, Nagappan A, et al. Flavonoids Isolated from Flowers of Lonicera japonica Thunb. Inhibit Inflammatory Responses in BV2 Microglial Cells by Suppressing TNF-α and IL-β Through PI3K/Akt/NF-kb Signaling Pathways. Phytother Res. 30,1824–1832(2016).

				mRNA levels.	
153	JYH(Flos Lonicerae extracts)	IL1B(IL1β)	-1	The suppression of transcription of IL-1β, IL-6, COX-2, and P-Selectin genes with Flos Lonicerae extracts was greater than that of CGA in PFOS-treated HUVECs, while the degree of suppression on PFOS-induced expression of NOS3 and ICAM-1 was greater for CGA.	Liao Y, Dong S, Kiyama R, et al. Flos lonicerae extracts and chlorogenic acid protect human umbilical vein endothelial cells from the toxic damage of perfluorooctane sulphonate. Inflammation.36,767–779(2013).
154	JYH(extracts of Lonicera japonica)	IL1B(IL1β)	-1	Boiled LJ aqueous extracts directly inhibited both COX-1 and COX-2 activity, while non-boiled extracts stimulated COX-1. Boiled LJ extracts also inhibited expression of IL-1beta-induced COX-2 protein expression and suppressed its mRNA induction by IL-1beta in A549 cells.	Xu Y, Oliverson BG, Simmons DL. Trifunctional inhibition of COX-2 by extracts of Lonicera japonica: direct inhibition, transcriptional and post-transcriptional down regulation. J Ethnopharmacol. 111,667–670(2007).
155	JYH(Honeysuckle)	TP53（P53）	-1	Honeysuckle mainly increases the effect on Mdm2 protein in the P53 pathway. P53 can initiate apoptosis, and Mdm2 can feedback inhibit P53 expression. Pharmacological effects	Ouyang Yulin. Study on literature research of Traditional Chinese Medicine in Intervening Cardiomyocyte apoptosis and the study on pharmacological mechanism of Yixin Jiedu Formula [D].Beijing University of Chinese Medicine.(2013).
156	JYH(Skutellaria extract SBX)	TP53（P53）	-1	Protein Pathway Array technology was used to analyze the effect of SBX on H1975 protein expression. A total of 166 phosphorylated and non-phosphorylated proteins were detected, 53 of which were expressed, and 15 differentially expressed proteins. Among them, Cdk6, EGFR, Survivin and mTor protein expression levels were up-regulated, while p27, p-RB, Cyclin D1, Cyclin E, XIAP, p53, p-p53, p-AKT, Akt, Notch4 and Cdk4 protein expression levels were down regulated.	Liu Xiaoliang, Zhao Xin, Han Wei,et al.Influence of Skutellaria extract SBX on NSCLC cell proliferation and signaling networks.Chinese Journal of Cancer Prevention and Treatment.21,25-28(2014).

157	JYH(Polysaccharide from Lonicera japonica)	BCL2	-1	The experimental results show that the Polysaccharide from Lonicera japonica in the 30 mg / (kg · d) and 90 mg / (kg · d) dose groups can up-regulate the expression level of Bax protein in mice S180 sarcoma and down-regulate the expression level of Bcl-2 protein, Bax / Bcl-2 Ratio increase	Liu Yuguo, Liu Yuhong, Jiang Haiqiang.Inhibitory Effect and Mechanism of Polysaccharide from Lonicera japonica on Mice Bearing S180 Sarcoma.Journal of Oncology.18,584-587(2012).
158	JYH(Honeysuckle)	BCL2	+1	To explore the molecular mechanism of the anti-oxidation of honeysuckle. The apoptosis rate of the Jb group was significantly lower than that of the H2 and Ja groups, the expression of Bcl-2 increased, and the expressions of HSP-70, NF-kB, Bax and Caspase-3 decreased.	Meng Mingli, Gong Cuicui, Zheng Yuxia,et al.The molecular mechanism of anti-oxidative effects of honeysukle: An experimental study.Practical Journal of Medicine & Pharmacy.1104-1106(2008).
159	JYH	TGFB1(TGFβ1)	/		
160	JYH	MAPK1	/		
161	JYH(Extracts From Flos lonicerae in Combination With Baikal skullcap)	CXCL8（IL8）	-1	pullorum challenge impaired (P < 0.05) the production performance (egg production, feed intake, and feed efficiency) of laying hens, increased (P < 0.05) serum endotoxin content and frequency of Salmonella -positive organs, as well as up-regulated (P < 0.05) ileal expression of pro-inflammatory cytokines including IFNG , TNFA , IL8 , and IL1B , whereas PE addition reversed (P < 0.05) these changes and increased (P < 0.05) ileal IL10 expression.	Wang Wei-Wei,Jia Hong-Jie,Zhang Hai-Jun,et al. Supplemental Plant Extracts From Flos lonicerae in Combination With Baikal skullcap Attenuate Intestinal Disruption and Modulate Gut Microbiota in Laying Hens Challenged by Salmonella pullorum .. Frontiers in microbiology.10,2019.
162	JYH(Honeysuckle decoction)	CRP	-1	Honeysuckle decoction can effectively reduce the levels of inflammatory factors and CPR in patients, and	Song Changliang, Du Xuefei, Yang Qiong, et al.Immunomodulatory effect of honeysuckle decoction on patients with radiation

				stabilize the proportion of lymphocyte subgroup structure	esophagitis.Journal of Modern Oncology.27,580-583(2019).
163	JYH	CCL2	/		
164	JYH((Honeysuckle) Caffeic acid CA)	MAPK8	-1	(Honeysuckle) Caffeic acid CA can inhibit APAP-induced phosphorylation activation of ERK1 / 2 and JNK. After applying inhibitors, we found that ERK1 / 2 inhibitors can inhibit APAP-induced Egr1 nuclear translocation	Pang Chun. Caffeic acid antagonizes acetaminophen hepatotoxicity and its mechanism[D].Shanghai:Shanghai University of Traditional Chinese Medcine.(2015).
165	JYH	FOS(C-FOS)	-1	Mechanistically, ChondroT and its constituent herbs downregulated the expressional levels of osteoclast-specific proteins such as NFATc1, c-Fos, Cathepsin K, and matrix metalloproteinase 9 (MMP9) by suppressing NF-κB translocation to nucleus and MAPKs phosphorylation at different levels.	Guo RH, Kim SJ, Choi CH, Na CS, Kang BY, Kim YR. Inhibitory effects of ChondroT and its constituent herbs on RANKL-induced osteoclastogenesis. BMC Complement Altern Med. 20,319(2019) .
166	JYH(WIN-34B)	FOS(C-FOS)	-1	WIN-34B increased the OPG/RANKL ratio and the expression of RUNX2, and suppressed the expression of IL-17, c-Fos, and TNF-α. It also suppressed the activation of NF-κB, IκBα, p38 MAPK, and JNK in a dose-dependent manner.	Seo BK, Ryu HK, Park YC, Huh JE, Baek YH. Dual effect of WIN-34B on osteogenesis and osteoclastogenesis in cytokine-induced mesenchymal stem cells and bone marrow cells. J Ethnopharmacol. 193,227-236(2016).
167	JYH(Honeysuckle Extract)	IL4	-1	The eosinophil count, serum IL-4, IL-17, sIgE levels, tissue IL-17 mRNA expression, IL-17 protein expression level in the nasal mucosa pathological section of the honeysuckle treatment group and the positive control group were all compared with the model The control group decreased (all P <0.01), while the serum levels of IL-2 and IFN-γ increased (all	Jian Lei, Xiao caiwen, he Qingwen, et al.Effect of Honeysuckle Extract on Expression of Cytokines in Mice with Allergic Rhinitis.Acta Medicinae Universitatis Scientiae et Technologiae Huazhong.46,285-290(2017).

				P <0.01).	
168	JYH	IL1AIL-1 α)	/		
169	JYH(Honeysuckle alcohol extract)	STAT1	-1	Honeysuckle alcohol extract can significantly inhibit the lipopolysaccharide-induced JNK, ERK 1/2, p38 MAPKs, PI3K / Akt and JAK1 pathway signaling and STAT1 / 3 transcriptional activation, thereby inhibiting the activation of NF-κB	Li Yongwei, Wang Zhisheng, Liu Xinwei.Advances in anti-infective effects of honeysuckle.China Modern Doctor.57,165-168(2019).
170	JYH	EGFR	/		
171	JYH	CXCL10	/		
172	JYH(Honeysuckle)	SOD1	+1	After intragastric administration of honeysuckle for 1 to 2 hours, the plasma levels of T-AOC, GSH-Px, GSH, and SOD in rats were significantly higher than before gavage (P <0.05), while the MDA content was significantly reduced (P <0.05)	Gong Cuicui, Zheng Naigang, Wu Jinglan,et al.Antioxidant effect of honeysuckle on hepatic RBL cells of rats in vivo and in vitro and mechanism.Journal of Jilin University(Medicine Edition).35,1074-1078(2009).
173	JYH	PARP1	/		
174	JYH	CASP8	/		
175	JYH(Flos Lonicerae Japonicas)	IFNG(IFN-γ)	-1	Compared with normal saline group, serum IFN-γ level and ICAM-I and IL-12 expression in skin lesions of dexamethasone group, Cortex Dictamni group, Flos Lonicerae Japonicas group and Radix Angelicae Sinensis group were significantly lower than that of normal saline group (P <0.01-0.05)	Huang Peng. Study on the mechanism of allergic contact dermatitis in mice with Cortex Dictamni, adhesive rehmannia, Herba Schizonepetae, Radix Angelicae Sinensis, Flos Lonicerae Japonicas and Radix Angelicae Dahuricae[D].Guangdong:Guangdong Medical University.(2007).
176	JYH(Honeysuckle Extract)	IFNG(IFN-γ)	+1	The eosinophil count, serum IL-4, IL-17, sIgE levels, tissue IL-17mRNA expression, IL-17 protein expression level in the nasal mucosa pathological	Jian Lei, Xiao caiwen, he Qingwen,et al.Effect of Honeysuckle Extract on Expression of Cytokines in Mice with Allergic Rhinitis.Acta Medicinae Universitatis Scientiae et Technologiae

				section of the honeysuckle treatment group and the positive control group were all compared with the model control Group decreased (all P <0.01), while serum IL-2 and IFN-γ levels increased (all P <0.01)	Huazhong.46,285-290(2017).
177	JYH(Honeysuckle Extract)	IL2	+1	The eosinophil count, serum IL-4, IL-17, sIgE levels, tissue IL-17mRNA expression, IL-17 protein expression level in the nasal mucosa pathological section of the honeysuckle treatment group and the positive control group were all compared with the model control Group decreased (all P <0.01), while serum IL-2 and IFN-γ levels increased (all P <0.01)	Jian Lei, Xiao caiwen, he Qingwen,et al.Effect of Honeysuckle Extract on Expression of Cytokines in Mice with Allergic Rhinitis.Acta Medicinae Universitatis Scientiae et Technologiae Huazhong.46,285-290(2017).
178	JYH(Flavonoids from Lonicera japonica Thunb)	CAT	+1	Flavonoids from Lonicera japonica Thunb in each dose group significantly increased the total antioxidant capacity of cells and cell culture fluids (P <0.05), and significantly increased SOD, GSH-Px, CAT activity and GSH content (P <0.05)	Luo Lei, Zhang Bingjie, Wei Qianqian, et AL.Protective Effects of Flavonoids from Lonicera japonica Thunb. on Hydrogen Peroxide Induced Toxicity in RAW 264.7 Cells.Journal of Chinese Institute of Food Science and Technology.19,18-25(2019).
179	JYH	IL10	/		
180	JYH	HMOX1	/		
181	JYH(Polysaccharide from Lonicera japonica)	BAX	+1	The experimental results show that the Polysaccharide from Lonicera japonica in the 30 mg / (kg · d) and 90 mg / (kg · d) dose groups can up-regulate the expression level of Bax protein in mice S180 sarcoma and down-regulate the expression level of Bcl-2 protein, Bax / Bcl-2 Ratio increase	Liu Yuguo, Liu Yuhong, Jiang Haiqiang.Inhibitory Effect and Mechanism of Polysaccharide from Lonicera japonica on Mice Bearing S180 Sarcoma.Journal of Chinese Oncology.18,584-587(2012).
182	JYH(Honeysukle)	BAX	-1	To explore the molecular mechanism of the anti-oxidation of honeysuckle. The apoptosis rate of	Meng Mingli, Gong Cuicui, Zheng Yuxia,et al.The molecular mechanism of anti-oxidative effects of honeysukle: An experimental

				the Jb group was significantly lower than that of the H2 and Ja groups, the expression of Bcl-2 increased, and the expressions of HSP-70, NF-kB, Bax and Caspase-3 decreased.	study.Practical Journal of Medicine & Pharmacy.1104-1106(2008).
183	JYH(Lonicera japonica solution)	BAX	-1	Lonicera japonica solution affects the expression of Bax protein, Caspase-3 protein and NF-κB protein in the myocardial tissue of mice with heart injury induced by ephedrine. Lonicera japonica solution has a strong antioxidant effect, which can reduce the production of oxidation products, increase the activity of antioxidant enzymes, reduce the expression of apoptosis proteins caused by ephedrine, reduce the damage of cardiomyocytes, and protect the heart	Liu Tingting. Effect of Lonicera japonica solution on the damage of mice heart induced by ephedrine[D].Northwest Normal University.(2016).
184	GC(Gl radix and glycyrrhizin (GL), the main part of Gl radix)	PTGS2	-1	The present study was performed to clarify the effects of Gl radix and glycyrrhizin (GL), the main part of Gl radix, on estradiol (E2)-related endometrial carcinogenesis. Both Gl radix and GL exerted a significant decrease in the COX-2, IL-1alpha and TNF-alpha mRNA expressions.	Niwa K, Lian Z, Onogi K, et al. Preventive effects of glycyrrhizin on estrogen-related endometrial carcinogenesis in mice. Oncol Rep. 17,617–622(2007).
185	GC(Glycyrrhizin)	PTGS2	-1	The results showed that glycyrrhizin significantly suppressed LPS-induced TNF-α, IL-1β, NO, and PGE2 production. Also, LPS-induced iNOS and COX-2 expression were attenuated by glycyrrhizin.	Wang XR, Hao HG, Chu L. Glycyrrhizin inhibits LPS-induced inflammatory mediator production in endometrial epithelial cells. Microb Pathog. 109,110–113(2017).
186	GC(Licorice ethanol extract）	PTGS2	+1	The transcriptional level of Kitl was not significantly different between the groups, whereas mRNA levels of Cyp11a1 and Ptgs2 were significantly up-regulated in	Yang H, Kim HJ, Pyun BJ, et al. Licorice ethanol extract improves symptoms of polycytic ovary syndrome in Letrozole-induced female rats. Integr Med Res. 7,264–270(2018).

				the GRR treatment group as compared with the PCOS group	
187	GC(18β glycyrrhetinic acid, isoliquiritigenin, and ursolic acid）	PTGS2	-1	18β glycyrrhetinic acid, isoliquiritigenin, and ursolic acid inhibited the gene expressions of ICAM-1, TNF-α, COX-2, and iNOS, partly through inhibiting NF-κB expression and attenuating NF-κB nuclear translocation.	Jun-Xian Zhou,Michael Wink. Evidence for Anti-Inflammatory Activity of Isoliquiritigenin, 18β Glycyrrhetinic Acid, Ursolic Acid, and the Traditional Chinese Medicine Plants Glycyrrhiza glabra and Eriobotrya japonica , at the Molecular Level. Medicines.6,(2019).
188	GC(Glycyrrhizae radix extract）	PTGS2	-1	DSS markedly induced COX-2 expression in colon tissue versus control group, but increased COX-2 expression was significantly reduced by GR administration	Jeon YD, Bang KS, Shin MK, et al. Regulatory effects of glycyrrhizae radix extract on DSS-induced ulcerative colitis. BMC Complement Altern Med. 16,459(2016).
189	GC(Glycyrrhizin）	iNOS(NOS2)	-1	The results showed that glycyrrhizin significantly suppressed LPS-induced TNF-α, IL-1β, NO, and PGE2 production. Also, LPS-induced iNOS and COX-2 expression were attenuated by glycyrrhizin.	Wang XR, Hao HG, Chu L. Glycyrrhizin inhibits LPS-induced inflammatory mediator production in endometrial epithelial cells. Microb Pathog. 109,110–113(2017).
190	GC(Total flavonoid from Glycyrrhizae Radix et Rhizoma (TFRG) and isoliquiritigenin (ISL)）	iNOS(NOS2)	+1	Total flavonoid from Glycyrrhizae Radix et Rhizoma (TFRG) and isoliquiritigenin (ISL) dose-dependently inhibited the expression of arginase 1 (Arg-1) at the gene and protein levels, and increased the heme oxygenase 1 (HO-1) Gene expression, while increasing the protein expression of inducible nitric oxide synthase (i NOS), increasing the expression of microRNA-155 and one of its target genes, SHIP1, and reducing signal transduction and transcriptional activation factors 3 and 6 (STAT3 / 6) Protein	Wang Yuanyuan, Tan Xi, Yang Xiaolu,et al.Total flavonoid from Glycyrrhizae Radix et Rhizoma and its ingredient isoliquiritigenin regulation M2 phenotype polarization of macrophages.China Journal of Chinese Materia Medica.40,4475-4481(2015).

				phosphorylation level	
191	GC(Glycyrrhetinic acid）	RELA(P65)	-1	The cells in the experimental group were treated with 100 μmol·L-1 glycyrrhetinic acid, the control group was treated with 10 μg · L-1 doxorubicin (DOX), and the cells in the blank group were treated with the same amount of normal saline. As a result, after intervention for 48 h, the p65 protein was relatively The expression levels were 1.06 ± 0.21,0.63 ± 0.15, 0.66 ± 0.24	Ding Peijian, Wang Jingjing, Zhao Jin,et al.Effect of glycyrrhetinic acid on proliferation of gastric cancer cell line SGC7901 and its mechanism.The Chinese Journal of Clinical Pharmacology.35,1902-1904+1908(2019).
192	GC(Glycyrrhizin ）	TNF	-1	After treatment, the TNF-α, IL-6 and PASI scores of the study group were lower than those of the control group, the difference was statistically significant (P <0.05);	Lin Junjie.Effect of Acitretin Capsule Combined With Compound Glycyrrhizin on Psoriasis Effect and Inflammation.China Health Standard Management.11,81-83(2020).
193	GC(Glycyrrhetinic acid）	TNF	-1	Glycyrrhetinic acid can reduce airway inflammation and improve asthma symptoms by reducing inflammatory cells (lymphocytes and eosinophils) and down-regulating the levels of cytokines IgE, IL-4 and TNF-α.	Chen Wei, Ma Lei, Yang Lishan. Effects of glycyrrherinic acid on IgE, IL-4 and TNF-α in bronchial asthma rats. Pharmacology and Clinics of Chinese Materia Medica.031, 52-55(2015).
194	GC(Glycyrrhizae radix extract）	IL6	-1	IL-6 levels were lower in the GR groups (25 mg/kg; 63.620 ± 7.942 pg/mL, 50 mg/kg; 36.143 ± 6.652 pg/mL)	Jeon YD, Bang KS, Shin MK, et al. Regulatory effects of glycyrrhizae radix extract on DSS-induced ulcerative colitis. BMC Complement Altern Med. 16,459(2016).
195	GC(Prepared Licorice Decoction）	IL6	-1	Prepared Licorice Decoction may promote the expression of IL-2 and IL-12 by inhibiting the expression of serum IL-1β and IL-6, regulate the immune function, reduce fatigue symptoms, and play the role of treating chronic fatigue syndrome	Liu Weicheng, Li Jiexuan, Guo Yongning,et al.Effects of Prepared Licorice Decoction on Behavioristics and 4 Kinds of IL in Rats with Chronic Fatigue Syndrome.Progress in Veterinary Medicine.39,70-73(2018).

196	GC(Isoliquiritigenin）	IL6	-1	Further analysis demonstrated that ISL not only downregulated IL-6 expression but also significantly decreased levels of phosphorylated ERK and STAT3 and could inhibit phosphorylation levels of ERK and STAT3 induced by recombinant human IL-6, which are critical signaling proteins in IL-6 signaling regulation networks.	Chen X, Wu Y, Jiang Y, et al.Isoliquiritigenin inhibits the growth of multiple myeloma via blocking IL-6 signaling. J Mol Med (Berl). 90,1311-9(2012).
197	GC(Isoliquiritigenin）	IL6	-1	The increased expression of fibrosis markers (α-SMA, FN, COL-1) and related inflammatory factors (CCL2, CD68, F4 / 80, TNF-α, and IL-6) in the model group were different degrees in the treatment group reduce.	He Lin. molecular mechanism of isoliquiritigenin in ameliorating experimental mouse chronic pancreatitis[D].The Second Military Medical University.2019.
198	GC(Isoliquiritigenin）	IL6	-1	In HepG2 and L02 cells, the expression levels of CXCL9, CXCL10, CXCL11, and IL-6 increased after treatment with IFN-γ. This high expression can be suppressed by ISL in a dose-dependent manner (IFN-γ + 5μg / mlISL Group vs IFN-y induction group, P <0.05)	Wu Shanshan. The role of the isoliquiritigenin on the inflammation reaction of IFN-γ-induced hepatocytes and its mechanism[D].Zhejiang University.(2016).
199	GC(Isoglycyrrhizinate）	IL6	-1	Magnesium isoglycyrrhizinate downregulates the expression of chemokines and their receptors CCL3, CCL5, CCL8, CCL11, CCL13, CCL19, CCL21, CXCL1, CXCL2, CXCL8, CCR1, CCR3, CCR4, CCR7, CXCR1, CXCR2 in gastric cancer tissues of mice; TLRs, MyD88, Tollip, NF-κb expression in mouse gastric cancer tissues; down-regulation of il1a, il1b, il1r1, il1rap, il1rn, il5, il6, il6r, il10, il10rb, il15, il17a, il18 in mouse gastric cancer tissues , Il22,	Miao Yuqing. Anti-tumor mechanism of Magnesium Isoglycyrrhizinate in regulating the inflammatory microenvironment of Gastric Cancer[D].The Second Military Medical University.(2017).

				il23a, il23r expression, up-regulated the expression of il2, il12	
200	GC(Glycyrrhetinic acid）	MAPK14（P38α）	-1	Glycyrrhetinic acid may inhibit ionizing radiation-induced inflammation by inhibiting NADPH oxidase / ROS / p38MAPK signaling pathway	Su Li, Wang Qi, Huang Fei,et al.Effect of glycyrrhetinic acid attenuating radiation-induced inflammation and its mechanism.Chinese Journal of Clinical Pharmacology and Therapeutics. 21,1088-1094(2016).
201	GC(flavonoids from Glycyrrhiza uralensis）	CASP3	-1	flavonoids from Glycyrrhiza uralensis can resist the thioacetamide-induced hepatic fibrosis in rats. Its mechanism may be related to the downregulation of the protein expressions of TGF-β1and Caspase-3.	Jing Jing, Zhao Jinying, Hua Bing,et al.Inhibitory effect of flavonoids from Glycyrrhiza uralensis on expressions of TGF-β1 and Caspase-3 in thioacetamide-induced hepatic fibrosis in rats.China Journal of Chinese Materia Medica.40,3034-3040(2015).
202	GC(Licochalcone A）	PTGS1(COX1)	—	Licochalcone A had no effect on COX-1-dependent PGE2 production, whereas indometacin (100 nM), a dual inhibitor of COX-1 and COX-2, was very effective.	Furuhashi I, Iwata S, Shibata S, et al. Inhibition by licochalcone A, a novel flavonoid isolated from liquorice root, of IL-1beta-induced PGE2 production in human skin fibroblasts. J Pharm Pharmacol.57, 1661–1666(2005).
203	GC(Isoliquiritigenin）	ICAM1	-1	The effect of isoliquiritigenin in inhibiting gliomas may be related to reducing the expression of ICAM-1 and VCAM-1 in glioma rats	Cao Yang, Wang Yong, Dai Jinying, et al.Effect of Isoliquiritigenin on the expression of ICAM-1and VCAM-1 in rat brain glioma.Progress of Anatomical Sciences.22,513-515(2016).
204	GC(Prepared Licorice Decoction）	IL1B(IL1β)	-1	Prepared licorice decoction may promote the expression of IL-2 and IL-12 by inhibiting the expression of serum IL-1β and IL-6, regulate the immune function, reduce fatigue symptoms, and play the role of treating chronic fatigue syndrome	Liu Weicheng, Li Jiexuan, Guo Yongning,et al.Effects of Prepared Licorice Decoction on Behavioristics and 4 Kinds of IL in Rats with Chronic Fatigue Syndrome.Progress in Veterinary Medicine.39,70-73(2018).
205	GC(Glycyrrhizin)	IL1B(IL1β)	-1	glycyrrhizin significantly suppressed LPS-induced TNF-α, IL-1β, NO, and PGE2 production. Also, LPS-induced iNOS and COX-2 expression were attenuated by glycyrrhizin. Furthermore, glycyrrhizin	Wang XR, Hao HG, Chu L. Glycyrrhizin inhibits LPS-induced inflammatory mediator production in endometrial epithelial cells. Microb Pathog. 109,110–113(2017).

				significantly attenuated TLR4 expression and NF-κB activation induced by LPS in MEEC.	
206	GC(Isoliquiritigenin(ISL)）	IL1B(IL1β)	-1	When stimulated by LPS for12 h,the expression of TNF-αand IL-1βm RNA in the culture medium are reduced by ISL pre-treatment(P<0.05).After LPS stimulation for 24 h,the expression of COX2 and i NOS m RNA and protein can be reduced(P<0.05).After stimulated by LPS from 0.5 to 2 h,p-ERK expression has be decreased.	FU Yan,YANG Pin,ZHAO Yang,XU Ying.Isoliquiritigenin suppresses microglial activation and neuroinflammation in primary microglia.中国药理学与毒理学杂志.30,1024(2016).
207	GC(Glycyrrhizin)	IL1B(IL1β)	-1	The results showed that glycyrrhizin significantly suppressed LPS-induced TNF-α, IL-1β, NO, and PGE2 production. Also, LPS-induced iNOS and COX-2 expression were attenuated by glycyrrhizin.	Wang XR, Hao HG, Chu L. Glycyrrhizin inhibits LPS-induced inflammatory mediator production in endometrial epithelial cells. Microb Pathog. 109,110–113(2017).
208	GC(Magnesium isoglycyrrhizinate）	IL1B(IL1β)	-1	Magnesium isoglycyrrhizinate downregulates the expression of chemokines and their receptors CCL3, CCL5, CCL8, CCL11, CCL13, CCL19, CCL21, CXCL1, CXCL2, CXCL8, CCR1, CCR3, CCR4, CCR7, CXCR1, CXCR2 in gastric cancer tissues of mice; TLRs, MyD88, Tollip, NF-κb expression in mouse gastric cancer tissues; down-regulation of il1a, il1b, il1r1, il1rap, il1rn, il5, il6, il6r, il10, il10rb, il15, il17a, il18 in mouse gastric cancer tissues , Il22, il23a, il23r expression, up-regulated the expression of il2, il12	Miao Yuqing. Anti-tumor mechanism of Magnesium Isoglycyrrhizinate in regulating the inflammatory microenvironment of Gastric Cancer[D].The Second Military Medical University.(2017).
209	GC(Glycyrrhiza Uralensis	TP53（P53）	—	Glycyrrhiza Uralensis extract has no effect on p53 gene expression	Ma Jing, Pang Daben. Apoptosis of Human Gastric Cancer Cell Line MGC-803 Induced by Glycyrrhiza Uralensis Extract. Chinese

	extract）				Journal of Integrated Traditional and Western Medicine.20,928-930(2000).
210	GC(Diammonium glycyrrhizinate (DG)）	TP53（P53）	+1	Diammonium glycyrrhizinate (DG) has obvious cytotoxicity and proliferation inhibition effect on human hepatocellular carcinoma SMMC-7721. The mechanism may be related to up-regulation of p53 expression.	Zhang Jianfeng, Li Hao, Li Chaoqian, et al. Effect of diammonium glycyrrhizinate on proliferation in liver cancer cell SMMC-7721 and p53 expression. Chongqing Medicine.41, 2852-2853(2012)
211	GC(Glycyrrhizic acid）	TP53（P53）	-1	glycyrrhizic acid can slow down the process of liver fibrosis, and its molecular mechanism may be related to the down-regulation of p53 protein expression	Cao Qin, Cai Yu, Guo Xiaoling, et al. Effects of glycyrrhizic acid on the expression of p53 protein in rat liver fibrosis induced by CCl4.Chinese Journal of Digestion and Medical Imageology(Electronic Edition).16-19(2014).
212	GC(Glycyrrhizae radix extract）	BCL2	+1	Glycyrrhizae radix extract (GRE) blocks Cd-induced cell death by inhibiting the transfer of Bad to mitochondria, the reduction of mitochondrial Bcl (xL) and cytochrome c, and the cleavage of poly ADP ribose polymerase.	Kim SC, Byun SH, Yang CH, et al. Cytoprotective effects of Glycyrrhizae radix extract and its active component liquiritigenin against cadmium-induced toxicity (effects on bad translocation and cytochrome c-mediated PARP cleavage). Toxicology. 197,239-51(2004) .
213	GC(Licorice flavonoids from Glycyrrhiza uralensis）	BCL2	+1	licorice flavonoids from Glycyrrhiza uralensis can significantly improve the stress and depression behavior of CUS rats; the expression of Bcl-xl protein in hippocampus is significantly increased, and the expression of Caspase-3 protein is significantly reduced	Cheng Ruifeng, Hua Bing, Jing Jing, et al, .Modulation of the apoptotic protein expression in hippocampus is associated with the antidepressant effects of licorice flavonoids from Glycyrrhiza uralensis in rats.Pharmacology and Clinics of Chinese Materia Medica.30,69-72(2014).
214	GC(the total flavonoids from radix glycyrrhizae）	BCL2	-1	the total flavonoids from radix glycyrrhizae in each group can significantly inhibit the growth of S180 mouse sarcoma. Immunohistochemistry showed that the expression of Bcl-2 protein in mouse tumor tissue	Zhao Shiyuan, nongzhixin, Zhong Zhenguo, et al.Experimental study on antitumor effect of the total flavonoids from radix glycyrrhizae and its mechanisms.Guangxi Medical Journal.1496-1499(2006).

				was down-regulated, while the expression of Bax protein was up-regulated	
215	GC(Glycyrrhizin)	TGFB1(TGFβ1)	-1	1000μmol / L glycyrrhizin can inhibit TGF-β1 secreted by hepatic stellate cells in the supernatant	Dong Ling. Effects of glycyrrhizin on TGF-β / smad signal transduction in hepatic stellate cells in rats[D]. Fudan University.(2005).
216	GC(18α glycyrrhizin）	TGFB1(TGFβ1)	-1	18α GL can significantly reduce the transcription and protein levels of TGF-β1, Smad2, and Smad3 in liver fibrosis tissue. Immunohistochemistry also found that 18α GL can reduce the expression of phosphorylated Smad2, phosphorylated Smad3, and transcription factor SP-1 without With significant changes in Smad7	Qu Ying. Effects of 18α glycyrrhizin on liver collagen and TGFβ1 / Smad signaling pathway [D]. (2012).
217	GC(Diammonium glycyrrhizinate）	TGFB1(TGFβ1)	-1	Diammonium glycyrrhizinate can reduce the level of TGF-β1 secreted by AECII induced by PQ.	Xiang Li, Fang Chen, Zhou Zhibing, et al. Effects of diammonium glycyrrhizinate on the expression of TGF-β1 in alveolar epithelial cell IIcultured with paraquat. The Journal of Practical Medicine.034, 2320-2323,2328(2018).
218	GC(Gancaoganjiang Decotion）	TGFB1(TGFβ1)	-1	After 28 days of action, Gancao Ganjiang Decoction can significantly increase the activity of superoxide dismutase in rat lung tissue, reduce the content of reactive oxygen species, malondialdehyde and hydroxyproline, and at the same time, significantly downregulate TGF-β1, Smad3, α-SMA, ColI And SIRT1 protein expression and up-regulate Smad7 protein expression	Lu Guohui, Li yanru, Gao Jianmei. Gancaoganjiang Decotion inhibit bleomycin induced pulmonary fibrosis through regulating SIRT1 and TGF-β1 exprssion. Pharmacology and Clinics of Chinese Materia Medica. 25-28(2014).
219	GC(Guizhi Gancao Decoction）	TGFB1(TGFβ1)	-1	Guizhi Gancao Decoction can effectively protect myocardial function in rats with heart failure caused by coronary artery ligation, and its mechanism may be	Shen Dongdong. Effects of Guizhi Gancao Decoction on Myocardial Apoptosis and Expression of TGF-β1 and ICAM-1 in Myocardial Cells of Rats with Chronic Heart Failure. Chinese

				related to its anti-lipid oxidation, scavenging oxygen free radicals and down-regulating the expression of TGF-β1 and ICAM-1	Archives of Traditional Chinese Medicine.036, 932-935(2018).
220	GC(Glycyrrhizin）	TGFB1(TGFβ1)	-1	From the protein and mRNA levels, it was confirmed that glycyrrhizin has a significant inhibitory effect on the expression of TGFβ1 protein and mRNA, and has an early protective effect on rat glomerulosclerosis, which provides a basis for clinical application.	Huang Yihui, Yu Li, Zhang Lei,et al.Protective effects of glycyrrhizin on experimental transforming growth factorβ1.Progress in Modern Biomedicine.9,3262-3265+3192(2009).
221	GC(Mahuang-Gancao Couplet Medicines）	TGFB1(TGFβ1)	-1	MG can significantly reduce airway hyperresponsiveness, inhibit the expression of IL-4, IL-5 and IL-13 in BALF and lung tissue, reduce the expression of blood EOS and serum IgE, and reduce the inflammation and collagen deposition of lung tissue. Further results indicate that MG can inhibit the expression of TGF-β1 in lung tissue	Yuan Weiyuan, Wei pan, Bao Kaifan,et al.Effects and Mechanism of Mahuang-Gancao Couplet Medicines on Allergic Asthma.Journal of Nanjing University of Traditional Chinese Medicine.36,41-45(2020).
222	GC(Glycyrrhizic acid）	MAPK1(ERK2)	-1	Asthmatic mice have ERK1 / 2 and p38 MAPK signaling pathways activated, and glycyrrhizin can inhibit ERK1 / 2 and p38 MAPK signaling pathways in asthmatic mice	Zhang Wuyue, Gu Yongchun, Tang Ying, et al.Effects of glycyrrhizic acid on ERK1/2 and p38 MAPK signaling pathway in a murine model of asthma.National Medical Journal of China.98,1273-1278(2018).
223	GC(Magnesium isoglycyrrhizinate）	CXCL8（IL8）	-1	Magnesium isoglycyrrhizinate downregulates the expression of chemokines and their receptors CCL3, CCL5, CCL8, CCL11, CCL13, CCL19, CCL21, CXCL1, CXCL2, CXCL8, CCR1, CCR3, CCR4, CCR7, CXCR1, CXCR2 in gastric cancer tissues of mice; TLRs, MyD88, Tollip, NF-κb expression in mouse gastric cancer tissues; down-regulation of il1a,	Miao Yuqing. Anti-tumor mechanism of Magnesium Isoglycyrrhizinate in regulating the inflammatory microenvironment of Gastric Cancer[D].The Second Military Medical University.(2017).

				il1b, il1r1, il1rap, il1rn, il5, il6, il6r, il10, il10rb, il15, il17a, il18 in mouse gastric cancer tissues , Il22, il23a, il23r expression, up-regulated the expression of il2, il12	
224	GC(Prepared licorice decoction）	CRP	-1	Effect of Prepared licorice decoction on Coronary Heart Disease with Arrhythmia If it is accurate, it can effectively reduce the patient's serum hs-CRP, TNF-α, IL-8 s level.	Jin Cunwang.Prepared licorice decoction for the Treatment of Coronary Heart Disease with Arrhythmia .Considerations of Contemporary Medicine.17,192-193(2019).
225	GC(Isoliquiritigenin）	CCL2	-1	The increased expression of fibrosis markers (α-SMA, FN, COL-1) and related inflammatory factors (CCL2, CD68, F4 / 80, TNF-α, and IL-6) in the model group were different degrees in the treatment group reduce.	He Lin. molecular mechanism of isoliquiritigenin in ameliorating experimental mouse chronic pancreatitis[D].The Second Military Medical University.(2019）.
226	GC(Isoliquiritigenin）	MAPK8	-1	Compared with the control group, the isoliquiritigenin group significantly induced apoptosis of HCT116 cells in a concentration-dependent manner (P <0.05), significantly increased the expression of cleared caspase-3 protein (P <0.05), and significantly inhibited the invasion and migration of HCT116 cells (P <0.05 0.05), significantly down-regulate pJNK / JNK, pERK1 / 2 / ERK1 / 2, pP38 / P38 (P ＜ 0.05)	Wang Zhiguo, Huang Xianming, Zhao Yan, et al.Effects of isoliquiritigenin on proliferation, invasion and migration of colorectal cancer cells.Chinese Traditional Patent Medicine.41,1800-1805(2019).
227	GC(Licochalcone A and B）	FOS(C-FOS)	+1	After treatment with Licochalcone B from Glycyrrhizin uralensis, the expression of genes related to the death receptor pathway at the mRNA level (TNF, TNF-R1, Caspase8, Caspases 8, Fas, FasL, FOS, JUN) and at the protein level (TNF-R1, Fas , Caspases 8)	Wang Jun. Licochalcone A and B from Glycyrrhizin uralensis: Extraction, Anti-cancerous potential and the Molecular Mechanism [D].Hefei University of Technology.(2019).

228	GC(Licoricidin）	FOS(C-FOS)	-1	The expression is up-regulated Mechanistically, this appeared to be due to licoricidin-dependent inhibition of mitogen-activated protein kinases (MAPK) phosphorylation, which resulted in decreased c-Jun activation and reduced c-Jun and c-Fos expression.(#br)Conclusion(#br)Licoricidin blocks UVA -induced photoaging via ROS scavenging. This activity converges to limit the activity of MMP -1.	K. J. Kim,S. H. Xuan,S. N. Park. Licoricidin, an isoflavonoid isolated from Glycyrrhiza uralensis Fisher, prevents UVA -induced photoaging of human dermal fibroblasts. International Journal of Cosmetic Science.39,(2017).
229	GC(18β-glycyrrhetinic acid sodium (18β-SGA))	IL4	-1	18β-glycyrrhetinic acid sodium (18β-SGA) may play an anti-inflammatory role by inhibiting the activity of the NF-κB signaling pathway, reducing the expression of NF-κBp50, and down-regulating serum IL-4 levels	Li Li, Wang Youhu, Hou Yun, et al.Effects of 18beta-solidum glycrrhetinic acid on nuclear factor-kappaBp50 of nasal mucosa and IL-4 of serum in rats with allergic rhinitis.Journal of Xi'an Jiaotong University(Medical Sciences).41,58-63(2020).
230	GC(Licorzinc granules combined with loratadine tablets)	IL4	-1	Treatment of chronic urticaria patients with licorzinc granules combined with loratadine tablets can effectively reduce IL-4 and IL-10 levels and improve Th1 / Th2 cell imbalance	Zhang Li.Study on the Effect of Combined Use of Licorzinc Granules and Loratadine Tablets in Treating Chronic Urticaria on IL-4 and IL-10.Health Guide.221(2018).
231	GC(Magnesium isoglycyrrhizinate)	IL1AIL-1α)	-1	Magnesium isoglycyrrhizinate downregulates the expression of chemokines and their receptors CCL3, CCL5, CCL8, CCL11, CCL13, CCL19, CCL21, CXCL1, CXCL2, CXCL8, CCR1, CCR3, CCR4, CCR7, CXCR1, CXCR2 in gastric cancer tissues of mice; TLRs, MyD88, Tollip, NF-κb expression in mouse gastric cancer tissues; down-regulation of il1a, il1b, il1r1, il1rap, il1rn, il5, il6, il6r, il10, il10rb, il15,	Miao Yuqing. Anti-tumor mechanism of Magnesium Isoglycyrrhizinate in regulating the inflammatory microenvironment of Gastric Cancer[D].The Second Military Medical University.(2017).

				il17a, il18 in mouse gastric cancer tissues , Il22, il23a, il23r expression, up-regulated the expression of il2, il12	
232	GC(Glycyrrhizin (GL))	STAT1	-1	glycyrrhizin (GL) may block IFN-γ-mediated JAK / STAT1 signal activation, thereby inhibiting IFN-γ-induced HaXaT cell secretion of CXCL10	Xu Manyuan, min Zhongsheng, Wei Yuegang.Glycyrrhizin inhibits IFN-γ-induced CXCL10 by suppressing the JAK/STAT1 signal pathway in HaCaT cells.Chinese Journal of Cellular and Molecular Immunology.34,708-713(2018).
233	GC(Licorzinc granules)	EGFR	+1	Licorzinc granules can protect gastric tissue and accelerate ulcer healing by decreasing ulcer index of gastric ulcer rats model and increasing expressions of EGF and EGFR protein in the gastric tissue.	Deng Shaobo, Bai Guang.Effect of Licorzinc Granules on Ulcer Healing and Expressions of EGF and EGFR in Gastric Ulcer Model in Rats.Liaoning Journal of Traditional Chinese Medicine.37,556-559(2010).
234	GC(Shaoyao Gancao Decoction)	EGFR	+1	After treatment, the expression levels of EGFR and EGF in the gastric mucosa of the two groups of patients were higher than before treatment, P <0.05; the expression levels of EGFR and EGF in the gastric mucosa of the experimental group were higher than those of the reference group, P <0.05 .	Du Guofu.Analysis of the Effect of Shaoyao Gancao Decoction on Acute Gastric Ulcer.Contemporary Medical Symposium.16,138-140(2018).
235	GC(Isoliquiritigenin)	CXCL10	-1	In HepG2 and L02 cells, the expression levels of CXCL9, CXCL10, CXCL11 and IL-6 increased after treatment with IFN-γ. This high expression can be suppressed by ISL (isoglycyrrhizin) in a dose-dependent (IFN- γ + 5μg / mlISL group vs IFN-y induced group, P <0.05)	Wu Shanshan. The role of the isoliquiritigenin on the inflammation reaction of IFN-γ-induced hepatocytes and its mechanism[D].Zhejiang University.(2016).
236	GC(Licorice)	SOD1	-1	After adding licorice, the activities of superoxide dismutase, peroxidase and malondialdehyde in the liver of crucian carp were lower than those in the	Wang Shifeng, Xu Ping, gulimire anwair, et al.Effect of Licorice on Antioxidant Enzyme Activity in Hepatopancreas and Micronucleus Number in Red Blood Cell of Crucian Carp Carassius auratus

				copper-added group.	exposed to Cu2+ Stress.Fisheries Science.33,713-717(2014).
237	GC(Zhigancao decoction)	SOD1	+1	Zhigancao decoction can significantly increase the activity of SOD in blood (P <0.01) and reduce the content of MDA and ROS (P <0.01)	Yuan Jie.The Effects of Zhi Gan Cao Decoction on the Left Cardiac Function and Antioxidative Enzymes in Ischemia-Reperfusion Injured Rats.Lishizhen Medicine and Materia Medica Research.411-412(2008).
238	GC(Licorice extract)	PARP1	+1	Western blot results showed that with the increase of the action time of licorice extract (GC-3) (25μg / ml), PARP-1 was activated and sheared, and the expression level of its cleaved protein Cleaved-PARP-124 kD increased accordingly, and From 6 h, there is a significant difference from the normal control group (P <0.05), showing a significant time-effect relationship	Shang Xiaoyun, Li Xin, Yu Bo, et al.The studies on apoptosis of HeLa cells induced by licorice extract in vitro and related molecular mechanism.Chinese Journal of Clinicians(Electronic Edition).10798-10801(2013).
239	GC(18β-glycyrrhetinic acid)	CASP8	+1	Compared with the control group, cells in the G2 / M phase of the 18β-GA and A30 groups were significantly increased, caspase-8 protein level was significantly increased, and Bcl2 level was significantly decreased	Zhong Like.18β-glycyrrhetinic acid piperazine derivative A30 inhibits the proliferation of SMMC-7721 hepatoma cells.Chinese Journal of Cellular and Molecular Immunology.33,1212-1216(2017).
240	GC(Compound glycyrrhizin)	IFNG(IFN-γ)	-1	The level of IFN-γ in patients with alopecia areata was significantly higher than that in the healthy control group (P <0.05) .There was no significant difference in the level of IL-4 compared with the healthy control group. , IL-4 level did not change significantly before treatment	Teng Wei, Wei Li, Bi Chunxia,et al.Effect of compound glycyrrhizin on serum cytokines IFN-γ and IL-4 in patients with alopecia areata.China Journal of Leprosy and Skin Diseases.27,694-695(2011).
241	GC(Prepared Licorice Decoction)	IL2	+1	Prepared Licorice Decoction may promote the expression of IL-2 and IL-12 by inhibiting the expression of serum IL-1β and IL-6, regulate the immune function, reduce fatigue symptoms, and play	Liu Weicheng, Li Jiexuan, Guo Yongning,et al.Effects of Prepared Licorice Decoction on Behavioristics and 4 Kinds of IL in Rats with Chronic Fatigue Syndrome.Progress in Veterinary Medicine.39,70-73(2018).

				the role of treating chronic fatigue syndrome	
242	GC(Magnesium Isoglycyrrhizinate)	IL2	+1	Magnesium isoglycyrrhizinate downregulates the expression of chemokines and their receptors CCL3, CCL5, CCL8, CCL11, CCL13, CCL19, CCL21, CXCL1, CXCL2, CXCL8, CCR1, CCR3, CCR4, CCR7, CXCR1, CXCR2 in gastric cancer tissues of mice; TLRs, MyD88, Tollip, NF-κb expression in mouse gastric cancer tissues; down-regulation of il1a, il1b, il1r1, il1rap, il1rn, il5, il6, il6r, il10, il10rb, il15, il17a, il18 in mouse gastric cancer tissues , Il22, il23a, il23r expression, up-regulated the expression of il2, il12	Miao Yuqing. Anti-tumor mechanism of Magnesium Isoglycyrrhizinate in regulating the inflammatory microenvironment of Gastric Cancer[D].The Second Military Medical University.(2017).
243	GC	CAT	/		
244	GC(Water Extract of Glycyrrhiza uralensis)	IL10	+1	Water Extract of Glycyrrhiza uralensis can alleviate acute liver injury induced by triptolide, the mechanism may be related to down-regulation of pro-inflammatory factor TNF-α expression and up-regulation of anti-inflammatory factor IL-10	Zhu Shengnan, Zhang Jing, Tan relatives and friends,et al.Improvement Effects of Water Extract of Glycyrrhiza uralensis on Acute Hepatic Injury Caused by Triptolide and Its Effects on the Levels of IL-10 and TNF-α in Rats.China Pharmacy.30,216-220(2019).
245	GC(Glycyrrhizic Acid)	IL10	+1	Compared with the model group, different concentrations of glycyrrhizic acid can significantly increase the activities of SOD, GSH and CAT ($P < 0.01$), and significantly reduce the expression of IL-1β, IL-6, and TNF-α ($P < 0.05$, $P < 0.01$)	Zhang Lihong, Fu Yun, Wang yeqiu, et al.Protective Role of Glycyrrhizic Acid on Light Aging Cells of HaCaT and Its Influence to Inflammatory Cytokines.Information on Traditional Chinese Medicine.35,9-12(2018).
246	GC(Magnesium Isoglycyrrhizinate)	IL10	-1	Magnesium isoglycyrrhizinate downregulates the expression of chemokines and their receptors CCL3, CCL5, CCL8, CCL11, CCL13, CCL19, CCL21,	Miao Yuqing. Anti-tumor mechanism of Magnesium Isoglycyrrhizinate in regulating the inflammatory microenvironment of Gastric Cancer[D].The Second Military Medical

				CXCL1, CXCL2, CXCL8, CCR1, CCR3, CCR4, CCR7, CXCR1, CXCR2 in gastric cancer tissues of mice; TLRs, MyD88, Tollip, NF-κb expression in mouse gastric cancer tissues; down-regulation of il1a, il1b, il1r1, il1rap, il1rn, il5, il6, il6r, il10, il10rb, il15, il17a, il18 in mouse gastric cancer tissues , Il22, il23a, il23r expression, up-regulated the expression of il2, il12	University.(2017).
247	GC	HMOX1	/		
248	GC(the total flavonoids from radix glycyrrhizae)	BAX	+1	the total flavonoids from radix glycyrrhizae in each group can significantly inhibit the growth of S180 mouse sarcoma. Immunohistochemistry showed that the expression of Bcl-2 protein in mouse tumor tissue was down-regulated, while the expression of Bax protein was up-regulated	Zhao Shiyuan, nongzhixin, Zhong Zhenguo, et al.Experimental study on antitumor effect of the total flavonoids from radix glycyrrhizae and its mechanisms.Guangxi Medical Journal.1496-1499(2006).
249	LQ(methanol extract of forsythia fructus)	PTGS2(COX2)	+1	FFE inhibited COX-2 expression in LPS-stimulated RAW 264.7 cells.In addition, the expression levels of iNOS protein in RAW 264.7 cells were evaluated. As shown in Fig. 7, FFE effectively inhibited iNOS expression, which was in-line with its observed effects on NO production.	Lee SE, Lim C, Kim H, Cho S.A study of the anti-inflammatory effects of the ethyl acetate fraction of the methanol extract of forsythia fructus.Afr J Tradit Complement Altern Med 13, 102-113(2016).
250	LQ(methanol extract of forsythia fructus)	iNOS	+1	FFE inhibited COX-2 expression in LPS-stimulated RAW 264.7 cells.In addition, the expression levels of iNOS protein in RAW 264.7 cells were evaluated. As shown in Fig. 7, FFE effectively inhibited iNOS expression, which was in-line with its observed	Lee SE, Lim C, Kim H, Cho S.A study of the anti-inflammatory effects of the ethyl acetate fraction of the methanol extract of forsythia fructus.Afr J Tradit Complement Altern Med 13, 102-113(2016).

				effects on NO production.	
251	LQ	RELA(P65, NF-KB3)	-1	Forsythia 3. 5 g/kg can significantly inhibit rat feeding kaolin chemotherapy, improve the gastrointestinal mucosa pathological damage, reduce chemotherapy rats serum TNF-α, IL-1β, PGE2 levels, inflammatory markers and PGE2 by gastric antrum and ileum in the organization the NF-κB p65 and gastric antrum COX-2 positive expression	Meng Qi, Zhang Tian, Li Mengjiao et al.Study on the prevention and treatment of chemotherapy-induced nausea and vomiting by Forsythia suspensa and its anti-inflammatory activity.Pharmacol Clin Chin Mater Med 35, 125-130(2019).
252	LQ	TNF	-1	This study focused on the effect of antipyretic and detoxification Chinese medicine honeysuckle forsythiae on TNF-α induced SV40 MES 13 NF-κB signaling pathway, and explored the effective mechanism of antipyretic and detoxification Chinese medicine in the treatment of kidney disease.TNF-α significantly promoted the proliferation of SV40MES 13 ($P<0.05$ or $P<0.01$), and the drug treatment group inhibited the proliferation of SV40MES 13 ($P<0.01$).	Yao Yuanzhang, Zhang Min, Cao Peng. The Influence of the Heat -clearing and Detoxifying Herbs to NF -κB Signaling Pathway in the Glomerular Mesangial Cellsof Mice Induced by TNF-α. Chinese journal of integrated traditional and western medicine nephropathy 14, 1047-1050(2013).
253	LQ	IL6	-1	ELISA results showed that the protein expression of cytoplasm p-IκBα and nucleus NF-κBp65 was significantly decreased in the groups treated with different drugs(Honeysuckle Bud and Flower, forsythia fructus, Dandelion) ($p <0.01$), and the contents of McP-1 and Il-6 in the supernatant of cell culture were significantly reduced ($p <0.01$).	Yao Yuanzhang, Zhang Min, Cao Peng. The Influence of the Heat － clearing and Detoxifying Herbs to NF -κB Signaling Pathway in the Glomerular Mesangial Cellsof Mice Induced by TNF-α. Chinese journal of integrated traditional and western medicine nephropathy 14, 1047-1050(2013).
254	LQ(Forsythiaside A)	MAPK14(P38α)	-1	To investigate the effects of forsythiaside A(FSA) on airway inflammation in asthmatic mice, and to ex- plore	Lin Xing, Li Junfeng, Che Nan et al.Forsythiaside A suppresses airway inflammation of asthma through inhibition of p38 MAPK

				its possible mechanism of action.Conclusion: FSA could inhibit airway inflammation in asthmatic mice, and its mechanism may be related to inhibition of p38 MAPK/NF-κB signaling pathway.	/NF-kappa B signaling pathway.Chinese journal of immunology 35, 2971-2974+2979(2019).
255	LQ(Phillyrin)	CASP3	-1	The alcohol-induced hepatocyte injury was alleviated by concentration of forsythia .DAPI staining results showed that forsythia could significantly reverse alcohol-induced liver nucleus concentration and nuclear disintegration, and the expression of apoptosis-related proteins PARP and caspase 3 was also significantly inhibited.	Liu Yinhua, Qi Zhilin, Xu Guoxiang et al. Protective effect of phillyrin on alcoholic liver injury. Chinese clinical pharmacology and therapeutics 21, 6-9+15(2016).
256	LQ	PTGS1(COX1)	/		
257	LQ	ICAM1	/		
258	LQ(Forsythiaside A)	IL1B(IL1β)	-1	Test showed that LPS can induce chicken spleen lymphocytes IL-1β protein and mRNA expression quantity increases, and forsythiaside A group prevention chicken spleen lymphocytes IL-1β protein and mRNA expression decreased, show forsythiaside A can inhibit LPS through transcription and translation way in chicken spleen lymphocytes induced by IL-1β protein and mRNA expression quantity rise, reduce inflammation, exert anti-inflammatory function.	Cheng Guangdong, Zhang Qiang, Guan Nannan. Effect of forsythiaside A on IL-1β of spleen lymphocyte induced by endotoxin in chicken. Feed Research 10-13+17(2017).
259	LQ(Phillyrin)	TP53(P53)	-1	In the phillyrin(PHN)(2, 5, 10 mg/ml) group, the cell activity and the expression levels of PCNA,	Luo Shan, Huo Yongxin, Liu Ying et al.Effect of phillyrin on IL-1β induced apoptosis of human articular chondrocytes and

				BCL-2 and PARP proteins were significantly increased, and the apoptosis rate and the expression levels of p53, Bad, Bax and cl-caspase-3 were significantly decreased.	extracellular matrix degradation. Journal of Immunology 35, 645-652, 658(2019).
260	LQ(Forsythia extract)	BCL2	-1	Alcohol extract from forsythia root can up-regulate Bax, Bad and Noxa, and down-regulate the expression of Bcl-2, Bcl-xl and McL-1, thus inducing the apoptosis of esophageal cancer cells	Zhao Lianmei, Sun Jiawei, Yan Xi et al.Study on the anti-esophageal cancer effect of components extracted from different parts of forsythia in wuan county, hebei province.The fourth hospital of hebei medical university (2016).
261	LQ(Phillyrin)	TGFB1(TGFβ1)	-1	Forsythin inhibits inflammatory response in diabetic nephropathy rats, alleviates renal tissue damage, and has a protective effect on diabetic nephropathy rats, and the mechanism of action is related to inhibition of TGF-β1 expression.	Leng Wei, Liu Chunying, Shang Cheng et al.Potective effect and mechanism of phillyrin on diabetic nephropathy rats.Chinese journal of immunology 35, 2604-2608(2019).
262	LQ(ethanol extract of Forsythia suspensa root)	MAPK1	-1	The alcohol extract from forsythia root down-regulated the expression of p-JAK, p-STAT3, and p-ERK proteins in the JAK/STAT and ERK signaling pathways	Yan Xi.Experimental study on the antitumor and immunomodulatory effects of alcohol extract from forsythia root.Hebei: Hebei medical university (2012).
263	LQ(Phillyrin)	CXCL8(IL8)	-1	ELISA showed that when forsythin was 50 mg· L-, the levels of TNF- and IL-8 secreted by human mononuclear macrophages stimulated by staphylococcus aureus were significantly increased ($P < 0.05$), and the inhibitory effect was more obvious as the concentration of forsythin increased ($P < 0.001$, $P < 0.01$), showing a concentration-dependent relationship.	Wang Jiahe. Wan Xiaoxu. Liu Dan. The inhibitory effect of forsythin on the inflammatory response of human mononuclear macrophages stimulated by staphylococcus aureus. Journal of Xinxiang Medical College 33, 466-468(2016).
264	LQ(Qinlian	CRP	-1	The 118 patients with pneumonia were divided into the	Li Jing, Lv Zhichao, Ren Hongna. Effects of Qinlian Qiaopi

	Qiaopi Recipe)			observation group (Qinlian Qiaopi Recipe) and the control group (conventional western medicine). Compared with the two groups before treatment, serum levels of HsCRP, PCT, IL-8 and TNF-α decreased significantly after treatment (P <0. 05), and the observation group was significantly lower than the control group (P<0. 05 or P <0. 01).	Recipe Combined with Western Medicine on Blood Ｒoutine and Inflammatory Factors in Patients with Pneumonia.World Chinese Medicine14, 2737-2740(2019).
265	LQ	CCL2	—	Tannic acid, at nontoxic concentrations, specifically inhibited CXCL12-induced human monocyte migration (IC(50), 7.5 micro g/ml) but did not inhibit CCL2-, CCL3-, CCL5-, formylmethionylleucylphenylalanine (fMLP)-, or C5a-induced migration. The compound markedly blocked CXCL12 binding to THP-1 cells (IC(50), 0.36 micro g/ml). Tannic acid also inhibited CXCL12-induced, but not epidermal growth factor-induced, migration of MDA 231 breast tumor cells.	
266	LQ	MAPK8	/		
267	LQ(Forsythiaside A)	FOS(C-FOS)	-1	In vitro experiments, we found that Forsythiaside A inhibited osteoclast differentiation and bone resorption, and down-regulated the expression of osteoclast marker genes, including C-FOS and NFATC1	Sun Xuewu. Discussion on the mechanism of Forsythiaside A inhibiting osteoclast differentiation and its therapeutic application. Zhejiang University (2018).
268	LQ(Phillyrin)	IL4	1	Results: the glomerular swelling and basement membrane thickening of diabetic nephropathy rats were significantly reduced after treatment with phillyrin. The	Leng Wei, Liu Chunying, Shang Cheng et al.Potective effect and mechanism of phillyrin on diabetic nephropathy rats.Chinese journal of immunology 35, 2604-2608(2019).

				contents of urea nitrogen, creatinine, uric acid and urinary albumin were significantly reduced. The levels of interleukin 4(IL-4), IL-6, IL-18, IL-1, tumor necrosis factor α(TNF-α), and monocyte chemotactic protein 1(McP-1) in renal tissue were significantly increased.	
269	LQ	IL1A(IL-1 α)	/		
270	LQ(Forsythioside A)	STAT1	+1	In the treatment group of forsythioside A, the expression of IFN-γ was induced.In addition, changes in STATl protein expression were consistent with this, suggesting that forsythioside A induced IFN 1 May regulate the expression of smad7 by activating the STATl signaling pathway.	Zhao Hailei. Gao Lei. Lu Yan etal. Effect of forsythioside A induced IFN-γ and inhibition of TGF-β1/Smads signaling pathway on renal tubulointerstitial fibrosis.*Chinese Association of Animal Science and Veterinary Medicine* (2013).
271	LQ	EGFR	/		
272	LQ	CXCL10	/		
273	LQ(Forsythin)	SOD1(SOD)	+1	To study the antiaging effect of forsythin on aging model mice. Compared with the model group, the quality enhancement rate and spleen index of the mice in the high, medium and low dose forsythin groups increased. The activities of SOD, GSH-Px and T-AOC in serum and liver tissue of mice in the high and medium dose groups were enhanced, and the content of MDA was decreased.	Yan Liyou, Liu Mingjuan, Yan Huiru et al.Study on the anti-aging effect of forsythin in mice.Chinese pharmacy 26, 37-39(2015).
274	LQ	PARP1	/		
275	LQ	CASP8	/		

276	LQ	IFNG(IFN-γ)	/		
277	LQ(Jingjie Lianqiao Decoction)	IL2	+1	The amount of serum TNF-α in patients with UC significantly decreased and the level of IL-2 significantly increased with the modified Jingjie Lianqiao Decoction, and the difference in its regulatory effect was statistically significant compared with that of the control group (P<0.05)	Zhang Yanjun. Regulating effect of modified Jingjie Lianqiao Decoction on Serum TNF-α and IL-2 in Patients with Ulcerative Colitis. Shandong J Tradit Chin Med 35, 966-968(2016).
278	LQ(water extract from forsythia)	CAT	+1	ater extract from forsythia taishan can significantly improve the phagocytosis rate and phagocytosis index of aging model mice. Significantly reduced MDA content and increased GSH-Px, SOD and CAT activity	Li Teng, Li Yalin, He Hao et al. Study on the antiaging effect of water extract from forsythia taishan. Journal of tai shan Medical College 38, 1223-1225(2017).
279	LQ(Forsythia extract)	IL10	—	Forsythia suspense extract can promot proliferation of PLP135-151-specific lymphocytes and decreased the secretion of IFN-γ , but had no effect on the secretion of IL-10.	Xing Guangyu, Yin Ling.Effect of forsythia suspense extract on the proliferation and secretion of PLP135-151-specific T cell.China J Neuroimmunol & Neurol 19, 105-107+120(2012).
280	LQ(Forsythiaside A)	IL10	-1	Compared with the normal control group, the Treg, Foxp3, IL-10 and TGF-β1 of the endotoxemia model group improved significantly (P<0.01) .With the treatment of forsythiaside A, the abnormal changes reduced in a dose-dependent manner.Result of high doses of the drug was more obvious but similar to that of the control group (P >0.05) .Forsyth- iaside A has significant immunomodulatory effects and its therapeutic mechanism for endotoxemia may be related	Yuan Wei, Yang Hui, Xie Yong et al.Effect of forsythiaside A on regulatory T cells in mice with systemic endotoxemia.Chinese Traditional Patent Medicine 36, 1584-1588(2014).

				to the regulation of Treg, interference of Foxp3 gene expression and secretion of cytokine IL-10 and TGF-β1.	
281	LQ(Phillyrin)	HMOX1	+1	Compared with the young control group, the content of protein in urine increased significantly in the aged control group.The expression of Nrf 2, HO-1 and NQO- 1 decreased significantly(P<0.01).	Yang Jieling, Li Shen, Liu Qing et al.Effects of forsythoside on oxidative stress and renal function in aged rats. Chinese journal of clinical anatomy 37, 77-82(2019).
282	LQ(Total flavones in forsythia)	BAX	+1	The results showed that Forsythia suspense flavonoids(FF) significantly inhibited the proliferation and colony formation of MGC80-3 cells, and its molecular mechanism was that FF down-regulated the protein level of mTOR and up-regulated the expressions of Bax as well as cellular autophagy factor Beclin 1 and LC3II, which promoted the autophagic cell death, then inhibiting the survival of MGC80-3 cells. Therefore, FF possesses the potential value fordeveloping anti-tumor drug.	Li Ping, Zhang Guiping, Hu Jianran. Effects of Total Flavonoids from Forsythia suspense on the Proliferation of Gastric Cancer Cell MGC80-3. Biotechnology bulletin 34, 199-203(2018).
283	MH	PTGS2(COX2)	-1	Reporter gene assays showed that Mao reduced LPS-inducible NF-kappaB-dependent transcription that plays a crucial role in induction of COX-2 gene expression.	Aoki K, Yamakuni T, Yoshida M, Ohizumi Y. Ephedorae herba decreases lipopolysaccharide-induced cyclooxgenase-2 protein expression and NF-kappaB-dependent transcription in C6 rat glioma cells. J Pharmacol Sci 98, 327–330(2005).
284	MH	PTGS2(COX2)	-1	Five mostly studied herbs, including Ephedra Herba, Amygdalus communis Vas, Platycodon grandiforus , Licorice and Scutellariae Radix, were selected from the literature.Of importance, we found that TNF, β2AR and PTGS2 play pivotal role in TCM mediated	Jian hong Sun, Fei Sun, Bin Yan, Jun yi Li, De li Xin. Data mining and systematic pharmacology to reveal the mechanisms of traditional Chinese medicine in Mycoplasma pneumoniae pneumonia treatment. Biomedicine & Pharmacotherapy 125(2020).

285	MH(Ephedra methanol extract)	iNOS	-1	MPP inhibition. In the case of COX2, the mRNA levels were not significantly lowered in response to either of the utilized aqueous extract concentrations. Interestingly, about 50–60% of COX2 mRNA levels were lost in response to either of the added ethanolic extract concentrations. However, the methanolic extract was less efficient, as only about 15% of COX2 mRNA levels were absent in response to 50 μg/ml but not 100 μg/ml of extract . In the case of iNOS, the 3 different extracts were efficient in terms of impairing iNOS transcription, with methanolic extract being the most efficient, followed by ethanolic extract, and finally aqueous extract.	Kallassy H, Fayyad-Kazan M, Makki R, et al. Chemical Composition and Antioxidant, Anti-Inflammatory, and Antiproliferative Activities of Lebanese Ephedra Campylopoda Plant. Med Sci Monit Basic Res 23, 313-325(2017).
286	MH	RELA(P65)	-1	The protein content of NF-κB p65 decreased in high and low dose groups of ephedra.	Yangping, Jin Suan, Che Lijuan et al. Study on the expression changes of TLR2 and NF-κB in mice with lung heat syndrome after 4 kinds of traditional Chinese medicines.China Journal of Chinese Materia Medica 39, 3359-3362(2014).
287	MH	TNF	-1	Ephedra, dried ginger, scutellaria baicalensis and white mulberry skin can interfere with lung heat syndrome and inhibit the expression of TNF-α, IL-1β inflammatory cytokines, which may be the main ways to reduce lung tissue damage caused by lung heat syndrome in mice.	Yangping, Jin Suan, Che Lijuan et al.Effects of Four Chinese Herbs Belong to Lung on Expression of TNF-α and IL-1β in Mice with Lung Heat Syndrome.Chinese journal of experimental formulae 20, 162-166(2014).
288	MH(Mahuang Lianqiao Chidou	IL6	-1	After treatment, ser- um IL-6, IL-8, TNF-α and hs-CRP levels were significantly lower than those before	Jiang Wei, Xue Qin, Li Jie et al. Clinical Efficacy of Addition and Subtraction of Mahuang Lianqiao Chidou Decoction in

	Decoction)			treatment in two groups, and the lev- els in observation group were significantly lower than those in control group (P<0.05).	Treatment of Patients with Damp-heat Cough Syndrome.PLA medical journal 31, 85-88(2019).
289	MH	MAPK14(P38α)	-1	Altogether 23 active ingredients were screened and 156 targets were obtained.Epidermal active growth factor receptor (EGFR) , E-selectin (SELE) , macro- phage migration inhibitory factor (MIF) , mitogen-activated protein kinase 14 (MAPK14) might be the important anti-inflammatory targets of ephedra treatment of asthma. All these pathways had epithelial cell signaling pathways.	Chen ou, Li Guoyong, Liu Aihong. Anti-inflammatory mechanism of ephedra treatment of asthma based on network pharmacology. Journal of shandong university: medical science 57, 69-75(2019).
290	MH	CASP3	/		
291	MH	PTGS1(COX1)	-1	Ethanol extracts of Artemisia persica (IC50: 0.5μg/mL),Dragocephalum paulsenii (IC50: 0.5 μg/mL), Ephedra intermedia (IC50: 3.8 μg/mL), Hyoscyamus pusillus, Nepeta parmiriensis (IC50:0.7μg/mL)and Rumex patientia subsp.pamiricus (IC50: 3.5 μg/mL) exhibited the best COX-1 inhibitory effect (Table 2).	Jeppesen AS, Soelberg J, Jäger AK. Antibacterial and COX-1 Inhibitory Effect of Medicinal Plants from the Pamir Mountains, Afghanistan. Plants (Basel) 1, 74-81(2012).
292	MH	ICAM1	-1	C3 positive expression, MPO activity, and ICAM-1 mRNA level were significantly weaker in the Ephedra sinica group than in the control group at all time points (12 h, day 1, day 3, day 7, and day 14 after SCI) (P < 0.01, P < 0.05).	Li LM, Zhu Y. Zhongguo Zhong Xi Yi Jie He Za Zhi. 2012;32(10):1385–1389.
293	MH(cassia twig and ephedra	IL1B(IL1β)	-1	ELISA showed that TNF-α, IL-6 and IL-1β expression levels in the prefrontal cortex and hippocampus were	Xu Liangkui. The mechanisms of post-ischemic neuroinflammation regulated by Ramulus Cinnamomi-Herba Ephedrae based on

				significantly increased after modeling (P <0.05) however, by osmanthus twig - ephedra (6 and 12g/kg, P<0.05) significant reduction of TNF-α, IL-6 and IL-1β levels after treatment suggested that cassia twig - ephedra (6 and 12 g/kg, i.g.) significantly inhibited the rise of TNF-α, IL-6 and IL-1β levels	TLR4/MyD88/MAPK pathway. Southern Medical University (2018).
294	MH	TP53(P53)	/		
295	MH	BCL2	/		
296	MH	TGFB1(TGFβ1)	/		
297	MH(Modified Shegan Mahuang Decoction)	MAPK1	-1	The mechanism of inhibiting airway remodeling by adding or subtracting ephedrine decoction may be to reduce the level of PCNA and inhibit the expression levels of ERK, p-ERK and PCNA proteins and inhibit the proliferation of airway smooth muscle, so as to improve the airway remodeling in asthmatic rats	Wang Jing. Zhang Bo. Effects of Modified Shegan Mahuang Decoction on Airway Remodeling and PCNA and ERK in Lung Tissue of Asthmatic Rats.Journal of emergency in traditional chinese medicine 27, 950-95(2018).
298	MH(Ephedrine)	CXCL8(IL8)	-1	Ephedrine can inhibit the expression and secretion of IL-8 in 16HBE induced by TNF-α, which may be one of the mechanisms of ephedrine in the treatment of asthma.	Li Zhongyan, Deng Bing, Xiong Bin et al. Effect of ephedrine on expression of interleukin - 8 in human bronchial epithelial cells.Shandong Medical Journal 56, 950-954(2018).
299	MH	CRP	-1	Seventy-three patients in the combined group were treated with Mahuang decoction plus budesonide, In the single drug group, 70 patients were treated with budesonide alone. The PCT value and hs-crp value of the combined group were lower than that of the single	

				drug group	
300	MH	CCL2(monocyte chemotactic protein-1)	-1	Meanwhile, EsM transplantation significantly reduced gene expression of proinflammatory cytokines interleukin-1 and monocyte chemotactic protein-1.	Wang JH, Kim BS, Han K, Kim H. Ephedra-Treated Donor-Derived Gut Microbiota Transplantation Ameliorates High Fat Diet-Induced Obesity in Rats. Int J Environ Res Public Health 14, 555(2017).
301	MH	MAPK8	/		
302	MH(Ephedrine)	FOS(C-FOS)	+1	The mean absorbance value of C-FOS and caspase-3 protein expression in the lung tissues of the ephedrine group was significantly increased.	Peng Jing, Li Chongyang, liu Tingting et al. Effects of ephedrine on lung tissue structure in mice. Acta Anatomica Sinica 47, 521-527(2016).
303	MH	IL4	-1	Ephedra and asarum are both effective drugs in xiaoqinglong decoction, which can reduce the level of IL-4 and increase the level of IFN-γ.	Shao Lijie, Tang Fang. Effects of ephedra and asarum on allergic rhinitis in rats. Information on Traditional Chinese Medicine 36, 47-49(2019).
304	MH	IL1AIL-1α)	/		
305	MH（water extract of ephedra）	STAT1	—	Ephedrine water extract has an obvious regulatory effect on airway inflammation in asthmatic guinea pigs, and its mechanism may be related to the inhibition of il-5 expression and inflammatory cell accumulation, but ephedrine cannot effectively inhibit the expression of STAT1 and abnormal signal transduction.	Xiong Ying, Xiong Bin, Wang Songping et al. The effect of aqueous extract of ephedra on the airway inflammation and epithelial STAT1 expression of guinea pig with asthma.Med J West China 41, 2206-2211(2018).
306	MH（Ephedra and Bitter Apricot Seed）	EGFR	-1	Ephedrine - almond medicine has the best effect on the down-regulation of EGFR mRNA in medicated intestinal absorption solution. N6A showed the best effect in down-regulating the expression of PI3K mRNA, EGFR protein and the total cell damage rate.	Xu Zhao, Wang Zhan, Zhou Kaifang et al. Effects of ephedrine - almond on the expression of EGFR and PI3K in histamine rat model of airway epithelial cell damage.Journal of Chinese Medicinal Materials 41, 2206-2211(2018).

307	MH	CXCL10	/		
308	MH（Ephedrine）	SOD1	±1	The activity of SOD and CAT in the lung tissues of ephedrine group was temporarily increased at 5d and significantly decreased at 10d and 15d.	Peng Jing, Li Chongyang, liu Tingting et al. Effects of ephedrine on lung tissue structure in mice. Acta Anatomica Sinica 47, 521-527(2016).
309	MH	PARP1	/		
310	MH	CASP8	/		
311	MH	IFNG(IFN -γ)	/		
312	MH(Mahuangjiazhu Decoction)	IL2	+1	Both ephedrine and ribavirin significantly increased the content of IL-2 and IFN-γ in mice ($P<0.001$), but there was no significant difference in the content of IL-2 between the Chinese and western groups.	Li Junlian, li Yanyan, Gao Peng et al. Effect of Mahuangjiazhu decoction on serum IL-2 and IFN-γ in mice infected with respiratory syncytial virus.Chinese journal of experimental formulae 19, 196-199(2013).
313	MH(Ephedrine)	CAT	±1	The activity of SOD and CAT in the lung tissues of ephedrine group was temporarily increased at 5d and significantly decreased at 10d and 15d.	Peng Jing, Li Chongyang, liu Tingting et al. Effects of ephedrine on lung tissue structure in mice. *Acta Anatomica Sinica* 47, 521-527(2016).
314	MH(Ephedrine)	CAT		The plasma activity of CK and CK-MB in ephedrine group was higher than that in control group ($P < 0.01$), and the activity of T-AOC and CAT in cardiac tissue was decreased.	Liu Tingting, Peng Jing, Li Chongyang. Effects of ephedrine on histological structure, activities of total antioxidant capacity and catalase in the myocardial tissue of mice.*Acat anatomica sinica* 47, 516-520(2016).
315	MH(Ephedrine)	CAT	-1	Compared with the normal saline group, the contents of MDA and NO in the frontal cortex of the ephedrine group were significantly increased, and the activities of SOD, CAT and GSH-PX were significantly decreased.	Zheng Fanghao, Luo Jiabo. Ma Huang (Ephedra) induces oxidative stress in the rat prefrontal cortex. *Lishizhen Med Mater Med Res* 27, 1313-1316(2016).
316	MH(Shegan Mahuang	IL10	+1	Shegan Mahuang Decoction has certain antagonism on airway inflammation in asthmatic mice.	Sui Bowen, Li Minghu, Zhai Pingpinget al. Effect of Shegan Mahuang Decoction on Asthma Mouse Model of Airway

	Decoction)			Its mechanism may be related to the decrease of serum IL-6 and increase IL-10 level.	Inflammation and Serum IL-6and IL-10 Levels.*Journal of emergency in traditional chinese medicine* 26, 783-785+822(2017).
317	MH	HMOX1	/		
318	MH(Ephedrine)	BAX	+1	The expression intensity of Bax protein in the heart of ephedrine group was significantly higher than that of the control group, ephedrine can promote apoptosis of cardiomyocytes	Liu Tingting. Effects of honeysuckle solution on cardiac injury induced by ephedrine in mice.Journal of northwest normal university (2013).
319	XR(Amygdalin)	PTGS2	-1	The present results showed that amygdalin suppressed the prostaglandin E(2) synthesis and the nitric oxide production by inhibiting the LPS-stimulated mRNA expressions of COX-2 and iNOS in the mouse BV2 cells.	Yang HY, Chang HK, Lee JW, et al. Amygdalin suppresses lipopolysaccharide-induced expressions of cyclooxygenase-2 and inducible nitric oxide synthase in mouse BV2 microglial cells. *Neurol Res*. 29, S59–S64(2007).
320	XR	iNOS	-1	Besides chromatographic analysis, cell culture experiments were performed using murine macrophages (RAW264.7) to study the capacity of different nut extracts (hazelnut, almond, walnut, macadamia, and pistachio) to modulate inflammatory processes. Oleic acid was the main fatty acid in hazelnut, almond, macadamia, and pistachio extracts. Both oily nut extracts and pure oleic acid significantly reduced the LPS-induced expression of iNos, Cox2, Tnfα, Il1β, and Il6 mRNAs. iNos protein expression was down-regulated followed by reduced nitric oxide formation. Thus, nut extracts at concentrations achievable in the digestive tract inhibit the expression and formation of inflammatory	Müller Anke Katharina, Schmölz Lisa, Wallert Maria, Schubert Martin, Schlörmann Wiebke, Glei Michael, Lorkowski Stefan. In Vitro Digested Nut Oils Attenuate the Lipopolysaccharide-Induced Inflammatory Response in Macrophages. Nutrients 11(2019).

				mediators in macrophages.	
321	XR	RELA(P65)	-1	Chlorogenic acid dose-dependently suppressed IL-1beta-induced mRNA expression of vascular cell adhesion molecule-1, intercellular cell adhesion molecule-1 and endothelial cell selectin. Chlorogenic acid also suppressed the IL-1beta-induced production of ROS. We also observed that chlorogenic acid attenuated or blocked IL-1beta-induced nuclear translocation of nuclear factor-kappaB subunits p50 and p65, which in turn attenuated CAM expression at the transcription level. Furthermore, chlorogenic acid significantly reduced the adhesion of human monocyte cells (U937) to IL-1beta-treated HUVECs in a dose-response manner. These results are similar to that of probucol.	Chang WC, Chen CH, Lee MF, Chang T, Yu YM. Chlorogenic acid attenuates adhesion molecules upregulation in IL-1β treated endothelial cells. Eur J Nutr 49, 267–275(2010).
322	XR(Almond skin polyphenol extract)	TNFα	-1	The proinflammatory mediators monocyte chemotactic protein-1 (MCP-1) and chemokine ligand 5 (CCL-5) were determined by enzyme-linked immunosorbent assay. We found that ASP significantly promoted phosphorylation of AMP-activated protein kinase (AMPK), increased activity of adipose triglyceride lipase and hormone-sensitive lipase, and inhibited adipogenesis-related transcription factors. In addition, ASP inhibited the tumor necrosis factor-α (TNF-α)-induced cell inflammatory response via downregulation of MCP-1 and CCL-5 secretion. This	Huang WC, Chen CY, Wu SJ. Almond Skin Polyphenol Extract Inhibits Inflammation and Promotes Lipolysis in Differentiated 3T3-L1 Adipocytes. *J Med Food* 20, 103-109(2017).

				study suggests that ASP regulates lipolysis through activation of AMPK, reduced adipogenesis, and suppresses proinflammatory cytokines in adipocytes.	
323	XR(Almond skin polyphenol extract)	TNFα	-1	Besides chromatographic analysis, cell culture experiments were performed using murine macrophages (RAW264.7) to study the capacity of different nut extracts (hazelnut, almond, walnut, macadamia, and pistachio) to modulate inflammatory processes. Oleic acid was the main fatty acid in hazelnut, almond, macadamia, and pistachio extracts. Both oily nut extracts and pure oleic acid significantly reduced the LPS-induced expression of iNos, Cox2, Tnfα, Il1β, and Il6 mRNAs. iNos protein expression was down-regulated followed by reduced nitric oxide formation. Thus, nut extracts at concentrations achievable in the digestive tract inhibit the expression and formation of inflammatory mediators in macrophages.	Müller Anke Katharina, Schmölz Lisa, Wallert Maria, Schubert Martin, Schlörmann Wiebke, Glei Michael, Lorkowski Stefan. In Vitro Digested Nut Oils Attenuate the Lipopolysaccharide-Induced Inflammatory Response in Macrophages. *Nutrients* 11(2019).
324	XR（Almond skin polyphenol extract）	TNFα	-1	Exercise also significantly increased IL6, IL8, VEGF, INFγ, TNFα, IL1α, IL1β, MCP1, and EGG production rates by LPS-stimulated PBMCs, and this response was attenuated by DHA supplementation.	Capó X, Martorell M, Sureda A, Batle JM, Tur JA, Pons A. Docosahexaenoic diet supplementation, exercise and temperature affect cytokine production by lipopolysaccharide-stimulated mononuclear cells. J Physiol Biochem 72, 421–434(2016).
325	XR	TNFα	-1	NS induced a significant decrease in HSV-2 replication, whereas extracts obtained from BS did not significantly influence the viral replication. High levels of cytokines	Arena A, Bisignano C, Stassi G, Mandalari G, Wickham MS, Bisignano G. Immunomodulatory and antiviral activity of almond skins. Immunol Lett 132, 18-23 (2010).

				production, such as IFN-alpha (38+/-5.3 pg/ml), IL-12 (215+/-17.1 pg/ml), IFN-gamma (5+/-0.7 IU/ml), TNF-alpha (3940+/-201.0 pg/ml), were detected. Moreover, IL-10 (210+/-12.2 pg/ml) and IL-4 (170+/-21.4 pg/ml), representative of Th2 responses, were found.	
326	XR	TNF	-1	High dose Apricot Kernel could effectively inhibit articular swelling , decrease inflammatory in tissue of AA rats and pull down the levels of TNF-α and sICAM -1.	Zhou Xiaotao, Fulati·Rexiti, An Huawei et al. Research on antiinflammatory mechanism of Apricot Kernel in rat with adjuvanticity arthritis.Modern Journal of Integrated Traditional Chinese and Western Medicine 20, 4198-4199(2011).
327	XR	IL6	-1	Of serum inflammatory markers, IL-10 was decreased by almond intake ($P \leq 0.05$), and ICAM-1, IL-1β, and IL-6 tended to be lower with almonds, compared to the cookies.	Jung H, Chen CO, Blumberg JB, Kwak HK. The effect of almonds on vitamin E status and cardiovascular risk factors in Korean adults: a randomized clinical trial. Eur J Nutr 57, 2069–2079(2018).
328	XR	IL6	-1	Besides chromatographic analysis, cell culture experiments were performed using murine macrophages (RAW264.7) to study the capacity of different nut extracts (hazelnut, almond, walnut, macadamia, and pistachio) to modulate inflammatory processes. Oleic acid was the main fatty acid in hazelnut, almond, macadamia, and pistachio extracts. Both oily nut extracts and pure oleic acid significantly reduced the LPS-induced expression of iNos, Cox2, Tnfα, Il1β, and Il6 mRNAs. iNos protein expression was down-regulated followed by reduced nitric oxide formation. Thus, nut extracts at	Müller Anke Katharina, Schmölz Lisa, Wallert Maria, Schubert Martin, Schlörmann Wiebke, Glei Michael, Lorkowski Stefan. In Vitro Digested Nut Oils Attenuate the Lipopolysaccharide-Induced Inflammatory Response in Macrophages. Nutrients 11(2019).

				concentrations achievable in the digestive tract inhibit the expression and formation of inflammatory mediators in macrophages.	
329	XR	IL6	-1	Exercise also significantly increased IL6, IL8, VEGF, INFγ, TNFα, IL1α, IL1β, MCP1, and EGG production rates by LPS-stimulated PBMCs, and this response was attenuated by DHA supplementation.	Capó X, Martorell M, Sureda A, Batle JM, Tur JA, Pons A. Docosahexaenoic diet supplementation, exercise and temperature affect cytokine production by lipopolysaccharide-stimulated mononuclear cells. J Physiol Biochem 72, 421–434(2016).
330	XR(Amygdalin)	MAPK14(P38α)	+1	The effect of amygdalin on LPS-induced RAW264.7 inflammation model in mouse peritoneal macrophages may be related to inhibiting the expression of inflammatory cytokines IL-17A, IL-23, and chemokines CCL2, CCL5, and the excessive activation of NF-κB and p38MAPK signal pathways.	Zhong Xiaoqin, Li Leng, Lu Chuanjian et al. Anti-inflammation Effect of Amygdalin on Macrophage 264.7 Cells Stimulated by Lipopolysaccharide.Tradit Chin Drug Res Pharmacol 29, 257-263(2018).
331	XR	CASP3	+1	Fermentation supernatants (FS) of raw and roasted almonds had no genotoxic effects. FS obtained from raw or mildly roasted almonds (R1) significantly increased mRNA levels of CAT (4.6-fold), SOD2 (5.6-fold) and GSTP1 (3.9-fold) but not of GPx1. FS of almonds significantly reduced the growth of LT97 cells in a time- and dose-dependent manner. Treatment with 5% almonds FS increased the number of early apoptotic cells (17.4%, on average) and caspase-3 activity (4.9-fold, on average). The results indicate a chemopreventive potential of in vitro-fermented almonds which is largely independent of the roasting	Schlörmann W, Fischer S, Saupe C, Dinc T, Lorkowski S, Glei M. Influence of roasting on the chemopreventive potential of in vitro fermented almonds in LT97 colon adenoma cells. Int J Food Sci Nutr 69, 52-63(2018).

				process. Amygdalin without zinc showed a strong anti-HepG2 activity. Furthermore, HepG2 cell lines treatment with amygdalin + 20μmol zinc and amygdalin + 800μmol zinc showed a highly significant apoptotic effect than the effect of amygdalin without zinc. Amygdalin treatment induced the cell cycle arrest at G2/M and increased the levels of P53, Bax, cytochrome c, and caspase-3 significantly, while it decreased the level of anti-apoptotic Bcl2.	El-Desouky MA, Fahmi AA, Abdelkader IY, Nasraldin KM. Anticancer effect of Amygdalin (vitamin B17) on hepatocellular carcinoma cell line(HepG2) in the presence and absence of Zinc . Anticancer Agents Med Chem 2020.
332	XR(Amygdalin)	CASP3	-1		
333	XR	PTGS1(COX1)	/		
334	XR	ICAM1	-1	Of serum inflammatory markers, IL-10 was decreased by almond intake (P ≤ 0.05), and ICAM-1, IL-1β, and IL-6 tended to be lower with almonds, compared to the cookies.	Jung H, Chen CO, Blumberg JB, Kwak HK. The effect of almonds on vitamin E status and cardiovascular risk factors in Korean adults: a randomized clinical trial. Eur J Nutr 57, 2069–2079(2018).
335	XR（Natural almond skin）	ICAM1	-1	NS powder also reduced NF-κB and p-JNK activation, the pro-inflammatory cytokines release, the appearance of i-NOS, nitrotyrosine and PARP in the colon and reduced the up-regulation of ICAM-1 and the expression of P-selectin.	Mandalari G, Bisignano C, Genovese T, et al. Natural almond skin reduced oxidative stress and inflammation in an experimental model of inflammatory bowel disease. Int Immunopharmacol 11, 915-924(2011).
336	XR	IL1B(IL1β)	-1	Of serum inflammatory markers, IL-10 was decreased by almond intake (P ≤ 0.05), and ICAM-1, IL-1β, and IL-6 tended to be lower with almonds, compared to the cookies.	Jung H, Chen CO, Blumberg JB, Kwak HK. The effect of almonds on vitamin E status and cardiovascular risk factors in Korean adults: a randomized clinical trial. Eur J Nutr 57, 2069–2079(2018).
337	XR	IL1B(IL1	-1	Besides chromatographic analysis, cell culture	Müller Anke Katharina, Schmölz Lisa, Wallert Maria, Schubert

		β)		experiments were performed using murine macrophages (RAW264.7) to study the capacity of different nut extracts (hazelnut, almond, walnut, macadamia, and pistachio) to modulate inflammatory processes. Oleic acid was the main fatty acid in hazelnut, almond, macadamia, and pistachio extracts. Both oily nut extracts and pure oleic acid significantly reduced the LPS-induced expression of iNos, Cox2, Tnfα, Il1β, and Il6 mRNAs. iNos protein expression was down-regulated followed by reduced nitric oxide formation. Thus, nut extracts at concentrations achievable in the digestive tract inhibit the expression and formation of inflammatory mediators in macrophages.	Martin, Schlörmann Wiebke, Glei Michael, Lorkowski Stefan. In Vitro Digested Nut Oils Attenuate the Lipopolysaccharide-Induced Inflammatory Response in Macrophages. Nutrients 11(2019).
338	XR	IL1B(IL1 β)	-1	Chlorogenic acid dose-dependently suppressed IL-1beta-induced mRNA expression of vascular cell adhesion molecule-1, intercellular cell adhesion molecule-1 and endothelial cell selectin. Chlorogenic acid also suppressed the IL-1beta-induced production of ROS. We also observed that chlorogenic acid attenuated or blocked IL-1beta-induced nuclear translocation of nuclear factor-kappaB subunits p50 and p65, which in turn attenuated CAM expression at the transcription level. Furthermore, chlorogenic acid significantly reduced the adhesion of human monocyte cells (U937) to IL-1beta-treated HUVECs in a	Chang WC, Chen CH, Lee MF, Chang T, Yu YM. Chlorogenic acid attenuates adhesion molecules upregulation in IL-1β treated endothelial cells. Eur J Nutr 49, 267–275(2010).

				dose-response manner. These results are similar to that of probucol.	
339	XR	IL1B(IL1β)	-1	Exercise also significantly increased IL6, IL8, VEGF, INFγ, TNFα, IL1α, IL1β, MCP1, and EGG production rates by LPS-stimulated PBMCs, and this response was attenuated by DHA supplementation.	Capó X, Martorell M, Sureda A, Batle JM, Tur JA, Pons A. Docosahexaenoic diet supplementation, exercise and temperature affect cytokine production by lipopolysaccharide-stimulated mononuclear cells. J Physiol Biochem 72, 421–434(2016).
340	XR(Amygdalin)	TP53（P53）	+1	Amygdalin without zinc showed a strong anti-HepG2 activity. Furthermore, HepG2 cell lines treatment with amygdalin + 20μmol zinc and amygdalin + 800μmol zinc showed a highly significant apoptotic effect than the effect of amygdalin without zinc. Amygdalin treatment induced the cell cycle arrest at G2/M and increased the levels of P53, Bax, cytochrome c, and caspase-3 significantly, while it decreased the level of anti-apoptotic Bcl2.	El-Desouky Mohamed A, Fahmi Abdelgawad A, Abdelkader Ibrahim Y et al. Anticancer effect of Amygdalin (vitamin B17) on hepatocellular carcinoma cell line(HepG2) in the presence and absence of Zinc. Anticancer Agents Med Chem (2020).
341	XR(Amygdalin)	BCL2	-1	Amygdalin without zinc showed a strong anti-HepG2 activity. Furthermore, HepG2 cell lines treatment with amygdalin + 20μmol zinc and amygdalin + 800μmol zinc showed a highly significant apoptotic effect than the effect of amygdalin without zinc. Amygdalin treatment induced the cell cycle arrest at G2/M and increased the levels of P53, Bax, cytochrome c, and caspase-3 significantly, while it decreased the level of anti-apoptotic Bcl2.	El-Desouky MA, Fahmi AA, Abdelkader IY, Nasraldin KM. Anticancer effect of Amygdalin (vitamin B17) on hepatocellular carcinoma cell line(HepG2) in the presence and absence of Zinc . Anticancer Agents Med Chem 2020.
342	XR(Amygdalin)	TGFB1(T	-1	Amygdalin group had a significant inhibitory effect on	Luo Huanhuan. Effect of amygdalin on activation and proliferation

		GFβ1)		TGF-βm RNA and CTGFm RNA	of HSC and its related cytokine network.Traditional Chinese Medicine University Of Guangzhou (2010).
343	XR	MAPK1	/		
344	XR	CXCL8（IL8）	/		
345	XR	CRP	/		
346	XR	CCL2	/		
347	XR	MAPK8	/		
348	XR	FOS(C-FOS)	/		
349	XR(Amygdalin)	IL4	-1	Infiltration of inflammatory cells such as eosinophilic cells, lymphocytes and macrophages in the tracheal submucosa of each dose of bitter almond group showed a decreasing trend, and significantly reduced the content of IL-4 in alveolar lavage fluid.	Wei Hao, Xu Dong, Yao Dongfeng et al. Effects of amygdalin on airway inflammation in mice with allergic asthma.Shanxi J Tradit Chin Med 37, 1691-1693(2016).
350	XR(Almond skins)	IL4	+1	NS induced a significant decrease in HSV-2 replication, whereas extracts obtained from BS did not significantly influence the viral replication. High levels of cytokines production, such as IFN-alpha (38+/-5.3 pg/ml), IL-12 (215+/-17.1 pg/ml), IFN-gamma (5+/-0.7 IU/ml), TNF-alpha (3940+/-201.0 pg/ml), were detected. Moreover, IL-10 (210+/-12.2 pg/ml) and IL-4 (170+/-21.4 pg/ml), representative of Th2 responses, were found.	Arena A, Bisignano C, Stassi G, Mandalari G, Wickham MS, Bisignano G. Immunomodulatory and antiviral activity of almond skins. *Immunol Lett* 132, 18-23 (2010).
351	XR(Almond skins)	IL4	-1	We have investigated the effect of almond skin extracts on the production of pro-inflammatory and	Arena A, Bisignano C, Stassi G, Filocamo A, Mandalari G. Almond Skin Inhibits HSV-2 Replication in Peripheral Blood

				anti-inflammatory cytokines in human peripheral blood mononuclear cells (PBMCs). PBMCs were either infected or not by herpes simplex virus type 2 (HSV-2), with and without prior treatment with almond skin extracts. Production of IL-17 induced by HSV-2 was inhibited by natural skins (NS) treatment. NS triggered PBMC in releasing IFN-α, IFN-γ and IL-4 in cellular supernatants. These results may explain the antiviral potential of almond skins./NS treatment was able to inhibit IL-17 production up-regulating IFN-α and IL-4. Therefore, NS could play a key role for the antiviral activity of almond skins since it is a good inducer of IFN-α.	Mononuclear Cells by Modulating the Cytokine Network. *Molecules* 20, 8816–8822 (2015).
352	XR	IL1A(IL-1 α)	-1	Exercise also significantly increased IL6, IL8, VEGF, INFγ, TNFα, IL1α, IL1β, MCP1, and EGG production rates by LPS-stimulated PBMCs, and this response was attenuated by DHA supplementation.	Capó X, Martorell M, Sureda A, Batle JM, Tur JA, Pons A. Docosahexaenoic diet supplementation, exercise and temperature affect cytokine production by lipopolysaccharide-stimulated mononuclear cells. J Physiol Biochem 72, 421–434(2016).
353	XR	STAT1	/		
354	XR	EGFR	/		
355	XR	CXCL10	/		
356	XR	SOD1	+1	After the almond intervention, serum alpha-tocopherol, SOD, and GPX increased significantly in smokers by 10, 35, and 16%, respectively and 8-OHdG, MDA, and DNA strand breaks decreased significantly by 28, 34, and 23%.	Li N, Jia X, Chen CY, et al. Almond consumption reduces oxidative DNA damage and lipid peroxidation in male smokers. J Nutr 137, 2717–2722(2007).

				In smokers, after almond supplementation, the concentration of 8-OHdG remained significantly greater than in nonsmokers by 98%. These results suggest almond intake can enhance antioxidant defenses and diminish biomarkers of oxidative stress in smokers.	
357	XR(Bitter almond essential oil)	PARP1	-1	Western blot results showed that the expressions of caspase-3 and PARP proteins were down-regulated after the treatment of 0.1, 0.25 and 0.5 mL/L bitter almond essential oil(BAEO) on HaCaT cells for 24 h	Yang Wenhua, Zhou Rui, Li Keyou.Effects of bitter almond essential oil on proliferation and apoptosis of HaCaT cells. Progress in Veterinary Medicine 84-89(2015).
358	XR	CASP8	/		
359	XR(Almond skin)	IFNG(IFN-γ)	-1	We have investigated the effect of almond skin extracts on the production of pro-inflammatory and anti-inflammatory cytokines in human peripheral blood mononuclear cells (PBMCs). PBMCs were either infected or not by herpes simplex virus type 2 (HSV-2), with and without prior treatment with almond skin extracts. Production of IL-17 induced by HSV-2 was inhibited by natural skins (NS) treatment. NS triggered PBMC in releasing IFN-α, IFN-γ and IL-4 in cellular supernatants. These results may explain the antiviral potential of almond skins./NS treatment was able to inhibit IL-17 production up-regulating IFN-α and IL-4. Therefore, NS could play a key role for the antiviral activity of almond skins since it is a good inducer of IFN-α.	Arena A, Bisignano C, Stassi G, Filocamo A, Mandalari G. Almond Skin Inhibits HSV-2 Replication in Peripheral Blood Mononuclear Cells by Modulating the Cytokine Network. Molecules 20, 8816–8822 (2015).

360	XR(Almond skin)	IFNG(IFN -γ)	+1	NS induced a significant decrease in HSV-2 replication, whereas extracts obtained from BS did not significantly influence the viral replication. High levels of cytokines production, such as IFN-alpha (38+/-5.3 pg/ml), IL-12 (215+/-17.1 pg/ml), IFN-gamma (5+/-0.7 IU/ml), TNF-alpha (3940+/-201.0 pg/ml), were detected. Moreover, IL-10 (210+/-12.2 pg/ml) and IL-4 (170+/-21.4 pg/ml), representative of Th2 responses, were found.	Arena A, Bisignano C, Stassi G, Mandalari G, Wickham MS, Bisignano G. Immunomodulatory and antiviral activity of almond skins. Immunol Lett 132, 18-23 (2010).
361	XR(Almond skin)	IFNG(IFN -γ)	-1	Exercise also significantly increased IL6, IL8, VEGF, INFγ, TNFα, IL1α, IL1β, MCP1, and EGG production rates by LPS-stimulated PBMCs, and this response was attenuated by DHA supplementation.	Capó X, Martorell M, Sureda A, Batle JM, Tur JA, Pons A. Docosahexaenoic diet supplementation, exercise and temperature affect cytokine production by lipopolysaccharide-stimulated mononuclear cells. J Physiol Biochem 72, 421–434(2016).
362	XR	IL2	/		
363	XR	CAT	+1	Fermentation supernatants (FS) of raw and roasted almonds had no genotoxic effects. FS obtained from raw or mildly roasted almonds (R1) significantly increased mRNA levels of CAT (4.6-fold), SOD2 (5.6-fold) and GSTP1 (3.9-fold) but not of GPx1. FS of almonds significantly reduced the growth of LT97 cells in a time- and dose-dependent manner. Treatment with 5% almonds FS increased the number of early apoptotic cells (17.4%, on average) and caspase-3 activity (4.9-fold, on average). The results indicate a chemopreventive potential of in vitro-fermented	Schlörmann W, Fischer S, Saupe C, Dinc T, Lorkowski S, Glei M. Influence of roasting on the chemopreventive potential of in vitro fermented almonds in LT97 colon adenoma cells. Int J Food Sci Nutr 69, 52-63(2018).

				almonds which is largely independent of the roasting process.	
364	XR	IL10	+1	NS induced a significant decrease in HSV-2 replication, whereas extracts obtained from BS did not significantly influence the viral replication. High levels of cytokines production, such as IFN-alpha (38+/-5.3 pg/ml), IL-12 (215+/-17.1 pg/ml), IFN-gamma (5+/-0.7 IU/ml), TNF-alpha (3940+/-201.0 pg/ml), were detected. Moreover, IL-10 (210+/-12.2 pg/ml) and IL-4 (170+/-21.4 pg/ml), representative of Th2 responses, were found.	Arena A, Bisignano C, Stassi G, Mandalari G, Wickham MS, Bisignano G. Immunomodulatory and antiviral activity of almond skins. Immunol Lett 132, 18-23 (2010).
365	XR	IL10	-1	Of serum inflammatory markers, IL-10 was decreased by almond intake (P ≤ 0.05), and ICAM-1, IL-1β, and IL-6 tended to be lower with almonds, compared to the cookies.	Jung H, Chen CO, Blumberg JB, Kwak HK. The effect of almonds on vitamin E status and cardiovascular risk factors in Korean adults: a randomized clinical trial. Eur J Nutr 57, 2069–2079(2018).
366	XR	HMOX1	/		
367	XR(Amygdalin)	BAX	-1	Amygdalin without zinc showed a strong anti-HepG2 activity. Furthermore, HepG2 cell lines treatment with amygdalin + 20µmol zinc and amygdalin + 800µmol zinc showed a highly significant apoptotic effect than the effect of amygdalin without zinc. Amygdalin treatment induced the cell cycle arrest at G2/M and increased the levels of P53, Bax, cytochrome c, and caspase-3 significantly, while it decreased the level of anti-apoptotic Bcl2.	El-Desouky MA, Fahmi AA, Abdelkader IY, Nasraldin KM. Anticancer effect of Amygdalin (vitamin B17) on hepatocellular carcinoma cell line(HepG2) in the presence and absence of Zinc . Anticancer Agents Med Chem 2020.
368	HX	PTGS2	±1	In the lps-induced model of RAW264.7 cell	Liu Yuhong.Study on the anti - gastric ulcer and ulcerative colitis by

				inflammation in vitro, 10, 20 and 40 mol/L of patchouli intervention reduced the level of PGE2 NO TNF-α and increased the level of IL-10, down-regulated the mRNA expression of COX-2 iNOS TNF-α and up-regulated the mRNA expression of IL-6 IL-1, thus slowing down the inflammatory response.	- patchouli alcohol metabolite in gastric juice. Traditional Chinese Medicine University Of Guangzhou (2018).
369	HX(Patchouli ketone)	PTGS2	-1	It inhibited the excessive production of inflammatory mediators (TNF-α, IL-1β, IL-6, IFN-γ, IL-12, PGE2, NO) in the serum of endotoxin shock mice, and down-regulated the expression of mediators related to inflammation (TNF-, IL-6, IL-12, COX-2, and iNOS) in the liver and lung tissues of endotoxin shock mice.	Li Yucui. Anti-inflammatory and antifungal activities and drug metabolism of Patchouli ketone and epimedium alcohol. Traditional Chinese Medicine University Of Guangzhou (2013).
370	HX(Epoxy patchouli)	PTGS2	-1	Epoxopatchouli significantly reduced the levels of proinflammatory cytokines such as TNF-α, IL-1β and IL-6 in the foot tissue, but also increased the levels of anti-inflammatory cytokines such as IL-4 and IL-10.Epoxide patchouli significantly down-regulated the expression of COX-2 and iNOS signaling pathways.	Liang Jiali. Effect and mechanism of Epoxy patchouli on anti-inflammation and anti-gastric ulcer.Traditional Chinese Medicine University Of Guangzhou (2018).
371	HX(Epoxy patchouli)	iNOS	-1	Epoxopatchouli significantly reduced the levels of proinflammatory cytokines such as TNF-α, IL-1β and IL-6 in the foot tissue, but also increased the levels of anti-inflammatory cytokines such as IL-4 and IL-10.Epoxide patchouli significantly down-regulated the expression of COX-2 and iNOS signaling pathways.	Liang Jiali. Effect and mechanism of Epoxy patchouli on anti-inflammation and anti-gastric ulcer.Traditional Chinese Medicine University Of Guangzhou (2018).
372	HX(Patchouli	iNOS	-1	In the lps-induced model of RAW264.7 cell	Liu Yuhong.Study on the anti - gastric ulcer and ulcerative colitis by

	alcohol)			inflammation in vitro, 10, 20 and 40 mol/L of patchouli intervention reduced the level of PGE2 NO TNF-α and increased the level of IL-10, down-regulated the mRNA expression of COX-2 iNOS TNF-α and up-regulated the mRNA expression of IL-6 IL-1, thus slowing down the inflammatory response.	- patchouli alcohol metabolite in gastric juice. Traditional Chinese Medicine University Of Guangzhou (2018).
373	HX(Patchouli ketone)	iNOS	-1	It inhibited the excessive production of inflammatory mediators (TNF-α, IL-1β, IL-6, IFN-γ, IL-12, PGE2, NO) in the serum of endotoxin shock mice, and down-regulated the expression of mediators related to inflammation (TNF-, IL-6, IL-12, COX-2, and iNOS) in the liver and lung tissues of endotoxin shock mice.	Li Yucui. Anti-inflammatory and antifungal activities and drug metabolism of Patchouli ketone and epimedium alcohol. Traditional Chinese Medicine University Of Guangzhou (2013).
374	HX(Patchouli alcohol)	RELA(P65)	-1	The contents of SOD GSH and CAT were increased and the content of MDA was decreased to inhibit oxidative stress.Reduced TNF-α IL-1β and IL-6 levels and down-regulated phosphorylation of p65 and IκB proteins to inhibit the NF-κB signaling pathway and reduce inflammation	Liu Yuhong.Study on the anti - gastric ulcer and ulcerative colitis by - patchouli alcohol metabolite in gastric juice. Traditional Chinese Medicine University Of Guangzhou (2018).
375	HX(Patchouli ketone)	TNF	-1	Patchouli ketone can significantly reduce the loss of hearing in the secretory otitis media model of guinea pigs, the thickness of mucous membrane and neutrophil infiltration, and the effect may be related to the inhibition of TNF-α and ICAM-1 expression in the ear mucous membrane.	Zeng Xiangyue, Sun Haiyan, Li Yangyang et al. Effect of Patchouli ketone on Expressions of TNF － α and ICAM － 1 in Ear Mucous Membrane of Guinea Pigs with Secretory Otitis Media.Chinese archives of traditional Chinese medicine 37, 1629-1633(2019).
376	HX(Patchouli	IL6	-1	In the lps-induced model of RAW264.7 cell	Liu Yuhong.Study on the anti - gastric ulcer and ulcerative colitis by

	alcohol)			inflammation in vitro, 10, 20 and 40 mol/L of patchouli intervention reduced the level of PGE2 NO TNF-α and increased the level of IL-10, down-regulated the mRNA expression of COX-2 iNOS TNF-α and up-regulated the mRNA expression of IL-6 IL-1, thus slowing down the inflammatory response.	- patchouli alcohol metabolite in gastric juice. Traditional Chinese Medicine University Of Guangzhou (2018).
377	HX(Patchouli ketone)	IL6	-1	Serum levels of TNF-α IL-6 IL-1β and MDA were decreased in dexamethasone group and patchoulone group	Zhang Weina, Zhang Mingming, Yu Min. Study on protective mechanism of Patchouli ketone on lung injury in COPD mice.Health Studies 39, 428-431(2019).
378	HX(Patchouli ketone)	IL6	-1	It inhibited the excessive production of inflammatory mediators (TNF-α, IL-1β, IL-6, IFN-γ, IL-12, PGE2, NO) in the serum of endotoxin shock mice, and down-regulated the expression of mediators related to inflammation (TNF-, IL-6, IL-12, COX-2, and iNOS) in the liver and lung tissues of endotoxin shock mice.	Li Yucui. Anti-inflammatory and antifungal activities and drug metabolism of Patchouli ketone and epimedium alcohol. Traditional Chinese Medicine University Of Guangzhou (2013).
379	HX(Epoxy patchouli)	IL6	-1	Epoxopatchouli significantly reduced the levels of proinflammatory cytokines such as TNF-α, IL-1β and IL-6 in the foot tissue, but also increased the levels of anti-inflammatory cytokines such as IL-4 and IL-10.Epoxide patchouli significantly down-regulated the expression of COX-2 and iNOS signaling pathways.	Liang Jiali. Effect and mechanism of Epoxy patchouli on anti - inflammation and anti - gastric ulcer.Traditional Chinese Medicine University Of Guangzhou (2018).
380	HX(Patchouli ketone)	MAPK14（P38α）	-1	Patchouli ketone can suppress the macrophages induced by LPS in the NF-κB JNK/SAPK and activation of p38MAPK signal transduction pathways, prompt Patchouli ketone of LPS in mice induced by endotoxin	Li Yucui. Anti-inflammatory and antifungal activities and drug metabolism of Patchouli ketone and epimedium alcohol. Traditional Chinese Medicine University Of Guangzhou (2013).

				shock protection mechanism, may be its blocking the NF-κB of LPS activated cells and MAPK signal transduction pathway, and regulate the expression of related genes, inflammatory mediators eventually curb excessive inflammatory mediators.	
381	HX	CASP3	+1	Epimedium significantly inhibited the proliferation of DU145 cells in a dose-dependent and time-dependent manner.It can enhance the expression of caspase-3 and Bax proteins in DU145 cells and reduce the expression of Livin bcl-2 proteins, so as to exert the effect of acarbazanol on inducing apoptosis and inhibiting proliferation of DUl45 cells	CAI Jian, Peng Cheng, Zhu Xiaoyan, et al.Inhibition of the growth of human androgen independent prostate cancer cells DU145 and its mechanism. Chinese journal of experimental formulae 165-169(2014).
382	HX(Patchouli alcohol)	PTGS1(COX1)	+1	After gavage of -patchouli at different doses, the protein expressions of COX-1, COX-2 and PGE2 were increased, and the expressions of VEGF and flt-1 were promoted.	Liu Yuhong.Study on the anti - gastric ulcer and ulcerative colitis by - patchouli alcohol metabolite in gastric juice. Traditional Chinese Medicine University Of Guangzhou (2018).
383	HX(Patchouli ketone)	ICAM1	-1	Patchouli ketone can significantly reduce the loss of hearing in the secretory otitis media model of guinea pigs, the thickness of mucous membrane and neutrophil infiltration, and the effect may be related to the inhibition of TNF-α and ICAM-1 expression in the ear mucous membrane.	Zeng Xiangyue, Sun Haiyan, Li Yangyang et al. Effect of Patchouli ketone on Expressions of TNF － α and ICAM － 1 in Ear Mucous Membrane of Guinea Pigs with Secretory Otitis Media.Chinese archives of traditional Chinese medicine 37, 1629-1633(2019).
384	HX(Patchouli alcohol)	IL1B(IL1β)	-1	The contents of SOD GSH and CAT were increased and the content of MDA was decreased to inhibit oxidative stress.Reduced TNF-α, IL-1β and IL-6 levels, and down-regulated phosphorylation of p65 and IκB	Liu Yuhong.Study on the anti - gastric ulcer and ulcerative colitis by - patchouli alcohol metabolite in gastric juice. Traditional Chinese Medicine University Of Guangzhou (2018).

				proteins to inhibit the NF-κB signaling pathway and reduce inflammation	
385	HX(Patchouli ketone)	IL1B(IL1 β)	-1	It inhibited the excessive production of inflammatory mediators (TNF-α, IL-1β, IL-6, IFN-γ, IL-12, PGE2, NO) in the serum of endotoxin shock mice, and down-regulated the expression of mediators related to inflammation (TNF-, IL-6, IL-12, COX-2, and iNOS) in the liver and lung tissues of endotoxin shock mice.	Li Yucui. Anti-inflammatory and antifungal activities and drug metabolism of Patchouli ketone and epimedium alcohol. Traditional Chinese Medicine University Of Guangzhou (2013).
386	HX(Patchouli ketone)	IL1B(IL1 β)	-1	Serum levels of TNF-α, IL-6, IL-1β and MDA were decreased in dexamethasone group and patchoulone group.	Zhang Weina, Zhang Mingming, Yu Min. Study on protective mechanism of Patchouli ketone on lung injury in COPD mice.Health Studies 39, 428-431(2019).
387	HX(Epoxy patchouli)	IL1B(IL1 β)	-1	Epoxopatchouli significantly reduced the levels of proinflammatory cytokines such as TNF-α, IL-1β and IL-6 in the foot tissue, but also increased the levels of anti-inflammatory cytokines such as IL-4 and IL-10. Epoxide patchouli significantly down-regulated the expression of COX-2 and iNOS signaling pathways.	Liang Jiali. Effect and mechanism of Epoxy patchouli on anti - inflammation and anti - gastric ulcer.Traditional Chinese Medicine University Of Guangzhou (2018).
388	HX	TP53（P53）	/		
389	HX	BCL2	/		
390	HX(Patchouli alcohol)	TGFB1(TGFβ1)	-1	PA has a mild hypotensive effect, and its mechanism may be related to inhibiting RAS and reducing TGF-TGF 1 and PAI-1 levels	Hu Guanying. Study on the mechanism of action of peregrine alcohol in the treatment of hypertensive renal damage. Chengdu University of TCM (2018).
391	HX(Patchouli oil)	MAPK1	-1	The results showed that the content of MDA, p38MAPK, Ras, Raf, MEK, ERK1 /2, Bax, Caspase9,	Song Qin, Song Jiquan. The effect of Patchouli oil on photoaging skin rats and the involvement of p38MAPK /EＲK signaling

				C -Fos and C -Jun were increased greatly (P<0.01).while the expression of Bcl2, SOD, GSHPX and CAT were decreased significantly(P<0.01)in model rats. The results also showed that these abnormal expressions were alleviated greatly after the medication of patchouli oil. At the mean time, the effect of Patchouli oil had a dose dependent manner(P<0.01).	pathway. Journal of Clinical and Experimental Medicine 15, 2191-2194 (2016).
392	HX(Wuwei Changyanning)	CXCL8 （IL8）	-1	Wuwei Changyanning can reduce the levels of inflammatory cytokines TNF-α and IL-8 in the serum of diseased rats and increase the levels of anti-inflammatory cytokines IL-10.It can reduce the content of MDA in the colon tissues of rats and increase the content of SOD and GSH-PX, indicating that it can restore normal immune function and reduce the damage and inflammation of colon mucosa in rats by down-regulating inflammatory cytokines and up-regulating anti-inflammatory cytokines and scavenging oxygen free radicals.	Yang Xian. Evaluation and quality control of the efficacy of Wuwei Changyanning in the treatment of ulcerative colitis.Chongqing University (2012).
393	HX	CRP	/		
394	HX	CCL2	/		
395	HX(Patchouli ketone)	MAPK8	-1	Patchouli ketone can suppress the macrophages induced by LPS in the NF-κB JNK/SAPK and activation of p38MAPK signal transduction pathways, prompt Patchouli ketone of LPS in mice induced by endotoxin shock protection mechanism, may be its blocking the NF-κB of LPS activated cells and MAPK signal	Li Yucui. Anti-inflammatory and antifungal activities and drug metabolism of Patchouli ketone and epimedium alcohol. Traditional Chinese Medicine University Of Guangzhou (2013).

				transduction pathway, and regulate the expression of related genes, inflammatory mediators eventually curb excessive inflammatory mediators.	
396	HX(Patchouli alcohol)	FOS(C-FOS)	+1	In the rat model of gastric ulcer induced by anhydrous ethanol, the contents of SOD GSH and CAT were increased and the content of MDA was decreased to inhibit oxidative stress after administration of different doses of -patchouli by gavage.Reduced TNF-α, IL-1β and IL-6 levels, as well as down-regulated phosphorylation of p65 and IκB proteins to inhibit NF-κB signaling and reduce inflammation.It can down-regulate the protein expression of Fas, FasL and caspase-3, up-regulate the gene expression of c-fos, c-jun and miR-21 and the phosphorylation level of ERK1/2 to inhibit the apoptosis of gastric mucosal cells.	Liu Yuhong.Study on the anti - gastric ulcer and ulcerative colitis by - patchouli alcohol metabolite in gastric juice. Traditional Chinese Medicine University Of Guangzhou (2018).
397	HX(Epoxy patchouli)	IL4	+1	Epoxopatchouli significantly reduced the levels of proinflammatory cytokines such as TNF-α, IL-1β and IL-6 in the foot tissue, but also increased the levels of anti-inflammatory cytokines such as IL-4 and IL-10.Epoxide patchouli significantly down-regulated the expression of COX-2 and iNOS signaling pathways.	Liang Jiali. Effect and mechanism of Epoxy patchouli on anti - inflammation and anti - gastric ulcer.Traditional Chinese Medicine University Of Guangzhou (2018).
398	HX(Patchouli oil)	IL4		Compared with the model control group, the fecal water content of the Patchouli oil group in the positive control group was all reduced, the defecation time was all prolonged, the serum SIgA, IL-4, IL-10 level of the	Huang Hongke, Luo Jianwei,Lli Xiaoting et al. Effect of Patchouli oil on intestinal mucosal barrier in rats with post-infectious irritable bowel synfrome. Chinese Journal of Nosocomiology 28, 971-974 (2018).

				relative contents of the colon tissues zo-1 and occludin was all increased, and the TNF-α level was decreased (P<0.05).(Positive control group (patchouli zhengqi liquid 3.3ml /kg) and Patchouli oil group (Patchouli oil 3.0g /kg)).	
399	HX	IL1AIL-1 α)	/		
400	HX	STAT1	/		
401	HX(Ageratum naphtha)	EGFR	+1	Rhizoma atractylodis and ageratum naphtha can be raised by G2M phase, S phase proportion in epithelial cell cycle, promote cell proliferation, improve cell vitality;Up-regulation of gene expression of EGF and EGFR, and regulation of ICE-6 cell activity/cell cycle cell proliferation and repair of cell damage by regulating P-ERK1 /ERK2 signaling proteins in the MAPK signaling channel.	Liu Fenghua. The repair mechanism of heat stress on intestinal epithelial cell damage and cooling granules in pigs. Nanjing Agricultural University (2009).
402	HX	EGFR		Immunohistochemical results showed that the expression of EGFR in gastric mucosa of rats in the model group was decreased (P<0.05), Huoxiang zhengqi liquid group increased significantly (P<0.05);Conclusion: Huoxiang zhengqi liquid can significantly improve the symptoms of spleen deficiency in rats with wet resistance syndrome and has a protective effect on gastric mucosa damage. The mechanism may be related to enhancing the ability of the body to resist oxidative stress and increasing the	Xue Xiaoqian, Huang Xuekuan, Gao Ning et al. Effects of Huoxiang Zhengqi Liquid on the Anti-oxidation and Expression of EGFR in Gastric Mucosa of Rats with Dampness Retention Syndrome. Chinese Journal of Experimental Traditional Medical Formulae 18,230-234(2012).

				expression of gastric mucosa EGFR.	
403	HX	CXCL10	/		
404	HX(Patchouli ketone)	SOD1	+1	Serum levels of TNF-α, IL-6, IL-1β and MDA were decreased in dexamethasone group and patchoulone group, SOD content all increased.	Zhang Weina, Zhang Mingming, Yu Min. Study on protective mechanism of Patchouli ketone on lung injury in COPD mice.Health Studies 39, 428-431(2019).
405	HX(Patchoulenone)	PARP1	+1	After treatment with 40, 60 and 80 g/mL of patchouli, the expression levels of activated caspase-9, activated caspase-3, and shear PRAP-1 proteins and Bax/ BCL-2 values in SGC-996 cells were significantly increased (all P < 0.05)	Wu Yaoshi, Huang Yuan, Dong Jiahong. Patchoulenone inhibits the proliferation of human gallbladder carcinoma SGC-996 cells.Tumor 37,50-57(2017).
406	HX	CASP8	+1		Kim, J. Y., Kim, E. H., Park, S. S. et al.. Quercetin sensitizes human hepatoma cells to TRAIL-induced apoptosis via Sp1-mediated DR5 up-regulation and proteasome-mediated c-FLIPS down-regulation. J Cell Biochem 105, 1386-1398(2008).
407	HX	IFNG(IFN-γ)	/		
408	HX	IL2	/		
409	HX(Patchouli oil)	CAT	+1	The results showed that the content of MDA, p38MAPK, Ras, Raf, MEK, ERK1 /2, Bax, Caspase9, C -Fos and C -Jun were increased greatly (P<0.01) while the expression of Bcl2, SOD, GSHPX and CAT were decreased significantly (P<0.01) in model rats . The results also showed that these abnormal expressions were alleviated greatly after the medication of patchouli oil . At the mean time, the effect of Patchouli oil had a dose dependent manner (P<0.01)	Song Qin, Song Jiquan. The effect of Patchouli oil on photoaging skin rats and the involvement of p38MAPK /EＲK signaling pathway. Journal of Clinical and Experimental Medicine 15, 2191-2194 (2016).

410	HX(Patchouli alcohol)	IL10	+1	In the lps-induced model of RAW264.7 cell inflammation in vitro, 10, 20 and 40 mol/L of patchouli intervention reduced the level of PGE2 NO TNF-α and increased the level of IL-10, down-regulated the mRNA expression of COX-2 iNOS TNF-α and up-regulated the mRNA expression of IL-6 IL-1, thus slowing down the inflammatory response.	Liu Yuhong.Study on the anti - gastric ulcer and ulcerative colitis by - patchouli alcohol metabolite in gastric juice. Traditional Chinese Medicine University Of Guangzhou (2018).
411	HX(Epoxy patchouli)	IL10	+1	Epoxopatchouli significantly reduced the levels of proinflammatory cytokines such as TNF-α, IL-1β and IL-6 in the foot tissue, but also increased the levels of anti-inflammatory cytokines such as IL-4 and IL-10.Epoxide patchouli significantly down-regulated the expression of COX-2 and iNOS signaling pathways.	Liang Jiali. Effect and mechanism of Epoxy patchouli on anti - inflammation and anti - gastric ulcer.Traditional Chinese Medicine University Of Guangzhou (2018).
412	HX(Patchouli oil)	IL10	+1	Compared with the model control group, the fecal water content of the Patchouli oil group in the positive control group was all reduced, the defecation time was all prolonged, the serum SIgA, IL-4, IL-10 level of the relative contents of the colon tissues zo-1 and occludin was all increased, and the TNF-α level was decreased ($P<0.05$).(Positive control group (patchouli zhengqi liquid 3.3ml /kg) and Patchouli oil group (Patchouli oil 3.0g /kg)).	Huang Hongke, Luo Jianwei,Lli Xiaoting et al. Effect of Patchouli oil on intestinal mucosal barrier in rats with post-infectious irritable bowel synfrome. Chinese Journal of Nosocomiology 28, 971-974+990 (2018).
413	HX	HMOX1(HO-1)	+1	β-patchouli ene anti infective inflammation effect of p-patchouli ene, after the treatment can significantly improve the acute lung injury induced by LPS in mice survival rate, reduce the damage rate of the lung tissue	Chen Xiaoying. Anti-inflammatory action and mechanism of patchouli-linn. Guangzhou University of TCM (2016).

				and edema, inhibit LPS induced the increase of MDA, MPO level in the lung tissue, lower alveolar lavage fluid of TNF-α, IL-1β and IL-6, raised Nrf2 and downstream antioxidant gene NQO1 GCLC mRNA and HO - 1 protein expression	
414	HX(Patchoulenone)	BAX	+1	Patchoulenone signifi cantly induced the apoptosis of SGC-996 cells after treatment with 40, 60 and 80 μg/mL patchoulenone and led to S-phase cell-cycle arrest (all $P < 0.05$). The expression levels of cleaved caspase-9, cleaved caspase-3, cleaved PARP-1 protein and the ratio of Bax to Bcl-2 in SGC-996 cells after treatment with 40, 60 and 80 μg/mL were up-regulated (all $P < 0.05$), whereas the expression levels of cyclin D1, cyclin A and cyclin B1 were down-regulated (all $P < 0.01$)	Wu Yaoshi, Huang Yuan, Dong Jiahong. Patchoulenone inhibits the proliferation of human gallbladder carcinoma SGC-996 cells.Tumor 37,50-57(2017).

414 literatures were found, using CNKI, PubMed, Web of Science and other literature database, to confirm the regulatory directions by 8 herbs. The searching key words included all of the synonyms of herbs and targets. Some other potential key words were used as well, such as one specific ingredient of the herb, a recipe containing one herb and some multiple treatments but including the herb, etc. which were noted in the brackets. Over 3 Literature largely corresponded to each herb with each target. The literatures involved were classed by IF (Impact Factor), study models (e.g., peripheral was better than neural, in vivo was superior in vitro, etc.), etc. We would further assess the direction based on these above rules. 8 herbs were named by the acronyms. The symbols were similar with Supplementary Table 5, which were used for indicating the regulation. Specifically, “+1” was up-regulated, “-1” was down-regulated, “/” was not listed, “-” indicated no effect. The key points were listed in details, and the literature references were attached as well.

Table S7. The preferable docking results among 919 ingredients of 8 herbs.

NO.	Compounds CID	Compounds Name	Docking Gscore (kcal/mol) 6LU7#	Docking Gscore (kcal/mol)6VSB#	Label in Fig 4G	Source Herbs
1	44258007	Madreselvin B	-9.017	-8.588	Red Dot (High in Both)	JYH(Flos Lonicerae, 金银花, Jin Yin Hua)
2	5280805	Rutin	-9.225	-6.377	Blue Dot (High in 6LU7)	LQ (Fructus Forsythiae, 连翘, Lian Qiao), MH(Herba Ephedrae, 麻黄, Ma Huang), GC (Radix Glycyrrhizae, 甘草, Gan Cao)
3	44259671	Madreselvin A	-7.626	-8.88	Green Dot (High in 6VSB)	JYH(Flos Lonicerae, 金银花, Jin Yin Hua)
4	42607811	Licorice glycoside E	-8.258	-8.74	Green Dot (High in 6VSB)	GC (Radix Glycyrrhizae, 甘草, Gan Cao)
5	6079994	Icos-5-enoic acid	-7.053	-8.563	Green Dot (High in 6VSB)	GC (Radix Glycyrrhizae, 甘草, Gan Cao)
6	486612	Matairesinoside	-7.284	-8.448	Green Dot (High in 6VSB)	LQ (Fructus Forsythiae, 连翘, Lian Qiao)
7	442428	Naringin	-6.891	-8.371	Green Dot (High in 6VSB)	GC (Radix Glycyrrhizae, 甘草, Gan Cao)
8	10009317	B-hydroxyacteoside	-8.383	-8.327	Green Dot (High in 6VSB)	LQ (Fructus Forsythiae, 连翘, Lian Qiao)

9	3081212	Fulvotomentoside A	-5.949	-8.24	Green Dot (High in 6VSB)	JYH (Flos Lonicerae, 金银花, Jin Yin Hua)
10	131484	Astrachrysoside A	-7.45	-8.226	Green Dot (High in 6VSB)	HQi (Radix Astragali seu Hedysari, 黄芪, Huang Qi)
11	127984	Astrasieversianin XV	-8.632	-8.21	Green Dot (High in 6VSB)	HQi (Radix Astragali seu Hedysari, 黄芪, Huang Qi)
12	14564503	Macranthoidin A	-7.597	-7.959	Green Dot (High in 6VSB)	JYH (Flos Lonicerae, 金银花, Jin Yin Hua)
13	132550846	Suspensaside B	-9.491	-7.682	Blue Dot (High in 6LU7)	LQ (Fructus Forsythiae, 连翘, Lian Qiao)
14	9986606	Plantainoside A	-9.116	-7.63	Blue Dot (High in 6LU7)	LQ (Fructus Forsythiae, 连翘, Lian Qiao)
15	10291003	Euchrenone	-9.133	-6.43	Blue Dot (High in 6LU7)	GC (Radix Glycyrrhizae, 甘草, Gan Cao)
16	5318767	Nicotiflorin	-8.966	-5.972	Blue Dot (High in 6LU7)	GC (Radix Glycyrrhizae, 甘草, Gan Cao)
17	932	Naringenin	-8.764	-5.553	Blue Dot (High in 6LU7)	MH (Herba Ephedrae, 麻黄, Ma Huang), GC (Radix Glycyrrhizae, 甘草, Gan Cao)
18	121304016	Remdesivir	-8.738	-6.754	Orange Dot (The Best Control among docking with 6LU7)	The Best Control within 6VSB
19	392622	Ritonavir	-8.089	-7.828	Orange Dot (The Best Control among docking with 6VSB)	The Best Control within 6LU7
20	92727	Lopinavir	-7.794	-7.037	Orange Dot (Control)	Positive Control
21	131411	Arbidol	-6.487	-3.934	Orange Dot (Control)	Positive Control

22	37542	Ribavirin	-6.159	-5.977	Orange Dot (Control)	Positive Control
23	64927	Chloroquine Phosphate	-5.936	-4.634	Orange Dot (Control)	Positive Control
24	492405	Favipiravir（T705）	-5.313	-5.489	Orange Dot (Control)	Positive Control

The preferable docking ingredients, with better efficiency either to only 6LU7, 6VSB or the both, were corresponded to herbs in turn based on the TCMSP database. The docking glide gscores, which indicates the docking energy or affinity with receptor (the lower, the better), were shown in details. The last seven (NO. 18 to NO.24) were the positive controls, which were reported as the potential drugs for combating SARS-CoV-2. Remdesivir (NO.18) and Ritonavir (NO.19) were the best control with 6VSB and 6LU7 respectively. Source herbs were presented in different names, including acronyms, Latin name, Chinese name and Chinese Pin Yin. Fig 4 H pictured this data. The full docking results of 8 herbs (919 unique ingredients) were listed in the Supplementary Table 8.

Table S8. The entire results of 919 ingredients from 8 herbs docking with the two core structures (6LU7 & 6VSB) of SAR-CoV-2.

NO.	CID	Molecule Name	Docking gscore (kcal/mol) 6VSB#	Docking gscore (kcal/mol) 6LU7#	Label
1	44259671	madreselvin A	-8.88	-7.626	High in 6VSB
2	42607811	licorice glycoside E	-8.74	-8.258	High in 6VSB
3	44258007	madreselvin B	-8.588	-9.017	High in Both
4	132550846	suspensaside B	-7.682	-9.491	High in 6LU7
5	392622	Ritonavir	-7.828	-8.089	The Best Control within 6VSB
6	51666248	neoliquiritin	-7.692	-6.649	Relatively Low
7	6079994	icos-5-enoic acid	-8.563	-7.053	High in 6VSB
8	486612	Matairesinoside	-8.448	-7.284	High in 6VSB

9	442428	naringin	-8.371	-6.891	High in 6VSB
10	10009317	B-hydroxyacteoside	-8.327	-8.383	High in 6VSB
11	3081212	fulvotomentoside A	-8.24	-5.949	High in 6VSB
12	131484	astrachrysoside A	-8.226	-7.45	High in 6VSB
13	127984	astrasieversianin XV	-8.21	-8.632	High in 6VSB
14	14564503	macranthoidin A	-7.959	-7.597	High in 6VSB
15	107876	Procyanidin	-7.677	-8.29	Relatively Low
16	118705380	Akebiasaponin D	-7.671	-6.655	Relatively Low
17	9986606	plantainoside A	-7.63	-9.116	High in 6LU7
18	101938907	Licorice glycoside A	-7.551	-7.601	Relatively Low
19	73356106	EEE	-7.556	-6.983	Relatively Low
20	5281800	acteoside	-7.513	-6.964	Relatively Low
21	6442994	Forsythoside F	-7.512	-8.263	Relatively Low
22	5281773	Forsythiaside	-7.491	-7.575	Relatively Low
23	5282150	Rhoifolin	-7.48	-7.198	Relatively Low
24	101231533	Forsythoside G	-7.456	-8.116	Relatively Low
25	6474310	isochlorogenic,acid	-7.425	-6.246	Relatively Low
26	503737	liquiritin	-7.41	-6.627	Relatively Low
27	5281798	suspensaside A	-7.391	-8.443	Relatively Low
28	5280441	vitexin	-7.367	-6.805	Relatively Low
29	101606424	gancaonin T	-7.301	-7.1	Relatively Low
30	122097	Soyasaponin I	-7.231	-5.189	Relatively Low
31	5353588	5,8,2'-Trihydroxy-7-methoxy flavone	-7.187	-8.643	Relatively Low
32	10621	hesperidin	-7.174	-7.762	Relatively Low
33	102183195	Centauroside	-7.072	-6.175	Relatively Low

34	73296	Helixin	-7.072	-7.079	Relatively Low
35	3037925	methyl-9-methyl tetradecanoate	-7.051	-6.692	Relatively Low
36	5315651	campneoside	-7.048	-8.046	Relatively Low
37	23928102	Forsythoside B	-6.97	-8.383	Relatively Low
38	131753130	6U-O-acetylliquiritin	-6.96	-7.654	Relatively Low
39	5282152	Scolymoside	-6.916	-6.341	Relatively Low
40	5281255	Corylifolinin	-7.02	-7.809	Relatively Low
41	163744	uralsaponin B	-6.857	-5.422	Relatively Low
42	5320092	neoisoliquiritin	-6.893	-6.056	Relatively Low
43	442664	Vicenin-2	-6.857	-7.332	Relatively Low
44	5318591	Isoliquiritin	-7.06	-6.232	Relatively Low
45	91895373	Isomartynoside	-6.744	-7.753	Relatively Low
46	5280637	luteolin	-6.727	-6.315	Relatively Low
47	71629	Resivit	-6.69	-7.404	Relatively Low
48	480816	Gancaonin S	-6.69	-6.742	Relatively Low
49	134694234	AstragalosideIV	-6.681	-7.401	Relatively Low
50	5282451	NK	-6.679	-7.878	Relatively Low
51	5317756	Glycycoumarin	-6.658	-6.934	Relatively Low
52	12314162	PHILLYRIN	-6.65	-7.009	Relatively Low
53	5280704	apigenin	-6.609	-6.341	Relatively Low
54	442665	violanthin	-6.61	-7.278	Relatively Low
55	442658	schaftoside	-6.595	-7.67	Relatively Low
56	480783	8-Prenylwighteone	-6.553	-5.975	Relatively Low
57	101939210	glycyroside	-6.501	-7.127	Relatively Low
58	11168362	(+)-epipinoresinol-4'-O-D-gl	-6.474	-6.882	Relatively Low

		ucoside			
59	195342	Araboglycyrrhizin	-6.454	-6.071	Relatively Low
60	195343	apioglycyrrhizin	-6.444	-5.267	Relatively Low
61	3084961	Oroxindin	-6.441	-6.827	Relatively Low
62	10291003	euchrenone	-6.43	-9.133	High in 6LU7
63	142443859	1,5,8-trimethyl-1,2-dihydro-naphthalene	-6.421	-5.978	Relatively Low
64	6438452	[(3R)-3,7-dimethylocta-1,6-dien-3-yl] acetate	-7.466	-7.942	Relatively Low
65	68245	delphinidin	-6.526	-6.823	Relatively Low
66	5281792	rosmarinic acid	-6.416	-5.873	Relatively Low
67	9064	(+)-catechin	-6.392	-6.945	Relatively Low
68	5316900	3,5-Dihydroxy-4',7-dimethoxyflavone	-6.408	-6.285	Relatively Low
69	101665834	acetylastragaloside I	-6.354	-3.641	Relatively Low
70	6450959	Gancaonin C	-6.359	-7.448	Relatively Low
71	5280805	rutin	-6.377	-9.225	High in 6LU7
72	480818	Gancaonin U	-6.322	-7.541	Relatively Low
73	9015	MEHQ	-6.304	-6.035	Relatively Low
74	5273570	(-)-Olivir	-6.287	-6.269	Relatively Low
75	5280633	Neochlorogenic acid	-6.264	-6.584	Relatively Low
76	5319160	4,5-di-O-caffeoylquinic acid methyl ester	-6.252	-6.947	Relatively Low
77	480817	Gancaonin V	-6.217	-6.997	Relatively Low
78	44593361	orobanchoside	-6.211	-5.974	Relatively Low
79	480802	Gancaonin Q	-6.223	-6.507	Relatively Low

80	62074	Glycyram	-6.212	-5.107	Relatively Low
81	14982	glycyrrhizin	-6.212	-5.107	Relatively Low
82	441457	Cathine	-6.217	-5.79	Relatively Low
83	69634125	Forsythoside E	-6.205	-6.91	Relatively Low
84	5281789	Licoisoflavone	-6.222	-6.55	Relatively Low
85	5282151	vitexin	-6.244	-6.525	Relatively Low
86	10336244	shinpterocarpin	-6.187	-5.597	Relatively Low
87	5282166	quercetin 7-O-β-D-glucoside	-6.205	-8.025	Relatively Low
88	5282102	kaempferol	-6.181	-7.954	Relatively Low
89	503731	Licocoumarone	-6.153	-7.089	Relatively Low
90	442813	Ononin	-6.152	-5.665	Relatively Low
91	5317480	Lupiwighteone	-6.156	-7.138	Relatively Low
92	3286789	leucopelargonidin	-6.144	-8.652	Relatively Low
93	5317300	Eurycarpin A	-6.15	-7.145	Relatively Low
94	5315127	Uralenol-3-methylether	-6.174	-6.125	Relatively Low
95	10052718	3,4-Dicaffeoylquinic acid	-6.14	-6.666	Relatively Low
96	5318585	Isolicoflavonol	-6.155	-7.088	Relatively Low
97	5317765	Glycyrrhiza flavonol A	-6.152	-6.993	Relatively Low
98	100528	arctiin	-6.109	-5.948	Relatively Low
99	892	inositol	-6.106	-5.934	Relatively Low
100	5481663	Narcissoside	-6.129	-7.743	Relatively Low
101	73205	Sigmoidin-B	-6.112	-6.615	Relatively Low
102	480859	Glyasperin C	-6.072	-6.541	Relatively Low
103	194727	EIC	-6.072	-4.383	Relatively Low
104	13889020	5-Hydroxy-7ﾉ *3 &,4'-trimethoxyflavanone*	-6.043	-6.751	Relatively Low

105	12889143	licorice-saponin H2	-6.044	-5.086	Relatively Low
106	5318267	Calycosin	-6.027	-5.929	Relatively Low
107	14135325	Dihydrobaicalin	-6.014	-6.352	Relatively Low
108	64982	Baicalin	-5.997	-6.273	Relatively Low
109	101422758	Isoviolanthin	-6.026	-7.869	Relatively Low
110	92123	Picein	-5.984	-6.302	Relatively Low
111	439533	taxifolin	-6.007	-7.306	Relatively Low
112	332427	Lariciresinol	-5.976	-7.631	Relatively Low
113	6101	Daturic acid	-5.975	-5.388	Relatively Low
114	442154	Afzelechin	-5.975	-8.267	Relatively Low
115	5280544	Herbacetin	-5.982	-7.497	Relatively Low
116	96506	Nortangeretin	-6.001	-8.19	Relatively Low
117	5318767	nicotiflorin	-5.972	-8.966	High in 6LU7
118	5281612	Diosmetin	-5.976	-5.96	Relatively Low
119	17047	RNG	-5.938	-6.527	Relatively Low
120	5281631	Euxanthone	-5.944	-6.161	Relatively Low
121	5317483	Gancaonin A	-5.948	-7.614	Relatively Low
122	10259181	methyl icos-11-enoate	-5.926	-6.255	Relatively Low
123	5317570	Germacrene D	-5.921	-5.52	Relatively Low
124	10253785	Luteolin 7-O-glucuronide	-5.96	-8.107	Relatively Low
125	5320083	Glycyrol	-5.932	-6.171	Relatively Low
126	480865	Licoricidin	-5.912	-5.987	Relatively Low
127	442411	Glepidotin A	-5.911	-7.082	Relatively Low
128	8468	vanillic acid	-5.906	-5.377	Relatively Low
129	182232	ent-Epicatechin	-5.881	-6.98	Relatively Low
130	5492110	Ochnaflavone	-5.961	-7.009	Relatively Low

131	102183193	7-epi-Vogeloside	-5.864	-5.994	Relatively Low
132	5273755	eupatilin	-5.896	-7.297	Relatively Low
133	101939796	vogeloside	-5.859	-6.656	Relatively Low
134	129901222	licorice-saponin B2	-5.862	-5.122	Relatively Low
135	5481964	Licoflavonol	-5.859	-6.222	Relatively Low
136	443639	epi-Afzelechin	-5.845	-8.599	Relatively Low
137	5352005	Retusin	-5.831	-6.209	Relatively Low
138	5280666	Chryseriol	-5.861	-6.734	Relatively Low
139	480815	Gancaonin R	-5.813	-6.403	Relatively Low
140	13254473	eriodictyol-7-o-glucoside	-5.808	-6.36	Relatively Low
141	5320946	Rhamnocitrin	-5.812	-7.651	Relatively Low
142	3084995	Isoschaftoside	-5.825	-8.026	Relatively Low
143	14891565	licorice-saponin C2_qt	-5.766	-4.325	Relatively Low
144	9840805	licochalcone C	-5.816	-7.206	Relatively Low
145	5202	DS	-5.726	-6.519	Relatively Low
146	9294	Eciphin	-5.729	-5.689	Relatively Low
147	130583	DFV	-5.727	-6.781	Relatively Low
148	10246505	Licoflavone	-5.734	-7.943	Relatively Low
149	124049	Glabranin	-5.724	-7.699	Relatively Low
150	392442	glyasperin F	-5.768	-6.996	Relatively Low
151	10548420	8-epiloganin	-5.711	-6.876	Relatively Low
152	69867	ICO	-5.711	-5.247	Relatively Low
153	7209	DEP	-5.698	-6.669	Relatively Low
154	72	protocatechuic acid	-5.691	-5.599	Relatively Low
155	5318645	isorhamnetin	-5.72	-6.988	Relatively Low
156	6508	quinic acid	-5.691	-6.089	Relatively Low

157	452864	licorice-saponin C2	-5.685	-5.409	Relatively Low
158	10296	ursolic acid	-5.666	-5.653	Relatively Low
159	2725045	(2S)-2-myristyloxirane	-5.689	-6.825	Relatively Low
160	3010930	8-hydroxypinoresinol	-5.646	-6.919	Relatively Low
161	46218174	5,2',6'-Trihydroxy-7,8-dimethoxyflavone	-5.679	-7.762	Relatively Low
162	10076238	Liquiritin apioside	-5.644	-5.954	Relatively Low
163	676295	7,4'-Dihydroxyflavone	-6.063	-6.745	Relatively Low
164	131627	eth	-5.625	-5.536	Relatively Low
165	6253	Arabinose,d	-5.616	-6.12	Relatively Low
166	480784	glyasperin B	-5.619	-7.154	Relatively Low
167	5280460	Scopoletol	-5.623	-5.917	Relatively Low
168	14187587	isoglycycoumarin	-5.614	-6.283	Relatively Low
169	5320287	Ombuin	-5.614	-6.048	Relatively Low
170	231114	Norlapachol	-5.613	-6.201	Relatively Low
171	5281708	daidzein	-5.607	-7.173	Relatively Low
172	440735	eriodictyol	-5.614	-6.399	Relatively Low
173	135398749	CHEBI:7	-5.87	-7.102	Relatively Low
174	5317481	Gancaonin D	-5.588	-7.168	Relatively Low
175	131420	Astraisoflavanin	-5.58	-6.837	Relatively Low
176	5280681	3-O-Methylquercetin	-5.611	-6.597	Relatively Low
177	11045420	rengyoside A	-5.571	-6.817	Relatively Low
178	64981	arctigenin	-5.571	-7.12	Relatively Low
179	221493	CHD	-5.559	-6.086	Relatively Low
180	5318869	Jaranol	-5.55	-7.778	Relatively Low
181	3001497	C10230	-6.862	-5.205	Relatively Low

182	44453332	forsythialan B	-5.543	-6.146	Relatively Low
183	57085343	4-[(3R)-3-hydroxybutyl] phenol	-5.657	-7.324	Relatively Low
184	932	naringenin	-5.553	-8.764	High in 6LU7
185	480787	Glycyrin	-5.535	-5.463	Relatively Low
186	68262	DMBQ	-5.526	-5.461	Relatively Low
187	5315126	Uralenol	-5.555	-6.546	Relatively Low
188	131751372	Kanzonol Z	-5.534	-6.923	Relatively Low
189	4484952	Phaseolinisoflavan	-5.514	-6.812	Relatively Low
190	2214	Apocynin	-5.546	-6.088	Relatively Low
191	323	coumarin	-5.505	-4.967	Relatively Low
192	91520	catalpol	-5.495	-5.378	Relatively Low
193	5280804	quercetin	-5.521	-7.255	Relatively Low
194	5281787	Caffeate	-5.49	-6.387	Relatively Low
195	5318999	Licochalcone B	-5.558	-6.516	Relatively Low
196	1794427	Heriguard	-5.479	-5.887	Relatively Low
197	5321205	Scutevulin	-5.51	-7.264	Relatively Low
198	65348	7-epi-Loganin_qt	-5.497	-7.127	Relatively Low
199	4374	()-N-Methylephedrine	-5.562	-6.688	Relatively Low
200	21238	Caeruloside C	-5.47	-5.776	Relatively Low
201	88295	19889-94-2	-5.468	-5.493	Relatively Low
202	132127	XLS	-6.498	-7.591	Relatively Low
203	87691	Loganin	-5.462	-5.566	Relatively Low
204	5315396	Yinyanghuo D	-5.5	-6.16	Relatively Low
205	7028	Psi-ephedrin	-5.467	-5.635	Relatively Low
206	5281672	myricetin	-5.482	-6.125	Relatively Low

207	11809239	Cornoside	-5.444	-6.656	Relatively Low
208	13405	p-Toluyl chloride	-5.443	-5.521	Relatively Low
209	7947	Fleet-X	-5.441	-5.215	Relatively Low
210	220841	2,6-Dimethylnaphthalene	-5.438	-5.921	Relatively Low
211	6476139	methyl chlorogenate	-5.431	-6.978	Relatively Low
212	443024	Acanthoside B	-5.422	-6.543	Relatively Low
213	439612	Secologanate	-5.431	-6.239	Relatively Low
214	480799	glyinflanin A	-6.137	-6.768	Relatively Low
215	102177109	4,5-Dicaffeoylquinic acid	-5.413	-5.873	Relatively Low
216	11596309	glabrol	-5.409	-7.556	Relatively Low
217	124050	Isoglycyrol	-5.402	-5.797	Relatively Low
218	445929	D-Galacturonic acid, homopolymer	-5.387	-5.574	Relatively Low
219	244	WLN: Q1R	-5.385	-5.85	Relatively Low
220	162868	Secoxyloganin	-5.373	-5.618	Relatively Low
221	5271991	Ganhuangenin	-5.404	-6.101	Relatively Low
222	53462251	uralenneoside	-5.385	-7.463	Relatively Low
223	44257530	Phaseol	-5.373	-6.541	Relatively Low
224	133867	Liconeolignan	-5.392	-6.523	Relatively Low
225	13892717	Adoxosidic acid	-5.366	-5.291	Relatively Low
226	5318998	licochalcone a	-5.412	-7.928	Relatively Low
227	5317777	Glyzaglabrin	-5.346	-6.836	Relatively Low
228	5281702	tricin	-5.365	-5.896	Relatively Low
229	5988	glyasperins D	-5.324	-6.301	Relatively Low
230	5280378	formononetin	-5.327	-6.519	Relatively Low
231	14136854	secologanoside	-5.335	-5.065	Relatively Low

		7-methylester_qt			
232	54717187	1,1,6-trimethyl-2H-	-5.317	-7.599	Relatively Low
233	12166	naphthalene	-5.314	-4.973	Relatively Low
234	8447	p-Nitrosotoluene	-5.311	-6.624	Relatively Low
235	5281677	Usaf cs-6	-5.306	-6.141	Relatively Low
236	10774324	pachypodol	-5.293	-4.135	Relatively Low
237	139041	suspenolic acid	-5.29	-5.788	Relatively Low
238	442811	phenanthrone	-5.28	-6.426	Relatively Low
239	5316952	Mucronulatol	-5.281	-4.135	Relatively Low
240	29408	Docosyl caffeate	-5.275	-4.856	Relatively Low
241	5742590	patchoulane	-5.274	-4.571	Relatively Low
242	44593449	Sitogluside	-5.269	-7.703	Relatively Low
243	5281607	5,7,4'-trihydroxy-6-	-5.309	-7.143	Relatively Low
244	15840593	methoxyflavanone	-5.259	-5.691	Relatively Low
245	64782	chrysin	-5.336	-6.275	Relatively Low
246	122851	Licoagrocarpin	-5.247	-6.509	Relatively Low
247	185617	Tybraine	-5.245	-7.187	Relatively Low
248	4978	licopyranocoumarin	-5.426	-7.974	Relatively Low
249	65575	scutellarein	-5.216	-5.171	Relatively Low
250	480873	Psuedohypericin	-5.214	-6.776	Relatively Low
251	5316802	Cedrol	-5.351	-7.136	Relatively Low
252	22135564	1-Methoxyphaseollidin	-5.207	-6.196	Relatively Low
253	938	Kanzonol E	-5.214	-5.264	Relatively Low
254	5317479	OXA	-5.214	-6.125	Relatively Low
255	15559328	nicotinic acid	-5.214	-5.718	Relatively Low
256	690730	Gancaonin B	-5.202	-6.019	Relatively Low

257	37517	forsythide	-5.2	-6.732	Relatively Low
258	11468	1Ph5SHTetrazol	-5.2	-5.692	Relatively Low
259	147394	LFA	-5.199	-5.587	Relatively Low
260	5320291	1-terpineol	-5.497	-6.53	Relatively Low
261	161276	Eriodictiol-7-glucoside	-5.187	-5.975	Relatively Low
262	10725564	Onjixanthone I	-5.181	-5.758	Relatively Low
263	637796	secologanin dimethylacetal_qt	-5.179	-5.855	Relatively Low
264	165536	rengyolone	-5.175	-4.742	Relatively Low
265	11363	Isosafrole	-5.174	-5.752	Relatively Low
266	91457	Aristolone	-5.172	-6.024	Relatively Low
267	188308	Pyruvophenone	-5.193	-8.331	Relatively Low
268	5281671	beta-Eudesmol	-5.164	-6.987	Relatively Low
269	5318679	Carthamidin	-5.185	-6.444	Relatively Low
270	31493	Morusin	-5.159	-5.408	Relatively Low
271	480774	Isotrifoliol	-5.157	-7.197	Relatively Low
272	3845	m-Ethylacetophenone	-5.15	-5.821	Relatively Low
273	182259	Glabrene	-5.149	-7.156	Relatively Low
274	5281605	KYNA	-5.199	-7.228	Relatively Low
275	108770	Vestitol	-5.145	-6.528	Relatively Low
276	6428458	baicalein	-5.143	-5.486	Relatively Low
277	23724664	ST069309	-5.142	-5.474	Relatively Low
278	68071	Methyl (Z)-cinnamate	-5.157	-7.991	Relatively Low
279	5316733	(-)-Medicocarpin	-5.152	-6.926	Relatively Low
280	5317768	Pinocembrin	-5.133	-6.41	Relatively Low
281	5144	dihydrooroxylin A	-5.128	-5.526	Relatively Low

282	5281691	Glypallichalcone	-5.12	-7.325	Relatively Low
283	10787	Safrol	-5.106	-6.457	Relatively Low
284	10223	Rhamnetin	-5.106	-6.097	Relatively Low
285	15380912	WLN: QR BQ DQ	-5.108	-6.376	Relatively Low
286	10721	delta-amorphene	-5.103	-4.869	Relatively Low
287	73399	Kanzonol F	-5.096	-5.28	Relatively Low
288	446578	WLN: NCR B1	-5.095	-6.048	Relatively Low
289	392443	Pinoresinol	-5.149	-6.655	Relatively Low
290	73352581	Fucopyranose, L-	-5.085	-6.451	Relatively Low
291	93009	licoisoflavanone	-5.082	-4.57	Relatively Low
292	188316	Xambioona	-5.076	-7.213	Relatively Low
293	5481948	L-Bornyl acetate	-5.094	-7.203	Relatively Low
294	171335	Moslosooflavone	-5.258	-7.007	Relatively Low
295	135465089	Semilicoisoflavone B	-5.15	-5.042	Relatively Low
296	7059596	Protopseudohypericin	-5.152	-5.507	Relatively Low
297	11455	DFA	-5.059	-5.181	Relatively Low
298	5281804	()-N-Methylpseudoephedrine	-5.058	-6.875	Relatively Low
299	101297655	m-Methylacetophenone	-5.057	-5.119	Relatively Low
300	440752	Prunetin	-5.052	-5.943	Relatively Low
301	326	Matatabiether	-5.037	-4.751	Relatively Low
302	520957	6-Hydroxykynurenate	-5.036	-5.353	Relatively Low
303	785	cuminal	-5.027	-6.193	Relatively Low
304	6442675	longipinene	-5.095	-7.728	Relatively Low
305	5481234	hydroquinone	-5.048	-6.394	Relatively Low
306	161271	echinatin	-5.021	-5.727	Relatively Low
307	11229486	Licoisoflavone B	-5.019	-4.809	Relatively Low

308	5319013	Salvigenin	-5.024	-6.017	Relatively Low
309	7929	CLOVENE	-5.015	-4.973	Relatively Low
310	5320118	Licoricone	-5.02	-7.037	Relatively Low
311	89640	m-xylene	-5.025	-5.703	Relatively Low
312	5318619	Neouralenol	-5.006	-5.767	Relatively Low
313	10363	loganic	-5.001	-5.964	Relatively Low
314	5481224	Isoononin	-5.026	-8.44	Relatively Low
315	49856081	carvenone	-5.252	-8.066	Relatively Low
316	10237	Guajavarin	-5.153	-6.628	Relatively Low
317	198186	licochalcone G	-5.139	-5.423	Relatively Low
318	15689652	bicuculline	-4.961	-6.8	Relatively Low
319	122635	2,5-Dimethylpyrazole	-4.97	-6.968	Relatively Low
320	6602508	7-O-methylisomucronulatol	-4.951	-4.642	Relatively Low
321	5281703	Emodinanthrone	-4.985	-7.216	Relatively Low
322	75818	stigmasterol-β-glucoside	-4.969	-5.424	Relatively Low
323	638278	wogonin	-5.223	-8.363	Relatively Low
324	14655552	Methylbenzylamine	-4.939	-6.076	Relatively Low
325	7013	isoliquiritigenin	-4.933	-7.334	Relatively Low
326	69453	oroxylin a	-4.922	-5.652	Relatively Low
327	636883	PANA	-4.921	-6.377	Relatively Low
328	65373	BZQ	-4.918	-6.14	Relatively Low
329	2797	Licoagroisoflavone	-4.913	-4.809	Relatively Low
330	5281704	Secoisolariciresinol	-4.918	-5.868	Relatively Low
331	5281522	isobutyric acid	-4.905	-4.445	Relatively Low
332	5322111	Castanin	-4.905	-5.099	Relatively Low
333	21722915	Isocaryophyllene	-4.894	-5.264	Relatively Low

334	14632193	(Z)-caryophyllene	-4.967	-6.984	Relatively Low
335	139291297	(+)-pinoresinol	-4.889	-6.093	Relatively Low
336	88301	monomethyl ether	-4.887	-4.926	Relatively Low
		4,2',4',			
337	164660	alpha-Tetrahydroxydihydroc halcone	-5.054	-6.261	Relatively Low
338	5320438	4-stearylmorpholine	-4.904	-6.18	Relatively Low
339	8433	19894-97-4	-5.146	-6.074	Relatively Low
340	5280442	Protohypericin	-4.901	-6.58	Relatively Low
341	91354	Pectolinarigenin	-4.861	-4.969	Relatively Low
342	363707	Karenzu DK2	-4.861	-5.234	Relatively Low
343	13965473	acacetin	-4.866	-6.042	Relatively Low
344	6989	alloaromadedrene	-4.856	-5.942	Relatively Low
345	50515	rengyol	-4.88	-6.726	Relatively Low
346	7469	Odoratin	-4.884	-5.94	Relatively Low
347	124052	thymol	-4.848	-6.603	Relatively Low
348	160876	FA	-4.844	-5.692	Relatively Low
349	192490	Piceol	-4.843	-6.295	Relatively Low
350	12303902	Glabridin	-4.838	-5.494	Relatively Low
351	5317652	epiberberine	-4.845	-6.522	Relatively Low
352	58160	Uralene	-4.835	-6.656	Relatively Low
353	736186	copaene	-4.826	-4.909	Relatively Low
354	1140	Glabrone	-4.825	-4.819	Relatively Low
355	23991884	Cyclobutanol, 1-ethyl-	-4.824	-6.246	Relatively Low
356	373261	isoferulic acid	-4.839	-6.411	Relatively Low
357	6428995	toluene	-4.816	-4.874	Relatively Low

358	439710	7-Methoxy-2-methyl isoflavone	-4.813	-5.954	Relatively Low
359	14896	Eriodyctiol (flavanone)	-4.81	-5.511	Relatively Low
360	192240	cis-Cinnamaldehyde	-4.811	-5.394	Relatively Low
361	10150	RAM	-4.896	-6.366	Relatively Low
362	480780	(-)-nopinene	-4.802	-5.912	Relatively Low
363	5281617	darendoside B	-4.797	-6.3	Relatively Low
364	5281520	2,6,2',4'-tetrahydroxy-6'-met hoxychaleone	-4.792	-5.159	Relatively Low
365	101731	Gancaonin G	-4.787	-4.64	Relatively Low
366	493570	Genkwanin	-4.782	-6.645	Relatively Low
367	926139	alpha-humulene	-4.763	-6.057	Relatively Low
368	13297	β-patchoulene	-4.761	-6.502	Relatively Low
369	12594	Flavaxin	-4.76	-5.064	Relatively Low
370	6024	FMT	-4.759	-5.884	Relatively Low
371	86609	PEL	-4.758	-5.688	Relatively Low
372	10364	3β-formylglabrolide	-4.752	-5.695	Relatively Low
373	90479675	OCT	-4.752	-4.069	Relatively Low
374	15228662	alpha-Cubebene	-4.743	-6.238	Relatively Low
375	92762	o-Thymol	-4.738	-5.031	Relatively Low
376	1274465	glabrolide	-5.065	-5.756	Relatively Low
377	480854	3'-Hydroxy-4'-O-Methylglab ridin	-4.749	-6.163	Relatively Low
378	3749	alpha-Eudesmol	-4.72	-6.111	Relatively Low
379	13889022	Scopine	-4.71	-5.507	Relatively Low
380	7410	3-Hydroxyglabrol	-4.706	-5.134	Relatively Low

381	259846	NON	-4.699	-3.655	Relatively Low
382	336327	rivularin	-4.686	-6.064	Relatively Low
383	5370646	Hypnon	-4.677	-5.795	Relatively Low
384	6949	lupeol	-4.675	-4.983	Relatively Low
385	5320399	Medicarpin	-4.672	-7.749	Relatively Low
386	3663	Methylbenzylideneacetone	-4.854	-8.021	Relatively Low
387	159278	guaiene	-4.666	-6.819	Relatively Low
388	5959	Panicolin	-4.665	-6.175	Relatively Low
389	6445180	hypericin	-4.684	-6.481	Relatively Low
390	1110	salidroside	-4.66	-3.869	Relatively Low
391	64685	CAM	-4.644	-5.926	Relatively Low
392	1548883	2-methyl-6-ethyl decane	-4.643	-4.705	Relatively Low
393	5281781	succinic acid	-4.664	-5.75	Relatively Low
394	480872	Heptan	-4.634	-6.912	Relatively Low
395	528708	FERULIC ACID (CIS)	-4.628	-6.296	Relatively Low
396	189685	irisolidone	-4.658	-5.472	Relatively Low
397	136419	1-Methoxyficifolinol	-4.621	-6.42	Relatively Low
398	7150	calacorene	-4.62	-5.529	Relatively Low
399	24055	EGENINE	-4.619	-5.16	Relatively Low
400	135502249	Daidzein dimethyl ether	-5.113	-6.775	Relatively Low
401	11902	Clorius	-4.612	-5.354	Relatively Low
402	10219606	2-Methyl-1-naphthol	-4.603	-5.711	Relatively Low
403	3515	EB	-4.603	-6.534	Relatively Low
404	798	WLN: T5OJ BVO1	-4.599	-6.199	Relatively Low
405	519743	(2S)-heptane-1,2-diol	-4.588	-4.553	Relatively Low
406	14704550	Azulol	-4.585	-6.674	Relatively Low

407	6047	indole	-4.572	-4.94	Relatively Low
408	638014	seychellene	-4.572	-5.397	Relatively Low
409	10847444	rhamnocitrin-3-O-glucoside	-4.569	-5.373	Relatively Low
410	7095	kanzonols L	-4.565	-5.622	Relatively Low
411	5319439	Trans-ionone	-4.563	-6.436	Relatively Low
412	93081	(2S,5R)-2-isopropenyl-5-methyl-1-cyclohexanone	-4.556	-4.803	Relatively Low
413	7362	BNL	-4.556	-5	Relatively Low
414	8554	3'-Methoxyglabridin	-4.541	-4.801	Relatively Low
415	65041	beta-Cubebene	-4.535	-5.108	Relatively Low
416	23677976	Furol	-4.535	-5.108	Relatively Low
417	5281674	Mipax	-6.078	-7.095	Relatively Low
418	124211	DL-Glucuronic acid	-4.526	-5.033	Relatively Low
419	443023	glucuronic acid	-4.524	-5.505	Relatively Low
420	127	Norwogonin	-4.511	-5.547	Relatively Low
421	79028	Skullcapflavone II	-4.51	-5.61	Relatively Low
422	7967	(+)-Syringaresinol	-4.509	-5.036	Relatively Low
423	5281600	4-Carboxymethylphenol	-4.632	-7.081	Relatively Low
424	31404	Heptan	-4.503	-5.727	Relatively Low
425	30248	CYH	-4.499	-5.126	Relatively Low
426	5283468	Amentoflavone	-4.493	-5.387	Relatively Low
427	92158	butylated hydroxytoluene	-4.489	-3.415	Relatively Low
428	325	l-Carvyl acetate	-4.487	-5.858	Relatively Low
429	94275	HMO	-4.485	-5.545	Relatively Low
430	129794163	LUPENONE	-4.488	-5.466	Relatively Low
431	637857	Cuminol	-4.482	-5.753	Relatively Low

432	85567	3691-11-0	-4.479	-5.62	Relatively Low
433	14466553	7-hydroxy-2-methyl-3-phenyl-chromone	-4.466	-5.819	Relatively Low
434	91510	Loniceracetalide A	-4.462	-6.012	Relatively Low
435	348154	cis-Piperitol	-4.454	-5.423	Relatively Low
436	521710	XYLOSTOSIDINE	-4.437	-4.67	Relatively Low
437	90351	Inermine	-4.435	-4.442	Relatively Low
438	15559941	HYKOP	-4.431	-4.403	Relatively Low
439	439263	α-patchoulene	-4.429	-5.555	Relatively Low
440	1549106	Acoradiene	-4.428	-4.838	Relatively Low
441	6436722	isoglabrolide	-4.422	-6.031	Relatively Low
442	10494	()-Neomenthol	-4.425	-4.451	Relatively Low
443	6321405	cis-p-Coumarate	-4.412	-5.926	Relatively Low
444	73659	phytofluene	-4.411	-3.716	Relatively Low
445	107801	oleanolic acid	-4.404	-4.311	Relatively Low
446	11467	(+/-)-Isoborneol	-4.387	-5.909	Relatively Low
447	638072	Maslinic acid	-4.387	-5.732	Relatively Low
448	6544	PHB	-4.386	-5.904	Relatively Low
449	11197	gamma-Terpineol	-4.389	-3.941	Relatively Low
450	3084311	Supraene	-4.383	-5.7	Relatively Low
451	689075	Izoforon	-4.447	-5.003	Relatively Low
452	72321	lignoceric acid	-4.376	-7.437	Relatively Low
453	581460	δ-cadinol	-4.374	-5.117	Relatively Low
454	247	Methyl caffeate	-4.364	-4.553	Relatively Low
455	637542	coptisine	-4.363	-3.889	Relatively Low
456	998	Isothiazole, trimethyl-	-4.359	-5.694	Relatively Low

457	6431015	betaine	-4.355	-3.706	Relatively Low
458	92874	p-coumaric acid	-4.34	-5.354	Relatively Low
459	160767	Hyacinthin	-4.331	-6.565	Relatively Low
460	519382	(-)-alpha-cedrene	-4.32	-4.996	Relatively Low
461	5481949	l-Verbenone	-4.329	-5.368	Relatively Low
462	54715116	isoflavanone	-5.135	-6.352	Relatively Low
463	237332	Dihydro-beta-ionone	-4.305	-4.884	Relatively Low
464	6432469	Gancaonin H	-4.305	-5.42	Relatively Low
465	9895	3,7-dimethyl-cyclopenta cyclooctene	-4.303	-5.499	Relatively Low
466	145742	HMF	-4.301	-5.256	Relatively Low
467	44715835	(-)-isomenthone	-4.298	-7.428	Relatively Low
468	439250	beta-Cyclocitral	-4.29	-5.026	Relatively Low
469	91753455	Prolinum	-4.279	-4.884	Relatively Low
470	5481962	darendoside B_qt	-4.279	-6.41	Relatively Low
471	61041	L-Limonen	-4.278	-5.259	Relatively Low
472	7501	Aromadendrene oxide 2	-4.265	-5.311	Relatively Low
473	442774	Artonin E	-4.265	-6.34	Relatively Low
474	14189465	Safranal	-4.264	-4	Relatively Low
475	107526	styrene	-4.259	-5.144	Relatively Low
476	565709	Hispaglabridin B	-4.257	-5.777	Relatively Low
477	54695756	18α -hydroxyglycyrrhetic acid	-4.254	-5.032	Relatively Low
478	3084282	GLO	-4.249	-3.351	Relatively Low
479	12306047	Aciphyllene	-4.24	-5.606	Relatively Low
480	62566	DHELWANGIN	-4.24	-5.308	Relatively Low

481	227829	Myricadiol	-4.239	-5.882	Relatively Low
482	556516	muurolene	-4.234	-5.742	Relatively Low
483	15560276	beta-Bourbonene	-4.225	-4.967	Relatively Low
484	11230	Guaiol	-4.222	-5.563	Relatively Low
485	8655	2-Caren-10-al	-4.303	-6.071	Relatively Low
486	21648	α-gurjunene	-4.209	-5.142	Relatively Low
487	11463	()-Terpinen-4-ol	-4.207	-5.632	Relatively Low
488	101273201	Syringaldehyde	-4.347	-6.229	Relatively Low
489	16666	Rheosmin	-4.206	-5.316	Relatively Low
490	5374041	Tereben	-4.204	-5.448	Relatively Low
491	196831	2,3,4-Trimethyl-5-phenyloxa zolidine	-4.199	-5.581	Relatively Low
492	108213	MENTHOL	-4.194	-5.481	Relatively Low
493	8575	cinerolon	-4.192	-5.262	Relatively Low
494	102007321	Licoriisoflavan A	-4.184	-5.478	Relatively Low
495	12313020	Bifendate	-4.18	-4.507	Relatively Low
496	442393	MBP	-4.18	-4.842	Relatively Low
497	203797	2,6,10-trimethyl-dodecane	-4.178	-5.54	Relatively Low
498	2879	γ-muurolene	-4.174	-6.02	Relatively Low
499	2345	beta-Selinene	-4.17	-6.109	Relatively Low
500	79812	Sweroside aglycone	-4.157	-4.674	Relatively Low
501	178323	PCR	-4.153	-5.31	Relatively Low
502	480863	BZM	-4.152	-6.291	Relatively Low
503	47124	WLN: VHR	-4.147	-6.15	Relatively Low
504	68313	136458-42-9	-4.146	-5.869	Relatively Low
505	3083590	Kanzonol H	-4.147	-6.3	Relatively Low

506	91747494	2-methyl-5-propyl -nonane	-4.141	-5.136	Relatively Low
507	381152	Hordenine	-4.134	-5.631	Relatively Low
508	61362	(-)-Phillygenin	-4.13	-5.335	Relatively Low
509	5281515	Campherenol	-4.121	-5.251	Relatively Low
510	7302	Piperitenone	-4.12	-4.937	Relatively Low
511	6267	d-Piperitone	-4.116	-4.503	Relatively Low
512	1742210	beta-caryophyllene	-4.103	-5.361	Relatively Low
513	442495	GBL	-4.092	-5.961	Relatively Low
514	176	Crystal VI	-4.093	-3.898	Relatively Low
515	13258	(-)-Epoxycaryophyllene	-4.083	-6.326	Relatively Low
516	10114	Pulegone	-4.08	-3.337	Relatively Low
517	5280794	acetic acid	-4.069	-5.09	Relatively Low
518	159055	3-METHYLPHENANTHRENE	-4.067	-5.082	Relatively Low
519	7463	18beta-glycyrrhetinic acid	-4.061	-5.589	Relatively Low
520	637894	Stigmasterol	-4.058	-5.07	Relatively Low
521	5317844	(S)-camphor	-4.054	-5.273	Relatively Low
522	6209	Cymol	-4.049	-4.884	Relatively Low
523	8766	(R)-()-3-Methylcyclopentanone	-4.049	-5.201	Relatively Low
524	82755	alpha-Guaiene	-4.039	-5.433	Relatively Low
525	6436598	choline	-4.032	-3.687	Relatively Low
526	3362	WLN: RSR	-4.465	-5.695	Relatively Low
527	7127	hydroxytyrosol	-4.021	-4.769	Relatively Low
528	1201518	22β-acetylglabric acid	-4.015	-5.269	Relatively Low
529	161171	Labroda	-4.01	-5.626	Relatively Low

530	22955476	Methyleugenol	-4.003	-5.048	Relatively Low
531	5280581	[(1S)-endo]-(-)-Borneol	-3.999	-4.212	Relatively Low
532	7909	Ephedroxane	-3.992	-5.15	Relatively Low
533	15596633	3,3-Dimethylpentane	-3.977	-5.121	Relatively Low
534	5281862	glyasperins Z	-3.973	-5.564	Relatively Low
535	24135	MIK	-4.714	-4.77	Relatively Low
536	94741	24-Ethylcholest-4-en-3-one	-3.957	-3.593	Relatively Low
537	11265	Urushiol III	-3.944	-5.058	Relatively Low
538	70962	Desaspidinol-A	-3.943	-5.26	Relatively Low
539	439570	Hentriacontan	-3.94	-4.41	Relatively Low
540	7461	2-Methylpentan-3-one	-3.937	-5.604	Relatively Low
541	442402	d-isomenthone	-3.932	-4.59	Relatively Low
542	7892	l-carvone	-3.923	-4.894	Relatively Low
543	7809	Moslene	-3.922	-4.954	Relatively Low
544	444539	widdrene	-3.918	-4.182	Relatively Low
545	26447	Isohexane	-3.916	-5.687	Relatively Low
546	6428535	p-xylene	-3.911	-4.46	Relatively Low
547	7462	cinnamic acid	-3.904	-5.609	Relatively Low
548	570597	l-Menthone	-3.893	-5.517	Relatively Low
549	75546	53111-25-4	-3.883	-3.959	Relatively Low
550	3314	Terpilene	-3.881	-4.567	Relatively Low
551	995	2,3-dimethyl-1-pentene	-3.876	-6.44	Relatively Low
552	12302132	Methyl lignocerate	-3.874	-5.07	Relatively Low
553	11095734	eugenol	-3.87	-4.957	Relatively Low
554	91472	PEY	-3.866	-3.658	Relatively Low
555	6427358	β-bulnesene	-3.865	-4.296	Relatively Low

556	94334	()-Aromadendrene	-3.862	-4.922	Relatively Low
557	3018525	Friedelin	-3.858	-5.009	Relatively Low
558	101031952	[(2S)-6-methylhept-5-en-2-yl] acetate	-3.844	-5.548	Relatively Low
559	14529	6892-80-4	-3.842	-5.775	Relatively Low
560	6918391	PHYTANTRIOL	-3.841	-4.627	Relatively Low
561	22227	2-ethylidene-1,1-dimethyl-cy clopentane	-3.837	-4.721	Relatively Low
562	144514377	p-Cymen-8-ol	-3.834	-5.71	Relatively Low
563	173183	beta-elemene	-3.831	-3.829	Relatively Low
564	6654	d-Dihydrocarvone	-3.827	-5.361	Relatively Low
565	22048	3-methylhexa-2,4-diene	-3.805	-3.917	Relatively Low
566	1796220	campest-5-en-3beta-ol	-3.797	-4.856	Relatively Low
567	87771	(-)-alpha-Pinene	-3.796	-3.99	Relatively Low
568	66841	METHYL HEXACOSANOATE	-3.792	-5.216	Relatively Low
569	138980964	junipene	-3.779	-5.6	Relatively Low
570	119	Trichloroicosylsilane	-3.773	-4.693	Relatively Low
571	15653	beta-Terpinene	-3.77	-5.801	Relatively Low
572	10104370	(5S)-3,5-dimethylcyclohex-2 -en-1-one	-3.76	-4.647	Relatively Low
573	70719	gamma-aminobutyric acid	-3.993	-5.626	Relatively Low
574	637563	2-Ethyl-p-xylene	-3.752	-4.597	Relatively Low
575	9963735	beta-Bisabolene	-3.745	-6.261	Relatively Low
576	15801231	3-Hydroxy-2-picoline	-3.741	-4.532	Relatively Low
577	12310283	anethole	-3.736	-5.253	Relatively Low

578	528127	adhyperforin	-3.736	-4.923	Relatively Low
579	688210	1,8-cineole	-3.734	-5.35	Relatively Low
580	119242	glycyrrhetol	-3.732	-5.004	Relatively Low
581	6560	2,3-DIMETHYLPYRAZINE	-3.725	-4.82	Relatively Low
582	5319562	BOX	-3.778	-5.123	Relatively Low
583	7047	16844-71-6	-3.724	-5.142	Relatively Low
584	81722	iso-Baurenylacetate	-3.711	-4.885	Relatively Low
585	110898	Methyl-p-coumarate	-3.709	-3.948	Relatively Low
586	68057	Leukol	-3.688	-6.104	Relatively Low
587	92221	delta-Terpineol	-3.684	-5.275	Relatively Low
588	10955174	2-isopropenyl-5-methylhex-4-enal	-3.669	-4.208	Relatively Low
589	441298	alpha. -Ionene	-3.668	-5.813	Relatively Low
590	131751571	D-Camphene	-3.659	-3.763	Relatively Low
591	12408	patchouli alcohol	-3.637	-2.153	Relatively Low
592	170833	hyperforin	-3.619	-4.892	Relatively Low
593	5321047	liquoric acid	-3.618	-4.219	Relatively Low
594	11074994	Octacosane	-3.596	-5.236	Relatively Low
595	222284	ISOPULEGOL	-3.591	-4.919	Relatively Low
596	457801	Atractylodin	-3.589	-4.515	Relatively Low
597	14353410	(+)-Ledol	-3.585	-4.868	Relatively Low
598	92173967	beta-sitosterol	-3.585	-4.59	Relatively Low
599	12048	poriferast-5-en-3beta-ol	-3.573	-4.828	Relatively Low
600	637511	rengyoxide	-3.568	-4.294	Relatively Low
601	8892	(4S)-2,4-dimethylhexane	-3.571	-3.02	Relatively Low
602	443177	3-Ethylpentane	-3.566	-5.684	Relatively Low

603	64971	cinnamaldehyde	-3.562	-3.032	Relatively Low
604	10393	hexanoic acid	-3.551	-5.081	Relatively Low
605	61450	(4S,6S)-cis-Carveol	-3.531	-4.332	Relatively Low
606	73299	Mairin	-3.531	-3.316	Relatively Low
607	66540	Tyrosol	-3.522	-4.553	Relatively Low
608	76969264	ETHYL FURAN	-3.517	-5.416	Relatively Low
609	268208	hederagenin	-3.516	-5.46	Relatively Low
610	5366074	Truflex OBP	-3.513	-4.784	Relatively Low
611	101596917	(2S,4S)-4-methyl-2-(2-methylprop-1-enyl) oxane	-3.506	-4.244	Relatively Low
612	931	7-Acetoxy-2-methylisoflavone	-3.504	-5.723	Relatively Low
613	7296	Damascenone	-3.493	-4.883	Relatively Low
614	13584	(β-maaliene)	-3.49	-4.265	Relatively Low
615	7237	naphthalene	-3.477	-4.919	Relatively Low
616	13572	Methylcyclopentane	-3.462	-3.964	Relatively Low
617	10467	Methyl behenate	-3.456	-3.419	Relatively Low
618	443163	o-xylene	-3.448	-4.779	Relatively Low
619	14296	6-Methylheptan-2-one	-3.442	-4.286	Relatively Low
620	443162	Arachic acid	-3.436	-4.949	Relatively Low
621	17100	(1R,2R,4R)-Dihydrocarveol	-3.436	-5.161	Relatively Low
622	1549025	tetramethylpyrazine	-3.426	-4.406	Relatively Low
623	75519	(L)-alpha-Terpineol	-3.426	-4.224	Relatively Low
624	8034	()-alpha-Terpineol	-3.419	-4.176	Relatively Low
625	564	NERYLACETATE	-3.414	-4.157	Relatively Low
626	19602	Methyl tricosanoate	-3.411	-3.642	Relatively Low

627	8748	MIAK	-3.4	-5.304	Relatively Low
628	11412	hexanoic acid	-3.39	-4.259	Relatively Low
629	14498	PENTYLFURAN	-3.388	-5.178	Relatively Low
630	6432404	β-terpineol	-3.365	-5.513	Relatively Low
631	6054	2,3-dimethylhexane	-3.361	-5.658	Relatively Low
632	14259	(1S,2S)-1,2-dimethylcyclopentane	-3.348	-3.976	Relatively Low
633	20240	CADINENE	-3.351	-5.524	Relatively Low
634	7962	PEL	-3.302	-5.097	Relatively Low
635	5315649	Methyl icosanoate	-3.295	-4.022	Relatively Low
636	17886	Isobutyl benzoate	-3.284	-5.268	Relatively Low
637	8094	Sextone B	-3.279	-2.628	Relatively Low
638	33934	gamma-Camphorene	-3.245	-5.775	Relatively Low
639	5368821	asernestioside B	-3.209	-4.483	Relatively Low
640	7907	heptanoic acid	-3.183	-5.161	Relatively Low
641	5352451	Diop	-3.151	-4.882	Relatively Low
642	7282	Allocymene	-3.142	-4.825	Relatively Low
643	638011	2,2-DIMETHYLPENTANE	-3.125	-3.563	Relatively Low
644	15314349	patchoulan 1,12-diol	-3.12	-3.956	Relatively Low
645	439202	3-Methylpentane	-3.26	-3.501	Relatively Low
646	519960	citral	-3.103	-4.77	Relatively Low
647	122844	(3S)-2,3-dimethylpentane	-3.067	-3.215	Relatively Low
648	5460988	GGB	-3.057	-3.574	Relatively Low
649	12613	()-Cyclosativene	-3.046	-4.126	Relatively Low
650	26519	Betulonic acid	-3.037	-3.254	Relatively Low
651	5460660	gadelaidic acid	-3.033	-3.908	Relatively Low

652	6549	Apple oil	-3.025	-3.88	Relatively Low
653	6344	Tetratriacontane	-3.025	-3.977	Relatively Low
654	440917	Docosanoate	-3.025	-5.018	Relatively Low
655	16330	Linalool	-3.023	-4.652	Relatively Low
656	1549778	WLN: G1G	-3.015	-3.983	Relatively Low
657	6782	Hemo-sol	-2.971	-4.493	Relatively Low
658	61665	WLN: 4OVR	-2.937	-4.651	Relatively Low
659	8842	Geranylacetone	-2.912	-4.266	Relatively Low
660	18635	DIBP	-2.899	-4.068	Relatively Low
661	5320251	Dehydroxylinalool oxide A	-2.888	-3.896	Relatively Low
662	12407	beta-Citronellol	-2.878	-3.444	Relatively Low
663	61738	Methional	-2.869	-4.737	Relatively Low
664	68406	Octacosanedioic acid	-2.864	-3.019	Relatively Low
665	8051	HEXACOSANE	-2.815	-3.406	Relatively Low
666	74458	2-Hexanoylfuran	-2.813	-4.334	Relatively Low
667	1549019	Octacosanol	-3.212	-4.834	Relatively Low
668	5280489	2-heptanone	-2.794	-4.69	Relatively Low
669	637566	Allyloxybenzene	-2.79	-4.16	Relatively Low
670	57491032	3-Methyl-2-pent-2-enyl-cyclopent-2-enone	-2.789	-3.752	Relatively Low
671	5367807	beta-carotene	-2.787	-3.881	Relatively Low
672	8723	geraniol	-2.771	-3.828	Relatively Low
673	5282109	3,7-dimethylnonane	-2.748	-4.138	Relatively Low
674	16057860	Isobutyl tiglate	-2.682	-2.604	Relatively Low
675	15610	2-methyl-butanol-1-ol	-2.673	-4.335	Relatively Low
676	5283335	Geranyl formate	-2.667	-1.836	Relatively Low

677	28928	Ginnol	-2.63	-4.261	Relatively Low
678	637920	METHYL NONADECANOATE	-2.61	-3.15	Relatively Low
679	169019	trans-2-nonenal	-2.579	-3.149	Relatively Low
680	11173	kanzonols T	-2.543	-3.856	Relatively Low
681	13143	pent-3-en-2-one	-2.535	-3.229	Relatively Low
682	8344	Threitol	-2.525	-3.25	Relatively Low
683	5282184	Prenol	-2.511	-3.562	Relatively Low
684	9862	Methyl butenone	-2.504	-3.659	Relatively Low
685	102247659	DMEP	-2.456	-3.72	Relatively Low
686	5281553	Mandenol	-2.452	-3.404	Relatively Low
687	12534	Sulcatone	-2.436	-3.355	Relatively Low
688	5367460	1,3-Heptadiene, 3-ethyl-2-methyl-	-2.417	-4.735	Relatively Low
689	5281168	p-Ocimene	-2.416	-2.733	Relatively Low
690	5318042	Tricosane	-2.39	-3.402	Relatively Low
691	5321950	Ethyl linolenate	-2.37	-3.138	Relatively Low
692	11251	Hexenal	-2.363	-3.385	Relatively Low
693	12592	2-Hexenol	-2.36	-3.205	Relatively Low
694	61020	Tiglaldehyde	-2.332	-3.364	Relatively Low
695	24762	MIPK	-2.331	-4.689	Relatively Low
696	637564	Tetracosane	-2.328	-2.169	Relatively Low
697	6184	Prenal	-2.306	-2.759	Relatively Low
698	68972	MYS	-2.303	-3.621	Relatively Low
699	3026	Hexadienal	-2.287	-3.08	Relatively Low
700	11519	hexanal	-2.265	-3.183	Relatively Low

701	11636	n-Triacontanol	-2.248	-3.16	Relatively Low
702	643820	DBP	-2.234	-4.678	Relatively Low
703	10975	3-methylheptane	-2.233	-2.851	Relatively Low
704	2724898	HEPTACOSANE	-2.233	-2.267	Relatively Low
705	12409	Nerol	-2.199	-2.466	Relatively Low
706	6431151	Myrcenol	-2.182	-3.714	Relatively Low
707	243696	(S)-Matsutake alcohol	-2.153	-2.455	Relatively Low
708	11507	Nonacosane	-2.132	-3.84	Relatively Low
709	8129	(-)-cis-beta-Elemene	-2.118	-2.073	Relatively Low
710	638122	Nonacosanol	-2.097	-2.148	Relatively Low
711	31272	3-methylhexane	-2.089	-2.984	Relatively Low
712	263	Heptanol	-2.073	-3.16	Relatively Low
713	1549026	C2H5CH=CHCOOH	-2.017	-4.409	Relatively Low
714	25403	Butylacetat	-2.002	-3.728	Relatively Low
715	92776	BuOH	-1.959	-4.484	Relatively Low
716	11582	Neryl acetate	-1.942	-3.213	Relatively Low
717	5281516	Ethyltrimethylethylene	-1.936	-1.672	Relatively Low
718	6561	Zingiberene	-1.904	-2.602	Relatively Low
719	8130	ISOHEPTANE	-1.895	-2.648	Relatively Low
720	246728	alpha-Farnesene	-1.887	-3.168	Relatively Low
721	94403	Isobutyral	-1.869	-3.508	Relatively Low
722	5283316	WLN: VH6	-1.857	-2.372	Relatively Low
723	5364961	EAK	-1.818	-3.037	Relatively Low
724	22434	Farnesol acetate	-1.815	-4.378	Relatively Low
725	6584	Heptenal	-1.81	-2.35	Relatively Low
726	5362851	(E)-1-butoxyhex-2-ene	-1.806	-3.25	Relatively Low

727	379	methyl henicosanoate	-1.792	-2.355	Relatively Low
728	143243	Tereton	-1.737	-4.505	Relatively Low
729	8158	2-Methylpent-2-en-1-ol	-1.713	-2.909	Relatively Low
730	31253	caprylic acid	-1.688	-2.964	Relatively Low
731	445070	1,6-Dicyclohexylhexane	-1.666	-1.819	Relatively Low
732	7793	nonanoic acid	-1.558	-4.266	Relatively Low
733	15395	Myrcene	-1.499	-2.786	Relatively Low
734	5280934	farnesol	-1.417	-1.956	Relatively Low
735	6276	beta-Rhodinol	-1.396	-2.744	Relatively Low
736	7826	Propyl vinyl ketone	-1.374	-2.601	Relatively Low
737	1549109	linolenic acid	-1.347	-2.071	Relatively Low
738	8091	Amylol	-1.334	-2.918	Relatively Low
739	5364920	Methyl heptoate	-1.317	-2.283	Relatively Low
740	11594	(Z,E)-farnesol	-1.273	-2.684	Relatively Low
741	1711945	Methyl octylate	-1.25	-3.055	Relatively Low
742	61346	(E)-Pent-2-en-1-ol	-1.203	-2.068	Relatively Low
743	5281525	Methylheptane	-1.194	-1.064	Relatively Low
744	31289	Farnesylacetone	-1.189	-2.4	Relatively Low
745	5356544	Vinyl amyl ketone	-1.188	-2.609	Relatively Low
746	8103	C09704	-1.187	-2.352	Relatively Low
747	517653	Nonanal	-1.151	-2.203	Relatively Low
748	5364919	Peruviol	-1.107	-2.638	Relatively Low
749	87370	1-hexanol	-1.007	-2.233	Relatively Low
750	31357	Octadiene	-0.951	-2.014	Relatively Low
751	1549107	CIS-2-PENTENOL	-0.91	-1.905	Relatively Low
752	6442707	1-Ethoxypentane	-0.883	-1.489	Relatively Low

753	10408	TBP	-0.883	-2.353	Relatively Low
754	21414	(Z,Z)-farnesol	-0.697	-1.121	Relatively Low
755	957	Safynol	-0.566	-2.611	Relatively Low
756	5281167	FITONE	-0.541	-1.908	Relatively Low
757	5284421	2-octyldodecan-1-ol	-0.477	-1.886	Relatively Low
758	5280435	octanol	-0.405	-2.683	Relatively Low
759	98299	3-Hexenol	-0.397	-2.093	Relatively Low
760	702	METHYL LINOLEATE	-0.303	-0.372	Relatively Low
761	5362889	phytol	-0.249	-1.293	Relatively Low
762	78062472	2-bromododecane	-0.223	-1.972	Relatively Low
763	70214	3-ethyl-7hydroxyphthalide	-0.222	-2.542	Relatively Low
764	11597	cis-.alpha.-Farnesene	-0.216	-1.696	Relatively Low
765	5281149	3,8-dimethylundecane	-0.18	-1.985	Relatively Low
766	17903416	Diisobutyl succinate	-0.102	-1.279	Relatively Low
767	522345	Hexene	0.02	-0.911	Relatively Low
768	69527	panaxynol	0.042	-3.329	Relatively Low
769	5271570	12-methyltetradecanoate	0.081	-1.812	Relatively Low
770	5281	Methyl isoheptadecanoate	0.129	-0.74	Relatively Low
771	105846	Lauric anhydride	0.147	-0.113	Relatively Low
772	5368064	DBF	0.154	-0.519	Relatively Low
773	12366	stearic acid	0.184	-1.704	Relatively Low
774	10446	heptadecyloxirane	0.211	-1.415	Relatively Low
775	5364509	Methyl 2-decenoate	0.242	-1.35	Relatively Low
776	445639	Ethylpalmitate	0.38	-0.566	Relatively Low
777	8042	neophytadiene	0.445	-0.75	Relatively Low
778	5463904	methyl (E)-octadec-2-enoate	0.524	-0.894	Relatively Low

779	8181	oleic acid	0.596	0.543	Relatively Low
780	8201	Promyr	0.601	-1.012	Relatively Low
781	12398	Ethyl 2-decenoate	0.636	1.679	Relatively Low
782	15267	methyl palmitate	0.661	0.482	Relatively Low
783	985	Methyl stearate	0.695	-0.582	Relatively Low
784	11005	Heptadekan	0.71	-0.258	Relatively Low
785	15609	2-METHYLPENTADECAN E	0.744	-0.683	Relatively Low
786	638303	palmitic acid	0.764	-0.462	Relatively Low
787	8139	myristic acid	0.784	-0.241	Relatively Low
788	3893	Methyl margarate	0.817	0.104	Relatively Low
789	5367650	Methyl palmitelaidate	1.103	-0.282	Relatively Low
790	5362793	Methyl laurate	1.119	-1.193	Relatively Low
791	21204	lauric acid	1.134	-0.79	Relatively Low
792	75364	13-Tetradecenyl acetate	1.137	-0.019	Relatively Low
793	13849	Methyl linolelaidate	1.242	-0.496	Relatively Low
794	61303	Methyl isomyristate	1.666	-0.446	Relatively Low
795	12403	2-Tetradecanone	1.768	0.423	Relatively Low
796	8186	PENTADECYLIC ACID	1.877	0.715	Relatively Low
797	5283356	2-PENTADECANONE	1.971	0.833	Relatively Low
798	11635	Henicosane	2.046	0.588	Relatively Low
799	25913	undecanal	2.052	1.464	Relatively Low
800	31284	Undecenal	2.286	0.107	Relatively Low
801	15099203	Oktadekan	2.514	0.56	Relatively Low
802	143689	Pentadecene	2.598	1.588	Relatively Low
803	8182	Methyl myristate	2.608	1.334	Relatively Low

804	12388	1,4-Eicosadiene	2.624	1.554	Relatively Low
805	12397	3-Undecyne	2.631	1.498	Relatively Low
806	12389	Dodekan	2.766	0.357	Relatively Low
807	14257	TRD	2.998	0.865	Relatively Low
808	138108061	Pentadecanol	-6.474	-7.898	Relatively Low
809	121304016	tetradecane	-6.754	-8.738	The Best Control within 6LU7
810	92727	UND	-7.037	-7.794	Control
811	131411	Macranthoside B	-3.934	-6.487	Control
812	37542	Remdesivir	-5.977	-6.159	Control
813	64927	Lopinavir	-4.634	-5.936	Control
814	492405	Arbidol	-5.489	-5.313	Control
815	503737	Ribavirin	-7.41	-6.627	Relatively Low
816	9064	Chloroquine Phosphate	-6.392	-6.945	Relatively Low
817	441957	Favipiravir（*T705*）	-6.288	-4.631	Relatively Low
818	14982	liquiritin	-6.212	-5.107	Relatively Low
819	892	(+)-catechin	-6.106	-5.934	Relatively Low
820	5320083	Ziziphin	-5.994	-6.171	Relatively Low
821	7067335	Glycyram	-5.865	-6.421	Relatively Low
822	5318999	inositol	-5.807	-6.516	Relatively Low
823	656516	Glycyrol	-5.703	-6.609	Relatively Low
824	44257530	terephthaldehyde	-5.652	-6.541	Relatively Low
825	439767	Licochalcone B	-5.649	-5.492	Relatively Low
826	440989	Amygdalin	-5.535	-7.252	Relatively Low
827	638278	Phaseol	-5.52	-8.363	Relatively Low
828	1794427	MNN	-5.479	-5.887	Relatively Low
829	9548674	Machiline	-5.407	-6.957	Relatively Low

830	244	isoliquiritigenin	-5.385	-5.85	Relatively Low
831	6917970	Heriguard	-5.198	-6.164	Relatively Low
832	5757	d-mandelonitrile	-4.972	-7.132	Relatively Low
833	119033	WLN: Q1R	-4.955	-6.729	Relatively Low
834	5497163	l-SPD	-4.917	-3.651	Relatively Low
835	240	17-beta-estradiol	-4.911	-4.975	Relatively Low
836	5281522	prunasin	-4.905	-4.445	Relatively Low
837	124052	Olein	-4.848	-6.603	Relatively Low
838	6918970	WLN: VHR	-4.845	-6.539	Relatively Low
839	12303645	Isocaryophyllene	-4.818	-4.822	Relatively Low
840	94253	Glabridin	-4.811	-5.573	Relatively Low
841	18183610	estrone	-4.765	-5.258	Relatively Low
842	6552009	sitosterol	-4.641	-5.238	Relatively Low
843	638014	Vulgarin	-4.572	-5.397	Relatively Low
844	11579	WLN: QVR BVQ	-4.513	-4.334	Relatively Low
845	31404	()-Borneol	-4.503	-5.727	Relatively Low
846	5325911	beta-Ionone	-4.436	-5.735	Relatively Low
847	11975273	LEVA	-4.426	-4.175	Relatively Low
848	64945	butylated hydroxytoluene	-4.383	-5.183	Relatively Low
849	17739	(5S)-5-ethyloxolan-2-one	-4.263	-5.078	Relatively Low
850	16666	(6Z,10E,14E,18E)-2,6,10,15,19,23-hexamethyltetracosa-2,6,10,14,18,22-hexaene	-4.206	-5.316	Relatively Low
851	2724161	ursolic acid	-4.104	-5.563	Relatively Low
852	442495	Cerulignol	-4.092	-5.961	Relatively Low
853	5280794	MENTHOL	-4.069	-5.09	Relatively Low

854	5997	()-Terpinen-4-ol	-3.912	-5.051	Relatively Low
855	5281331	Pulegone	-3.898	-5.911	Relatively Low
856	3314	Stigmasterol	-3.881	-4.567	Relatively Low
857	5282805	CLR	-3.754	-4.361	Relatively Low
858	7057904	Spinasterol	-3.678	-4.914	Relatively Low
859	12408	eugenol	-3.637	-2.153	Relatively Low
860	64971	11,14-eicosadienoic acid	-3.562	-3.032	Relatively Low
861	768	(5S)-5-hexyloxolan-2-one	-3.526	-3.757	Relatively Low
862	10467	Octacosane	-3.456	-3.419	Relatively Low
863	5282768	Mairin	-3.439	-4.561	Relatively Low
864	443162	[CHN]	-3.436	-4.949	Relatively Low
865	19602	Arachic acid	-3.411	-3.642	Relatively Low
866	5312549	gondoic acid	-3.357	-4.28	Relatively Low
867	77914	(L)-alpha-Terpineol	-3.173	-3.243	Relatively Low
868	26519	PENTYLFURAN	-3.037	-3.254	Relatively Low
869	8215	11-docosenoic acid	-3.033	-3.908	Relatively Low
870	6782	6-Oxooctanoic acid	-2.971	-4.493	Relatively Low
871	12407	Tetratriacontane	-2.878	-3.444	Relatively Low
872	12406	Docosanoate	-2.86	-3.277	Relatively Low
873	443158	DIBP	-2.812	-3.88	Relatively Low
874	637566	HEXACOSANE	-2.79	-4.16	Relatively Low
875	11008	PENTACOSANE	-2.757	-3.352	Relatively Low
876	12535	(R)-linalool	-2.484	-3.673	Relatively Low
877	12534	geraniol	-2.436	-3.355	Relatively Low
878	5281168	Bicetyl	-2.416	-2.733	Relatively Low
879	5318042	TRIACONTANE	-2.39	-3.402	Relatively Low

880	12592	Tricosane	-2.36	-3.205	Relatively Low
881	5283349	Hexenal	-2.341	-2.997	Relatively Low
882	120074	2-Hexenol	-2.333	-5.327	Relatively Low
883	6184	Tetracosane	-2.306	-2.759	Relatively Low
884	3026	trans-2,4-decadienal	-2.287	-3.08	Relatively Low
885	11636	Diisooctyl succinate	-2.248	-3.16	Relatively Low
886	12409	hexanal	-2.199	-2.466	Relatively Low
887	12405	DBP	-2.178	-2.945	Relatively Low
888	5283316	HEPTACOSANE	-1.857	-2.372	Relatively Low
889	12413	Nonacosane	-1.79	-3.442	Relatively Low
890	12523	TWT	-1.7	-2.339	Relatively Low
891	12411	Heptenal	-1.657	-3.613	Relatively Low
892	5280934	Pentatriacontane	-1.417	-1.956	Relatively Low
893	31289	phytane	-1.189	-2.4	Relatively Low
894	8103	Tritriacontane	-1.187	-2.352	Relatively Low
895	12410	linolenic acid	-1.093	-2.454	Relatively Low
896	12575964	Nonanal	-1.003	-0.458	Relatively Low
897	1810797	1-hexanol	-0.568	-1.656	Relatively Low
898	957	Hentriacontan	-0.566	-2.611	Relatively Low
899	5284421	2-Hydroxy-hexadecanoic acid	-0.477	-1.886	Relatively Low
900	5282760	ZINC02169908	-0.427	-1.194	Relatively Low
901	5280435	octanol	-0.405	-2.683	Relatively Low
902	15979	METHYL LINOLEATE	-0.192	-2.183	Relatively Low
903	5280450	Isooleic acid	-0.085	-2.122	Relatively Low
904	445638	phytol	0.03	-0.412	Relatively Low

905	5281	pristane	0.129	-0.74	Relatively Low
906	5364509	EIC	0.242	-1.35	Relatively Low
907	520298	zoomaric acid	0.322	-1.067	Relatively Low
908	445639	stearic acid	0.38	-0.566	Relatively Low
909	10465	Exceparl M-OL	0.451	-0.678	Relatively Low
910	8181	Aseanostatin P1	0.596	0.543	Relatively Low
911	8201	oleic acid	0.601	-1.012	Relatively Low
912	12398	Daturic acid	0.636	1.67	Relatively Low
913	985	methyl palmitate	0.695	-0.582	Relatively Low
914	11005	Methyl stearate	0.71	-0.258	Relatively Low
915	3893	Heptadekan	0.817	0.104	Relatively Low
916	5282728	palmitic acid	1.08	0.53	Relatively Low
917	12391	myristic acid	1.225	0.876	Relatively Low
918	12401	lauric acid	1.309	-0.17	Relatively Low
919	12403	2-undecenoic acid	1.768	0.423	Relatively Low
920	8222	MYS	1.896	-0.437	Relatively Low
921	11635	UPL	2.046	0.588	Relatively Low
922	11006	Henicosane	2.31	1.436	Relatively Low
923	5283345	LFA	2.426	1.163	Relatively Low
924	12388	Oktadekan	2.624	1.554	Relatively Low
925	12389	hexadecane	2.766	0.357	Relatively Low
926	8914	dec-2-enal	3.1	1.276	Relatively Low

919 unique ingredients of the 8 candidates were extracted from TCMSP and PubChem (PubChem CID and Molecular Name were attached here in details), and these all developed valid docking terminally. All docking results, besides seven positive controls, were showed in glide gscore (the lower, the better). In

addition, the notes were attached here for indicating the results contrasted with the best control (Ritonavir to 6VSB: -7.828 kcal/mol while Remdesivir to 6LU7: -8.738 kcal/mol). This included High in 6VSB (< -7.828 kcal/mol) / 6LU7 (< -8.738 kcal/mol), High in both (high affinity in both), Relatively Low (lower efficiency of the both), Control, The Best Control within 6VSB and The Best Control within 6LU7. The preferable results were listed by priority according to the docking results.

Table S9. Preferable docking results among 2042 natural ingredients.

NO.	Compounds CID	Compounds Name	Docking Gscore (kcal/mol) 6LU7#	Docking Gscore (kcal/mol) 6VSB#	Label in Fig 4I	Source Herbs (Abbreviation (Latin Name, Chinese Pinyin))
1	9987332	Sanggenone D	-9.14	-7.509	Blue Dot (High in 6LU7)	SGen (Morus alba L., Sang Gen)
2	124222341	Tectorigenin 7-O-xylosylglucoside	-8.798	-8.089	Blue Dot (High in 6LU7)	YX (Folium Ginkgo, Yin Xing)
3	10169367	Quercetin 3-O-β-D-(6"-p- coumaroyl) glucopyranosyl (1- 2)-α-L-rhamnopyranoside	-9.782	-7.506	Blue Dot (High in 6LU7)	Black Tea
4	137119925	Theaflavin-3-gallate	-9.149	-7.468	Blue Dot (High in 6LU7)	JL (Tribulus terrestris L. , Ji Li)
5	137796485	Quercetin 3-O-β-D-glucose-7-O-β-D-gentiobioside	-9.674	-7.433	Blue Dot (High in 6LU7)	YYH (*E.brevicornum* Maxim., Yin Yang Huo)
6	92043273	Epimedin A1	-9.633	-7.382	Blue Dot (High in 6LU7)	JXC (*Centella asiatica* (L.) Urban, Ji Xue Cao)
7	45356919	Madecassoside	-8.917	-7.346	Blue Dot (High in 6LU7)	Black Tea
8	71307578	Theaflavin-3'-gallate	-10.328	-7.228	Blue Dot (High in 6LU7)	SDH (Radix Rehmanniae Recens, Sheng Di Huang)
9	5281782	Jionoside B1	-8.951	-7.169	Blue Dot (High in 6LU7)	ZJNZ (Fructus Ligustri Lucidi, Zi Jing Nv Zhen)

10	10101498	Ligupurpuroside B	-9.921	-7.09	Blue Dot (High in 6LU7)	DGP (Cortex Lycii, Di Gu Pi)
11	10346914	Kukoamine B	-8.925	-6.916	Blue Dot (High in 6LU7)	YZ (Polygala tenuifolia Willd, Yuan Zhi)
12	46933844	Tenuifoliside A	-8.871	-6.843	Blue Dot (High in 6LU7)	LQ (Forsythiae Fructus, Lian Qiao)
13	23958169	Isoforsythiaside	-9.92	-6.763	Blue Dot (High in 6LU7)	LLY (Sauropus spatulifolius Beille , Long Li Ye)
14	9960512	Kaempferol 3-O-gentiobioside	-8.754	-6.469	Blue Dot (High in 6LU7)	HM (Flos Sophorae Immaturus, Huai Mi)
15	5280805	Rutin	-9.225	-6.377	Blue Dot (High in 6LU7)	HM (Flos Sophorae Immaturus, Huai Mi)
16	5318767	Kaempferol-3-Rutinoside	-8.966	-5.972	Blue Dot (High in 6LU7)	HH (Sophora Japonica L., Huai Hua), YX (Folium Ginkgo, Yin Xing)
17	5320863	Quercetin 3-O-β-D- xylopyranoside	-8.934	-5.671	Blue Dot (High in 6LU7)	RS (Radix Ginseng, Ren Shen)
18	9986191	Panasenoside	-8.786	-5.444	Blue Dot (High in 6LU7)	KS (Sophora flavescens Ait., Ku Shen)
19	72936	Kushenol F	-9.202	-5.218	Blue Dot (High in 6LU7)	BGZ (Psoraleae Fructus, Bu Gu Zhi)
20	193679	Isobavachin	-8.895	-4.843	Blue Dot (High in 6LU7)	ZS (Fructus Aurantii Immaturus, Zhi Shi)
21	439246	Naringenin	-8.764	-4.79	Blue Dot (High in 6LU7)	RCR (Cistanches Herba, Rou Cong Rong)
22	21637830	Tubuloside A	-9.104	-3.753	Blue Dot (High in 6LU7)	Black Tea
23	118718881	Acetylarenobufagin	-6.616	-10.954	Green Dot (High in 6VSB)	CS (Bufonis Venenum, Chan Su)
24	137796326	Ligupurpuroside C	-6.615	-10.116	Green Dot (High in 6VSB)	ZJNZ (Fructus Ligustri Lucidi, Zi Jing Nv Zhen)

25	51346147	Pseudoprotodioscin	-4.983	-9.713	Green Dot (High in 6VSB)	CSL (Dioscorea nipponicaMakino, Chuan Shan Long)
26	71307448	Cistanoside A	-8.044	-9.636	Green Dot (High in 6VSB)	RCR (Cistanches Herba, Rou Cong Rong)
27	441885	Protodeltonin	-6.709	-9.42	Green Dot (High in 6VSB)	/
28	442439	Neohesperidin	-7.682	-9.041	Green Dot (High in 6VSB)	CP (Pericarpium Citri Reticulatae, Chen Pi)
29	53462233	Anemarsaponin B	-7.008	-8.863	Green Dot (High in 6VSB)	ZM (Rhizoma Anemarrhenae, Zhi Mu)
30	75061243	Parishin B	-8.173	-8.758	Green Dot (High in 6VSB)	TM (Gastrodiae Rhizoma, Tian Ma)
31	14037387	Tubeimoside II	-4.594	-8.745	Green Dot (High in 6VSB)	TBM (Rhizoma Bolbostemmatis, Tu Bei Mu)
32	5320686	Tiliroside	-7.936	-8.739	Green Dot (High in 6VSB)	JXH (Edgeworthia chrysantha Lindl., Jie Xiang Hua)
33	53317652	Platycodin D2	-7.474	-8.665	Green Dot (High in 6VSB)	JG (Radix Platycodonis, Jie Geng)
34	441892	Protogracillin	-7.41	-8.642	Green Dot (High in 6VSB)	CSL (Dioscorea nipponicaMakino, Chuan Shan Long)
35	51346169	Jujuboside A	-5.393	-8.64	Green Dot (High in 6VSB)	SZR (Ziziphi Spinosae Semen, Suan Zao Ren)
36	71571492	Clinodiside A	-6.827	-8.61	Green Dot (High in 6VSB)	DXL (Cholla Stem Caulis Opuntiae, Duan Xue Liu)
37	5281693	Robinin	-8.562	-8.604	Green Dot (High in 6VSB)	YJH (Dendranthema indicum, Ye Ju Hua)
38	14189963	Rubrofusarin gentiobiosid	-8.013	-8.593	Green Dot (High in 6VSB)	JMZ (Catsia tora Linn, Jue Ming Zi)

39	5274591	Isoliensinine	-7.855	-8.577	Green Dot (High in 6VSB)	LZX (Nelumbon nucifera Gaertn, Lian Zi Xin)
40	6442411	Poliumoside	-8.592	-8.556	Green Dot (High in 6VSB)	ZZ (Callicarpa dichotoma, Zi Zhu)
41	11491905	Hederacoside C	-6.554	-8.55	Green Dot (High in 6VSB)	CCT (Hedera nepalensis var. sinensis (Tobl.) Rehd, Chang Chun Teng)
42	71307558	Anemoside B4	-7.364	-8.515	Green Dot (High in 6VSB)	BTW (Pulsatillae Radix, Bai Tou Weng)
43	46887681	Gypenoside IX	-6.174	-8.515	Green Dot (High in 6VSB)	JGL (Pulsatillae Radix, Jiao Gu Lan)
44	101568804	Grosvenorine	-6.198	-8.489	Green Dot (High in 6VSB)	LHG (Mormordica grosvenorii Swingle, Luo Han Guo)
45	69964214	Neodiosmin	-7.313	-8.482	Green Dot (High in 6VSB)	/
46	169853	Procyanidin C1	-6.794	-8.475	Green Dot (High in 6VSB)	SZ (Crataegus L., Shan Zha)
47	75412556	Notoginsenoside Fc	-7.111	-8.407	Green Dot (High in 6VSB)	SQ (Notoginseng Radix et Rhizoma , San Qi)
48	2724385	Digoxin	-6.316	-8.406	Green Dot (High in 6VSB)	/
49	127256227	Polygalacin D	-7.323	-8.404	Green Dot (High in 6VSB)	YZ (Polygalae Radix, Yuan Zhi)
50	442428	Naringin	-6.891	-8.371	Green Dot (High in 6VSB)	ZS (*Citrus* aurantium L., Zhi Shi)
51	135403795	Theaflavine-3,3'-digallate	-7.814	-8.329	Green Dot (High in 6VSB)	Black Tea
52	100941542	Ginsenoside Ra1	-6.627	-8.316	Green Dot (High in 6VSB)	RS (Radix Ginseng, Ren Shen)
53	101389834	Anemarsaponin C	-6.355	-8.266	Green Dot (High in 6VSB)	ZM (Anemarrhenae Rhizoma, Zhi Mu)
54	133561676	Ophiopogonin C	-5.611	-8.262	Green Dot (High in 6VSB)	MD (Radix Ophiopogonis, Mai Dong)
55	441891	Protodioscin	-6.369	-8.257	Green Dot (High in 6VSB)	CSL (Dioscorea nipponicaMakino,

						Chuan Shan Long)
56	71307562	Congmunoside VII	-6.13	-8.238	Green Dot (High in 6VSB)	CM (Aralia chinensis, Cong Mu)
57	134687322	HuangjiangSu A	-5.637	-8.216	Green Dot (High in 6VSB)	JH (Rhizoma Curcumae Longae, Jiang Huang)
58	24177534	2,3,4,5-Tetracaffeoyl-D- Glucaric acid	-6.82	-8.153	Green Dot (High in 6VSB)	XFH (Inula japonica Thunb., Xuan Fu Hua)
59	15940177	vina-ginsenoside R4	-4.752	-8.147	Green Dot (High in 6VSB)	RS (Radix Ginseng, Ren Shen)
60	14681435	Arctigenin 4'-O-β-gen	-8.578	-8.135	Green Dot (High in 6VSB)	NBZ (Fructus Arctii, Niu Bang Zi)
61	71307572	Polyphyllin G	-7.251	-8.12	Green Dot (High in 6VSB)	CL (Rhizoma Paridis, Chong Lou)
62	442456	Poncirin	-6.276	-8.1	Green Dot (High in 6VSB)	ZS (*Citrus* aurantium L., Zhi Shi)
63	10557926	Parishin A	-6.667	-8.09	Green Dot (High in 6VSB)	TM (Gastrodiae Rhizoma, Tian Ma)
64	44575945	Timosaponin B II	-7.287	-8.075	Green Dot (High in 6VSB)	ZM (Anemarrhenae Rhizoma, Zhi Mu)
65	11993928	Acanthopanaxoside B	-6.22	-8.07	Green Dot (High in 6VSB)	CCT (Hedera nepalensis var. sinensis (Tobl.) Rehd, Chang Chun Teng)
66	176233	Polyphyllin VII	-6.546	-8.055	Green Dot (High in 6VSB)	CL (Rhizoma Paridis, Chong Lou)
67	46200821	Polyphyllin D	-7.078	-8.049	Green Dot (High in 6VSB)	CL (Rhizoma Paridis, Chong Lou)
68	44575944	Anemarsaponin BIII	-5.294	-8.039	Green Dot (High in 6VSB)	ZM (Rhizoma Anemarrhenae, Zhi Mu)
69	6325450	Jionoside A1	-8.727	-8.029	Green Dot (High in 6VSB)	SDH (Radix Rehmanniae Recens, Sheng Di Huang)
70	11828754	Alcesefoliside	-8.139	-7.976	Green Dot (High in 6VSB)	XGZ (Rubus corchorifolius L.f., Xuan Gou Zi)
71	14564503	Macranthoidin A	-7.597	-7.959	Green Dot (High in 6VSB)	SYH (Lonicera confusa DC., Shan

						Yin Hua)
72	71307447	Ciwujianoside B	-6.41	-7.953	Green Dot (High in 6VSB)	CWJ (Radix Acanthopanacis Senticos, Ci Wu Jia)
73	6443665	Safflomin A	-7.276	-7.937	Green Dot (High in 6VSB)	HH (Carthamus tinctorius L., Hong Hua)
74	134715163	3-Feruloyl-1-Sinapoyl sucrose	-7.59	-7.935	Green Dot (High in 6VSB)	YZ (Polygala tenuifolia Willd, Yuan Zhi)
75	10374155	Polygalasaponin V	-6.095	-7.927	Green Dot (High in 6VSB)	GZJ (Lemmaphyllum microphyllum, Gua Zi Jin)
76	91440	Sennoside B	-7.65	-7.904	Green Dot (High in 6VSB)	FXY (Sennae Folium, Fan Xie Ye)
77	138108061	Macranthoside B	-6.162	-7.898	Green Dot (High in 6VSB)	JYH (Lonicerae Japonicae Flos, Jin Yin Hua)
78	441905	Astragaloside III	-4.967	-7.892	Green Dot (High in 6VSB)	HQi (Hedysarum Multijugum Maxim., Huang Qi)
79	21598300	Saikosaponin F	-5.863	-7.892	Green Dot (High in 6VSB)	CH (Radix Bupleuri, Chai Hu)
80	108174	Carboxyatractyloside Potassium Salt	-5.647	-7.882	Green Dot (High in 6VSB)	CEZ (Fructus Xanthii, Cang Er Zi)
81	51346137	Saikosaponin D	-6.597	-7.862	Green Dot (High in 6VSB)	CH (Radix Bupleuri, Chai Hu)
82	12855889	Ginsenoside Rc	-5.798	-7.848	Green Dot (High in 6VSB)	RS (Radix Ginseng, Ren Shen)
83	6451798	Trilobatin	-5.828	-7.839	Green Dot (High in 6VSB)	DSK (Lithocarpus polystachyus Rehd, Duo Sui Ke)
84	123339619	5-MethoxyPinocembroside	-9.359	-10.791	Red Dot (High in Both)	GHC (*Penthorum chinense* Pursh, Gan Huang Cao)
85	65238	1,2,3,4,6-Pentagalloylglucose	-8.927	-8.889	Red Dot (High in Both)	WBZ (*Rhus chinensis* Mill., Wu Bei Zi)
86	11664897	Kaempferol 3-O-β-D-(6"-p-	-11.316	-8.732	Red Dot (High in Both)	YX (Folium Ginkgo, Yin Xing)

		coumaroyl) glucopyranosyl (1-2)-α-L-rhamnopyranoside				
87	392622	Ritonavir	-8.089	-7.828	Orange Dot (The Best Control within 6VSB)	/
88	121304016	Remdesivir	-8.738	-6.754	Orange Dot (The Best Control within 6LU7)	/
89	92727	Lopinavir	-7.794	-7.037	Orange Dot (Control)	/
90	131411	Arbidol	-6.487	-3.934	Orange Dot (Control)	/
91	37542	Ribavirin	-6.159	-5.977	Orange Dot (Control)	/
92	64927	Chloroquine Phosphate	-5.936	-4.634	Orange Dot (Control)	/
93	492405	Favipiravir（T705）	-5.313	-5.489	Orange Dot (Control)	/

We then extended to all 2042 natural compounds. Higher efficiency with 6LU7 was shown in NO.1 to NO.22, while 6VSB was NO.23 to NO.83 and the both was NO.84 to NO.86. The last seven listed were the same control. Source Herbs were named similar as Table 5, except for the Black Tea, using English. Fig 4I showed these data.

Table S10. Extended potential ingredients with source herbs based on the newly docking results.

NO.	Components	Docking Gscore (kcal/mol) 6LU7#	Docking Gscore (kcal/mol) 6VSB#	Source Herbs
1	K3（Kaempferol 3-O-β-D-(6''-p- coumaroyl)	-11.316	-8.732	YX (Folium Ginkgo, 银杏, Yin Xing)

	glucopyranosyl (1-2)-α-L-rhamnopyranoside）			
2	Q3（Quercetin3-O-β-D-(6"-p-coumaroyl) glucopyranosyl (1-2)-α-L-rhamnopyranoside）	-9.782	-7.506	YX (Folium Ginkgo, 银杏, Yin Xing)
3	Jionoside A1	-8.727	-8.029	SDH (Radix Rehmanniae Recens, 生地黄, Shen Di Huang)
4	Jionoside B1	-8.951	-7.169	SDH (Radix Rehmanniae Recens, 生地黄, Shen Di Huang)
5	Cistanoside A	-8.044	-9.636	RCR (Cistanches Herba, 肉苁蓉, Rou Cong Rong)
6	Tubuloside A	-9.104	-3.753	RCR (Cistanches Herba, 肉苁蓉, Rou Cong Rong)
7	Theaflavine-3,3'-digallate	-7.814	-8.329	Black Tea
8	Theaflavin-3-gallate	-9.149	-7.468	Black Tea
9	Theaflavin-3'-gallate	-10.328	-7.228	Black Tea
10	Ginsenoside Ra1	-6.627	-8.316	RS (Radix Ginseng, 人参, Ren Shen)
11	vina-ginsenoside R4	-4.752	-8.147	RS (Radix Ginseng, 人参, Ren Shen)
12	Ginsenoside Rc	-5.798	-7.848	RS (Radix Ginseng, 人参, Ren Shen)
13	Panasenoside	-8.786	-5.444	RS (Radix Ginseng, 人参, Ren Shen)
14	Polygalacin D	-7.323	-8.404	YZ (Polygalae Radix, 远志, Yuan Zhi)
15	3-Feruloyl-1-Sinapoyl sucrose	-7.59	-7.935	YZ (Polygalae Radix, 远志, Yuan Zhi)
16	Tenuifoliside A	-8.871	-6.843	YZ (Polygalae Radix, 远志, Yuan Zhi)
17	Naringin	-6.891	-8.371	ZS (Fructus Aurantii Immaturus, 枳实, Zhi Shi)
18	Poncirin	-6.276	-8.1	ZS (Fructus Aurantii Immaturus, 枳实, Zhi Shi)
19	Naringenin	-8.764	-4.79	ZS (Fructus Aurantii Immaturus, 枳实,

				Zhi Shi)
20	Polyphyllin G	-7.251	-8.12	CL (Rhizoma Paridis, 重楼, Chong Lou)
21	Polyphyllin VII	-6.546	-8.055	CL (Rhizoma Paridis, 重楼, Chong Lou)
22	Polyphyllin D	-7.078	-8.049	CL (Rhizoma Paridis, 重楼, Chong Lou)

This table showed the data in details, which was corresponded to Fig 4J. The most potential ingredients were selected after contrasted to controls, and traced back to corresponded herbs in turn. Herbs were presented by Acronyms (Latin name, Chinese name, Chinese Pin Yin), e.g., YX (Folium Ginkgo, 银杏, Yin Xing), etc. except for Black Tea using English name directly.

Table S11. The all docking lists of 2,042 extended natural compounds.

NO.	CID	Components Name	Docking gscore (kcal/mol) 6LU7#	Docking gscore (kcal/mol) 6VSB#	Label
1	492405	Favipiravir（T705）	-5.313	-5.489	Control
2	121304016	Remdesivir	-8.738	-6.754	The Best Control within 6LU7
3	392622	Ritonavir	-8.089	-7.828	The Best Control within 6VSB
4	92727	Lopinavir	-7.794	-7.037	Control
5	131411	Arbidol	-6.487	-3.934	Control

6	37542	Ribavirin	-6.159	-5.977	Control
7	64927	Chloroquine Phosphate	-5.936	-4.634	Control
8	9987332	Sanggenone D	-9.14	-7.509	High in 6LU7
9	124222341	Tectorigenin 7-O-xylosylglucoside	-8.798	-8.089	High in 6LU7
10	10169367	Quercetin 3-O-β-D-(6"-p-coumaroyl)glucopyranosyl(1-2)-α-L-rhamnopyranoside	-9.782	-7.506	High in 6LU7
11	137119925	Theaflavin-3-gallate	-9.149	-7.468	High in 6LU7
12	137796485	Quercetin 3-O-β-D-glucose-7-O-β-D-gentiobioside	-9.674	-7.433	High in 6LU7
13	92043273	Epimedin A1	-9.633	-7.382	High in 6LU7
14	45356919	Madecassoside	-8.917	-7.346	High in 6LU7
15	71307578	Theaflavin-3'-gallate	-10.328	-7.228	High in 6LU7
16	5281782	Jionoside B1	-8.951	-7.169	High in 6LU7
17	10101498	Ligupurpuroside B	-9.921	-7.09	High in 6LU7
18	10346914	Kukoamine B	-8.925	-6.916	High in 6LU7
19	46933844	Tenuifoliside A	-8.871	-6.843	High in 6LU7
20	23958169	Isoforsythiaside	-9.92	-6.763	High in 6LU7
21	9960512	Kaempferol 3-O-gentiobioside	-8.754	-6.469	High in 6LU7
22	5280805	Rutin	-9.225	-6.377	High in 6LU7

23	5318767	Kaempferol-3-Rutinoside	-8.966	-5.972	High in 6LU7
24	5320863	Quercetin 3-O-β-D-xylopyranoside	-8.934	-5.671	High in 6LU7
25	9986191	Panasenoside	-8.786	-5.444	High in 6LU7
26	72936	Kushenol F	-9.202	-5.218	High in 6LU7
27	193679	Isobavachin	-8.895	-4.843	High in 6LU7
28	439246	Naringenin	-8.764	-4.79	High in 6LU7
29	21637830	Tubuloside A	-9.104	-3.753	High in 6LU7
30	118718881	Acetylarenobufagin	-6.616	-10.954	High in 6VSB
31	137796326	Ligupurpuroside C	-6.615	-10.116	High in 6VSB
32	51346147	Pseudoprotodioscin	-4.983	-9.713	High in 6VSB
33	71307448	Cistanoside A	-8.044	-9.636	High in 6VSB
34	441885	Protodeltonin	-6.709	-9.42	High in 6VSB
35	442439	Neohesperidin	-7.682	-9.041	High in 6VSB
36	53462233	Anemarsaponin B	-7.008	-8.863	High in 6VSB
37	75061243	Parishin B	-8.173	-8.758	High in 6VSB
38	14037387	Tubeimoside II	-4.594	-8.745	High in 6VSB
39	5320686	Tiliroside	-7.936	-8.739	High in 6VSB
40	53317652	Platycodin D2	-7.474	-8.665	High in 6VSB
41	441892	Protogracillin	-7.41	-8.642	High in 6VSB
42	51346169	Jujuboside A	-5.393	-8.64	High in 6VSB
43	71571492	Clinodiside A	-6.827	-8.61	High in 6VSB
44	5281693	Robinin	-8.562	-8.604	High in 6VSB
45	14189963	Rubrofusarin gentiobiosid	-8.013	-8.593	High in 6VSB

46	5274591	Isoliensinine	-7.855	-8.577	High in 6VSB
47	6442411	Poliumoside	-8.592	-8.556	High in 6VSB
48	11491905	Hederacoside C	-6.554	-8.55	High in 6VSB
49	71307558	Anemoside B4	-7.364	-8.515	High in 6VSB
50	46887681	Gypenoside IX	-6.174	-8.515	High in 6VSB
51	101568804	Grosvenorine	-6.198	-8.489	High in 6VSB
52	69964214	Neodiosmin	-7.313	-8.482	High in 6VSB
53	169853	Procyanidin C1	-6.794	-8.475	High in 6VSB
54	75412556	Notoginsenoside Fc	-7.111	-8.407	High in 6VSB
55	2724385	Digoxin	-6.316	-8.406	High in 6VSB
56	127256227	Polygalacin D	-7.323	-8.404	High in 6VSB
57	442428	Naringin	-6.891	-8.371	High in 6VSB
58	135403795	Theaflavine-3,3'-digallat e	-7.814	-8.329	High in 6VSB
59	100941542	Ginsenoside Ra1	-6.627	-8.316	High in 6VSB
60	101389834	Anemarsaponin C	-6.355	-8.266	High in 6VSB
61	133561676	Ophiopogonin C	-5.611	-8.262	High in 6VSB
62	441891	Protodioscin	-6.369	-8.257	High in 6VSB
63	71307562	Congmunoside VII	-6.13	-8.238	High in 6VSB
64	134687322	HuangjiangSu A	-5.637	-8.216	High in 6VSB
65	24177534	2,3,4,5-Tetracaffeoyl-D-Glucaric acid	-6.82	-8.153	High in 6VSB
66	15940177	vina-ginsenoside R4	-4.752	-8.147	High in 6VSB
67	14681435	Arctigenin 4'-O-β-gen	-8.578	-8.135	High in 6VSB
68	71307572	Polyphyllin G	-7.251	-8.12	High in 6VSB
69	442456	Poncirin	-6.276	-8.1	High in 6VSB

70	10557926	Parishin A	-6.667	-8.09	High in 6VSB
71	44575945	Timosaponin B II	-7.287	-8.075	High in 6VSB
72	11993928	Acanthopanaxoside B	-6.22	-8.07	High in 6VSB
73	176233	Polyphyllin VII	-6.546	-8.055	High in 6VSB
74	46200821	Polyphyllin D	-7.078	-8.049	High in 6VSB
75	44575944	Anemarsaponin BIII	-5.294	-8.039	High in 6VSB
76	6325450	Jionoside A1	-8.727	-8.029	High in 6VSB
77	11828754	Alcesefoliside	-8.139	-7.976	High in 6VSB
78	14564503	Macranthoidin A	-7.597	-7.959	High in 6VSB
79	71307447	Ciwujianoside B	-6.41	-7.953	High in 6VSB
80	6443665	Safflomin A	-7.276	-7.937	High in 6VSB
81	134715163	3-Feruloyl-1-Sinapoyl sucrose	-7.59	-7.935	High in 6VSB
82	10374155	Polygalasaponin V	-6.095	-7.927	High in 6VSB
83	91440	Sennoside B	-7.65	-7.904	High in 6VSB
84	138108061	Macranthoside B	-6.162	-7.898	High in 6VSB
85	441905	Astragaloside III	-4.967	-7.892	High in 6VSB
86	21598300	Saikosaponin F	-5.863	-7.892	High in 6VSB
87	108174	Carboxyatractyloside Potassium Salt	-5.647	-7.882	High in 6VSB
88	51346137	Saikosaponin D	-6.597	-7.862	High in 6VSB
89	12855889	Ginsenoside Rc	-5.798	-7.848	High in 6VSB
90	6451798	Trilobatin	-5.828	-7.839	High in 6VSB
91	123339619	5-MethoxyPinocembroside	-9.359	-10.791	High in Both
92	65238	1,2,3,4,6-Pentagalloylgl	-8.927	-8.889	High in Both

		ucose			
93	11664897	Kaempferol 3-O-β-D-(6''-p-coumaroyl)glucopyranosyl(1-2)-α-L-rhamnopyranoside	-11.316	-8.732	High in Both
94	10033524	Ophiopogonin D'	-5.059	-6.722	Relatively Low
95	122169313	Terrestrosin D	-7.839	-7.819	Relatively Low
96	11968389	3′,6-Disinapoylsucrose.	-6.126	-7.817	Relatively Low
97	21636280	Saikosaponin H	-6.103	-7.817	Relatively Low
98	114627	Neoeriocitrin	-7.509	-7.813	Relatively Low
99	101920412	Esculentoside H	-5.774	-7.811	Relatively Low
100	102004611	Isosakuranin	-6.088	-7.808	Relatively Low
101	51106	Daurisoline	-6.984	-7.803	Relatively Low
102	72284	Chebulinic acid	-6.339	-7.754	Relatively Low
103	4613731	Methyl Hesperidin	-7.479	-7.753	Relatively Low
104	16760075	Didymin	-6.613	-7.738	Relatively Low
105	90657714	Notoginsenoside Fe	-6.409	-7.737	Relatively Low
106	70698266	Desapioplatycodin D	-4.967	-7.728	Relatively Low
107	125181875	Hosenkoside F	-6.024	-7.714	Relatively Low
108	72814155	Dimethyl lithospermate B	-8.541	-7.713	Relatively Low
109	11629473	Polygalasaponin F	-5.851	-7.712	Relatively Low
110	5280746	Apiin	-6.704	-7.702	Relatively Low
111	5748394	Epimedin C	-7.843	-7.7	Relatively Low

112	51666248	Neoliquiritin	-6.649	-7.692	Relatively Low
113	30231	Neohesperidin Dihydrochalcone	-6.698	-7.69	Relatively Low
114	25087702	Baohuoside V	-8.42	-7.685	Relatively Low
115	5317025	Acaciin	-6.556	-7.685	Relatively Low
116	639665	Xanthohumol	-7.298	-7.685	Relatively Low
117	162859	Platycodin D	-6.528	-7.682	Relatively Low
118	91973814	Notoginsenoside Ft1	-5.849	-7.678	Relatively Low
119	6476333	Isoacteoside	-6.331	-7.674	Relatively Low
120	160644	Liensinine	-7.262	-7.672	Relatively Low
121	71307566	Liensinine Diperchlorate	-7.9	-7.672	Relatively Low
122	14284436	Asperosaponin VI	-5.92	-7.671	Relatively Low
123	102004930	Hosenkoside G	-8.236	-7.662	Relatively Low
124	44630346	Xyloglucan heptasaccharide	-8.497	-7.659	Relatively Low
125	10396409	Hosenkoside M	-6.24	-7.646	Relatively Low
126	10328746	Ardisiacrispin A	-6.726	-7.645	Relatively Low
127	51346142	20(R)-Ginsenoside Rg3	-6.315	-7.623	Relatively Low
128	74977425	Chrysin 7-O-β-gentiobioside	-5.972	-7.617	Relatively Low
129	5486199	Kaempferitrin	-6.298	-7.61	Relatively Low
130	5281667	kuwanon G	-6.958	-7.605	Relatively Low
131	24721205	Asiaticoside	-6.896	-7.602	Relatively Low
132	6072	Phloridzin	-7.347	-7.602	Relatively Low

133	102482481	Officinalisinin I	-7.952	-7.596	Relatively Low
134	138112388	Proanthocyanidin A4	-8.078	-7.588	Relatively Low
135	101838198	Notoginsenoside FP2	-6.63	-7.583	Relatively Low
136	71307556	Anemarsaponin E	-6.242	-7.583	Relatively Low
137	5748393	Epimedin B	-5.59	-7.578	Relatively Low
138	102594495	Mogroside II A2	-6.954	-7.55	Relatively Low
139	441923	Ginsenoside Rg1	-6.537	-7.542	Relatively Low
140	441934	Notoginsenoside R1	-6.442	-7.54	Relatively Low
141	122169314	Terrestrosin K	-5.661	-7.529	Relatively Low
142	76959646	Rhodiosin	-6.992	-7.528	Relatively Low
143	5489425	Tubeimoside III	-6.141	-7.528	Relatively Low
144	3082025	Dryocrassin	-8.237	-7.528	Relatively Low
145	46783811	Saikosaponin C	-6.637	-7.527	Relatively Low
146	5486699	Troxerutin	-7.908	-7.527	Relatively Low
147	5282160	Quercetin-7-O-beta-D-glucopyranoside	-6.12	-7.52	Relatively Low
148	185958	Vicenin III	-7.334	-7.519	Relatively Low
149	21597353	Spinosin B	-6.987	-7.514	Relatively Low
150	5281800	Acteoside	-6.964	-7.513	Relatively Low
151	131849056	Gypenoside A	-6.784	-7.5	Relatively Low
152	131636627	1,3,5-Tricaffeoylquinic acid	-8.26	-7.492	Relatively Low
153	11263254	Methyl protodioscin	-6.383	-7.491	Relatively Low
154	5281773	Forsythoside A	-7.575	-7.491	Relatively Low
155	91758420	Buddlejasaponin IVb	-7.904	-7.491	Relatively Low
156	5282150	Rhoifolin	-7.198	-7.48	Relatively Low

157	23757181	Angoroside C	-8.195	-7.47	Relatively Low
158	11592917	Clitorin	-7.478	-7.457	Relatively Low
159	71717038	Momordicoside A	-6.599	-7.456	Relatively Low
160	44584733	Punicalagin	-2.666	-7.45	Relatively Low
161	138107979	Rebaudioside F	-7.179	-7.437	Relatively Low
162	46173830	Sennoside D	-7.086	-7.432	Relatively Low
163	21599443	Bacopasaponin C	-7.796	-7.428	Relatively Low
164	21629996	2-Acetylacteoside	-6.67	-7.427	Relatively Low
165	6474310	3,5-Dicaffeoylquinic acid	-6.022	-7.425	Relatively Low
166	159654	Neferine	-6.285	-7.425	Relatively Low
167	101422334	Vitexin 2"-O-p-coumarate	-7.805	-7.423	Relatively Low
168	102004853	9"-Methyl salvianolate B	-8.171	-7.417	Relatively Low
169	503737	Liquiritin	-6.627	-7.41	Relatively Low
170	10417462	ligustroflavone	-6.545	-7.409	Relatively Low
171	11968391	Tenuifoliside C	-6.22	-7.408	Relatively Low
172	167928	Saikosaponin A	-5.866	-7.407	Relatively Low
173	204	Allantoin	-7.54	-7.401	Relatively Low
174	452707	1,3,6-Tri-O-galloyl-beta -D-	-7.175	-7.399	Relatively Low
175	21637637	11(α)-methoxysaikosaponin F	-6.623	-7.394	Relatively Low
176	122738	Procyanidin B2	-6.422	-7.39	Relatively Low
177	73111	Sennoside A	-5.982	-7.386	Relatively Low

178	5281668	Kuwanon H	-7.884	-7.38	Relatively Low
179	21599442	Bacopaside I	-6.46	-7.379	Relatively Low
180	5280441	Vitexin	-6.805	-7.367	Relatively Low
181	5317644	Aloe-emodin-8-O-beta-D-glucopyranoside	-7.975	-7.361	Relatively Low
182	5089683	Corilagin	-5.066	-7.358	Relatively Low
183	72941582	Dulcoside A	-6.519	-7.355	Relatively Low
184	21635749	Picfeltarraenin IV	-4.953	-7.349	Relatively Low
185	101953010	Otophylloside A	-5.44	-7.337	Relatively Low
186	168849	Pectolinarin	-7.536	-7.333	Relatively Low
187	442437	Neoastilbin	-6.704	-7.332	Relatively Low
188	5281233	Crocin I	-3.495	-7.331	Relatively Low
189	11250133	Procyanidin B1	-6.474	-7.322	Relatively Low
190	249332	Vincristine Sulfate	-4.755	-7.313	Relatively Low
191	36314	Paclitaxel	-6.558	-7.301	Relatively Low
192	5978	Vincristine	-5.307	-7.297	Relatively Low
193	16007240	Gypenoside XLIX	-4.77	-7.283	Relatively Low
194	92794	Naringenin-7-O-β-D-glucoside	-6.778	-7.278	Relatively Low
195	5492427	Baohuoside VII	-5.953	-7.277	Relatively Low
196	46173829	Sennoside C	-7.497	-7.265	Relatively Low
197	10079485	Otophylloside B	-5.435	-7.264	Relatively Low
198	124005	Maltopentaose	-7.821	-7.252	Relatively Low
199	6917976	Ginsenoside Rb2	-5.586	-7.25	Relatively Low
200	5320313	Oroxin A	-7.364	-7.25	Relatively Low

201	143802279	8,9-epoxy-3,10-diisobutyryloxythymol	-6.596	-7.248	Relatively Low
202	70694423	6-Methoxykaempferol 3-O-rutinoside	-7.841	-7.246	Relatively Low
203	21637642	Saikosaponin B2	-5.586	-7.242	Relatively Low
204	21627940	Dipsacoside B	-6.948	-7.24	Relatively Low
205	122097	Soyasaponin Bb	-5.189	-7.231	Relatively Low
206	5317093	Epimedoside A	-8.664	-7.224	Relatively Low
207	5322089	Resveratrol 4'-Glucoside	-5.835	-7.222	Relatively Low
208	21633072	Pseudoginsenoside F11	-6.755	-7.205	Relatively Low
209	73400	Dauricine	-6.756	-7.191	Relatively Low
210	442458	Sanggenon C	-7.499	-7.184	Relatively Low
211	101248035	Luteolin 7-apiosyl-(1->2)-glucoside	-7.742	-7.178	Relatively Low
212	10621	Hesperidin	-7.762	-7.174	Relatively Low
213	5281321	Cucurbitacin I	-5.5	-7.168	Relatively Low
214	10077207	Oroxin B	-7.682	-7.168	Relatively Low
215	71307582	Vaccarin	-6.601	-7.155	Relatively Low
216	11593362	3α-Hydroxymogrol	-6.058	-7.15	Relatively Low
217	5281793	Salvianolic acid A	-6.692	-7.14	Relatively Low
218	21637632	Saikosaponin E	-5.375	-7.132	Relatively Low
219	119245	Dioscin	-5.078	-7.129	Relatively Low
220	102120501	Picfeltarraenin IB	-4.919	-7.124	Relatively Low

221	127509	Apiopaeonoside	-6.718	-7.117	Relatively Low
222	10473311	Licochalcone D	-5.93	-7.112	Relatively Low
223	5469424	Demethoxycurcumin	-7.719	-7.112	Relatively Low
224	21626520	Pennogenin 3-O-beta-chacotrioside	-4.499	-7.102	Relatively Low
225	129449684	Forsythoside H	-8.092	-7.099	Relatively Low
226	71522133	17-Hydroxy sprengerinin C	-5.343	-7.097	Relatively Low
227	21581293	sibiricaxanthone B	-7.331	-7.096	Relatively Low
228	17752183	11-Oxomogroside III	-5.956	-7.083	Relatively Low
229	169511	Tracheloside	-6.972	-7.079	Relatively Low
230	11650910	Pulsatilla saponin D	-4.941	-7.073	Relatively Low
231	73296	Alpha-Hederin	-7.079	-7.072	Relatively Low
232	12358846	1,4-Dicaffeoylquinic acid	-6.199	-7.067	Relatively Low
233	135403798	Theaflavin	-7.112	-7.066	Relatively Low
234	137796480	Saikosaponin B4	-5.364	-7.05	Relatively Low
235	5388496	Punicalin	-4.792	-7.049	Relatively Low
236	91808875	Monensin B	-5.079	-7.042	Relatively Low
237	25115190	Camelliaside B	-7.239	-7.042	Relatively Low
238	5281318	Cucurbitacin D	-5.241	-7.035	Relatively Low
239	83489	Eriocitrin	-7.117	-7.027	Relatively Low
240	75412560	Tenacissoside H	-5.121	-7.027	Relatively Low
241	5281255	Isobavachalcone	-7.809	-7.02	Relatively Low
242	6918448	Neomangiferin	-7.537	-7.019	Relatively Low
243	5281645	Lancerin	-6.52	-7.015	Relatively Low

244	9823887	Rosavin	-7.877	-7.011	Relatively Low
245	9918693	Ginsenoside Rg3	-6.133	-7.01	Relatively Low
246	120742	Nepetin-7-glucoside	-6.634	-7.003	Relatively Low
247	21603986	Paris saponin II	-5.116	-7.003	Relatively Low
248	146798	Procyanidin B3	-8.076	-6.993	Relatively Low
249	11953944	Purpureaside C	-8.146	-6.985	Relatively Low
250	5281613	Diosmin	-6.089	-6.981	Relatively Low
251	124034	Swertisin	-5.707	-6.975	Relatively Low
252	6453359	2"-O-Galloylhyperin	-7.498	-6.971	Relatively Low
253	23928102	Forsythoside B	-8.383	-6.97	Relatively Low
254	5318597	Isomangiferin	-6.398	-6.96	Relatively Low
255	159646	Lycobetaine	-7.958	-6.957	Relatively Low
256	100966786	Steviol-19-O-Glucoside	-7.149	-6.954	Relatively Low
257	92044471	Cimiracemoside D	-5.974	-6.945	Relatively Low
258	10146542	Xylopentaose	-7.852	-6.945	Relatively Low
259	24096391	Octopamine, N-feruloyl-	-6.653	-6.939	Relatively Low
260	181183	Hemslecin A	-5.942	-6.919	Relatively Low
261	101604422	Syringaresnol-4-O-beta-D-apiofuranosy	-7.001	-6.917	Relatively Low
262	5282152	Lonicerin	-6.341	-6.916	Relatively Low
263	11330069	Styraxlignolide F	-6.142	-6.91	Relatively Low
264	5281316	Cucurbitacin B	-4.371	-6.908	Relatively Low
265	102004864	Orientin 2"-O-p-trans-coumarate	-8.253	-6.908	Relatively Low
266	442089	Stevioside	-8.207	-6.907	Relatively Low

267	442659	Swertiajaponin	-7.218	-6.896	Relatively Low
268	5273567	Calceolarioside B	-7.868	-6.895	Relatively Low
269	134692398	Pinocembroside	-6.544	-6.895	Relatively Low
270	12960460	Kaempferol 3-sophoroside-7-glucoside	-7.212	-6.894	Relatively Low
271	11629084	Salvianolic acid B	-7.391	-6.893	Relatively Low
272	10883790	14,15beta-Dihydroxyklaineanone	-5.835	-6.887	Relatively Low
273	129896873	Glomeratose A	-5.731	-6.884	Relatively Low
274	21637635	Saikosaponin B3	-5.741	-6.882	Relatively Low
275	101881655	Caesappanin C	-5.074	-6.872	Relatively Low
276	101740054	Polygalaxanthone XI	-7.237	-6.869	Relatively Low
277	102004870	14-hydroxysprengerinin C	-5.027	-6.866	Relatively Low
278	101967019	Sylvestroside I	-7.132	-6.857	Relatively Low
279	442664	Vicenin II	-7.332	-6.857	Relatively Low
280	11016019	Diosmetin-7-O-beta-D-glucopyranoside	-6.047	-6.856	Relatively Low
281	169961	Rhodojaponin V	-4.895	-6.848	Relatively Low
282	14136851	Oleuroside	-6.468	-6.842	Relatively Low
283	9898279	Ginsenoside Rb1	-6.695	-6.833	Relatively Low
284	5487343	Camellianin A	-6.583	-6.829	Relatively Low
285	21630094	Hederacoside D	-6.682	-6.823	Relatively Low
286	12912363	Ginsenoside Rb3	-6.42	-6.805	Relatively Low
287	101826550	Saikosaponin G	-7.152	-6.799	Relatively Low

288	2153	Theophylline	-6.66	-6.797	Relatively Low
289	10317069	Xanthohumol D	-8.254	-6.796	Relatively Low
290	6326021	Sibiricose A6	-6.995	-6.787	Relatively Low
291	10055215	Tenuifoliside B	-7.521	-6.778	Relatively Low
292	6326022	Sibirioside A	-7.105	-6.777	Relatively Low
293	6450879	Bavachalcone	-6.098	-6.777	Relatively Low
294	9875547	Saikosaponin B1	-6.08	-6.767	Relatively Low
295	71307561	Congmunoside V	-6.562	-6.758	Relatively Low
296	5281771	Echinacoside	-5.969	-6.754	Relatively Low
297	176596	Momordin Ic	-6.717	-6.74	Relatively Low
298	46173859	Ophiopogonin D	-6.098	-6.739	Relatively Low
299	24121289	Picroside III	-7.247	-6.734	Relatively Low
300	28523	Tomatine	-7.574	-6.734	Relatively Low
301	5281718	Polydatin	-5.003	-6.732	Relatively Low
302	5280637	Luteoloside	-6.315	-6.727	Relatively Low
303	45033634	Rhapontigenin 3'-O-glucoside	-6.621	-6.727	Relatively Low
304	107905	Epicatechin gallate	-6.346	-6.727	Relatively Low
305	21723007	Isosilybin	-7.142	-6.716	Relatively Low
306	60208888	Rebaudioside C	-7.678	-6.712	Relatively Low
307	10772	Coixol	-6.627	-6.708	Relatively Low
308	5280960	Naringenin chalcone	-6.829	-6.703	Relatively Low
309	3001497	Geraniin	-5.114	-6.701	Relatively Low
310	91618002	Asiaticoside B	-5.883	-6.699	Relatively Low
311	21599924	Ginsenoside Rg2	-4.491	-6.693	Relatively Low
312	138112434	Procyanidin A1	-8.068	-6.693	Relatively Low

313	21631098	Mudanpioside C	-8.225	-6.686	Relatively Low
314	442431	Narirutin	-8.226	-6.681	Relatively Low
315	5281788	Plantamajoside	-7.872	-6.681	Relatively Low
316	70680623	Alpha-Solamarine	-5.633	-6.68	Relatively Low
317	65064	Epigallocatechin gallate	-7.367	-6.673	Relatively Low
318	5318997	Icariin	-7.251	-6.671	Relatively Low
319	24721031	Jujuboside B	-4.187	-6.662	Relatively Low
320	101672279	Anemarrhenasaponin I	-6.274	-6.644	Relatively Low
321	11622076	Hederacolchiside A1	-7.541	-6.643	Relatively Low
322	134715175	Kudinoside D	-4.853	-6.637	Relatively Low
323	91973815	Soyasaponin Ba	-5.108	-6.63	Relatively Low
324	442731	Chrysophanol-8-O-beta-D-glucopyranoside	-5.77	-6.628	Relatively Low
325	137796330	5,6,7,40-tetrahydroxyisoflavone-6,7-di-O-β-D-glucopyranoside	-5.505	-6.626	Relatively Low
326	60148697	Isoastragaloside I	-5.539	-6.624	Relatively Low
327	9917980	Secoisolariciresinol Diglucoside	-7.736	-6.624	Relatively Low
328	5315244	Gaultherin	-7.13	-6.622	Relatively Low
329	9894584	Naringin dihydrochalcone	-6.776	-6.622	Relatively Low
330	9876264	Bacopaside II	-6.173	-6.618	Relatively Low

331	165901	Higenamine hydrochloride	-7.624	-6.618	Relatively Low
332	114840	Higenamine	-7.624	-6.618	Relatively Low
333	11007422	Saponin C, from Liriope muscari	-4.297	-6.614	Relatively Low
334	442665	Violanthin	-7.278	-6.61	Relatively Low
335	5280704	Apigetrin	-6.341	-6.609	Relatively Low
336	5319484	Apigenin-7-O-glucuronide	-6.736	-6.608	Relatively Low
337	6453452	Engeletin	-5.617	-6.607	Relatively Low
338	101887367	Pseudoginsenoside Rh2	-5.39	-6.606	Relatively Low
339	174742	Raddeanin A	-5.022	-6.606	Relatively Low
340	267250	Dihydrocucurbitacin B	-6.178	-6.603	Relatively Low
341	5496475	5,7,3'-Trihydroxy-6,4',5'-trimethoxyflavone	-6.912	-6.602	Relatively Low
342	10320370	Rosarin	-6.959	-6.601	Relatively Low
343	442658	Schaftoside	-7.67	-6.595	Relatively Low
344	10343508	Rhein-8-glucoside	-6.717	-6.594	Relatively Low
345	148124	Docetaxel	-4.931	-6.594	Relatively Low
346	73253	heraclenol	-6.74	-6.591	Relatively Low
347	5281807	Puerarin	-5.919	-6.587	Relatively Low
348	131844296	Acetyl Perisesaccharide C	-4.113	-6.586	Relatively Low
349	5481645	Triptonine B	-3.569	-6.581	Relatively Low

350	46887678	Ginsenoside F3	-6.442	-6.575	Relatively Low
351	6436208	Cephalomannine	-6.002	-6.573	Relatively Low
352	68247	Cyanidin chloride	-7.093	-6.567	Relatively Low
353	182251	oxyimperatorin	-6.543	-6.558	Relatively Low
354	13878151	Hederoside D2	-6.249	-6.556	Relatively Low
355	5352014	Isocucurbitacin B	-5.218	-6.554	Relatively Low
356	441921	Ginsenoside Re	-6.259	-6.545	Relatively Low
357	13991590	Salvianolic acid C	-6.009	-6.528	Relatively Low
358	21631107	Benzoyloxypaeoniflorin	-7.459	-6.515	Relatively Low
359	442830	(-)-Syringaresinol di-O-glucoside	-6.319	-6.514	Relatively Low
360	10676408	Parishin C	-6.711	-6.511	Relatively Low
361	5319485	3'-MethoxyPuerarin	-5.982	-6.51	Relatively Low
362	5282155	Kaempferol-3-O-sophoroside	-7.047	-6.502	Relatively Low
363	10481797	Cucurbitacin IIb	-5.051	-6.501	Relatively Low
364	102004835	Ginsenoside F4	-5.715	-6.5	Relatively Low
365	197081	Cyanidin-3-glucoside chloride	-7.596	-6.499	Relatively Low
366	71312557	Eleutheroside E	-6.295	-6.493	Relatively Low
367	6443484	Mulberroside A	-6.256	-6.492	Relatively Low
368	21676217	Mirificin	-6.423	-6.491	Relatively Low
369	5488307	Luteolin 7-glucuronide	-6.723	-6.49	Relatively Low
370	45055483	Vinorelbine Tartrate	-7.663	-6.485	Relatively Low
371	5390854	Gelsemine	-5.443	-6.475	Relatively Low

372	5281777	Iridin	-6.682	-6.472	Relatively Low
373	5320644	Podocarpusflavone A	-7.79	-6.465	Relatively Low
374	6758	Rotenone	-4.658	-6.462	Relatively Low
375	2723635	D-Glucosamine hydrochloride	-6.549	-6.458	Relatively Low
376	5321954	Acacetin-7-Glucoside	-5.711	-6.457	Relatively Low
377	441381	Saponarin	-7.845	-6.456	Relatively Low
378	102004868	Ophiogenin 3-O-α-L-rhamnopyranosyl-(1→2)-β-D-glucopyranoside	-6.987	-6.453	Relatively Low
379	3084961	Wogonoside	-6.827	-6.441	Relatively Low
380	6441498	Lithospermic acid	-6.149	-6.44	Relatively Low
381	159861	Gracillin	-6.116	-6.432	Relatively Low
382	5318761	Kaempferol-3-O-glucorhamnoside	-7.364	-6.428	Relatively Low
383	101834999	Carboxyatractyloside	-5.771	-6.426	Relatively Low
384	21599925	Notoginsenoside R2	-6.543	-6.424	Relatively Low
385	667639	Piceatannol	-7.036	-6.423	Relatively Low
386	5490351	Kakkalide	-7.514	-6.422	Relatively Low
387	5281792	Rosmarinic acid	-5.873	-6.416	Relatively Low
388	5318569	Isoginkgetin	-7.706	-6.411	Relatively Low
389	3068143	Emetine Dihydrochloride	-5.352	-6.403	Relatively Low
390	5281654	Isorhamnetin	-6.88	-6.403	Relatively Low
391	161557	Dihydromyricetin	-6.789	-6.399	Relatively Low

392	107957	Catechin hydrate	-6.947	-6.396	Relatively Low
393	90477999	Ophiopojaponin C	-5.361	-6.395	Relatively Low
394	161409	Columbianetin acetate	-6.212	-6.395	Relatively Low
395	114776	Homoorientin	-7.097	-6.395	Relatively Low
396	9854073	Cabazitaxel	-5.435	-6.394	Relatively Low
397	65084	(+)-Gallocatechin	-6.375	-6.393	Relatively Low
398	6603320	Emetine Hydrochloride	-6.5	-6.393	Relatively Low
399	9064	Catechin	-6.945	-6.392	Relatively Low
400	91884878	Picfeltarraenin IA	-4.414	-6.383	Relatively Low
401	25115189	Camelliaside A	-8.514	-6.378	Relatively Low
402	5272653	7,3',4'-Tri-O-methyllute olin	-6.882	-6.376	Relatively Low
403	11240167	Pseudoprotogracillin	-7.505	-6.371	Relatively Low
404	185914	Dihydroresveratrol	-7.571	-6.367	Relatively Low
405	324796	Homoarbutin	-5.828	-6.364	Relatively Low
406	441922	Ginsenoside Rf	-6.389	-6.361	Relatively Low
407	70702337	Glucotropaeolin	-7.217	-6.36	Relatively Low
408	21591681	Rebaudioside G	-7.019	-6.36	Relatively Low
409	5281222	butein	-6.631	-6.358	Relatively Low
410	11550001	Ginsenoside Rg5	-5.431	-6.357	Relatively Low
411	3133561	12-epinapelline	-5.417	-6.344	Relatively Low
412	9849283	Picroside II	-5.975	-6.34	Relatively Low
413	14162695	Scutellarin methyl ester	-6	-6.339	Relatively Low
414	6474640	1,5-Dicaffeoylquinic acid	-6.713	-6.337	Relatively Low

415	157631	Tinnevellin glucoside	-5.805	-6.336	Relatively Low
416	5281670	Morin	-8.236	-6.327	Relatively Low
417	5320954	Rhapontigenin	-6.083	-6.317	Relatively Low
418	162350	Isovitexin	-7.215	-6.317	Relatively Low
419	5318865	Kukoamine A	-8.567	-6.316	Relatively Low
420	5318083	Homoplantaginin	-6.665	-6.314	Relatively Low
421	71306914	Timosaponin A3	-7.195	-6.312	Relatively Low
422	21573759	Khasianine	-4.897	-6.31	Relatively Low
423	10571940	Zeylenone	-7.049	-6.309	Relatively Low
424	5318882	Kurarinone	-7.969	-6.308	Relatively Low
425	51346141	Albiflorin	-5.721	-6.307	Relatively Low
426	44584555	Gypenoside XVII	-6.755	-6.307	Relatively Low
427	5481958	Mulberrin	-8.037	-6.301	Relatively Low
428	241902	Vinblastine sulfate	-4.48	-6.299	Relatively Low
429	119247	Solasonine	-5.173	-6.293	Relatively Low
430	155094	6-Prenylnaringenin	-8.033	-6.292	Relatively Low
431	164648	Androsin	-6.378	-6.29	Relatively Low
432	14778357	Rhodiolin	-6.13	-6.29	Relatively Low
433	10794070	Gambogenic acid	-5.871	-6.287	Relatively Low
434	71307082	Typhaneoside	-7.847	-6.283	Relatively Low
435	5280863	Kaempferol	-8.455	-6.283	Relatively Low
436	445154	Resveratrol	-7.336	-6.28	Relatively Low
437	10079497	Araloside A	-5.126	-6.278	Relatively Low
438	5320227	Notopterol	-6.798	-6.278	Relatively Low
439	5384799	Ochnaflavone 4'-methyl ether	-7.485	-6.273	Relatively Low

440	92044472	Rehmannioside D	-7.289	-6.265	Relatively Low
441	637213	Rhapontin	-5.97	-6.265	Relatively Low
442	5280633	5-Caffeoylquinic acid	-6.584	-6.264	Relatively Low
443	51346122	Astragaloside I	-5.127	-6.262	Relatively Low
444	11228694	Cornuside	-6.858	-6.261	Relatively Low
445	53486204	Lobetyolin	-7.056	-6.255	Relatively Low
446	114829	Liquiritigenin	-8.717	-6.254	Relatively Low
447	5748205	3'-hydroxyPuerarin	-5.814	-6.253	Relatively Low
448	68079	Isopimpinellin	-5.643	-6.251	Relatively Low
449	5281712	Astringin	-6.354	-6.25	Relatively Low
450	5282151	2''-Rhamnosylvitexin	-6.525	-6.244	Relatively Low
451	132993930	Iristectorin A	-6.177	-6.239	Relatively Low
452	5464078	Gamma-mangostin	-6.169	-6.239	Relatively Low
453	9798666	Cryptochlorogenic acid	-6.044	-6.238	Relatively Low
454	160544	Oxypeucedanin	-6.132	-6.236	Relatively Low
455	155692	Spinosin	-6.658	-6.231	Relatively Low
456	158845	Curculigoside	-6.057	-6.231	Relatively Low
457	135398635	Guanosine	-6.967	-6.224	Relatively Low
458	486614	Pinoresinol 4-O-glucoside	-6.639	-6.223	Relatively Low
459	17536	Oxypeucedanin hydrate	-6.857	-6.221	Relatively Low
460	4825	Pimpinellin	-5.677	-6.219	Relatively Low
461	5280343	Quercetin	-6.137	-6.219	Relatively Low
462	12896796	Helicide	-6.504	-6.217	Relatively Low
463	5284452	Quercetin Dihydrate	-6.16	-6.217	Relatively Low

464	72956	Yadanzioside A	-6.236	-6.213	Relatively Low
465	313325	Aloin(mixture of A&B)	-5.858	-6.212	Relatively Low
466	14982	Glycyrrhizic acid	-5.107	-6.212	Relatively Low
467	62074	Monoammonium glycyrrhizinate	-5.107	-6.212	Relatively Low
468	656852	Dipotassium Glycyrrhizinate	-5.107	-6.212	Relatively Low
469	9915886	Thiocolchicoside	-5.886	-6.21	Relatively Low
470	439538	Xylobiose	-6.674	-6.207	Relatively Low
471	91873341	Xylotriose	-6.664	-6.206	Relatively Low
472	6452133	Glucovanillin	-5.897	-6.206	Relatively Low
473	5281319	Cucurbitacin E	-5.423	-6.205	Relatively Low
474	69634125	Forsythoside E	-6.91	-6.205	Relatively Low
475	5282166	Baimaside	-8.025	-6.205	Relatively Low
476	11499198	Ginsenoside RK1	-5.885	-6.204	Relatively Low
477	53461957	Isomucronulatol 7-O-glucoside	-6.72	-6.204	Relatively Low
478	5281769	1,3-Dicaffeoylquinic acid	-5.603	-6.204	Relatively Low
479	102185205	3-O-β-D-Glucopyranosylplatycodigenin	-5.186	-6.201	Relatively Low
480	3083909	Chimonanthine	-5.339	-6.198	Relatively Low
481	5492406	Complanatuside	-7.836	-6.192	Relatively Low
482	5315459	Bilobetin	-7.451	-6.189	Relatively Low

483	137796406	Cimifugin 4'-O-β-D-glucopyranoside	-6.115	-6.185	Relatively Low
484	14355298	Ormosin VI	-6.538	-6.181	Relatively Low
485	5282102	Astragalin	-7.954	-6.181	Relatively Low
486	5748601	Quercetin 7-rhamnoside	-6.075	-6.178	Relatively Low
487	29435	1-Deoxynojirimycin	-6.532	-6.176	Relatively Low
488	969516	Curcumin	-8.044	-6.173	Relatively Low
489	5495926	Garcinone D	-5.542	-6.172	Relatively Low
490	71905	Olaquindox	-6.518	-6.169	Relatively Low
491	24721355	Taxifolin 7-rhamnoside	-6.65	-6.169	Relatively Low
492	21633075	Pseudoginsenoside RT5	-5.426	-6.165	Relatively Low
493	134715181	6'-O-Cinnamoyl harpagide	-7.279	-6.155	Relatively Low
494	24721561	Ginsenoside Rd	-8.019	-6.154	Relatively Low
495	74315890	Uvarigranol C	-6.705	-6.154	Relatively Low
496	442813	Ononin	-5.665	-6.152	Relatively Low
497	92201	columbianetin	-6.871	-6.151	Relatively Low
498	164722	Hamaudol	-6.245	-6.148	Relatively Low
499	13347321	(5R)-trans-1,7-diphenyl-5-hydroxy-6-hepten-3-one	-6.621	-6.142	Relatively Low
500	12305761	Aloin A	-7.09	-6.141	Relatively Low
501	12575	isovanillic acid	-5.697	-6.14	Relatively Low
502	21630160	Liriopesides B	-5.755	-6.134	Relatively Low

503	458010	heraclenin	-6.258	-6.131	Relatively Low
504	5481663	Narcissoside	-7.743	-6.129	Relatively Low
505	11068834	Octahydrocurcumin	-7.463	-6.128	Relatively Low
506	102393334	Isoastragaloside IV	-4.371	-6.127	Relatively Low
507	6325127	Ajugol	-6.167	-6.125	Relatively Low
508	130796	Picrocrocin	-5.454	-6.125	Relatively Low
509	144883010	Isocoptisine acetate	-4.369	-6.125	Relatively Low
510	44584241	Hamamelitannin	-5.945	-6.122	Relatively Low
511	11169063	Polygalaxanthone III	-7.16	-6.121	Relatively Low
512	135413566	Vicine	-6.741	-6.119	Relatively Low
513	25056407	Corylifol A	-6.167	-6.116	Relatively Low
514	6326020	Sibiricose A5	-6.876	-6.115	Relatively Low
515	5461026	Manninotriose	-7.614	-6.11	Relatively Low
516	100528	Arctiin	-5.948	-6.109	Relatively Low
517	5281627	Hinokiflavone	-7.621	-6.106	Relatively Low
518	78384985	Glucosylgentiopicroside	-6.629	-6.102	Relatively Low
519	10228095	6"-O-Acetylglycitin	-7.095	-6.101	Relatively Low
520	10478277	Sec-O-glucosylhamaudol	-6.344	-6.1	Relatively Low
521	14987	Lysergol	-5.809	-6.1	Relatively Low
522	114778	Kahweol	-5.062	-6.095	Relatively Low
523	101601989	Xylotetrose	-6.908	-6.091	Relatively Low
524	166775	Nystose	-6.846	-6.088	Relatively Low
525	5281426	7-Hydroxycoumarin	-5.596	-6.088	Relatively Low
526	21626477	Rhodionin	-5.543	-6.086	Relatively Low
527	133561686	Uvarigranol B	-5.574	-6.08	Relatively Low

528	133561674	Nuezhenidic acid	-5.333	-6.08	Relatively Low
529	5281696	Sciadopitysin	-7.585	-6.079	Relatively Low
530	5281674	Norwogonin	-7.095	-6.078	Relatively Low
531	46173858	Lirioprolioside B	-6.332	-6.077	Relatively Low
532	124025	Procyanidin A2	-6.119	-6.076	Relatively Low
533	7121	Veratric acid	-5.759	-6.074	Relatively Low
534	75231	4-Methoxysalicylic acid	-5.333	-6.072	Relatively Low
535	115149	Amarogentin	-7.659	-6.072	Relatively Low
536	122213508	Ilexsaponin B1	-5.307	-6.067	Relatively Low
537	5281239	Fucoxanthin	-4.606	-6.066	Relatively Low
538	71307080	Picroside I	-5.033	-6.06	Relatively Low
539	102041439	Periplogenin 3-[O-β-glucopyranosyl-(1→4)-β-sarmentopyranoside]	-4.921	-6.057	Relatively Low
540	6438919	Salviaflaside	-5.736	-6.057	Relatively Low
541	441764	Silychristin	-5.84	-6.055	Relatively Low
542	6442433	Isoliquiritin apioside	-6.099	-6.055	Relatively Low
543	101553595	Ginkgolide K	-4.055	-6.053	Relatively Low
544	21576180	Apigenin 6-C-α-L-arabinopyranosyl-8-C-β-D-xylopyranoside	-7.31	-6.052	Relatively Low
545	439514	Scopolin	-6.074	-6.051	Relatively Low
546	5318987	2"-O-rhamnosyl	-6.922	-6.047	Relatively Low

		icariside II			
547	5281614	Fisetin	-6.401	-6.045	Relatively Low
548	12889143	(18β,20α)-Glycyrrhizic acid	-5.086	-6.044	Relatively Low
549	5281810	Tectoridin	-7.256	-6.039	Relatively Low
550	199472	Gallocatechin gallate	-8.313	-6.039	Relatively Low
551	5494868	Sotetsuflavone	-7.317	-6.036	Relatively Low
552	6324923	Chrysophanol 1-glucoside	-7.168	-6.03	Relatively Low
553	5320826	Quercetagitrin	-6.418	-6.03	Relatively Low
554	16088242	CiMigenol 3-beta-D-xylopyranoside	-4.422	-6.028	Relatively Low
555	5318267	calycosin-7-O-beta-D-glucoside	-5.929	-6.027	Relatively Low
556	122850	Dihydrokaempferol	-8.299	-6.026	Relatively Low
557	101422758	Isoviolanthin	-7.869	-6.026	Relatively Low
558	11349817	Licoflavone B	-6.29	-6.021	Relatively Low
559	99649	Emodin-8-glucoside	-7.388	-6.021	Relatively Low
560	73384	Brazilin	-6.886	-6.018	Relatively Low
561	10212	Imperatorin	-6.367	-6.016	Relatively Low
562	5318517	Andrographolide	-4.742	-6.011	Relatively Low
563	44257354	Iristectorin B	-6.405	-6.008	Relatively Low
564	10994544	Fabiatrin	-7.089	-6.007	Relatively Low
565	9918692	Ginsenoside F2	-4.997	-6.007	Relatively Low
566	439533	Taxifolin	-7.306	-6.007	Relatively Low
567	821347	Dehydronuciferin	-6.036	-6.005	Relatively Low

568	21631106	Benzoylpaeoniflorin	-7.464	-6.002	Relatively Low
569	24832659	Benzoylmesaconine	-4.68	-6.002	Relatively Low
570	10146	Nuciferine	-6.747	-6.002	Relatively Low
571	5317764	Glycyrrhisoflavone	-6.695	-6.001	Relatively Low
572	6441913	Coniferyl ferulate	-7.533	-6.001	Relatively Low
573	5281717	Oxyresveratrol	-8.267	-5.997	Relatively Low
574	64982	Baicalin	-6.273	-5.997	Relatively Low
575	5281601	7,4′ -Di-O-methylapigenin	-6.256	-5.996	Relatively Low
576	24721571	Phillyrin	-6.192	-5.991	Relatively Low
577	5320834	Quercetin 3-gentiobioside	-7.69	-5.988	Relatively Low
578	5280544	Herbacetin	-7.497	-5.982	Relatively Low
579	92043450	Hastatoside	-5.417	-5.981	Relatively Low
580	54580480	20(R)-Ginsenoside Rh2	-4.727	-5.981	Relatively Low
581	44566720	Leucoside	-7.946	-5.98	Relatively Low
582	31553	Silybin(Mixture A&B)	-6.941	-5.979	Relatively Low
583	5281680	Quercetagetin	-6.395	-5.979	Relatively Low
584	102004659	Cratoxylone	-6.702	-5.978	Relatively Low
585	5281612	Diosmetin	-5.96	-5.976	Relatively Low
586	65085	Crotonoside	-7.325	-5.973	Relatively Low
587	100990912	Puerarin 6"-O-xyloside	-8.558	-5.972	Relatively Low
588	97214	Eupatorin	-6.552	-5.971	Relatively Low
589	9852185	Gambogic acid	-4.306	-5.971	Relatively Low

590	15596617	4'-O-Glucosylvitexin	-7.871	-5.971	Relatively Low
591	5280537	N-trans-Feruloyltyramine	-8.046	-5.971	Relatively Low
592	73067	Medicarpin	-6.839	-5.97	Relatively Low
593	129539794	5-Ethoxy-10-Gingerol	-4.611	-5.968	Relatively Low
594	480764	8-Prenylnaringenin	-8.601	-5.967	Relatively Low
595	73466	Monotropein	-4.418	-5.966	Relatively Low
596	5281544	Oleuropein	-4.239	-5.961	Relatively Low
597	5280457	Pinosylvin	-6.86	-5.961	Relatively Low
598	10253785	Luteolin-3'-D-glucuronide	-8.107	-5.96	Relatively Low
599	5316673	Kaempferin	-7.254	-5.959	Relatively Low
600	23874492	Octopamine, N-p-coumaroyl-	-6.802	-5.958	Relatively Low
601	164943	Lycorine hydrochloride	-7.071	-5.957	Relatively Low
602	448779	(R)-α-Methyltryptamine	-6.744	-5.952	Relatively Low
603	5317471	Luteolin-5-O-glucoside	-7.637	-5.949	Relatively Low
604	165549	Sophoridine	-6.64	-5.948	Relatively Low
605	5281764	Chicoric acid	-4.553	-5.942	Relatively Low
606	56662029	taccalonolide B	-3.908	-5.941	Relatively Low
607	5320053	Neobavaisoflavone	-6.327	-5.939	Relatively Low
608	65752	Rutaecarpine	-6.351	-5.938	Relatively Low
609	10865594	Bacopaside V	-5.855	-5.937	Relatively Low
610	71463728	Bruceine D	-5.222	-5.936	Relatively Low
611	11968737	Melittoside	-6.03	-5.932	Relatively Low

612	162305	Aloenin A	-6.425	-5.927	Relatively Low
613	581676	Tetrahydropiperine	-5.788	-5.927	Relatively Low
614	10429233	Dihydrocurcumin	-7.874	-5.926	Relatively Low
615	10795088	Apigenin-7-O -(2G-rhamnosyl)gentiobioside	-7.455	-5.914	Relatively Low
616	10095180	Kaempferol-7-O-beta-D-glucopyranoside	-5.96	-5.911	Relatively Low
617	11146840	Specnuezhenide	-7.823	-5.911	Relatively Low
618	101916326	7-O-Methylmangiferin	-6.929	-5.91	Relatively Low
619	8468	Vanillic acid	-5.377	-5.906	Relatively Low
620	25659	Nitidine Chloride	-6.15	-5.902	Relatively Low
621	6438568	Neogambogic acid	-5.4	-5.899	Relatively Low
622	143905847	Mogroside IIa	-6.222	-5.899	Relatively Low
623	108052	Cafestol	-4.462	-5.898	Relatively Low
624	5273755	Eupatilin	-7.297	-5.896	Relatively Low
625	638285	Isopropylidenylacetyl-marmesin	-6.419	-5.894	Relatively Low
626	6419835	Catechin gallate	-6.801	-5.892	Relatively Low
627	114850	Oxymatrine	-5.405	-5.891	Relatively Low
628	442021	Brucine	-5.228	-5.889	Relatively Low
629	442416	Agnuside	-6.711	-5.889	Relatively Low
630	14989	Aloin B	-7.085	-5.887	Relatively Low
631	442923	Paeonolide	-7.684	-5.885	Relatively Low
632	12299879	Atractyloside potassium salt	-5.068	-5.885	Relatively Low

633	442433	Feretoside	-5.395	-5.882	Relatively Low
634	11876135	Dihydrolycorine	-7.481	-5.881	Relatively Low
635	452967	Steviol	-5.047	-5.881	Relatively Low
636	5281673	Myricetrin	-6.285	-5.881	Relatively Low
637	5281377	Genistin	-6.656	-5.88	Relatively Low
638	23615629	Cephaelin Hydrochloride	-7.343	-5.875	Relatively Low
639	442195	Cephaeline	-7.341	-5.875	Relatively Low
640	21670038	5-O-methylvisammioside	-6.093	-5.868	Relatively Low
641	90479257	Edpetiline	-5.817	-5.868	Relatively Low
642	5770	Reserpine	-4.079	-5.868	Relatively Low
643	5317284	Eupafolin	-6.417	-5.868	Relatively Low
644	6436246	Columbianadin	-6.151	-5.864	Relatively Low
645	102022989	Heterophyllin B	-4.269	-5.863	Relatively Low
646	5320521	Raspberry ketone glucoside	-6.666	-5.859	Relatively Low
647	71307571	Polyphyllin VI	-7.959	-5.855	Relatively Low
648	69502	Alloimperatorin	-6.151	-5.855	Relatively Low
649	5281542	Harpagoside	-5.17	-5.853	Relatively Low
650	6915739	Linderane	-4.716	-5.851	Relatively Low
651	73467	Verbenalin	-5.588	-5.851	Relatively Low
652	441071	Strychnine	-6.411	-5.851	Relatively Low
653	10314695	Rosiridin	-6.332	-5.848	Relatively Low
654	441685	Taccalonolide A	-3.398	-5.846	Relatively Low
655	5321398	Sophoricoside	-6.11	-5.844	Relatively Low

656	5281647	Mangiferin	-6.836	-5.844	Relatively Low
657	72369	Tripterifordin	-4.441	-5.843	Relatively Low
658	5280459	Quercitrin	-6.643	-5.842	Relatively Low
659	2198	Anisodamine	-7.121	-5.841	Relatively Low
660	5273569	Fraxetin	-6.217	-5.841	Relatively Low
661	5429	Theobromine	-6.005	-5.84	Relatively Low
662	5280352	Bilirubin	-7.956	-5.839	Relatively Low
663	11721847	Anemoside A3	-6.554	-5.838	Relatively Low
664	14211225	Aloeresin D	-7.653	-5.836	Relatively Low
665	131801361	Ganoderenic acid E	-4.668	-5.835	Relatively Low
666	72277	Epigallocatechin	-6.554	-5.835	Relatively Low
667	5481882	Juglalin	-8.549	-5.834	Relatively Low
668	5352005	Quercetin 3,3',4',7-O-tetramethyl ether	-6.209	-5.831	Relatively Low
669	234441	rel-(8R,8'R)-dimethyl-(7S,7'R)-bis(3,4-methylenedioxyphenyl)tetrahyd ro-furan	-6.715	-5.829	Relatively Low
670	5311497	Vinorelbine	-5.524	-5.829	Relatively Low
671	162876	Erigeroside	-5.918	-5.828	Relatively Low
672	5280372	coniferin	-6.654	-5.828	Relatively Low
673	91458	Aucubin	-6.458	-5.827	Relatively Low
674	6175	Cytidine	-6.003	-5.826	Relatively Low
675	14163819	Fuziline	-4.43	-5.826	Relatively Low
676	6917970	L-Stepholidine	-4.811	-5.826	Relatively Low

677	155831	10-Deacetyltaxol	-7.233	-5.825	Relatively Low
678	3084995	Isoshaftoside	-8.026	-5.825	Relatively Low
679	5281567	Methysticin	-6.925	-5.82	Relatively Low
680	91895489	Ginsenoside Rg6	-5.577	-5.818	Relatively Low
681	9840805	Licochalcone C	-5.72	-5.816	Relatively Low
682	102004842	Kaempferol 3-sophoroside-7-rhamnoside	-7.668	-5.815	Relatively Low
683	72276	Epicatechin	-6.965	-5.811	Relatively Low
684	12302276	Calycanthoside	-6.327	-5.808	Relatively Low
685	70700401	Orcinol glucoside	-6.473	-5.805	Relatively Low
686	5481982	Baohuoside II	-7.07	-5.804	Relatively Low
687	5316606	Desoxyrhaponticin	-5.971	-5.802	Relatively Low
688	131900	Peimine	-4.594	-5.799	Relatively Low
689	119258	Astilbin	-7.031	-5.799	Relatively Low
690	11487078	Tetramethylcurcumin	-6.121	-5.798	Relatively Low
691	5281675	Orientin	-6.739	-5.797	Relatively Low
692	99514	Isosteviol	-4.069	-5.793	Relatively Low
693	6440400	Menisdaurin	-5.445	-5.792	Relatively Low
694	10155076	1-O-Caffeoylquinic acid	-6.511	-5.792	Relatively Low
695	76336194	Alisol A,24-acetate	-3.997	-5.791	Relatively Low
696	13892722	Shanzhiside methylester	-6.437	-5.788	Relatively Low
697	392450	8-Desoxygartanin	-6.974	-5.788	Relatively Low
698	5321884	2,3,5,4'-Tetrahydroxyl-diphenylethylene-2-O-b	-8.44	-5.787	Relatively Low

		eta-D-glucoside			
699	162147	Aloperine	-5.413	-5.785	Relatively Low
700	10383616	Aldehydoisoophiopogonanone A,	-6.71	-5.785	Relatively Low
		6-			
701	115067	Gastrodin	-6.753	-5.784	Relatively Low
702	5280656	Rosin	-6.656	-5.778	Relatively Low
703	155569	Yunaconitine	-4.812	-5.775	Relatively Low
704	5372945	Paprazine	-6.822	-5.774	Relatively Low
705	9809542	Ginsenoside F1	-6.537	-5.773	Relatively Low
706	13966122	Rosmanol	-4.929	-5.771	Relatively Low
707	3037884	Hirsutine	-5.566	-5.771	Relatively Low
708	6890	Gramine	-6.498	-5.765	Relatively Low
709	446495	Maltotetraose	-6.846	-5.757	Relatively Low
710	71307564	Cyasterone	-4.948	-5.755	Relatively Low
711	10163855	Vincosamide	-6.38	-5.752	Relatively Low
712	16757287	20(R)-Notoginsenoside R2	-6.341	-5.751	Relatively Low
713	122169315	(2R,3R)-3,7,4'-Trihydroxy-5-methoxy-8-prenylflavanone	-8.719	-5.751	Relatively Low
714	9929189	Sophoflavescenol	-7.767	-5.748	Relatively Low
715	5320496	Pilloin	-6.524	-5.747	Relatively Low
716	3083726	8-Hydroxybergapten	-5.765	-5.746	Relatively Low

717	5464452	Sinomenine hydrochloride	-5.823	-5.746	Relatively Low
718	12127	Isovanillin	-5.732	-5.744	Relatively Low
719	14463159	Periplocin	-6.66	-5.743	Relatively Low
720	5464170	irigenin	-6.147	-5.742	Relatively Low
721	5316860	Eleutheroside B	-8.092	-5.741	Relatively Low
722	6694	Rhodamine B	-7.122	-5.741	Relatively Low
723	5281806	Psoralidin	-6.471	-5.741	Relatively Low
724	124219	Rubimaillin	-6.284	-5.741	Relatively Low
725	115723	BETA-Febrifugine	-6.957	-5.74	Relatively Low
726	5281616	Galangin	-7.178	-5.739	Relatively Low
727	164619	D-Pinitol	-6.786	-5.738	Relatively Low
728	137795177	17-Hydroxyisolathyrol	-5.588	-5.735	Relatively Low
729	5318565	Isofraxidin	-6.099	-5.735	Relatively Low
730	10246505	Licoflavone C	-7.943	-5.734	Relatively Low
731	160511	Abrine	-5.852	-5.731	Relatively Low
732	808227	PiperlotineC	-6.561	-5.731	Relatively Low
733	5318980	Icaritin	-7.112	-5.73	Relatively Low
734	513197	Isoxanthohumol	-7.8	-5.729	Relatively Low
735	6029	Uridine	-6.385	-5.726	Relatively Low
736	5316803	1,7-Dihydroxy-2,3-methylenedioxyxanthone	-6.882	-5.726	Relatively Low
737	10748	Herniarin	-5.635	-5.722	Relatively Low
738	15223582	Baicalin methyl ester	-5.794	-5.722	Relatively Low
739	71609288	Ziyuglycoside I	-4.068	-5.721	Relatively Low

740	5318645	Isorhamnetin-3-O-beta-D-Glucoside	-6.988	-5.72	Relatively Low
741	5319333	Emodin 1-glucoside	-7.794	-5.719	Relatively Low
742	20055771	14-Benzoylaconine	-4.839	-5.717	Relatively Low
743	5477212	Solanesol	-5.079	-5.716	Relatively Low
744	69867	Indole-3-carboxylic acid	-5.247	-5.711	Relatively Low
745	6070	Veratramine	-5.769	-5.711	Relatively Low
746	13820511	Isorosmanol	-6.5	-5.709	Relatively Low
747	46886723	Methylophiopogonanone B	-6.804	-5.708	Relatively Low
748	68082	Isobergapten	-5.7	-5.707	Relatively Low
749	127584	7-Ethylcamptothecin	-6.205	-5.707	Relatively Low
750	24721373	Rubusoside	-5.527	-5.704	Relatively Low
751	656516	Amygdalin	-6.609	-5.703	Relatively Low
752	71307451	atractyloside A	-6.835	-5.699	Relatively Low
753	91973812	Tenacissoside I	-5.415	-5.697	Relatively Low
754	99693	Skimmin	-6.132	-5.697	Relatively Low
755	86328677	Isorhamnetin-3-O-galactoside	-7.618	-5.693	Relatively Low
756	72	Protocatechuic Acid	-5.599	-5.691	Relatively Low
757	6508	Quinic Acid	-5.922	-5.691	Relatively Low
758	6255462	Resveratrol 4'-methyl ether	-7.14	-5.691	Relatively Low
759	107971	Daidzin	-6.567	-5.69	Relatively Low

760	176079	Calenduloside E	-4.056	-5.69	Relatively Low
761	10422896	KinsenosideKinsenoside	-5.732	-5.686	Relatively Low
762	6917864	Artesunate	-4.769	-5.684	Relatively Low
763	23616873	Continentalic acid	-4.52	-5.681	Relatively Low
764	23872112	1-(3,4-dimethoxyphenyl)-2-(4- allly-2,6-dimethoxyphenoxy)propan-1-ol	-7.265	-5.678	Relatively Low
765	14135335	Chrysin 7-glucuronide	-6.052	-5.678	Relatively Low
766	102594479	2"-O-beta-L-galactopyranosylorientin	-6.376	-5.678	Relatively Low
767	21599923	20(R)-Ginsenoside Rh1	-5.549	-5.676	Relatively Low
768	6479915	Methyl rosmarinate	-6.386	-5.676	Relatively Low
769	21588226	Tenuifolin	-5.771	-5.676	Relatively Low
770	146487	Daphnetin 7-methyl ether	-5.786	-5.673	Relatively Low
771	5281666	Kaempferide	-6.343	-5.669	Relatively Low
772	5379033	Dehydrodiisoeugenol	-6.452	-5.668	Relatively Low
773	802	Indole-3-acetic acid	-5.743	-5.668	Relatively Low
774	5280961	Genistein	-7.23	-5.667	Relatively Low
775	10153	Corydine	-6.692	-5.666	Relatively Low
776	121587	Galanthamine hydrobromide	-5.731	-5.665	Relatively Low
777	13844274	Schizantherin E	-5.028	-5.66	Relatively Low
778	72767078	Eupalinilide B	-5.036	-5.651	Relatively Low

779	5351344	Combretastatin A4	-6.246	-5.647	Relatively Low
780	23682211	Sinigrin	-5.138	-5.645	Relatively Low
781	10076238	Liquiritin apioside	-5.954	-5.644	Relatively Low
782	160500	Corytuberine	-6.322	-5.644	Relatively Low
783	4970	Protopine	-5.075	-5.641	Relatively Low
784	122826	Aaptamine	-5.189	-5.638	Relatively Low
785	101688189	Cistanoside F	-6.672	-5.637	Relatively Low
786	1548943	Capsaicin	-5.702	-5.637	Relatively Low
787	5481968	Morusinol	-6.348	-5.636	Relatively Low
788	96710	Aristololactam	-5.7	-5.636	Relatively Low
789	5354284	5,7-Dihydroxy-4-methylcoumarin	-6.815	-5.635	Relatively Low
790	94160	Mitraphylline	-6.929	-5.635	Relatively Low
791	8417	Scoparone	-5.684	-5.634	Relatively Low
792	71621984	4''-methyloxy-Genistin	-5.524	-5.632	Relatively Low
793	91884885	Ganoderenic acid D	-4.206	-5.63	Relatively Low
794	15558620	Alisol B	-4.376	-5.627	Relatively Low
795	5281780	3,4-Dicaffeoylquinic acid	-4.633	-5.625	Relatively Low
796	5280460	Scopoletin	-5.917	-5.623	Relatively Low
797	13606036	Agarotetrol	-7.146	-5.622	Relatively Low
798	197173	Monocrotaline N-Oxide	-5.252	-5.619	Relatively Low
799	134715174	Gambogellic acid	-5.118	-5.616	Relatively Low
800	6253	Arabinocytosine	-6.12	-5.616	Relatively Low
801	440735	Eriodictyol	-6.399	-5.614	Relatively Low

802	5280373	Biochanin A	-7.006	-5.613	Relatively Low
803	122196267	Isoginsenoside Rh3	-5.276	-5.61	Relatively Low
804	21626375	Gypsogenin 3-O-β-D-glucuronopyranoside	-4.325	-5.61	Relatively Low
805	5318581	Isokurarinone	-6.393	-5.609	Relatively Low
806	11018329	Polyphyllin I	-6.719	-5.609	Relatively Low
807	5281708	Daidzein	-7.173	-5.607	Relatively Low
808	5392245	Calycanthine	-5.634	-5.607	Relatively Low
809	54682930	4-Hydroxycoumarin	-5.292	-5.604	Relatively Low
810	125181686	Rhmannioside C	-6.631	-5.602	Relatively Low
811	5281608	Chrysosplenetin B	-6.582	-5.602	Relatively Low
812	14539911	Norisoboldine	-6.906	-5.602	Relatively Low
813	11425923	15,16-Dihydrotanshinone I	-5.953	-5.601	Relatively Low
814	5473050	Pinostilbene	-7.617	-5.598	Relatively Low
815	3082301	Genipin 1-gentiobioside	-6.936	-5.597	Relatively Low
816	5488822	Baohuoside I	-5.862	-5.594	Relatively Low
817	10865257	Acetylcimigenol arabinoside	-4.477	-5.593	Relatively Low
818	23616879	Veratrosine	-7.449	-5.591	Relatively Low
819	442534	Paeoniflorin	-6.394	-5.59	Relatively Low
820	91973797	Parishin E	-6.253	-5.586	Relatively Low
821	101686456	Toringin	-6.909	-5.586	Relatively Low
822	71490387	Epoxymicheliolide	-5.856	-5.585	Relatively Low
823	174003	Pinoresinol diglucoside	-7.366	-5.579	Relatively Low
824	6293081	Flavokawain C	-6.232	-5.576	Relatively Low

825	11968867	Asperulosidic acid	-6.16	-5.575	Relatively Low
826	442514	Hematoxylin	-6.569	-5.573	Relatively Low
827	765514	1-Cinnamoylpyrrolidine	-6.458	-5.572	Relatively Low
828	5491408	Myricetin 3-O-galactoside	-6.778	-5.572	Relatively Low
829	64981	Arctigenin	-7.12	-5.571	Relatively Low
830	15294091	Ankaflavin	-6.252	-5.57	Relatively Low
831	71621987	Daidzein 7-O-beta-D-glucoside 4"-O-methylate	-6.276	-5.57	Relatively Low
832	5889042	4-Hydroxylonchocarpin	-5.348	-5.57	Relatively Low
833	15736732	Kirenol	-5.444	-5.569	Relatively Low
834	476537	Ecliptasaponin A	-5.038	-5.567	Relatively Low
835	25149302	Perisesaccharide B	-5.143	-5.565	Relatively Low
836	66065	Bergenin	-5.226	-5.564	Relatively Low
837	14034912	Prim-O-glucosylcimifugin	-6.992	-5.562	Relatively Low
838	104842	7-Ethyl-10-Hydroxycamptothecin	-6.463	-5.56	Relatively Low
839	10658	Angelicin	-5.825	-5.559	Relatively Low
840	9548634	Glucoraphanin	-5.678	-5.559	Relatively Low
841	13844298	Chicanin	-6.388	-5.558	Relatively Low
842	5281343	5,7-Dihydroxychromone	-5.96	-5.558	Relatively Low
843	5318999	Licochalcone B	-6.516	-5.558	Relatively Low
844	243	Benzoic acid	-5.207	-5.557	Relatively Low
845	5356121	Flavokawain B	-6.481	-5.555	Relatively Low

846	660	Dihydrocoumarin	-5.318	-5.554	Relatively Low
847	21630000	Liriope muscari baily saponins C	-7.149	-5.554	Relatively Low
848	20839223	Ginsenoside Rh3	-4.329	-5.553	Relatively Low
849	5281408	Rhynchophylline	-4.226	-5.553	Relatively Low
850	6477182	Dimethylcurcumin	-6.822	-5.552	Relatively Low
851	6305	L-Tryptophan	-5.416	-5.551	Relatively Low
852	5318869	Kumatakenin	-7.778	-5.55	Relatively Low
853	471005	Ganoderic Acid H	-4.388	-5.548	Relatively Low
854	9804842	(-)-EGCG-3"-O-Me	-6.681	-5.547	Relatively Low
855	2214	Acetovanillone	-6.088	-5.546	Relatively Low
856	5318547	Isodemethylwedelolactone	-6.692	-5.546	Relatively Low
857	11790	alpha-Naphthoflavone	-6.36	-5.543	Relatively Low
858	14019178	Kajiichigoside F1	-3.978	-5.54	Relatively Low
859	16401086	10-hydroxy aconitine	-4.376	-5.537	Relatively Low
860	445858	Ferulic Acid	-4.849	-5.535	Relatively Low
861	5281636	Gentisin	-6.334	-5.53	Relatively Low
862	36462	Etoposide	-5.937	-5.529	Relatively Low
863	21599928	Ginsenoside Rh4	-5.097	-5.525	Relatively Low
864	5280448	Calycosin	-6.708	-5.523	Relatively Low
865	13846690	Protosappanin B	-6.104	-5.522	Relatively Low
866	5280804	Isoquercitrin	-7.255	-5.521	Relatively Low
867	5375252	Noreugenin	-6.272	-5.52	Relatively Low
868	91144	Farrerol	-6.644	-5.515	Relatively Low
869	14140130	Hydroprotopine	-6.711	-5.515	Relatively Low

870	5481237	Kushenol I	-8.182	-5.515	Relatively Low
871	12305198	Arenobufagin	-5.736	-5.514	Relatively Low
872	5316653	Dichotomitin	-6.842	-5.514	Relatively Low
873	641785	Cardamonin	-7.237	-5.511	Relatively Low
874	11982272	Silydianin	-6.104	-5.51	Relatively Low
875	7172	Synephrine	-5.664	-5.507	Relatively Low
876	42609626	Synephrine hydrochloride	-5.664	-5.507	Relatively Low
877	442432	paederoside	-6.395	-5.505	Relatively Low
878	323	Coumarin	-4.967	-5.505	Relatively Low
879	6755	Lawsone	-6.132	-5.504	Relatively Low
880	134715187	Astraganoside	-5.283	-5.502	Relatively Low
881	73191	Nodakenin	-6.232	-5.501	Relatively Low
882	72340	Tetrahydroalstonine	-6.038	-5.5	Relatively Low
883	5489605	Demethylwedelolactone	-6.127	-5.499	Relatively Low
884	91520	Catalpol	-5.378	-5.495	Relatively Low
885	91895267	Koumine	-4.424	-5.494	Relatively Low
886	5318650	Isorhapontigenin	-6.531	-5.49	Relatively Low
887	24721282	Esculentoside A	-6.545	-5.484	Relatively Low
888	441145	Monensin	-5.729	-5.482	Relatively Low
889	5281672	Myricetin	-6.125	-5.482	Relatively Low
890	5315472	Bisdemethoxycurcumin	-6.961	-5.48	Relatively Low
891	1794427	Chlorogenic acid	-5.887	-5.479	Relatively Low
892	129371873	Laetanine	-6.839	-5.477	Relatively Low
893	102507168	Jasminoside B	-6.346	-5.474	Relatively Low
894	3469	Gentisic acid	-5.779	-5.474	Relatively Low

895	3081405	Phellodendrine	-6.276	-5.474	Relatively Low
896	59818	Phellodendrine chloride	-5.424	-5.474	Relatively Low
897	10168	Rhein	-6.699	-5.47	Relatively Low
898	7014	Sparteine	-5.652	-5.465	Relatively Low
899	85245726	Ginsenoside Rh8	-6.004	-5.463	Relatively Low
900	10217	Dihydroberberine	-4.717	-5.462	Relatively Low
901	87691	Loganin	-5.566	-5.462	Relatively Low
902	72281	Hesperetin	-7.408	-5.456	Relatively Low
903	6990	2,4-Dihydroxyacetophenone	-6.181	-5.453	Relatively Low
904	95693	Isoacetovanillone	-5.581	-5.451	Relatively Low
905	42994	Lindleyin	-7.502	-5.448	Relatively Low
906	441742	Karacoline	-4.87	-5.447	Relatively Low
907	14699964	Bernardioside A	-5.272	-5.445	Relatively Low
908	386331	3,5,6,7,3',4'-Hexamethoxyflavone	-6.214	-5.443	Relatively Low
909	162823	8-O-Acetyl shanzhiside methyl ester	-5.203	-5.442	Relatively Low
910	187808	Glycitin	-6.325	-5.441	Relatively Low
911	591524	2-hydroxy-6-methoxybenzoic acid）	-5.744	-5.439	Relatively Low
912	102004869	Ophiogenin 3-O-α-L-rhamnopyranosyl(1→2)	-4.323	-5.438	Relatively Low

		[β-D-xylopyranosyl(1→3)]-β-D-glucopyranoside			
913	5646	Usnic acid	-7.767	-5.438	Relatively Low
914	5319741	Methylophiopogonanone A	-6.897	-5.437	Relatively Low
915	5271805	Ginkgetin	-7.257	-5.436	Relatively Low
916	10243535	4'-Hydroxy-5,6-dehydro kawain	-6.95	-5.433	Relatively Low
917	5320315	Oroxylin A	-6.054	-5.433	Relatively Low
918	10211	Byakangelicin	-6.002	-5.432	Relatively Low
919	6476139	3-O-Caffeoylquinic acid methyl ester	-6.978	-5.431	Relatively Low
920	10062187	Nortanshinone	-6.401	-5.429	Relatively Low
921	10022392	Angelol B	-5.759	-5.428	Relatively Low
922	442872	securinine	-5.414	-5.428	Relatively Low
923	124072	Tetrahydrocurcumin	-7.415	-5.426	Relatively Low
924	1183	Vanillin	-6.005	-5.426	Relatively Low
925	4978	Pseudohypericin	-7.974	-5.426	Relatively Low
926	11968396	Danmelittoside	-6.046	-5.421	Relatively Low
927	11492597	DihydroDaidzein	-7.106	-5.42	Relatively Low
928	5281565		-6.93	-5.418	Relatively Low
929	69894	Isoscopoletin	-6.444	-5.418	Relatively Low
930	636822	Asarone	-5.765	-5.413	Relatively Low

931	11667940	Neotuberostemonine	-5.09	-5.413	Relatively Low
932	16401639	steviolbioside	-5.493	-5.412	Relatively Low
933	5318998	Licochalcone A	-7.928	-5.412	Relatively Low
934	5281811	Tectorigenin	-6.753	-5.411	Relatively Low
935	11596309	Glabrol	-7.556	-5.409	Relatively Low
936	5490064	Avicularin	-7.895	-5.408	Relatively Low
937	26305	Nodakenitin	-7.507	-5.407	Relatively Low
938	54706833	2-O-β-D-Glucopyranosyl-L- ascorbic acid	-5.98	-5.404	Relatively Low
939	101249251	sargentol	-6.549	-5.402	Relatively Low
940	442725	Aurantio-obtusin β-D-glucoside	-6.466	-5.402	Relatively Low
941	16094542	5,7,4'-Trihydroxy-8-methylflavanone	-7.944	-5.399	Relatively Low
942	3082856	Sesamoside	-5.842	-5.395	Relatively Low
943	44715524	Darutoside	-5.254	-5.394	Relatively Low
944	348130	Sakuranetin	-7.275	-5.392	Relatively Low
945	10836072	Smyrindioloside	-6.716	-5.39	Relatively Low
946	443354	Geniposidic acid	-5.164	-5.389	Relatively Low
947	338	Salicylic acid	-5.343	-5.388	Relatively Low
948	320711	Thalidezine	-4.377	-5.388	Relatively Low
949	51346120	Lappaconitine hydrobromide	-5.876	-5.385	Relatively Low
950	23655938	(2R)-8-Methylsocotrin-4'-ol	-7.707	-5.384	Relatively Low
951	9978650	8-Acetylharpagide	-5.346	-5.383	Relatively Low

952	155380	Obtusin	-4.86	-5.383	Relatively Low
953	6325269	Paederosidic acid methyl ester	-6.224	-5.38	Relatively Low
954	890	Phytic acid	-3.748	-5.377	Relatively Low
955	5355469	Flavokawain A	-6.402	-5.377	Relatively Low
956	5471851	Poricoic acid A	-5.197	-5.376	Relatively Low
957	471719	7-O-Methylbaicalein	-6.491	-5.376	Relatively Low
958	5320203	6,7-dihydroxy-4-phenylcoumarin	-5.989	-5.376	Relatively Low
959	10207	Aloe emodin	-7.157	-5.373	Relatively Low
960	162868	Secoxyloganin	-5.618	-5.373	Relatively Low
961	10065830	Methylophiopogonone A	-6.916	-5.373	Relatively Low
962	92043620	vina-ginsenoside R3	-5.176	-5.37	Relatively Low
963	138111462	Eupalinolide K	-6.397	-5.369	Relatively Low
964	134715164	Licochalcone E	-6.846	-5.368	Relatively Low
965	60699	Topotecan Hydrochloride	-6.684	-5.367	Relatively Low
966	60700	Topotecan	-6.684	-5.367	Relatively Low
967	11020893	Dihydroartemisinic acid	-4.46	-5.365	Relatively Low
968	144	:5-Hydroxy-DL-tryptophan	-5.528	-5.364	Relatively Low
969	63123	Arborin	-6.676	-5.357	Relatively Low
970	14109405	Ganoderic acid D	-4.353	-5.357	Relatively Low
971	137796517	(9Z,12Z)-N-(3-	-6.624	-5.356	Relatively Low

		Methoxybenzyl)octadeca-9,12-dienamide			
972	442674	Chebulagic acid	-3.971	-5.356	Relatively Low
973	65090	Xanthotoxol	-5.816	-5.355	Relatively Low
974	10992619	Zeylenol	-6.764	-5.353	Relatively Low
975	44568160	Corynoxeine	-4.631	-5.353	Relatively Low
976	12315350	Deacetylasperulosidic acid	-5.696	-5.352	Relatively Low
977	472335	Rubitecan	-6.324	-5.351	Relatively Low
978	5316802	Kanzonol C	-7.136	-5.351	Relatively Low
979	78071341	Deacetyl ganoderic acid F	-4.912	-5.35	Relatively Low
980	135398638	Hypoxanthine	-5.921	-5.35	Relatively Low
981	44561398	Tenacissoside G	-6.515	-5.349	Relatively Low
982	102004640	N-Methylnuciferine	-6.757	-5.349	Relatively Low
983	44159808	Garcinone C	-6.296	-5.346	Relatively Low
984	65305	Cephalotaxine	-5.387	-5.346	Relatively Low
985	442106	Hypaphorine	-5.924	-5.343	Relatively Low
986	102144112	Acetylseneciphylline N-oxide	-4.6	-5.341	Relatively Low
987	11948668	Shanzhiside	-6.09	-5.34	Relatively Low
988	442977	Imperialine	-5.269	-5.338	Relatively Low
989	88308	Dihydromethysticin	-6.851	-5.334	Relatively Low
990	21637711	Rehmapicroside	-5.656	-5.333	Relatively Low
991	115221	Ginkgolide A	-4.218	-5.332	Relatively Low

992	114917	Tanshinone I	-5.749	-5.33	Relatively Low
993	5491637	Iristectorigenin A	-6.164	-5.329	Relatively Low
994	73117	Pinoresinol dimethyl ether	-5.466	-5.327	Relatively Low
995	442070	Phorbol	-5.365	-5.327	Relatively Low
996	5280378	Formononetin	-6.519	-5.327	Relatively Low
997	115196	Brassinolide	-4.016	-5.323	Relatively Low
998	5321919	Thalrugosaminine	-4.021	-5.321	Relatively Low
999	5379096	Jaceosidin	-6.85	-5.319	Relatively Low
1000	3055167	Byakangelicol	-6.264	-5.317	Relatively Low
1001	5281406	Dephnoretin	-6.248	-5.316	Relatively Low
1002	10189	Eugenin	-6.013	-5.315	Relatively Low
1003	21589010	(-)-corynoxidine	-5.443	-5.314	Relatively Low
1004	5315865	3'-O-Acetylhamaudol	-5.709	-5.313	Relatively Low
1005	5458190	Catharanthine	-5.696	-5.313	Relatively Low
1006	91895516	Catharanthine hemitartrate	-5.696	-5.313	Relatively Low
1007	72343	Hernandezine	-4.406	-5.312	Relatively Low
1008	6303	Cordycepin	-6.687	-5.31	Relatively Low
1009	14157896	Loureirin C	-6.377	-5.31	Relatively Low
1010	5281607	Chrysin	-7.143	-5.309	Relatively Low
1011	204810	Pseudobufarenogin	-5.796	-5.307	Relatively Low
1012	5319000	Licoflavone A	-6.948	-5.306	Relatively Low
1013	441960	Cimifugin	-7.009	-5.304	Relatively Low
1014	24721095	Gardenoside	-6.753	-5.302	Relatively Low
1015	91457	Beta-Eudesmol	-6.067	-5.301	Relatively Low

1016	4788	Phloretin	-5.865	-5.301	Relatively Low
1017	3037151	Hirsuteine	-5.654	-5.3	Relatively Low
1018	72376	Narciclasine	-7.236	-5.299	Relatively Low
1019	135	4-Hydroxybenzoic acid	-5.476	-5.296	Relatively Low
1020	442169	Armepavine	-5.669	-5.296	Relatively Low
1021	11869417	Asarinin	-6.663	-5.294	Relatively Low
1022	5320755	Pseudoaspidin	-5.596	-5.292	Relatively Low
1023	65567	10-Nitro Camptothecin	-6.115	-5.291	Relatively Low
1024	5316097	Corylin	-6.704	-5.285	Relatively Low
1025	5281643	Hyperoside	-8.042	-5.285	Relatively Low
1026	72310	Columbamine	-5.694	-5.28	Relatively Low
1027	8357	3,4,5-trimethoxybenzoic acid	-5.591	-5.276	Relatively Low
1028	441913	Cimicifugoside	-4.051	-5.276	Relatively Low
1029	190453	Mulberroside C	-6.552	-5.275	Relatively Low
1030	5742590	Eleutheroside A	-4.571	-5.274	Relatively Low
1031	3664	hypocrellin A	-5.746	-5.27	Relatively Low
1032	14031163	Bayogenin 3-O-β-D-glucopyranoside	-5.536	-5.268	Relatively Low
1033	5319322	Medicagol	-6.203	-5.265	Relatively Low
1034	15886258	20(S),24(R)-Ocotillol	-4.134	-5.264	Relatively Low
1035	98055304	Ranaconitine	-6.139	-5.264	Relatively Low
1036	57041970	Triglochinic acid	-4.288	-5.26	Relatively Low
1037	442249	Ipecoside	-6.375	-5.259	Relatively Low

1038	45482321	Episyringaresinol 4'-O-β-D-glncopyranoside	-7.196	-5.254	Relatively Low
1039	10659145	Osthol hydrate	-5.732	-5.253	Relatively Low
1040	102004875	Precyasterone	-4.288	-5.251	Relatively Low
1041	5320351	Artemitin	-6.193	-5.25	Relatively Low
1042	6199	Psoralen	-6.034	-5.248	Relatively Low
1043	10170	Berbamine	-5.487	-5.247	Relatively Low
1044	5318214	3'-hydroxygenkwanin	-6.961	-5.246	Relatively Low
1045	155948	Atractylenolide III	-5.602	-5.246	Relatively Low
1046	185617	Scutellarin	-7.187	-5.245	Relatively Low
1047	10163	Lucidin	-5.69	-5.245	Relatively Low
1048	46240156	Norcimifugin	-7.252	-5.244	Relatively Low
1049	10337211	Bavachinin	-6.66	-5.243	Relatively Low
1050	12799036	Oxyepiberberine	-6.005	-5.241	Relatively Low
1051	165274	Aristolochic acid C	-5.803	-5.239	Relatively Low
1052	118856308	Cimicidanol-3-O-alpha-L-arabinoside	-5.819	-5.238	Relatively Low
1053	592704	Nudifloric acid	-5.566	-5.237	Relatively Low
1054	442088	Evodiamine	-6.5	-5.231	Relatively Low
1055	56926890	Taccalonolide AJ	-3.984	-5.23	Relatively Low
1056	78381113	Lotusine	-7.58	-5.23	Relatively Low
1057	5316525	7-Demethylsuberosin	-5.871	-5.228	Relatively Low
1058	5281653	Methylswertianin	-5.641	-5.228	Relatively Low
1059	13844293	Wulignan A1	-5.822	-5.228	Relatively Low

1060	11914	Mandelic acid	-5.458	-5.226	Relatively Low
1061	638278	Isoliquiritigenin	-8.363	-5.223	Relatively Low
1062	5281575	Yangonin	-6.012	-5.219	Relatively Low
1063	60208818	Mulberroside F	-7.093	-5.217	Relatively Low
1064	14528828	1,2,3,7-tetramethoxyxanthone	-5.997	-5.216	Relatively Low
1065	363209	Demethyleneberberine	-6.599	-5.214	Relatively Low
1066	100781	Tuberostemonine	-6.313	-5.213	Relatively Low
1067	75412558	Salvianolic acid D	-6.289	-5.212	Relatively Low
1068	5462442	Huperzine B	-5.793	-5.212	Relatively Low
1069	3083575	Obtusifolin	-6.395	-5.21	Relatively Low
1070	5745470	Icariside I	-6.442	-5.206	Relatively Low
1071	3084335	magnolioside	-6.377	-5.203	Relatively Low
1072	13936691	Eurycomanone	-5.2	-5.201	Relatively Low
1073	124928615	Perisesaccharide C	-5.304	-5.201	Relatively Low
1074	14286954	Pedunculoside	-4.174	-5.199	Relatively Low
1075	151108	L-Quebrachitol	-7.387	-5.199	Relatively Low
1076	6325021	6-alpha-Hydroxygeniposide	-6.037	-5.199	Relatively Low
1077	5281605	Baicalein	-7.228	-5.199	Relatively Low
1078	102004874	Ecliptasaponin D	-4.506	-5.195	Relatively Low
1079	13343336	Benzoylhypaconine	-5.622	-5.192	Relatively Low
1080	154279	Alpinetin	-8.105	-5.19	Relatively Low
1081	24360	Camptothecin	-6.731	-5.189	Relatively Low
1082	5280445	Luteolin	-5.905	-5.188	Relatively Low
1083	12855920	Ginsenoside Rh1	-5.763	-5.183	Relatively Low

1084	5380876	Senecionine N-Oxide	-4.732	-5.182	Relatively Low
1085	638024	Piperine	-6.403	-5.182	Relatively Low
1086	9851101	Toosendanin	-5.151	-5.18	Relatively Low
1087	78384572	Uvarigrin	-6.035	-5.18	Relatively Low
1088	9415	Monocrotaline	-4.661	-5.179	Relatively Low
1089	14448070	Atractylenolide II	-6.208	-5.177	Relatively Low
1090	5810	L-Hydroxyproline	-5.322	-5.175	Relatively Low
1091	6169	Yohimbine Hydrochloride	-5.503	-5.175	Relatively Low
1092	165536	Aristolone	-5.012	-5.174	Relatively Low
1093	69600	4-Methoxysalicylaldehyde	-6.035	-5.174	Relatively Low
1094	479499	Beta,beta-Dimethylacrylshikonin	-7.038	-5.172	Relatively Low
1095	25087713	Geoside	-6.709	-5.169	Relatively Low
1096	5321286	Shikonofuran A	-6.567	-5.169	Relatively Low
1097	124886	Glutathione	-5.418	-5.169	Relatively Low
1098	68081	Isoimperatorin	-5.908	-5.168	Relatively Low
1099	427877	Sarracenin	-5.459	-5.166	Relatively Low
1100	161036	Sweroside	-5.57	-5.166	Relatively Low
1101	5280536	4-Hydroxy-3-methoxycinnamaldehyde	-4.909	-5.166	Relatively Low
1102	5281671	Morusin	-6.987	-5.164	Relatively Low
1103	160481	Isosakuranetin	-6.774	-5.163	Relatively Low
1104	10143	Isocorydine	-6.614	-5.162	Relatively Low

1105	3083928	Di-O-methylfraxetin	-5.508	-5.16	Relatively Low
1106	101600079	Ganoderenic acid C	-4.723	-5.16	Relatively Low
1107	68071	Pinocembrin	-7.991	-5.157	Relatively Low
1108	72965	Ailanthone	-5.226	-5.156	Relatively Low
1109	10237	Bicuculline	-6.628	-5.153	Relatively Low
1110	457928	Bevirimat	-4.44	-5.152	Relatively Low
1111	5273568	Fraxin	-7.649	-5.152	Relatively Low
1112	670971	N-Methylcytisine	-5.901	-5.144	Relatively Low
1113	602152	Isomucronulatol	-6.62	-5.143	Relatively Low
1114	6450278	Guggulsterone E&Z	-4.827	-5.138	Relatively Low
1115	21602024	Daphylloside	-6.186	-5.137	Relatively Low
1116	164676	Tanshinone IIA	-6.602	-5.134	Relatively Low
1117	71456946	Songorine	-5.811	-5.134	Relatively Low
1118	45358012	Ginkgolide J	-4.776	-5.133	Relatively Low
1119	15109	2,6-Dimethoxybenzoic acid	-5.216	-5.13	Relatively Low
1120	441793	Eurycomalactone	-5.379	-5.129	Relatively Low
1121	170569	Irisflorentin	-6.597	-5.126	Relatively Low
1122	10154	Boldine	-6.265	-5.126	Relatively Low
1123	2327270	1-(3,4-Dimethoxycinnamoyl)piperidine	-6.902	-5.125	Relatively Low
1124	10206	Cepharanthine	-5.147	-5.122	Relatively Low
1125	54693473	2-O-α-D-Glucopyranosyl-L- ascorbic acid	-4.973	-5.121	Relatively Low
1126	441831	Podecdysone B	-6.03	-5.12	Relatively Low

1127	15296614	PeucedanolPeucedanol	-6.279	-5.12	Relatively Low
1128	5281691	beta-Rhamnocitrin	-7.325	-5.12	Relatively Low
1129	198051	Rhodojaponin II	-4.127	-5.119	Relatively Low
1130	10604651	4-O-beta-Glucopyranosyl-cis-coumaric acid	-5.325	-5.118	Relatively Low
1131	3085362	Gigantol	-6.74	-5.115	Relatively Low
1132	161218	Aristolochic acid D	-5.803	-5.114	Relatively Low
1133	73285	Isoalantolactone	-5.283	-5.112	Relatively Low
1134	65126	Carnosic acid	-4.336	-5.112	Relatively Low
1135	927642	Cochliophilin A	-6.762	-5.11	Relatively Low
1136	10429112	Uncarine C	-6.924	-5.108	Relatively Low
1137	122667	4'-Demethylpodophyllotoxin	-5.984	-5.106	Relatively Low
1138	4114	Xanthotoxin	-5.812	-5.104	Relatively Low
1139	23149	Magnoflorine chloride	-6.482	-5.104	Relatively Low
1140	73337	Magnoflorine	-6.482	-5.104	Relatively Low
1141	6451920	Magnoflorine Iodide	-6.482	-5.104	Relatively Low
1142	14236566	Bavachin	-8.035	-5.103	Relatively Low
1143	53266	Magnocurarine	-6.948	-5.1	Relatively Low
1144	5281417	Esculin	-6.324	-5.1	Relatively Low
1145	311	Citric acid	-4.029	-5.097	Relatively Low
1146	8742	Shikimic acid	-5.875	-5.096	Relatively Low
1147	10320238	Bruceine H	-4.904	-5.095	Relatively Low
1148	6442675	Echinatin	-6.43	-5.095	Relatively Low
1149	10742	Syringic acid	-5.452	-5.09	Relatively Low

1150	5317598	Alnustone	-6.138	-5.09	Relatively Low
1151	10478550	Arnidiol	-3.757	-5.09	Relatively Low
1152	162878	Tarasaponin VI	-4.686	-5.089	Relatively Low
1153	73399	(+)-Pinoresinol	-5.28	-5.088	Relatively Low
1154	84800	Primin	-5.817	-5.088	Relatively Low
1155	73657193	Ganoderic Acid G	-5.49	-5.087	Relatively Low
1156	151529	Schizantherin A	-4.74	-5.082	Relatively Low
1157	5708351	14-Deoxy-11,12-didehydroandrographolide	-4.541	-5.082	Relatively Low
1158	71307576	Talatisamine	-3.89	-5.081	Relatively Low
1159	188316	Moslosooflavone	-7.213	-5.076	Relatively Low
1160	24721165	Isocolumbin	-5.025	-5.074	Relatively Low
1161	5070783	Meranzin hydrate	-5.359	-5.072	Relatively Low
1162	5281623	Bellidifolin	-6.474	-5.072	Relatively Low
1163	275182	Berbamine dihydrochloride	-4.716	-5.071	Relatively Low
1164	21633061	Deacylmetaplexigenin	-6.312	-5.069	Relatively Low
1165	85364165	13-Oxyingenol dodecanoat	-4.828	-5.066	Relatively Low
1166	5281416	Esculetin	-6.319	-5.066	Relatively Low
1167	441805	Rutaevin	-4.046	-5.064	Relatively Low
1168	160254	Cryptotanshinone	-6.564	-5.062	Relatively Low
1169	231412	Fuscaxanthone C	-5.698	-5.062	Relatively Low
1170	5322079	Isoanhydroicaritin	-6.45	-5.062	Relatively Low
1171	5468749	Kaempferol	-6.671	-5.061	Relatively Low

		3,7,4'-trimethyl ether			
1172	100341	Griffonilide	-6.196	-5.06	Relatively Low
1173	5281804	Prunetin	-6.875	-5.058	Relatively Low
1174	73062	Kaurenoic acid	-5.03	-5.057	Relatively Low
1175	20054813	Aconine	-5.284	-5.056	Relatively Low
1176	969488	D-Tetrahydropalmatine	-6.421	-5.056	Relatively Low
1177	164660	Protohypericin	-6.261	-5.054	Relatively Low
1178	164879	Carabrone	-5.964	-5.052	Relatively Low
1179	5281727	Pterostilbene	-6.498	-5.049	Relatively Low
1180	5281511	Xanthatin	-5.723	-5.048	Relatively Low
1181	471722	5-Hydroxy-6,7-dimethoxylflavone	-5.877	-5.048	Relatively Low
1182	15513544	De-O-acetylcinobufotalin	-4.611	-5.046	Relatively Low
1183	150893	3,5,6,7,8,3',4'-Heptamethoxyflavone	-6.091	-5.045	Relatively Low
1184	131751666	8-epidiosbulbin E acetate	-4.91	-5.042	Relatively Low
1185	177359	Interiotherin A	-4.502	-5.042	Relatively Low
1186	440936	Arbutin	-6.153	-5.04	Relatively Low
1187	14108469	Wilforgine	-4.642	-5.039	Relatively Low
1188	122839	Sennidin A	-5.155	-5.036	Relatively Low
1189	10459879	Sennidin B	-4.297	-5.036	Relatively Low
1190	5280371	Bergaptol	-5.74	-5.036	Relatively Low
1191	11877495	Desacetylcinobufagin	-4.51	-5.032	Relatively Low

1192	5281954	7-O-Methylchrysin	-6.713	-5.032	Relatively Low
1193	44575270	Andropanoside	-6.486	-5.031	Relatively Low
1194	22179	Norboldine	-7.358	-5.03	Relatively Low
1195	123617	9-Methoxycamptothecin	-6.129	-5.029	Relatively Low
1196	5280906	Senecionine	-5.418	-5.028	Relatively Low
1197	5488919	3,3'-Di-O-methylellagic acid	-5.659	-5.027	Relatively Low
1198	5481224	Quercetin 3-O-alpha-L-Arabinopyranoside	-8.44	-5.026	Relatively Low
1199	89640	Loganic acid	-5.703	-5.025	Relatively Low
1200	161271	Salvigenin	-5.727	-5.021	Relatively Low
1201	90476678	Qingyangshengenin	-5.145	-5.021	Relatively Low
1202	145659	Sinensetin	-5.786	-5.02	Relatively Low
1203	115269	Sophocarpine	-6.283	-5.02	Relatively Low
1204	5281750	Seneciphylline	-6.623	-5.019	Relatively Low
1205	22524560	Usaramine N-oxide	-5.188	-5.017	Relatively Low
1206	354159	8,9-epoxy-3-isobutyryloxy-10-(2-methylbutanoyl)thymol	-6.099	-5.016	Relatively Low
1207	637858	PIPERLONGUMINE	-5.505	-5.016	Relatively Low
1208	5315263	Casticin	-6.542	-5.014	Relatively Low
1209	5274585	Quercetin 3-O-β-D-glucuronide	-6.44	-5.012	Relatively Low
1210	5280443	Apigenin	-8.43	-5.011	Relatively Low

1211	5321961	Toddalolactone	-5.875	-5.01	Relatively Low
1212	5464381	Velutin	-7.057	-5.007	Relatively Low
1213	7092	6-Methylcoumarin	-5.469	-5.007	Relatively Low
1214	75412555	Ginsenoside Rk3	-4.732	-5.005	Relatively Low
1215	11092	Paeonol	-5.698	-5.004	Relatively Low
1216	3564	Harmaline	-5.881	-4.995	Relatively Low
1217	6440397	Caftaric acid	-4.885	-4.993	Relatively Low
1218	21721956	Oroxylin A 7-O-beta-D-glucuronide methyl ester	-6.352	-4.993	Relatively Low
1219	4101463	Pinostrobin	-6.824	-4.989	Relatively Low
1220	10903113	Salvianolic acid F	-6.321	-4.987	Relatively Low
1221	5281662	Swertianolin	-6.841	-4.986	Relatively Low
1222	5281703	Wogonin	-7.216	-4.985	Relatively Low
1223	14240934	Hupehenine	-4.839	-4.984	Relatively Low
1224	6683	Purpurin	-6.448	-4.983	Relatively Low
1225	370	Gallic acid	-5.615	-4.982	Relatively Low
1226	13936703	13-alpha-(21)-Epoxyeurycomanone	-4.932	-4.981	Relatively Low
1227	13873655	3-Isomangostin	-4.852	-4.979	Relatively Low
1228	72724	Helenine	-5.447	-4.978	Relatively Low
1229	6651	4-p-Menthan-1,8-diol	-5.132	-4.975	Relatively Low
1230	5280569	Daphnetin	-5.313	-4.975	Relatively Low
1231	9838995	Brevifolincarboxylic acid	-6.409	-4.975	Relatively Low

1232	189695	3-Furfuryl 2-pyrrolecarboxylate	-6.579	-4.973	Relatively Low
1233	21151017	Rhodojaponin III	-5.238	-4.971	Relatively Low
1234	139291217	Mogroside I A1	-6.581	-4.967	Relatively Low
1235	442523	DendrobineDendrobine	-4.977	-4.964	Relatively Low
1236	5281633	Gartanin	-6.438	-4.957	Relatively Low
1237	6602508	Stigmasterol glucoside	-4.642	-4.951	Relatively Low
1238	735755	3,4,5-Trimethoxycinnamic acid	-5.373	-4.95	Relatively Low
1239	21592249	Paederosidic acid	-5.191	-4.949	Relatively Low
1240	5281437	Costunolide	-5.254	-4.948	Relatively Low
1241	72948694	Caudatin	-5.967	-4.948	Relatively Low
1242	222528	Deoxycholic acid	-5.355	-4.948	Relatively Low
1243	854026	Huperzine A	-5.539	-4.948	Relatively Low
1244	453213	Perakine	-4.96	-4.948	Relatively Low
1245	497204	Lindenenol acetate	-5.271	-4.943	Relatively Low
1246	5317750	Glycitein	-6.467	-4.943	Relatively Low
1247	24836956	Neocurdione	-4.762	-4.942	Relatively Low
1248	169234	Magnolin	-5.634	-4.941	Relatively Low
1249	11827970	Polyphyllin A	-4.876	-4.94	Relatively Low
1250	5321018	Atractylenolide I	-6.218	-4.939	Relatively Low
1251	14655552	Oroxyloside	-6.076	-4.939	Relatively Low
1252	1550607	Auraptene	-5.803	-4.937	Relatively Low
1253	9851693	ISOFEBRIFUGINE (B604866K055)	-7.326	-4.937	Relatively Low
1254	68085	Dictamnine	-5.265	-4.936	Relatively Low

1255	5281813	Wedelolactone	-5.966	-4.935	Relatively Low
1256	84298	Asperuloside	-6.155	-4.934	Relatively Low
1257	440229	Tetrahydrocolumbamine	-5.845	-4.932	Relatively Low
1258	119041	Obacunone	-3.283	-4.93	Relatively Low
1259	2950	1,8-Dihydroxyanthraquinone	-6.599	-4.929	Relatively Low
1260	168115	10-Gingerol	-6.308	-4.928	Relatively Low
1261	10038868	3'-Hydroxypterostilbene	-5.945	-4.926	Relatively Low
1262	10220256	Dihydrokawain	-6.413	-4.923	Relatively Low
1263	60961	Adenosine	-6.693	-4.92	Relatively Low
1264	72703	Berberrubine	-5.764	-4.92	Relatively Low
1265	12313579	N-nornuciferine	-5.776	-4.919	Relatively Low
1266	72435	Picropodophyllotoxin	-6.655	-4.918	Relatively Low
1267	65373	Secoisolariciresinol	-6.14	-4.918	Relatively Low
1268	101671037	Mesaconine	-5.143	-4.918	Relatively Low
1269	6440408	Kuwanon E	-6.038	-4.915	Relatively Low
1270	11664505	Isorhamnetin 3-O-neohesperoside	-6.719	-4.914	Relatively Low
1271	442279	Micheliolide	-6.114	-4.912	Relatively Low
1272	31244	p-Anisaldehyde	-5.257	-4.911	Relatively Low
1273	137706510	Eupalinolide A	-6.772	-4.909	Relatively Low
1274	9910474	Neoruscogenin	-6.455	-4.907	Relatively Low
1275	441887	Gitogenin	-5.578	-4.905	Relatively Low
1276	14525327	Mogrol	-4.981	-4.905	Relatively Low
1277	57396771	Ganoderic Acid C2	-4.274	-4.905	Relatively Low
1278	10358150	Eriosematin	-6.306	-4.904	Relatively Low

1279	5320438	Pectolinarigenin	-6.18	-4.904	Relatively Low
1280	71308174	Chebulic acid	-4.479	-4.904	Relatively Low
1281	160876	Epiberberine	-5.762	-4.903	Relatively Low
1282	71306915	Astragaloside II	-5.88	-4.903	Relatively Low
1283	11556558	3,29-O-Dibenzoylmultiflor-8-en-3-alpha,7beta,29-triol	-3.797	-4.903	Relatively Low
1284	5280442	Acacetin	-6.58	-4.901	Relatively Low
1285	11316212	Cowaxanthone B	-6.101	-4.9	Relatively Low
1286	101679160	Methylnissolin-3-O-glucoside	-5.668	-4.898	Relatively Low
1287	5281628	Hispidulin	-6.106	-4.896	Relatively Low
1288	197810	Chelidonine	-5.286	-4.894	Relatively Low
1289	107848	Geniposide	-5.894	-4.891	Relatively Low
1290	189670	Loureirin B	-6.941	-4.891	Relatively Low
1291	442435	Swertiamarin	-6.201	-4.888	Relatively Low
1292	5321010	Oridonin	-5.5	-4.888	Relatively Low
1293	3220	Emodin	-7.035	-4.888	Relatively Low
1294	161294	Peimisine	-4.36	-4.884	Relatively Low
1295	442042	Ingenol	-5.369	-4.883	Relatively Low
1296	3806	Juglone	-6.09	-4.883	Relatively Low
1297	101300	D-Dicentrine	-6.708	-4.883	Relatively Low
1298	14016780	Luteolin 7-sulfate	-5.461	-4.881	Relatively Low
1299	97283	10-Methoxycamptothecin	-6.161	-4.88	Relatively Low
1300	120682	Bullatine B	-5.953	-4.88	Relatively Low

1301	96539	Gardenin B	-6.001	-4.875	Relatively Low
1302	11815492	Ginsenoside Ro	-4.219	-4.873	Relatively Low
1303	632135	Isosinensetin	-6.259	-4.872	Relatively Low
1304	5284649	Demethyltexasin	-7.369	-4.872	Relatively Low
1305	5909	Pilocarpine Hydrochloride	-5.871	-4.872	Relatively Low
1306	10031398	Pseudolaric acid B-O-beta-D-glucopyranoside	-6.849	-4.871	Relatively Low
1307	5321765	O-Methylbroussochalcone B, 4'	-6.878	-4.867	Relatively Low
1308	5281946	Galangin 3-methyl ether	-6.957	-4.865	Relatively Low
1309	5944	Cantharidin	-5.806	-4.864	Relatively Low
1310	5355836	7,8-Dihydroxy-4-methylcoumarin	-5.757	-4.863	Relatively Low
1311	101746	Sesamolin	-6.085	-4.861	Relatively Low
1312	24721502	Ginkgolide C	-5.528	-4.861	Relatively Low
1313	102258917	Tenacigenin B, 3-O-β-Allopyranosyl-(1→4)-β-oleandropyranosyl-11-O-isobutyryl-12-O-acetyl-	-6.121	-4.86	Relatively Low
1314	14036813	Alisol C Monoacetate	-4.304	-4.86	Relatively Low
1315	14077830	3-Hydroxy-9,10-Dimethoxypterocarpan	-5.516	-4.86	Relatively Low

1316	15227299	Stachysterone D	-6.989	-4.859	Relatively Low
1317	71307553	8-Deacetyl yunaconitine	-5.277	-4.859	Relatively Low
1318	6989	Thymol	-5.942	-4.856	Relatively Low
1319	3663	Hypericin	-8.021	-4.854	Relatively Low
1320	441737	Hypaconitine	-4.251	-4.853	Relatively Low
1321	173273	Triptophenolide	-6.151	-4.852	Relatively Low
1322	5317519	Ganolactone B	-3.771	-4.852	Relatively Low
1323	24721310	Bulleyaconitine A	-6.221	-4.852	Relatively Low
1324	497203	Lindenenol	-6.332	-4.851	Relatively Low
1325	124052	Glabridin	-6.603	-4.848	Relatively Low
1326	65243	Ginkgolide B	-5.206	-4.847	Relatively Low
1327	5317652	Glabrone	-6.522	-4.845	Relatively Low
1328	10639	Physcion	-6.969	-4.84	Relatively Low
1329	285033	Homoharringtonine	-4.801	-4.835	Relatively Low
1330	25252741	2'-O-methyl-Kurarinone	-6.752	-4.834	Relatively Low
1331	159999	Crebanine	-5.984	-4.831	Relatively Low
1332	73353637	N-(3-Methoxybenzyl)(9Z,12Z,15Z)-octadeca-9,12,15-trienamide	-5.336	-4.829	Relatively Low
1333	21589011	(-)-epicorynoxidine	-5.089	-4.827	Relatively Low
1334	6474309	4,5-Dicaffeoylquinic acid	-7.304	-4.826	Relatively Low
1335	736186	Isoferulic Acid	-4.909	-4.826	Relatively Low

1336	2355	Bergapten	-6.214	-4.825	Relatively Low
1337	234823	Eudesmin	-5.101	-4.825	Relatively Low
1338	10032468	Actein	-3.521	-4.824	Relatively Low
1339	6441391	Curdione	-4.537	-4.82	Relatively Low
1340	92044469	Ingenol-5,20-acetonide	-5.815	-4.82	Relatively Low
1341	124069	Dihydrosanguinarine	-5.275	-4.818	Relatively Low
1342	10235	Cytisine	-6.906	-4.816	Relatively Low
1343	23669322	Tanshinone IIA sodium sulfonate	-4.523	-4.814	Relatively Low
1344	9890994	Saikogenin D	-5.758	-4.814	Relatively Low
1345	5495925	Beta-mangostin	-5.386	-4.813	Relatively Low
1346	10988340	Alismoxide	-6.113	-4.809	Relatively Low
1347	9885603	Uncarine E	-4.011	-4.809	Relatively Low
1348	441747	Mesaconitine	-4.493	-4.808	Relatively Low
1349	10051937	Gymnemagenin	-4.451	-4.807	Relatively Low
1350	102410351	Lucidenic acid B	-3.549	-4.807	Relatively Low
1351	11061578	Prosapogenin A	-6.923	-4.805	Relatively Low
1352	1268142	Nootkatone	-5.725	-4.802	Relatively Low
1353	6917974	Bufogenin	-4.411	-4.8	Relatively Low
1354	471003	Ganoderic Acid B	-4.664	-4.8	Relatively Low
1355	102263760	3-Oxo-21α-methoxy-24,25,26,27-tetranortirucall-7-ene-23(21)-lactone	-5.085	-4.798	Relatively Low
1356	8955	Pregnenolone	-6.844	-4.798	Relatively Low

1357	5281617	Genkwanin	-6.3	-4.797	Relatively Low
1358	6223	Gliotoxin	-5.934	-4.792	Relatively Low
1359	5574924	Arglabin	-5.115	-4.791	Relatively Low
1360	20056103	Lucidenic acid C	-3.382	-4.79	Relatively Low
1361	10475115	Corynoxine	-4.935	-4.787	Relatively Low
1362	157539	Crassicauline A	-4.07	-4.787	Relatively Low
1363	434211	Oleaside A	-4.23	-4.786	Relatively Low
1364	5281304	Bruceantin	-4.472	-4.786	Relatively Low
1365	358901	Phyllanthin	-5.813	-4.786	Relatively Low
1366	10547386	Palmatrubine	-6.447	-4.783	Relatively Low
1367	73078	Tetrandrine	-4.317	-4.783	Relatively Low
1368	12302076	Brevilin A	-5.046	-4.782	Relatively Low
1369	102004721	25-methoxyalisol A	-3.179	-4.78	Relatively Low
1370	177014	Corynoline	-5.404	-4.779	Relatively Low
1371	358832	5-O-Demethylnobiletin	-7.047	-4.777	Relatively Low
1372	5281279	Lappaconitine	-5.787	-4.777	Relatively Low
1373	441740	Indaconitine	-4.563	-4.776	Relatively Low
1374	101595	Vomicine	-5.442	-4.775	Relatively Low
1375	23711819	Sodium Danshensu	-4.837	-4.771	Relatively Low
1376	91466	Matrine	-5.632	-4.77	Relatively Low
1377	73481	Fangchinoline	-4.572	-4.77	Relatively Low
1378	44563121	Kushenol A	-8.405	-4.768	Relatively Low
1379	10926754	Fargesin	-6.077	-4.767	Relatively Low
1380	9975641	Przewalskinic acid A	-4.827	-4.766	Relatively Low
1381	5281626		-6.09	-4.766	Relatively Low
1382	5281855	Elllagic Acid	-5.985	-4.764	Relatively Low

1383	260535	Vindoline	-4.955	-4.761	Relatively Low
1384	72344	Nobiletin	-5.902	-4.76	Relatively Low
1385	441893	Ruscogenin	-6.015	-4.759	Relatively Low
1386	439503	Salicin	-6.654	-4.759	Relatively Low
1387	14109375	Lucidenic acid A	-4.107	-4.755	Relatively Low
1388	10364	Carvacrol	-5.695	-4.752	Relatively Low
1389	88708	Gentiopicrin	-6.772	-4.749	Relatively Low
1390	443026	Veraguensin	-6.7	-4.749	Relatively Low
1391	11521428	Senkyunolide I	-5.866	-4.747	Relatively Low
1392	77547517	N-Benzyllinolenamide	-5.726	-4.744	Relatively Low
1393	479500	Isobutylshikonin	-6.604	-4.742	Relatively Low
1394	20055073	Worenine	-6.25	-4.741	Relatively Low
1395	7456	Methyl 4-hydroxybenzoate	-5.062	-4.739	Relatively Low
1396	72323	Jatrorrhizine	-5.739	-4.738	Relatively Low
1397	371256	Jatrorrhizine chloride	-5.739	-4.738	Relatively Low
1398	245005	Aconitine	-4.866	-4.737	Relatively Low
1399	3047739	Fraxinol	-5.574	-4.736	Relatively Low
1400	134606	Trigonelline Hydrochloride	-5.403	-4.733	Relatively Low
1401	75412551	20(R)-Ginsenoside Rg2	-6.209	-4.733	Relatively Low
1402	12305974	Periplocymarin	-4.747	-4.731	Relatively Low
1403	54580250	Morellic acid	-4.704	-4.731	Relatively Low
1404	107985	Triptolide	-5.114	-4.73	Relatively Low
1405	5283820	Hyodeoxycholic acid	-4.138	-4.729	Relatively Low

1406	6443046	Geissoschizine methyl ether	-4.89	-4.724	Relatively Low
1407	441826	Ajugasterone C	-5.666	-4.722	Relatively Low
1408	16220013	Tomatidine HCl	-5.491	-4.717	Relatively Low
1409	185605	(+)-Corypalmine	-5.643	-4.717	Relatively Low
1410	6442374	Furanodienon	-4.845	-4.715	Relatively Low
1411	5321317	Tetrahydroepiberberine	-6.73	-4.71	Relatively Low
1412	5321251	Senkyunolide H	-6.927	-4.71	Relatively Low
1413	38347607	Praeruptorin A	-4.86	-4.709	Relatively Low
1414	334704	Marmesin	-7.335	-4.708	Relatively Low
1415	10974362	27-Deoxyactein	-4.666	-4.704	Relatively Low
1416	5281783	Ethyl 4-methoxycinnamate	-5.16	-4.703	Relatively Low
1417	637110	O-Methylaloeresin A, 7-	-7.753	-4.7	Relatively Low
1418	259846	Lupeol	-3.655	-4.699	Relatively Low
1419	4871	Pomiferin	-6.058	-4.699	Relatively Low
1420	160006	Bruceine A	-4.332	-4.699	Relatively Low
1421	11290503	20-deoxyingenol	-5.655	-4.695	Relatively Low
1422	689043	Caffeic acid	-4.481	-4.695	Relatively Low
1423	3884	Lapachol	-6.586	-4.695	Relatively Low
1424	1254	Menthol	-5.244	-4.692	Relatively Low
1425	442009	Carnosol	-6.196	-4.688	Relatively Low
1426	73174	Dehydrocostus Lactone	-5.579	-4.688	Relatively Low
1427	71448947	Dahurinol	-4.907	-4.683	Relatively Low
1428	10574	Periplogenin	-5.1	-4.682	Relatively Low

1429	5317808	Gomisin O	-6.673	-4.68	Relatively Low
1430	1174	Uracil	-6.411	-4.677	Relatively Low
1431	20525	Asarylaldehyde	-5.706	-4.676	Relatively Low
1432	14078177	Schisandrone	-6.076	-4.673	Relatively Low
1433	5359389	Harmine hydrochloride	-5.651	-4.673	Relatively Low
1434	21592283	Lucidenic acid LM1	-4.204	-4.672	Relatively Low
1435	74787691	Dracorhodin perchlorate	-7.648	-4.668	Relatively Low
1436	159278	Salidroside	-6.819	-4.666	Relatively Low
1437	68827	Artemisinin	-5.261	-4.665	Relatively Low
1438	135394272	6-Methoxydihydrosangu inarine	-5.798	-4.664	Relatively Low
1439	5154	Sanguinarine	-5.859	-4.664	Relatively Low
1440	68635	Sanguinarium chloride	-5.859	-4.664	Relatively Low
1441	442985	Solasodine	-5.97	-4.664	Relatively Low
1442	5281781	Irisolidone	-5.75	-4.664	Relatively Low
1443	194000	Agrimol B	-6.882	-4.663	Relatively Low
1444	7067324	Andropanolide	-4.925	-4.661	Relatively Low
1445	10208	Chrysophanol	-6.945	-4.661	Relatively Low
1446	1738	Homovanillic acid	-5.379	-4.66	Relatively Low
1447	1110	Succinic acid	-3.869	-4.66	Relatively Low
1448	5316848	Angelicide	-5.611	-4.659	Relatively Low
1449	92043552	Dehydrocorydaline	-5.975	-4.659	Relatively Low
1450	638234	Thermopsine	-5.792	-4.658	Relatively Low
1451	259803	Gamabufotalin	-5.894	-4.657	Relatively Low
1452	21122581	Rosamultin	-5.813	-4.657	Relatively Low
1453	3037048	Isorhynchophylline	-5.056	-4.655	Relatively Low

1454	6770	Tetrahydrocoptisine	-6.993	-4.655	Relatively Low
1455	124062	Rubiadin	-5.703	-4.654	Relatively Low
1456	8768	Protocatechualdehyde	-5.931	-4.653	Relatively Low
1457	68486	Suberosin	-5.364	-4.65	Relatively Low
1458	929262	Sclareolide	-4.965	-4.646	Relatively Low
1459	78385214	Liquidambaric lactone	-3.939	-4.646	Relatively Low
1460	10177	Indirubin	-5.728	-4.646	Relatively Low
1461	64685	2-Borneol	-5.926	-4.644	Relatively Low
1462	6552009	(+)-Borneol	-5.238	-4.641	Relatively Low
1463	259776	Cinobufotalin	-4.514	-4.641	Relatively Low
1464	442424	Genipin	-5.736	-4.639	Relatively Low
1465	89406	Dihydroisotanshinone I	-7.056	-4.639	Relatively Low
1466	31211	Zingerone	-5.386	-4.638	Relatively Low
1467	182199	Arnicolide D	-5.059	-4.636	Relatively Low
1468	71765531	N-(3-Methoxybenzyl)palmitamide	-5.572	-4.635	Relatively Low
1469	13322806	Hydroxylongispinogenin, 23-	-5.026	-4.635	Relatively Low
1470	75130910	Gomisin D	-3.112	-4.634	Relatively Low
1471	9852086	Ginsenoside CK	-6.152	-4.634	Relatively Low
1472	441975	Tetrahydroalstonine	-4.703	-4.634	Relatively Low
1473	27924	Phorbol-12-Myristate-13-	-5.245	-4.633	Relatively Low
1474	5281600	Amentoflavone	-7.081	-4.632	Relatively Low

1475	13945489	Kamebakaurine	-4.421	-4.631	Relatively Low
1476	23669636	Sodium Ferulate	-4.455	-4.63	Relatively Low
1477	3000518	Dihydroartemisinin	-6.673	-4.629	Relatively Low
1478	5317238	Ethyl caffeate	-5.966	-4.626	Relatively Low
1479	134715246	Arenobufagin 3-hemisuberate	-4.5	-4.622	Relatively Low
1480	136419	7,4'-Di-O-methyldaidzein	-6.42	-4.621	Relatively Low
1481	115250	Taraxasterol	-3.792	-4.616	Relatively Low
1482	97226	10-Hydroxycamptothecin	-7.671	-4.616	Relatively Low
1483	1549095	Coniferyl alcohol	-5.822	-4.613	Relatively Low
1484	10856530	lepomerine	-6.222	-4.612	Relatively Low
1485	90474153	Handelin	-3.834	-4.611	Relatively Low
1486	3000322	Hyoscine	-5.374	-4.611	Relatively Low
1487	6603108	Scopolamine hydrobromide	-6.573	-4.611	Relatively Low
1488	134715183	Epimagnolin B	-5.878	-4.608	Relatively Low
1489	441828	25R-Inokosterone	-6.095	-4.606	Relatively Low
1490	101616676	28-demethyl -β-amyrone	-3.537	-4.606	Relatively Low
1491	3037448	Isocorynoxeine	-4.876	-4.606	Relatively Low
1492	5316262	Isoolivil	-5.615	-4.605	Relatively Low
1493	9859136	Eleutheroside C	-4.656	-4.603	Relatively Low
1494	3515	Guaiazulene	-6.534	-4.603	Relatively Low
1495	5317564	8-Geranopsoralen	-6.278	-4.601	Relatively Low

1496	10205	plumbapin	-5.957	-4.6	Relatively Low
1497	11953931	Voacamine	-4.896	-4.598	Relatively Low
1498	99615	Betulinaldehyde	-3.726	-4.595	Relatively Low
1499	5281756	Usaramine	-5.064	-4.592	Relatively Low
1500	10569	Abietic acid	-4.354	-4.587	Relatively Low
1501	11541511	Oleandrin	-4.471	-4.586	Relatively Low
1502	8369	Maltol	-5.317	-4.586	Relatively Low
1503	61361	Butylphthalide	-5.872	-4.585	Relatively Low
1504	91915	Sclareol glycol	-5.238	-4.583	Relatively Low
1505	439877	N-Caffeoylputrescine, (E)-	-5.459	-4.583	Relatively Low
1506	5471349	Bergamottin	-6.756	-4.582	Relatively Low
1507	91453	Hecogenin	-5.55	-4.581	Relatively Low
1508	5003667	Naringenin trimethyl ether	-6.315	-4.58	Relatively Low
1509	5281758	Beta-Asarone	-4.807	-4.579	Relatively Low
1510	644019	Cannabidiol	-4.722	-4.576	Relatively Low
1511	9942292	Osmundacetone	-5.031	-4.575	Relatively Low
1512	68289	Sesamol	-5.822	-4.574	Relatively Low
1513	5881	Dehydroepiandrosterone	-5.957	-4.573	Relatively Low
1514	73581	Bilobalide A	-5.729	-4.573	Relatively Low
1515	6047	Levodopa	-4.94	-4.572	Relatively Low
1516	9974762	Diosbulbin B	-4.635	-4.572	Relatively Low
1517	11724027	Guaiacin	-5.9	-4.572	Relatively Low
1518	21669994	Angelol A	-5.511	-4.569	Relatively Low
1519	6452086	Alpha Cyperone	-5.564	-4.565	Relatively Low

1520	44150282	Stachydrine hydrochloride	-4.89	-4.561	Relatively Low
1521	5281697	Scutellarein	-7.511	-4.56	Relatively Low
1522	20485	Tabersonine	-5.352	-4.558	Relatively Low
1523	442972	Cyclopamine	-4.703	-4.557	Relatively Low
1524	5284513	Acitretin	-5.847	-4.553	Relatively Low
1525	479501	Acetylshikonin	-6.606	-4.552	Relatively Low
1526	5420895	5-hydroxy-7-acetoxylsaccharidesavone	-5.571	-4.55	Relatively Low
1527	259991	Telocinobufagin	-5.057	-4.546	Relatively Low
1528	79050	1-Monomyristin	-5.251	-4.546	Relatively Low
1529	14036811	Alisol B Acetate	-4.031	-4.545	Relatively Low
1530	15120074	Seneciphyllinine	-5.991	-4.545	Relatively Low
1531	6442619	Seneciphylline N-Oxide	-5.559	-4.539	Relatively Low
1532	443028	Yangambin	-5.231	-4.534	Relatively Low
1533	7428	Methyl gallate	-5.475	-4.534	Relatively Low
1534	10228	Osthol	-5.751	-4.533	Relatively Low
1535	2734290	5-Methyl-7-methoxyisoflavone	-6.781	-4.528	Relatively Low
1536	10947	Muscone	-5.359	-4.527	Relatively Low
1537	73554029	Sinapine thiocyanate	-6.649	-4.525	Relatively Low
1538	5316132	Crocetine dimethyl ester	-5.383	-4.524	Relatively Low
1539	821449	Cnidilin	-6.091	-4.524	Relatively Low
1540	442127	Decursinol	-7.205	-4.523	Relatively Low

1541	2758	Eucalyptol	-4.574	-4.52	Relatively Low
1542	476856	meso-dihydroguaiaretic acid	-6.099	-4.519	Relatively Low
1543	5281120	Nervonic Acid	-4.684	-4.518	Relatively Low
1544	160921	Lysionotin	-6.378	-4.516	Relatively Low
1545	139291057	3-O-Acetyl-20-hydroxy ecdysone	-5.227	-4.516	Relatively Low
1546	98570	Allocryptopine	-4.981	-4.513	Relatively Low
1547	15558616	Alisol A	-5.015	-4.512	Relatively Low
1548	127	4-Hydroxyphenylacetic acid	-5.547	-4.511	Relatively Low
1549	6918670	Ingenol 3-Angelate	-4.624	-4.511	Relatively Low
1550	5316810	Dehydroeffusol	-6.999	-4.507	Relatively Low
1551	72303	Honokiol	-6.73	-4.505	Relatively Low
1552	441074	Quinidine	-5.11	-4.505	Relatively Low
1553	736681	Ferulic acid ethyl ester	-5.387	-4.503	Relatively Low
1554	65366	Baccatin III	-4.463	-4.502	Relatively Low
1555	91895364	Moluccanin	-6.607	-4.502	Relatively Low
1556	91503	Hydroquinidine	-5.896	-4.5	Relatively Low
1557	183466	3'-Demethylnobiletin	-6.174	-4.5	Relatively Low
1558	177015	Acetylcorynoline	-4.159	-4.499	Relatively Low
1559	876160	Isoeugenol acetate	-5.123	-4.497	Relatively Low
1560	14583584	β-Anhydroicaritin	-5.732	-4.492	Relatively Low
1561	479503	Shikonin	-6.978	-4.492	Relatively Low
1562	13909684	Chikusetsusaponin IVa	-5.174	-4.49	Relatively Low

1563	5319502	4-Methyl-6,7-dihydroxycoumarin	-6.327	-4.489	Relatively Low
1564	3080635	Atractylon	-5.95	-4.487	Relatively Low
1565	25256772	Protostemonine	-5.446	-4.487	Relatively Low
1566	10091424	Corynoxine B	-4.61	-4.485	Relatively Low
1567	479502	Beta-Hydroxylsovalerylshikonin	-5.852	-4.483	Relatively Low
1568	5471852	Poricoic acid B	-4.877	-4.482	Relatively Low
1569	7251185	Parthenolide	-5.151	-4.481	Relatively Low
1570	356759	Erianin	-5.601	-4.481	Relatively Low
1571	7118	Veratryl alcohol	-5.371	-4.48	Relatively Low
1572	276389	Harringtonine	-6.252	-4.48	Relatively Low
1573	10471963	glaucocalyxin A	-5.458	-4.479	Relatively Low
1574	19009	Palmatine	-5.085	-4.478	Relatively Low
1575	5281224	Astaxanthin	-6.007	-4.478	Relatively Low
1576	21120798	O-glucopyranosylepiederagenin, 28-	-4.752	-4.478	Relatively Low
1577	24721564	Denudatine	-5.287	-4.478	Relatively Low
1578	34781	Dehydrocorydalin	-5.603	-4.475	Relatively Low
1579	92043243	Dehydrocorydaline nitrate	-5.603	-4.475	Relatively Low
1580	34458	Tetrahydroberberine	-5.212	-4.475	Relatively Low
1581	21631105	Oxypaeoniflorin	-5.53	-4.474	Relatively Low

1582	2703	Chelerythrine	-6.5	-4.473	Relatively Low
1583	547	3,4-Dihydroxyphenylacetic acid	-5.67	-4.47	Relatively Low
1584	265237	Withaferin A	-5.582	-4.468	Relatively Low
1585	471002	Ganoderic Acid A	-4.603	-4.46	Relatively Low
1586	23915	Schizandrol A	-5.071	-4.456	Relatively Low
1587	91826818	Benzoylgomisin O	-5.109	-4.454	Relatively Low
1588	11992083	EGCG Octaacetate	-6.883	-4.454	Relatively Low
1589	154417	Hyoscyamine	-6.222	-4.454	Relatively Low
1590	11479437	Hyoscyamine sulfate hydrate	-6.196	-4.448	Relatively Low
1591	26204131	Angeloylgomisin H	-5.012	-4.447	Relatively Low
1592	75528891	Inulicin	-5.023	-4.446	Relatively Low
1593	243725	Dihydrorotenone	-5.655	-4.441	Relatively Low
1594	5317235	6-Ethoxysanguinarine	-4.739	-4.437	Relatively Low
1595	158477	Wilforlide A	-4.369	-4.437	Relatively Low
1596	119287	Cucurbitacin S	-4.047	-4.433	Relatively Low
1597	6450191	Linderalactone	-5.233	-4.431	Relatively Low
1598	11969542	Cinobufagin	-4.443	-4.431	Relatively Low
1599	108168	Aristolochic Acid B	-5.884	-4.43	Relatively Low
1600	24868376	7-beta-Hydroxylathyrol	-5.077	-4.429	Relatively Low
1601	90472238	Ginsenoside RK2	-4.436	-4.421	Relatively Low
1602	185481	Evodol	-4.65	-4.419	Relatively Low
1603	9547215	Bufalin	-5.096	-4.417	Relatively Low
1604	5459840	Beta-Ecdysterone	-4.872	-4.416	Relatively Low

1605	15541911	Cimigenol-3-O-alpha-L-arabinoside	-5.891	-4.415	Relatively Low
1606	44258296	Kuwanon A	-6.56	-4.414	Relatively Low
1607	260439	Cyclovirobuxine D	-5.746	-4.413	Relatively Low
1608	10043694	Cnidicin	-6.284	-4.413	Relatively Low
1609	73659	Maslinic acid	-3.716	-4.411	Relatively Low
1610	91864462	Angeloylgomisin O	-4.603	-4.405	Relatively Low
1611	72307	Sesamin	-5.879	-4.398	Relatively Low
1612	102212087	Lancifodilactone F	-5.215	-4.397	Relatively Low
1613	138112783	Gomisin H	-5.436	-4.391	Relatively Low
1614	5317228	6-Ethoxychelerthrine	-5.732	-4.39	Relatively Low
1615	1023495	Dihydropalmatine	-5.422	-4.389	Relatively Low
1616	13934282	Ganoderal A	-4.48	-4.384	Relatively Low
1617	14057196	4',7-Di-O-methylnaringenin	-6.815	-4.383	Relatively Low
1618	64945	Ursolic acid	-5.183	-4.383	Relatively Low
1619	100780	Nepodin	-5.473	-4.383	Relatively Low
1620	38347601	DL-Praeruptorin A	-5.604	-4.382	Relatively Low
1621	5280385	Sinapine	-5.93	-4.382	Relatively Low
1622	93009	Bornyl acetate	-4.549	-4.38	Relatively Low
1623	16020000	Cimigenol	-4.248	-4.377	Relatively Low
1624	15376	Vincamine	-6.125	-4.375	Relatively Low
1625	72321	Coptisine Chloride	-7.433	-4.369	Relatively Low
1626	72322	Coptisine sulfate	-7.433	-4.369	Relatively Low
1627	441813	Bryodulcosigenin	-4.548	-4.367	Relatively Low
1628	13968328	Regelidine	-6.765	-4.366	Relatively Low

1629	163263	Sclareol	-5.15	-4.365	Relatively Low
1630	247	Betaine	-4.553	-4.364	Relatively Low
1631	11545	Betaine hydrochloride	-4.553	-4.364	Relatively Low
1632	637542	p-Coumaric acid	-3.889	-4.363	Relatively Low
1633	642376	3-Butylidenephthalide	-6.284	-4.363	Relatively Low
1634	99516	Tigogenin	-5.53	-4.361	Relatively Low
1635	73294	Ganodermanondiol	-4.959	-4.36	Relatively Low
1636	119093	Cynaropicrin	-5.308	-4.359	Relatively Low
1637	9877482	Norcantharidin	-5.883	-4.357	Relatively Low
1638	10074228	Flemiphilippinin A	-5.21	-4.353	Relatively Low
1639	73432	Brusatol	-4.203	-4.353	Relatively Low
1640	188289	Columbin	-5.907	-4.351	Relatively Low
1641	6918114	11-Keto-beta-boswellic acid	-3.103	-4.349	Relatively Low
1642	10248	Elemicin	-4.871	-4.348	Relatively Low
1643	596894	Kakuol	-5.768	-4.348	Relatively Low
1644	10466307	Loganetin	-5.351	-4.342	Relatively Low
1645	148670	13-methylberberine	-5.242	-4.34	Relatively Low
1646	418033	Dihydrowithaferin A	-5.041	-4.336	Relatively Low
1647	179651	Limonin	-4.475	-4.335	Relatively Low
1648	102004923	1-O-acetylbritannilacton e	-5.283	-4.33	Relatively Low
1649	9848024	Neoandrographolide	-5.612	-4.33	Relatively Low
1650	6287	L-Valine	-4.453	-4.33	Relatively Low
1651	440080	1-Kestose	-5.314	-4.329	Relatively Low
1652	11624161	14-Deoxyandrographoli	-5.247	-4.328	Relatively Low

		de			
1653	92785	Taraxerone	-3.757	-4.324	Relatively Low
1654	20056131	Maoecrystal A	-5.63	-4.316	Relatively Low
1655	92231	Spathulenol	-5.033	-4.315	Relatively Low
1656	12300142	Bisabolangelone	-6.819	-4.308	Relatively Low
1657	354616	Gentiannine	-5.391	-4.306	Relatively Low
1658	237332	5-Hydroxymethylfurfura l	-4.884	-4.305	Relatively Low
1659	467319	Epitheaflagallin 3-O-gallate	-7.572	-4.303	Relatively Low
1660	145742	L-Proline	-5.256	-4.301	Relatively Low
1661	46783795	Nomilin	-4.57	-4.3	Relatively Low
1662	14704104	Hydroxyphenethylanisat e, 4-	-6.253	-4.299	Relatively Low
1663	90453	Cinchonine hydrochloride	-6.735	-4.298	Relatively Low
1664	6540717	Dehydroandrographolid e succinate	-3.504	-4.297	Relatively Low
1665	23685777	Dehydroandrographolid e Succinate Potasium Salt	-3.504	-4.297	Relatively Low
1666	65411	Triptonide	-4.943	-4.295	Relatively Low
1667	21598997	3-Deoxyaconitine	-3.55	-4.295	Relatively Low
1668	21679042	Deoxyandrographolide	-5.298	-4.294	Relatively Low
1669	637775	Sinapic acid	-4.873	-4.292	Relatively Low

1670	634470	Schizandrol B	-5.481	-4.289	Relatively Low
1671	98914	Deoxyshikonin	-6.832	-4.286	Relatively Low
1672	72300	Magnolol	-5.961	-4.286	Relatively Low
1673	124039	Fraxinellone	-5.849	-4.284	Relatively Low
1674	14656910	Arnicolide C	-4.653	-4.277	Relatively Low
1675	168114	8-Gingerol	-5.721	-4.276	Relatively Low
1676	122797	4'-Demethylepipodophyllotoxin	-5.692	-4.275	Relatively Low
1677	71307574	Schizantherin B	-4.505	-4.273	Relatively Low
1678	72301	L-Tetrahydropalmatine	-5.555	-4.273	Relatively Low
1679	91510	Maackiain	-6.126	-4.271	Relatively Low
1680	185786	Ergolide	-5.671	-4.27	Relatively Low
1681	6475945	Demethylpseudolaric acid B	-4.482	-4.26	Relatively Low
1682	71300866	Bullatine A	-4.373	-4.259	Relatively Low
1683	21599000	3-Acetylaconitine	-4.094	-4.257	Relatively Low
1684	1023768	Hypocrellin B	-6.801	-4.256	Relatively Low
1685	773630	Piperlotine A	-6.655	-4.254	Relatively Low
1686	54695756	Pogostone	-5.032	-4.254	Relatively Low
1687	442126	Decursin	-5.541	-4.252	Relatively Low
1688	3085257	Senkyunolide A	-5.234	-4.252	Relatively Low
1689	629964	6-Demethoxytangeretin	-6.062	-4.246	Relatively Low
1690	92097	Taraxerol	-3.881	-4.246	Relatively Low
1691	8434	Ethylparaben	-4.858	-4.246	Relatively Low
1692	5319022	Z-Ligustilide	-5.95	-4.245	Relatively Low
1693	14193399	Glaucocalyxin B	-4.643	-4.239	Relatively Low

1694	6918774	Corosolic acid	-3.38	-4.238	Relatively Low
1695	5319081	Loureirin A	-6.632	-4.237	Relatively Low
1696	70698035	Levistilide A	-5.412	-4.235	Relatively Low
1697	12314399	Platycodigenin	-3.202	-4.23	Relatively Low
1698	161954	Jolkinolide B	-4.327	-4.219	Relatively Low
1699	6852391	Scopolamine butylbromide	-6.057	-4.217	Relatively Low
1700	71773126	Ziyuglycoside II	-4.641	-4.21	Relatively Low
1701	21648	Raspberry Ketone	-5.142	-4.209	Relatively Low
1702	11066	Berlambine	-5.548	-4.208	Relatively Low
1703	53232	Lovastatin	-5.225	-4.208	Relatively Low
1704	16666	L-Menthol	-5.316	-4.206	Relatively Low
1705	6251	D-Mannitol	-4.11	-4.203	Relatively Low
1706	9934504	20-O-Acetylingenol-3-angelate	-4.684	-4.2	Relatively Low
1707	473252	Isomeranzin	-5.95	-4.2	Relatively Low
1708	119307	Ginsenoside Rh2	-4.511	-4.197	Relatively Low
1709	72521	Alkannin	-7.14	-4.197	Relatively Low
1710	441727	Delsoline	-5.928	-4.189	Relatively Low
1711	6543478	Arteannuin B	-5.589	-4.182	Relatively Low
1712	5273621	Demethoxyyangonin	-6.206	-4.181	Relatively Low
1713	24721223	Lathyrol	-5.476	-4.181	Relatively Low
1714	442827	Trifolirhizin	-5.847	-4.18	Relatively Low
1715	485077	Dihydrochelerythrine	-5.679	-4.177	Relatively Low
1716	3001686	Gomisin J	-5.436	-4.176	Relatively Low
1717	6433206	Sulforaphene	-3.88	-4.175	Relatively Low

1718	10098	Jervine	-4.636	-4.175	Relatively Low
1719	154272	10-deacetylbaccatin III	-4.416	-4.173	Relatively Low
1720	10287099	Disodium trans-crocetinate	-4.787	-4.172	Relatively Low
1721	2353	Berberine	-5.522	-4.16	Relatively Low
1722	12456	Berberine hydrochloride	-5.522	-4.16	Relatively Low
1723	12457	Berberine Sulfate	-5.522	-4.16	Relatively Low
1724	11969465	Marinobufagin	-4.483	-4.155	Relatively Low
1725	3083590	Phillygenin	-6.3	-4.147	Relatively Low
1726	3084708	groenlandicine	-6.791	-4.147	Relatively Low
1727	68313	Hordenine	-5.869	-4.146	Relatively Low
1728	101301	Corydaline	-6.354	-4.146	Relatively Low
1729	99474	Diosgenin	-5.937	-4.144	Relatively Low
1730	10607	Podophyllotoxin	-6.254	-4.137	Relatively Low
1731	10001602	Isoforskolin	-5.855	-4.136	Relatively Low
1732	14992067	Gomisin G	-5.602	-4.135	Relatively Low
1733	68742556	(9Z,12Z)-N-Benzylocta deca- 9,12-dienamide	-6.282	-4.134	Relatively Low
1734	10394	Phloretic acid	-5.047	-4.128	Relatively Low
1735	6438196	Bacdanol	-4.581	-4.125	Relatively Low
1736	11090206	Tokinolide B	-4.253	-4.125	Relatively Low
1737	133504	Schinifoline	-6.097	-4.122	Relatively Low
1738	89594	L-Nicotine	-4.966	-4.118	Relatively Low

1739	6267	L-Asparagine	-4.503	-4.116	Relatively Low
1740	444679	Ergosterol	-5.447	-4.113	Relatively Low
1741	345501	Deoxypodophyllotoxin	-6.256	-4.11	Relatively Low
1742	12442765	Polygalic Acid	-3.391	-4.109	Relatively Low
1743	78577438	Dehydroandrographolide	-5.796	-4.107	Relatively Low
1744	155011	Aurantioobtusin	-5.317	-4.106	Relatively Low
1745	108130	Schisandrin B	-6.312	-4.104	Relatively Low
1746	821366	L-Sinoacutine	-6.138	-4.098	Relatively Low
1747	442495	Pulegone	-5.961	-4.092	Relatively Low
1748	2236	Aristolochic acid A	-5.809	-4.09	Relatively Low
1749	11468733	(20S)-Protopanaxatriol	-4.681	-4.089	Relatively Low
1750	864	α-Lipoic acid	-4.68	-4.084	Relatively Low
1751	10114	Glycyrrhetinic acid	-3.337	-4.08	Relatively Low
1752	76314443	Alisol G	-4.209	-4.078	Relatively Low
1753	6475946	demethoxydeacetoxypseudolaric acid B	-4.395	-4.072	Relatively Low
1754	5280794	Stigmasterol	-5.09	-4.069	Relatively Low
1755	12118082	Monascin	-6.897	-4.066	Relatively Low
1756	443955	Vinpocetine	-5.714	-4.065	Relatively Low
1757	73401	Marrubiin	-5.812	-4.063	Relatively Low
1758	5315892	Cinnamyl Alcohol	-4.98	-4.063	Relatively Low
1759	11869658	3-Epioleanolic acid	-4.515	-4.05	Relatively Low
1760	5320113	Danshenxinkun B	-5.898	-4.049	Relatively Low
1761	168136	Nardosinone	-4.352	-4.047	Relatively Low

1762	101671038	Hypaconine	-5.132	-4.043	Relatively Low
1763	82755	Hydroxytyrosol	-5.433	-4.039	Relatively Low
1764	101810	Quillaic acid	-4.361	-4.039	Relatively Low
1765	220495	N-Benzylstearamide	-4.547	-4.038	Relatively Low
1766	68316	Perillene	-4.312	-4.034	Relatively Low
1767	9817839	Dehydroevodiamine	-6.892	-4.029	Relatively Low
1768	135393457	Dehydroevodiamine hydrochloride	-6.892	-4.029	Relatively Low
1769	102004667	Incensole	-4.199	-4.028	Relatively Low
1770	124928704	O-Tigloylgymnemagenin, 21-	-5.932	-4.024	Relatively Low
1771	7127	Methyleugenol	-4.769	-4.021	Relatively Low
1772	14466541	Britannin	-6.147	-4.009	Relatively Low
1773	73412	Madecassic acid	-4.343	-4.007	Relatively Low
1774	11725801	Sauchinone	-4.829	-4.006	Relatively Low
1775	158103	Gomisin N	-5.009	-4.004	Relatively Low
1776	12315259	Santalol	-5.264	-4.001	Relatively Low
1777	155256	Schisandrin A	-6.267	-3.995	Relatively Low
1778	76310822	Alisol F	-5.705	-3.995	Relatively Low
1779	5470187	Zerumbone	-5.189	-3.989	Relatively Low
1780	142768	etin dimetyl ether?	-5.586	-3.988	Relatively Low
1781	5281650	Alpha-Mangostin	-5.511	-3.987	Relatively Low
1782	431129	Asatone	-4.095	-3.985	Relatively Low
1783	91472	Friedelin	-3.652	-3.985	Relatively Low
1784	125	4-Hydroxybenzyl alcohol	-6.055	-3.978	Relatively Low

1785	6434600	Isopropyl ferulate	-5.279	-3.972	Relatively Low
1786	3032313	Epigoitrin	-6.444	-3.972	Relatively Low
1787	9301	Arecoline hydrobromide	-5.172	-3.969	Relatively Low
1788	7163177	3-Epiursolic acid	-3.997	-3.968	Relatively Low
1789	72311	Chelerythrine chloride	-6.251	-3.964	Relatively Low
1790	88311768	β-Crocetin	-5.023	-3.961	Relatively Low
1791	23247892	Lucidenic Acid E2	-4.104	-3.952	Relatively Low
1792	73309	Echinocystic acid	-3.802	-3.951	Relatively Low
1793	3503	Gossypol	-4.739	-3.941	Relatively Low
1794	6440704	Pseudolaric Acid C	-3.698	-3.935	Relatively Low
1795	13943286	Cycloastragenol	-4.293	-3.929	Relatively Low
1796	4947	Propyl gallate	-5.202	-3.921	Relatively Low
1797	444539	Cinnamic acid	-4.182	-3.918	Relatively Low
1798	71522011	Valechlorine	-5.54	-3.918	Relatively Low
1799	235307	Obtucarbamate A	-5.475	-3.908	Relatively Low
1800	442720	Dimethylacrylalkannin, β,β-	-6.321	-3.905	Relatively Low
1801	13889352	Taraxasteryl acetate	-4.018	-3.904	Relatively Low
1802	5281425	Pteryxin	-5.933	-3.902	Relatively Low
1803	5281331	Alpha-Spinasterol	-5.911	-3.898	Relatively Low
1804	10367978	Heteroclitin D	-3.228	-3.895	Relatively Low
1805	12305221	Bayogenin	-3.032	-3.887	Relatively Low
1806	227456	Acetate gossypol	-4.605	-3.884	Relatively Low
1807	3314	Eugenol	-4.567	-3.881	Relatively Low
1808	6436348	Germacrone	-4.748	-3.877	Relatively Low

1809	59054177	Blinin	-6.09	-3.877	Relatively Low
1810	155240	Hydroxytyrosol Acetate	-4.821	-3.875	Relatively Low
1811	73057	Schisanhenol	-5.829	-3.875	Relatively Low
1812	21672692	Anemosapogenin	-3.344	-3.875	Relatively Low
1813	15940183	Euphorbiasteroid	-2.48	-3.859	Relatively Low
1814	47936	Forskolin	-5.352	-3.841	Relatively Low
1815	51666243	Angelol K	-6.198	-3.841	Relatively Low
1816	173183	Campesterol	-3.829	-3.831	Relatively Low
1817	5469634	Ginkgolic Acid C17-1	-5.683	-3.82	Relatively Low
1818	46173999	Clemaphenol A	-5.861	-3.816	Relatively Low
1819	101749	Galgravin	-6.849	-3.809	Relatively Low
1820	9847853	(20R)-Protopanaxatriol	-3.954	-3.805	Relatively Low
1821	10322911	Demethylzeylasteral	-5.142	-3.792	Relatively Low
1822	119242	Epifriedelanol	-4.954	-3.791	Relatively Low
1823	21709636	1-Methyl-2-pentyl-4(1H)-quinolinone	-6.464	-3.783	Relatively Low
1824	5319964	Murrayone	-5.311	-3.779	Relatively Low
1825	9750	L-Citrulline	-4.027	-3.774	Relatively Low
1826	119	Gamma-Aminobutyric acid	-4.693	-3.773	Relatively Low
1827	6760	Skimmianine	-6.354	-3.77	Relatively Low
1828	14240392	Curcumol	-5.359	-3.769	Relatively Low
1829	71463992	Eupalinolide B	-4.34	-3.769	Relatively Low
1830	31234	Hydrocinnamyl alcohol	-4.476	-3.762	Relatively Low

1831	10175	Cotoin	-5.985	-3.757	Relatively Low
1832	10399139	Isocurcumenol	-5.054	-3.741	Relatively Low
1833	443027	Schisandrin C	-5.256	-3.734	Relatively Low
1834	124222343	Neoprzewaquinone A	-4.33	-3.727	Relatively Low
1835	6288	L-Threonine	-4.093	-3.722	Relatively Low
1836	2773624	4-Hydroxyisoleucine	-4.407	-3.715	Relatively Low
1837	12315075	Rotundic acid	-3.229	-3.71	Relatively Low
1838	471426	Euscaphic acid	-3.211	-3.7	Relatively Low
1839	10404245	Anwuligan	-6.678	-3.698	Relatively Low
1840	9890209	Ursonic acid	-3.778	-3.698	Relatively Low
1841	7184	Butyl 4-Hydroxybenzoate	-5.256	-3.695	Relatively Low
1842	12315507	Shionone	-3.23	-3.694	Relatively Low
1843	439378	Theanine	-3.846	-3.689	Relatively Low
1844	74990	Irinotecan Hydrochloride	-4.342	-3.689	Relatively Low
1845	115012	Soyasapogenol B	-3.631	-3.688	Relatively Low
1846	87310	Alliin	-3.55	-3.68	Relatively Low
1847	167812	Curcumenol	-5.378	-3.679	Relatively Low
1848	98608	Phellopterin	-6.1	-3.676	Relatively Low
1849	10577938	5,15-Diacetyl-3-benzoyllathyrol	-2.733	-3.674	Relatively Low
1850	10955174	Patchouli alcohol	-4.213	-3.662	Relatively Low
1851	92802	OLEAN-12-ENE-3B,28-DIOL	-3.725	-3.66	Relatively Low
1852	15560302	Gymnestrogenin	-4.897	-3.657	Relatively Low

1853	2998	Nonivamide	-3.822	-3.656	Relatively Low
1854	6442612	10-Shogaol	-5.638	-3.656	Relatively Low
1855	11850	Dulcitol	-3.969	-3.655	Relatively Low
1856	71307581	Tussilagone	-4.935	-3.649	Relatively Low
1857	5321047	Atractylodin	-4.219	-3.618	Relatively Low
1858	7991	N-Valeric acid	-3.713	-3.61	Relatively Low
1859	9805290	Polyporenic acid C	-5.06	-3.607	Relatively Low
1860	20055812	Chasmanine	-4.207	-3.605	Relatively Low
1861	445724	L-Fucitol	-4.134	-3.602	Relatively Low
1862	70775	2-hydroxychavicol	-4.256	-3.6	Relatively Low
1863	11198769	Macamide B	-6.607	-3.598	Relatively Low
1864	222284	Beta-Sitosterol	-4.919	-3.591	Relatively Low
1865	12442762	Senegenin	-3.041	-3.585	Relatively Low
1866	23462	L-Cysteine hydrochloride monohydrate	-4.177	-3.571	Relatively Low
1867	637511	Cinnamaldehyde	-4.294	-3.568	Relatively Low
1868	64971	Betulinic acid	-3.032	-3.562	Relatively Low
1869	10393	Tyrosol	-5.081	-3.551	Relatively Low
1870	26248	Diacerein	-6.036	-3.549	Relatively Low
1871	10687292	Cytosporone B	-6.277	-3.548	Relatively Low
1872	131676044	Euphorbia factor L1	-3.9	-3.531	Relatively Low
1873	73299	Hederagenin	-3.316	-3.531	Relatively Low
1874	161388	Polygalacic acid	-3.786	-3.526	Relatively Low
1875	13250	Gallic Acid Ethyl Ester	-5.096	-3.513	Relatively Low

1876	25717254	Praeruptorin B	-4.7	-3.499	Relatively Low
1877	161464	Leonurine	-4.858	-3.498	Relatively Low
1878	46837042	Leonurine Hydrochloride	-4.858	-3.498	Relatively Low
1879	517326	Sodium dichloroacetate	-3.108	-3.488	Relatively Low
1880	3516	Guaifenesin	-4.993	-3.483	Relatively Low
1881	73755086	Incensole acetate	-4.116	-3.479	Relatively Low
1882	92158	Lupenone	-3.33	-3.468	Relatively Low
1883	12442849	Soyasapogenol A	-3.86	-3.467	Relatively Low
1884	776123	Decursinol angelate	-5.756	-3.453	Relatively Low
1885	12315005	Roburic Acid	-4.092	-3.449	Relatively Low
1886	119034	Asiatic acid	-4.543	-3.446	Relatively Low
1887	14296	Chuanxingzine	-4.286	-3.442	Relatively Low
1888	156709	Ligustrazine Hydrochloride	-4.286	-3.442	Relatively Low
1889	5962	L-Lysine	-4.125	-3.439	Relatively Low
1890	73346080	N-(3-Methoxybenzyl)oleamide	-5.255	-3.438	Relatively Low
1891	11213350	20(S)-Protopanaxadiol	-4.688	-3.438	Relatively Low
1892	4133	Methyl salicylate	-5.196	-3.426	Relatively Low
1893	9958136	3-O-Acetyl-16alpha-hydroxytrametenolic acid	-4.393	-3.421	Relatively Low
1894	159516	Pristimerin	-4.859	-3.419	Relatively Low
1895	5281858	Ginkgolic Acid C15-1	-5.491	-3.417	Relatively Low

1896	441678	Euphol	-3.88	-3.414	Relatively Low
1897	53343513	Protostemotinine	-4.552	-3.411	Relatively Low
1898	161800	Glycyrrhetic Acid 3-O-β-D- Glucuronide	-4.213	-3.41	Relatively Low
1899	151202	3-O-acetyloleanolic acid	-3.597	-3.393	Relatively Low
1900	9920281	20(R)-Protopanaxadiol	-4.88	-3.389	Relatively Low
1901	123976	27-Hydroxycholesterol	-6.521	-3.385	Relatively Low
1902	6475119	3-acetylursolic acid	-4.247	-3.376	Relatively Low
1903	125468	Tiglic acid	-3.172	-3.357	Relatively Low
1904	10359753	Daidzein Diacetate	-3.589	-3.347	Relatively Low
1905	5281232	Crocetin	-4.165	-3.345	Relatively Low
1906	5320692	Praeruptorin C	-5.714	-3.337	Relatively Low
1907	6440581	Praeruptorin E	-5.506	-3.33	Relatively Low
1908	157081	Ingenol-3,4-5,20-diacetonide	-3.562	-3.322	Relatively Low
1909	72326	Betulin	-4.014	-3.321	Relatively Low
1910	107982	Dihydrocapsaicin	-5.81	-3.308	Relatively Low
1911	3083352	13-Acetyl-9-dihydrobaccatin	-2.108	-3.302	Relatively Low
1912	11148	Glycerol tritetradecanoate	-5.316	-3.288	Relatively Low
1913	12313704	Oleanonic acid	-4.174	-3.282	Relatively Low
1914	442436	valepotriate	-6.195	-3.273	Relatively Low
1915	182140	Hypophyllanthin	-4.976	-3.262	Relatively Low
1916	14219	1,1-Dimethylbiguanide	-3.313	-3.262	Relatively Low

		hydrochloride			
1917	12444386	Arjungenin	-4.272	-3.261	Relatively Low
1918	73193	Tormentic acid	-3.499	-3.251	Relatively Low
1919	10922465	Artemisic acid	-4.477	-3.25	Relatively Low
1920	65048	Medicagenic acid	-3.673	-3.23	Relatively Low
1921	485711	3-Epibetulinic acid	-3.109	-3.22	Relatively Low
1922	442793	6-Gingerol	-3.7	-3.215	Relatively Low
1923	101761	Erythrodiol	-5.242	-3.194	Relatively Low
1924	94225	Taraxerol acetate	-3.251	-3.183	Relatively Low
1925	15559100	Beta-Elemonic acid	-3.806	-3.164	Relatively Low
1926	56833075	3-Hydroxybakuchiol	-4.979	-3.142	Relatively Low
1927	21594228	Phytolaccagenin	-3.446	-3.138	Relatively Low
1928	92044470	Ingenol-5,20-acetonide-3-O-angelate	-4.406	-3.125	Relatively Low
1929	6442560	8-Shogaol	-5.512	-3.102	Relatively Low
1930	10819	Perilla alcohol	-5.84	-3.084	Relatively Low
1931	122844	Liquidambaric acid	-3.21	-3.084	Relatively Low
1932	9577379	L-Sulforaphane	-3.583	-3.077	Relatively Low
1933	643915	Angelic acid	-3.119	-3.075	Relatively Low
1934	168836	Nordihydrocapsaicin	-4.321	-3.067	Relatively Low
1935	38359583	Isodeoxyelephantopin	-6.147	-3.051	Relatively Low
1936	572766	Curzerene	-5.48	-3.047	Relatively Low
1937	6137	L-Methionine	-3.738	-3.045	Relatively Low
1938	6436278	Pseudolaric Acid A	-4.677	-3.031	Relatively Low

1939	6549	Linalool	-3.88	-3.025	Relatively Low
1940	441728	deltaline	-3.939	-3.018	Relatively Low
1941	184492	7-Epitaxol	-6.213	-3.007	Relatively Low
1942	11168203	3-Acetyl-11-keto-beta-boswellic Acid	-3.913	-2.985	Relatively Low
1943	44630058	Angelic anhydride	-3.915	-2.954	Relatively Low
1944	68911	Artemether	-4.871	-2.92	Relatively Low
1945	134714896	Isoasatone A	-4.602	-2.919	Relatively Low
1946	122724	Celastrol	-4.755	-2.91	Relatively Low
1947	68406	1-Octacosanol	-3.019	-2.864	Relatively Low
1948	161306	Ginkgolic Acid C13-0	-5.679	-2.861	Relatively Low
1949	155245	β-acetoxyisovalerylalkannin	-6.493	-2.855	Relatively Low
1950	601100	Wilforine	-5.694	-2.852	Relatively Low
1951	5468522	Bakuchiol	-4.312	-2.806	Relatively Low
1952	5280489	Carotene	-4.69	-2.794	Relatively Low
1953	637566	Geraniol	-4.16	-2.79	Relatively Low
1954	6322	L-Arginine	-3.374	-2.751	Relatively Low
1955	46870578	Hydroxy-ε-sanshool	-3.252	-2.708	Relatively Low
1956	10220912	Hydroxy-β-sanshool	-3.741	-2.68	Relatively Low
1957	14135317	Hydroxy-γ-sanshool	-1.972	-2.573	Relatively Low
1958	5281794	6-Shogaol	-4.355	-2.558	Relatively Low
1959	5281326	Fucosterol	-5.026	-2.42	Relatively Low
1960	8294	Linalyl Acetate	-4.085	-2.381	Relatively Low
1961	68972	Triacontanol	-3.621	-2.303	Relatively Low
1962	3218	Embelin	-2.522	-2.289	Relatively Low

1963	23663544	Sodium houttuyfonate	-2.223	-2.229	Relatively Low
1964	5283263	Falcarindiol	-3.049	-2.201	Relatively Low
1965	12409	Nonacosane	-2.466	-2.199	Relatively Low
1966	61253	Octyl gallate	-4.373	-2.129	Relatively Low
1967	10084135	Hydroxy-α-sanshool	-3.592	-2.009	Relatively Low
1968	5962587	AcetylResveratrol	-3.675	-1.955	Relatively Low
1969	5284507	Nerolidol	-2.227	-1.238	Relatively Low
1970	643684	Ricinoleic acid	-2.236	-1.092	Relatively Low
1971	9940690	Crocin II	-6.19	-1.014	Relatively Low
1972	126312	Panaxydol	-2.824	-0.989	Relatively Low
1973	71307573	Pseudolaric Acid B	-4.488	-0.861	Relatively Low
1974	5469789	Panaxynol	-1.434	-0.429	Relatively Low
1975	985	Palmitic acid	-0.582	0.695	Relatively Low
1976	11005	Myristic acid	-0.258	0.71	Relatively Low
1977	8164	Octyl acetate	0.37	1.551	Relatively Low
1978	5312738	Royal jelly acid	0.939	1.738	Relatively Low

We then extended to one TCM natural compounds library to exploiting other unlisted potential ingredients and herbs. 1971 ingredients with the valid docking terminally among 2042 compounds, whose PubChem CID were attached in the side. All docking results, besides seven positive controls, were showed in glide gscore (the lower, the better). In addition, the notes were attached here for indicating the results contrasted with the best control (Ritonavir to 6VSB: -7.828 kcal/mol while Remdesivir to 6LU7: -8.738 kcal/mol). This included High in 6VSB (< -7.828 kcal/mol) / 6LU7 (< -8.738 kcal/mol), High in both (high efficiency in both), Relatively Low (lower efficiency in the both), Control, The Best Control within 6VSB and The Best Control within 6LU7.

Table S12. The best docking results with the both (6LU7 & 6VSB) among all compounds above.

NO.	Compounds CID	Compounds Name	Docking gscore (kcal/mol) 6LU7#	Docking gscore (kcal/mol) 6VSB#	Source Herbs (Abbreviation (Latin Name, Chinese Pinyin))
1	44258007	Madreselvin B	-9.017	-8.588	JYH (Lonicerae Japonicae Flos, Jin Yin Hua)
2	123339619	5-MethoxyPinocembroside	-9.359	-10.791	GHC (*Penthorum chinense* Pursh, Gan Huang Cao)
3	65238	1,2,3,4,6-Pentagalloylglucose	-8.927	-8.889	WBZ (*Rhus chinensis* Mill., Wu Bei Zi)
4	11664897	Kaempferol 3-O-β-D-(6"-p- coumaroyl) glucopyranosyl (1-2)-α-L-rhamnopyranoside	-11.316	-8.732	YX (Folium Ginkgo, Yin Xing)

Here we collected the most potential compounds targeting SARS-CoV-2 from the two docking results (8 herbs and 2042 natural compounds), which presented better to the both core structures (6LU7 and 6VSB) compared with controls.

Table S13. The occupation of SSMs' (symptoms, syndromes and medical conditions) targets within the entire targets database we discussed.

SSMs	Incorporated Genes	Excluded Genes	Occupations (%)
SARS	38	48	44.19
Lymphopenia/Decreased Lymphocyte	318	340	48.33
COVID-19	172	176	49.43
Diarrhea	76	76	50.00
Pyrexia/Fever	159	153	50.96
Pneumonia	110	102	51.89
Myalgia/Musle Soreness	165	150	52.38
Mucus/Phlegm/Suptum	124	106	53.91
Dyspnea	52	35	59.77
Asthenia/Fatigue	68	45	60.18
Cytokine storm	23	14	62.16
Coughing	32	14	69.57
Nausea/Vomiting	58	23	71.60
Oxygen Saturation	41	16	71.93
**Average**			56.88

The molecular targets of major SSMs for COVID-19 were obtained from NCBI and Genecards database. COVID-19 was u s e d the same as Fig.3. The targets with correlation score ≥ 20 in Genecards and all in NCBI targets were combined after TCMSP filter and duplicates removal, which were shown in the "Incorporated Genes". And "Excluded Genes" indicated the actual eliminates of TCMSP filter. The occupations were calculated from the amounts of incorporated genes divided by the amounts of uneliminated data (incorporated genes add to excluded genes). Here we can see that over 50% were

incorporated in TCMSP largely and the average was 56.88% signed at the bottom, which means a certain targets of SSMs would left after constraining with the TCMSP datbase. And this data was pictured in Fig 5A.

Table S14. Coherence analysis between TCM remedies and COVID-19 related SSMs.

Recipes	No. of intersection	COVID-19 （172）	No. of intersection	pneumonia （110）	No. of intersection	fatigue （68）	No. of intersection	fever （159）	No. of intersection	Myalgia/ Muscle soreness （165）
SMI (Shengmai Injection) [363]	34	4.27	22	2.98	30	19.00	31	3.77	40	8.33
LSY (Preventive prescription by Liu Shangyi) [382]	61	24.64	44	21.89	32	20.48	59	25.81	63	28.97
RDNI (Reduning Injection) [447]	69	26.58	52	26.68	35	20.75	64	24.79	70	29.83
SFT (Shenfu Injection) [447]	57	15.06	33	7.14	32	16.45	47	9.67	55	14.72
SJZT (Sijunzi Decoction) [498]	66	19.10	42	12.12	34	16.34	57	14.30	66	20.78
XBCQT (Xuanbai Chengqi Decoction) [507]	57	11.51	39	9.30	35	17.17	56	13.01	63	17.42
ECT (Erchen Decoction) [551]	70	18.77	47	14.03	37	17.50	59	12.90	70	20.48
XBJI (Xuebijing Injection) [624]	74	17.36	53	15.96	39	16.58	67	14.99	75	19.88
HXZQ (Huoxiang Zhengqi Powder) [728]	86	20.57	63	20.26	41	14.78	80	19.41	85	21.75
YPFS (Yupingfeng Powder) [780]	68	7.27	47	6.49	37	9.54	58	4.75	72	10.37
DYY (Dayuan Decoction) [856]	82	11.90	54	8.62	38	8.61	68	7.10	81	12.77

MXYG (Maxingyiigan Decoction) [1010]	91	11.54	62	9.59	41	7.80	77	7.31	88	11.44
YQS (Yinqiao Powder) [1017]	92	11.84	66	11.92	46	11.41	78	7.57	92	13.42
MXSG (Maxingshigan Decoction) [1021]	91	11.19	63	9.92	41	7.61	78	7.48	88	11.10
JHQG (Jinhuaqinggan Granule) [1120]	103	14.45	68	10.57	48	10.84	86	8.67	100	14.55
HSBD (Huashibaidu Decoction) [1131]	97	10.94	67	9.73	44	7.77	82	6.74	98	13.06
LHQW (Lianhuaqingwen Capsule) [1154]	102	12.80	71	11.60	49	10.94	84	7.06	99	12.89
QFPD (Qingfeipaidu Decoction) [1202]	102	11.39	73	11.69	47	8.62	92	9.44	103	13.61

(Continued)

**Recipes**	**No. of intersection**	**Lymph-openia（318）**	**No. of intersection**	**cytokine storm（23）**	**No. of intersection**	**oxygen saturation（41）**	**No. of intersection**	**Diarrhea（76）**	**No. of intersection**	**Dyspnea/ Difficult Breathing（52）**
SMI (Shengmai Injection) [363]	68	10.37	8	3.83	13	5.03	20	5.21	17	6.77
LSY (Preventive prescription by Liu Shangyi) [382]	116	50.46	13	11.44	20	13.93	29	13.47	25	16.88
RDNI (Reduning Injection) [447]	124	46.32	16	15.23	21	12.59	32	13.60	26	14.86
SFT (Shenfu Injection) [447]	95	20.14	13	9.32	14	4.29	23	5.22	17	4.68
SJZT (Sijunzi Decoction) [498]	111	26.60	14	9.64	18	7.13	27	6.86	20	6.13
XBCQT (Xuanbai Chengqi	109	24.08	10	4.01	21	10.45	26	5.88	24	9.90

Decoction) [507]										
ECT (Erchen Decoction) [551]	122	29.18	14	8.39	22	10.40	32	9.42	25	9.65
XBJI (Xuebijing Injection) [624]	133	29.69	14	7.01	22	8.52	35	9.74	28	10.75
HXZQ (Huoxiang Zhengqi Powder) [728]	151	32.54	19	12.32	26	10.55	39	10.09	33	13.24
YPFS (Yupingfeng Powder) [780]	118	10.23	11	2.41	23	6.55	31	3.87	28	7.18
DYY (Dayuan Decoction) [856]	137	14.74	15	5.14	23	5.47	33	3.82	28	5.94
MXYG (Maxingyiigan Decoction) [1010]	156	15.71	17	5.59	27	6.60	38	4.21	32	6.54
YQS (Yinqiao Powder) [1017]	161	17.67	17	5.53	28	7.33	42	6.17	33	7.16
MXSG (Maxingshigan Decoction) [1021]	157	15.63	17	5.49	27	6.46	39	4.55	32	6.39
JHQG (Jinhuaqinggan Granule) [1120]	177	20.10	18	5.56	30	7.61	44	5.75	35	7.14
HSBD (Huashibaidu Decoction) [1131]	173	17.65	17	4.57	27	5.21	42	4.61	34	6.30
LHQW (Lianhuaqingwen Capsule) [1154]	180	19.83	18	5.28	30	7.18	44	5.29	36	7.39
QFPD (Qingfeipaidu Decoction) [1202]	187	20.83	18	4.90	30	6.61	47	6.21	36	6.76

(Continued)

Recipes	No. of intersection	Mucus/ phlegm/	No. of intersection	SARS（38）	No. of intersection	coughing（32）	No. of intersection	Nausea/ vomiting

		suptum（124）						（58）
SMI (Shengmai Injection) [363]	28	5.04	10	2.83	12	6.20	16	4.68
LSY (Preventive prescription by Liu Shangyi) [382]	44	17.26	18	12.34	12	5.74	18	5.95
RDNI (Reduning Injection) [447]	49	18.36	19	11.03	15	7.98	20	6.03
SFT (Shenfu Injection) [447]	34	5.93	15	5.97	13	5.51	19	5.19
SJZT (Sijunzi Decoction) [498]	46	12.47	18	8.15	11	2.88	22	6.50
XBCQT (Xuanbai Chengqi Decoction) [507]	39	7.04	16	5.76	14	5.47	20	4.72
ECT (Erchen Decoction) [551]	48	11.61	18	6.92	12	3.06	24	6.95
XBJI (Xuebijing Injection) [624]	52	11.57	19	6.52	14	3.79	24	5.43
HXZQ (Huoxiang Zhengqi Powder) [728]	65	17.32	22	7.59	17	5.05	29	7.22
YPFS (Yupingfeng Powder) [780]	51	6.26	18	3.59	14	2.37	25	3.75
DYY (Dayuan Decoction) [856]	57	7.52	21	4.87	17	3.68	24	2.48
MXYG (Maxingyiigan Decoction) [1010]	62	6.62	22	3.99	23	6.94	30	3.80
YQS (Yinqiao Powder) [1017]	67	8.96	21	3.32	19	3.69	30	3.73
MXSG (Maxingshigan Decoction) [1021]	63	6.86	22	3.89	24	7.75	30	3.69
JHQG (Jinhuaqinggan Granule) [1120]	68	7.24	24	4.29	24	6.57	32	3.69
HSBD (Huashibaidu Decoction) [1131]	69	7.49	23	3.58	25	7.34	32	3.59
LHQW (Lianhuaqingwen Capsule) [1154]	72	8.49	25	4.64	24	6.21	32	3.38
QFPD (Qingfeipaidu Decoction) [1202]	76	9.51	24	3.62	26	7.46	35	4.39

18 representative recipes were refered to this coherence research, including SMI, LSY, RDNI, SFT, SJZT, XBCQT, ECT, XBJI, HXZQ, YPFS, DYY, MXYG, YQS, MXSG, JHQG, HSBD, LHQW and QFPD, which were sorted in order according to the merged target amounts (the actual number sit in the “[ ]”, e.g., QFPD (Qingfeipaidu Decoction) [1202], etc. 14 SSMs across the COVID-19 phases were listed in the first row, the same attached with target amounts in the “( )”, e.g., Mucus/phlegm/suptum（124）, etc. Different keywords for the same SSM were divided by “/”. The intersection of SSMs and remedies was presented the details in No. of intersection. The significant level of coherence (SLC) was the indicator to the effect on remedies to SSMs, attached here with

two decimal points.

TableS 15. The clinical trials of TCM remedies for combating COVID-19 registered in ChiCTR (Chinese Clinical Trial Registry).

NO.	Registration ID	Record Date	Title	Specific TCM Remedies	Sample Size
1	ChiCTR2000029381	2020/1/27	A prospective comparative study for Xue-Bi-Jing injection in the treatment of novel coronavirus pneumonia (COVID-19)	XBJI (Xuebijing injection)	No record
2	ChiCTR2000029400	2020/1/29	Clinical Controlled Trial for Traditional Chinese Medicine in the treatment of Novel Coronavirus Pneumonia (COVID-19)	/	60
3	ChiCTR2000029418	2020/1/30	Chinese Herbal medicine for Severe nevel coronavirus pneumonia (COVID-19): a Randomized Controlled Trial	/	42
4	ChiCTR2000029432	2020/2/1	A Real World Study for the Efficacy and Safety of Large Dose Tanreqing Injection in the Treatment of Patients with Novel Coronavirus Pneumonia (COVID-19)	Tanreqing Injection	72
5	ChiCTR2000029433	2020/2/1	A randomized, open-label, blank-controlled trial for Lian-Hua Qing-Wen Capsule/Granule in the treatment of suspected novel coronavirus pneumonia (COVID-19)	LHQW (Lianhuaqingwen capsule/granule)	240

6	ChiCTR2000029434	2020/2/1	A randomized, open-label, blank-controlled trial for Lian-Hua Qing-Wen Capsule/Granule in the treatment of novel coronavirus pneumonia (COVID-19)	LHQW (Lianhuaqingwen capsule/granule)	240
7	ChiCTR2000029435	2020/2/1	Randomized controlled trial for traditional Chinese medicine in the prevention of novel coronavirus pneumonia (COVID-19) in high risk population	/	160
8	ChiCTR2000029479	2020/2/2	Research for Traditional Chinese Medicine Technology Prevention and Control of 2019-nCoV Pneumonia (Novel Coronavirus Pneumonia, NCP) in the Community Population	/	20000
9	ChiCTR2000029487	2020/2/2	Clinical Study for Gu-Biao Jie-Du-Ling in Preventing of 2019-nCoV Pneumonia (Novel Coronavirus Pneumonia, NCP) in Children	Gu-Biao Jie-Du-Ling	200
10	ChiCTR2000029493	2020/2/2	Traditional Chinese Medicine for Pulmonary Fibrosis, Pulmonary Function and Quality of Life in Patients With 2019-nCoV Pneumonia (Novel Coronavirus Pneumonia, NCP) in Convalescent Period: a Randomized Controlled Trial	/	100
11	ChiCTR2000029589	2020/2/5	An open, prospective, multicenter clinical study for the efficacy and safety of Reduning injection in the treatment of 2019-nCoV pneumonia (novel coronavirus pneumonia,	RDNI (Reduning injection)	60

			NCP)		
12	ChiCTR2000029601	2020/2/6	Community based prevention and control for Chinese medicine in the treatment of 2019-nCoV pneumonia (novel coronavirus pneumonia, NCP) in the isolate suspected and confirmed population	/	400
13	ChiCTR2000029605	2020/2/7	A randomized, open-label, blank-controlled, multicenter trial for Shuang-Huang-Lian oral solution in the treatment of 2019-nCoV pneumonia (novel coronavirus pneumonia, NCP)	Shuang-Huang-Lian oral solution	No record
14	ChiCTR2000029624	2020/2/7	A real world study for traditional Chinese Medicine in the treatment of 2019-nCoV pneumonia (novel coronavirus pneumonia, NCP)	/	500
15	ChiCTR2000029628	2020/2/7	A clinical observational study for Xin-Guan-2 formula in the treatment of suspected 2019-nCoV pneumonia (novel coronavirus pneumonia, NCP)	Xin-Guan-2 formula	100
16	ChiCTR2000029637	2020/2/8	An observational study for Xin-Guan-1 formula in the treatment of 2019-nCoV pneumonia (novel coronavirus pneumonia, NCP)	Xin-Guan-1 formula	100

17	ChiCTR2000029747	2020/2/11	Effect evaluation and prognosis of Chinese medicine based on Novel Coronavirus Pneumonia (COVID-19)	/	200
18	ChiCTR2000029755	2020/2/12	A randomized, open, parallel-controlled trial for the efficacy and safety of Jingyebaidu granules in treating novel coronavirus pneumonia (COVID-19)	Jingyebaidu granules	120
19	ChiCTR2000029756	2020/2/12	Clinical study of nebulized Xiyanping injection in the treatment of novel coronavirus pneumonia (COVID-19)	Xiyanping injection	238
20	ChiCTR2000029763	2020/2/12	The efficacy of traditional chinese medicine on Novel Coronavirus Pneumonia (COVID-19) patients treated in square cabin hospital: a prospective, randomized controlled trial	/	408
21	ChiCTR2000029769	2020/2/13	Babaodan Capsule used for the adjuvant treatment of Severe novel coronavirus pneumonia (COVID-19)	Babaodan Capsule	40
22	ChiCTR2000029780	2020/2/13	A multicenter, randomized, open, controlled trial for the efficacy and safety of Shen-Qi Fu-Zheng injection in the treatment of novel coronavirus pneumonia (COVID-19)	Shen-Qi Fu-Zheng injection	160
23	ChiCTR2000029813	2020/2/14	Clinical Trial for Tanreqing Capsules in the Treatment of Novel Coronavirus Pneumonia (COVID-19)	/	72
24	ChiCTR2000029819	2020/2/14	Ba-Bao-Dan in the adjuvant therapy of novel coronavirus pneumonia (COVID-19) patients	Ba-Bao-Dan	80

25	ChiCTR2000029822	2020/2/14	A randomized controlled trial for honeysuckle decoction in the treatment of patients with novel coronavirus (COVID-19) infection	Honeysuckle decoction	110
26	ChiCTR2000029855	2020/2/15	A randomized, open and controlled clinical trial for traditional Chinese medicine in the treatment of novel coronavirus pneumonia (COVID-19)	/	180
27	ChiCTR2000029896	2020/2/16	Evaluate the effectiveness of Traditional Chinese medicine in the treatment of novel coronavirus pneumonia (COVID-19)	/	100
28	ChiCTR2000029941	2020/2/17	A randomized controlled trial for Traditional Chinese Medicine in the treatment for Novel Coronavirus Pneumonia (COVID-19)	/	200
29	ChiCTR2000029947	2020/2/17	A Randomized Controlled Trial for Qingyi No. 4 Compound in the treatment of Convalescence Patients of Novel Coronavirus Pneumonia (COVID-19)	Qingyi No. 4	200
30	ChiCTR2000029954	2020/2/17	Efficacy and safety of honeysuckle oral liquid in the treatment of novel coronavirus pneumonia (COVID-19): a multicenter, randomized, controlled, open clinical trial	Honeysuckle oral liquid	300
31	ChiCTR2000029956	2020/2/17	Chinese Medicine Promotes Rehabilitation Recommendations after 2019 Novel Coronavirus Infection (COVID-19)	/	120

32	ChiCTR2000029976	2020/2/18	The effect of Gymnastic Qigong Yangfeifang on functional recovery and quality of life in patients with mild novel coronavirus pneumonia (COVID-19)	Gymnastic Qigong Yangfeifang	160
33	ChiCTR2000029991	2020/2/18	A randomized, open-label, controlled trial for the safety and efficiency of Kesuting syrup and Keqing capsule in the treatment of mild and moderate novel coronavirus pneumonia (COVID-19)	Kesuting syrup and Keqing capsule	72
34	ChiCTR2000030022	2020/2/20	A parallel, randomized controlled clinical trial for the efficacy and safety of Pediatric Huatanzhike granules (containing ipecacuanha tincture) in the treatment of mild and moderate novel coronavirus pneumonia (COVID-19)	Pediatric Huatanzhike granules	100
35	ChiCTR2000030033	2020/2/21	A study for the intervention of Xiangxue antiviral oral solution and Wu-Zhi-Fang-Guan-Fang on close contacts of novel coronavirus pneumonia (COVID-19)	Xiangxue antiviral oral solution and Wu-Zhi-Fang-Guan-Fang	828
36	ChiCTR2000030034	2020/2/21	Traditional Chinese Medicine in the treatment of novel coronavirus pneumonia (COVID-19): a multicentre, randomized controlled trial	/	132
37	ChiCTR2000030043	2020/2/21	Shen-Fu injection in the treatment of severe novel coronavirus pneumonia (COVID-19): a multicenter, randomized, open-label, controlled trial	SFT (Shen-Fu injection)	300

38	ChiCTR2000030117	2020/2/23	A multicenter, randomized, open, parallel controlled trial for the evaluation of the effectiveness and safety of Xiyanping injection in the treatment of common type novel coronavirus pneumonia (COVID-19)	Xiyanping injection	348
39	ChiCTR2000030166	2020/2/24	Randomized, parallel control, open trial for Qing-Wen Bai-Du-Yin combined with antiviral therapy in the treatment of novel coronavirus pneumonia (COVID-19)	Qing-Wen Bai-Du-Yin	20
40	ChiCTR2000030188	2020/2/24	Clinical Study on Syndrome Differentiation of TCM in Treating Severe and Critical novel coronavirus pneumonia (COVID-19)	/	120
41	ChiCTR2000030215	2020/2/25	Study for the efficacy of Kangguan No. 1-3 prescription in the treatment of novel coronavirus pneumonia (COVID19)	Kangguan No. 1-3	120
42	ChiCTR2000030288	2020/2/27	Efficacy of Traditional Chinese Medicine in the Treatment of Novel Coronavirus Pneumonia (COVID-19): a Randomized Controlled Trial	/	204
43	ChiCTR2000030314	2020/2/28	Traditional Chinese medicine Ma-Xing-Shi-Gan-Tang and Sheng-Jiang-San in the treatment of children with novel coronavirus pneumonia (COVID-19)	MXSG (Maxingshigan decoction) and Shengjiang powder	40
44	ChiCTR2000030388	2020/3/1	Efficacy and safety of Xue-Bi-Jing injection in the treatment of severe cases of novel coronavirus pneumonia (COVID-19)	XBJI (Xuebijing injection)	60

45	ChiCTR2000030469	2020/3/2	A randomized parallel controlled trial for LIUSHENWAN in Treatment of Novel Coronavirus Pneumonia (COVID-19)	Liu-Shen-Wan	96
46	ChiCTR2000030479	2020/3/3	Study for the Effectiveness and Safety of Yi-Qi Hua-shi Jie-Du-Fang in the Treatment of the Novel Coronavirus Pneumonia (COVID-19)	Yi-Qi Hua-shi Jie-Du-Fang	100
47	ChiCTR2000030518	2020/3/5	Zedoary Turmeric Oil for Injection in the treatment of Novel Coronavirus Pneumonia (COVID-19): a randomized, open, controlled trial	Zedoary Turmeric Oil for Injection	60
48	ChiCTR2000030522	2020/3/5	Efficacy and safety of Ma-Xing-Gan-Shi decoction in the treatment of novel coronavirus pneumonia (COVID-19)	MXSG (Maxingshigan decoction	100
49	ChiCTR2000030545	2020/3/6	Efficacy and safety of honeysuckle oral liquid in the treatment of novel coronavirus pneumonia (COVID-19): a multicenter, randomized, controlled, open clinical trial	Honeysuckle oral liquid	300
50	ChiCTR2000030704	2020/3/10	Observation Of Clinical Efficacy And Safety of Bufonis Venenum Injection In The Treatment Of Severe Novel Coronavirus Pneumonia (COVID-19)	Bufonis Venenum Injection	50
51	ChiCTR2000030751	2020/3/13	Clinical Research for Traditional Mongolian Medicine in the treatment of novel coronavirus pneumonia (COVID-19)	/	60

52	ChiCTR2000030759	2020/3/13	Study for the therapeutic effect and mechanism of traditional Chinese medicine in the treatment of novel coronavirus pneumonia (COVID-19)	/	70
53	ChiCTR2000030804	2020/3/15	Exocarpium Citri Grandis Relieves Symptoms of Novel Coronavirus Pneunomia (COVID-19): a Randomized Controlled Clinical Trial	Exocarpium citri grandis	128
54	ChiCTR2000030864	2020/3/16	Traditional Chinese Medicine for novel coronavirus pneumonia (COVID-19)	/	50
55	ChiCTR2000030898	2020/3/16	Evaluation on the effect of Chushifangyi prescription in preventing novel coronavirus pneumonia (COVID-19)	Chushifangyi prescription	1000
56	ChiCTR2000030920	2020/3/17	Evaluation of the effect of taking tricholoma matsutake, cannabis sativa capsule and dendrobium candidum to nutrition intervention of patients with novel coronavirus pneumonia (COVID-19) during convalescence.	tricholoma matsutake, cannabis sativa capsule and dendrobium candidum	100
57	ChiCTR2000030936	2020/3/18	A real-world study for the Chinese medicines "Xinguan 2" and "Xinguan 3" in the treatment of novel coronavirus pneumonia (COVID-19)	Xin-Guan-2 formula, Xin-Guan-3 formula	2840
58	ChiCTR2000030937	2020/3/18	A randomized, open-label, controlled trial for Gu-Shen Ding-Chuan-Wan in the treatment of patients with novel coronavirus pneumonia (COVID-19) at recovery phase with Fei-Pi-Qi-Xu Zhen	Gu-Shen Ding-Chuan-Wan	144

59	ChiCTR2000030988	2020/3/20	A randomized controlled trial for Hua-Shi Bai-Du granules in patients with novel coronavirus pneumonia (COVID-19)	HSBD (Huashibaidu granules)	204
60	ChiCTR2000031089	2020/3/22	A medical records based study for Tou-Jie-Qu-Wen Granules in the Treatment of mild and moderate patients with novel coronavirus pneumonia (COVID-19)	Tou-Jie-Qu-Wen Granules	300
61	ChiCTR2000031672	2020/4/5	Development and application of TCM body regulating protection scheme for the convalescent population of novel coronavirus pneumonia (COVID-19)	/	150
62	ChiCTR2000031888	2020/4/13	A medical records based study for "Guangdong Pneumonia NO.1" in the Treatment of Novel Coronavirus Pneumonia (COVID-19)	Guangdong Pneumonia NO.1	300
63	ChiCTR2000031955	2020/4/15	Study for prevention of novel coronavirus pneumonia (COVID-19) in high risk population by Chinese medicine	/	3808
64	ChiCTR2000031982	2020/4/16	Clinical observation for the effect of Ke-Gan-Li-Yan oral liquid on the relief of laryngeal symptoms of novel coronavirus pneumonia (COVID-19) convalescence and suspected patients and other susceptible people	Ke-Gan-Li-Yan oral liquid	240
65	ChiCTR2000032098	2020/4/19	Danggui Shaoyao Powder in the synergistic treatment of novel coronavirus pneumonia (COVID-19)	Danggui Shaoyao Powder	300

66	ChiCTR2000032165	2020/4/21	A multicenter, randomized, double-blind, parallel-controlled trial for Qi-Mai-Fei-Luo-Ping Mixture in the improvement of lung function of novel coronavirus pneumonia (COVID-19) in the convalescent period	Qi-Mai-Fei-Luo-Ping Mixture	60
67	ChiCTR2000032205	2020/4/23	A multicenter randomized, double-blind, placebo-controlled trial for Sheng-Mai-Yin for improvement of the pulmonary heart function related symptoms of convalescence patients of new coronavirus pneumonia	SMI (Sheng-Mai-Yin)	200
68	ChiCTR2000032237	2020/4/23	A multicenter, randomized, double-blind, placebo-controlled trial for Xiang-Sha-Liu-Jun Pill in the treatment of novel coronavirus pneumonia (COVID-19) decline in digestive function during convalescence	Xiang-Sha-Liu-Jun Pill	200
69	ChiCTR2000032313	2020/4/25	Study for efficacy and safety of Jie-Xing-Jun-Zi granules in the Treatment of convalescent patients of novel coronavious pneumonia (COVID-19)	Jie-Xing-Jun-Zi granules	60
70	ChiCTR2000032399	2020/4/27	A multicenter, randomized, double-blind, placebo-controlled trial for Xiaoyao capsule in the improvement of sleep mood disorder of convalescence patients of novel coronavirus pneumonia (COVID-19)	Xiaoyao capsule	200

71	ChiCTR2000032412	2020/4/27	A medical record based retrospective study for the effectiveness and safety of Xi-Yan-Ping injection combined with conventional protocol in the treatment of common type novel coronavirus pneumonia (COVID-19)	Xiyanping injection	426
72	ChiCTR2000032461	2020/4/29	A medical record based retrospective study for effect of applying individualized Chinese herbal medicine in treatment of patients with novel coronavirus pneumonia (COVID-19)	/	500
73	ChiCTR2000032480	2020/4/29	Study on data management and diagnosis-treatment mode of TCM intervention for novel coronavirus pneumonia (COVID-19) convalescence	/	1000
74	ChiCTR2000032573	2020/5/3	A randomized, double-blind, controlled trial for Bu-Fei-Huo-Xue Capsule in the treatment of novel coronavirus pneumonia (COVID-19) convalescence patient with "Fei-Pi-Qi-Xu zhen"	Bu-Fei-Huo-Xue Capsule	120
75	ChiCTR2000032635	2020/5/4	Study for Screening of Chinese Patent Drugs in the Rehabilitation Treatment of novel coronavirus pneumonia (COVID-19)	/	60
76	ChiCTR2000032767	2020/5/9	A Medical Records Based Study for Clinical Efficacy and Safety of "clear lung detoxification soup" in the treatment of Novel Coronavirus Pneumonia (COVID-19)	QFPD (Qingfeipaidu decoction)	782

76 clinical trials with TCM remedies or TCM patents were selected here from 605 registered trials nationwide (the Supplementary Table 13 showed the full lists). Registration ID, record date, research title, TCM remedies, sample size, study design and current phase were included in this table. TCM remedies were named according to their titles, except for some discussed in Fig 5, e.g., QFPD (Qingfeipaidu decoction), etc.

TableS 16. The all list of clinical trials for COVID-19 registered in ChiCTR (Chinese Clinical Trial Registry).

NO.	Registration ID	Record Date	Research Institutions	Trials Title
1	ChiCTR2000029381	2020/1/27	The First Affiliated Hospital of Guangzhou Medical University	A prospective comparative study for Xue-Bi-Jing injection in the treatment of novel coronavirus pneumonia (COVID-19)
2	ChiCTR2000029400	2020/1/29	China Academy of Chinese Medical Sciences	Clinical Controlled Trial for Traditional Chinese Medicine in the treatment of Novel Coronavirus Pneumonia (COVID-19)
3	ChiCTR2000029418	2020/1/30	Dongzhimen Hospital Affiliated to Beijing University of Chinese Medicine	Chinese Herbal medicine for Severe nevel coronavirus pneumonia (COVID-19): a Randomized Controlled Trial
4	ChiCTR2000029432	2020/2/1	The First Afflicated Hospital of Guangzhou University of Chinese Medicine	A Real World Study for the Efficacy and Safety of Large Dose Tanreqing Injection in the Treatment of Patients with Novel Coronavirus Pneumonia (COVID-19)
5	ChiCTR2000029433	2020/2/1	Hebei Yiling Hospital, Renmin Hospital of Wuhan University	A randomized, open-label, blank-controlled trial for Lian-Hua Qing-Wen Capsule/Granule in the treatment of suspected novel coronavirus pneumonia (COVID-19)
6	ChiCTR2000029434	2020/2/1	Hebei Yiling Hospital, Renmin Hospital of Wuhan University	A randomized, open-label, blank-controlled trial for Lian-Hua Qing-Wen Capsule/Granule in the treatment of novel coronavirus pneumonia (COVID-19)
7	ChiCTR2000029435	2020/2/1	Wuhan 1st Hospital	Randomized controlled trial for traditional Chinese medicine in the prevention of novel coronavirus pneumonia (COVID-19) in high risk population

8	ChiCTR2000029479	2020/2/2	Hospital of Chengdu University of Traditional Chinese Medicine	Research for Traditional Chinese Medicine Technology Prevention and Control of 2019-nCoV Pneumonia (Novel Coronavirus Pneumonia, NCP) in the Community Population
9	ChiCTR2000029487	2020/2/2	Wuhan Hospital of Integrated Traditional Chinese and Western Medicine	Clinical Study for Gu-Biao Jie-Du-Ling in Preventing of 2019-nCoV Pneumonia (Novel Coronavirus Pneumonia, NCP) in Children
10	ChiCTR2000029493	2020/2/2	1. Xinhua affiliated hospital, Hubei University of Chinese Medicine; 2. Hubei Provincial Hospital of Integrated Chinese and Western Medicine	Traditional Chinese Medicine for Pulmonary Fibrosis, Pulmonary Function and Quality of Life in Patients With 2019-nCoV Pneumonia (Novel Coronavirus Pneumonia, NCP) in Convalescent Period: a Randomized Controlled Trial
11	ChiCTR2000029589	2020/2/5	Beijing hospital of Traditional Chinese Medicine; Hubei Integrated Traditional Chinese and Western Medicine Hospital	An open, prospective, multicenter clinical study for the efficacy and safety of Reduning injection in the treatment of 2019-nCoV pneumonia (novel coronavirus pneumonia, NCP)
12	ChiCTR2000029601	2020/2/6	Hubei Provincial Hospital of TCM	Community based prevention and control for Chinese medicine in the treatment of 2019-nCoV pneumonia (novel coronavirus pneumonia, NCP) in the isolate suspected and confirmed population
13	ChiCTR2000029605	2020/2/7	Tongji Hospital, Tongji Medical College, Huazhong University of Science and Technology	A randomized, open-label, blank-controlled, multicenter trial for Shuang-Huang-Lian oral solution in the treatment of 2019-nCoV pneumonia (novel coronavirus pneumonia, NCP)
14	ChiCTR2000029624	2020/2/7	Shanghai Public Health Clinical Center	A real world study for traditional Chinese Medicine in the treatment of 2019-nCoV pneumonia (novel coronavirus pneumonia, NCP)
15	ChiCTR2000029628	2020/2/7	Guangdong Provincial Hospital of Chinese Medicine	A clinical observational study for Xin-Guan-2 formula in the treatment of suspected 2019-nCoV pneumonia (novel coronavirus pneumonia, NCP)
16	ChiCTR2000029637	2020/2/8	Guangdong Provincial Hospital of Chinese Medicine	An observational study for Xin-Guan-1 formula in the treatment of 2019-nCoV pneumonia (novel coronavirus pneumonia, NCP)

17	ChiCTR2000029747	2020/2/11	The First Affiliated Hospital of Anhui University of Traditional Chinese Medicine	Effect evaluation and prognosis of Chinese medicine based on Novel Coronavirus Pneumonia (COVID-19)
18	ChiCTR2000029755	2020/2/12	Institutional Review Board, Tongji Hospital, Tongji Medical College, Huazhong University of Science and Technology	A randomized, open, parallel-controlled trial for the efficacy and safety of Jingyebaidu granules in treating novel coronavirus pneumonia (COVID-19)
19	ChiCTR2000029756	2020/2/12	Renmin Hospital of Wuhan University	Clinical study of nebulized Xiyanping injection in the treatment of novel coronavirus pneumonia (COVID-19)
20	ChiCTR2000029763	2020/2/12	China Academy of Chinese Medical Sciences	The efficacy of traditional chinese medicine on Novel Coronavirus Pneumonia (COVID-19) patients treated in square cabin hospital: a prospective, randomized controlled trial
21	ChiCTR2000029769	2020/2/13	Taizhou Hospital of Zhejiang Province	Babaodan Capsule used for the adjuvant treatment of Severe novel coronavirus pneumonia (COVID-19)
22	ChiCTR2000029780	2020/2/13	Union Hospital affiliated to Tongji Medical College of Huazhong University of Science and Technology	A multicenter, randomized, open, controlled trial for the efficacy and safety of Shen-Qi Fu-Zheng injection in the treatment of novel coronavirus pneumonia (COVID-19)
23	ChiCTR2000029813	2020/2/14	Shanghai Public Health Clinical Center	Clinical Trial for Tanreqing Capsules in the Treatment of Novel Coronavirus Pneumonia (COVID-19)
24	ChiCTR2000029819	2020/2/14	Sir Run Run Show Hospital, School of Medicine, Zhejiang University	Ba-Bao-Dan in the adjuvant therapy of novel coronavirus pneumonia (COVID-19) patients
25	ChiCTR2000029822	2020/2/14	Nanjing Second Hospital	A randomized controlled trial for honeysuckle decoction in the treatment of patients with novel coronavirus (COVID-19) infection
26	ChiCTR2000029855	2020/2/15	The First Affiliated Hospital of Medical College of Zhejiang University	A randomized, open and controlled clinical trial for traditional Chinese medicine in the treatment of novel coronavirus pneumonia (COVID-19)
27	ChiCTR2000029896	2020/2/16	Affiliated Hospital of Changchun University of traditional Chinese Medicine	Evaluate the effectiveness of Traditional Chinese medicine in the treatment of novel coronavirus pneumonia (COVID-19)

28	ChiCTR2000029941	2020/2/17	Longhua Hospital Affiliated to Shanghai University of Traditional Chinese Medicine	A randomized controlled trial for Traditional Chinese Medicine in the treatment for Novel Coronavirus Pneumonia (COVID-19)
29	ChiCTR2000029947	2020/2/17	Longhua Hospital Affiliated to Shanghai University of Traditional Chinese Medicine	A Randomized Controlled Trial for Qingyi No. 4 Compound in the treatment of Convalescence Patients of Novel Coronavirus Pneumonia (COVID-19)
30	ChiCTR2000029954	2020/2/17	Hubei Hospital of Traditional Chinese Medicine	Efficacy and safety of honeysuckle oral liquid in the treatment of novel coronavirus pneumonia (COVID-19): a multicenter, randomized, controlled, open clinical trial
31	ChiCTR2000029956	2020/2/17	Hospital of Chengdu University of Traditional Chinese Medicine	Chinese Medicine Promotes Rehabilitation Recommendations after 2019 Novel Coronavirus Infection (COVID-19)
32	ChiCTR2000029976	2020/2/18	Shanghai University of Traditional Chinese Medicine	The effect of Gymnastic Qigong Yangfeifang on functional recovery and quality of life in patients with mild novel coronavirus pneumonia (COVID-19)
33	ChiCTR2000029991	2020/2/18	The First Affiliated Hospital of Nanchang University	A randomized, open-label, controlled trial for the safety and efficiency of Kesuting syrup and Keqing capsule in the treatment of mild and moderate novel coronavirus pneumonia (COVID-19)
34	ChiCTR2000030022	2020/2/20	First Affiliated Hospital of Anhui Medical University	A parallel, randomized controlled clinical trial for the efficacy and safety of Pediatric Huatanzhike granules (containing ipecacuanha tincture) in the treatment of mild and moderate novel coronavirus pneumonia (COVID-19)
35	ChiCTR2000030033	2020/2/21	The First Affiliated Hospital of Guangzhou Medical University	A study for the intervention of Xiangxue antiviral oral solution and Wu-Zhi-Fang-Guan-Fang on close contacts of novel coronavirus pneumonia (COVID-19)
36	ChiCTR2000030034	2020/2/21	Tongde Hospital of Zhejiang Province	Traditional Chinese Medicine in the treatment of novel coronavirus pneumonia (COVID-19): a multicentre, randomized controlled trial

37	ChiCTR2000030043	2020/2/21	Peking University Third Hospital	Shen-Fu injection in the treatment of severe novel coronavirus pneumonia (COVID-19): a multicenter, randomized, open-label, controlled trial
38	ChiCTR2000030117	2020/2/23	Jiangxi Qingfeng Pharmaceutical Co., Ltd.	A multicenter, randomized, open, parallel controlled trial for the evaluation of the effectiveness and safety of Xiyanping injection in the treatment of common type novel coronavirus pneumonia (COVID-19)
39	ChiCTR2000030166	2020/2/24	The 5th Medical Center Chinese PLA General Hospital	Randomized, parallel control, open trial for Qing-Wen Bai-Du-Yin combined with antiviral therapy in the treatment of novel coronavirus pneumonia (COVID-19)
40	ChiCTR2000030188	2020/2/24	Guangdong Provincial Hospital of Chinese Medicine - Zhuhai Hospital	Clinical Study on Syndrome Differentiation of TCM in Treating Severe and Critical novel coronavirus pneumonia (COVID-19)
41	ChiCTR2000030215	2020/2/25	The First Affiliated Hospital of Nanchang University	Study for the efficacy of Kangguan No. 1-3 prescription in the treatment of novel coronavirus pneumonia (COVID19)
42	ChiCTR2000030288	2020/2/27	China Academy of Chinese Medical Sciences	Efficacy of Traditional Chinese Medicine in the Treatment of Novel Coronavirus Pneumonia (COVID-19): a Randomized Controlled Trial
43	ChiCTR2000030314	2020/2/28	Xiangyang Central Hospital, Affiliated Hospital of Hubei University of Arts and Sciences	Traditional Chinese medicine Ma-Xing-Shi-Gan-Tang and Sheng-Jiang-San in the treatment of children with novel coronavirus pneumonia (COVID-19)
44	ChiCTR2000030388	2020/3/1	Jingzhou First People's Hospital	Efficacy and safety of Xue-Bi-Jing injection in the treatment of severe cases of novel coronavirus pneumonia (COVID-19)
45	ChiCTR2000030469	2020/3/2	Shuguang Hospital Affiliated to Shanghai University of T.C.M.	A randomized parallel controlled trial for LIUSHENWAN in Treatment of Novel Coronavirus Pneumonia (COVID-19)
46	ChiCTR2000030479	2020/3/3	Affiliated Hospital of traditional Chinese and Western Medicine Nanjing University of Chinese Medicine	Study for the Effectiveness and Safety of Yi-Qi Hua-shi Jie-Du-Fang in the Treatment of the Novel Coronavirus Pneumonia (COVID-19)

47	ChiCTR2000030518	2020/3/5	the Second Affiliated Hospital of Wenzhou Medical University	Zedoary Turmeric Oil for Injection in the treatment of Novel Coronavirus Pneumonia (COVID-19): a randomized, open, controlled trial
48	ChiCTR2000030522	2020/3/5	Affiliated Hospital of Chengdu University of Traditional Chinese Medicine	Efficacy and safety of Ma-Xing-Gan-Shi decoction in the treatment of novel coronavirus pneumonia (COVID-19)
49	ChiCTR2000030545	2020/3/6	Hubei Hospital of Traditional Chinese Medicine	Efficacy and safety of honeysuckle oral liquid in the treatment of novel coronavirus pneumonia (COVID-19): a multicenter, randomized, controlled, open clinical trial
50	ChiCTR2000030704	2020/3/10	Jiangsu Provincial Hospital of Integrated Traditional Chinese and Western Medicine	Observation Of Clinical Efficacy And Safety Of Bufonis Venenum Injection In The Treatment Of Severe Novel Coronavirus Pneumonia (COVID-19)
51	ChiCTR2000030751	2020/3/13	Affilated Hospital of Inner Mongolia University for the Nationalities	Clinical Research for Traditional Mongolian Medicine in the treatment of novel coronavirus pneumonia (COVID-19)
52	ChiCTR2000030759	2020/3/13	The First Affiliated Hospital of Wenzhou Medical University	Study for the therapeutic effect and mechanism of traditional Chinese medicine in the treatment of novel coronavirus pneumonia (COVID-19)
53	ChiCTR2000030804	2020/3/15	Maoming People's Hospital	Exocarpium Citri Grandis Relieves Symptoms of Novel Coronavirus Pneunomia (COVID-19): a Randomized Controlled Clinical Trial
54	ChiCTR2000030864	2020/3/16	Xiangyang 1st People's Hospital	Traditional Chinese Medicine for novel coronavirus pneumonia (COVID-19)
55	ChiCTR2000030898	2020/3/16	Affiliated Hospital of Changchun University of Traditional Chinese Medicine	Evaluation on the effect of Chushifangyi prescription in preventing novel coronavirus pneumonia (COVID-19)
56	ChiCTR2000030920	2020/3/17	HwaMei Hospital, University of Chinese Academy of Sciences	Evaluation of the effect of taking tricholoma matsutake, cannabis sativa capsule and dendrobium candidum to nutrition intervention of patients with novel coronavirus pneumonia (COVID-19) during convalescence.

57	ChiCTR2000030936	2020/3/18	Hospital of Chengdu University of Traditional Chinese Medicine	A real-world study for the Chinese medicines "Xinguan 2" and "Xinguan 3" in the treatment of novel coronavirus pneumonia (COVID-19)
58	ChiCTR2000030937	2020/3/18	the First Affiliated Hospital of Guangzhou Medical University	A randomized, open-label, controlled trial for Gu-Shen Ding-Chuan-Wan in the treatment of patients with novel coronavirus pneumonia (COVID-19) at recovery phase with Fei-Pi-Qi-Xu Zhen
59	ChiCTR2000030988	2020/3/20	Guangdong Provincial Hospital of Chinese Medicine	A randomized controlled trial for Hua-Shi Bai-Du granules in patients with novel coronavirus pneumonia (COVID-19)
60	ChiCTR2000031089	2020/3/22	Guangzhou Eighth People's Hospital	A medical records based study for Tou-Jie-Qu-Wen Granules in the Treatment of mild and moderate patients with novel coronavirus pneumonia (COVID-19)
61	ChiCTR2000031672	2020/4/5	Beijing University of Chinese Medicine	Development and application of TCM body regulating protection scheme for the convalescent population of novel coronavirus pneumonia (COVID-19)
62	ChiCTR2000031888	2020/4/13	Guangzhou Eighth People's Hospital	A medical records based study for "Guangdong Pneumonia NO.1" in the Treatment of Novel Coronavirus Pneumonia (COVID-19)
63	ChiCTR2000031955	2020/4/15	Beijing University of traditional Chinese Medicine	Study for prevention of novel coronavirus pneumonia (COVID-19) in high risk population by Chinese medicine
64	ChiCTR2000031982	2020/4/16	The Fifth Affiliated Hospital of Sun Yat-Sen University	Clinical observation for the effect of Ke-Gan-Li-Yan oral liquid on the relief of laryngeal symptoms of novel coronavirus pneumonia (COVID-19) convalescence and suspected patients and other susceptible people
65	ChiCTR2000032098	2020/4/19	Union Hospital	Danggui Shaoyao Powder in the synergistic treatment of novel coronavirus pneumonia (COVID-19)

66	ChiCTR2000032165	2020/4/21	Hubei Provincial Hospital of TCM	A multicenter, randomized, double-blind, parallel-controlled trial for Qi-Mai-Fei-Luo-Ping Mixture in the improvement of lung function of novel coronavirus pneumonia (COVID-19) in the convalescent period
67	ChiCTR2000032205	2020/4/23	Wuhan Hospital Of Traditional Chinese and Western Medicine (Wuhan 1st Hospital)	A multicenter randomized, double-blind, placebo-controlled trial for Sheng-Mai-Yin for improvement of the pulmonary heart function related symptoms of convalescence patients of new coronavirus pneumonia
68	ChiCTR2000032237	2020/4/23	Ezhou Hospital of traditional Chinese Medicine	A multicenter, randomized, double-blind, placebo-controlled trial for Xiang-Sha-Liu-Jun Pill in the treatment of novel coronavirus pneumonia (COVID-19) decline in digestive function during convalescence
69	ChiCTR2000032313	2020/4/25	Beijing University of Chinese Medicine, Third Affiliated Hospital	Study for efficacy and safety of Jie-Xing-Jun-Zi granules in the Treatment of convalescent patients of novel coronavious pneumonia (COVID-19)
70	ChiCTR2000032399	2020/4/27	Xiaogan Traditional Chinese Medicine Hospital	A multicenter, randomized, double-blind, placebo-controlled trial for Xiaoyao capsule in the improvement of sleep mood disorder of convalescence patients of novel coronavirus pneumonia (COVID-19)
71	ChiCTR2000032412	2020/4/27	Jiangxi Qingfeng Pharmaceutical Co., Ltd./Shanghai Public Health Clinical Center	A medical records based retrospective study for the effectiveness and safety of Xi-Yan-Ping injection combined with conventional protocol in the treatment of common type novel coronavirus pneumonia (COVID-19)
72	ChiCTR2000032461	2020/4/29	Chengdu University of Traditional Chinese Medicine	A medical records based retrospective study for effect of applying individualized Chinese herbal medicine in treatment of patients with novel coronavirus pneumonia (COVID-19)
73	ChiCTR2000032480	2020/4/29	Institute of Basic Research for Clinical Medicine, China Academy of Chinese Medical Sciences/Guang'anmen Hospital, China Academy of Chinese Medical Sciences/	Study on data management and diagnosis-treatment mode of TCM intervention for novel coronavirus pneumonia (COVID-19) convalescence

74	ChiCTR2000032573	2020/5/3	The First Affiliated Hospital of Guangzhou Medical University	A randomized, double-blind, controlled trial for Bu-Fei-Huo-Xue Capsule in the treatment of novel coronavirus pneumonia (COVID-19) convalescence patient with "Fei-Pi-Qi-Xu zhen"
75	ChiCTR2000032635	2020/5/4	Hubei Provincial Hospital of TCM	Study for Screening of Chinese Patent Drugs in the Rehabilitation Treatment of novel coronavirus pneumonia (COVID-19)
76	ChiCTR2000029430	2020/2/1	Hubei Integrated Hospital of Traditional Chinese and Western Medicine	Study for the TCM syndrome characteristics of novel coronavirus pneumonia (COVID-19)
77	ChiCTR2000029460	2020/2/2	1. Xinhua affiliated hospital, Hubei University of Chinese Medicine; 2. Hubei Provincial Hospital of Integrated Chinese and Western Medicine	The effect of shadowboxing for pulmonary function and quality of life in patients with 2019-nCoV pneumonia (novel coronavirus pneumonia, NCP) in rehabilitation period
78	ChiCTR2000029462	2020/2/2	The First Affiliated Hospital of He'nan University of Chinese Medicine	Study for clinical characteristics and distribution of TCM syndrome of 2019-nCoV pneumonia (novel coronavirus pneumonia, NCP)
79	ChiCTR2000029517	2020/2/3	Zhejiang Chinese Medical University	Chinese medicine prevention and treatment program for suspected 2019-nCoV pneumonia (novel coronavirus pneumonia, NCP): a perspective, double-blind, placebo, randomised controlled trial
80	ChiCTR2000029518	2020/2/3	Zhejiang Chinese Medical University	Chinese medicine prevention and treatment program for 2019-nCoV pneumonia (novel coronavirus pneumonia, NCP): a perspective, double-blind, placebo, randomised controlled trial
81	ChiCTR2000029578	2020/2/5	Zhejiang Chinese Medical University	Chinese medicine prevention and treatment program for 2019-nCoV pneumonia (novel coronavirus pneumonia, NCP): a perspective, sing-arm trial
82	ChiCTR2000029789	2020/2/13	He'nan University of Chinese Medicine	Randomized controlled trial for TCM syndrome differentiation treatment impacting quality of life of post-discharge patients with novel coronavirus pneumonia (COVID-19)

83	ChiCTR2000029978	2020/2/18	Tuina Institute, Yueyang Hospital of Integrated Traditional Chinese and Western Medicine, Shanghai University of Traditional Chinese Medicine	A randomized controlled trial for the efficacy of Dao-Yin in the prevention and controlling novel coronavirus pneumonia (COVID-19)
84	ChiCTR2000029994	2020/2/19	Huangshi Hospital of TCM/Faculty of Rehabilitation Medicine, Shanghai University of Chinese Medicine	Liu-Zi-Jue Qigong and Acupressure Therapy for Pulmonary Function and Quality of Life in Patient with Severe novel coronavirus pneumonia (COVID-19): A Randomized Controlled Trial
85	ChiCTR2000030225	2020/2/25	Yueyang Hospital of Integrated Traditional Chinese and Western Medicine, Shanghai University of Traditional Chinese Medicine	Clinical Reseach of Acupuncture in the treatment of Novel Coronavirus Pneumonia (COVID-19)
86	ChiCTR2000030324	2020/2/28	Xiangyang Central Hospital, Affiliated Hospital of Hubei University of Arts and Sciences	Traditional Chinese Medicine 'Zang-Fu Point-pressing' massage for children with novel coronavirus pneumonia (COVID-19)
87	ChiCTR2000030386	2020/2/18	Hu'nan University of Chinese Medicine	Study for moxibustion in the preventing of novel coronavirus pneumonia (COVID-19)
88	ChiCTR2000030389	2020/3/1	Hubei Provincial Hospital of TCM	A Comparative Study for the Effectiveness of "triple energizer treatment" Method in Repairing Lung Injury in Patients with Novel coronavirus pneumonia (COVID-19)
89	ChiCTR2000030418	2020/3/1	The First Affiliated Hospital of Fujian Medical University	Application of rehabilitation lung exercise eight-segment exercise in patients with novel coronavirus pneumonia (COVID-19)
90	ChiCTR2000030420	2020/3/1	Institute of Integrative Medicine of Dalian Medical University	A Clinical Trial Study for the Influence of TCM Psychotherapy on Negative Emotion of Patients with Novel Coronavirus Pneumonia (COVID-19) Based on Network Platform
91	ChiCTR2000030432	2020/3/1	The First Affiliated Hospital of Fujian Medical University	Application of rehabilitation and Lung eight-segment exercise in front-line nurses in the prevention of novel coronavirus pneumonia (COVID-19) epidemic

92	ChiCTR2000030433	2020/3/1	The First Affiliated Hospital of Fujian Medical University	Application of Rehabilitation and Lung Eight-segment Exercise in Home Rehabilitation of Survivors from novel coronavirus pneumonia (COVID-19)
93	ChiCTR2000030467	2020/3/2	Dalian Medical University	A Randomized Controlled Trial for the Influence of TCM Psychotherapy on Negative Emotion of Patients with Novel Coronavirus Pneumonia (COVID-19) Based on Network Platform
94	ChiCTR2000030468	2020/3/2	Affiliated Hospital of Shandong University of Traditional Chinese Medicine	Study for the key technique of integrative therapy of Novel Coronavirus Pneumonia (COVID-19): the TCM symptoms and treatment regulation
95	ChiCTR2000030528	2020/3/5	The Second Clinical College of Guangzhou University of Chinese Medicine	Application of TCM Nursing Scheme in Patients with Novel Coronavirus Pneumonia (COVID-19)
96	ChiCTR2000030597	2020/3/8	Xiyuan Hospital, Chinese Academy of Traditional Chinese Medicine	Medical records based study for the correlation between Chinese medicine certificate and lung image of novel coronavirus pneumonia (COVID-19)
97	ChiCTR2000030606	2020/3/8	Xiyuan Hospital, Chinese Academy of Traditional Chinese Medicine	Medical records based study for the correlation between Chinese medicine certificate and lung image of novel coronavirus pneumonia (COVID-19)
98	ChiCTR2000030747	2020/3/13	Hubei Provincial Hospital of TCM	A prospective cohort study for comprehensive treatment of Chinese medicine in the treatment of convalescent patients of novel coronavirus pneumonia (COVID-19)
99	ChiCTR2000030810	2020/3/15	Hubei 672 Orthopaedics Hospital of Integrated Chinese＆Western Medicine	Clinical observation and evaluation of traditional Chinese medicine in the treatment of novel coronavirus pneumonia (COVID-19) in Hubei 672 Orthopaedics Hospital of Integrated Chinese＆Western Medicine
100	ChiCTR2000030896	2020/3/16	Zhengzhou People's Hospital	Clinical Application and Theoretical Discussion of Fu-Zheng Qing-Fei Thought in Treating Non-Critical Novel Coronavirus Pneumonia (COVID-19)
101	ChiCTR2000030938	2020/3/18	First Teaching Hospital of Tianjin University of	Clinical investigation and reseach on TCM syndrome of novel coronavirus

			Traditional Chinese Medicine	pneumonia (COVID-19)
102	ChiCTR2000030940	2020/3/18	Longhua Hospital Affiliated to Shanghai University of traditional Chinese Medicine	Study for "Bai-Du Duan Fang" application on the acupoint in the treatment of general type novel coronavirus pneumonia (COVID-19)
103	ChiCTR2000030962	2020/3/20	Hospital of Chengdu University of Traditional Chinese Medicine	Clinical efficacy of TCM syndrome differentiation in the treatment of severe/critical type of novel coronavirus pneumonia (COVID-19): a prospective, observational, one-arm clinical study
104	ChiCTR2000030996	2020/3/20	Nursing Department of Xiyuan Hospital, Chinese Academy of Traditional Chinese Medicine	Effect of Auricular point pressing on insomnia of novel coronavirus pneumonia (COVID-19) patients: a randomized controlled trial.
105	ChiCTR2000031203	2020/3/24	Zhengzhou People's Hospital	Study for the effect of Moxibustion Guidance and Intervention based on internet for the discharged patients with novel coronavirus pneumonia (COVID-19)
106	ChiCTR2000032367	2020/4/26	Yueyang Integrated Traditional Chinese and Western Medicine Hospital Affiliated to Shanghai University of Traditional Chinese Medicine	Efficacy of Liu-zi-jue in Patients with 2019 Novel Coronavirus Pneumonia (COVID-19): a randomized controlled trial
107	ChiCTR2000032767	2020/5/9	Institute of Basic Research for Clinical Medicine, China Academy of Chinese Medical Sciences	A Medical Records Based Study for Clinical Efficacy and Safety of "clear lung detoxification soup" in the treatment of Novel Coronavirus Pneumonia (COVID-19)
108	ChiCTR2000029436	2020/2/1	The First Hospital of He'nan University of Chinese Medicine	A single arm study for evaluation of integrated traditional Chinese and western medicine in the treatment of novel coronavirus pneumonia (COVID-19)
109	ChiCTR2000029437	2020/2/1	Hubei Provincial Integrated Hospital of traditional Chinese and Western Medicine	A single arm study for combination of traditional Chinese and Western Medicine in the treatment of novel coronavirus pneumonia (COVID-19)

110	ChiCTR2000029438	2020/2/1	Beijing hospital of Traditional Chinese medicine; Hubei integrated traditional Chinese and Western Medicine Hospital	A randomized controlled trial of integrated TCM and Western Medicine in the treatment of severe 2019-nCoV pneumonia (novel coronavirus pneumonia, NCP)
111	ChiCTR2000029439	2020/2/1	Beijing Hospital of Traditional Chinese Medicine affiliated to Capital Medical University/Beijing institute of Traditional Chinese MedHubei integrated traditional Chinese and Western Medicine Hospital	Combination of traditional chinese medicne and western medicine in the treatment of common type 2019-nCoV pneumonia (novel coronavirus pneumonia, NCP)
112	ChiCTR2000029461	2020/2/2	1. Xinhua affiliated hospital, Hubei University of Chinese Medicine; 2. Hubei Provincial Hospital of Integrated Chinese and Western Medicine	A Randomized Controlled Trial for Integrated Traditional Chinese Medicine and Western Medicine in the Treatment of Common Type 2019-nCoV Pneumonia (Novel Coronavirus Pneumonia, NCP)
113	ChiCTR2000029549	2020/2/4	Hospital of Chengdu University of Traditional Chinese Medicine	Recommendations of Integrated Traditional Chinese and Western Medicine for 2019-nCoV Pneumonia (Novel Coronavirus Pneumonia, NCP)
114	ChiCTR2000029558	2020/2/4	Hospital of Chengdu University of Traditional Chinese Medicine	Recommendations of Integrated Traditional Chinese and Western Medicine for Diagnosis and Treatment of 2019-nCoV Pneumonia (Novel Coronavirus Pneumonia, NCP) in Sichuan Province
115	ChiCTR2000029751	2020/2/12	The First Affiliated Hospital of Zhejiang University of Traditional Chinese Medicine	Clinical Study for Traditional Chinese Medicine in the Prevention and Treatment of Novel Coronavirus Pneumonia (COVID-19)
116	ChiCTR2000029777	2020/2/13	Huangshi Hospital of Traditional Chinese Medicine	A multicenter, randomized, controlled trial for integrated chinese and western medicine in the treatment of novel coronavirus pneumonia (COVID-19) based on the 'Truncated Torsion' strategy
117	ChiCTR2000029778	2020/2/13	Shuguang Hospital Affiliated to Shanghai University of TCM.	Clinical Study for Traditional Chinese Medicine Combined With Western Medicine in Treatment of Novel Coronavirus Pneumonia (COVID-19)
118	ChiCTR2000029788	2020/2/13	Dongzhimen Hospital Affiliated to Beijing University of Chinese Medicine	Traditional Chinese medicine cooperative therapy for patients with novel coronavirus pneumonia (COVID-19): a randomized controlled trial

119	ChiCTR2000029790	2020/2/13	Shanghai Pulmonary Hospital	Clinical study for the integration of traditional Chinese and western medicine in the treatment of novel coronavirus pneumonia (COVID-19)
120	ChiCTR2000029814	2020/2/14	Children's Hospital of Fudan University	Clinical Trial for Integrated Chinese and Western Medicine in the Treatment of Children with Novel Coronavirus Pneumonia (COVID-19)
121	ChiCTR2000029869	2020/2/15	Huangshi Hospital of Traditional Chinese Medicine	A multicenter, randomized, controlled trial for integrated Chinese and western medicine in the treatment of ordinary novel coronavirus pneumonia (COVID-19) based on the ' Internal and External Relieving -Truncated Torsion' strategy
122	ChiCTR2000029960	2020/2/17	The First Affiliated Hospital of Hu'nan University of traditional Chinese Medicine	Comparative study for integrate Chinese and conventional medicine the the treatment of novel coronavirus pneumonia (COVID-19) in Hu'nan province
123	ChiCTR2000029993	2020/2/19	The First Affiliated Hospital of Guangzhou Medical University	A pilot study for Integrated Chinese and Western Medicine in the treatment of non-critical novel coronavirus pneumonia (COVID-19)
124	ChiCTR2000030000	2020/2/19	Nanchang Ninth Hospital	An open, controlled clinical trial for evaluation of ganovo combined with ritonavir and integrated traditional Chinese and Western medicine in the treatment of novel coronavirus infection (COVID-19)
125	ChiCTR2000030003	2020/2/19	Affiliated Hospital of Chengdu University of Traditional Chinese Medicine	Optimization Protocal of Integrated Traditional Chinese and Western Medicine in the Treatment for Novel Coronavirus Pneumonia (COVID-19)
126	ChiCTR2000030027	2020/2/20	Dongzhimen Hospital Affiliated to Beijing University of Chinese Medicine, Beijing China	Traditional Chinese medicine cooperative therapy for patients with Novel coronavirus pneumonia (COVID-19) and its effect on spermatogenesis: a randomized controlled trial
127	ChiCTR2000030219	2020/2/25	First Teaching Hospital of Tianjin University of Traditional Chinese Medicine	Study for evaluation of integrated traditional Chinese and Western Medicine in the treatment of Novel Coronavirus Pneumonia (COVID-19)

128	ChiCTR2000030305	2020/2/28	The Fourth Affiliated Hospital of Zhejiang University School of Medicine	Multiomics study and emergency plan optimization of spleen strengthening clearing damp and stomach therapy combined with antiviral therapy for novel coronavirus pneumonia (COVID-19)
129	ChiCTR2000030315	2020/2/28	Affiliated Hospital of Changchun University of traditional Chinese Medicine	Clinical Study for Traditional Chinese Medicine in the Prevention and Treatment of Novel Coronavirus Pneumonia (COVID-19)
130	ChiCTR2000030619	2020/3/8	The First Affiliated Hospital of Bengbu Medical College	A medical records based real world study for the characteristics and correlation mechanism of traditional Chinese medicine combined with western medicine in the treatment of patients with novel coronavirus pneumonia (COVID-19)
131	ChiCTR2000030719	2020/3/11	Wuhan Hospital of Integrated Traditional Chinese and Western Medicine	A retrospective cohort study for integrated traditional Chinese and western medicine in the treatment of 1071 patients with novel coronavirus pneumonia (COVID-19) in Wuhan
132	ChiCTR2000030806	2020/3/15	Wuhan 3rd Hospital	Retrospective study for the efficacy of ulinastatin combined with "clear lung detoxification soup" in the treatment of novel coronavirus pneumonia (COVID-19)
133	ChiCTR2000030836	2020/3/15	Longhua Hospital Affiliated to Shanghai University of traditional Chinese Medicine	Novel coronavirus pneumonia (COVID-19) combined with Chinese and Western medicine based on "Internal and External Relieving -Truncated Torsion" strategy
134	ChiCTR2000030933	2020/3/18	The First Affiliated Hospital of Nanchang University	Effectiveness of "Liu-Zi-Jue" combined with respiratory muscle training for respiratory function in novel coronavirus pneumonia (COVID-19) patients: a randomized controlled trial
135	ChiCTR2000032040	2020/4/18	Affiliated Hospital of Guangdong Medical University	A medical records based study for the curative effect of combined traditional Chinese and western medicine in the treatment of severe novel coronavirus pneumonia (COVID-19)

136	ChiCTR2000032717	2020/5/8	Xi'an International Medical Center Hospital	Efficacy and safety of high-dose vitamin C combined with traditional Chinese medicine in the treatment of moderate and severe novel coronavirus pneumonia (COVID-19)
137	ChiCTR2000029308	2020/1/23	Wuhan Jinyintan Hospital (Wuhan Infectious Diseases Hospital)	A randomized, open-label, blank-controlled trial for the efficacy and safety of lopinavir-ritonavir and interferon-alpha 2b in hospitalization patients with novel coronavirus pneumonia (COVID-19)
138	ChiCTR2000029386	2020/1/28	Chongqing Public Health Medical Center	Adjunctive Corticosteroid Therapy for Patients with Severe Novel Coronavirus Pneumonia (COVID-19): a Randomized Controlled Trial
139	ChiCTR2000029387	2020/1/28	Chongqing Public Health Medical Center	Comparison of efficacy and safety of three antiviral regimens in patients with mild to moderate novel coronavirus pneumonia (COVID-19): a randomized controlled trial
140	ChiCTR2000029431	2020/2/1	Affiliated Zhongshan Hospital of Dalian University	Clinical study for the remedy of M1 macrophages target in the treatment of novel coronavirus pneumonia (COVID-19)
141	ChiCTR2000029459	2020/2/2	1.Xinhua affiliated hospital, Hubei University of Chinese Medicine; 2. Hubei Provincial Hospital of Integrated Chinese and Western Medicine	The effect of pulmonary rehabilitation for pulmonary function and quality of life in patients with 2019-nCoV pneumonia (novel coronavirus pneumonia, NCP) in rehabilitation period
142	ChiCTR2000029468	2020/2/2	Institute of Emergency Medicine and Disaster Medicine Sichuan People's Hospital, Sichuan Academy of Medical Sciences	A real-world study for lopinavir/ritonavir (LPV/r) and emtritabine (FTC) / Tenofovir alafenamide Fumarate tablets (TAF) regimen in the treatment of 2019-nCoV pneumonia (novel coronavirus pneumonia, NCP)
143	ChiCTR2000029495	2020/2/2	1. Xinhua Affiliated Hospital, Hubei University of Chinese Medicine; 2. Hubei Provincial Hospital of Integrated Chinese and Western Medicine	Traditional Chinese Medicine, Psychological Intervention and Investigation of Mental Health for Patients With 2019-nCoV Pneumonia (Novel Coronavirus Pneumonia, NCP) in Convalescent Period

144	ChiCTR2000029496	2020/2/3	The First Hospital of Changsha; The Second Xiangya Hospital of Central South University	A randomized, open label, parallel controlled trial for evaluating the efficacy of recombinant cytokine gene-derived protein injection in eliminating novel coronavirus in patients with 2019-nCoV pneumonia (novel coronavirus pneumonia, NCP)
145	ChiCTR2000029539	2020/2/3	Tongji Hospital, Tongji Medical College, Huazhong University of Science and Technology	A randomized, open-label study to evaluate the efficacy and safety of Lopinavir-Ritonavir in patients with mild 2019-nCoV pneumonia (novel coronavirus pneumonia, NCP)
146	ChiCTR2000029541	2020/2/3	Zhongnan Hospital of Wuhan University	A randomised, open, controlled trial for darunavir/cobicistat or Lopinavir/ritonavir combined with thymosin a1 in the treatment of 2019-nCoV pneumonia (novel coronavirus pneumonia, NCP)
147	ChiCTR2000029542	2020/2/3	Sun Yat sen Memorial Hospital of Sun Yat sen University	Study for the efficacy of chloroquine in patients with 2019-nCoV pneumonia (novel coronavirus pneumonia, NCP)
148	ChiCTR2000029544	2020/2/3	The First Hospital Affiliated to Zhejiang University's Medical School	A randomized controlled trial for the efficacy and safety of Baloxavir Marboxil, Favipiravir tablets in 2019-nCoV pneumonia (novel coronavirus pneumonia, NCP) patients who are still positive on virus detection under the current antiviral therapy
149	ChiCTR2000029548	2020/2/4	The First Affiliated Hospital, Zhejiang University School of Medicine	Randomized, open-label, controlled trial for evaluating of the efficacy and safety of Baloxavir Marboxil, Favipiravir, and Lopinavir-Ritonavir in the treatment of 2019-nCoV pneumonia (novel coronavirus pneumonia, NCP) patients
150	ChiCTR2000029550	2020/2/4	Hospital of Chengdu University of Traditional Chinese Medicine	Recommendations for Diagnosis and Treatment of Influenza Patients in the Hospital of Chengdu University of Traditional Chinese Medicine Under the Raging of 2019-nCoV Pneumonia (Novel Coronavirus Pneumonia, NCP)
151	ChiCTR2000029559	2020/2/4	Renmin Hospital of Wuhan University	Therapeutic effect of hydroxychloroquine on 2019-nCoV pneumonia (novel coronavirus pneumonia, NCP)

152	ChiCTR2000029569	2020/2/4	Xiangyang 1st People's Hospital	Safety and efficacy of umbilical cord blood mononuclear cells conditioned medium in the treatment of severe and critically 2019-nCoV pneumonia (novel coronavirus pneumonia, NCP): a randomized controlled trial
153	ChiCTR2000029572	2020/2/5	Xiangyang First People's Hospital	Safety and efficacy of umbilical cord blood mononuclear cells in the treatment of severe and critically 2019-nCoV pneumonia (novel coronavirus pneumonia, NCP): a randomized controlled clinical trial
154	ChiCTR2000029573	2020/2/4	The First Affiliated Hospital of Medical College of Zhejiang University	A multicenter, randomized, open-label, positive-controlled trial for the efficacy and safety of recombinant cytokine gene-derived protein injection combined with abidole, lopinavir/litonavir in the treatment of 2019-nCoV pneumonia (novel coronavirus pneumonia, NCP) patients
155	ChiCTR2000029579	2020/2/5	Department of Hematology, Tongji Hospital, Tongji Medical College, Huazhong University of Science and Technology	Cytokines profiling and their clinical significance analysis of 2019-nCoV pneumonia (novel coronavirus pneumonia, NCP) patients
156	ChiCTR2000029580	2020/2/5	Department of Hematology, Tongji Hospital, Tongji Medical College, Huazhong University of Science and Technology	A prospective, single-blind, randomized controlled trial for Ruxolitinib combined with mesenchymal stem cell infusion in the treatment of patients with severe 2019-nCoV pneumonia (novel coronavirus pneumonia, NCP)
157	ChiCTR2000029592	2020/2/5	Union Hospital, Tongji Medical College, Huazhong University of Science and Technology	Study for Arbidol Hydrochloride in the Prophylaxis of Novel Coronavirus Infection in High-risk Population with History of Exposed to 2019-nCoV pneumonia
158	ChiCTR2000029600	2020/2/6	The Third People's Hospital of Shenzhen	Clinical study for safety and efficacy of Favipiravir in the treatment of 2019-nCoV pneumonia (novel coronavirus pneumonia, NCP)
159	ChiCTR2000029602	2020/2/6	Hubei Provincial Hospital of TCM	Clinical study for community based prevention and control strategy of novel coronavirus pneumonia (COVID-19) in the isolate suspected and confirmed population

160	ChiCTR2000029603	2020/2/6	The First Affiliated Hospital of Zhejiang University School of Medicine	A Randomized, Open-Label, Multi-Centre Clinical Trial Evaluating and Comparing the Safety and Efficiency of ASC09/Ritonavir and Lopinavir/Ritonavir for Confirmed Cases of 2019-nCoV Pneumonia (Novel Coronavirus Pneumonia, NCP)
161	ChiCTR2000029606	2020/2/7	The First Affiliated Hospital, College of Medicine, Zhejiang University	Clinical Study for Human Menstrual Blood-Derived Stem Cells in the Treatment of Acute Novel Coronavirus Pneumonia (NCP)
162	ChiCTR2000029609	2020/2/6	The Fifth Affiliated Hospital of Sun Yat-Sen University	A prospective, open-label, multiple-center study for the efficacy of chloroquine phosphate in patients with 2019-nCoV pneumonia (novel coronavirus pneumonia, NCP)
163	ChiCTR2000029621	2020/2/7	Ruijin Hospital, Shanghai Jiao Tong University School of Medicine	Clinical study of arbidol hydrochloride tablets in the treatment of 2019-nCoV pneumonia (novel coronavirus pneumonia, NCP)
164	ChiCTR2000029625	2020/2/7	The First Affiliated Hospital of Zhejiang University School of Medicine	Construction of Early Warning and Prediction System for Patients with Severe / Critical 2019-nCoV Pneumonia (Novel Coronavirus Pneumonia, NCP)
165	ChiCTR2000029626	2020/2/7	The First Affiliated Hospital of Zhejiang University School of Medicine	Immune Repertoire (TCR & BCR) Evaluation and Immunotherapy Research in Peripheral Blood of 2019-nCoV Pneumonia (Novel Coronavirus Pneumonia, NCP) Patients
166	ChiCTR2000029636	2020/2/8	Union Hospital, Tongji Medical College, Huazhong University of Science and Technology	Efficacy and safety of aerosol inhalation of vMIP in the treatment of 2019-nCoV pneumonia (novel coronavirus pneumonia, NCP): a single arm clinical trial
167	ChiCTR2000029638	2020/2/8	West China Hospital, Sichuan University	Multicenter randomized controlled trial for novel recombinant high-efficiency compound interferon in the treatment of novel coronavirus pneumonia (COVID-19)
168	ChiCTR2000029639	2020/2/8	Department of Psychology, the Fifth Affiliated Hospital Sun Yat-Sen University	Study for Mental health and psychological status of doctors, nurses and patients in novel coronavirus pneumonia (COVID-19) designated hospital and effect of interventions

169	ChiCTR2000029656	2020/2/9	Wuhan Pulmonary Hospital	A randomized, open-label study to evaluate the efficacy and safety of low-dose corticosteroids in hospitalized patients with 2019-nCoV pneumonia (novel coronavirus pnuemonia, NCP)
170	ChiCTR2000029695	2020/2/10	Shenzhen Third People's Hospital	Early Detection of Novel Coronavirus Pneumonia Based on a Novel High-Throughput Mass Spectrometry Analysis With Exhaled Breath
171	ChiCTR2000029728	2020/2/10	Tongji Hospital, Tongji Medical College, Huazhong University of Science and Technology	A Retrospective Study for Preventive Medication in Tongji Hospital during the epidemic of 2019-nCoV Pneumonia (novel coronavirus pneumonia, NCP)
172	ChiCTR2000029732	2020/2/10	West China Hospital, Sichuan University	Impact of vitamin D deficiency on prognosis of novel coronavirus pneumonia patients
173	ChiCTR2000029734	2020/2/10	The First People's Hospital of Huaihua	Epidemiological investigation and clinical characteristics analysis of novel coronavirus pneumonia (NCP)
174	ChiCTR2000029735	2020/2/10	Tongji Hospital, Tongji Medical College, Huazhong University of Science and Technology	Risks of Death and Severe cases in Patients with 2019 Novel Coronavirus Pneumonia
175	ChiCTR2000029739	2020/2/11	The First Affiliated Hospital of Guangzhou Medical University	A Multicenter, Randomized, Parallel Controlled Clinical Study of Hydrogen-Oxygen Nebulizer to Improve the Symptoms of Patients With Novel Coronavirus Pneumonia (COVID-19)
176	ChiCTR2000029740	2020/2/11	The First hospital of Peking University	Efficacy of therapeutic effects of hydroxycholoroquine in 2019-nCoV pneumonia (novel coronavirus pneumonia) patients
177	ChiCTR2000029741	2020/2/11	The Fifth Affiliated Hospital Sun Yat-Sen University	Efficacy of Chloroquine and Lopinavir/ Ritonavir in mild/general novel coronavirus (CoVID-19) infections: a prospective, open-label, multicenter randomized controlled clinical study
178	ChiCTR2000029742	2020/2/11	Tongji Hospital, Tongji Medical College, Huazhong University of Science and Technology	A randomized, parallel controlled trial for the efficacy and safety of Sodium Aescinate Injection in the treatment of patients with pneumonia (COVID-19)

179	ChiCTR2000029754	2020/2/12	West China Hospital, Sichuan University	Study for the Effect of Novel Coronavirus Pneumonia (COVID-19) on the Health of Different People
180	ChiCTR2000029757	2020/2/12	Institute of Blood Transfusion, Chinese Academy of Medical Sciences	Convalescent plasma for the treatment of severe novel coronavirus pneumonia (COVID-19): a prospective randomized controlled trial
181	ChiCTR2000029758	2020/2/12	West China Hospital, Sichuan University	Cohort Study of Novel Coronavirus Pneumonia (COVID-19) Critical Ill Patients
182	ChiCTR2000029764	2020/2/13	West China Hospital, Sichuan University	Imaging Features and Mechanisms of Novel Coronavirus Pneumonia (COVID-19): a Multicenter Study
183	ChiCTR2000029765	2020/2/13	The First Affiliated Hospital of University of science and technology of China (Anhui Provincial Hospital)	A multicenter, randomized controlled trial for the efficacy and safety of tocilizumab in the treatment of new coronavirus pneumonia (COVID-19)
184	ChiCTR2000029768	2020/2/13	Zhongnan Hospital of Wuhan University	A randomized, open, controlled trial for diammonium glycyrrhizinate enteric-coated capsules combined with vitamin C tablets in the treatment of common novel coronavirus pneumonia (COVID-19) in the basic of clinical standard antiviral treatment to evaluate the safety and efficiency
185	ChiCTR2000029770	2020/2/13	Union Hospital, Tongji Medical College, Huazhong University of Science and Technology	Study for epidemiology, diagnosis and treatment of novel coronavirus pneumonia (COVID-19)
186	ChiCTR2000029776	2020/2/13	The First Affiliated of Wenzhou Medical University	A randomized, open-label, blank-controlled, multicenter trial for Polyinosinic-Polycytidylic Acid Injection in the treatment of novel coronavirus pneumonia (COVID-19)
187	ChiCTR2000029779	2020/2/13	Wuxi People's Hospital	Study for the key issues of the diagnosis and treatment of novel coronavirus pneumonia (COVID-19) based on the medical imaging
188	ChiCTR2000029781	2020/2/13	Union Hospital affiliated to Tongji Medical College of Huazhong University of Science and Technology	A multicenter, randomized, open and controlled trial for the efficacy and safety of Kang-Bing-Du granules in the treatment of novel coronavirus pneumonia (COVID-19)

189	ChiCTR2000029782	2020/2/13	Zhejiang Provincial People's Hospital	Clinical study for the changes in mental state of medical staff in the department of radiotherapy in a comprehensive tertiary hospital in Zhejiang Province during the epidemic of novel coronavirus infection (COVID-19)
190	ChiCTR2000029803	2020/2/14	Renmin Hospital of Wuhan University	A prospective, randomized, open-label, parallel controlled trial for the preventive effect of hydroxychloroquine on medical personnel after exposure to COVID-19
191	ChiCTR2000029804	2020/2/14	Wuhan Jinyintan Hospital (Wuhan Infectious Diseases Hospital)	Clinical Application of ECMO in the Treatment of Patients with Very Serious Respiratory Failure due to novel Coronavirus Pneumonia (COVID-19)
192	ChiCTR2000029805	2020/2/14	Wuhan Jinyintan Hospital (Wuhan Infectious Diseases Hospital)	Analysis of clinical characteristics of severe novel coronavirus pneumonia (COVID-19)
193	ChiCTR2000029806	2020/2/14	Wuhan Jinyintan Hospital (Wuhan Infectious Diseases Hospital)	Immunomodulatory Therapy for Severe Novel Coronavirus Pneumonia (COVID-19)
194	ChiCTR2000029810	2020/2/14	Shenzhen Second People's Hospital	Clinical study of a novel high sensitivity nucleic acid assay for novel coronavirus pneumonia (COVID-19) based on CRISPR-cas protein
195	ChiCTR2000029815	2020/2/14	Guangdong Provincial Hospital of Chinese Medicine	Psychological survey of frontline medical staff in various regions of China during the epidemic of novel coronavirus pneumonia (COVID-19)
196	ChiCTR2000029821	2020/2/14	Deyang integrative medicine hospital	Based on Delphi Method to Preliminarily Construct a Recommended Protocol for the Prevention of Novel Coronavirus Pneumonia (COVID-19) in Deyang Area by Using Chinese Medicine Technology and its Clinical Application Evaluation
197	ChiCTR2000029829	2020/2/15	Three Gorges Central Hospital	Medical records based study for Heart-type fatty acid-binding protein on prognosis of novel coronavirus pneumonia (COVID-19)

198	ChiCTR2000029830	2020/2/15	The Fifth Affiliated Hospital of Sun Yat-Sen University	A study for the psychological status, social support, and care needs of tumor patients admitted to a general hospital during the novel coronavirus pneumonia (COVID-19) outbreak
199	ChiCTR2000029839	2020/2/15	Ningbo First Hospital	An observational study on the clinical characteristics, treatment and outcome of novel coronavirus pneumonia (COVID-19)
200	ChiCTR2000029849	2020/2/15	The First Affiliated Hospital of Zhengzhou University	Application of Regulating Intestinal Flora in the Treatment of Severe Novel Coronavirus Pneumonia (COVID-19)
201	ChiCTR2000029850	2020/2/15	The First Affiliated Hospital of Zhejiang University, State Key Laboratory for Diagnosis and Treatment of Infectious Diseases, National Clinical Research Center for Infectious Disease	Study for convalescent plasma treatment for severe patients with novel coronavirus pneumonia (COVID-19)
202	ChiCTR2000029851	2020/2/15	Zhongshan Hospital, Fudan University	A multicenter, randomized controlled trial for the efficacy and safety of Alpha lipoic acid (iv) in the treatment of patients of severe novel coronavirus pneumonia (COVID-19)
203	ChiCTR2000029853	2020/2/15	People's Hospital of Guangshan County	A randomized, open-label, controlled clinical trial for azvudine in the treatment of novel coronavirus pneumonia (COVID-19)
204	ChiCTR2000029865	2020/2/15	Union Hospital Affiliated to Tongji Medical College of Huazhong University of Science and Technology	Descriptive study for the clinical characteristics and outcomes of novel coronavirus pneumonia (COVID-19) in cardiovascular patients
205	ChiCTR2000029866	2020/2/15	Enze Hospital of Taizhou Enze Medical Center (Group)/Taizhou Hospital of Zhejiang Province	Early warning prediction of patients with severe novel coronavirus pneumonia (COVID-19) based on multiomics
206	ChiCTR2000029867	2020/2/15	Beijing You'an Hospital, Capital Medical University	The efficacy and safety of carrimycin treatment in patients with novel coronavirus infectious disease (COVID-19): a multicenter, randomized, open- label controlled trial
207	ChiCTR2000029868	2020/2/15	Ruijin Hospital, Shanghai Jiaotong University School of Medicine	Hydroxychloroquine treating novel coronavirus pneumonia (COVID-19): a multicenter, randomized controlled trial

208	ChiCTR2000029870	2020/2/15	Jiangsu Institute of Parasitic Diseases	Evaluation of Rapid Diagnostic Kit (IgM/IgG) for Novel Coronavirus Pneumonia (COVID-19)
209	ChiCTR2000029883	2020/2/16	Tongji Hospital, Tongji Medical College, Huazhong University of Science and Technology	A comparative study on the sensitivity of nasopharyngeal and oropharyngeal swabbing for the detection of influenza virus by real-time PCR
210	ChiCTR2000029898	2020/2/16	Peking University Third Hospital	A Randomized, Open-label, Parallel, Controlled Trial for Evaluation of the Efficacy and Safety of Chloroquine Phosphate in the treatment of Severe Patients with Novel Coronavirus Pneumonia (COVID-19)
211	ChiCTR2000029899	2020/2/16	Peking University Third Hospital	A Prospective, Multicenter, Open, Randomized Controlled Trial for Evaluation of the Efficacy and Safety of Chloroquine Phosphate and Hydroxychloroquine Sulfate in the treatment of Patients With Mild and Common type novel coronavirus pneumonia (COVID-19)
212	ChiCTR2000029900	2020/2/16	Renmin Hospital of Wuhan University	Research for Risks Associated with Novel Coronavirus Pneumonia (COVID-19) in the Hospital Workers and Nosocomial Prevention and Control Strategy
213	ChiCTR2000029905	2020/2/16	Zhongnan Hospital of Wuhan University	A medical records based study of novel coronavirus pneumonia (COVID-19) and influenza virus co-infection
214	ChiCTR2000029907	2020/2/16	West China Hospital, Sichuan University	Study for construction and assessment of early warning score of the clinical risk of novel coronavirus (COVID-19) infected patients
215	ChiCTR2000029935	2020/2/17	HwaMei Hospital, University of Chinese Academy of Sciences	A Single-arm Clinical Trial for Chloroquine Phosphate in the treatment of Novel Coronavirus Pneumonia 2019 (COVID-19)
216	ChiCTR2000029939	2020/2/17	HwaMei Hospital, University of Chinese Academy of Sciences	A Single-blind, Randomized, Controlled Clinical Trial for Chloroquine Phosphate in the Treatment of Novel Coronavirus Pneumonia 2019 (COVID-19)

217	ChiCTR2000029949	2020/2/17	Emergency Department of Zhongnan hospital of Wuhan University	A Medical Records Based Study for the Effectiveness of Extracorporeal Membrane Oxygenation in Patients with Severe Novel Coronavirus Pneumonia (COVID-19)
218	ChiCTR2000029952	2020/2/17	Chongqing Three Gorges Central Hospital	Medical records based study for epidemiological and clinical characteristics of 2019 novel coronavirus pneumonia (COVID-19) in Chongqing
219	ChiCTR2000029953	2020/2/17	Emergency Department of Zhongnan hospital of Wuhan University	Construction and Analysis of Prognostic Predictive Model of Novel Coronavirus Pneumonia (COVID-19)
220	ChiCTR2000029955	2020/2/17	West China Second University Hospital, Sichuan University	Evaluation of myocardial injury of novel coronavirus pneumonia (COVID-19) assessed by multimodal MRI imaging
221	ChiCTR2000029958	2020/2/17	Zhongnan Hospital of Wuhan University	A medical records based study for the clinical characteristics of anesthesia novel coronavirus pneumonia (COVID-19) patients during perioperative period and assessment of infection and mental health of Anesthesiology Department
222	ChiCTR2000029959	2020/2/17	West China Second University Hospital, Sichuan University	Clinical observation and research of Severe acute respiratory syndrome coronavirus 2(COVID-19) infection in perinatal newborns
223	ChiCTR2000029972	2020/2/17	Tongji Hospital, Tongji Medical College, Huazhong University of Science and Technology	A randomized controlled trial for the Efficacy of Ultra Short Wave Electrotherapy in the treatment of Novel Coronavirus Pneumonia (COVID-19)
224	ChiCTR2000029974	2020/2/18	Qilu Hospital of Shandong University	A prospective, multicenter, open-label, randomized, parallel-controlled trial for probiotics to evaluate efficacy and safety in patients infected with 2019 novel coronavirus pneumonia (COVID-19)
225	ChiCTR2000029975	2020/2/18	The First Hospital of Jilin University	Single arm study for exploration of chloroquine phosphate aerosol inhalation in the treatment of novel coronavirus pneumonia (COVID-19)

226	ChiCTR2000029981	2020/2/18	Tongji Hospital, Tongji Medical College, Huazhong University of Science and Technology	Study for nucleic acid detection of novel coronavirus pneumonia (COVID-2019) in female vaginal secretions
227	ChiCTR2000029982	2020/2/18	Union Hospital, Tongji Medical College, Huazhong University of Science and Technology	Study for using multiomics in the diagnosis and treatment of novel coronavirus pneumonia (COVID-19)
228	ChiCTR2000029985	2020/2/18	Sichuan Academy of Medical Sciences & Sichuan Provincial People's Hospital	Study for mental health status and influencing factors of nurses during epidemic prevention of novel coronavirus pneumonia (COVID-19)
229	ChiCTR2000029988	2020/2/18	Zhongnan Hospital of Wuhan University	Clinical Study of Chloroquine Phosphate in the Treatment of Severe Novel Coronavirus Pneumonia (COVID-19)
230	ChiCTR2000029989	2020/2/18	Chinese PLA General Hospital	A randomized controlled Trial for therapeutic efficacy of Recombinant Human Interferon alpha 1b Eye Drops in the treatment of elderly with novel coronavirus pneumonia (COVID-19)
231	ChiCTR2000029990	2020/2/18	institute of basic medicine, Chinese Academy of medical sciences	Clinical trials of mesenchymal stem cells for the treatment of pneumonitis caused by novel coronavirus pneumonia (COVID-19)
232	ChiCTR2000029992	2020/2/18	Zhongshan Hospital Affiliated to Xiamen University	A prospective, randomized, open label, controlled trial for chloroquine and hydroxychloroquine in patients with severe novel coronavirus pneumonia (COVID-19)
233	ChiCTR2000029995	2020/2/19	Shanghai General hospital of Shanghai Jiaotong University	Study on anxiety of different populations under novel coronavirus (COVID-19) infection
234	ChiCTR2000029996	2020/2/19	Beijing Chaoyang Hospital, Capital Medical University	
235	ChiCTR2000029999	2020/2/19	Shanghai 10th People's Hospital, Tongji University	A clinical study for probiotics in the regulation of intestinal function and microflora structure of novel coronavirus pneumonia (COVID-19)
236	ChiCTR2000030001	2020/2/19	Heilongjiang Province hospital	The efficacy and safety of Triazavirin for 2019 novel coronary pneumonia (COVID-19): a multicenter, randomized, double blinded, placebo-controlled trial

237	ChiCTR2000030002	2020/2/19	The First Affiliated Hospital of University of science and technology of China (Anhui Provincial Hospital)	Clinical study of novel NLRP Inflammasome inhibitor (Tranilast) in the treatment of novel coronavirus pneumonia (COVID-19)
238	ChiCTR2000030004	2020/2/19	West China Hospital, Sichuan University	Effect of Novel Coronavirus Pneumonia (COVID-19) on the Mental Health of College Students
239	ChiCTR2000030005	2020/2/19	Tongji Hospital, Tongji Medical College, Huazhong University of Science and Technology	Nucleic acid analysis of novel coronavirus (COVID-19) in morning salivary samples and pharyngeal swabs-a prospectively diagnostic test
240	ChiCTR2000030006	2020/2/19	Union Hospital, Tongji Medical College, Huazhong University of Science and Technology	A randomized controlled trial for the efficacy of ozonated autohemotherapy in the treatment of Novel Coronavirus Pneumonia (COVID-19)
241	ChiCTR2000030007	2020/2/19	Multicenter randomized controlled trial for rhG-CSF in the treatment of novel coronavirus pneumonia (COVID-19)	Multicenter randomized controlled trial for rhG-CSF in the treatment of novel coronavirus pneumonia (COVID-19)
242	ChiCTR2000030008	2020/2/19	The Fifth Affiliated Hospital of Sun Yat-Sen University	Correalation between anxiety as well as depression and gut microbiome among staff of hospital during the novel coronavirus pneumonia (COVID-19) outbreak
243	ChiCTR2000030010	2020/2/19	Wuhan Jinyintan Hospital (Wuhan Infectious Diseases Hospital)	A randomized, double-blind, parallel-controlled, trial to evaluate the efficacy and safety of anti-SARS-CoV-2 virus inactivated plasma in the treatment of severe novel coronavirus pneumonia patients (COVID-19)
244	ChiCTR2000030012	2020/2/19	The First People's Hospital of Chenzhou, Institute of Translational Medicine,University of South China	Development of anti-2019-nCoV therapeutic antibody from the recovered novel coronavirus pneumonia patients (COVID-19)
245	ChiCTR2000030013	2020/2/20	Chinese PLA General Hospital	A prospective clinical study for recombinant human interferon alpha 1b spray in the prevention of novel coronavirus (COVID-19) infection in highly exposed medical staffs.
246	ChiCTR2000030014	2020/2/20	Wuhan Fourth Hospital	Effect of early pulmonary training on lung function and quality of life for novel coronavirus pneumonia (COVID-19) patients

247	ChiCTR2000030015	2020/2/20	Tongji Hospital,Tongji Medical College, Huazhong University of Science and Technology	Study for the correlation between the incidence and outcome of novel coronary pneumonia (COVID-2019) and ovarian function in women
248	ChiCTR2000030016	2020/2/20	Beihai People's Hospital/Department of respiratory medicine, the First Affiliated Hospital of Guangxi Medical University	Basic and clinical study of inhalation of inactivated mycobacterium vaccine in the treatment of Novel coronavirus pneumonia (COVID-19)
249	ChiCTR2000030017	2020/2/20	Renmin Hospital of Wuhan University	Feature of Multiple Organs in Ultrasound Investigation for Clinical Management and Prognostic Evaluation of Novel Coronavirus Pneumonia (COVID-19)
250	ChiCTR2000030019	2020/2/20	Zhongshan Hospital Fudan University	The COVID-19 Mobile Health Study (CMHS), a large-scale clinical observational registration study using nCapp
251	ChiCTR2000030020	2020/2/20	Second Hospital of University of South China, Hengyang	The clinical application and basic research related to mesenchymal stem cells to treat novel coronavirus pneumonia (COVID-19)
252	ChiCTR2000030021	2020/2/20	Department of Respiratory and Critical Care Medicine, Tongji Hospital, Tongji Medical College, Huazhong University of Science and Technology	A medical records based analysis for the clinical characteristics of novel coronavirus pneumonia (COVID-19) in immunocompromised patients
253	ChiCTR2000030028	2020/2/20	West China Hospital, Sichuan University	Clinical comparative study of PD-1 mAb in the treatment of severe and critical patients with novel coronavirus pneumonia (COVID-19)
254	ChiCTR2000030029	2020/2/21	The First Affiliated Hospital of Zhejiang University School of Medicine	A multi-center study on the efficacy and safety of suramin sodium in adult patients with novel coronavirus pneumonia (COVID-19)
255	ChiCTR2000030030	2020/2/21	Tongji Hospital, Tongji Medical College, Huazhong University of Science and Technology	A medical records based study for acute kidney injury in novel coronavirus pneumonia (COVID-19)
256	ChiCTR2000030031	2020/2/21	The Sixth Affiliated Hospital of Guangzhou Medical University (Qingyuan People's Hospital)	A randomized, double-blind, parallel, controlled trial for comparison of phosphoric chloroquine combined with standard therapy and standard therapy in mild/common patients with novel coronavirus pneumonia

				(COVID-19)
257	ChiCTR2000030032	2020/2/21	Xi'an Chest Hospital	A medical records based study for ultrasonographic manifestations of new type of novel coronavirus pneumonia (covid-19) in non-critical stage of pulmonary lesions
258	ChiCTR2000030039	2020/2/21	Affiliated Hospital of Xuzhou Medical University	Clinical study for infusing convalescent plasma to treat patients with new coronavirus pneumonia (COVID-19)
259	ChiCTR2000030041	2020/2/21	Zhongnan Hospital of Wuhan University	A single-arm, single-center clinical trial for Azivudine tablets in the treatment of adult novel coronavirus pneumonia (COVID-19)
260	ChiCTR2000030046	2020/2/21	First People's Hospital of Jiangxia District, Wuhan (Union Jiangnan Hospital)	A single arm trial to evaluate the efficacy and safety of anti-2019-nCoV inactivated convalescent plasma in the treatment of novel coronavirus pneumonia patient (COVID-19)
261	ChiCTR2000030054	2020/2/22	Zhongshan Hospital Affiliated to Xiamen University	A prospective, open label, randomized, control trial for chloroquine or hydroxychloroquine in patients with mild and common novel coronavirus pulmonary (COVIP-19)
262	ChiCTR2000030055	2020/2/22	The First Affiliated Hospital of Guangzhou Medical University	Multicenter study for the treatment of Dipyridamole with novel coronavirus pneumonia (COVID-19)
263	ChiCTR2000030056	2020/2/22	Union Hospital, Tongji Medical College, Huazhong University of Science & Technology	Study for the effect of early endotracheal intubation on the outcome of novel coronavirus pneumonia (COVID-19) patients
264	ChiCTR2000030058	2020/2/22	Renmin Hospital of Wuhan University	A multicenter, randomized, double-blind, controlled clinical trial for leflunomide in the treatment of novel coronavirus pneumonia (COVID-19)
265	ChiCTR2000030084	2020/2/22	The First Hospital of Shanxi Medical University	A multicenter study for efficacy of intelligent psychosomatic adjustment system intervention in the treatment of novel coronavirus pneumonia (COVID-19) patients with mild to moderate anxiety and depression

266	ChiCTR2000030086	2020/2/22	Union Hospital, Tongji Medical College, Huazhong University of Science & Technology	A medical records based study for the impacting on medical providers' infection rate and mental health after performing different anesthesia schemes in cesarean section for novel coronavirus pneumonia (COVID-19) puerperae
267	ChiCTR2000030087	2020/2/22	Union Hospital, Tongji Medical College, Huazhong University of Science and Technology	Clinical study for the diagnostic value of pulmonary ultrasound for novel coronavirus pneumonia (COVID-19)
268	ChiCTR2000030088	2020/2/23	The Sixth Medical Center of PLA General Hospital	Umbilical cord Wharton's Jelly derived mesenchymal stem cells in the treatment of severe novel coronavirus pneumonia (COVID-19)
269	ChiCTR2000030089	2020/2/23	Shanghai Changzheng Hospital	A clinical study for the efficacy and safety of Adalimumab Injection in the treatment of patients with severe novel coronavirus pneumonia (COVID-19)
270	ChiCTR2000030090	2020/2/23	West China Hospital, Sichuan University	A Prospective Randomized Controlled Trial for Home Exercise Prescription Intervention During Epidemic of Novel Coronary Pneumonia (COVID-19) in College Students
271	ChiCTR2000030091	2020/2/23	West China Hospital, Sichuan University	A prospective randomized controlled trial for the home exercise prescription intervention in nursing students during epidemic of novel coronary pneumonia (COVID-19)
272	ChiCTR2000030092	2020/2/23	Union Hospital, Tongji Medical College, Huazhong University of Science & Technology	Assessment of cardiac function in patients with Novel Coronavirus Pneumonia (COVID-19) by echocardiography and its new techniques
273	ChiCTR2000030093	2020/2/23	The Second Hospital of Shanxi Medical University	Study for application of simplified cognitive-behavioral therapy for related emergency psychological stress reaction of medical providers working in the position of treatment and control of novel coronavirus pneumonia (COVID-19)
274	ChiCTR2000030094	2020/2/23	Chengdu University of Traditional Chinese Medicine	Effects of novel coronavirus pneumonia (COVID-19) on menstruation, TCM body construction and psychological state for female at different ages

275	ChiCTR2000030095	2020/2/23	The First Affiliated Hospital of Nanchang University	A medical records based study for optimization and evaluation of the comprehensive diagnosis and treatment of novel coronavirus pneumonia (COVID-19) and the assessment of risk factors for severe pneumonia
276	ChiCTR2000030096	2020/2/23	Peking University First Hospital	Study for establishment of correlation between virological dynamics and clinical features in noveal coronavirus pneumonia (COVID-19)
277	ChiCTR2000030113	2020/2/23	Clinical study for safety and efficacy of Favipiravir in the treatment of novel coronavirus pneumonia (COVID-19) with poorly responsive ritonavir/ritonavir	Randomized controlled trial for safety and efficacy of Favipiravir in the treatment of novel coronavirus pneumonia (COVID-19) with poorly responsive ritonavir/ritonavir
278	ChiCTR2000030114	2020/2/23	Peking University People's Hospital	Lung ultrasound in the diagnosis, treatment and prognosis of pulmonary lesions of novel coronavirus pneumonia (COVID-19)
279	ChiCTR2000030115	2020/2/23	Tongji Hospital, Tongji Medical College, Huazhong University of Science and Technology	A clinical research for the changes of Blood cortisol ACTH level and adrenal morphology in blood cortisol to guide the application of individualized hormone in severe novel coronavirus pneumonia (COVID-19) patients
280	ChiCTR2000030116	2020/2/23	The First Affiliated Hospital of Nanchang University	Safety and effectiveness of human umbilical cord mesenchymal stem cells in the treatment of acute respiratory distress syndrome of severe novel coronavirus pneumonia (COVID-19)
281	ChiCTR2000030138	2020/2/24	Chinese PLA General Hospital	Clinical Trial for Human Mesenchymal Stem Cells in the Treatment of Severe Novel Coronavirus Pneumonia (COVID-19)
282	ChiCTR2000030163	2020/2/24	Yueyang Hospital of Integrated Traditional Chinese Medicine and Western Medicine Affiliated to Shanghai University of Tranditional Medicine	A Medical Based Retrospective Real World Study for Assessment of Effectiveness of Comprehensive Traditional Chinese Medicine in the treatment of Novel Coronavirus Pneumonia (COVID-19)
283	ChiCTR2000030164	2020/2/24	Union Hospital, Tongji Medical College, Huazhong University of Science and Technology	A cross-sectional study of novel coronavirus pneumonia (COVID-19) patients in ICU

284	ChiCTR2000030165	2020/2/24	Haihe hospital, Tianjin University	Clinical study for ozonated autohemotherapy in the treatment of Novel Coronavirus Pneumonia (COVID-19)
285	ChiCTR2000030167	2020/2/24	Renmin Hospital of Wuhan University	Clinical Trial for Recombinant Human Interleukin-2 in the Treatment of Novel Coronavirus Pneumonia (COVID-19)
286	ChiCTR2000030170	2020/2/24	Shanghai Public Health Clinical Center	Study for safety and efficacy of Jakotinib hydrochloride tablets in the treatment severe and acute exacerbation patients of novel coronavirus pneumonia (COVID-19)
287	ChiCTR2000030173	2020/2/24	Hu'nan Yuanpin Cell Biotechnology Co., Ltd/Nanhua Hospital Affiliated to Nanhua University	Key techniques of umbilical cord mesenchymal stem cells for the treatment of novel coronavirus pneumonia (COVID-19) and clinical application demonstration
288	ChiCTR2000030179	2020/2/24	The First Affiliated Hospital of Nanchang University	Experimental study of novel coronavirus pneumonia rehabilitation plasma therapy severe novel coronavirus pneumonia (COVID-19)
289	ChiCTR2000030185	2020/2/24	Department of Critical Care Medicine, West China Hospital of Sichuan University	The Value of Critical Care Ultrasound in Rapid Screening, Diagnosis, Evaluation of Effectiveness and Intensive Prevention of Novel Coronavirus Pneumonia (COVID-19)
290	ChiCTR2000030187	2020/2/24	Jingzhou First People's Hospital	Clinical study for Lopinavir and Ritonavir in the treatment of novel coronavirus pneumonia (COVID-19)
291	ChiCTR2000030196	2020/2/25	Central South Hospital, Wuhan University	A multicenter, single arm, open label trial for the efficacy and safety of CMAB806 in the treatment of cytokine release syndrome of novel coronavirus pneumonia (COVID-19)
292	ChiCTR2000030198	2020/2/25	West China Hospital, Sichuan University	Clinical research of pulmonary rehabilitation in survivors due to severe or critial novel coronavirus pneumonia (COVID-19)
293	ChiCTR2000030218	2020/2/25	Fifth People's Hospital of Ganzhou	Study of Pinavir / Ritonavir Tablets (Trade Name: Kelizhi) Combined with Xiyanping Injection for Novel Coronavirus Pneumonia (COVID-19)

294	ChiCTR2000030223	2020/2/25	JC School Of Public Health And Primary Care, The Chinese University Of Hong Kong	Quality of life among Chinese residents during and after novel coronavirus pneumonia (COVID19) outbreak: an online survey
295	ChiCTR2000030226	2020/2/25	The Fourth Hospital of Hebei Medical University	The treatment status and risk factors related to prognosis of hospitalized patients with novel coronavirus pneumonia (COVID-19) in intensive care unit, Hebei, China: a descriptive study
296	ChiCTR2000030253	2020/2/26	Ganzi Hospital of West China Hospital, Sichuan University	Exploration and Research for a new method for detection of novel coronavirus (COVID-19) nucleic acid
297	ChiCTR2000030254	2020/2/26	Zhongnan Hospital of Wuhan University	A randomized, open-controlled trial for farpiravir tablets in the treatment of novel coronavirus pneumonia (COVID-19)
298	ChiCTR2000030255	2020/2/26	Shanghai University of Traditional Chinese Medicine	Efficacy and Safety of Jing-Yin Granule in the treatment of novel coronavirus pneumonia (COVID-19) wind-heat syndrome
299	ChiCTR2000030256	2020/2/26	Daping Hospital, Army Medical University	Medical records based study for epidemic and clinical characteristics of hospitalized patients with novel coronavirus pneumonia (COVID-19) in Huoshenshan hospital, Wuhan
300	ChiCTR2000030257	2020/2/26	Guangdong Second Provincial General Hospital/Puren Hospital of Wuhan City	The coagulation function of novel coronavirus pneumonia (COVID-19) patients
301	ChiCTR2000030258	2020/2/26	The Fourth Affiliated Hospital of Harbin Medical University	A multicenter, randomized, controlled trial for efficacy and safety of hydrogen inhalation in the treatment of novel coronavirus pneumonia (COVID-19) patients
302	ChiCTR2000030259	2020/2/26	Shanghai Changzheng Hospital	Evaluation Danorevir sodium tablets combined with ritonavir in the treatment of novel coronavirus pneumonia (COVID-19): a randomized, open and controlled trial
303	ChiCTR2000030260	2020/2/26	Ganzi Hospital of West China Hospital, Sichuan University	Clinical study for individualized nutritional assessment and supportive treatment of novel coronavirus pneumonia (COVID-19) patients in Tibetan Plateau

304	ChiCTR2000030261	2020/2/26	Wuxi Fifth People's Hospital	A study for the key technology of mesenchymal stem cells exosomes atomization in the treatment of novel coronavirus pneumonia (COVID-19)
305	ChiCTR2000030262	2020/2/26	Shanghai Public Health Clinical Center	Clinical study for combination of anti-viral drugs and type I interferon and inflammation inhibitor TFF2 in the treatment of novel coronavirus pneumonia (COVID-19)
306	ChiCTR2000030263	2020/2/26	Beijing Geriatric Hospital	Investigation and analysis of psychological status of hospital staff during the novel coronavirus pneumonia (COVID-19) epidemic
307	ChiCTR2000030264	2020/2/26	Department of critical care, Zhongnan Hospital of Wuhan University	ICU healthcare personnel burnout investigation during the fight against novel coronavirus pneumonia (COVID-19)
308	ChiCTR2000030265	2020/2/26	The First Affiliated Hospital of Harbin Medical University	Clinical research program of continuous renal replacement therapy with adsorption filter for the treatment of the novel coronavirus pneumonia (COVID-19)
309	ChiCTR2000030283	2020/2/27	Xianning Central Hospital	Correlation between imaging characteristics and laboratory tests of new coronavirus pneumonia (COVID-19)
310	ChiCTR2000030290	2020/2/27	Xiamen University	Health related quality of life and its influencing factors among front line nurses caring patients with new coronavirus pneumonia (COVID-19) from two hospitals in China
311	ChiCTR2000030293	2020/2/27	Shanghai Public Health Clinical Center	Clinical observation and research plan of novel coronavirus pneumonia (COVID-19) patients
312	ChiCTR2000030304	2020/2/28	The Third Affiliated Hospital of Sun Yat-sen University	Protective factors of mental resilience in first-line nurses with novel coronavirus pneumonia (COVID-19)
313	ChiCTR2000030312	2020/2/28	First People's Hospital of Jiangxi District	A single-center, open-label and single arm trial to evaluate the efficacy and safety of anti-SARS-CoV-2 inactivated convalescent plasma in the treatment of novel coronavirus pneumonia (COVID-19) patient

314	ChiCTR2000030317	2020/2/28	West China Hospital, Sichuan University	Clinical study for a new type of Gastroscope isolation mask for preventing and controlling the novel coronavirus pneumonia (COVID-19) Epidemic period
315	ChiCTR2000030322	2020/2/28	Xinyang Central Hospital	Identification and Clinical Treatment of Severe novel coronavirus pneumonia (COVID-19) Patients
316	ChiCTR2000030325	2020/2/28	Xiangyang Central Hospital, Affiliated Hospital of Hubei University of Arts and Sciences	A survey for mental health of first-line medical providers and to construction of crisis intervention model for novel coronavirus pneumonia (COVID-19) in Xiangyang
317	ChiCTR2000030327	2020/2/28	The Second Affiliated hospital of Anhui Medical University	Analysis of clinical characteristics of novel coronavirus pneumonia (COVID-19)
318	ChiCTR2000030331	2020/2/29	The First Affiliated Hospital of University of science and technology of China (Anhui Provincial Hospital)	Construction of a Bio information platform for novel coronavirus pneumonia (COVID-19) patients follow-up in Anhui
319	ChiCTR2000030333	2020/2/29	Tongji Hospital of Tongji Medical College; Huazhong Science and Technology University	A randomized, open-label controlled trial for the efficacy and safety of Pirfenidone in patients with severe and critical novel coronavirus pneumonia (COVID-19)
320	ChiCTR2000030334	2020/2/29	College of Life Sciences, Xianlin Campus, Nanjing University/Nanjing Second Hospital	microRNA as a marker for early diagnosis of novel coronavirus infection (COVID-19)
321	ChiCTR2000030363	2020/2/29	Tongji Hospital, Tongji Medical College, Huazhong University of Science and Technology	Novel Coronavirus Infected Disease (COVID-19) in children: epidemiology, clinical features and treatment outcome
322	ChiCTR2000030381	2020/2/29	First people's hospital of Jiangxi district, Wuhan	A randomized, open-label, controlled and single-center trial to evaluate the efficacy and safety of anti-SARS-CoV-2 inactivated convalescent plasma in the treatment of novel coronavirus pneumonia (COVID-19) patient
323	ChiCTR2000030382	2020/2/29	Hubei Provincial Hospital of TCM	Construction and application of non-contact doctor-patient interactive diagnosis and treatment mode of moxibustion therapy for novel coronary pneumonia (COVID-19) based on mobile internet

324	ChiCTR2000030387	2020/3/1	Tongji Hospital of Tongji University	Clinical observation and research of multiple organs injury in severe patients with novel coronavirus pneumonia (COVID-19)
325	ChiCTR2000030390	2020/3/1	Key Laboratory of Ministry of Industry and Information Technology of Biomedical Engineering and Translational Medicine	Research and Development of Diagnostic Assistance Decision Support System for novel coronavirus pneumonia (COVID-19) Based on Big Data Technology
326	ChiCTR2000030391	2020/3/1	Lishui Central Hospital	A medical records based analysis for antiviral therapy effect on novel coronavirus pneumonia COVID-19 patients
327	ChiCTR2000030398	2020/3/1	Wuhan Jinyintan Hospital (Wuhan Infectious Diseases Hospital)	A randomized, double-blind, placebo-controlled trial for evaluation of the efficacy and safety of bismuth potassium citrate capsules in the treatment of patients with novel coronavirus pneumonia (COVID-19).
328	ChiCTR2000030417	2020/3/1	Harbin Peiyou Jiandi Biotechnology Co., Ltd/Heilongjiang Social Rehabilitation Hospital	Efficacy and safety of chloroquine phosphate inhalation combined with standard therapy in the treatment of novel coronavirus pneumonia (COVID-19)
329	ChiCTR2000030424	2020/3/1	He'nan Provincial People's Hospital	A single-center, single-arm clinical trial for azvudine in the treatment of novel coronavirus pneumonia (COVID-19）
330	ChiCTR2000030436	2020/3/1	Shanghai Sixth People's Hospital	Application of flash glucose monitoring to evaluate the effect of blood glucose changes on prognosis in patients with novel coronavirus pneumonia (COVID-19)
331	ChiCTR2000030453	2020/3/2	The First Affiliated Hospital of Wenzhou Medical University	Clinical study for the effects of ACEIs/ARBs on the infection of novel coronavirus pneumonia (CoVID-19)
332	ChiCTR2000030464	2020/3/2	The Third Xiangya Hospital, Central South University	Study on the clinical characteristics of novel coronavirus pneumonia (COVID-19)
333	ChiCTR2000030471	2020/3/2	Maoming People's Hospital	Efficacy and safety of lipoic acid injection in reducing the risk of progression in common patients with novel coronavirus pneumonia (COVID-19)

334	ChiCTR2000030472	2020/3/3	Shenyang Sixth People's Hospital	An open and controlled clinical study to evaluate the efficacy and safety of Ganovo combined with ritonavir in the treatment of novel coronavirus pneumonia (COVID-19)
335	ChiCTR2000030475	2020/3/3	Peking Union Medical College Hospital, Chinese Academy of Medical Sciences and Peking Union Medical College	Cytosorb in Treating Critically Ill Hospitalized Adult Patients with novel coronavirus pneumonia (COVID-19)
336	ChiCTR2000030476	2020/3/3	Cancer Institute, Longhua Hospital, Shanghai University of Traditional Chinese Medicine	Study for emotion regulation of traditional Chinese medicine assists for the rehabilitation of patients with novel coronaviruse pneumonia (COVID-19)
337	ChiCTR2000030477	2020/3/3	Peking Union Medical College Hospital, Chinese Academy of Medical Sciences and Peking Union Medical College	oXiris Membrane in Treating Critically Ill Hospitalized Adult Patients with novel coronavirus pneumonia (COVID-19)
338	ChiCTR2000030480	2020/3/3	Department of Respiratory and Critical Care Medicine, Tongji Hospital of Tongji Medical College, Huazhong University of Science and Technology	Randomized, open, blank controlled trial for the efficacy and safety of recombinant human interferon alpha 1beta in the treatment of Wuhan patients with novel coronavirus pneumonia (COVID-19)
339	ChiCTR2000030481	2020/3/3	Zhongnan Hospital of Wuhan University	The clinical value of corticosteroid therapy timing in the treatment of novel coronavirus pneumonia (COVID-19): a prospective randomized controlled trial
340	ChiCTR2000030482	2020/3/3	Peking University First Hospital/China Cardiovascular Association	A Multicenter, Long- term Follow-up and Registration Study for Myocardial Injury and Prognosis of Novel coronavirus pneumonia (COVID-19)
341	ChiCTR2000030484	2020/3/3	Beijing Darwin Cell Biotechnology Co., Ltd/Hubei Shiyan Taihe hospital	HUMSCs and Exosomes Treating Patients with Lung Injury following Novel Coronavirus Pneumonia (COVID-19)
342	ChiCTR2000030485	2020/3/3	Eergency / Intensive Care Department of Tongji Hospital, Tongji Medical College,Huazhong University of Science and Technology	Study for timing of mechanical ventilation for critically ill patients with novel coronavirus pneumonia (COVID-19): A medical records based retrospective Cohort study

343	ChiCTR2000030487	2020/3/4	The First Affiliated Hospital of HeNan University of CM	A single-center, single-arm clinical trial for azvudine in the treatment of novel coronavirus pneumonia (COVID-19）
344	ChiCTR2000030489	2020/3/4	Department of Ophthalmology, Tongji Hospital, Tongji Medical College, Huazhong University of Science and Technology	Study for the route of ocular surface transmission of novel coronavirus pneumonia (COVID-19) infection and related eye diseases
345	ChiCTR2000030490	2020/3/4	Changhai Hospital/Huo-Shen-Shan Hospital	To evaluate the efficacy and safety of diammonium glycyrrhizinate enteric-coated capsules combined with hydrogen-rich water in the treatment of novel coronavirus pneumonia (COVID-19)
346	ChiCTR2000030491	2020/3/4	West China Hospital, Sichuan University	A medical records based study for Comparing Differences of Clinical Features and Outcomes of Novel Coronavirus Pneumonia (COVID-19) Patients between Sichuan Province and Wuhan City
347	ChiCTR2000030492	2020/3/4	The First Affiliated Hospital of Hu'nan University of traditional Chinese Medicine	Retrospective study for integrate Chinese and conventional medicine treatment of novel coronavirus pneumonia (COVID-19) in Hu'nan province
348	ChiCTR2000030493	2020/3/4	Renmin Hospital of Wuhan University	Survey for sleep, anxiety and depression status of Chinese residents during the outbreak of novel coronavirus infected disases (COVID-19)
349	ChiCTR2000030494	2020/3/4	West China Hospital, Sichuan University	Early risk stratification of the Novel coronavirus infected diseases (COVID-19): a multicenter retrospective study (ERS-COVID-19 study)
350	ChiCTR2000030503	2020/3/5	The First Affiliated Hospital of Zhejiang University, State Key Laboratory for Diagnosis and Treatment of Infectious Diseases, National Clinical Research Center for Infectious Disease	Extracorporeal blood purification therapy using Li's Artifical Liver System for patients with severe novel coronavirus pneumonia (COVID19) patient
351	ChiCTR2000030539	2020/3/6	Guangzhou Eighth People's Hospital	Study for clinical oral characteristics of patients with novel coronavirus pneumonia (COVID-19) and Effect of 3% hydrogen peroxide gargle on the Intraoral novel coronavirus

352	ChiCTR2000030540	2020/3/6	Tongji Hospital Affiliated to Tongji Medical College of Huazhong University of science and technology	Research for the mechanism of improvement of novel coronavirus pneumonia (COVID-19) patients' pulmonary exudation by continuous renal replacement therapy
353	ChiCTR2000030541	2020/3/6	Shijiazhuang Medical College/Xiamen Medical College	Novel coronavirus pneumonia (COVID-19) epidemic survey of medical students in various provinces and municipalities throughout the country
354	ChiCTR2000030542	2020/3/6	West China Hospital, Sichuan University	A clinical study about the diagnosis and prognosis evaluation of novel coronacirus pneumonia (COVID-19) based on viral genome, host genomic sequencing, relative cytokines and other laboratory indexes.
355	ChiCTR2000030543	2020/3/6	Central Theater General Hospital of PLA	Detection of coronavirus in simultaneously collecting tears and throat swab samples collected from the patients with novel coronavirus pneumonia (COVID-19)
356	ChiCTR2000030544	2020/3/6	Emergency / Intensive Care Department of Tongji Hospital, Tongji Medical College,Huazhong University of Science and Technology	Study for the risk factors of critically ill patients with novel coronavirus pneumonia (COVID-19)
357	ChiCTR2000030556	2020/3/7	Anhui Cancer Hospital	Clinical study of nano-nose and its extended technology in diagnosis of novel coronavirus pneumonia (COVID-19)
358	ChiCTR2000030557	2020/3/7	The Third Affiliated Hospital of the Second Military Medical University	A retrospective study on virus typing, Hematological Immunology and case Review of novel coronavirus infected and convalescent patients (COVID-19)
359	ChiCTR2000030559	2020/3/7	The First Affiliated Hospital of Zhejiang University, State Key Laboratory for Diagnosis and Treatment of Infectious Diseases, National Clinical Research Center for Infectious Disease	Safety of artificial liver cluster nursing in critically ill patients with novel coronavirus pneumonia (COVID-19)
360	ChiCTR2000030564	2020/3/7	Tongji Hospital, Tongji Medical College, Huazhong University of Science and Technology	Psychological Intervention of Children with Novel Coronavirus Disease (COVID-19)

361	ChiCTR2000030578	2020/3/8	Shandong Provincial Hospital	Clinical Prediction and Intervention of Pulmonary Function Impairment in Patients with Novel Coronavirus Pneumonia (COVID-19)
362	ChiCTR2000030579	2020/3/8	Shandong Provincial Hospital	Risk Factors for Outcomes of Novel Coronavirus Pneumonia (COVID-19)
363	ChiCTR2000030580	2020/3/8	Shanghai General Hospital	Efficacy and safety of adamumab combined with tozumab in severe and critical patients with novel coronavirus pneumonia (COVID-19)
364	ChiCTR2000030581	2020/3/8	West China Hospital, Sichuan University	The immediate psychological impact of novel coronavirus pneumonia (COVID-19) outbreak on medical students in anesthesiology and how the cope
365	ChiCTR2000030593	2020/3/8	The First Affiliated Hospital, College of Medicine of Zhejiang University	Novel coronavirus pneumonia (COVID-19) antiviral related liver dysfunction: a multicenter, retrospective, observational study
366	ChiCTR2000030627	2020/3/8	The First Affiliated Hospital of Zhengzhou University	Study for using the healed novel coronavirus pneumonia (COVID-19) patients plasma in the treatment of severe critical cases
367	ChiCTR2000030679	2020/3/9	Wuhan Children's Hospital (Wuhan Maternal and Child Healthcare Hospital), Tongji Medical College, Huazhong University of Science & Technology	Cohort study of Novel Coronavirus Pneumonia (COVID-19) in children
368	ChiCTR2000030681	2020/3/9	Liaocheng people's hospital	An anaesthesia procedure and extubation strategy for reducing patient agitation and cough after extubation that can be used to prevent the spread of novel coronavirus pneumonia (COVID-19) and other infectious viruses in the operating Room
369	ChiCTR2000030683	2020/3/9	The Sixth Affiliated Hospital of Sun Yat-sen University	The prediction value of prognosis of novel coronavirus pneumonia (COVID-19) in elderly patients by modified early warning score (MEWS): a medical records based retrospective observational study

370	ChiCTR2000030686	2020/3/9	Peking University Third Hospital	The effects of prevention and control measures on treatment and psychological status of cancer patients during the novel coronavirus pneumonia (COVID-19) outbreak
371	ChiCTR2000030687	2020/3/9	Department of Nephrology, Wuhan Children's Hospital	Novel coronavirus pneumonia (COVID-19) associated kidney injury in children
372	ChiCTR2000030690	2020/3/10	The Third Affiliated Hospital of the Second Military Medical University	Study for immune cell subsets in convalescent patients with novel coronavirus pneumonia (COVID-19)
373	ChiCTR2000030697	2020/3/10	Ningbo First Hospital, Ningbo Hospital of Zhejiang University	A multi-center, open-label observation study for psychological status and intervention efficacy of doctors, nurses, patients and their families in novel coronavirus pneumonia (COVID-19) designated hospitals
374	ChiCTR2000030700	2020/3/10	Shenzhen Hepalink Pharmaceutical Group Co., Ltd/Union Hospital affiliated to Tongji Medical College of Huazhong University of Science and Technology	Study for the Efficacy and safety of Prolongin (Enoxaparin Sodium Injection) in treatment of novel coronavirus pneumonia (COVID-19) adult common patients
375	ChiCTR2000030701	2020/3/10	Shenzhen Hepalink Pharmaceutical Group Co., Ltd/Union Hospital affiliated to Tongji Medical College of Huazhong University of Science and Technology	A randomized, parallel controlled open-label trial for the efficacy and safety of Prolongin (Enoxaparin Sodium Injection) in the treatment of adult patients with novel coronavirus pneumonia (COVID-19)
376	ChiCTR2000030702	2020/3/10	China-Japan Friendship Hospital	Using plasma of the convalescent in the treatment of novel coronavirus pneumonia (COVID-19) common patient: a prospective clinical trial
377	ChiCTR2000030703	2020/3/10	Xiangya Hospital of Central South University	A randomized, blinded, controlled, multicenter clinical trial to evaluate the efficacy and safety of Ixekizumab combined with conventional antiviral drugs in patients with novel coronavirus pneumonia (COVID-19)
378	ChiCTR2000030707	2020/3/11	Ganzi Hospital of West China Hospital, Sichuan University	Retrospective study on novel coronavirus pneumonia (COVID-19) in Tibetan Plateau

379	ChiCTR2000030708	2020/3/11	West China Hospital, Sichuan University	Cross sectional study of dialysis treatment and mental status under the outbreak of novel coronavirus pneumonia (COVID-2019) in China
380	ChiCTR2000030716	2020/3/11	Huazhong University of Science and Technology	Shedding of SARS-CoV-2 in human semen and evaluation of reproductive health of novel coronavirus pneumonia (COVID-19) male patients
381	ChiCTR2000030717	2020/3/11	Department of Emergency, Tongji Hospital, Tongji Medical College, Huazhong University of Science and Technology	A medical records based study for risk assessment and treatment timing of invasive fungal infection in novel coronavirus pneumonia (COVID-19) critical patients
382	ChiCTR2000030718	2020/3/11	Zhongnan Hospital of Wuhan University	Randomized controlled trial for Chloroquine Phosphate in the Treatment of novel coronavirus pneumonia (COVID-19)
383	ChiCTR2000030720	2020/3/11	Hubei Provincial Hospital of TCM	Prognosis Investigation and Intervention Study on Patients with novel coronavirus pneumonia (COVID-19) in recovery period Based on Community Health Management
384	ChiCTR2000030721	2020/3/12	Affiliated Hospital of Guangdong Medical University	A comparative study for the sensitivity of induced sputum and throat swabs for the detection of SARS-CoV-2 by real-time PCR in patients with novel coronavirus pneumonia (COVID-19)
385	ChiCTR2000030722	2020/3/12	Shanghai First People's Hospital	Auscultatory characteristics of novel coronavirus pneumonia (COVID-19)
386	ChiCTR2000030739	2020/3/13	Shandong Provincial Chest hospital	Exploration of the Clinical Characteristics of Patients with Novel Coronavirus Pneumonia (COVID-19) and Its Differences from Patients with Severe Influenza A and MERS
387	ChiCTR2000030740	2020/3/13	Shandong Provincial Chest hospital	Analysis of the incidence and risk factors of ARDS in patients with Novel Coronavirus Pneumonia (COVID-19).
388	ChiCTR2000030741	2020/3/13	Shandong Provincial Chest hospital	Observational Study for Prone Position Ventilation and Conventional Respiratory Support in ARDS Patients with Novel Coronavirus Pneumonia (COVID-19)

389	ChiCTR2000030742	2020/3/13	Department of Geriatrics, Tongji Hospital, Tongji Medical College, Huazhong University of Science and Technology	Characteristics, prognosis, and treatments effectiveness of critically ill patients with Novel Coronavirus Pneumonia (COVID-19)
390	ChiCTR2000030744	2020/3/13	Shandong Provincial Chest hospital	Clinical Application of ECMO(or Ultra-Protective Lung Mechanical Ventilation) in the Treatment of Patients with ARDS due to novel Coronavirus Pneumonia (COVID-19)
391	ChiCTR2000030750	2020/3/13	Shenzhen Third People's Hospital/Shenzhen Ruipuxun Academy for Stem Cell & Regenerative Medicine	A clinical study for effectiveness and safety evaluation for recombinant chimeric COVID-19 epitope DC vaccine in the treatment of novel coronavirus pneumonia
392	ChiCTR2000030752	2020/3/13	Shandong Provincial Chest hospital	A medical records based analysis for risk factors for death in patients with Novel Coronavirus Pneumonia (COVID-19)
393	ChiCTR2000030753	2020/3/13	Shandong Provincial Chest hospital	A medical records based analysis of the Incidence and Risk Factors of Ventilator-associated Pneumonia in ARDS Patients with Novel Coronavirus Pneumonia (COVID-19)
394	ChiCTR2000030754	2020/3/13	The Third Affiliated Hospital of Sun Yat-sen University	Medical records based study for the accuracy of SARS-CoV-2 IgM antibody screening for diagnosis of novel coronavirus pneumonia (COVID-19)
395	ChiCTR2000030755	2020/3/13	Department of Geriatrics, Tongji Hospital, Tongji Medical College, Huazhong University of Science and Technology	A medical records based study for characteristics, prognosis of ederly patients with Novel Coronavirus Pneumonia (COVID-19) in Wuhan area
396	ChiCTR2000030756	2020/3/13	Shenzhen Hospital of Southern Medical University	Detection of SARS-CoV-2 in EPS / semen of patients with novel coronavirus pneumonia (COVID-19)
397	ChiCTR2000030757	2020/3/13	Clinical Research Institute, Shenzhen-Peking University-The Hong Kong University of Science & Technology Medical Center	Impact of Novel Coronavirus Pneumonia (COVID-19) Epidemic Period on the Management of investigator-initiated clinical trial and the resilience of medical service providers
398	ChiCTR2000030758	2020/3/13	Eergency / Intensive Care Department of Tongji Hospital, Tongji Medical College,Huazhong University of Science	A medical records based study for sedation and Analgesia Usage in critically ill patients with novel coronavirus pneumonia (COVID-19)

			and Technology	
399	ChiCTR2000030760	2020/3/13	Zhejiang Hospital	A medical records based study for clinical characteristics of 2019 novel coronavirus pneumonia (COVID-19) in Zhejiang province, China
400	ChiCTR2000030761	2020/3/13	Renji Hospital Affiliated to Shanghai Jiaotong University School of Medicine	Continuous renal replacement therapy (CRRT) alleviating inflammatory response in severe patients with novel coronavirus pneumonia (COVID-19) associated with renal injury: A Prospective study
401	ChiCTR2000030763	2020/3/13	Shandong Provincial Chest hospital	A Medical Records Based analysis for Risk Factors for Outcomes After Respiratory Support in Patients with ARDS Due to Novel Coronavirus Pneumonia (COVID-19)
402	ChiCTR2000030764	2020/3/13	Xiyuan Hospital of China Academy of Chinese Medical Sciences	Research for the influence of epidemic of novel coronavirus pneumonia (COVID-19) on sleep, psychological and chronic diseases among different populations
403	ChiCTR2000030768	2020/3/14	Zhongnan Hospital of Wuhan University	Study for the Psychological Status of Medical Staff of Otolaryngology Head and Neck Surgery in Hubei Province under the Epidemic of novel coronavirus pneumonia (COVID-19)
404	ChiCTR2000030771	2020/3/14	The 2nd Xiangya Hospital of CSU	Screening and identification of peripheral blood biomarkers in patients with novel coronavirus pneumonia (COVID-19) based on multiomics studies
405	ChiCTR2000030772	2020/3/14	Huashan Hospital Fudan University	Application of blood purification in the treatment of novel coronavirus pneumonia (COVID-19)
406	ChiCTR2000030778	2020/3/14	Ningbo First Hospital	A medical records based study for epidemic and clinical features of novel coronavirus pneumonia (COVID-19) in Ningbo First Hospital
407	ChiCTR2000030779	2020/3/14	Shanghai Changzheng Hospital	A clinical trial for Ulinastatin Injection in the treatment of patients with

				severe novel coronavirus pneumonia (COVID-19)
408	ChiCTR2000030782	2020/3/14	Cangzhou People's Hospital	The value of CD4 / CD8 cells, CRP / ALB and APCHEII in novel coronavirus pneumonia (COVID-19)
409	ChiCTR2000030784	2020/3/14	The People's Hospital of Guangxi Zhuang Autonomous Region	A study for clinical characteristics of novel coronavirus pneumonia (COVID-19) patients follow-up in Guangxi
410	ChiCTR2000030795	2020/3/15	Tongji Hospital, Tongji Medical College, Huazhong University of Science and Technology	A multicenter retrospective study of rheumatic patients with novel coronavirus pneumonia (COVID-19)
411	ChiCTR2000030796	2020/3/15	The Second Hospital of Hebei Medical University	Clinical characteristics and treatment of novel coronavirus pneumonia (COVID-19)
412	ChiCTR2000030797	2020/3/15	Shanghai Fifth People's Hospital, Fudan University	Clinical study for hemodynamics and cardiac arrhythmia of novel coronavirus pneumonia (COVID-19) patients
413	ChiCTR2000030798	2020/3/15	Wuhan 3rd Hospital	A medical records based study for clinical characteristics of novel coronavirus pneumonia (COVID-19)
414	ChiCTR2000030799	2020/3/15	Tongji Hospital, Tongji Medical College, Huazhong University of Science and Technology	Establishment and validation of Premonitory model of deterioration of the 2019 novel corona virus pneumonia (COVID-19)
415	ChiCTR2000030801	2020/3/15	Wuhan 3rd Hospital	Analysis of risk factors affecting prognosis of elderly patients infected with novel coronavirus pneumonia (COVID-19): a single-center retrospective observational study
416	ChiCTR2000030802	2020/3/15	Tongji Hospital, Tongji Medical College of HUST	A retrospective study of clinical drug therapy in patients with novel coronavirus pneumonia (COVID-19)
417	ChiCTR2000030803	2020/3/15	Union Hospital affiliated to Tongji Medical College of Huazhong University of Science and Technology	Collection and analysis of clinical data in severe and critically ill patients with novel coronavirus pneumonia (COVID-19)
418	ChiCTR2000030805	2020/3/15	Tongji Hospital, Tongji Medical College, Huazhong University of Science & Technology	Quantitative CT characteristic estimate the severity of novel coronavirus pneumonia (COVID-19)

419	ChiCTR2000030807	2020/3/15	Tongji Hospital, Tongji Medical College, Huazhong University of Science and Technology(HUST)	Clinical characteristics and prognosis of cancer patients with novel coronavirus pneumonia (COVID-19)
420	ChiCTR2000030809	2020/3/15	Tongji Hospital, Tongji Medical College, Huazhong University of Science and Technology(HUST)	A medical records based study for clinical outcomes and follow-up of novel coronavirus pneumonia (COVID-19) patients
421	ChiCTR2000030812	2020/3/15	Shanghai Ninth People's Hospital	Study for the virus molecular evolution which driven the immune-pathological responses and the protection mechanisms of novel coronavirus pneumonia (COVID-19) patients
422	ChiCTR2000030814	2020/3/15	Tongji hospital, Tongji medical College, Huazhong University of Science and Technology	A medical records based analysis of clinical evidence of human-to-human transmission of novel coronavirus pneumonia (COVID-19) by conjunctival route
423	ChiCTR2000030816	2020/3/15	Tongji hospital, Tongji medical College, Huazhong University of Science and Technology	Nutritional risk assessment and outcome prediction of critically ill novel coronavirus pneumonia (COVID-19) patients
424	ChiCTR2000030817	2020/3/15	Union Hospital affiliated to Tongji Medical College of Huazhong University of Science and Technology	Multicenter clinical study of evaluation of multi-organ function in patients with novel coronavirus pneumonia (COVID-19) by ultrasound
425	ChiCTR2000030818	2020/3/15	The First Affiliated Hospital, He'nan Traditional Chinese Medicine University	A medical records based study for the value of Lymphocyte subsets in the diagnose and treatment of novel coronavirus pneumonia (COVID-19)
426	ChiCTR2000030819	2020/3/15	Wuhan 3rd Hospital	Retrospective analysis of digestive system symptoms in 600 cases of novel coronavirus pneumonia (COVID-19) in Guanggu district, Wuhan
427	ChiCTR2000030830	2020/3/15	Hwa Mei Hospital, University of Chinese Academy of Sciences	Development and application of novel coronavirus pneumonia (COVID-19) intelligent image classification system based on deep learning
428	ChiCTR2000030831	2020/3/15	Tongji Hospital, Tongji Medical College, Huazhong University of Science and Technology	The analysis of related factors on improving oxygenation status by endotracheal intubation ventilation in severe patients suffered from novel coronavirus pneumonia (COVID-19): a single center and descriptive study in Wuhan

429	ChiCTR2000030832	2020/3/15	Tongji Hospital, Tongji Medical College, Huazhong University of Science and Technology(HUST)	Study for the pathogenesis and effective intervention of mood disorders caused by the novel coronavirus pneumonia (COVID-19)
430	ChiCTR2000030834	2020/3/15	Tongji Hospital, Tongji Medical College, Huazhong University of Science and Technology(HUST)	Epidemiological Characteristics and Antibody Levels of novel coronavirus pneumonia (COVID-19) of Pediatric Medical Staff working in Quarantine Area
431	ChiCTR2000030835	2020/3/15	the First Affiliated Hospital of Xinxiang Medical University	Clinical study for the efficacy of Mesenchymal stem cells (MSC) in the treatment of severe novel coronavirus pneumonia (COVID-19)
432	ChiCTR2000030838	2020/3/15	Wuhan University Central South Hospital	Development of warning system with clinical differential diagnosis and prediction for severe type of novel coronavirus pneumonia (COVID-19) patients based on artificial intelligence and CT images
433	ChiCTR2000030839	2020/3/15	Tongren Hospital, Shanghai Jiao Tong University School of Medicine	Preliminary screening of novel coronavirus pneumonia (COVID-19) by special laboratory examination and CT imaging before surgery
434	ChiCTR2000030841	2020/3/15	Union Hospital affiliated to Tongji Medical College of Huazhong University of Science and Technology	Exploratory study for Immunoglobulin From Cured COVID-19 Patients in the Treatment of Acute Severe novel coronavirus pneuvirus (COVID-19)
435	ChiCTR2000030849	2020/3/15	Zhengzhou People's Hospital	Investigation on psychological status of novel coronavirus pneumonia (COVID-19) rehabilitation patients in Zhengzhou City and research on coping strategies
436	ChiCTR2000030850	2020/3/15	the First Affiliated Hospital, Guangzhou University of Chinese Medicine	Study for the physical and mental health status of medical workers under the novel coronavirus pneumonia (COVID-19) epidemic
437	ChiCTR2000030852	2020/3/16	Beijing Anzhen Hospital, Capital Medical University	Factors associated with death in patients with novel coronavirus pneumonia (COVID-19)
438	ChiCTR2000030853	2020/3/16	The Third Affiliated Hospital of Zunyi Medical University	Evaluation of the protective effect of dexmedetomidine on patients with severe novel coronavirus pneumonia (COVID-19)

439	ChiCTR2000030854	2020/3/16	The First Affiliated Hospital, College of Medicine,Zhejiang University	A clinical multicenter study for the occurrence, development and prognosis of novel coronavirus pneumonia (COVID-19)
440	ChiCTR2000030855	2020/3/16	The Third Affiliated Hospital of Zunyi Medical University	Study for the effect of external diaphragmatic pacing assisted invasive ventilation and weaning in patients with severe novel coronavirus pneumonia (COVID-19)
441	ChiCTR2000030856	2020/3/16	Tongji Hospital of Tongji Medical College of Huazhong University of Science and Technology	An artificial intelligence assistant system for suspected novel coronavirus pneumonia (COVID-19) based on chest CT
442	ChiCTR2000030857	2020/3/16	Tongji Hospital of Tongji Medical College of Huazhong University of Science and Technology	Clinical study for bronchoscopic alveolar lavage in the treatment of critically trachea intubation patients with new coronavirus pneumonia (COVID-19)
443	ChiCTR2000030858	2020/3/16	Hubei Cancer Hospital	Clinical characteristics and outcomes of 483 mild patients with novel coronavirus pneumonia (COVID-19) in Wuhan, China during the outbreak: A single-center, retrospective study from the mobile cabin hospital
444	ChiCTR2000030859	2020/3/16	Wuhan 3rd Hospital	A medical based analysis for influencing factors of death of novel coronavirus pneumonia (COVID-19) patients in Wuhan third hospital
445	ChiCTR2000030860	2020/3/16	Tongji Hospital, Tongji Medical College, Huazhong University of Science and Technology	A medical records based study for investigation of dynamic profile of RT-PCR test for SARS-CoV-2 nucleic acid of novel coronavirus pneumonia (COVID-19) patients
446	ChiCTR2000030861	2020/3/16	The First Affiliated Hospital of the Guangzhou Medical University	Development and application of a new intelligent robot for novel coronavirus (2019-nCOV) oropharygeal sampling
447	ChiCTR2000030862	2020/3/16	Tongji Hospital of Tongji Medical College of Huazhong University of Science and Technology	Correlation analysis of blood eosinophil cell levels and clinical type category of novel coronavirus pneumonia (COVID-19): a medical records based retrospective study
448	ChiCTR2000030863	2020/3/16	Jinling Hospital, Medical School of Nanjing University	Clinical and CT imaging Characteristics of novel coronavirus pneumonia (COVID-19): An Multicenter Cohort Study

449	ChiCTR2000030865	2020/3/16	Tongji Hospital, Huazhong University of Science and Technology	Establishment of an early warning model for maternal and child vertical transmission of COVID-19 infection
450	ChiCTR2000030866	2020/3/16	The First Hospital of Changsha	Open-label, observational study of human umbilical cord derived mesenchymal stem cells in the treatment of severe and critical patients with novel coronavirus pneumonia (COVID-19)
451	ChiCTR2000030892	2020/3/16	State Key Laboratory of Respiratory Disease, National Clinical Center for Respiratory Disease, Guangzhou Institute of Respiratory Health, The First Affiliated Hospital of Guangzhou Medical University	Efficacy and Safety of Pirfenidone in the Treatment of Severe Post-Novel Coronavirus Pneumonia (COVID-19) Fibrosis: a prospective exploratory experimental medical study
452	ChiCTR2000030893	2020/3/16	Zhengzhou People's Hospital	Study for effects of crisis intervention based on positive psychology for medical staffs working in the novel coronavirus pneumonia (COVID-19) field
453	ChiCTR2000030894	2020/3/16	Peking University First Hospital	Favipiravir Combined With Tocilizumab in the Treatment of novel coronavirus pneumonia (COVID-19) - A Multicenter, Randomized, Controlled Trial
454	ChiCTR2000030895	2020/3/16	Tongji Hospital, Tongji Medical College, Huazhong University of Science and Technology	Retrospective and Prospective Study for Nosocomial infection in Stomatology Department under the Background of novel coronavirus pneumonia (COVID-19) epidemic period
455	ChiCTR2000030897	2020/3/16	HwaMei Hospital, University of Chinese Academy of Sciences	Evaluation of the effect of taking Newgen beta-gluten probiotic composite powder to nutrition intervention of patients with novel coronavirus pneumonia (COVID-19)
456	ChiCTR2000030902	2020/3/16	Jingzhou Mental Health Center	Analysis on the mental health status of residents in Jingzhou during the outbreak of the novel coronavirus pneumonia (COVID-19) and corresponding influencing factors
457	ChiCTR2000030906	2020/3/16	Insitute of Biotechbology, Military Medical Science, PLA of China	A phase I clinical trial for recombinant novel coronavirus (2019-CoV) vaccine (adenoviral vector)

458	ChiCTR2000030919	2020/3/17	Intensive Care Unit, XiangYa Hospital, Central South University	An observational study for cardiac and pulmonary ultrasound and evaluation of treatment of severe patients with novel coronavirus pneumonia (COVID-19)
459	ChiCTR2000030922	2020/3/17	Department of Infectious Diseases, Foshan First People's Hospital	Prospective, open-label, controlled, multicenter cohort study of long-acting interferon plus ribavirin in patients with novel coronavirus pneumonia (COVID-19)
460	ChiCTR2000030923	2020/3/17	Affiliated Hospital of Shaanxi University of Traditional Chinese Medicine	The treatment and diagnosis plan of integrated traditional Chinese and Western medicine for novel coronavirus pneumonia (COVID-19)
461	ChiCTR2000030929	2020/3/17	Renmin Hospital of Wuhan University	A randomized, double-blind, parallel-controlled trial to evaluate the efficacy and safety of anti-SARS-CoV-2 virus inactivated plasma in the treatment of severe novel coronavirus pneumonia (COVID-19)
462	ChiCTR2000030931	2020/3/18	HuiZhou Municipal Central Hospital	A Medical Records Based Study for Clinical Characteristic and Outcomes of Hospitalized Patients With Novel Coronavirus Pneumonia (COVID-19)
463	ChiCTR2000030932	2020/3/18	Department of Infectious Diseases, Anqing Municipal Hospital	Correlation between virological negative conversion and clinical factors and prognosis in patients with novel coronavirus pneumonia (COVID-19)
464	ChiCTR2000030934	2020/3/18	The Fifth Affiliated Hospital of Sun Yat-sen University	A Platform for Rapid Immuno-detection and Emergency Vaccine Development of Novel Coronavirus (2019-COV)
465	ChiCTR2000030939	2020/3/18	PLA General Hospital	Preliminary evaluation of the safety and efficacy of oral LL-37 antiviral peptide (CAS001) in the treatment of novel coronavirus pneumonia (COVID-19)
466	ChiCTR2000030941	2020/3/18	Hu'nan Provincial People's Hospital, Hu'nan Normal University	A medical records based study for clinical features and prognosis of severe patients with novel coronavirus pneumonia (COVID-19) in Huanggan, Hubei in Huanggang.

467	ChiCTR2000030942	2020/3/19	Shanghai Kongjiang Hospital	Combined diagnostic value of novel coronavirus (2019-CoV) infection detected by NLR and CRP
468	ChiCTR2000030943	2020/3/19	Shandong Provincial Hospital	Venous Thrombosis Risk of novel coronavirus pneumonia (COVID-19) Patients: A Prospective Study
469	ChiCTR2000030944	2020/3/19	The Second Affiliated Hospital of Nanchang University	Clinical study of human NK cells and MSCs transplantation for severe novel coronavirus pneumonia (COVID-19)
470	ChiCTR2000030946	2020/3/19	Effects of different VTE prevention methods on the prognosis of hospitalized patients with novel coronavirus pneumonia (COVID-19)	Effects of different VTE prevention methods on the prognosis of hospitalized patients with novel coronavirus pneumonia (COVID-19)
471	ChiCTR2000030947	2020/3/19	Beijing Chaoyang Hospital	Clinical application of extracorporeal membrane oxygenation in the treatment of severe respiratory failure patients with novel coronavirus pneumonia (COVID-19)
472	ChiCTR2000030950	2020/3/19	West China Hospital, Sichuan University	Study for novel coronavirus pneumonia (COVID-19) patients etiology and immune response and guidance for vaccine design
473	ChiCTR2000030951	2020/3/19	Tongji Hospital, Tongji Medical College, Huazhong University of Science and Technology	Developing and evaluating of artificial intelligence triage system for suspected novel coronavirus pneumonia (COVID-19): a retrospective study
474	ChiCTR2000030952	2020/3/19	The Fifth Hospital Affiliated to Sun Yat-sen University	Myocardial injury and arrythmias in the novel coronavirus pneumonia (COVID-19) patients
475	ChiCTR2000030961	2020/3/20	Department of Respiratory Medicine, Changzheng Hospital, Naval Military Medical University	A Medical Records Based Retrospective Study for Clinical Characteristics, Treatments and Prognosis of Patients with Novel Coronavirus Pneumonia (COVID-19) in WuHan
476	ChiCTR2000030985	2020/3/20	Renmin Hospital of Wuhan University	Psychological Responses of Medical Staff during the Metaphase of novel coronavirus pneumonia (COVID-19) Outbreak in Hubei, China

477	ChiCTR2000030986	2020/3/20	Shenzhen Third People's Hospital	Correlation of T lymphocytes level and clinical severity in novel coronavirus pneumonia (COVID-19) patients: a medical records based retrospective study
478	ChiCTR2000030987	2020/3/20	Beijing Chao-yang Hospital, Capital Medical University	A Randomized Controlled Trial for Favipiravir Tablets Combine With Chloroquine Phosphate in the Treatment of Novel Coronavirus Pneumonia (COVID-19)
479	ChiCTR2000030989	2020/3/20	Mental Health Service Center, Beijing Normal University	Internet based Solution Focused Brief Therapy treating adolescent anxiety under the novel coronavirus pneumonia (COVID-19) outbreak: A randomized controlled trial
480	ChiCTR2000030992	2020/3/20	Department of Respiratory Medicine, Changzheng Hospital, Naval Military Medical University	A medical records based study for the diagnosis and prognosis prediction AI model of novel coronavirus pneumonia (COVID-19)
481	ChiCTR2000030993	2020/3/20	Wuhan Third Hospital	A medical records based study for the value of Upper Respiratory Tract Virus Detection in the assessment of rehabilitation of novel coronavirus pneumonia (COVID-19) patient
482	ChiCTR2000031014	2020/3/21	HwaMei Hospital, University of Chinese Academy of Sciences	Study for SARS-COV-2 RNA Level in Blood and Excrement of Novel Coronavirus Pneumonia (COVID-19) Convalescent Patients
483	ChiCTR2000031088	2020/3/22	Xiangyang 1st People's Hospital	A prognosis study of novel coronavirus pneumonia (COVID-19)
484	ChiCTR2000031090	2020/3/22	Ningbo Kangning Hospital	Psychological Support for Diagnosed Patients with novel coronavirus pneumonia (COVID-19)
485	ChiCTR2000031104	2020/3/22	The Fifth Affiliated Hospital of Sun Yat-Sen University	Study for metagenomics of patients with novel coronavirus pneumonia (COVID-19)
486	ChiCTR2000031115	2020/3/22	Zhengzhou People's Hospital	A medical records based study of peripheral blood T lymphocyte subsets in patients with novel coronavirus pneumonia (COVID-19)

487	ChiCTR2000031138	2020/3/22	The Third Xiangya Hospital of Central South University	A randomized controlled trial for the efficacy and safety of pirfenidone capsules in the treatment of severe novel coronavirus pneumonia (COVID-19)
488	ChiCTR2000031139	2020/3/22	Wuhan Jinyintan Hospital (Wuhan Infectious Diseases Hospital)	Safety and Effectiveness of Human embryonic stem cell-derived M cells (CAStem) for Pulmonary Fibrosis Correlated with novel coronavirus pneumonia (COVID-19)
489	ChiCTR2000031140	2020/3/22	Union Hospital West Campus, Tongji Medical College, Huazhong University of Science and Technology	Study for the impact on fetus and neonates of vertical transmission of 2019-nCoV
490	ChiCTR2000031150	2020/3/23	Hwa Mei Hospital, University of Chinese Academy of Sciences	Application of radiology in the prevention and control system of emergency respiratory infectious diseases
491	ChiCTR2000031163	2020/3/23	The People's Hospital of GuangXi Zhuang Autonomous Region	The relationship between Vitamin D andnovel coronavirus pneumonia (COVID-19)
492	ChiCTR2000031174	2020/3/23	Shanghai Public Health Clinical Center	Effectiveness and safety of hydroxychloroquine sulfate in the preventive treatment of novel coronavirus pneumonia (COVID-19)
493	ChiCTR2000031176	2020/3/23	People's hospital of Zhengzhou	Study on home pharmaceutical care for chronic patients over the novel coronavirus pneumonia (COVID-19) epidemic
494	ChiCTR2000031187	2020/3/23	Wuhan Third Hospital & Tongren Hospital of Wuhan University	A medical records based retrospective study for novel coronavirus pneumonia (COVID-19)
495	ChiCTR2000031196	2020/3/23	Diagnosis, treatment and Research Center for infectious diseases, the fifth medical center of the PLA	Efficacy and optimization of antiviral therapy for novel coronavirus pneumonia (COVID-19) patients
496	ChiCTR2000031204	2020/3/24	Beijing Institute of Pharmacology and Toxicology/Beijing You'an Hospital, Capital Medical University	A multicenter, single-blind, randomized controlled clinical trial for chloroquine phosphate in the treatment of novel coronavirus pneumonia (COVID-19)
497	ChiCTR2000031214	2020/3/24	The First Rehabilitation Hospital of Shanghai	Clinical study on the correlation between novel coronavirus pneumonia (COVID-19) clinical rehabilitation assessment and prognosis

498	ChiCTR2000031227	2020/3/24	Renmin Hospital of Wuhan University	Clinical features and prognosis of invasive echanicalventilation patients with novel coronavirus pneumonia (COVID-19) in Wuhan, China: a single-centered, retrospective, observational study
499	ChiCTR2000031244	2020/3/25	Tongji Hospital, Tongji Medical College, Huazhong University of Science and Technology	Shedding virus and functional prognosis in patients with novel coronavirus pneumonia (COVID-19): a cohort study
500	ChiCTR2000031245	2020/3/25	Renmin Hospital of Wuhan University	Epidemiologic and Clinical Characteristics of novel coronavirus pneumonia (COVID-19) in surgical patients: a medical records based retrospective study
501	ChiCTR2000031246	2020/3/25	School of Public Health and Management, Chongqing Medical University	Epidemiological survey of close contacts of novel coronavirus pneumonia (COVID-19)
502	ChiCTR2000031247	2020/3/25	West China Hospital, Sichuan University	Study on the mental state of medical staff during the epidemic period of novel coronavirus pneumonia (COVID-19)
503	ChiCTR2000031252	2020/3/25	Department of Public Health and Management, Chongqing Medical University	Outcomes and infectivity of patients with asymptomatic novel coronavirus (COVID-19) infection
504	ChiCTR2000031271	2020/3/26	Department of Pediatrics, The Third Affiliated Hospital of Sun Yat-sen University	Metabolic abnormalities and rearrangement reversal treatment of patient with novel coronavirus pneumonia (COVID-19)
505	ChiCTR2000031272	2020/3/26	China European International Business School (CEIBS)	The effectiveness of mindfulness intervention in improving people's psychological and physical well-being during novel coronavirus pneumonia (COVID-19) outbreak
506	ChiCTR2000031293	2020/3/27	Tongji Hospital of Tongji Medical College, Huazhong University of Science and Technology	Epidemiological study of novel coronavirus infection (COVID-19) in children at medium/low risk
507	ChiCTR2000031296	2020/3/27	Yichang Central People's Hospital	A medical records based study for clinical features of novel coronavirus pneumonia (COVID-19) patients and risk factors of death

508	ChiCTR2000031301	2020/3/27	China-Japan Union hospital of Jilin University	A retrospective study of clinical characteristics and prognosis of novel coronavirus pneumonia (COVID-19) patients with myocardial injury
509	ChiCTR2000031319	2020/3/27	Center for Regenerative Medicine, Renmin Hospital of Wuhan University	Safety and Efficacy Study of Allogeneic Human Dental Pulp Mesenchymal Stem Cells to Treat Severe novel coronavirus pneumonia (COVID-19) patients
510	ChiCTR2000031324	2020/3/27	Nanjing University Medical College Affiliated Drum Tower Hospital	The investigation on medical adhesive-related skin injury caused by protective dressings of medical staffs during the period of novel coronavirus pneumonia (COVID-19)
511	ChiCTR2000031327	2020/3/27	Tongji Hosptial, Tongji Medical College, Huazhong University of Science and Technology	Clinical characteristics and death risk factors in severe novel coronavirus pneumonia (COVID-19) patients: a single-center respective analysis based on medical records
512	ChiCTR2000031328	2020/3/27	First Teaching Hospital of Tianjin University of Traditional Chinese Medicine	The protective effect of sleep psychology and music therapy of novel coronavirus pneumonia (COVID-19) mild and moderate type patients
513	ChiCTR2000031329	2020/3/27	Guangdong Second Provincial General Hospital	Influence of novel coronavirus pneumonia (COVID-19) on disease activity, medical and mental condition of patients with rheumatic diseases
514	ChiCTR2000031330	2020/3/27	Shandong Eye Institute	Analysis for prevention and control effects of orthokeratology lens on myopia adolescents during the novel coronavirus pneumonia (COVID-19) pandemic situation
515	ChiCTR2000031336	2020/3/28	Union Hospital, Tongji Medical College, Huazhong University of Science and Technology	AI based prognostic evaluation of novel coronavirus pneumonia (COVID-19)
516	ChiCTR2000031337	2020/3/28	Shandong Eye Institute	The relationship between habits of using eyes, degree of visual fatigue and myopia progression in students during the novel coronavirus pneumonia (COVID-19) pandemic
517	ChiCTR2000031356	2020/3/28	Union Hospital, Tongji Medical College, Huazhong	Study for the changes of the brain structure and function in healed patients

			University of Science and Technology	of novel coronavirus pneumonia (COVID-19)
518	ChiCTR2000031361	2020/3/28	Maternal and Child Health Hospital of Hubei Province	Retrospective analysis of anesthesia management of emergency cesarean section in non-pneumonia hospital of Wuhan during pandemic of novel coronavirus pneumonia (COVID-19)
519	ChiCTR2000031365	2020/3/29	Taizhou Hospital of Zhejiang Province	To explore the pathogenesis and course prediction of novel coronavirus pneumonia (COVID-19) severe patients
520	ChiCTR2000031366	2020/3/29	Department of Pulmonary & Critical Care Medicine, Chinese People's Liberation Army (PLA) General Hospital	Causes of fever in outpatient outside Wuhan during novel coronavirus pneumonia (COVID-19) pandemic: a medical records based retrospective analysis
521	ChiCTR2000031376	2020/3/29	Peking University Third Hospital	A medical records based study for safety and effectiveness analysis of data from novel coronavirus pneumonia (COVID-19) patients with conventional therapy
522	ChiCTR2000031427	2020/3/31	the First People's Hospital of Jiangxia District	Novel coronavirus infection (COVID-19) IgM detection kit (magnetic particle chemiluminescence) clinical trial
523	ChiCTR2000031428	2020/3/31	Wuhan Asia General Hospital	Clinical application value of multiple tests for novel coronavirus pneumonia (COVID-19)
524	ChiCTR2000031429	2020/3/31	Tongji Hospital, Tongji Medical College, Huazhong University of Science and Technology	The Effects of novel coronavirus pneumonia (COVID-19) Risk Factors on Dynamic Changes of Immunological, biochemical, and Metabolic Parameters: a Retrospective Observational Study
525	ChiCTR2000031430	2020/3/31	The Fifth Medical Center of PLA General Hospital	Clinical study of human umbilical cord mesenchymal stem cells in the treatment of novel coronavirus pneumonia (COVID-19) induced pulmonary fibrosis
526	ChiCTR2000031432	2020/4/1	Nanjing University	A single center clinical study of evaluating the injection of microRNA2911 plasmid in healthy adults

527	ChiCTR2000031438	2020/4/1	Affiliated Hospital of Southwest Medical University	Analysis of changes in high risk factors of cervical spondylosis during novel coronavirus pneumonia (COVID-19) pandemic
528	ChiCTR2000031439	2020/4/1	Tongji Hospital, Tongji Medical College, Huazhong University of Science and Technology	A medical records based study for novel coronavirus pneumonia (COVID-19) patients undergoing endotracheal intubation
529	ChiCTR2000031453	2020/4/1	Tongji Hospital, Tongji Medical College, Huazhong University of Science and Technology	Efficacy and safety of Nintedanib esilate soft capsules in the treatment of pulmonary fibrosis in healed moderate to severe patients of novel coronavirus pneumonia (COVID-19): a single-center, randomized, placebo-controlled trial
530	ChiCTR2000031454	2020/4/1	The Fifth Affiliated Hospital of Sun Yat-Sen University	Clinical study for prevention and treatment of digestive tract lesions caused by novel coronavirus pneumonia (COVID-19)
531	ChiCTR2000031465	2020/4/2	the First People's Hospital of Jiangxia District	Novel coronavirus (2019-nCOV) IgG detection kit (magnetic particle chemiluminescence) clinical trial
532	ChiCTR2000031494	2020/4/2	Huangshi Hospital of Traditional Chinese Medicine	Clinical study for stem cells in the treatment of severe novel coronavirus pneumonia (COVID-19)
533	ChiCTR2000031499	2020/4/2	Shanghai Oriental Hospital	Sleep quality of patients with novel coronavirus pneumonia (COVID-19) treated in mobile cabin hospital: a retrospective study
534	ChiCTR2000031500	2020/4/2	Peking University First Hospital	Impact of measuring distance and cold outdoor environment on the screening of novel coronavirus pneumonia (COVID-19)
535	ChiCTR2000031501	2020/4/2	Eastern theater General Hospital	The efficacy of convalescent plasma in patients with critical novel coronavirus pneumonia (COVID-19): a pragmatic, prospective cohort study
536	ChiCTR2000031503	2020/4/3	Shanghai Sixth People's Hospital	Application of non-invasive multichannel cardiopulmonary monitoring cloud management platform in patients with Novel Coronavirus Pneumonia (COVID-19)

537	ChiCTR2000031504	2020/4/3	Tongji Hospital, Tongji Medical College, Huazhong University of Science and Technology	A multi-center telephone follow-up study of disease management and infection in rheumatic patients during novel coronavirus pneumonia (COVID-19) pandemic in Hubei province
538	ChiCTR2000031533	2020/4/3	Peking Union Hospital	Survey of psychological status and related factors of emergency medical staff in the country during the novel coronavirus pneumonia (COVID-19) pandemic
539	ChiCTR2000031538	2020/4/3	Ninth People's Hospital, Shanghai Jiao Tong University School of Medicine	Assessment of mental health status of first-line dental emergency medical personnel during novel coronavirus pneumonia (COVID-19) pandemic
540	ChiCTR2000031539	2020/4/3	Wuhan Women and Children's Health Care Center	A Medical Records Based Study for the Correlation between Angiotensin II Type 1 Receptor Blockers (ARBs) and the Progression and Outcome of Novel Coronavirus Pneumonia (COVID-19)
541	ChiCTR2000031540	2020/4/3	Affiliated Hospital of Zunyi Medical University	Study for clinical effect of rehabilitation nursing program for patients with novel coronavirus pneumonia (COVID-19)
542	ChiCTR2000031587	2020/4/4	Tongji Hospital, Tongji Medical College, Huazhong University of Science and Technology	A paired clinical study for novel coronavirus pneumonia (COVID-19) patients with ordinary, severe, critical, and deceased patients in Tongji Hospital
543	ChiCTR2000031589	2020/4/4	Wuhan University Renmin Hospital	Investigation for the sleep quality in non-health care workers during the pandemic of novel coronavirus pneumonia (COVID-19)
544	ChiCTR2000031627	2020/4/4	2nd People's Hospital of Fuyang City	Study for the Mechanism of the Relationship between Infection of novel coronavirus pneumonia (COVID-19) and Host Innate Immune
545	ChiCTR2000031630	2020/4/4	Guangzhou Eighth People's Hospital	Clinical study for Celebrex in the treatment of novel coronavirus pneumonia (COVID-19)

546	ChiCTR20000316392020/4/4	Peking University Sixth Hospital	Effects of a single session internet-delivered cognitive behavioral therapy for Acute Insomnia during the pandemic of novel coronavirus pneumonia (COVID-19)
547	ChiCTR20000316752020/4/5	Women's Hospital, Medical School of Zhejiang University	A medical records based retrospective analysis of maternal and infant outcomes in Cesarean delivery in Hangzhou non pneumonia Hospital during pandemic of Novel coronovirus pneumonia(COVID-19)
548	ChiCTR20000316992020/4/7	Wenzhou Central Hospital	Study for immune antibody and multi-group function of novel coronavirus pneumonia (COVID-19) patients
549	ChiCTR20000317002020/4/7	Chinese PLA General Hospital	Development and clinical application of novel coronavirus pneumonia (COVID-19) antigen reagent
550	ChiCTR20000317052020/4/7	Shanghai Jiao Tong University Affiliated Sixth People's Hospital	Study for early biological warning signals in patients with acute renal injury caused by novel coronavirus pneumonia (COVID-19)
551	ChiCTR20000317072020/4/7	Shanghai Orient Hospital	Study for sleep quality of medical teams members moved to Hubei for the fighting of novel coronavirus pneumonia (COVID-19) pandemic
552	ChiCTR20000317122020/4/7	Chinese University of Hong Kong	Study for investigation of contamination by novel coronavirus pneumonia (COVID-19) in the clinical environment
553	ChiCTR20000317342020/4/8	Huoshenshan Hospital	a randomized, open-label, controlled trial
554	ChiCTR20000317352020/4/8	Huzhou Central Hospital	Clinical study for natural killer (NK) cells from umbilical cord blood in the treatment of novel coronavirus pneumonia (COVID-19)
555	ChiCTR20000317512020/4/8	Wuhan Fourth Hospital	Novel coronavirus pneumonia (COVID-2019) patients' prognosis and their influence on heart and lung function
556	ChiCTR20000317522020/4/9	the First Affiliated Hospital of Nanjing Medical University	A medical records based observational study for myocardial injury and cardiac function in critically ill patients with novel coronavirus pneumonia (COVID-19)

557	ChiCTR2000031770	2020/4/10	Shanghai Jiao Tong University Affiliated Sixth People's Hospital	Experiences in nursing care for patients with suspected novel coronavirus pneumonia (COVID-19) in Shanghai
558	ChiCTR2000031779	2020/4/10	Shandong Provincial Hospital	Effect of novel coronavirus pneumonia (COVID-19) upon male reproductive system
559	ChiCTR2000031781	2020/4/10	Insitute of Biotechnology, Academy of Military Medical Sciences, PLA of China/Jiangsu Provincial Center for Disease Control and Prevention	A randomized, double-blinded, placebo-controlled phase II clinical trial for Recombinant Novel Coronavirus (2019-nCOV) Vaccine (Adenovirus Vector)
560	ChiCTR2000031782	2020/4/10	The Second Xiangya Hospital of Central South University	A questionnaire investigation of hydroxychloroquine for its potential protective effect against novel coronavirus infection (COVID-19)
561	ChiCTR2000031794	2020/4/10	Huanggang Central Hospital	A medical records based retrospective study for analysis clinical characteristics and risk factors of death in patients with novel coronavirus pneumonia (COVID-19)
562	ChiCTR2000031809	2020/4/10	Wuhan Institute of Biological Products co., LTD./He'nan Provincial Center for Disease Control and Prevention	A randomized, double-blind, placebo parallel-controlled phase I/II clinical trial for inactivated COVID-19 vaccine (Vero cells)
563	ChiCTR2000031834	2020/4/11	Chengdu Shangyi Information Technology co. LTD/Jiangsu Provincial Hospital	A clinical study for the effect of remote monitoring exercise rehabilitation on the discharged patients with novel coronavirus pneumonia (COVID-19)
564	ChiCTR2000031836	2020/4/11	The First Affiliated Hospital of Yangtze University	A Medical Records Based study for the Clinical Characteristics Of Hospitalized Novel Coronavirus Pneumonia (COVID-19) Patients With Acute Respiratory Distress Syndrome
565	ChiCTR2000031860	2020/4/12	Beijing Sports University	Study for the exercise rehabilitation therapy for the dysfunction of cured discharged novel coronavirus pneumonia (COVID-19) patients
566	ChiCTR2000031896	2020/4/14	Fujian Provincial Hospital	Study for the influence of novel coronavirus pneumonia (COVID-19) on mental health of Chinese public hospital stuffs

567	ChiCTR2000031928	2020/4/14	The Chinese University of Hong Kong	Delineate the prevalence, risk factors, temporal distribution and epidemiological characteristics of hidden novel coronavirus (2019-nCoV) infection in the community
568	ChiCTR2000031930	2020/4/15	Affiliated Hospital of Zunyi Medical University	Analysis of clinical characteristics and therapeutic effect of 9 cases of novel coronavirus pneumonia (COVID-19)
569	ChiCTR2000031954	2020/4/15	International Peace Maternity and Children Health Hospital, School of Medicine, Shanghai Jiao Tong University	Medical Records Based Study for Maternal and Perinatal Outcomes of Women with novel coronavirus pneumonia (COVID-19)
570	ChiCTR2000032009	2020/4/17	Renji Hospital, Shanghai Jiaotong University School of Medicine	Clinical characteristics and risk factors of novel coronavirus pneumonia (COVID-19) patients with chronic liver disease
571	ChiCTR2000032010	2020/4/17	Union Hospital, Tongji Medical College, Huazhong University of Science and Technology	Novel coronavirus pneumonia (COVID-19) Intelligent Assistant Analysis System:A Multi-center Clinical Research
572	ChiCTR2000032011	2020/4/17	Sixth Medical Center of PLA General Hospital	Effecacy and Safety of Hyperbaric Oxygen Therapy to patients with novel coronavirus pneumonia (COVID-19)
573	ChiCTR2000032013	2020/4/17	The Fourth Medical Center of Chinese PLA General Hospital	The prevention and treatment strategy of the protective respirator related facial pressure injuries among healthcare professionals fighting novel coronavirus pneumonia (COVID-19)
574	ChiCTR2000032095	2020/4/19	The Third Affiliated Hospital of Sun Yat-sen University	Study for Healthy Behavior, Psychological Analysis and Psychological Reconstruction Strategies of Students Population Keeping in Home Under the Pandemic of Novel Coronavirus Pneumonia (COVID-19)
575	ChiCTR2000032135	2020/4/20	Shanghai Public Health Clinical Center	Efficacy and Safety of ulinastatin in the Treatment of novel coronavirus pneumonia (COVID-19)
576	ChiCTR2000032161	2020/4/21	Tongji Hospital, Tongji Medical College, Huazhong University of Science and Technology	A retrospective study for evolution and clinical outcomes study of novel coronavirus pneumonia (COVID-19) patients

577	ChiCTR2000032162	2020/4/21	Children's Hospital of Chongqing Medical University/Seventh Medical Center of PLA General Hospital	A medical records based study for ECMO in the rescue therapy of extremely critical novel coronavirus pneumonia (COVID-19) patients
578	ChiCTR2000032213	2020/4/23	the First Affiliated Hospital of Chongqing Medical University	A medical records based study for the impact of wearing medical masks on oxygen saturation in adult surgical patients after general anaesthesia during novel coronavirus pneumonia (COVID-19) pandemic
579	ChiCTR2000032214	2020/4/23	Hubei Provincial Hospital of TCM	Screening of proprietary Chinese medicines in convalescent rehabilitation of novel coronavirus pneumonia (COVID-19) convalescent patients
580	ChiCTR2000032233	2020/4/23	Xinhua Hospital Affiliated to Shanghai Jiao Tong University School of Medicine	Clinical study for the potential impact of novel coronavirus pneumonia (COVID-19) on endocrine system
581	ChiCTR2000032239	2020/4/23	Yangzhou Jiangdu People's Hospital	Epidemiological characteristics of 88 patients with new coronavirus pneumonia (COVID-19) and its impact on severe disease
582	ChiCTR2000032242	2020/4/24	The First Affiliated Hospital of Harbin Medical University	A multicenter, randomized, open, controlled trial for the efficacy and safety of oral kolimycin in the treatment of patients with new coronavirus pneumonia (CoVID-19)
583	ChiCTR2000032314	2020/4/25	West China Hospital, Sichuan University	A randomized clinical trial for the efficacy and safety of Aliskiren and Nifedipine in novel coronavirus pneumonia (COVID-19) patients with hypertension
584	ChiCTR2000032346	2020/4/26	The First Afflicated Hospital of Guangzhou University of Chinese Medicine	Lingnan Fire-Needle Therapy Improves the Quality of Life in General Population Under the Pandemic of novel coronavirus pneumonia (COVID-19): A Prospective, Randomized Controlled Trial
585	ChiCTR2000032368	2020/4/26	China-Japan Friendship Hospital	Investigation on the mental health status and intervention of the medical staff of the national rescue medical team in hubei province during the pandemic of novel coronavirus pneumonia (COVID-19)

586	ChiCTR2000032400	2020/4/27	Emergency department of Rujin Hospital, Shanghai Jiao Tong University School of Medicine	the efficacy and safety of high dose intravenous vitamin C in the treatment of novel coronavirus pneumonia (COVID-19): a prospective, randomize, controlled trial
587	ChiCTR2000032455	2020/4/28	Ningxia Ning'an Hospital	A study for the mental health status of novel coronavirus pneumonia (COVID-19) convalescent patients and first-line medical staff and Isolate residents and Hospital staffand the intervention strategy of psychological crisis in Ningxia
588	ChiCTR2000032456	2020/4/28	Shanghai Public Health Clinical Center	A Randomized Controlled Trial for the Effects of Low-Oxygen Consumption Instruction on the Prognosis of Patients with Novel Coronavirus Pneumonia (COVID-19)
589	ChiCTR2000032457	2020/4/28	West Campus of Union Hospital affiliated to Tongji Medical College of Huazhong University of Science and Technology	Application of pulmonary rehabilitation exercise in the treatment of novel coronavirus pneumonia (COVID-19) patients during convalescence period.
590	ChiCTR2000032458	2020/4/28	Affiliated Hospital of Zunyi Medical University	A medical records based retrospective study: Leukopenia is more common in asymptomatic patients less than 18 years old with novel coronavirus pneumonia (COVID-19) in convalescence
591	ChiCTR2000032459	2020/4/28	Beijing Institute of Biological Products Co., LTD. /He'nan Provincial Center for Disease Control and Prevention	A phase I/II clinical trial for inactivated novel coronavirus (2019-CoV) vaccine (Vero cells)
592	ChiCTR2000032460	2020/4/29	The First Affiliated Hospital of university of Science and Technology of China	Early warning model and new biomarker of severe novel coronavirus pneumonia (COVID-19)
593	ChiCTR2000032476	2020/4/29	Xiangya Hospital Central South University	Analysis of clinical features of novel coronavirus pneumonia (COVID-19): a medical records based study
594	ChiCTR2000032478	2020/4/29	Institute of Basic Research for Clinical Medicine, China Academy of Chinese Medical Sciences/Guang'anmen Hospital, China Academy of Chinese Medical Sciences	TCM Intervention study for novel coronavirus pneumonia (COVID-19) patient during home/designate-unit isolation based on community prevention and control

595	ChiCTR2000032487	2020/4/29	Shanghai Public Health Clinical Center	Study for using sulfate in the prevention and control of novel coronavirus pneumonia (COVID-19) in high and low prevalence communities
596	ChiCTR2000032514	2020/4/30	Zhongnan Hospital of Wuhan University	Direct evidence of rectal cancer patient infected with novel coronavirus pneumonia (COVID-19)
597	ChiCTR2000032527	2020/5/1	Eergency / Intensive Care Department of Tongji Hospital, Tongji Medical College,Huazhong University of Science and Technology	Developing and validating of clinical prediction model for novel coronavirus pneumonia (COVID-19) patients progressing to critical type, acute respiratory distress syndrome and death: a medical records based study
598	ChiCTR2000032574	2020/5/3	Ganzhou People's Hospital	Diagnostic value of chest CT in outpatient for novel coronavirus pneumonia (COVID-19) compared to RT-PCR
599	ChiCTR2000032666	2020/5/5	Union Hospital, Tongji Medical College, Huazhong University of Science and Technology	Follow-up study of pregnancy outcomes of novel coronavirus pneumonia (COVID-19) complicated in the first and second trimester
600	ChiCTR2000032686	2020/5/6	Huazhong University of Science and Technology	Study for the application of novel coronavirus pneumonia (COVID-19) intestinal tract toxicity in diagnosis and its prognostic effect
601	ChiCTR2000032716	2020/5/8	Ruijin Hospital Affiliated to Shanghai Jiaotong University School of Medicine	High dose intravenous vitamin C might be used as an important rescue therapy for aggravation of severe and critical novel coronavirus pneumonia (COVID-19) patients
602	ChiCTR2000032737	2020/5/8	The First Affiliated Hospital of Guangdong Pharmaceutical University	Clinical trial for the washed microbiota transplantation in the treatment of novel coronavirus pneumonia (COVID-19) patients suspected with gut microbiota dysbiosis
603	ChiCTR2000032743	2020/5/9	The First Affiliated Hospital of Dalian Medical University	The effectiveness of diaphragmatic breathing relaxation training for improving sleep quality among nursing staff during the novel coronavirus pneumonia (COVID-19) pandemic: a before and after study

604	ChiCTR2000032768	2020/5/9	Department of Nursing, Xiangya Third Hospital, Central South University	Correlation between novel coronavirus pneumonia (COVID-19) and mental health of breast cancer patients: a cross-sectional study in Hunan Province
605	ChiCTR2000032770	2020/5/10	Affiliated Hospital of Zunyi Medical University	Asymptomatic novel coronavirus pneumonia (COVID-19) patients Have Longer Treatment Cycle Than Mild and Moderate Patients: a medical records based study

605 clinical trials were registered nationwide for combating COVID-19. Registration ID, Record Date, Research Institutions and Trials Title were the mainly points with details here. In particular, 137 clinical trials were related to TCM, containing 76 definitely related to remedies or TCM patents, 32 with other TCM treatments (e.g., Shadowboxing, Liu-Zi-Jue Qigong, moxibustion, acupuncture, Triple Energizer Treatment, etc.) and 29 combining with Western Medicine; while the last were largely no concerned with TCM.
